# Searching for Novel COVID19 Treatments: Towards Covalent TMPRSS2 Inhibitors

**DOI:** 10.1002/cmdc.70524

**Published:** 2026-10-12

**Authors:** Marco Minneci, Barbara Farkaš, Adeyemi Rahman, Inga Nehlmeier, Amy Eichmann, Stefan Pöhlmann, Isabel Rozas

**Affiliations:** ^1^ School of Chemistry TBSI Trinity College Dublin Dublin Ireland; ^2^ Infection Biology Unit German Primate Center – Leibniz Institute for Primate Research Göttingen Germany

**Keywords:** covalent inhibition, guanidino derivatives, molecular modelling, SARS‐CoV‐2, TMPRSS2

## Abstract

We present the design, synthesis and biophysical and biochemical evaluation of new guanidinium derivatives with potential as covalent inhibitors of Transmembrane Protease Serine 2 (TMPRSS2), aiming to find therapies against COVID‐19. TMPRSS2 is a key protease for SARS‐CoV‐2 entry into host cells, making it a promising drug target since blocking viral entry, viral replication is stopped. We have designed five families of guanidinium derivatives with different scaffolds that would allow establishing covalent interactions with the target. The proposed structures were optimised for better binding and drug‐like properties. Docking studies showed that some of the new compounds had improved binding scores over nafamostat. Physicochemical and ADME properties analysis indicated that most of the compounds exhibit favourable drug‐like properties except for a small number that showed poor solubility or absorption. Different synthetic pathways were designed and optimised for the preparation of the five families, and the 40 compounds obtained were fully characterised. Unfortunately, in cell‐based assays, none of the new compounds prepared effectively blocked SARS‐CoV‐2 entry, suggesting that further optimisation is needed. Overall, the work presented expands the chemical space for targeting TMPRSS2 and provides valuable insights for future antiviral drug development.

## Graphical abstract

We designed, synthesised, and evaluated new guanidine derivatives as potential covalent inhibitors of TMPRSS2, a key protease for SARS‐CoV‐2 entry. Computational studies showed that some compounds have improved binding over nafamostat. However, experimentally none effectively blocked viral entry in cell assays, indicating further optimisation is needed.

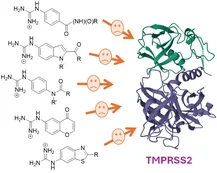

## 1 Introduction

Continuing with our studies aiming to find inhibitors of the type 2 transmembrane serine protease TMPRSS2as potential therapies against the SARS coronavirus 2 (SARS‐CoV‐2, pathogen responsible for the COVID‐19 pandemic [1]) [2], we now present the design, synthesis and biological evaluation of several families of potential TMPRSS2 covalent inhibitors. Still today, after 6 years of the pandemic declaration, only three antiviral drugs have been approved as COVID‐19 therapeutic agents, targeting the main viral protease M^Pro^ (nirmatrelvir—as part of paxlovid) or the viral RNA polymerase (remdesivir, molnupiravir) [3, 4, 5]. However, therapeutic approaches to treat COVID‐19 are still needed for immunocompromised patients, and this is reflected in the pipelines of many pharmaceutical companies, which are developing new small molecules mostly targeting M^Pro^ [6, 7]. It is well known that entry into host cells is a very good target to stop viral replication, and, hence, proteins involved in this step are very interesting targets for therapeutic development. In the case of SARS‐CoV‐2, their spike protein (S) requires proteolytic processing by furin at the S1/S2 site and the subsequent cleavage by TMPRSS2 at the S2ʹ site during cell entry [8, 9, 10, 11, 12]. For that reason, TMPRSS2 is an interesting target to develop therapies against COVID‐19 and other coronavirus diseases. Despite findings that omicron subvariants prefer cathepsin L over TMPRSS2 for entry into cell lines [8, 13, 14], inhibitor studies with primary respiratory epithelial cells and analyses of TMPRSS2 KO cells, organoids and mice h,ave shown that TMPRSS2 is essential to the dissemination and pathogenesis of pre‐omicron and omicron variants [15, 16, 17, 18, 19, 20, 21, 22]. It is significant to note that a TMPRSS2 inhibitor that was unable to interfere with SARS‐CoV‐2 infection upon systemic application in a rodent model was, however, able to very competently inhibit SARS‐CoV‐2 upon topical application [23], as well as other TMPRSS2 inhibitors topically tested in rodent models [24, 25, 26]. Additionally, another TMPRSS2 inhibitor, aprotinin, when administered by aerosol, was found to be clinically beneficial and well tolerated in COVID‐19 patients [27].

In our previous work [2], performed before the crystal structures of TMPRSS2 with camostat [28] or nafamostat [29] had been resolved, displaying their covalent mechanism of inhibition (Figure 1, up), we reported a family of diarylguanidino thioureas as potential competitive inhibitors of this protease (Figure 1, down). These compounds showed potential in the computational studies but did not exhibit the sought‐after TMPRSS2 inhibitory activity.

**FIGURE 1:**
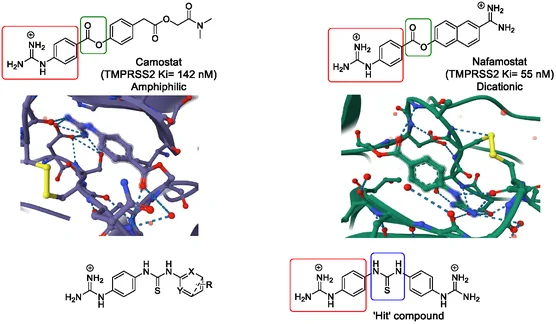
Upper panel: structure of the known TMPRSS2 inhibitors camostat and nafamostat with the corresponding inhibition constants and close‐up view of the catalytic pocket of TMPRSS2 with the benzoate guanidinium moiety of camostat (left) or nafamostat (right) covalently bound (taken from their crystal structures, 7Y0E [28] and 7MEQ [29]). Lower panel: general structure of the thioureas studied in our previous work [2], and structure of the ‘hit’ thiourea initially found.

Now, taking into account the proved covalent inhibition of TMPRSS2 by both guanidininium analogues (i.e., camostat and nafamostat, see Figure 1), we propose to computationally design, synthesise, and biochemically study five new families of guanidinium derivatives as potential covalent irreversible inhibitors (Figure 2).

**FIGURE 2:**
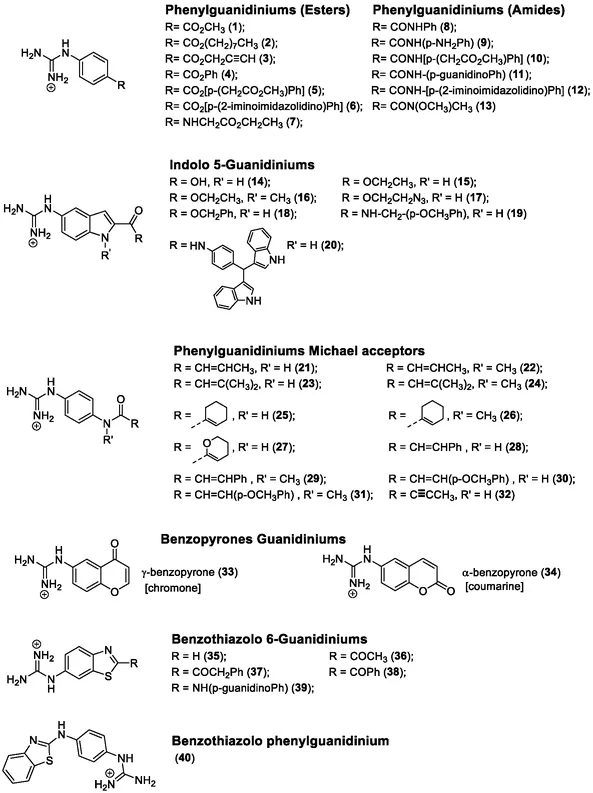
Structures proposed as potential irreversible inhibitors of TMPRSS2: phenylguanidiniums (esters **1–7** and amides **9–13**), indologuanidiniums (acid, esters and amides **14–20**), Michael acceptors (**21–32**), benzopyrones (**33–34**) and benzothiazole derivatives (**35–40**).

These novel guanidinium derivatives were selected considering different mechanisms of action that would result in the covalent inhibition of TMPRSS2. First, and following the example of camostat and nafamostat, we suggested guanidinophenyl esters and amides that could covalently bind to the Ser residue of the catalytic triad after being subjected to the protease activity, such as esters **1** to **7** and amides **8** to **13** (Figure 2). Additionally, we proposed guanidino benzimidazole derivatives carrying carboxylic acids, esters, and amides such as **14** to **20** (Figure 2) that could covalently bind to the mentioned Ser residue of the TMPRSS2 catalytic site. Furthermore, α,β‐unsaturated carbonyl phenylguanidinium derivatives (compounds **21** to **32**, Figure 2) as well as γ‐ and α‐benzopyrones (compounds **33** and **34**, Figure 2), which can act as electrophilic warheads enabling covalent Michael addition to the TMPRSS2 catalytic site, were devised. Besides, 6‐guanidino benzothiazole (compound **35**, Figure 2) and 6‐guanidino benzothiazole‐2‐carbonyl derivatives (compounds **36** to **38**, Figure 2) were considered since they could trap the Ser OH group. Ketobenzothiazoles have been shown to be effective inhibitors of TMPRSS2 and related proteases such as matriptase because this warhead covalently reacts with the hydroxyl group of a serine within the catalytic triad of the protease in a reversible manner, in contrast to nafamostat and camostat, which are irreversible inhibitors [24, 30, 31, 32, 33, 34]. Finally, compounds **39** and **40** were proposed to test the benzothiazole core as a warhead in potential non‐covalent inhibitors.

## 2 Results and Discussion

### 2.1 Structural Design

Structural design was guided by the crystal structure of TMPRSS2 with covalently bound nafamostat (7MEQ) and the non‐covalent precursor binding mode obtained by docking into an apo‐like protein structure recovered through the protein preparation after removal of the covalent ligand (Figure 3, left) since the molecule should be properly oriented within the catalytic site for the proteolytic activity to take place. Thus, three areas were identified for binding optimisation (Figure 3, right): (i) reactivity and positioning of the Ser441 warhead, (ii) affinity of the R^1^ group binding into the S1 sub‐pocket (Asp435‐Ser436‐Gly464), and (iii) specificity of the R^2^ group binding across the S2, S3, and S4 sub‐pockets (Gln299‐Lys300‐Lys342‐Gln438). First, for optimal covalent interaction, distance of the warhead to the Ser441 hydroxylic group is expected to be around 3–4 Å; this is conditioned by the non‐covalent binding mode, mainly established through the interactions of the R^2^ group, amongst which the most prominent are the π–π stacking with His296, and polar network with charged side chains of Glu299 and Lys300, whereas residue Lys342 provides a window of specificity for TMPRSS2 protease. Finally, the bulk of potency comes from the R^1^ group binding in the main S1 sub‐pocket, providing an opportunity for cooperative hydrogen bonding and salt bridging to side chains and backbone groups of Asp435, Ser436, and Gly464. From the binding mode of nafamostat, the phenyl‐guanidinium R^1^ group has a saturated coordination to the hydrogen‐bonding partners of the S1 sub‐pocket, with the phenyl ring acting as a spacer between the reactive warhead and guanidinium‐binding moiety without a clear interactive benefit.

**FIGURE 3:**
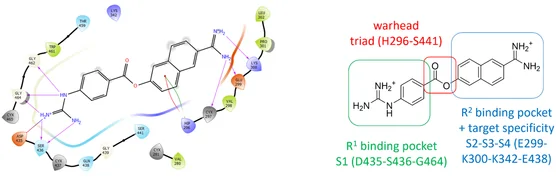
Left: interaction profile of non‐covalent docking of nafamostat into apo‐like TMPRSS2 crystal structure (7MEQ processed after removal of covalently bound nafamostat). Right: three areas for binding optimisation of covalent TMPRSS2 binders: R^1^ to bind the S1 pocket, R^2^ to bind the S2‐S3‐S4 pocket for target specificity and the warhead to target the catalytic triad.

The remaining portion of the molecule encompassing naphthyl sufficiently stacks with His296, while the amidinium substituent coordinates Glu299 and Lys300. In addition, there is room for improvement of physicochemical properties such as pK_aH_. Hence, structure‐guided design of this work mainly focused on the optimisation of the R^1^ group by changing the phenyl scaffold to spacer groups better suited to fill the sub‐pocket volume and providing improved hydrogen bond (HB) coordination of the guanidinium moiety, with the ultimate reward of a lower pK_aH_, variations of the warhead, and, to a lesser extent, modifications in the non‐covalent binding and specificity by targeting Lys342 as well as maximising interactions with cores and substituents of better flexibility than naphthyl‐amidinium. The warheads of this study included esters, amides, and Michael acceptors; a variety of R^1^ groups spanned (but were not limited to) indoles, benzothiazoles, or benzopyrones; and a selection of R^2^ groups were mostly phenyl guanidinium systems. The benzothiazole warhead had additional mechanistic implications in trapping serine residues.

### 2.2 Docking Studies

First, the fit of designed R^1^ groups was tested for both non‐covalent and post‐covalent modification binding modes, and the results are shown in Table S1. The non‐covalent precursor binding was sampled with the maintained naphthyl‐amidinium R^2^ core of nafamostat for a direct comparison, and a number of new R^1^ groups exceeded the nafamostat docking score, including indole groups incorporated in compounds **14**, **15**, **17**, **18**, **19**, and **20** and the methylated indole present in compound **16**. Improvement in the non‐covalent portion of the binding event is expected to be beneficial for faster and more efficient formation of the final covalently bound complex by improving the warhead‐to‐Ser441 geometry. Representative interaction diagrams for nafamostat and indole warhead analogues are depicted in Figure 4, where an indole bicycle fills the S1 sub‐pocket (Asp435‐Ser436‐Gly464) more effectively by either function as a better spacer for the improved positioning of the ester moiety or by forming an additional hydrogen bond with the backbone carbonyl of Gly464. The rest of the molecule is also seated deeper in the pocket with a stronger interaction network with the available polar residues (Glu299‐Lys300‐Lys342‐Gln438).

**FIGURE 4:**
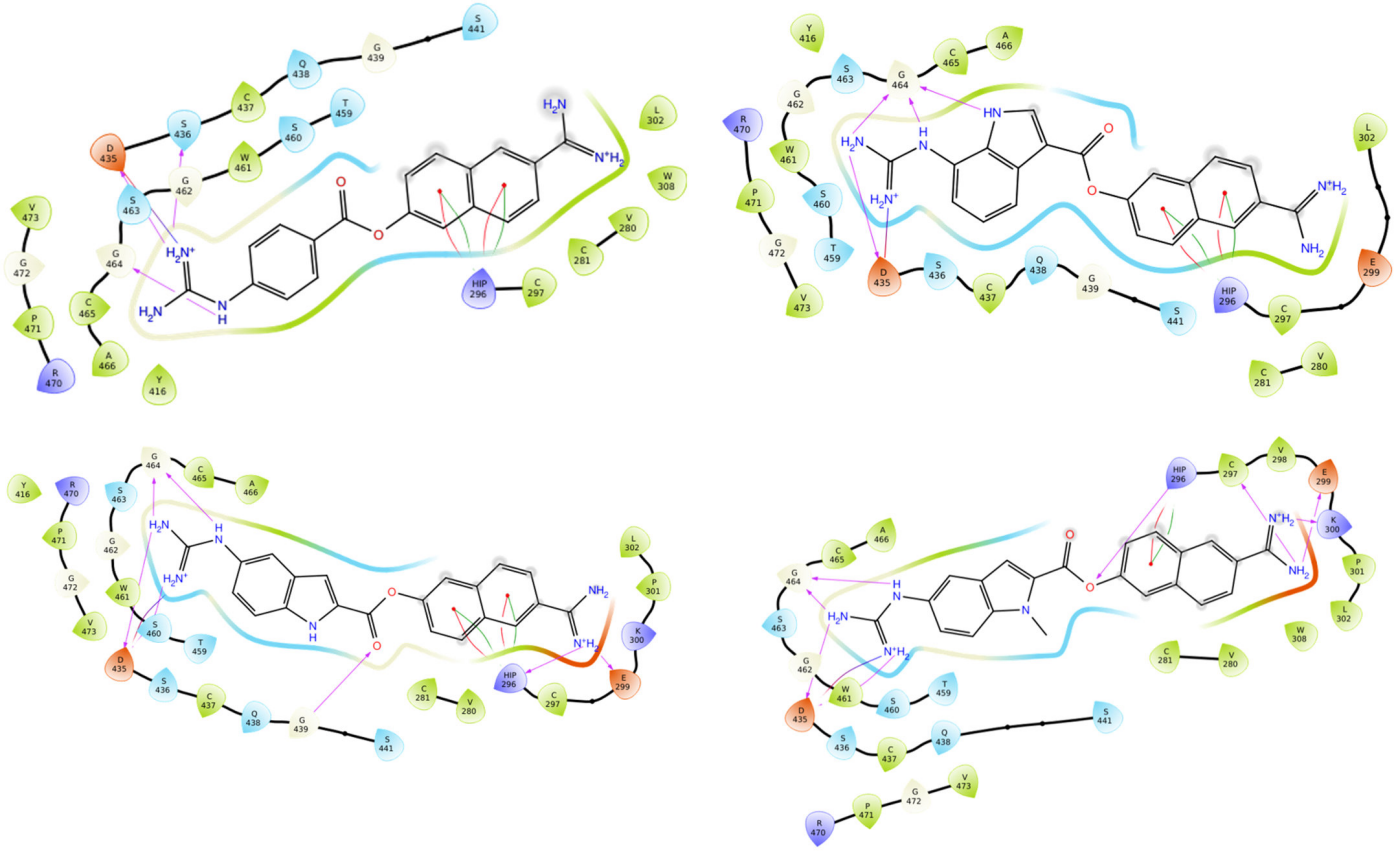
Ligand interaction diagrams for nafamostat and its indole warhead analogue as docked in the TMPRSS2 (7MEQ). Hydrogen bonds are shown directionally as magenta arrows, salt bridges are represented by red‐to‐blue gradient connection lines, and π‐cation and π–π stacking as red and green connection lines, respectively.

For the compounds synthesised in this work, the analysis of the docking scores and ligand–protein binding complexes is summarised in Table 1.

**TABLE 1:** Docking scores (kcal/mol) and main interactions to the protein obtained with the compounds proposed in this work, calculated with Glide XP. Aminoacids are represented by single letter codes (i.e., G, Gly; D, Asp; S, Ser; H, His).

#Cmpd	Glide XP	HBD–RES	HBA–RES	π–π	Salt bridge
**Nafamostat**	−6.8	G464, D435, S436		H296	D435
**1**	−6.6	G464, D435, S436	G439		D435
**2**	−6.0	G464, D435, S436	G439		D435
**3**	−4.7	G464, D435, S436	G439		D435
**4**	−6.8	G464, D435, S436	G439	H296	D435
**5**	−6.4	G464, D435, S436	G439	H296	D435
**6**	−6.3	G464, D435, S436	G439	H296	D435
**7**	−3.0	G464, D435, S436	G439		D435
**8**	−7.0	G464, D435, S436	G439, S441	H296	D435
**9**	−7.2	G464, D435, S436	G439	H296	D435
**10**	−7.6	G464, D435, S436	G439, S441	H296	D435
**11**	−7.0	G464, D435, S436	G439, S441	H296	D435
**12**	−7.2	G464, D435, S436	G439, S441	H296	D435
**13**	−6.7	G464, D435, S436	H296, S441		D435
**14**	−6.9	G464, D435, S436	G439		D435
**15**	−5.9	G464, D435, S436	H296, G439		D435
**16**	−7.6	G464, D435, S436	G439		D435
**17**	−5.2	G464, D435, S436	G439		D435
**18**	−6.6	G464, D435, S436	G439		D435
**19**	−5.9	G464, D435, S436	G439, S441		D435
**20**	−7.9	G464, D435, S436	H296, G439		D435
**21**	−3.3	G464, D435, S436	G439, S441		D435
**22**	−2.2	G464, D435, S436	G439, S441		D435
**23**	−3.4	G464, D435, S436	S441		D435
**24**	−3.4	G464, D435, S436	S441		D435
**25**	−3.7	G464, D435, S436	G439, S441		D435
**26**	−5.7	G464, D435, S436	G439		D435
**27**	−2.1	G464, D435, S436	G439		D435
**28**	−2.0	G464, D435, S436	G439	H296	D435
**29**	−2.2	G464, D435, S436	G439, S441		D435
**30**	−2.0	G464, D435, S436	G439	H296	D435
**31**	−2.2	G464, D435, S436	G439, S441	H296	D435
**32**	−3.6	G464, D435, S436	G439		D435
**33**	−7.1	G464, D435, S436	S441		D435
**34**	−6.9	G464, D435, S436	H296, S441		D435
**35**	−5.8	G464, D435, S436	G439		D435
**36**	−7.3	G464, D435, S436	G439		D435
**37**	−7.0	G464, D435, S436	G439	H296	D435
**38**	−6.6	G464, D435, S436			D435
**39**	−5.8	G464, D435, S436	S441		D435
**40**	−5.4	G464, D435, S436	G439		D435

Of the direct analogues of nafamostat’s original ester warhead to react with Ser441 (ester derivatives **1–6**), a significant reduction in the size of the R^2^ group shows, in general, a minimal loss in the overall docking score. A non‐cyclic extension between the phenyl‐guanidinium moiety and the reactive ester is predicted to be detrimental to the non‐covalent fit of compound **7**. Exchange of the ester for the amide in compounds **8–13** allows for a direct HB with Ser441, slightly increasing the favourability of the established precursor binding scores. Compounds with indole esters and amides in diverse flavours, **14–20**, show variations in docking scores with the size of the R^2^ group. A large selection of acrylamides (compounds **21–31**), as well as a single example of butynamide (compound **32**), scored significantly higher, and their R^2^ groups will likely need further optimisation for the protein side chain and backbone group coordination. The remaining compounds of this set are examples of the highest diversification from nafamostat. Two benzopyrones, **33** and **34**, with a noticeable reduction in size and lipophilicity, are still capable of recovering the main interaction points with the protein, resulting in a modest improvement of the docking scores but an appreciable increase in ligand efficiency. Of the three compounds with the ketobenzothiazole warhead targeted as a serine trap (compounds **35–37**), compounds **36** and **37** showcase comparable scores to nafamostat and provide an opportunity to circumnavigate the highly reactive ester functionality.

### 2.3 Theoretical Assessment of Pharmacokinetic (ADME) Properties

Drug‐likeness of the different families of compounds proposed was assessed using the SwissADME online tool [35] and will be discussed based on properties such as molecular weight (MW), number of rotatable bonds, hydrogen bond donors and acceptors (HBD and HBA), topological polar surface area (TPSA) [36], molar refractivity (MR), calculated lipophilicity (cLogP or Consensus logP, which is calculated as the average of the following five logP predictors: iLOGP: see ref. [37], XLOGP3: see ref. [38], WLOGP: see ref. [39], MLOGP: see ref. [40], SILICOS‐IT: see ref. [41]), water solubility [42], gastrointestinal absorption (GI), susceptibility to the P‐glycoprotein (ATP‐dependent membrane transporter working as an efflux pump to remove xenobiotics from cells, P‐gp), and the presence of any pan assay interference structures (PAINS [43]). Results are summarised in Table 2.

**TABLE 2:** Drug‐likeness metrics for the compounds’ libraries calculated with SwissADME. MW is expressed in g/mol, TPSA in Å^2^, MR in mL/mol, and water solubility in mg/mL. Those values that are considered ‘bad’ ADME indicators are shown in bold.

	MW	#Rot. bond	HBA/HBD	TPSA	MR	cLogP	Water solubility	GI/P‐gp	PAINS
**1**	194.21	3	2/3	90.37	51.94	0.95	2.72	High/no	0
**2**	292.40	**10**	2/3	90.37	85.58	3.31	**6.37 · 10** ^**−4**^	High/**yes**	0
**3**	218.23	3	2/3	90.37	59.71	1.05	4.27	High/no	0
**4**	256.28	3	2/3	90.37	72.06	1.98	8.63 · 10^−2^	High/no	0
**5**	328.34	6	4/3	116.67	87.92	1.98	9.53 · 10^−2^	High/no	0
**6**	340.38	4	2/**6**	126.46	101.79	1.27	2.71 · 10^−1^	High/**yes**	0
**7**	237.28	6	3/4	102.40	65.67	0.74	1.24	High/no	0
**8**	255.30	3	1/4	93.17	74.87	1.53	7.63 · 10^−1^	High/no	0
**9**	270.31	4	2/5	119.19	79.27	1.02	1.17	High/no	**anil.no. alk**
**10**	327.36	6	3/4	119.47	90.73	1.62	3.24 · 10^−1^	High/no	0
**11**	313.36	4	1/**7**	**157.24**	89.08	0.55	2.08	**Low**/no	0
**12**	339.39	4	1/7	129.26	104.60	0.87	9.43 · 10^−1^	High/**yes**	0
**13**	223.25	4	2/3	93.61	59.64	0.47	11.2	High/no	0
**14**	219.22	2	2/5	117.16	59.47	0.63	100	High/no	0
**15**	247.27	4	2/4	106.16	68.60	1.28	4.13 · 10^−1^	High/**yes**	0
**16**	261.30	5	2/3	95.30	73.50	1.32	8.13 · 10^−1^	High/**yes**	0
**17**	288.29	5	2/4	**155.91**	73.42	1.04	1.20 · 10^−2^	**Low**/no	**azo_A**
**18**	309.34	4	2/4	106.16	88.28	2.20	3.48 · 10^−2^	High/**yes**	0
**19**	338.38	6	2/5	118.19	96.49	1.78	8.91 · 10^−2^	High/**no**	0
**20**	**538.62**	6	1/7	**140.54**	**164.38**	**4.68**	**6.64 · 10** ^**−6**^	**Low**/no	0
**21**	219.26	4	1/4	93.17	64.11	0.94	3.49	High/no	0
**22**	233.29	5	1/3	84.38	69.01	1.14	3.61	High/no	0
**23**	233.29	5	1/4	93.17	68.92	1.26	8.24 · 10^−1^	High/no	0
**24**	247.32	5	1/3	84.38	73.82	1.46	8.69 · 10^−1^	High/no	0
**25**	259.33	3	1/4	93.17	76.42	1.65	2.98 · 10^−1^	High/**yes**	0
**26**	273.35	4	1/3	84.38	81.32	1.82	3.12 · 10^−1^	High/**yes**	0
**27**	261.30	3	2/4	102.40	72.69	1.01	1.33	High/**yes**	0
**28**	281.33	4	1/4	93.17	84.58	2.02	7.89 · 10^−2^	High/no	0
**29**	295.36	5	1/3	84.38	89.48	2.12	2.25 · 10^−1^	High/no	0
**30**	311.36	6	2/4	102.40	91.07	1.95	6.01 · 10^−2^	High/no	0
**31**	325.38	**7**	2/3	93.61	95.97	2.17	1.70 · 10^−1^	High/no	0
**32**	217.25	3	1/4	93.17	62.74	0.87	2.30	High/no	0
**33**	204.21	1	2/3	94.28	56.70	0.67	4.10	High/no	0
**34**	204.21	1	2/3	94.28	56.70	0.71	10.9	High/no	0
**35**	235.29	3	3/3	122.27	64.03	1.32	2.91 · 10^−1^	High/no	0
**36**	311.38	4	3/3	122.27	88.51	2.52	**8.63 · 10** ^**−3**^	High/**yes**	0
**37**	297.35	3	3/3	122.27	83.71	2.21	**6.97 · 10** ^**−3**^	High/**yes**	0
**38**	193.25	1	2/3	105.20	53.83	1.15	1.39	High/no	0
**39**	342.42	4	3/6	**181.30**	97.59	1.46	1.44 · 10^−2^	**Low**/no	0
**40**	284.36	3	3/3	117.23	83.38	2.47	1.55 · 10^−2^	High/**yes**	0

All compounds have MW < 350 with the exception of compound **20,** which have a MW > 500; however, this is not a problem since it is now well‐proved that many oral drugs are in the ‘beyond the rule of 5’ space [44]. Rigid drugs show better binding to the target (less energy penalty achieving the bioactive conformation) than those with many degrees of freedom (rotatable bonds); most of the molecules considered have <6 rotatable bonds, except compounds **2** and **31** with 10 and 7 rotatable bonds, respectively; in any case, these values are still within the limits established by Veber’s rules (≤10 rotatable bonds). Regarding the HBDs and HBAs, all compounds fulfil the rule of 5 (Ro5) in terms of HBAs but not in terms of HBDs (<10 HBA and <5 HBD); however, most of them follow Veber’s rule regarding the TPSA that should be <140 Å^2^ (except for compounds **11**, **17**, **20,** and **39**), and this parameter is more relevant concerning target‐ligand recognition. Molar refractivity (MR) is an indicator of molecular size and electron distribution, and values between 50 and 120 are considered favourable for drug‐like structures, more when combined with good MW and TPSA values. According to this, all molecules except compound **20** present optimal MR values. SwissADME provides several calculated values for logP (i.e., iLOGP, XLOGP3, WLOGP, MLOGP, and SILICO‐IT) [37, 38, 39, 40, 41], and the ‘Consensus LogP’ has been selected as it is calculated as the average value of the five values calculated for each compound.

All compounds proposed have cLogP within 0.47 and 3.31, indicating optimal lipophilicity for drug‐like activity (Table 2). Again, the exception is compound **20** with a cLogP of 4.68, which, even though larger than the optimal value of 3, is still within the Ro5. Water solubility is essential for good druggability since compounds need to be dissolved in the aqueous environment of the body, and thus, calculating the water solubility of potential drugs is important. SwissADME provides three different calculations for water solubility, and in the present work, the method developed by Ali has been chosen since the resulting values are systematically within those obtained with the other two methods. Most of the compounds were found to be ‘very soluble’ to ‘moderately soluble’, and only compounds **2**, **20**, **36,** and **37** were considered ‘poorly soluble'.

Additionally, the potential GI absorption and blood–brain barrier permeability (BBB, see Table 2) were assessed, and the results indicate that most of the compounds would be well absorbed in the GI (except for **11**, **17**, **20,** and **39**), but none of them will be able to cross the BBB. When a drug is a substrate for the P‐gp, it indicates that it is recognised by this efflux pump and transported out of the cells, limiting absorption and increasing excretion. Around 70% of the compounds studied were not found to be P‐gp substrates, and only compounds **2**, **6**, **12**, **15**, **16**, **18**, **19**, **25–27**, **36**, **37,** and **40** were considered substrates for this efflux pump. Finally, the presence of PAINS, which are disruptive functional groups with high non‐specificity to numerous biological targets, was assessed with SwissADME. PAINS were not found in most of the compounds proposed, except for compounds **9** and **17,** which showed ‘anil.no.alk’ and ‘azo_A’ sub‐structures, respectively. The ‘anil.no.alk’ code refers to the presence of certain anilines, nitro, or alkene groups, and in the case of compound **9**, there is a *p*‐aniline attached to the amino group of the amide linker. The ‘azo_A’ code refers to the presence of azo compounds, and compound **17** has an azide group.

Other parameters assessed by SwissADME were the bioavailability score (BS) and the potential of the compounds to be inhibitors of different cytochrome P450 isoenzymes (see Table S2). The BS was defined by Y. C. Martin as the probability that F (oral bioavailability) is >10% in rat [45]. According to the SwissADME calculations, all but one of the compounds studied have a BS = 0.55 (or 55%, which is considered an optimal threshold; see Table S1), indicating that they are expected to show ‘moderate to good’ oral absorption, meaning that they have a high probability of being absorbed and becoming biologically active. The exception was compound **20** with a BS = 0.17 (17%), suggesting a poor oral absorption for this compound. It has been estimated that between 50% and 90% of therapeutics are substrates of five cytochrome P450 isoforms (i.e., CYP1A2, CYP2C19, CYP2C9, CYP2D6, and CYP3A4, [46, 47]); therefore, inhibition of these isoenzymes could result in drug–drug interactions limiting the potential of these compounds as possible drugs because of toxic secondary effects; SwissADME provides a qualitative assessment of the possibility of the compounds studied to be substrates of the mentioned isoenzymes. In general, the compounds proposed are expected to have a good ADME/pharmacokinetic profile except for compound **21,** which showed bad results for most of the properties calculated (Table 2).

### 2.4 Synthesis

Preparation of the esters and amide derivatives of phenylguanidinium (**1–7** and **8–13**, respectively) involves the Boc‐guanidylation of the corresponding aniline, which is then Boc‐deprotected by treatment with TFA (Scheme 1).

**SCHEME 1:**
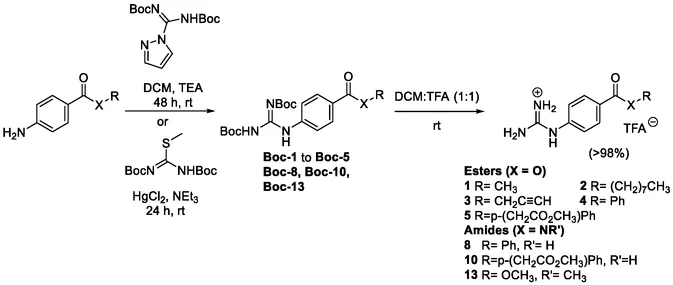
General route for the preparation of the trifluoroacetate salts of the esters **1–5** and amides **8, 10, 13**, by direct guanidylation of the corresponding anilines to produce the Boc‐protected guanidines (**Boc‐1** to **Boc‐5**, **Boc‐8**, **Boc‐10**, **Boc‐13**), followed by Boc‐deprotection.

The aniline precursor of compound **1** (methyl 4‐aminobenzoate) was commercially available and therefore, direct guanidylation with the Boc‐protected *N*‐amidinopyrazole derivative, as described in Scheme 1, followed by Boc‐deprotection yielded the final trifluoroacetate salt. In the case of compound **2**, 4‐‍aminobenzoic acid was treated with *n*‐octanol to yield the ester octyl 4‐aminobenzoate (Scheme S1), which was then treated with the *N*‐amidinopyrazole guanidylating agent to yield the Boc‐guanidine derivative (**Boc‐2**) and finally Boc‐deprotected with TFA (Scheme 1). The precursor anilines of compounds **3**, **4**, **8**, and **13** had a common starting material, i.e., 4‐nitrobenzoyl chloride (**41**, Scheme S1), which was prepared from 4‐‍nitrobenzoic acid and SOCl_2_. Then, it was treated either with propargylic alcohol or with *N*,*O*‐dimethylhydroxylammonium chloride to yield the corresponding nitro derivatives **42** and **43**, which were reduced to the corresponding anilino derivatives by means of Zinc dust (Scheme S1) and directly guanidylated (using the *N*‐amidinopyrazole route as described in Scheme 1) to yield compounds **Boc‐3** and **Boc‐13**. These compounds were treated with TFA to yield the final esters **3** and **13**. Alternatively, 4‐nitrobenzoyl chloride was treated with phenol or aniline to produce the benzyl ester and benzylamide nitro derivatives **44** and **45**, which were reduced with Zn to form the corresponding anilines (Scheme S1), then Boc‐guanidylated (yielding **Boc‐4** and **Boc‐8**) and Boc‐deprotected (*N*‐amidinopyrazole guanidylating route, Scheme 1) to yield compounds **4** and **8**. In the case of compounds **5** and **10**, the phenol and aniline required to form the corresponding ester and amide linkers were first prepared (Scheme S2); thus, methyl 2‐‍(4‐hydroxyphenyl)acetate was prepared by methyl esterification of 2‐(4‐hydroxyphenyl)acetic acid, while intermediate **46** was formed from 2‐(4‐nitrophenyl)acetic acid, and the nitro group was reduced to the corresponding aniline by hydrogenation. Next, the phenol and the aniline derivatives prepared were reacted with 4‐nitrobenzoyl chloride to produce ester **47** and amide **48**, and the nitro group of these compounds was hydrogenated, yielding the corresponding amines (Scheme S2), which were directly Boc‐guanidylated by the pseudothiourea route (yielding **Boc‐5** and **Boc‐10**) and Boc‐deprotected, as shown in Scheme 1, to produce the aimed salts.

Esters **6** and **7**, as well as benzamides **9**, **11** (previously synthesised by us [48]) and **12**, were prepared following different approaches. Thus, ethyl ester **7** was prepared by direct alkylation of the Boc‐protected 4‐aminophenylguanidinium, followed by deprotection with TFA (Scheme S3). Regarding amides **9** and **12**, it should be noted that we had previously prepared their analogues where the CONH link was swapped to NHCO [49]. Thus, for these compounds, the corresponding bis‐anilines were first mono‐guanidylated using the mercury‐supported Boc‐protected pseudo‐thiourea approach, yielding compounds **49** (amide‐linked) and **50** (ester‐linked), as shown in Scheme 2. Boc‐deprotection of **49** with TFA and ion interchange to hydrochloride using a basic resin resulted in salt **9**. Additionally, compound **50** was treated with Boc‐protected imidazolidin‐2‐one and mercury(II) chloride, followed by Boc deprotection with TFA and hydrochloride interchange, yielding compound **6** (Scheme 2). Following the same synthetic sequence, but starting from the mono‐cation **49**, hydrochloride **12** was obtained (Scheme 2). Repeating the reaction route with compound **50** and the pseudothiourea guanidylation yielded hydrochloride **11** (Scheme 2).

**SCHEME 2:**
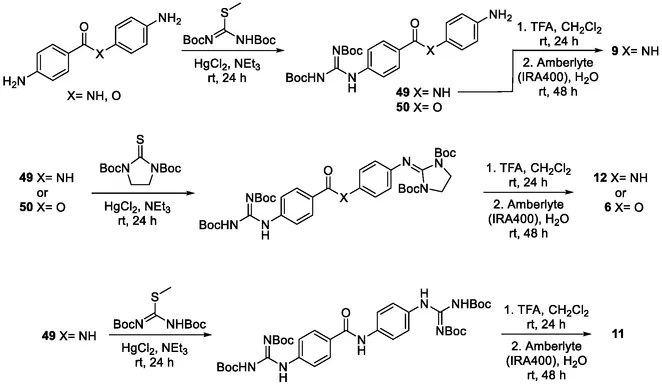
Preparation of hydrochloride salts of compounds **6**, **9**, **11** and **12** by successive guanidylations with thiourea‐like agents.

5‐Guanidinoindole derivatives carrying ester or amide warheads (compounds **14** to **20**) were prepared following the synthetic pathways shown in Scheme 3. Thus, the nitro group of ethyl 5‐‍nitro‐1H‐indole‐2‐carboxylate and its *N*‐methyl derivative was reduced to form the corresponding 5‐aminoindole derivatives, which were further guanidylated using the *N*‐amidinopyrazole route (yielding **Boc‐15** and **Boc‐16**) and Boc was deprotected to produce salts **15** and **16** (Scheme 3). Additionally, ethyl 5‐‍nitro‐1H‐indole‐2‐carboxylate was hydrolysed to yield the corresponding carboxylic acid **51**, and the nitro group was reduced to produce the corresponding 6‐aminoindole, which was directly guanidylated using the pseudothiourea route, yielding **52**. This Boc‐protected 5‐guanidinoindole **52** was the precursor not only for the trifluoroacetate **14** but also for the esters **17** and **18** and the amides **19** and **20** that were obtained after Boc deprotection of **Boc‐17** to **Boc‐20** (Scheme 3). Compounds **17** and **20** required the preparation of alcohol **53** and aniline **54**, respectively, as shown in Scheme S4.

**SCHEME 3:**
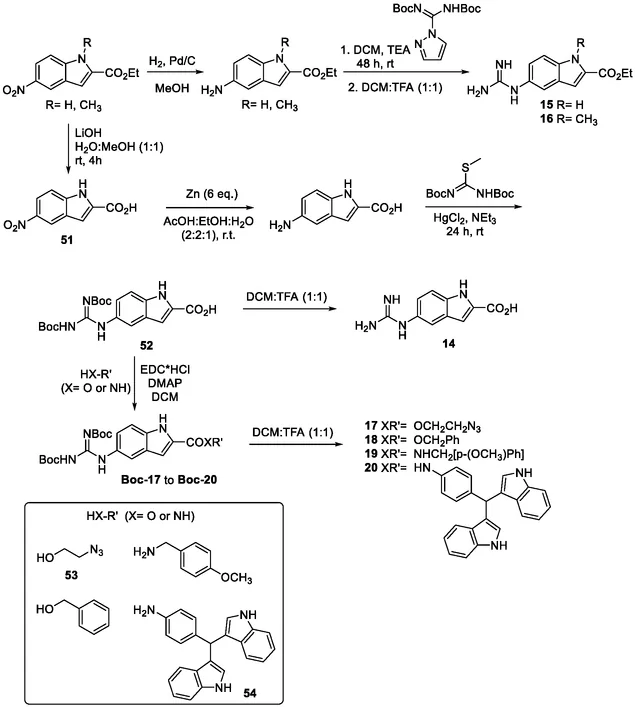
Preparation of indole trifluoroacetate salts **14** to **20** starting from ethyl 5‐nitro‐1H‐indole‐2‐carboxylate and its *N*‐methyl derivative.

Michael acceptor derivatives (**21–** **32**) were prepared, as shown in Scheme 4. Thus, preparation of compounds **21**, **23**, **25**, **27**, **28,** and **30** started by reacting mono‐Boc‐protected 4‐aminoaniline with the corresponding acrylic acid derivative or with but‐2‐ynoic acid, followed by Boc‐deprotection of the amino group and guanidylation according to the *N*‐amidinopyrazole route. In the case of the *N*‐methyl amido derivatives **22**, **24**, **26**, **29,** and **31**, their preparation started by the reduction of the nitro group of *N*‐‍methyl‐4‐nitroaniline, followed by guanidylation using the *N*‐amidinopyrazole agent to yield **55**; this was followed by treatment with the corresponding acrylic acid, yielding compounds **56** to **60** that were Boc‐deprotected with TFA (Scheme 4).

**SCHEME 4:**
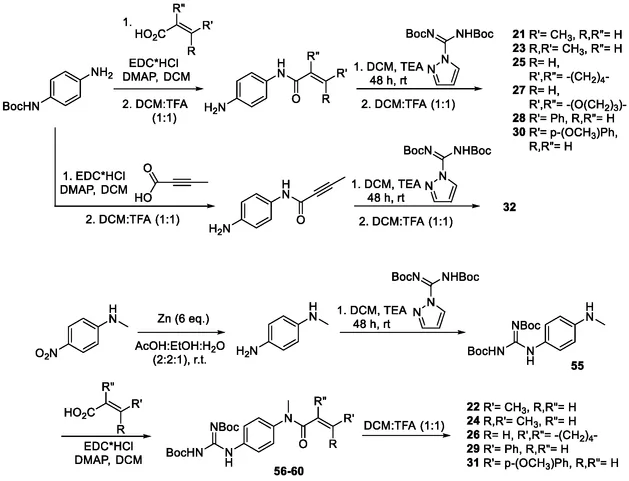
Preparation of hydrochloride salts of compounds **22–** **33** by amide formation and guanidylations with the amidinopyrazole derivative.

Benzopyrones **33** and **34** were easily prepared by the reduction of the corresponding nitro derivatives and further guanidylation and Boc‐deprotection, as shown in Scheme S5. Guanidino acyl benzothiazole warhead derivatives **35**, **36**, **37,** and **38** were prepared from 5‐aminobenzothiazole. Guanidylation of the latter yielded intermediate **61,** and deprotonation of its thiazole moiety was achieved by treatment with *t*‐BuLi. Although the C─H is relatively acid (pK_a_ ≈ 25), *t*‐BuLi was necessary since any nucleophilic base would have attacked the guanidinyl system, destroying the molecule. Addition of the desired Weinreb amide led to the formation of compounds **62–64**, which were Boc‐deprotected with TFA to yield final salts **35** **–37**. Compound **38** was obtained by the direct deprotection of **61**.

Considering the potential of the benzothiazole core, phenylguanidinium derivatives of this heteroaromatic system were also prepared (Scheme 5) to probe the binding site of the TMPRSS2 enzyme by non‐covalent reversible inhibition. Thus, compounds **39** and **40** were prepared following a similar synthetic pathway starting with the reaction of 2‐chloro‐6‐nitrobenzo[*d*]thiazole or 2‐chloro‐benzo[d]thiazole, respectively, with *p*‐nitroaniline, followed by the reduction of the nitro group with Zn. Then, guanidylation of the anilino intermediates, followed by Boc‐deprotection, yielded the corresponding salts (Scheme S6).

**SCHEME 5:**
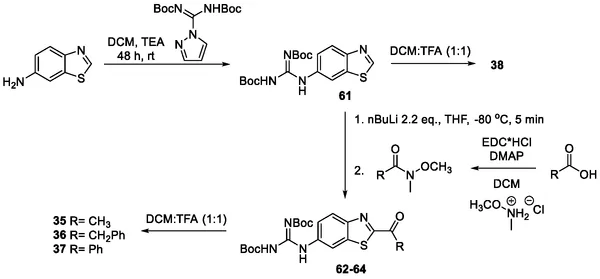
Preparation of trifluoroacetate salts of compounds **35–38** implementing a Weinreb amide strategy.

### 2.5 Biophysical and Biochemical Studies

#### 2.5.1 DNA UV‐Thermal Denaturation

Considering the similarity of some of the compounds prepared with known DNA minor groove binders (MGBs) and the potential toxicity associated, the ability of these guanidinium derivatives to bind to DNA was tested. UV‐‍Thermal DNA denaturation experiments are well known to inform on the affinity of compounds towards DNA (i.e. the thermal melting temperature, Δ*T*_m_, which is the difference in melting between that of isolated DNA and that of the DNA–ligand complex). Compound **11** had already been reported by us to be a strong DNA MGB with an Δ*T*_m_ = 40.1 °C using an oligo rich in AT pairs [poly(dA•dT)_2_] [48]. We had previously reported the synthesis and DNA binding of the NHCO analogues of compounds **9** and **12** [49]; thus, the NHCO analogue of **9** exhibited an Δ*T*_m_ = 1 °C using salmon testes DNA and the NHCO analogue of **12** exhibited an Δ*T*_m_ = 26.1 °C using [poly(dA•dT)_2_]. For this study, we chose a representative for each family to test their potential as DNA binders; thus, compounds **2**, **15**, **30,** and **35** were studied for their affinity to unspecific salmon testis DNA and in all cases very small to null Δ*T*_m_ values were found (0.2, 0.4, 1.0, and 0.4 °C, respectively; Figure S1), indicating that these particular structures are not expected to bind to DNA.

Therefore, compound **11** would not be a good drug candidate for its strong DNA binding, as well as probably compound **12**, because its NHCO analogue was also a strong MGB. The rest of the compounds tested as representatives of the different families studied showed no DNA toxicity.

#### 2.5.2 Cell Viability

As sampling tests, compounds of each of the families prepared (**2**, **15**, **30,** and **35**) were tested to assess whether they can affect or not the viability of healthy human cells; hence, the chosen assay is the well‐established cell viability AlamarBlue in vitro assay, and the breast MCF‐10A cells were selected as a model of non‐cancerous cells. The dose‐dependent cell viability curves obtained are shown in Figure S2, showing that no effect on the viability of the MCF‐10A cell was induced by these compounds.

#### 2.5.3 Inhibition of SARS‐CoV‐2 Entry Study

Guanidinium salts **1–** **40** were evaluated for their ability to prevent SARS‐CoV‐2 from entering cells (Figures 5 and S3–S17). With this in mind, pseudotyped particles bearing the SARS‐CoV‐2 B.1 S protein (amino acid sequence identical to that of the Wuhan‐Hu‐1 S protein but includes mutation D614G) were utilised. These particles are safe and mimic key aspects of SARS‐CoV‐2 cell entry and its inhibition [50]. Additionally, pseudotyped particles bearing the glycoprotein of vesicular stomatitis virus (VSV‐G) were employed as controls since VSV‐G‐driven entry is independent of ACE2 and TMPRSS2. As target cells, Calu‐3 human lung cells, which express endogenous TMPRSS2 and allow for TMPRSS2‐dependent cell entry, and Vero76 African green monkey kidney cells, which do not express TMPRSS2 but allow for cathepsin L‐dependent entry, were used [51]. Lastly, the serine protease inhibitor camostat was employed to test for TMPRSS2‐dependent viral entry, and chloroquine, which elevates endosomal pH, was used to test for cathepsin L‐dependent entry since low pH is required for cathepsin L activity. Furthermore, chloroquine is known to block entry of viruses such as VSV that require low pH for the triggering of their glycoproteins.

**FIGURE 5:**
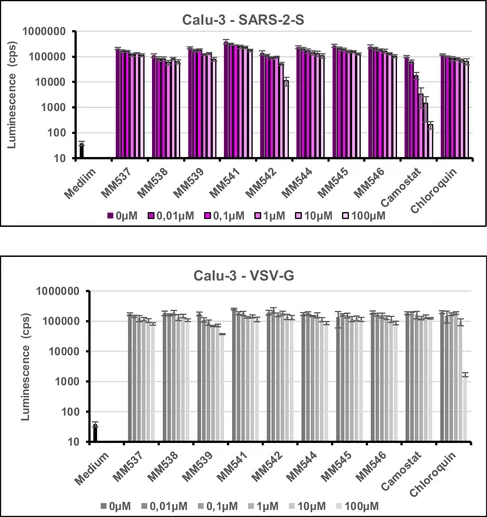
Calu‐3 cells seeded in 96‐well plates were incubated with the indicated compounds at the indicated concentrations and inoculated with vesicular stomatitis virus (VSV) pseudoparticles bearing SARS‐CoV‐2 S protein (SARS‐2‐S, top panel) or the VSV glycoprotein (VSV‐G, bottom panel) or control inoculated with medium (black bar). Luciferase activity in cell lysates was determined at 16–18 h post infection. The results of a single experiment carried out with technical quadruplicates are shown. Error bars indicate SD. Compounds **MM537** (**16**), **MM538** (**8**), **MM539** (**36**), **MM541** (**21**), **MM542** (**38**), **MM544** (**19**), **MM545** (**41**) and **MM546** (**37**) jointly with camostat and chloroquine were analysed in separate experiments.

As expected, entry into Vero76 cells driven by the SARS‐CoV‐2 S protein and VSV‐G was not inhibited by camostat but was reduced by the highest concentration of chloroquine tested (Figures 5 (up) and S3). Additionally, SARS‐CoV‐2 S protein‐mediated entry into Calu‐3 cells was inhibited by camostat but not chloroquine in a dose‐dependent manner, while the opposite was observed for VSV‐G‐driven entry (Figures 5 (down) and S4). On the contrary, none of the compounds tested reduced SARS‐CoV‐2 S protein or VSV‐G‐driven entry into Vero76 (TMPRSS2‐independent SARS‐CoV‐2 S protein‐driven entry, Figures S3, S5, S7, S9, S11, S14, and S16) or Calu‐3 cells (TMPRSS2‐dependent SARS‐CoV‐2 S protein‐driven entry, Figures 5, S4, S6, S8, S10, S12, S13, S15, and S17), indicating that they were unable to inhibit TMPRSS2 activity or failed to reduce TMPRSS2 activity to the extent required for antiviral activity.

## 3 Conclusions

In this study, we have successfully designed and synthesised five families of guanidinium derivatives as potential covalent inhibitors of TMPRSS2, inspired by the known inhibitors camostat and nafamostat. Several structural scaffolds such as phenyl esters and amides, indoles, Michael acceptors, benzopyrones, and benzothiazoles were used to explore different mechanism of action and their ability to form covalent interactions with the target. Of the 41 compounds proposed, several showed improved docking scores compared to nafamostat, and most of them displayed favourable drug‐like properties (i.e., good solubility, low toxicity, and ADME properties). Different synthetic routes were put in place to prepare all these guanidinium derivatives in good yields and with minimum side‐product formation. Preliminary toxicity assessments were carried out to measure potential DNA binding and cytotoxicity against ‘normal’ cells, such as MCF‐10. Despite the promising computational results, none of the new compounds effectively blocked SARS‐CoV‐2 entry in cell‐based assays. Although activity against recombinant TMPRSS2 was not examined, a limitation of the present study, these results indicate that further optimisation is needed to achieve antiviral efficacy.

Despite failing to find new TMPRSS2 inhibitors as potential antiviral agents for COVID19 treatment, these results provide valuable insights, as they can be used as a negative control for further machine learning studies that require diverse structure–activity information; this means including not only very active compounds but also compounds that, even though predicted to bind to TMPRSS2 by computational studies, did not show the expected activity. Our results provide valuable insights for future antiviral drug development.

## 4 Experimental Section

### 4.1 Computational Studies

Details of the docking of all proposed compounds can be found in the Supporting Information. In this modelling step, all compounds were prepared using LigPrep with Epik [52] at physiological conditions (pH = 7.0 ± 0.4) to establish the favoured tautomerization and protonation states, while the crystal structure of TMPRSS2 with covalently bound nafamostat (7MEQ) was pre‐processed via Protein Preparation Wizard [53] as provided in the Schrodinger Suite (version 2020‐1) and covalently modified serine residue recovered into its natural state with covalent ligand removed and protein recovered to an apo‐like state which was further used to dock non‐covalently nafamostat and subsequent analogues library. Molecular docking was performed using Glide [54, 55] with the extra precision (XP) scoring function and induced fit docking (IFD) [56] to account for any protein movement in the near vicinity of the bound ligand.

The drug‐likeness was probed in the form of ADME profiles using the online SwissADME tool [35], focusing on the molecular weight (MW), number of hydrogen bond donors and acceptors (HBD and HBA), polar surface area (TPSA), ligand lipophilicity (Consensus logP), molar refractivity (MR), water solubility, gastrointestinal absorption (GI), susceptibility to the P‐gp, and the presence of any pan assay interference structures (PAINS), as well as the bioavailability score (BS) and potential of the compounds to be inhibitors of different cytochrome P450 isoenzymes.

### 4.2 Synthesis: General Procedures for the Boc‐Deprotection of the Guanidinium Derivatives Prepared

The corresponding Boc‐protected guanidines (1eq.) were dissolved in a 1:1 TFA:DCM solution (0.05 M). The reaction was allowed to proceed for 4 h at room temperature under a N_2_ atm. The crude product was concentrated in vacuo. The excess TFA was removed with Et_2_O. Deprotected salts were obtained in quantitative yields.

### 4.3 Biochemical Experiments

#### 4.3.1 Cell Culture

All cell cultures were maintained at 37 °C in a humidified incubator with 5% CO_2_. 293T cells (human, female, kidney; ACC‐635, DSMZ; RRID: CVCL_0063) and Vero76 cells (African green monkey, female, kidney; CRL‐1586, ATCC; RRID: CVCL_0574; kindly provided by Andrea Maisner) were cultured in Dulbecco’s Modified Eagle Medium (DMEM; PAN‐Biotech) supplemented with 10% fetal bovine serum (FBS; Biochrom) and 1% penicillin–‍streptomycin (PAN‐Biotech). Calu‐3 cells (human, male, lung; HTB‐55, ATCC; RRID: CVCL_0609; kindly provided by Stephan Ludwig) were maintained in DMEM/F‐12 with Nutrient Mix (Thermo Fisher Scientific) supplemented with 10% FBS, 1% penicillin–streptomycin, 1% non‐essential amino acids, and 1 mM sodium pyruvate. Cell line authentication was performed via short tandem repeat (STR) profiling or PCR amplification and sequencing of a cytochrome c oxidase gene fragment, along with assessment of cell morphology and growth behaviour. All cell lines were routinely tested for mycoplasma contamination. 293T cells were transfected using a calcium phosphate precipitation method.

#### 4.3.2 Production of Pseudotypes and Inhibition of Viral Entry

Pseudotyped virus particles were produced as documented previously [10]. In brief, 293T cells were transfected with plasmids encoding the SARS‐CoV‐2 S protein or VSV‐G. After 24 h, the medium was removed and the cells inoculated with VSV‐G‐trans‐complemented VSV*Δ*G* (FLuc) (kindly provided by Gert Zimmer [57]). Following a 1 h incubation at 37 °C, cells were washed, and SARS‐CoV‐2 S protein‐expressing cells were cultured in medium containing anti‐VSV‐G antibody (I1‐hybridoma supernatant; ATCC CRL‐2700). After 16–18 h, the supernatants were harvested, clarified by centrifugation (4000×*g*, 10 min), aliquoted, and stored at −80 °C.

Vero76 (African green monkey, kidney) and Calu‐3 (human, lung) cells were seeded in 96‐well plates. The following day, the culture medium was removed, and 50 µL of fresh medium containing the test compounds at the concentrations indicated in the Figures was added, followed by incubation of the cells for 120 min at 37 °C. Camostat (Tocris), a serine protease inhibitor active against TMPRSS2, the cellular protease that activates the SARS‐CoV‐2 spike protein (SARS‐2‐S) for entry into Calu‐3 cells [8, 51], and chloroquine (Tocris), a compound that elevates endo/lysosomal pH and thereby inhibits the activity of cathepsin L, the cysteine protease activating SARS‐2‐S for endosomal entry into Vero cells [8, 51], were used as controls. It should be noted that chloroquine also inhibits cell entry driven by the vesicular stomatitis virus glycoprotein (VSV‐G), which initiates membrane fusion upon exposure to low pH. Subsequently, 50 µL of vesicular stomatitis virus (VSV)‐based pseudotypes encoding luciferase and harbouring either SARS‐2‐S or VSV‐G was added, and the cells were incubated at 37 °C for 16–18 h. Finally, the medium was removed, cells were lysed using 0.5% Tergitol 15‐S‐9 (Carl Roth) for 30 min at room temperature, and luciferase activity in cell lysates was determined using a commercial luciferase substrate (Beetle‐Juice, PJK). Luminescence was measured using a Hidex Sense Plate Luminometer (Hidex).

## Funding

This study was supported by Science Foundation Ireland/Enterprise Ireland/Industrial Development Agency COVID‐19 Rapid Response Funding Call (Grant 20/COV/0155), Science Foundation Ireland (Grant 20/FFP‐P/8760).

## Conflicts of Interest

The authors declare no conflicts of interest.

## Supporting information

Computational details; synthetic methods’ details and characterisation data for all compounds prepared; UV‐thermal DNA denaturation details; cell viability assays (i.e. AlamarBlue) with MCF10 cell line experimental conditions; biochemical experiments protocols and results; ^1^H NMR and ^13^C NMR spectra; and SwissADME results for all compounds here studied.

## Data Availability

The data that support the findings of this study are included in the Supplemental Information document and available from the corresponding author upon reasonable request.
