# Supplementary material for: Searching for Novel COVID19 Treatments: Towards Covalent TMPRSS2 Inhibitors
Source: ChemMedChem. 2026 Oct 12;21(19):e70524. doi: 10.1002/cmdc.70524 (PMC13659029; doi:10.1002/cmdc.70524)
Supplement: Supplementary file 1 [file CMDC-21-e70524-s001.pdf]

Supplementary Information

Searching for novel COVID19 treatments: aiming for covalent TMPRSS2 inhibitors

Marco Minecci,[a],§,‡ Barbara Farkaš,[a],#,‡ Adeyemi Rahman,[a],¶ Amy Kempf,[b] Inga Nehlmeier,[b] Stefan Pöhlmann[b] and Isabel Rozas[a],*

[a]*School of Chemistry, TBSI, Trinity College Dublin, The University of Dublin, 152-160 Pearse Street, Dublin D02 R590, Ireland.* [b]*Infection Biology Unit, German Primate Center, Kellnerweg 4, 37077 Göttingen, Germany.*

Table of contents

S1. Computational details S2

S2. Synthetic details S4

S3. UV thermal denaturation experiments S111

S4. Cell viability assays S112

S5. Analysis of SARS-CoV-2 spike protein driven entry into cells and its inhibition S113

S6. ^{1}H NMR and ^{13}C NMR spectra of relevant compounds S128

S7. SwissADME results for all compounds modelled S216

S8. References S257

S1. Computational details

S1.1. Molecular docking

Molecular docking studies were performed using Glide with extra precision (XP) scoring function[1] and CovDock[2] for the covalent docking and induced fit docking (IFD)[3] to account for any protein movement in the near vicinity of the bound ligand, both built in the Schrodinger Suite (version 2020-1). Compounds were prepared using LigPrep with Epik[4] at physiological conditions (pH 7.0 ±0.4) to determine the favoured tautomerisation and protonation states, while TMPRSS2 was pre-processed via Protein Preparation Wizard[5] with the covalently modified serine residue recovered into its natural state.

Table S1. Docking scores of compounds with different R^1 groups attached to the non-covalent naphthyl-amidinium core of nafamostat as well as of two benzopyrone derivatives. Predicted pK_{aH} values for the guanidinium group are also provided.

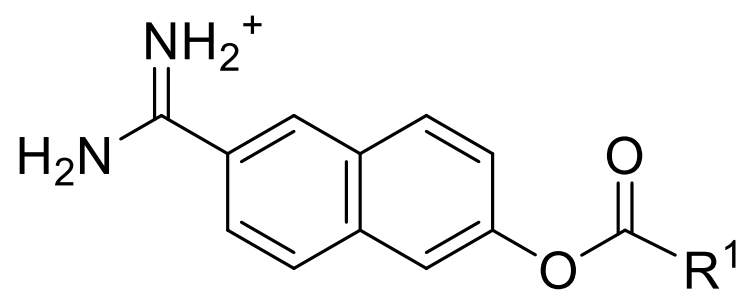

R^1	Glide XP	Glide covalent	pK_{aH}
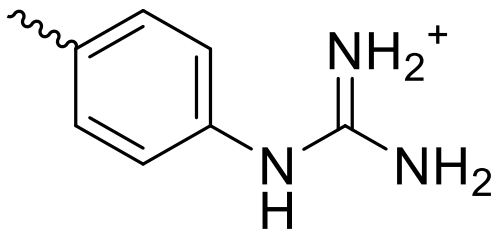 Nafamostat	-6.8	-7.1	7.6
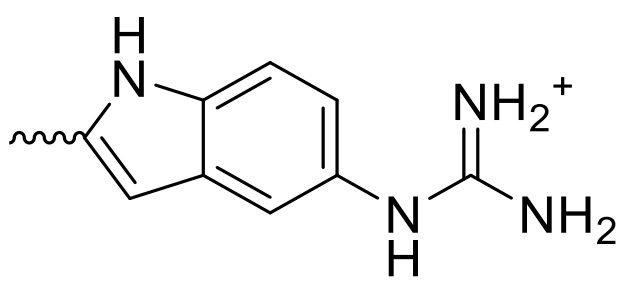	-7.3	-7.7	7.9
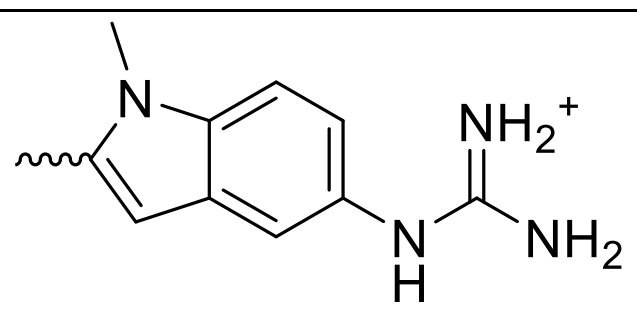	-8.2	-7.0	7.7
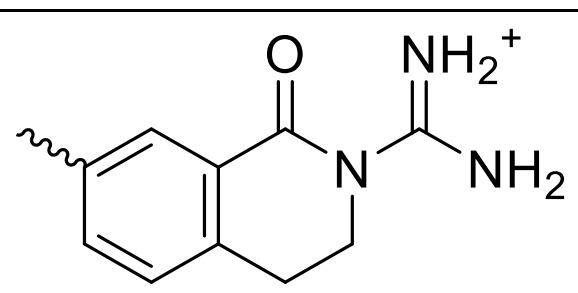	-7.0	-7.5	6.2
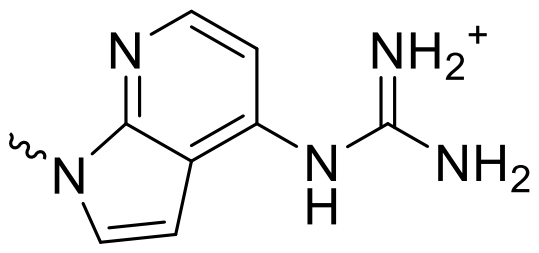	-6.4	-7.3	-7.7
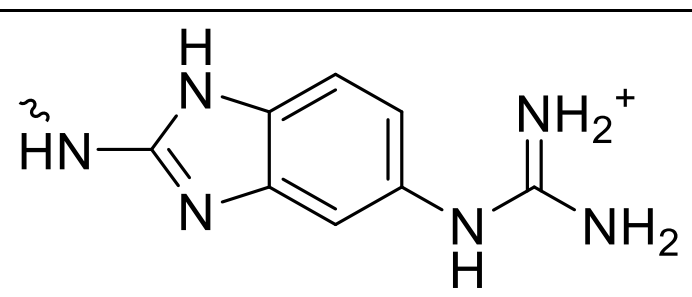	-6.4	-7.7	8.3

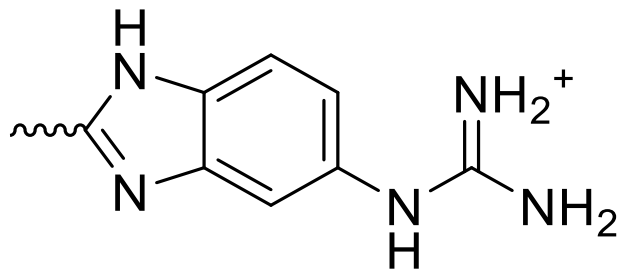	-4.6	-7.5	7.6
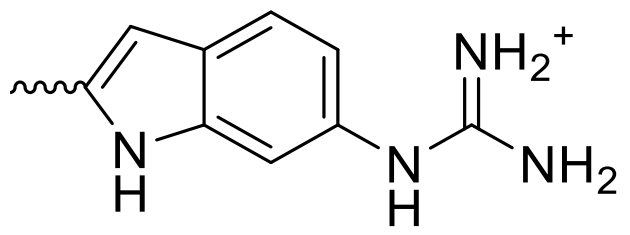	-6.8	-6.7	7.8
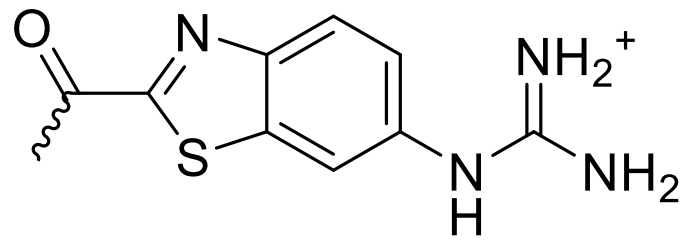 *ketone warhead instead of an ester	-6.7	-7.7	7.5
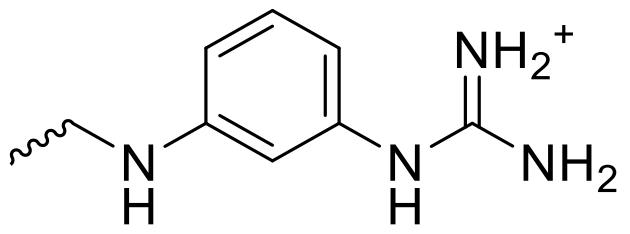	-6.2	-6.1	10.6
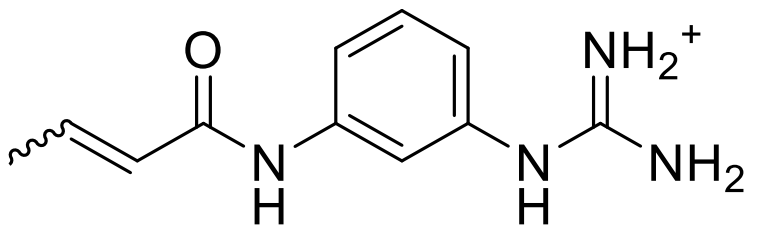 *acrylamide warhead instead of an ester	-3.3	-5.5	8.8
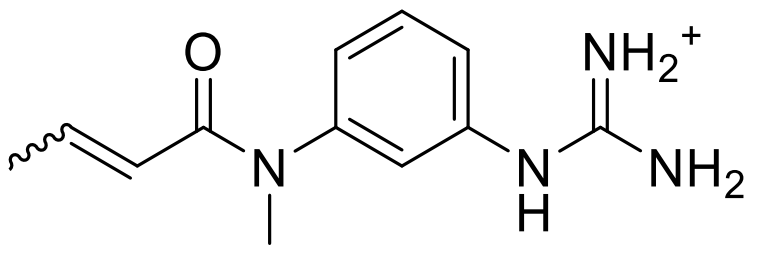 *acrylamide warhead instead of an ester	-4.6	-5.0	8.2
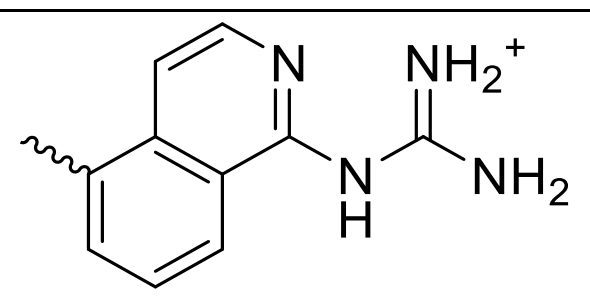	-5.7	-7.7	6.6
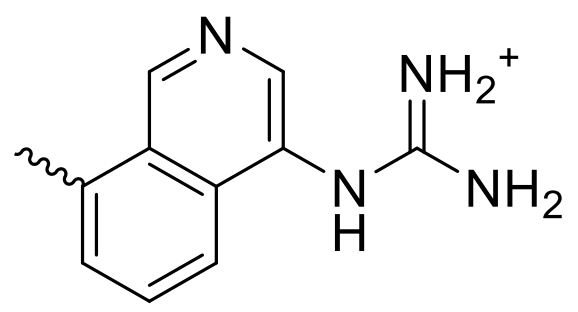	-5.5	-7.8	7.2
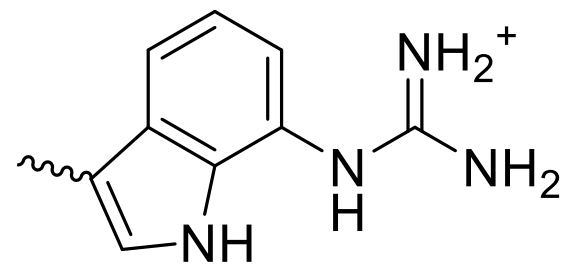	-7.6	-8.0	7.5
**Whole molecule**	**Glide XP**	**Glide covalent**	**pK_{aH}**
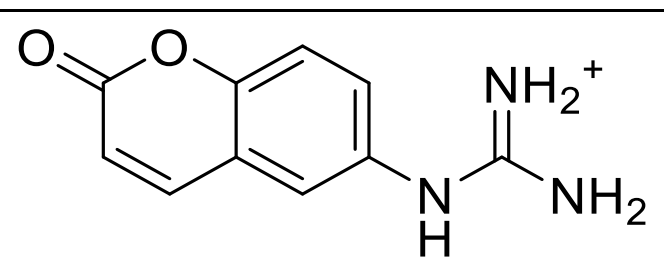	N/A	-7.6	9.8
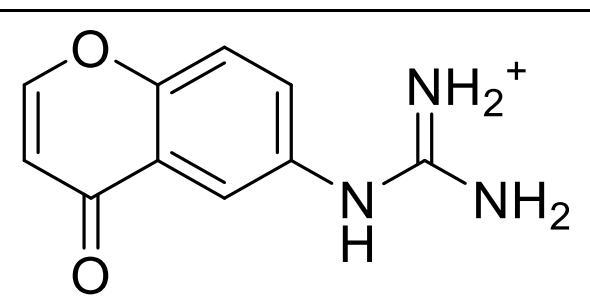	N/A	-7.0	9.7

S2. Synthetic details

S2.1. Materials and Methods

All commercial chemicals were obtained from either Sigma-Aldrich, Fischer Scientific or Fluorochem and used without further purification. Deuterated solvents for NMR analysis were purchased from Fischer Scientific. Column chromatography was performed using Sigma-Aldrich silica gel 100-200 mesh. Solvents for synthesis purposes were used at HPLC grade. Analytical TLC was performed using Merck Kieselgel 60 F-254 silica gel plates. Visualisation was completed using a UV light (254 nm). NMR spectra were recorded on Bruker DPX-400 Advance and Bruker AV-600 spectrometers, operating on either 400.13 and 600.1 MHz for ^{1}H NMR; 100.6 and 150.9 MHz for ^{13}C NMR. Shifts are reported as referenced to the internal solvent signal. NMR data was processed using TopSpin, Bruker's NMR data analysis software. HRMS spectra were measured on a Micromass LCT electrospray TOF instrument with a WATERS 2690 autosampler and methanol/acetonitrile as carrier solvent.

2.2. Schemes for the preparation of starting anilines and some guanidine derivatives

Scheme S1.- Preparation of anilines precursors of guanidinium derivatives **3**, **4**, **5**, **9** and **14**

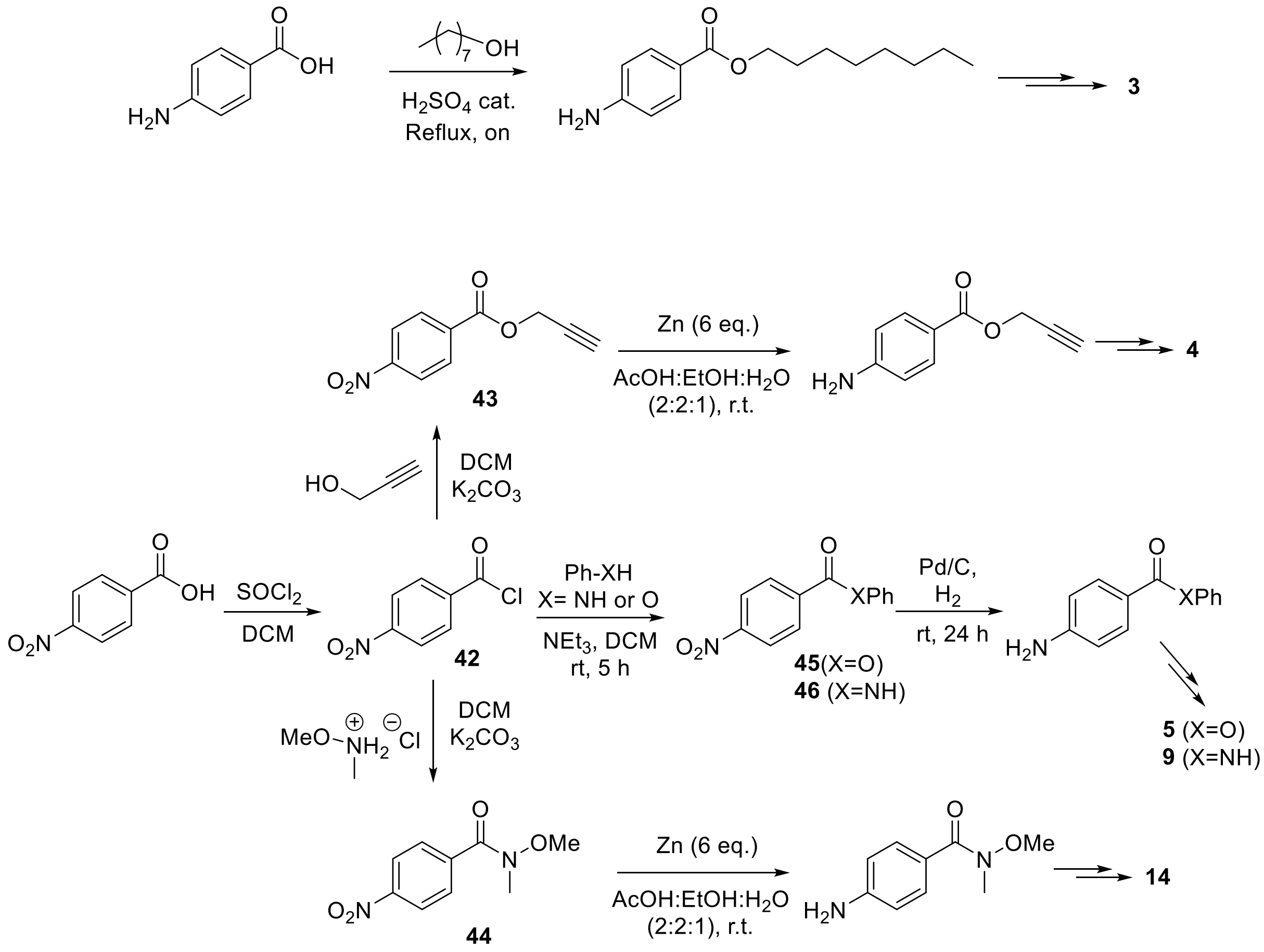

Scheme S2. Preparation of the aniline precursors of compounds **6** and **11**

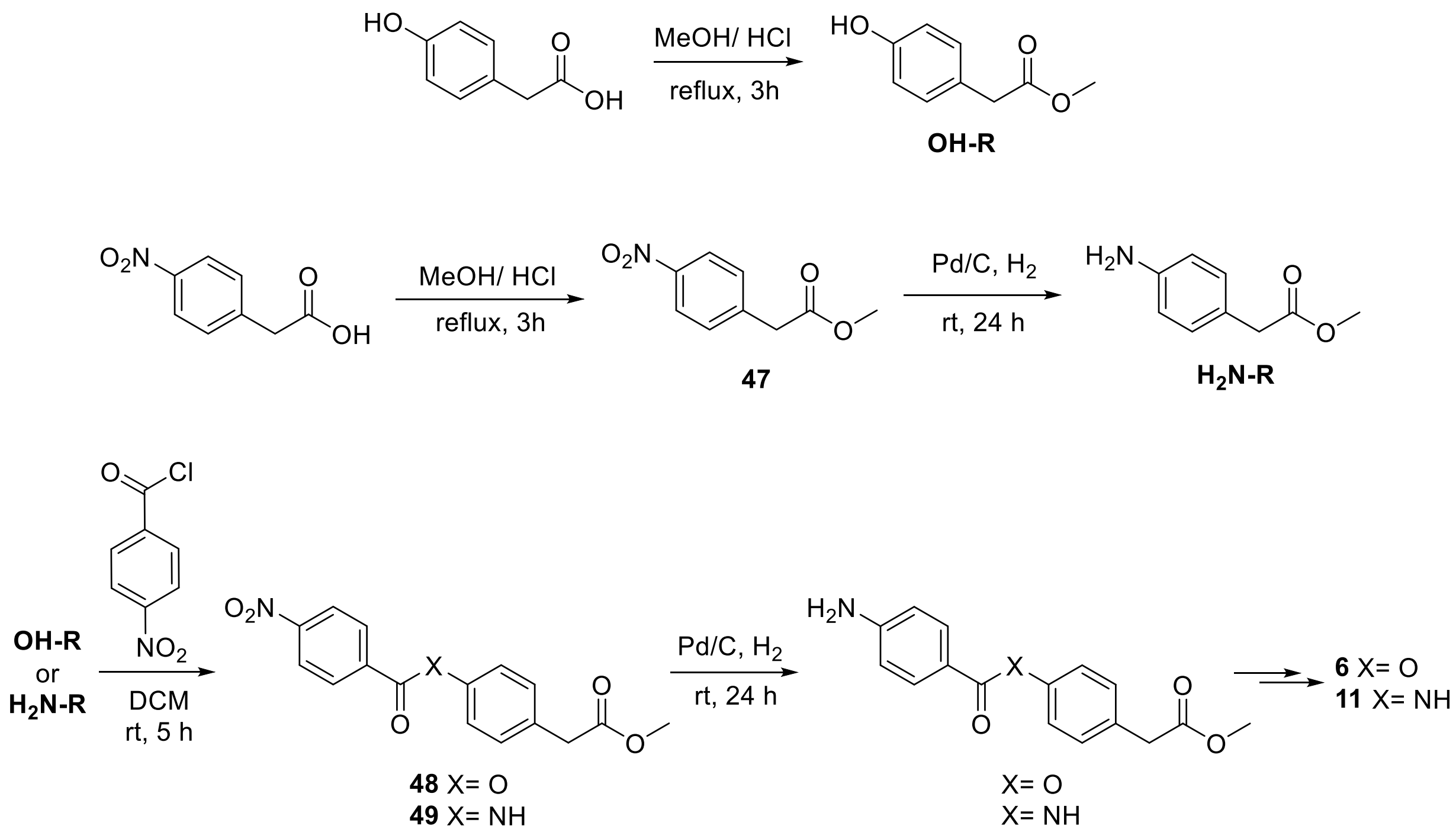

Scheme S3. Preparation of compound **8**

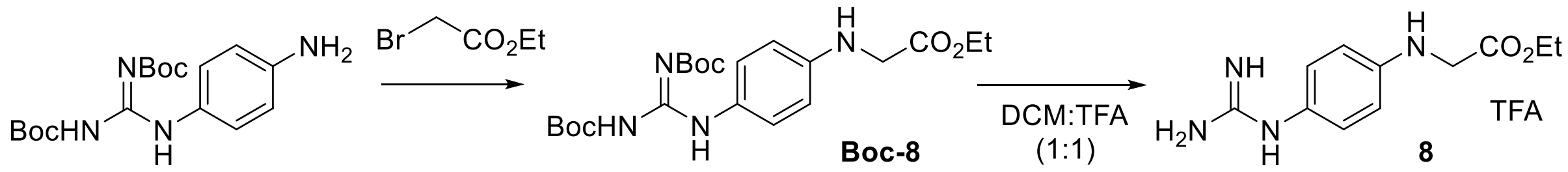

Scheme S4. Preparation of 2-azidoethanol **54** and aniline **55**

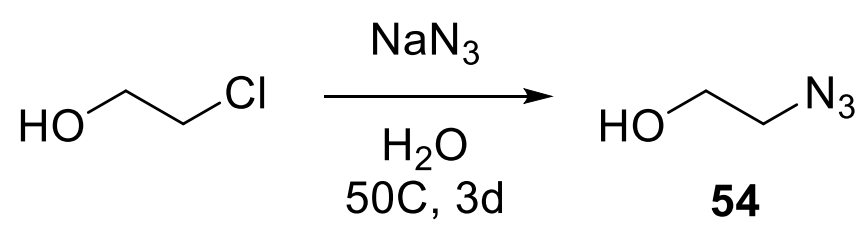

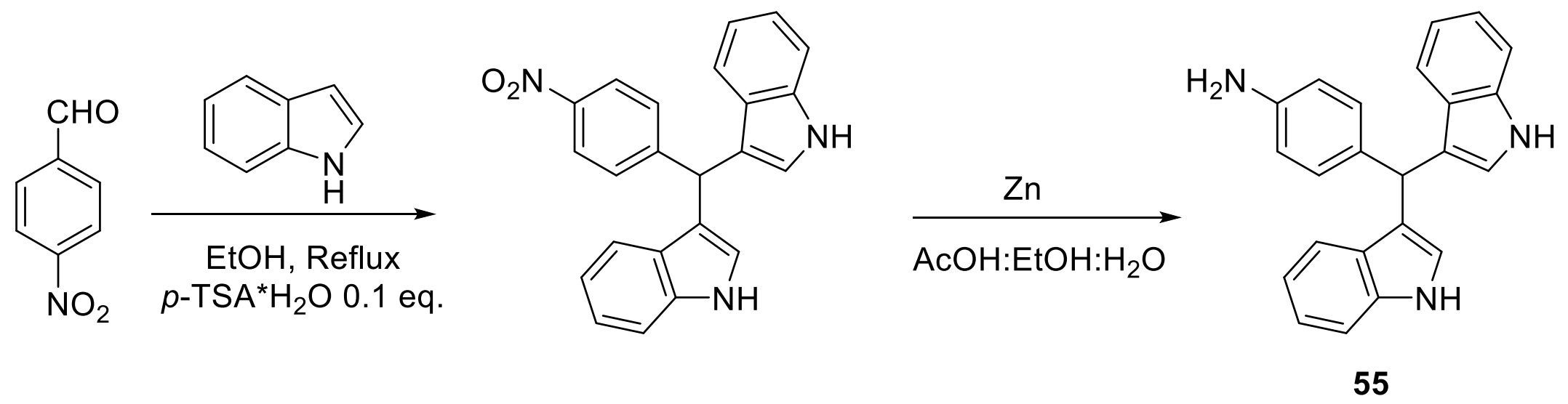

Scheme S5. Preparation of compounds **34** and **35**.

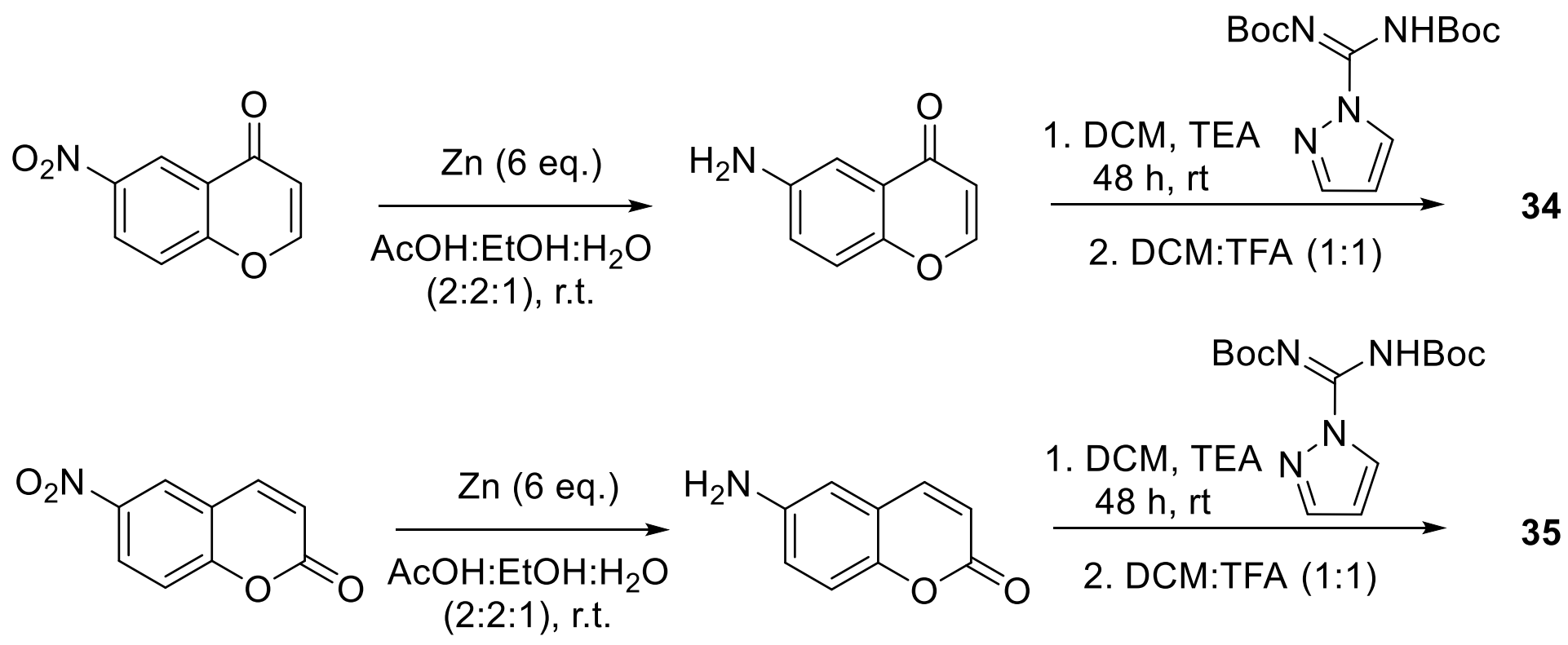

Scheme S6. Preparation of phenylguanidine derivatives of benzothiazole compounds **40** to **41**.

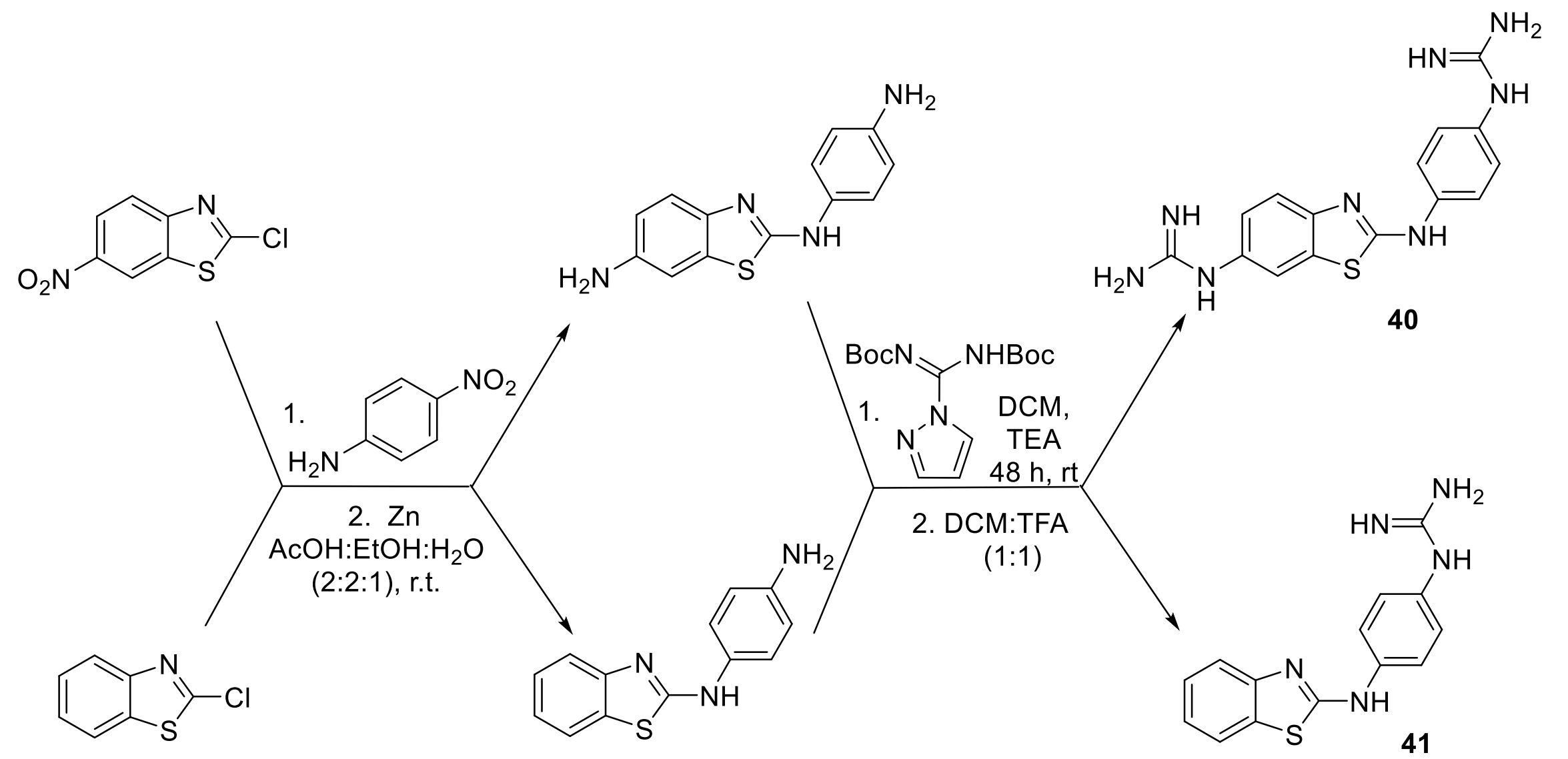
O2N
N
S
Cl
1.
NO2
H2N
2. Zn
AcOH:EtOH:H2O
(2:2:1), r.t.
NH2
H2N
NH
BocN
NHBoc
1.
DCM,
TEA
48 h, rt
2. DCM:TFA
(1:1)
NH2
HN
NH
H2N
NH
40
41

Method A.1 – Conjugated Amide Preparation

The corresponding carboxylic acids (1 eq.) were dissolved in 50 mL of dry DCM, [0.2 M], DMAP (1.5 eq.), EDC*HCl (1.5 eq.) and the corresponding amine (1.2 eq.) was then added in one portion. The solution was stirred at room temperature for 16 hours. The mixture was washed with 1 M HCl (2 x 50 mL), sat. $NaHCO_3$ (1 x 50 mL) and brine (1 x 50 mL). The organic phase was then dried over Na_2SO_4 and concentrated under vacuo. Products were typically isolated in almost quantitative yields and sufficiently pure to be directly used in the following step.

Method A.2 – Boc-Protected Conjugated Amide Preparation

The corresponding carboxylic acids (1 eq.) were dissolved in 50 mL of dry DCM, [0.2 M], DMAP (1.5 eq.), EDC*HCl (1.5 eq.) and *tert*-butyl (4-aminophenyl)carbamate (1.2 eq.) were added in one portion. The solution is stirred at room temperature for 16 hours. The mixture was washed with 1M HCl (2 x 50 mL), sat. $NaHCO_3$ (1 x 50 mL) and brine (1 x 50 mL). The organic phase was dried over Na_2SO_4 and concentrated under vacuo. Products were typically isolated in almost quantitative yields and sufficiently pure to be directly used in the following step.

Method A.3 – Weinreb Amide preparation

Benzoic and phenylacetic acid (1 eq.) were reacted with DMAP (1.5 eq.), EDC*HCl (1.5 eq.) and *N*,*O*-dimethyl hydroxylamine hydrochloride (1.5 eq.) in 50 mL of DCM. Each solution was stirred at room temperature for 16 hours. The mixture was washed with 1 M HCl (2 x 50 mL), sat. $NaHCO_3$ (1 x 50 mL) and brine (1 x 50 mL). The organic phase was dried over Na_2SO_4 and concentrated under vacuo, affording the corresponding Weinreb amide. Isolation occurred in almost quantitative yields and sufficiently purity, for the next step.

Method B.1 – Esterification or Amidation *via p*-Nitrobenzoyl chloride intermediate

p-Nitrobenzoic acid (1 eq.) was dissolved in dry DCM, [0.1]M, and $SOCl_2$ (1.2 eq.) was slowly added. DMF was added as catalyst (1-2 drops). The solution is stirred at room temperature overnight.

Volatiles were removed at low pressure. Crude *p*-nitrobenzoyl chloride was dissolved in dry DCM and either TEA or K_2CO_3 (2.5 eq.) were used as base. Addition of propargyl alcohol or phenol afforded the corresponding esters, while *N*,*O*-dimethyl hydroxylamine hydrochloride and aniline led to the formation of the corresponding amides. Upon completion, the reaction was washed with water twice, sat. $NaHCO_3$ and once with brine. The organic phase was dried over Na_2SO_4 and concentrated under vacuo. Products were isolated in almost quantitative yields and sufficiently pure to be directly used in the following step.

Method B.2 – Esterification or Amidation of (*Z*)-5-(2,3-Bis(*tert*-butoxycarbonyl)guanidino)-1H-indole-2-carboxylic acid

(*Z*)-5-(2,3-Bis(*tert*-butoxycarbonyl)guanidino)-1H-indole-2-carboxylic acid (1 eq.) was dissolved in 10 mL of dry DCM, [0.1] M. Next, DMAP (1.5 eq.), EDC*HCl (1.5 eq.) and 1.2 equivalents of the desired alcohol (2-azidoethanol or benzylic alcohol) or amine (p-methoxybenzyl amine or compound **54**) were added too. The solution was stirred at room temperature for 16 hours. The mixture was washed with 1M HCl (2 x 50 mL), sat. $NaHCO_3$ (1 x 50 mL) and brine (1 x 50 mL). The organic phase was dried over Na_2SO_4 and concentrated under vacuo. Products were typically isolated in almost quantitative yields and sufficiently pure to be directly used in the following step.

Method C.1 – Nitro reduction with Zinc dust

Nitro derivatives (1 eq.) were reacted with zinc powder (6 eq.) in an 2:2:1 EtOH:AcOH:H_2O [0.1] M solution under an N_2 environment. The reaction was allowed to proceed for 1-2 hours at room temperature, monitored by TLC (Hex:EtOAc, 1:1). Zinc dust in excess was filtered via syringe using a PTFE syringe filter (0.45 µm) and the solvent mixture was removed at low pressure. Traces of water and acetic acid were removed *in vacuo*. Amines were directly used in the next step without further purification.

Method C.2 – Nitro reduction by hydrogenation

Nitro derivatives (1 eq.) were dissolved in MeOH, [0.05] M. 10% Pd/C was added under inert atmosphere. The reaction was stirred overnight at room temperature under H_2 atmosphere. The following day, the catalyst is filtered off over a celite pad. Methanol is removed at low pressure to afford the desired amines, which were directly used in the next step without further purification.

Method D – Acyl Benzothiazole Synthesis

Benzo[d]thiazol-6-amine (1 eq.) was dissolved in 10 mL of dry THF, [0.1] M. The solution was cooled at -80° C. 3.1 eq. of *t*-BuLi (1.7 M in pentane) was added dropwise. The reaction was stirred for 15 minutes. In the meantime, the desired Weinreb Amide (1.2 eq.) was dissolved in 2 mL of dry THF. The latter solution was dripped into the first one. The reaction was allowed to proceed for 0.5 hours at -80° C, monitored by TLC (Hex:EtOAc, 7:3). Upon completion, the reaction was quenched with 1 mL MeOH. The crude was concentrated in vacuo and subjected to flash chromatography (Eluent: Hexane with a gradient of EtOAc from 0% to 20%).

Method E.1 – *N,N'*-Bis-Boc Protected Pyrazole-1-carboxamidine Guanidylation

The corresponding amines obtained in quantitative yields were directly subjected to guanidylation by dissolving them (1 eq.) in DCM [0.05] M in the presence of excess of triethylamine (5 eq.) and the *N,N'*-bis-Boc protected pyrazole-1-carboxamidine (1.2 eq.), which was used as guanidylating agent, was then added. The reaction was allowed to proceed for 48 h at room temperature under N_2 atm. The crude product was concentrated in vacuo and purified *via* flash chromatography (Eluent: Hexane with a gradient of EtOAc from 0% to 50%).

Method E.2 – Bis(*tert*-butoxycarbonyl)thiopseudourea Guanidylation

The corresponding amines (1 eq.) were dissolved in DCM [0.05]M in the presence of excess of triethylamine (5 eq.) and bis(*tert*-butoxycarbonyl)thiopseudourea (1.2 eq.) and $HgCl_2$ (1.2 eq.) were then added. The reaction was allowed to proceed for 16 h at room temperature under N_2 atm.

Precipitates formed during the reaction were filtered by a PTFE syringe filter (0.45 µm) and the crude was concentrated in vacuo and purified *via* flash chromatography (Eluent: Hexane with a gradient of EtOAc from 0% to 50%).

Method E.3– Bis-(*tert*-butoxycarbonyl)imidazolidine-2-thione Aminoimidazolidylation

The corresponding amines (1 eq.) were dissolved in DCM [0.05]M in the presence of excess of triethylamine (5 eq.) and bis(*tert*-butoxycarbonyl)imidazolidine-2-thione (1.2 eq.) and $HgCl_2$ (1.2 eq.) were then added. The reaction was allowed to proceed for 16 h at room temperature under N_2 atm. Precipitates formed during the reaction were filtered by a PTFE syringe filter (0.45 µm) and the crude was concentrated in vacuo and purified *via* flash chromatography (Eluent: Hexane with a gradient of EtOAc from 0% to 50%).

Method F.1 – Boc Deprotection

The corresponding Boc-protected guanidines (1 eq.) were dissolved in a 8:2 CH_2Cl_2:TFA solution [0.05]M. The reaction was allowed to proceed for 1 h at room temperature under N_2 atm. The reaction mixture was concentrated in vacuo. The crude was then diluted with 50 mL of CH_2Cl_2 and washed with $NaHCO_3$ sat. (30 mL x 2) and brine (20 mL x 1). The organic phase is dried over Na_2SO_4 and evaporated at low pressure.

Method F.2 – *Boc* Deprotection to final compounds

Bis-Boc protected compounds (1 eq.) were dissolved in a 1:1 CH_2Cl_2:TFA solution [0.05] M. The reaction was allowed to proceed for 4 h at room temperature under N_2 atm. The crude product was concentrated in vacuo. The excess of TFA was removed with Et_2O. Deprotected salts were obtained in quantitative yields.

Final compounds details

Esters and Amides

Amino((4-(methoxycarbonyl)phenyl)amino)methaniminium trifluoroacetate, 1 (scheme 1)

Compound **1** was deprotected according to ***Method F.2***. (quant. yield)

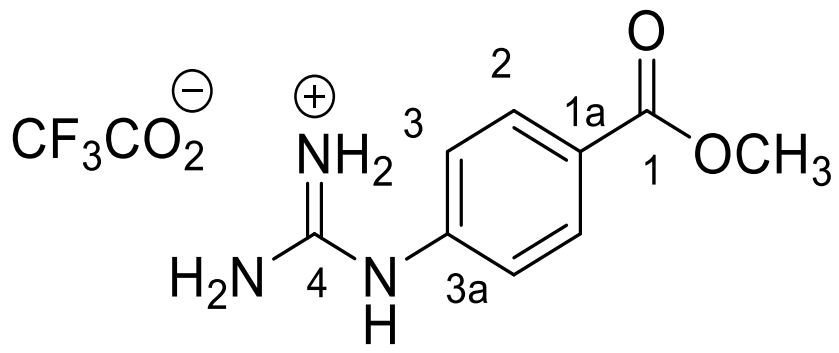

^{1}H NMR (400 MHz, CD_3OD) δ 8.12 (d, 2H, H_3, J_{2-3} = 8.8 Hz), 7.40 (d, 2H, H_2, J_{2-3} = 8.8 Hz), 3.93 (s, 3H, OC$\underline{H}_3$).

^{13}C NMR (150 MHz, CD_3OD) δ 167.6 (C_1), 157.7 (C_4), 141.1 (C_{3a}), 132.3 (C_3), 129.6 (C_{1a}), 125.0 (C_2), 52.8 (O$\underline{C}H_3$).

HRMS (m/z ESI$^+$): m/z found 194.0931 $[M+1]^+$; $C_9H_{12}N_3O_2$ required 194.0925

Amino((4-((octyloxy)carbonyl)phenyl)amino)methaniminium trifluoroacetate, 2 (scheme 1)

Compound **2** was deprotected according to ***Method F.2***. (quant. yield)

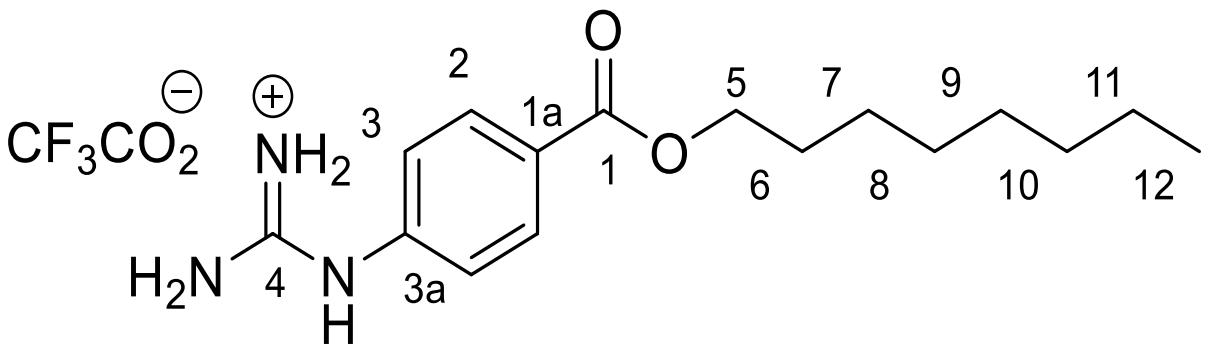

^{1}H NMR (400 MHz, CD_3OD) 8.11 (d, 2H, H_3, $J_{2\text{-}3}$ = 8.2 Hz), 7.40 (d, 2H, H_2, $J_{2\text{-}3}$ = 8.2 Hz), 4.35 (t, 2H, H_5, $J_{5\text{-}6}$ = 6.5 Hz), 1.80 (q, 2H, H_6, $J_{5\text{-}6}$ = $J_{6\text{-}7}$ = 7.8 Hz), 1.52 – 1.16 (m, 10H, H_7, H_8, H_9, H_{10}, H_{11}), 0.92 (t, 3H, H_{12}, $J_{11\text{-}12}$ = 6.9 Hz).

^{13}C NMR (150 MHz, CD_3OD) δ 165.8(C_1), 156.3 (C_4), 139.7 (C_{3a}), 130.8 (C_3), 128.3 (C_{1a}), 123.5 (C_2), 65.0 (C_5), 31.5 (C_6), 28.9 (C_7, C_8), 28.4 (C_9), 25.7 (C_{10}), 22.3 (C_{11}), 13.0 (C_{12}).

HRMS (m/z ESI$^+$): m/z found 292.2028 $[M+1]^+$; $C_{16}H_{426}N_3O_2$ required 292.2020

Amino((4-((prop-2-yn-1-yloxy)carbonyl)phenyl)amino)methaniminium trifluoroacetate, 3 (scheme 1)

Compound **3** was deprotected according to ***Method F.2***. (quant. yield)

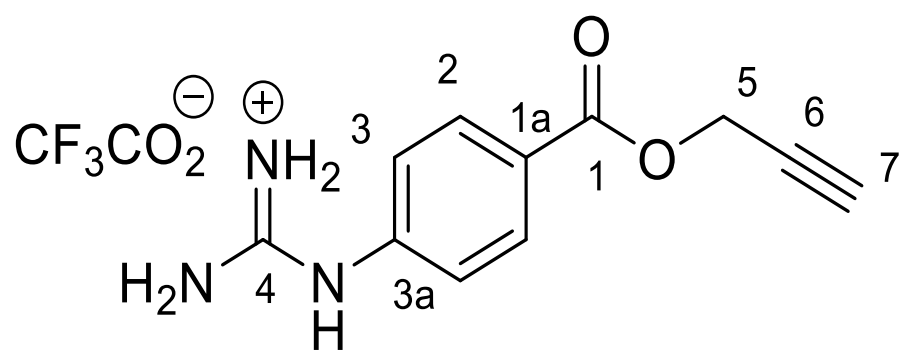

^{1}H NMR (400 MHz, CD_3OD) δ 8.14 (d, 2H, H_3, $J_{2\text{-}3}$ = 8.8 Hz), 7.41 (d, 2H, H_2, $J_{2\text{-}3}$ = 8.8 Hz), 4.97 (d, 2H, H_5, $J_{5\text{-}7}$ = 2.4 Hz), 3.01 (t, 1H, H_7, $J_{5\text{-}7}$ = 2.4 Hz).

^{13}C NMR (150 MHz, CD_3OD) δ 166.2 (C_1), 157.7 (C_4), 141.5 (C_{1a}), 132.5 (C_3), 129.0 (C_{3a}), 124.9 (C_2), 78.6 (C_6), 76.5 (C_7), 53.4 (C_5).

HRMS (m/z ESI$^+$): m/z found 218.0920 $[M+1]^+$; $C_{11}H_{12}N_3O_2$ required 218.0925

Phenyl 4-guanidinobenzoate, 4 (scheme 1)

Following method E.2, this compound was produced as a brown solid (34 mg, 91% yield)

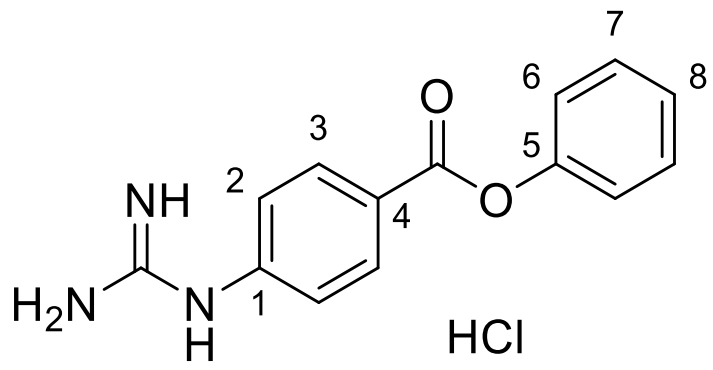

^{1}H NMR (400 MHz, CD_3OD) δ (400 MHz, *d_6*-DMSO); 10.53 (s, 1H, NH), 8.17 (d, J = 8.7 Hz, 2H, NH, H-3 and H-3'), 7.87 (s, 1H, NH), 7.49 (dd, J = 8.3, 7.6 Hz, 2H, H-7 and H-7'), 7.44 (d, J = 8.7 Hz, 2H, H-2 and H-2'), 7.36 – 7.31 (m, 1H, H-8), 7.30 – 7.27 (m, 2H, H-6 and H-6').

^{13}C NMR (150 MHz, CD_3OD) δ (151 MHz, *d_6*-DMSO); 163.95 (s, CO), 155.53 (s, CN), 150.53 (s, C-1), 141.22 (s, C-5), 133.07 (s, C-4), 131.42 (s, C-3), 129.58 (s, C-7), 122.67 (s, C-2), 122.19 (s, C-8), 121.86 (s, C-6).

HRMS (m/z ESI+): Found ($[M+H]^+$ $C_{14}H_{13}N_3O_2$ Requires 255.1008)

4-(2-Methoxy-2-oxoethyl)phenyl 4-guanidinobenzoate, 5 (scheme 1)

Following method **E.2**, this compound was obtained as a yellow solid (40 mg, 94% yield)

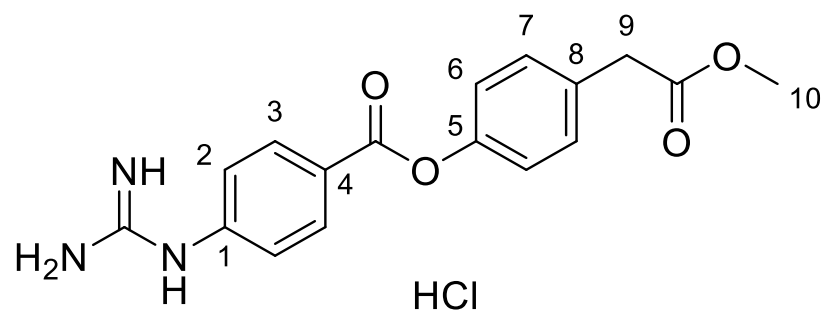

^{1}H NMR (400 MHz, d_6-DMSO) δ_H 12.52 (s, 1H), 10.63 (s, 1H), 8.13 (d, J = 8.1 Hz, 2H), 7.41 (d, J = 8.1 Hz, 2H), 7.33 (d, J = 8.1 Hz, 2H), 7.19 (d, J = 8.1 Hz, 2H), 3.60 (s, 2H), 3.04 (s, 3H).

^{13}C NMR (150 MHz, d_6-DMSO) δ 173.8 (CO), 165.2 (CO), 157.1 (C=N), 148.2 (Ar), 143.7 (Ar), 131.5 (Ar), 131.1 (Ar), 130.1 (Ar), 122.0 (Ar), 116.2 (Ar), 116.3 (Ar), 51.9, 40.4.

HRMS (m/z ESI+): Found ([M+H] $^+$ $C_{17}H_{19}N_7O$ Requires 328.1219)

4-(Imidazolidin-2-ylideneamino)phenyl 4-guanidinobenzoate, 6 (scheme 2)

Following method **E.3**, this compound was produced as a brown solid (85 mg, 95% yield)

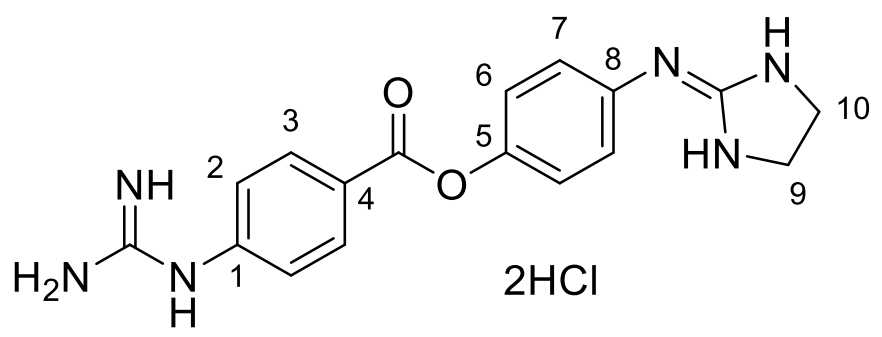

^{1}H NMR (400 MHz, CD_3OD) δ_H (400 MHz, d_6-DMSO); 10.75 (s, 1H, NH), 10.54 (s, 1H, NH), 8.40 (s, 2H, NH), 8.16 (d, J = 8.7 Hz, 2H, H-3 and H-3'), 7.87 (s, 2H, NH), 7.45 (d, J = 8.7 Hz, 4H, H-2 and H-2'), 7.37 (s, 6H, H-6, H-6', H-7 and H-7'), 3.68 (s, H-9 and H-10)

^{13}C NMR (150 MHz, CD_3OD δ_C (151 MHz, d_6-DMSO); 163.91 (s, CO), 157.89 (s, CN), 155.57 (s, CN), 148.41 (s, C-8), 141.20 (s, C-1), 131.47 (s, C-3), 133.65 (s, C-5), 125.09 (s, C-4), 124.62 (s, C-6), 123.06 (s, C-7), 122.59 (s, C-2), 42.66 (s, C9 and C-10).

HRMS (m/z ESI+): Found ($[M+H]^+$ $C_{17}H_{18}N_6O_2$ Requires 338.1491)

Amino((4-((2-ethoxy-2-oxoethyl)amino)phenyl)amino)methaniminium trifluoroacetate, 7 (scheme S3)

Compound **7** was deprotected according to ***Method F.2***. (quant. yield)

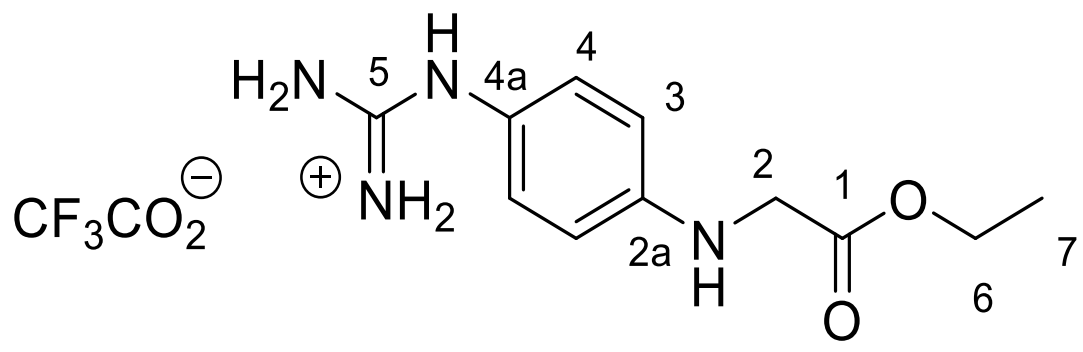

^{1}H NMR (400 MHz, CD_3OD) δ 7.04 (d, 2H, H_3, $J_{3\text{-}4}$ = 8.9 Hz), 6.67 (d, 1H, H_4, $J_{3\text{-}4}$ = 8.9 Hz), 4.19 (q, 2H, H_6, $J_{6\text{-}7}$ = 7.3 Hz),), 3.94 (a, 2H, H_2),), 1.26 (t, 3H, H_7, $J_{6\text{-}7}$ = 7.3 Hz).

^{13}C NMR (150 MHz, CD_3OD) δ 171.7 (C_1), 157.4 (C_5), 148.1 (C_{4a}), 127.4 (C_4), 123.4 (C_{2a}), 113.1 (C_3), 60.8 (C_6), 44.7 (C_2), 13.1 (C_7).

HRMS (m/z ESI$^+$): m/z found 237.1351 $[M+1]^+$; $C_{11}H_{17}N_4O_2$ required 237.1347

4-Guanidino-N-phenylbenzamide, 8 (scheme 1)

Following method E.2, this compound was obtained as a light brown solid (50 mg, 81% yield)

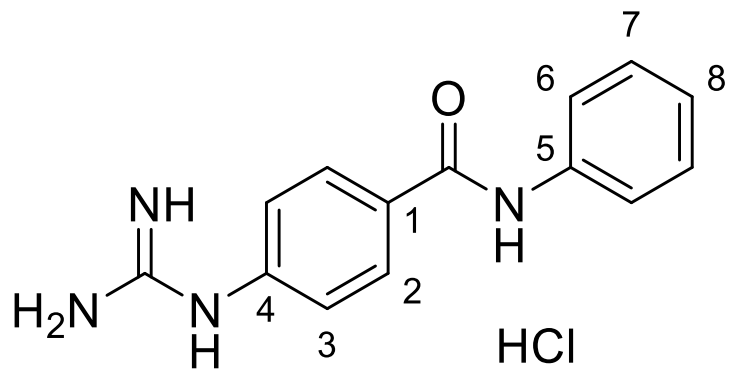

^{1}H NMR (400 MHz, *d6*-DMSO) δ 10.27 (s, 1H, NH), 10.09 (s, 1H, NH), 8.06 (d, J = 8.6 Hz, 2H, H-3 and H-3'), 7.78 (d, J = 7.7 Hz, 2H, H-6 and H-6'), 7.64 (s, 1H, NH), 7.40 – 7.33 (m, 4H, H-2, H-2', H-7 and H-7'), 7.12 (t, J = 7.4 Hz, 1H, H-8).

^{13}C NMR (150 MHz, *d6*-DMSO) δ 164.58 (s, CO), 155.65 (s, CN), 139.06 (s, C-4), 138.72 (s, C-1), 131.92 (s, C-5), 129.27 (s, C-3), 128.67 (s, C-7), 123.85 (s, C-8), 123.17 (s, C-2), 120.55 (s, C-6).

HRMS (m/z ESI+): Found 255.1238 ([M+H] $^+$ $C_{14}H_{14}N_4$ Requires 254.1168)

N-(4-aminophenyl)-4-guanidinobenzamide, 9 (scheme 2)

Following the method described in *J. Med. Chem.*, 4397-4406, *55* (**2012**) a white solid was obtained (95%).

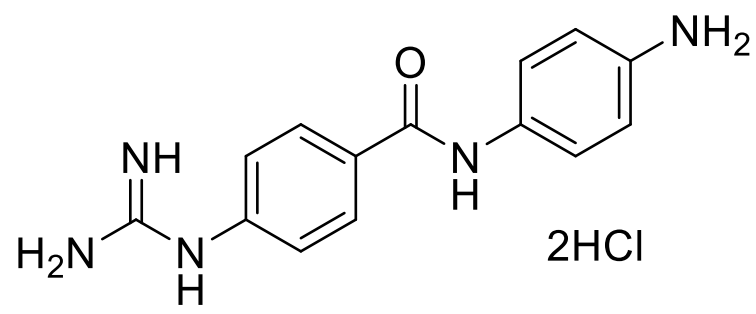

^{1}H NMR (400 MHz, D2O) δ 8.10 (d, 2H, J = 7.5 Hz, Ar.), 8.17 (d, 2H, J = 7.5 Hz, Ar.), 8.23 (d, 2H, J = 7.5 Hz, Ar.), 8.42 (d, 2H, J = 7.5 Hz, Ar.).

^{13}C NMR (150 MHz, D2O) δ 109.9, 117.4, 122.1, 123.3, 126.3, 128.9, 130.9, 135.8 (Ar.), 155.7 (CN), 168.0 (CO).

Methyl 2-(4-(4-guanidinobenzamido)phenyl)acetate, 10 (scheme 1)

Following method **E.2**, this compound was obtained as a yellow powder (60 mg, %yield)

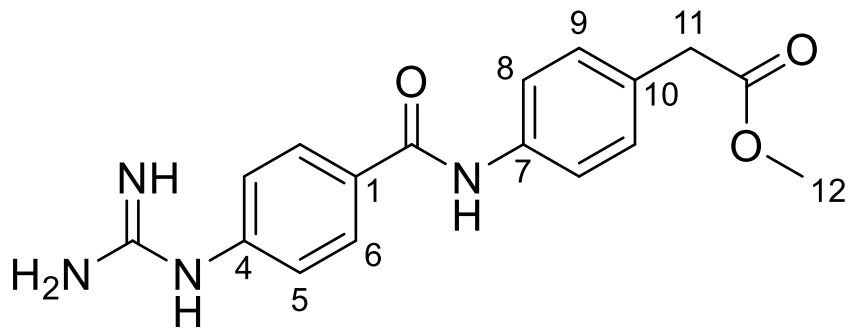

^{1}H NMR (400 MHz, *d6*-DMSO) δ: 10.04 (bs, H, CONH), 8.93 (bs, H, NH), 7.84 (bs, H, NH), 6.63 (bs, 2H, NH), 7.54 (m, 4H, H_6, H_9), 7.53 (d, 2H, H_8, J_{8-9} = 8.8 Hz), 6.97 (d, 2H, H_5, J_{5-6} = 8.7 Hz), 3.59 (s, 2H, C$\underline{H}_2$CO)., 3.66 (s, 3H, OC$\underline{H}_3$).

^{13}C NMR (150 MHz, *d6*-DMSO) δ 173.8 (CO), 164.6 (CONH), 157.2 (C=N), 141.9 (Ar), 136.6 (Ar), 130.4 (Ar), 130.2 (Ar), 129.9 (Ar), 124.2 (Ar), 122.1 (Ar), 116.4 (Ar), 51.9, 40.4.

HRMS (m/z ESI+): Found ($[M+H]^+$ $C_{17}H_{18}N_4O_3$ Requires 327.1379)

4-Guanidino-N-(4-guanidinophenyl)benzamide, 11 (scheme 2)

Following the method described in *J. Med. Chem.* 2008, *51,* 909–923**,** a white solid was obtained (98%)

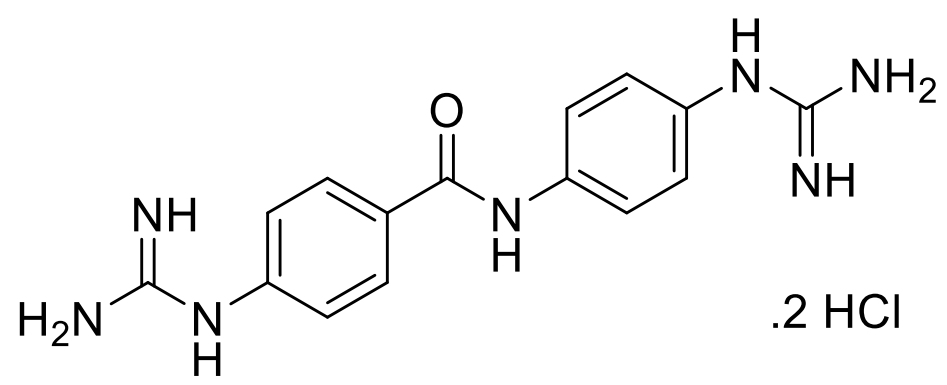

^{1}H NMR (400 MHz, D2O) δ: 7.32 (d, 2H, *J* = 8.0 Hz, arom.), 7.40 (d, 2H, *J* = 8.0 Hz, arom.), 7.59 (d, 2H, *J* = 8.0 Hz, arom.), 7.89 (d, 2H, *J* = 8.0 Hz, arom.).

13C NMR (D2O), δ: 123.0, 124.1, 126.1, 128.7, 130.7, 131.6, 135.9, 137.8 (arom.), 155.3, 155.8 (CN), 167.9 (CO).

N-(4-((4,5-dihydro-1H-imidazol-2-yl)amino)phenyl)-4-guanidinobenzamide, 12 (scheme 2)

Following method **E.3**, this compound was obtained as a white powder (50 mg, 88% yield)

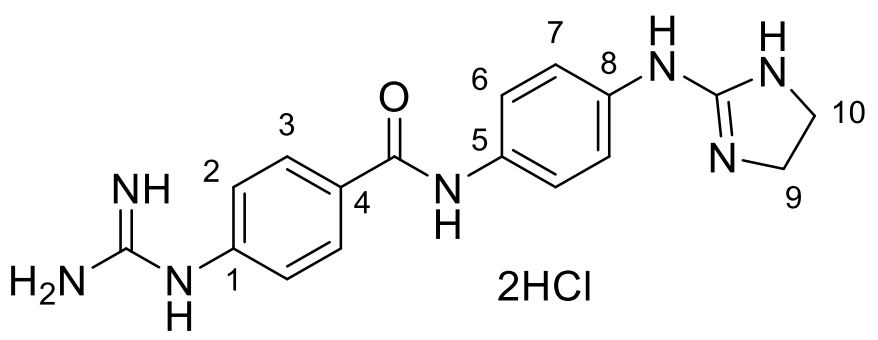

1H NMR, δ_H (400 MHz, *d2O*); 7.77 (d, J = 8.5 Hz, 2H, H-2 and H-2'), 7.45 (d, J = 8.7 Hz, 2H, H-7 and H-7'), 7.29 (d, J = 8.5 Hz, 2H, H-3 and H-3'), 7.18 (d, J = 8.7 Hz, 2H, H-6 and H-6'), 3.62 (s, 4H, H-9 and H-10).

13C NMR, δ_C (151 MHz, *d2O*); 168.45 (s, CO), 158.61 (s, CN), 155.74 (s, CN), 138.21 (s, C-1), 135.86 (s, C-8), 132.13 (s, C-4), 132.06 (s, C-5), 129.17 (s, C-2), 124.88 (s, C-7), 124.52 (s, C-3), 123.39 (s, C-6), 42.66 (s, C-9 and C-10).

HRMS (m/z ESI+): Found ([M+H] $^+$ $C_{17}H_{19}N_7O$ Requires 337.1651)

Amino((4-(methoxy(methyl)carbamoyl)phenyl)amino)methaniminium trifluoroacetate, 13 (scheme 1)

Compound **13** was deprotected according to ***Method F.2***. (quant. yield)

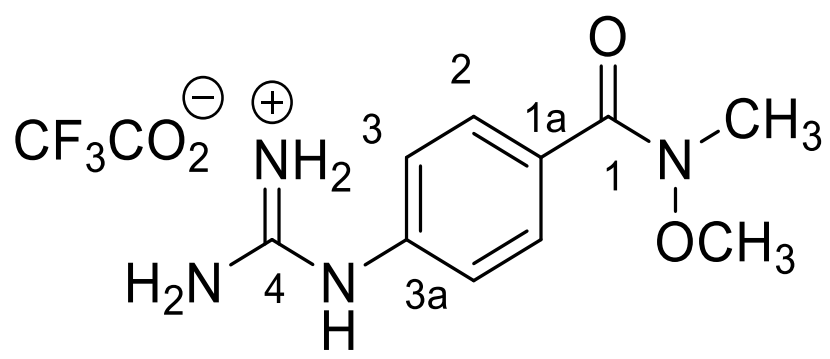

^{1}H NMR (400 MHz, CD_3OD) δ 7.76 (d, 2H, H_3, $J_{2\text{-}3}$ = 8.9 Hz), 7.38 (d, 2H, H_2, $J_{2\text{-}3}$ = 8.9 Hz), 3.63 (s, 3H, $OC\underline{H}_3$), 3.39 (s, 3H, $NC\underline{H}_3$).

^{13}C NMR (150 MHz, CD_3OD) δ 170.6 (C_1), 157.9 (C_4), 138.8 (C_{3a}), 133.7 (C_{1a}), 131.0 (C_2), 125.3 (C_3), 61.7 ($O\underline{C}H_3$), 37.7 ($N\underline{C}H_3$).

HRMS (m/z ESI^{+}): m/z found 223.1197 $[M+1]^{+}$; $C_{10}H_{15}N_4O_2$ required 223.1190

Indole derivatives

Amino((2-carboxy-1*H*-indol-5-yl)amino)methaniminium trifluoroacetate, 14 (scheme 3)

Compound **14** was deprotected according to ***Method F.2***. (quant. yield)

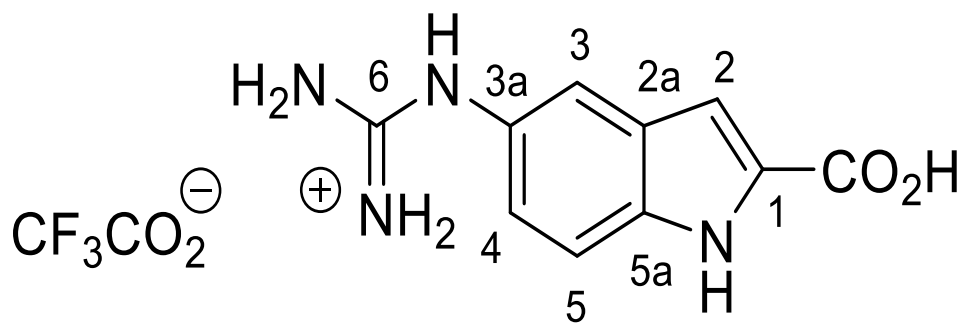

^{1}H NMR (400 MHz, CD_3OD) δ 7.64 (s, 1H, H_3), 7.62 (d, 1H, H_5, $J_{4\text{-}5}$ = 8.8 Hz), 7.34 (s, 1H, H_2), 7.25 (dd, 1H, H_4, $J_{4\text{-}5}$ = 8.8 Hz, $J_{3\text{-}4}$ = 2.0 Hz).

^{13}C NMR (100 MHz, CD_3OD) δ 163.3 (C_1), 157.3 (C_6), 138.9 (C_{1a}), 127.0 (C_{3a}), 126.3 (C_{2a}), 122.9 (C_4), 120.1 (C_3), 111.7 (C_5), 109.9 (C_2).

HRMS (m/z ESI$^+$): m/z found 219.0882 $[M+1]^+$; $C_{10}H_{11}N_4O_2$ required 219.0877

Amino((2-(ethoxycarbonyl)-1*H*-indol-5-yl)amino)methaniminium trifluoroacetate, 15 (scheme 3)

Compound **15** was deprotected according to ***Method F.2***. (quant. yield)

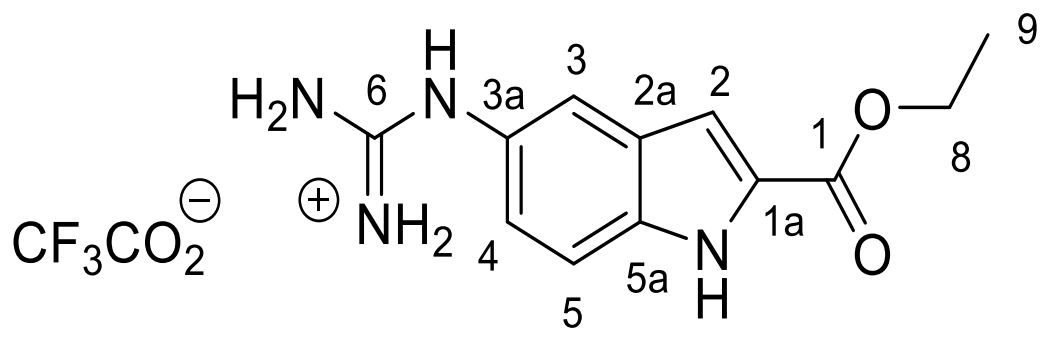

^{1}H NMR (400 MHz, CD_3OD) δ 7.63 (d, 1H, H_3, $J_{3\text{-}4}$ = 1.6 Hz), 7.58 (d, 1H, H_5, $J_{4\text{-}5}$ = 8.7 Hz), 7.24 (s, 1H, H_2), 7.19 (d, 1H, H_4, $J_{4\text{-}5}$ = 8.7 Hz, $J_{3\text{-}4}$ = 1.6 Hz), 4.42 (q, 2H, H_8, $J_{8\text{-}9}$ = 7.2 Hz), 1.43 (t, 3H, H_9, $J_{8\text{-}9}$ = 7.2 Hz).

^{13}C NMR (150 MHz, CD_3OD) δ 163.1 (C_1), 158.8 (C_6), 138.1 (C_{2a}), 130.8 (C_{1a}), 129.1 (C_{5a}), 128.2 (C_{3a}), 124.4 (C_4), 121.4(C_3), 114.8 (C_5), 109.2 (C_2), 62.1 (C_8), 14.7 (C_9).

HRMS (m/z ESI$^+$): m/z found 247.1196 $[M+1]^+$; $C_{12}H_{15}N_4O_2$ required 247.1190

Amino((2-(ethoxycarbonyl)-1-methyl-1*H*-indol-5-yl)amino)methaniminium trifluoroacetate, 16 (scheme 3)

Compound **16** was deprotected according to ***Method F.2***. (quant. yield)

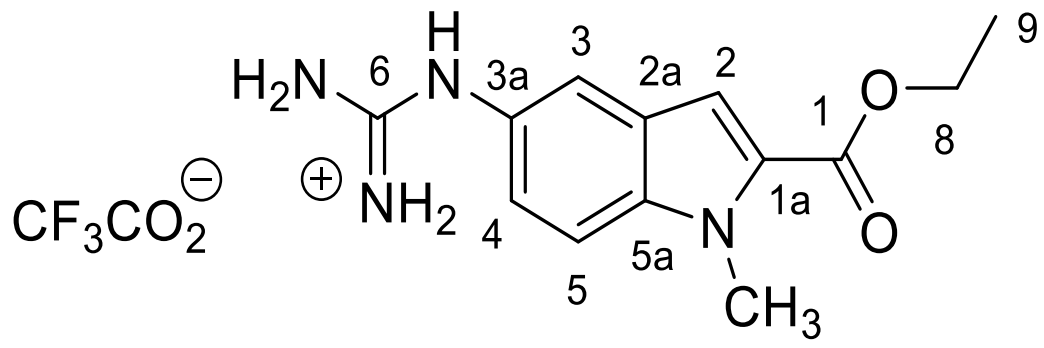

^{1}H NMR (400 MHz, CD_3OD) δ 7.61 (d, 1H, H_3, $J_{3\text{-}4}$ = 1.7 Hz), 7.56 (d, 1H, H_5, $J_{4\text{-}5}$ = 8.7 Hz), 7.21 (s, 1H, H_2), 7.17 (dd, 1H, H_4, $J_{3\text{-}4}$ = 1.7 Hz, $J_{4\text{-}5}$ = 8.7 Hz,), 4.40 (q, 2H, H_8, $J_{8\text{-}9}$ = 7.3 Hz), 2.54 (s, 3H, N$\underline{C}H_3$), 1.41 (t, 3H, H_9, $J_{8\text{-}9}$ = 7.3 Hz).

^{13}C NMR (150 MHz, CD_3OD) δ 161.6 (C_1), 157.3 (C_6), 136.7 (C_{3a}), 129.4 (C_{2a}), 127.7 (C_{5a}), 126.8 (C_{1a}), 123.0 (C_4), 120.0 (C_3), 113.4 (C_5), 107.8 (C_2), 60.6 (C_8), 23.9 (N$\underline{C}H_3$), 13.3 (C_9).

HRMS (m/z ESI$^+$): m/z found 261.1359 $[M+1]^+$; $C_{13}H_{17}N_4O_2$ required 261.1347

Amino((2-((2-azidoethoxy)carbonyl)- 1*H*-indol-5-yl)amino)methaniminium trifluoroacetate, 17 (scheme 3)

Compound **17** was deprotected according to ***Method F.2***. (quant. yield)

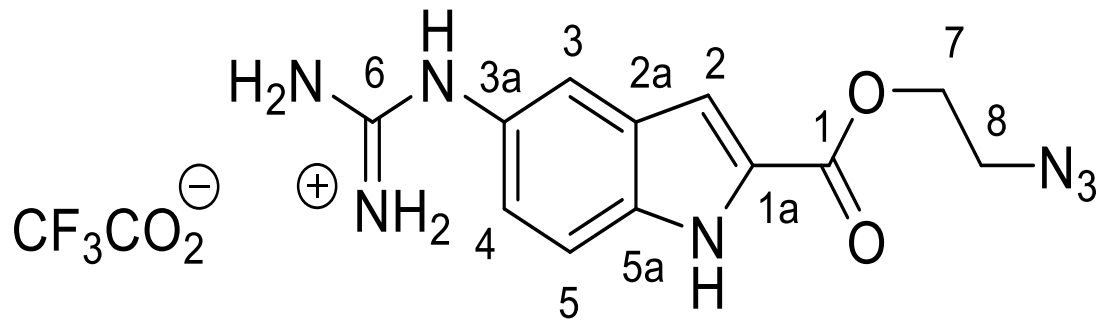

^{1}H NMR (400 MHz, CD_3OD) δ 8.82 (s, 1H, H_2), 7.74 (d, 1H, H_5, $J_{4\text{-}5}$ = 9.0 Hz), 7.22 (d, 1H, H_3, $J_{3\text{-}4}$ = 2.2 Hz), 6.93 (dd, 1H, H_4, $J_{3\text{-}4}$ = 2.2 Hz, $J_{4\text{-}5}$ = 9.0 Hz), 3.56 (t, 2H, H_7, $J_{7\text{-}8}$ = 5.5 Hz), 2.71 (t, 2H, H_8, $J_{7\text{-}8}$ = 5.5 Hz).

^{13}C NMR (100 MHz, CD_3OD) δ 161.9 (C_1), 158.0 (C_6), 150.3 (C_2), 146.8 (C_{2a}), 145.3 (C_{3a}), 135.1 (C_{5a}), 133.2 (C_{1a}), 122.5 (C_5), 115.8 (C_4), 104.9 (C_3), 62.9 (C_7), 43.1 (C_8).

HRMS (m/z ESI$^+$): m/z found 288.1210 $[M+1]^+$; $C_{12}H_{14}N_7O_2$ required 288.1204

Amino((2-((benzyloxy)carbonyl)- 1*H*-indol-5-yl)amino)methaniminium trifluoroacetate, 18 (scheme 3)

Compound **18** was deprotected according to ***Method F.2***. (quant. yield)

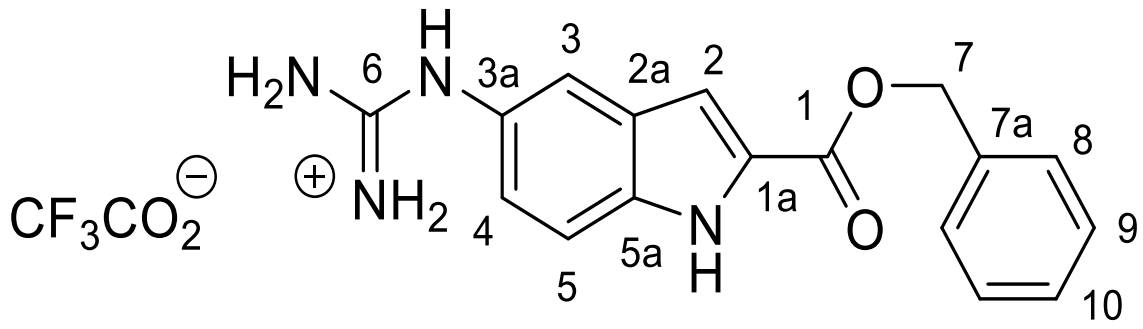

^{1}H NMR (400 MHz, CD_3OD) δ 7.63 (d, 1H, H_3, $J_{3\text{-}5}$ = 2.0 Hz), 7.58 (d, 1H, H_5, $J_{4\text{-}5}$ = 8.7 Hz), 7.50 (d, 2H, H_8, $J_{8\text{-}9}$ = 8.4 Hz), 7.45 – 7.33 (m, 3H, H_9, H_{10}), 7.28 (d, 1H, H_2, $J_{2\text{-}3}$ = 0.5 Hz), 7.19 (dd, 1H, H_4, $J_{3\text{-}4}$ = 8.4 Hz, $J_{3\text{-}4}$ = 2.0 Hz), 5.42 (s, 2H, H_7).

^{13}C NMR (150 MHz, CD_3OD) δ 162.8 (C_1), 158.8 (C_6), 138.2 (C_{2a}), 137.5 (C_{7a}), 130.5 (C_{5a}), 129.6 (C_9), 129.4 (C_8), 129.1 (C_{10}), 128.3 (C_{1a}), 124.5 (C_4), 121.4 (C_3), 114.9 (C_5), 109.6 (C_2), 67.6 (C_7).

HRMS (m/z ESI$^+$): m/z found 309.1339 $[M+1]^+$; $C_{17}H_{17}N_4O_2$ required 309.1347

Amino((2-((4-methoxybenzyl)carbamoyl)-1*H*-indol-5-yl)amino)methaniminium trifluoroacetate, 19 (scheme 3)

Compound **19** was deprotected according to ***Method F.2***. (quant. yield)

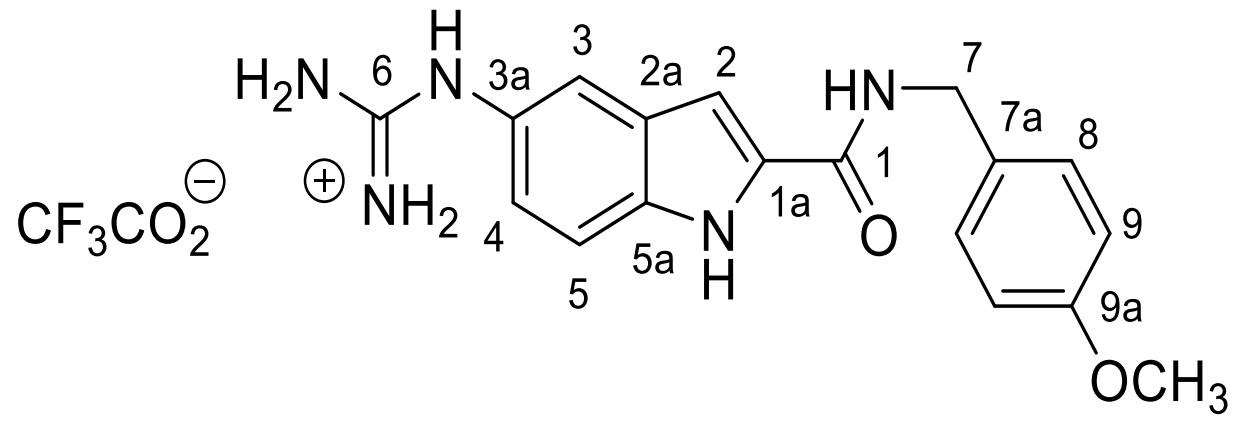

^{1}H NMR (400 MHz, CD_3OD) δ 7.60 – 7.52 (m, 2H, H_3, H_5), 7.30 (d, 2H, H_8, $J_{8\text{-}9}$ = 8.6 Hz), 7.14 (s, 1H, H_2), 7.13 (dd, 1H, H_4, $J_{3\text{-}4}$ = 2.1 Hz, $J_{2\text{-}3}$ = 8.7 Hz), 6.89 (d, 2H, H_9, $J_{8\text{-}9}$ = 8.6 Hz), 4.53 (s, 2H, H_7), 3.78 (s, 3H, $OC\underline{H}_3$).

^{13}C NMR (150 MHz, CD_3OD) δ 160.5 (C_1), 157.5 (C_{10}), 155.8 (C_6), 134.6 (C_{1a}), 131.3 (C_{2a}), 129.2 (C_{3a}), 127.0 (C_8), 126.6 (C_{7a}), 125.1 (C_{5a}), 120.8 (C_2), 118.1 (C_3), 112.0 (C_9), 111.7 (C_5), 101.4 (C_4), 52.8 (C_7), 40.7 ($O\underline{C}H_3$).

HRMS (m/z ESI$^+$): m/z found 338.1618 $[M+1]^+$; $C_{18}H_{20}N_5O_2$ required 338.1612

Amino((2-((4-(di(1*H*-indol-2-yl)methyl)phenyl)carbamoyl)- 1*H*-indol-5-yl)amino) methaniminium trifluoroacetate, 20 (scheme 3)

Compound **20** was deprotected according to ***Method F.2***. (quant. yield)

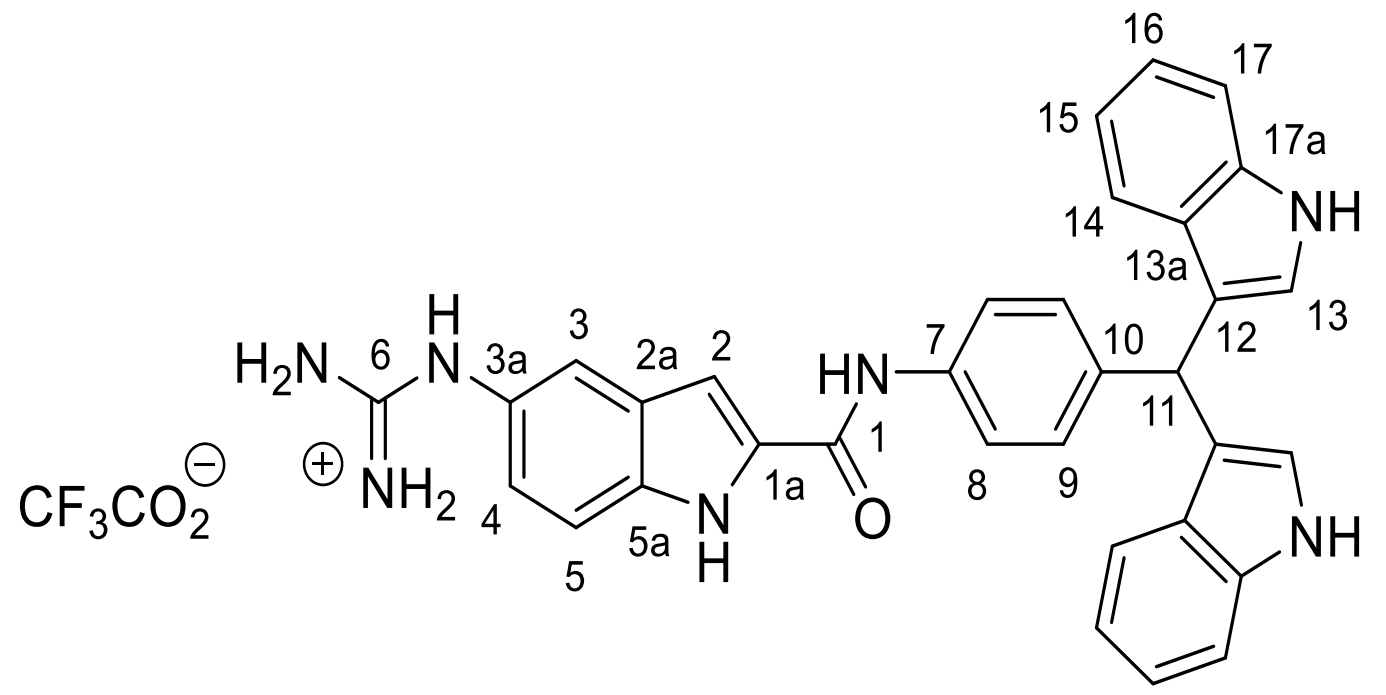

^{1}H NMR (400 MHz, CD_3OD) δ 8.71 (d, 1H, H_3, J_{3-4} = 2.2 Hz), 8.08 (dd, 1H, H_4, J_{4-5} = 9.1 Hz, J_{3-4} = 2.2 Hz), 7.68 (d, 2H, H_8, J_{8-9} = 8.3 Hz), 7.64 (s, 1H, H_2), 7.58 (d, 1H, H_5, J_{4-5} = 9.1 Hz), 7.36 – 7.30 (m, 4H, H_9, H_{17}), 7.28 (d, 2H, H_{14}, J_{14-15} = 7.9 Hz), 7.02 (t, 2H, H_{15}, J_{14-15} = J_{15-16} = 7.3 Hz), 6.85 (t, 2H, H_{16}, J_{15-16} = J_{16-17} = 7.3 Hz), 6.81 (s, 2H, H_{11}).

^{13}C NMR (100 MHz, CD_3OD) δ 162.3 (C_1), 158.7 (C_6), 141.3 (C_7), 140.6 (C_{10}), 139.5 (C_{2a}), 136.6 (C_{15a}), 136.3 (C_{3a}), 135.1 (C_{1a}), 128.5 (C_9), 126.6 (C_{17a}), 126.3 (C_{5a}), 123.5 (C_{13}), 120.8 (C_{16}), 120.1 (C_8), 119.3 (C_3), 119.1 (C_{14}), 118.7 (C_4), 118.1 (C_{12}, C_{15}), 112.9 (C_5), 111.4 (C_{17}), 105.9 (C_2), 35.7 (C_{11}).

HRMS (m/z ESI^+): m/z found 538.2355 $[M+1]^+$; $C_{33}H_{28}N_7O$ required 538.2350

Michael acceptors

(E)-amino((4-(but-2-enamido)phenyl)amino)methaniminium trifluoroacetate, 21 (scheme 4)

Compound **21** was deprotected according to ***Method F.2***. (quant. yield)

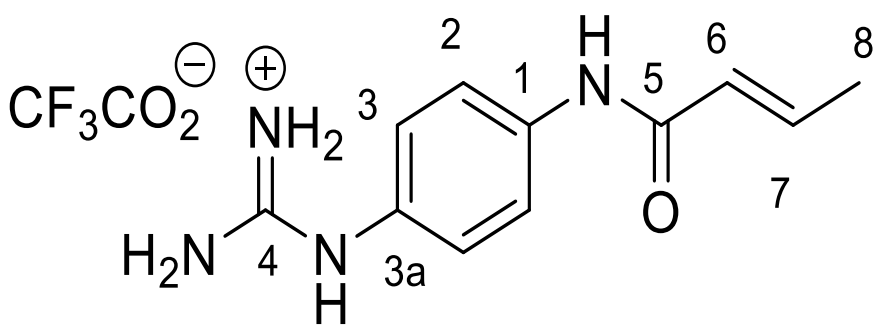

^{1}H NMR (400 MHz, CD_3OD) δ 7.74 (d, 2H, H_3, $J_{2\text{-}3}$ = 8.8 Hz), 7.26 (d, 2H, H_2, $J_{2\text{-}3}$ = 8.8 Hz), 6.98 (dq, 1H, H_7, $J_{6\text{-}7}$ = 15.6 Hz, $J_{7\text{-}8}$ = 7.0 Hz), 6.14 (dq, 1H, H_6, $J_{6\text{-}7}$ = 15.6 Hz, $J_{6\text{-}8}$ = 1.7 Hz), 1.71 (dd, 3H, H_8, $J_{7\text{-}8}$ = 7.0 Hz, $J_{6\text{-}8}$ = 1.7 Hz).

^{13}C NMR (150 MHz, CD_3OD) δ 166.8 (C_5), 158.3 (C_4), 142.8 (C_7), 139.7 (C_1), 131.5 (C_{3a}), 127.5 (C_2), 126.3 (C_6), 122.5 (C_3), 18.0 (C_8).

HRMS (m/z ESI$^+$): m/z found 219.1251 $[M+1]^+$; $C_{11}H_{15}N_4O$ required 219.1241

(E)-amino((4-(N-methylbut-2-enamido)phenyl)amino)methaniminium trifluoroacetate, 22 (scheme 4)

Compound **22** was deprotected according to ***Method F.2***. (quant. yield)

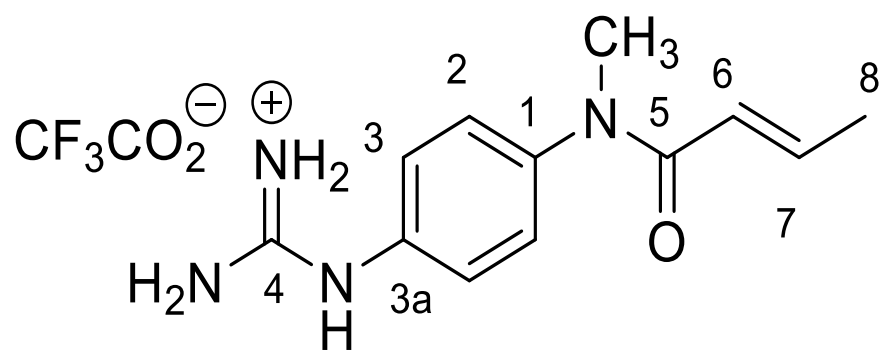

^{1}H NMR (400 MHz, CD_3OD) δ 7.46 – 7.30 (m, 4H, H_2, H_3), 6.90 (dq, 1H, H_7, $J_{6\text{-}7}$ = 15.0 Hz, $J_{13\text{-}14}$ = 6.9 Hz), 5.93 (brd, 1H, H_6), 3.32 (s, 3H, $NC\underline{H}_3$), 1.78 (d, 3H, H_8, $J_{7\text{-}8}$ = 5.9 Hz).

^{13}C NMR (150 MHz, CD_3OD) δ 162.0 (C_5), 158.1 (C_4), 143.7 (C_1), 143.4 (C_7), 135.7 (C_{3a}), 130.0 (C_3), 127.6 (C_2), 123.6 (C_6), 37.3 ($N\underline{C}H_3$), 18.0 (C_8).

HRMS (m/z ESI$^+$): m/z found 233.1392 $[M+1]^+$; $C_{12}H_{17}N_4O$ required 233.1397

Amino((4-(3-methylbut-2-enamido)phenyl)amino)methaniminium trifluoroacetate, 23 (scheme 4)

Compound **23** was deprotected according to ***Method F.2***. (quant. yield)

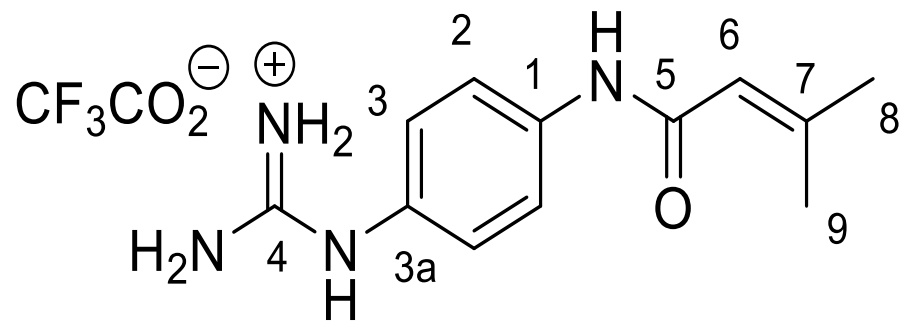

^{1}H NMR (400 MHz, CD_3OD) δ 7.71 (d, 2H, H_3, $J_{2\text{-}3}$ = 8.6 Hz), 7.24 (d, 2H, H_2, $J_{2\text{-}3}$ = 8.6 Hz), 5.92 – 5.87 (m, 1H, H_6), 2.21 (d, 3H, H_8, $J_{6\text{-}8}$ = 1.1 Hz), 1.95 (d, 3H, H_9, $J_{6\text{-}9}$ = 1.1 Hz).

^{13}C NMR (150 MHz, CD_3OD) δ 167.8 (C_5), 158.3 (C_4), 154.8 (C_7), 140.0 (C_1), 131.6 (C_{3a}), 127.3 (C_3), 122.3 (C_2), 119.5 (C_6), 27.5 (C_9), 20.1 (C_8).

HRMS (m/z ESI$^+$): m/z found 233.1389 $[M+1]^+$; $C_{12}H_{17}N_4O$ required 233.1397

Amino((4-(N,3-dimethylbut-2-enamido)phenyl)amino)methaniminium trifluoroacetate, 24 (scheme 4)

Compound **24** was deprotected according to **Method F.2**. (quant. yield)

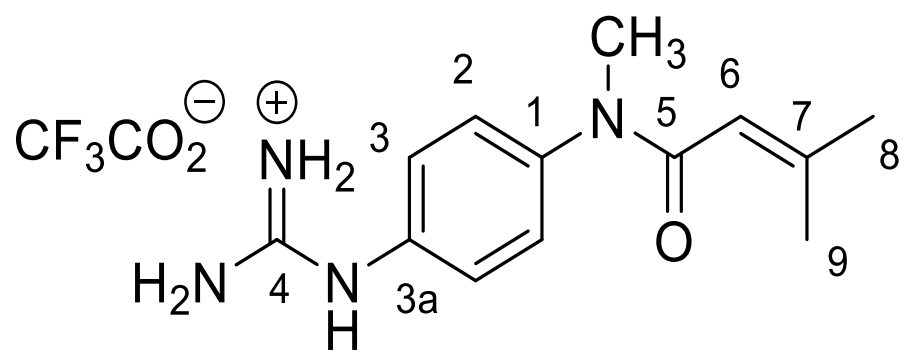

^{1}H NMR (400 MHz, CD_3OD) δ 7.64 (s, 4H, H_2, H_3), 5.61 (brd, 1H, H_6), 3.32 (s, 3H, $NC\underline{H}_3$), 2.07 (s, 3H, H_8), 1.75 (s, 3H, H_9).

^{13}C NMR (150 MHz, CD_3OD) δ 161.9 (C_5), 158.1 (C_4), 152.0 (C_7), 144.2 (C_{3a}), 135.4 (C_1), 129.7 (C_3), 127.4 (C_2), 118.4 (C_6), 37.1 ($N\underline{C}H_3$), 26.9 (C_9), 20.4 (C_8).

HRMS (m/z ESI$^+$): m/z found 247.1560 $[M+1]^+$; $C_{25}H_{37}N_4O_5$ required 247.1554

Amino((4-(cyclohex-1-ene-1-carboxamido)phenyl)amino)methaniminium trifluoroacetate, 25 (scheme 4)

Compound **25** was deprotected according to ***Method F.2***. (quant. yield)

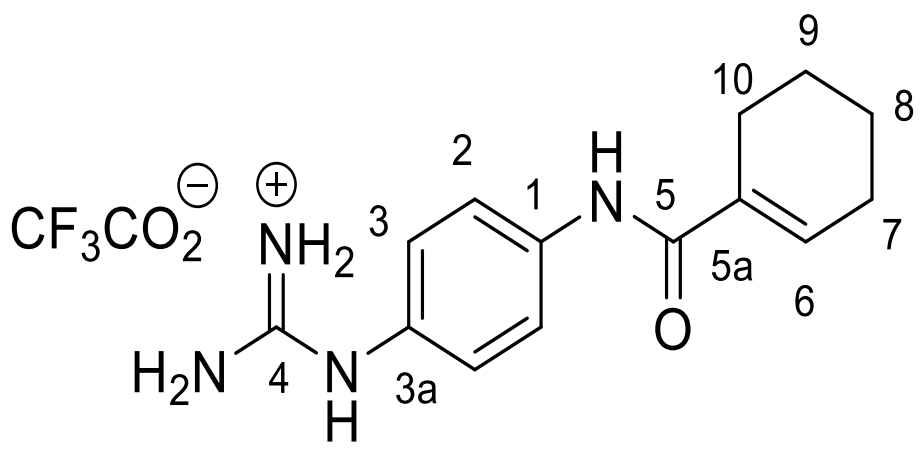

^{1}H NMR (400 MHz, CD$_3$OD) δ 7.72 (d, 2H, H_3, $J_{2\text{-}3}$ = 9.0 Hz), 7.25 (d, 2H, H_2, $J_{2\text{-}3}$ = 9.0 Hz), 6.72 (s, 1H, H_6), 2.40 – 2.20 (m, 4H, H_7, H_{10}), 1.80 – 1.60 (m, 4H, H_8, H_9).

^{13}C NMR (100 MHz, CD$_3$OD) δ 169.5 (C_5), 157.5 (C_4), 138.5 (C_{3a}), 134.1 (C_7), 133.9 (C_6), 130.2 (C_1), 125.9 (C_3), 121.9 (C_2), 25.3 (C_{11}), 24.5 (C_8), 22.2 (C_{10}), 21.7 (C_9).

HRMS (m/z ESI$^+$): m/z found 259.1549 [M+1]$^+$; $C_{14}H_{19}N_4O$ required 259.1554

Amino((4-(N-methylcyclohex-1-ene-1-carboxamido)phenyl)amino)methaniminium trifluoroacetate, 26 (scheme 4)

Compound **26** was deprotected according to ***Method F.2***. (quant. yield)

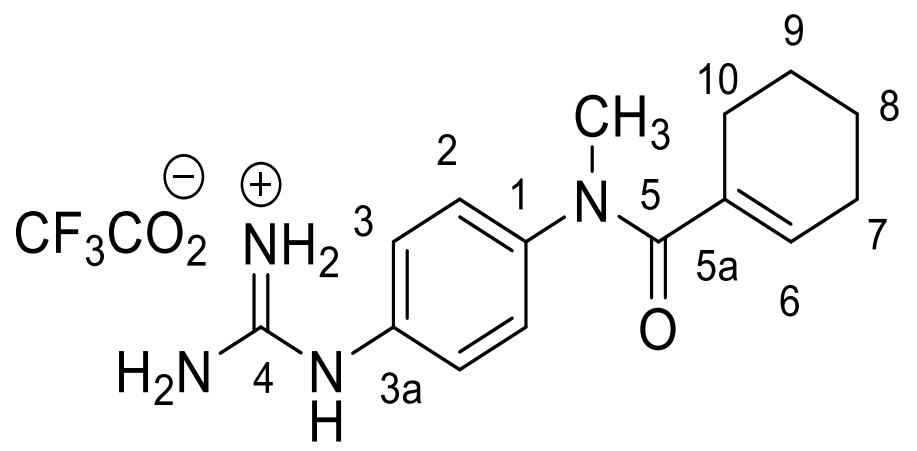

^{1}H NMR (400 MHz, CD_3OD) δ 7.34 (s, 4H, H_2, H_3), 5.86 (s, 1H, H_6), 3.36 (s, 3H, NC$\underline{H}_3$), 2.10 – 1.90 (m, 4H, H_7, H_{10}), 1.60 – 1.45 (m, 4H, H_8, H_9).

^{13}C NMR (100 MHz, CD_3OD) δ 174.9 (C_5), 158.1 (C_4), 144.9 (C_1), 141.1 (C_{5a}), 135.4 (C_{3a}), 133.7 (C_6), 129.2 (C_3), 127.2 (C_2), 38.4 (N$\underline{C}H_3$), 26.9 (C_7), 25.8 (C_8), 23.1 (C_9), 22.5 (C_{10}).

HRMS (m/z ESI$^+$): m/z found 273.1703 $[M+1]^+$; $C_{15}H_{21}N_4O$ required 273.1710

Amino((4-(3,4-dihydro-2H-pyran-6-carboxamido)phenyl)amino)methaniminium trifluoroacetate, 27 (scheme 4)

Compound **27** was deprotected according to ***Method F.2***. (quant. yield)

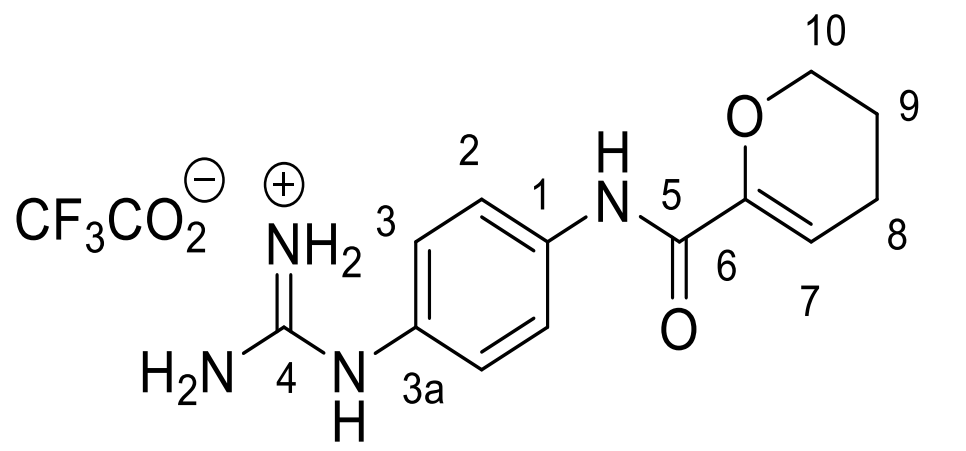

^{1}H NMR (400 MHz, CD$_3$OD) δ 7.78 (d, 2H, H_3, J_{2-3} = 9.0 Hz), 7.26 (d, 2H, H_2, J_{2-3} = 9.0 Hz), 6.07 (d, 1H, H_7, J_{7-8} = 4.1 Hz), 5.20 (d, 2H, H_{10}, J_{9-10} = 4.9 Hz), 2.20 – 2.10 (m, 2H, H_8), 1.97 – 1.87 (m, 2H, H_9).

^{13}C NMR (150 MHz, CD$_3$OD) δ 163.1 (C_5), 158.3 (C_4), 147.3 (C_6), 138.7 (C_{3a}), 132.0 (C_1), 127.3 (C_3), 123.4 (C_2), 109.2 (C_7), 67.9 (C_{10}), 22.8 (C_9), 21.5 (C_8).

HRMS (m/z ESI$^+$): m/z found 261.1359 $[M+1]^+$; $C_{13}H_{17}N_4O_2$ required 261.1347

Amino((4-cinnamamidophenyl)amino)methaniminium trifluoroacetate, 28 (scheme 4)

Compound **28** was deprotected according to ***Method F.2***. (quant. yield)

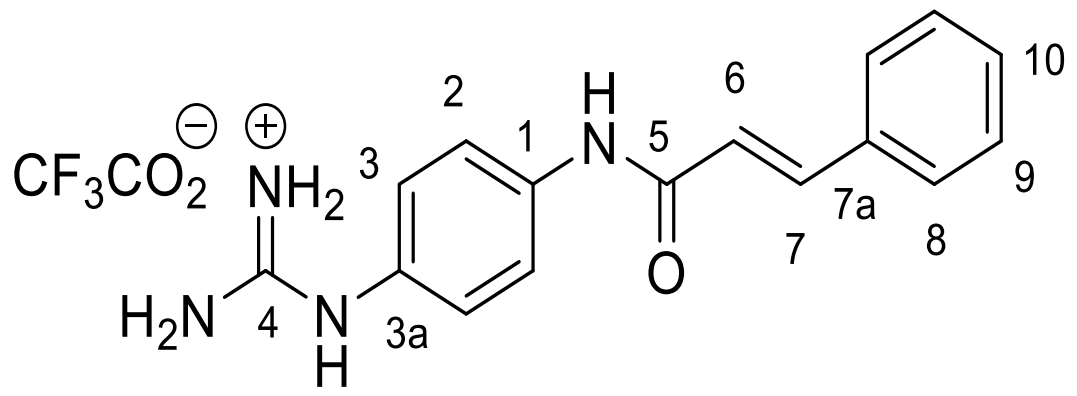

^{1}H NMR (400 MHz, CD_3OD) δ 7.81 (d, 2H, H_3, $J_{2\text{-}3}$ = 8.9 Hz), 7.72 (d, 1H, H, $J_{6\text{-}7}$ = 15.7 Hz), 7.63 (d, 2H, H_8, $J_{8\text{-}9}$ = 7.8 Hz), 7.55 - 7.35 (m, 1H, H_9, H_{10}), 7.63 (d, 2H, H_2, $J_{2\text{-}3}$ = 8.9 Hz), 6.67 (d, 1H, H_6, $J_{6\text{-}7}$ = 15.7 Hz).

^{13}C NMR (150 MHz, CD_3OD) δ 166.8 (C_5), 158.3 (C_4), 143.3 (C_7), 139.8 (C_{7a}), 136.1 (C_{3a}), 131.6 (C_1), 131.2 (C_{10}), 130.1 (C_9), 129.0 (C_8), 127.6 (C_2), 122.5 (C_3), 121.9 (C_6).

HRMS (m/z ESI$^+$): m/z found 281.1406 $[M+1]^+$; $C_{26}H_{33}N_4O_5$ required 281.1397

Amino((4-(N-methylcinnamamido)phenyl)amino)methaniminium trifluoroacetate, 29 (scheme 4)

Compound **29** was deprotected according to ***Method F.2***. (quant. yield)

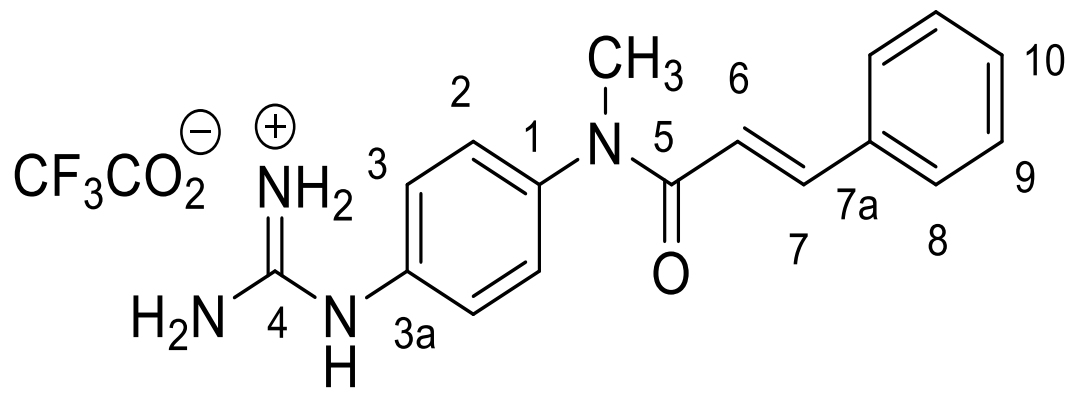

^{1}H NMR (400 MHz, CD_3OD) δ 7.64 (d, 2H, H_7, $J_{6\text{-}7}$ = 15.9 Hz), 7.50 – 7.28 (m, 9H, H_2, H_3, H_8, H_9 H_{10}) 6.53 (brd, 1H, H_6), 3.42 (s, 3H, $NC\underline{H}_3$).

^{13}C NMR (150 MHz, CD_3OD) δ 168.2 (C_{15}), 162.4 (C_4), 158.1 (C_{3a}), 143.7 (C_7, C_{7a}), 136.2 (C_1), 131.1 (C_8), 130.0 (C_{10}), 129.9 (C_9), 129.0 (C_2), 127.6 (C_3), 119.4 (C_6), 38.0 ($N\underline{C}H_3$).

HRMS (m/z ESI$^+$): m/z found 295.1561 $[M+1]^+$; $C_{17}H_{19}N_4O$ required 295.1554

(E)-amino((4-(3-(4-methoxyphenyl)acrylamido)phenyl)amino)methaniminium trifluoroacetate, 30 (scheme 4)

Compound **30** was deprotected according to **Method F.2**. (quant. yield)

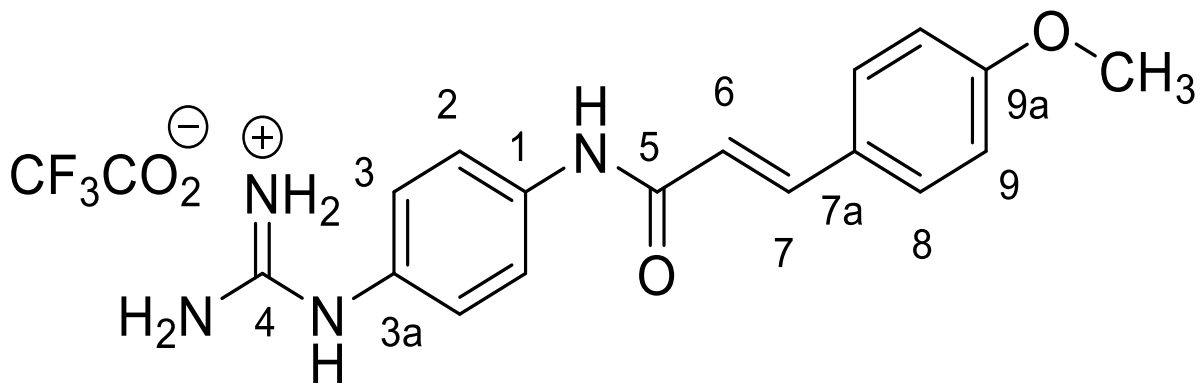

^{1}H NMR (400 MHz, CD_3OD) δ 7.80 (d, 2H, H_3, $J_{2\text{-}3}$ = 8.7 Hz), 7.66 (d, 1H, H_7, $J_{6\text{-}7}$ = 15.3 Hz), 7.59 (d, 2H, H_2, $J_{2\text{-}3}$ = 8.7 Hz), 7.29 (d, 2H, H_8, $J_{8\text{-}9}$ = 8.7 Hz), 7.00 (d, 1H, H_9, $J_{8\text{-}9}$ = 8.7 Hz), 6.67 (d, 1H, H_6, $J_{6\text{-}7}$ = 15.3 Hz), 3.86 (s, 3H, $OC\underline{H}_3$).

^{13}C NMR (100 MHz, CD_3OD) δ 167.3 (C_5), 163.0 (C_{9a}), 158.3 (C_4), 143.2 (C_7), 139.9 (C_{7a}), 131.5 (C_{3a}), 130.7 (C_2), 128.7 (C_1), 127.5 (C_8), 122.4 (C_3), 119.2 (C_6), 115.5 (C_9), 55.9 ($O\underline{C}H_3$).

HRMS (m/z ESI$^+$): m/z found 311.1517 $[M+1]^+$; $C_{17}H_{19}N_4O_{02}$ required 311.1503

(E)-amino((4-(3-(4-methoxyphenyl)-N-methylacrylamido)phenyl)amino)methaniminium trifluoroacetate, 31 (scheme 4)

Compound **31** was deprotected according to ***Method F.2***. (quant. yield)

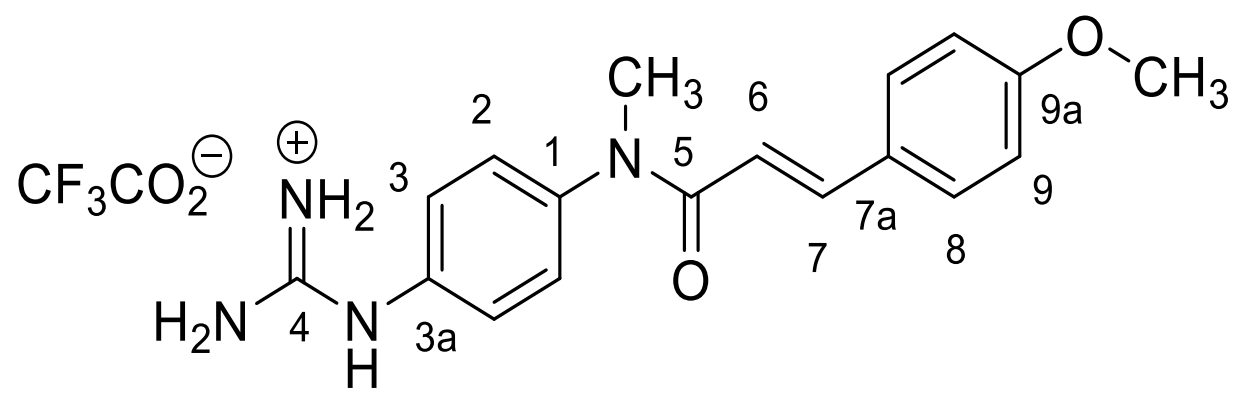

^{1}H NMR (400 MHz, CD_3OD) δ 7.60 (d, 1H, H_7, $J_{6\text{-}7}$ = 16.1 Hz), 7.43 (s, 4H, H_2, H_3), 7.36 (d, 2H, H_8, $J_{8\text{-}9}$ = 8.0 Hz), 6.89 (d, 2H, H_9, $J_{8\text{-}9}$ = 8.0 Hz), 6.40 (brd, 1H, H_6), 3.80 (s, 3H, $OC\underline{H}_3$), 3.42 (s, 3H, $NC\underline{H}_3$).

^{13}C NMR (150 MHz, CD_3OD) δ 168.7 (C_5), 162.8 (C_{9a}), 158.1 (C_4), 143.8 (C_7), 143.7 (C_{7a}), 135.7 (C_1), 130.7 (C_8), 130.0 (C_3), 128.8 (C_{3a}), 127.6 (C_2), 116.7 (C_6), 115.3 (C_9), 55.8 ($O\underline{C}H_3$), 38.0 ($N\underline{C}H_3$).

HRMS (m/z ESI$^+$): m/z found 325.1663 $[M+1]^+$; $C_{18}H_{21}N_4O_2$ required 325.1660

Amino((4-(but-2-ynamido)phenyl)amino)methaniminium trifluoroacetate, 32 (scheme 4)

Compound **32** was deprotected according to ***Method F.2***. (quant. yield)

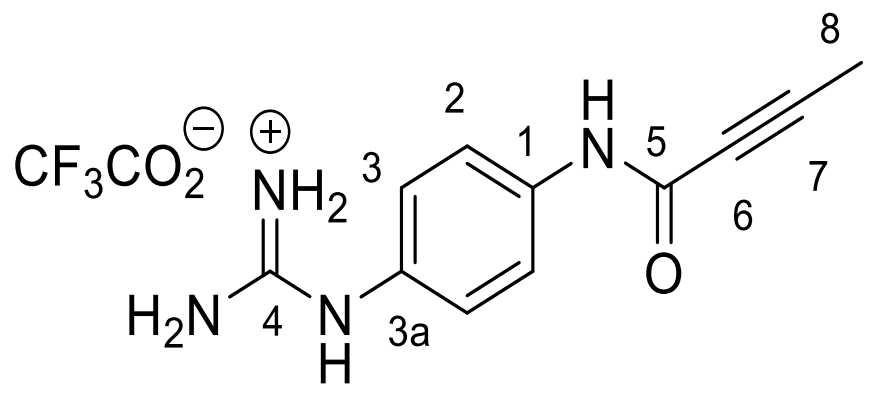

^{1}H NMR (400 MHz, CD_3OD) δ 7.68 (d, 2H, H_3, $J_{2\text{-}3}$ = 8.6 Hz), 7.25 (d, 2H, H_2, $J_{2\text{-}3}$ = 8.6 Hz), 2.06 (s, 3H, H_8).

^{13}C NMR (150 MHz, CD_3OD) δ 162.0 (C_5), 158.3 (C_4), 139.1 (C_{3a}), 131.9 (C_1), 127.5 (C_2), 122.4 (C_3), 86.2 (C_6), 75.6 (C_7), 3.7 (C_8).

HRMS (m/z ESI$^+$): m/z found 217.1085 $[M+1]^+$; $C_{11}H_{13}N_4O$ required 217.1085

Pyrone derivatives

Amino((4-oxo-4H-chromen-6-yl)amino)methaniminium trifluoroacetate, 33 (scheme S5)

Compound **33** was deprotected according to ***Method F.2***. (quant. yield)

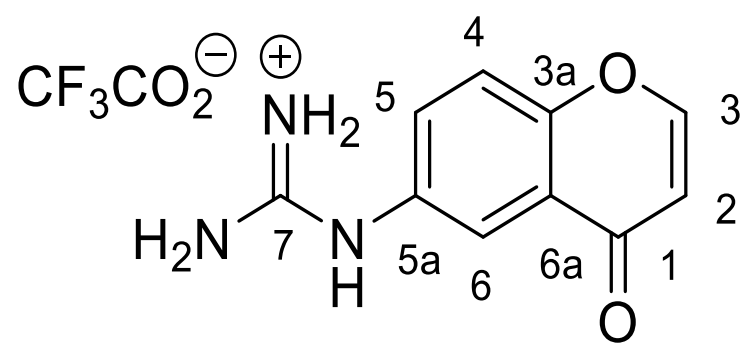

^{1}H NMR (400 MHz, CD_3OD) δ 8.23 (d, 1H, H_3, $J_{2\text{-}3}$ = 6.0 Hz), 8.03 (dd, 1H, H_5, $J_{4\text{-}5}$ = 2.3 Hz, $J_{5\text{-}6}$ = 0.8 Hz), 7.73 (d, 1H, H_6, $J_{5\text{-}6}$ = 0.8 Hz), 7.72 (d, 1H, H_4, $J_{4\text{-}5}$ = 2.3 Hz), 6.43 (d, 1H, H_2, $J_{2\text{-}3}$ = 6.0 Hz).

^{13}C NMR (150 MHz, CD_3OD) δ 179.0 (C_1), 158.7 (C_3), 156.8 (C_{3a}), 133.9 (C_{5a}), 132.9 (C_5), 126.5 (C_{6a}), 122.8 (C_4), 121.8 (C_6), 113.3 (C_2).

HRMS (m/z ESI$^+$): m/z found 204.0779 $[M+1]^+$; $C_{10}H_{210}N_3O_2$ required 204.0768

Amino((2-oxo-2H-chromen-6-yl)amino)methaniminium trifluoroacetate, 34 (scheme S5)

Compound **34** was deprotected according to ***Method F.2***. (quant. yield)

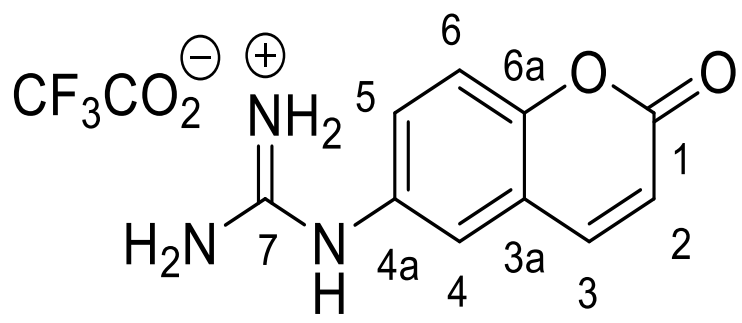

^{1}H NMR (400 MHz, CD_3OD) δ 7.98 (d, 1H, H_3, $J_{2\text{-}3}$ = 9.5 Hz), 7.62 (d, 1H, H_4, $J_{4\text{-}5}$ = 2.5 Hz), 7.52 (dd, 1H, H_5, $J_{4\text{-}5}$ = 8.7 Hz, $J_{5\text{-}6}$ = 2.5 Hz), 7.46 (d, 1H, H_6, $J_{5\text{-}6}$ = 2.3 Hz), 6.53 (d, 1H, H_2, $J_{2\text{-}3}$ = 9.5 Hz).

^{13}C NMR (150 MHz, CD_3OD) δ 160.7 (C_1), 157.0 (C_7), 152.9 (C_{6a}), 142.3 (C_3), 131.1 (C_{4a}), 129.4 (C_5), 125.4(C_4), 120.0 (C_{3a}), 117.9 (C_6), 116.9(C_2).

HRMS (m/z ESI$^+$): m/z found 204.0777 $[M+1]^+$; $C_{10}H_{210}N_3O_2$ required 204.0768

Benzothiazole derivatives

((2-acetylbenzothiazol-6-yl)amino)methaniminium trifluoroacetate, 35 (scheme 5)

Compound **35** was deprotected according to ***Method F.2***. (quant. yield)

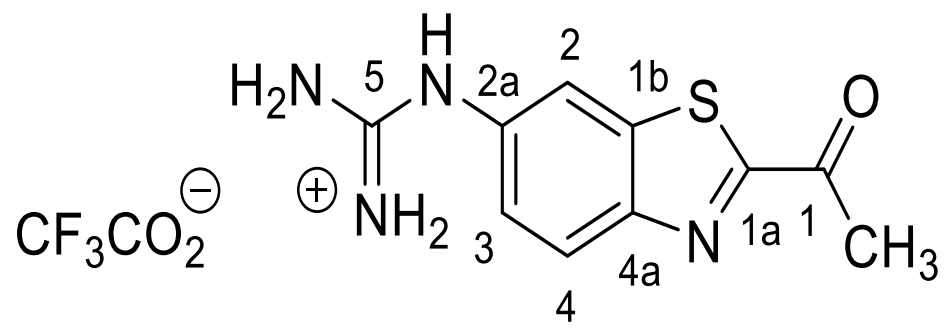

^{1}H NMR (400 MHz, CD_3OD) δ 8.24 (d, 1H, H_4, $J_{3\text{-}4}$ = 8.8 Hz), 8.05 (d, 1H, H_2, $J_{2\text{-}3}$ = 2.1 Hz), 7.52 (dd, 1H, H_3, $J_{3\text{-}4}$ = 8.8 Hz, $J_{2\text{-}3}$ = 2.1 Hz), 2.77 (s, 3H, $\underline{C}H_3$).

^{13}C NMR (100 MHz, CD_3OD) δ 192.6 (C_1), 167.5 (C_{1a}), 156.8 (C_5), 152.1 (C_{2a}), 138.4 (C_{4a}), 134.7 (C_{1b}), 126.1 (C_4), 124.4 (C_3), 118.7 (C_2), 24.6 ($N\underline{C}H_3$).

HRMS (m/z ESI^+): m/z found 235.0653 $[M+1]^+$; $C_{10}H_{11}N_4OS$ required 235.0649

Amino((2-(2-phenylacetyl)benzothiazol-6-yl)amino)methaniminium trifluoroacetate, 36 (scheme 5)

Compound **36** was deprotected according to ***Method F.2***. (quant. yield)

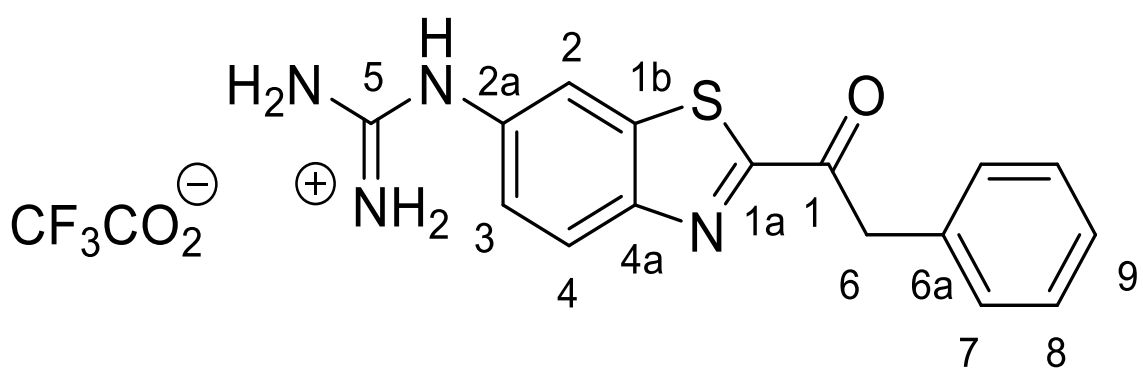

^{1}H NMR (400 MHz, CD_3OD) δ 8.32 (d, 1H, H_4, $J_{3\text{-}4}$ = 8.5 Hz), 8.07 (d, 1H, H_2, $J_{2\text{-}3}$ = 1.9 Hz), 7.55 (dd, 1H, H_3, $J_{2\text{-}3}$ = 8.5 Hz, $J_{3\text{-}4}$ = 1.9 Hz), 7.38 (d, 2H, H_7, $J_{7\text{-}8}$ = 7.4 Hz), 7.33 (t, 2H, H_8, $J_{7\text{-}8}$ = 7.4 Hz, $J_{7\text{-}8}$ = 7.7 Hz), 7.26 (t, 1H, H_9, $J_{8\text{-}9}$ = 7.2 Hz), 4.58 (s, 2H, H_6).

^{13}C NMR (150 MHz, CD_3OD) δ 193.7 (C_1), 158.2 (C_5), 153.6 (C_{3a}), 139.9 (C_{4a}), 136.2 (C_{1b}), 135.0 (C_{6a}), 131.0 (C_7), 129.6 (C_8), 128.1 (C_9), 127.7 (C_4), 125.9 (C_3), 120.2 (C_2), 45.6 (C_6).

HRMS (m/z ESI$^+$): m/z found 311.3817 $[M+1]^+$; $C_{16}H_{15}N_4OS$ required 311.3825

Amino((2-benzoylbenzothiazol-6-yl)amino)methaniminium trifluoroacetate, 37 (scheme 5)

Compound **37** was deprotected according to ***Method F.2***. (quant. yield)

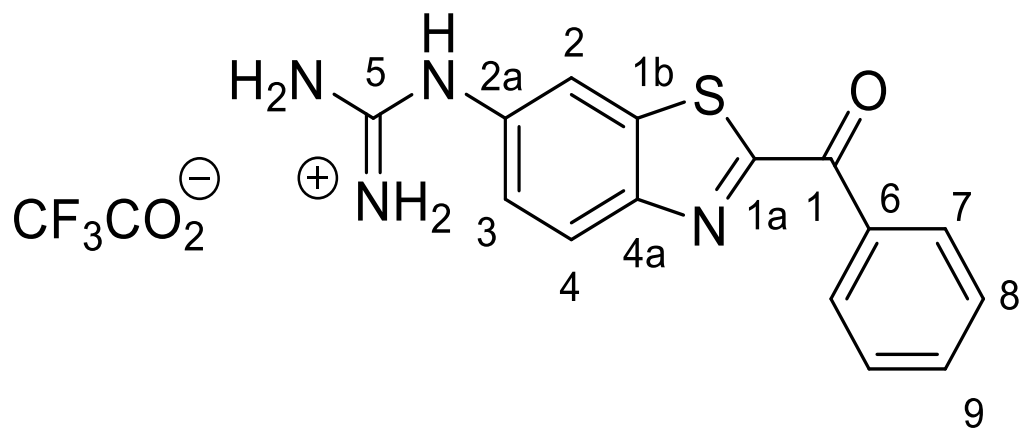

^{1}H NMR (400 MHz, CD_3OD) δ 8.56 (d, 2H, H_7, $J_{7\text{-}8}$ = 7.8 Hz), 8.32 (d, 1H, H_4, $J_{3\text{-}4}$ = 8.7 Hz), 8.12 (d, 1H, H_2, $J_{2\text{-}3}$ = 2.2 Hz), 7.75 (t, 1H, H_9, $J_{8\text{-}9}$ = 7.8 Hz), 7.61 (d, 2H, H_8, $J_{7\text{-}8}$ = $J_{8\text{-}9}$ = 7.8 Hz), 7.56 (dd, 1H, H_3, $J_{3\text{-}4}$ = 8.7 Hz, $J_{2\text{-}3}$ = 2.2 Hz).

^{13}C NMR (100 MHz, CD_3OD) δ 184.7 (C_1), 168.1 (C_5), 156.8 (C_{2a}), 152.5 (C_{1a}), 138.0 (C_{4a}), 134.7 (C_{1b}, C_{16}), 133.9 (C_9), 130.8 (C_7), 128.2 (C_8), 126.4 (C_4), 124.5 (C_3), 118.5 (C_2).

HRMS (m/z ESI$^+$): m/z found 397.3561 $[M+1]^+$; $C_{15}H_{13}N_4OS$ required 397.3555

Amino(benzothiazol-6-ylamino)methaniminium trifluoroacetate, 38 (scheme 5)

Compound **38** was deprotected according to ***Method F.2***. (quant. yield)

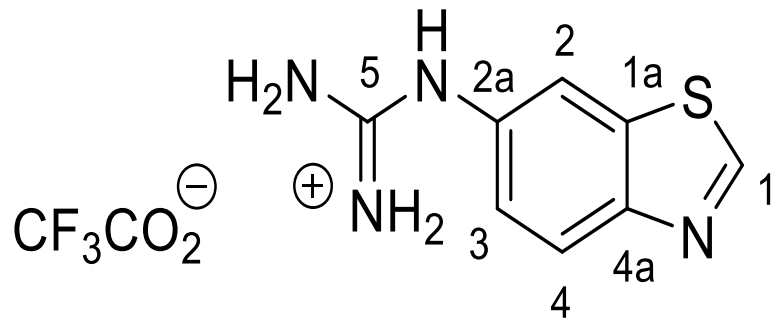

^{1}H NMR (400 MHz, CD_3OD) δ 9.33 (s, 1H, H_1), 8.17 (d, 1H, H_4, $J_{3\text{-}4}$ = 8.7 Hz), 8.07 (d, 1H, H_2, $J_{2\text{-}3}$ = 2.0 Hz), 7.49 (dd, 1H, H_3, $J_{3\text{-}4}$ = 8.7 Hz, $J_{2\text{-}3}$ = 2.0 Hz).

^{13}C NMR (150 MHz, CD_3OD) δ 158.4 (C_1), 153.4 (C_5), 136.4 (C_{1a}), 133.9 (C_{2a}), 125.6 (C_3), 125.1 (C_4), 120.8 (C_2).

HRMS (m/z ESI$^+$): m/z found 193.0551 $[M+1]^+$; $C_8H_9N_4S$ required 193.0543

Amino((4-((6-((amino(iminio)methyl)amino)benzothiazol-2-yl)amino)phenyl)amino) methaniminium trifluoroacetate, 39 (scheme S4)

Compound **39** was prepared according to ***Method F.2***. (quant. yield)

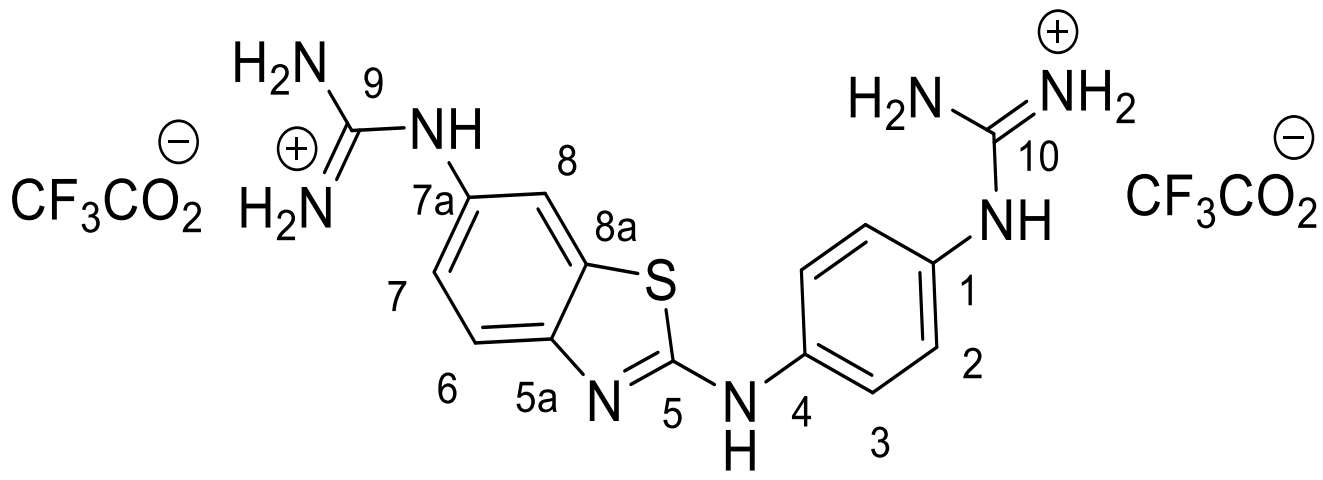

^{1}H NMR (400 MHz, CD_3OD) δ 7.53 (d, 2H, H_3, $J_{2\text{-}3}$ = 8.4 Hz), 7.43 (d, 1H, H_6, $J_{6\text{-}7}$ = 9.1 Hz), 7.39 (dd, 1H, H_7, $J_{7\text{-}8}$ = 9.1 Hz, $J_{6\text{-}7}$ = 2.1 Hz), 7.30 – 7.22 (m, 3H, H_2, H_8).

^{13}C NMR (150 MHz, CD_3OD) δ 168.3 (C_9, C_{10}), 152.2 (C_{7a}), 149.3 (C_5), 145.7 (C_{5a}), 144.3 (C_4), 134.1 (C_{8a}), 130.4 (C_2), 121.5 (C_8), 116.6 (C_1), 114.8 (C_7), 111.9 (C_3), 104.0 (C_6).

HRMS (m/z ESI$^+$): m/z found 171.0698 $[M+2]^{2+}$; $C_{35}H_{49}N_8O_8S$ required 171.0682

Amino((4-(benzothiazol-2-ylamino)phenyl)amino)methaniminium trifluoroacetate, 40 (scheme S6)

Compound **40** was prepared according to ***Method F.2***. (quant. yield)

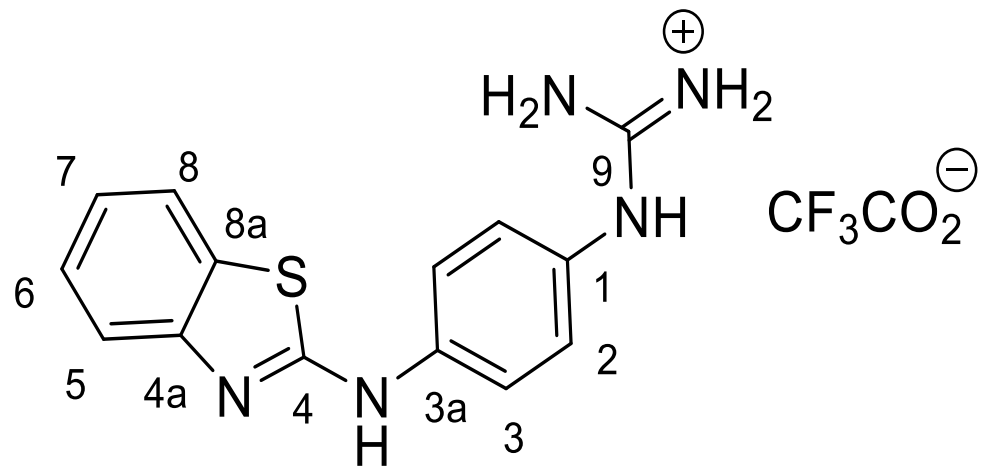

^{1}H NMR (400 MHz, CD_3OD) δ 7.85 (d, 2H, H_2, $J_{2\text{-}3}$ = 8.9 Hz), 7.73 (d, 1H, H_5, $J_{5\text{-}6}$ = 7.8 Hz), 7.62 (d, 1H, H_8, $J_{7\text{-}8}$ = 7.8 Hz), 7.37 (t, 1H, H_7, $J_{6\text{-}7}$ = $J_{7\text{-}8}$ = 7.8 Hz), 7.31 (d, 2H, H_3, $J_{2\text{-}3}$ = 8.9 Hz), 7.20 (t, 1H, H_6, $J_{5\text{-}6}$ = $J_{6\text{-}7}$ = 7.8 Hz).

^{13}C NMR (100 MHz, CD_3OD) δ 164.4 (C_4), 158.5 (C_9), 152.6 (C_{8a}), 141.5 (C_{3a}), 131.2 (C_{4a}), 130.3 (C_1), 128.0 (C_2), 127.2 (C_7), 124.0 (C_6), 122.0 (C_5), 121.0 (C_3), 120.2 (C_8).

HRMS (m/z ESI$^+$): m/z found 284.0972 $[M+1]^+$; $C_{14}H_{15}N_5S$ required 284.0965

Bis-Boc-protected derivatives

Methyl 4-(2,3-bis(*tert*-butoxycarbonyl)guanidino)benzoate, Boc-1 (scheme 1)

Compound **Boc-1** was prepared according to ***Method E.1*** from *p*-Amino methyl (90% yield)

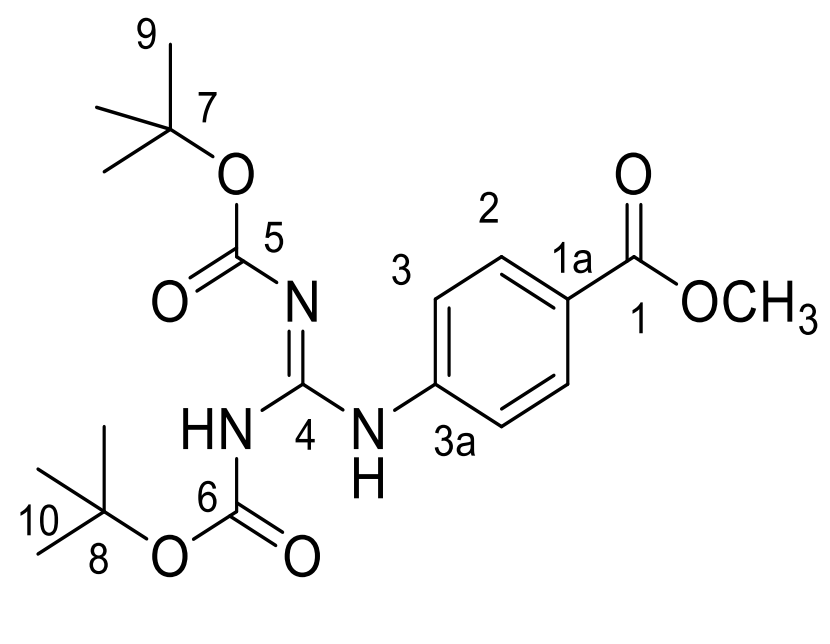

^{1}H NMR (400 MHz, DMSO-d$_6$) δ 11.20 (s, 1H, N<u>H</u>Boc), 10.16 (s, 1H, N<u>H</u>Ar), 7.91 (d, 2H, H_3, $J_{2\text{-}3}$ = 8.7 Hz), 7.71 (d, 2H, H_2, $J_{2\text{-}3}$ = 8.7 Hz), 3.81 (s, 3H, OC<u>H</u>$_3$), 1.49 (s, 9H, C(C<u>H</u>$_3$)$_3$), 1.40 (s, 9H, C(C<u>H</u>$_3$)$_3$).

^{13}C NMR (150 MHz, DMSO-d$_6$) δ 166.2 (C_1), 162.5 (C_4), 152.4 (C_5), 152.3 (C_6), 141.9 (C_{3a}), 130.4 (C_3), 125.5 (C_{1a}), 121.9 (C_2), 83.8 (C_7), 79.6 (C_8), 52.5 (O<u>C</u>H$_3$), 28.3 (C_9), 28.1 (C_{10}).

HRMS (m/z ESI$^+$): m/z found 394.1966 $[M+1]^+$; $C_{19}H_{28}N_3O_6$ required 394.1973

Octyl 4-(2,3-bis(*tert*-butoxycarbonyl)guanidino)benzoate, Boc-2 (scheme 1)

Compound **Boc-2** was prepared according to ***Method E.1*** (32% yield)

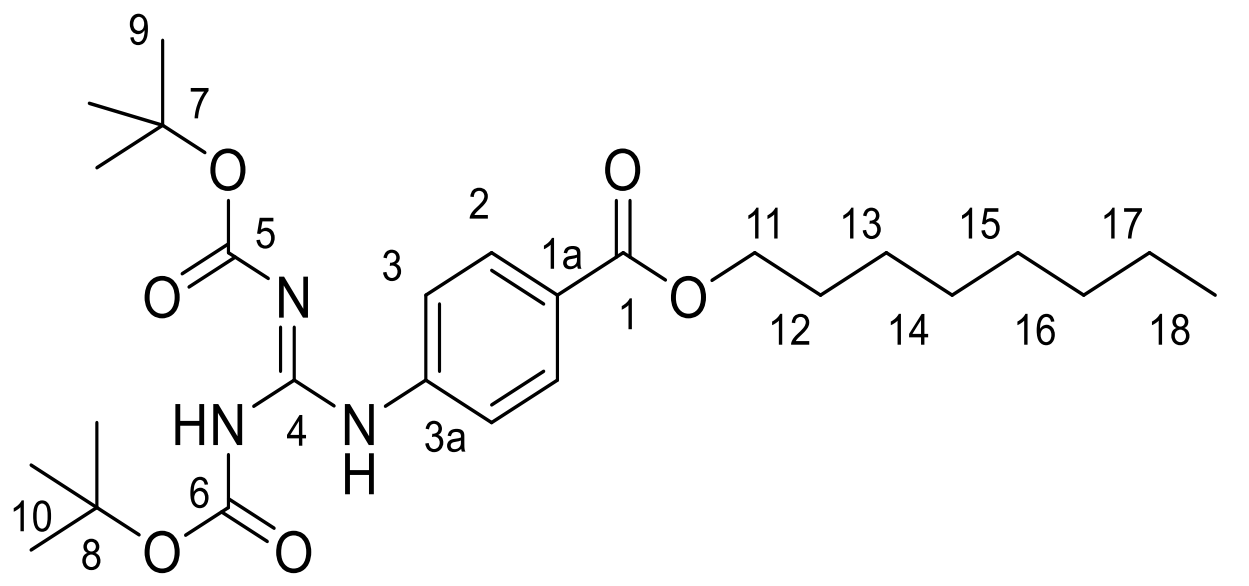

^{1}H NMR (400 MHz, DMSO-d$_6$) δ 11.20 (s, 1H, N<u>H</u>Boc), 10.16 (s, 1H, N<u>H</u>Ar), 7.90 (d, 2H, H_3, $J_{2\text{-}3}$ = 8.8 Hz), 7.71 (d, 2H, H_2, $J_{2\text{-}3}$ = 8.8 Hz), 4.22 (t, 2H, H_4, $J_{4\text{-}5}$ = 6.8 Hz), 1.62 (q, 2H, H_{12}, $J_{4\text{-}5}$ = $J_{5\text{-}6}$ = 6.9 Hz), 1.60 – 1.15 (m, 28H, H_9, H_{10}, H_{13}, H_{14}, H_{15}, H_{16}, H_{17}), 0.84 (t, 3H, H_{18}, $J_{17\text{-}18}$ = 6.6 Hz).

^{13}C NMR (100 MHz, DMSO-d$_6$) δ 165.2 (C_1), 162.1 (C_4), 151.9 (C_5, C_6), 141.4 (C_{1a}), 129.9 (C_2), 125.3 (C_{3a}), 121.4 (C_2), 83.3 (C_7), 79.1 (C_8), 64.5 (C_{11}), 31.2 (C_{17}), 28.6 (C_{14}), 28.5 (C_{15}), 28.1 (C_{12}), 27.8 (C_9), 27.6 (C_{10}), 25.4 (C_{16}), 22.0 (C_{13}), 13.9 (C_{18}).

HRMS (m/z ESI$^+$): m/z found 492.3081 $[M+1]^+$; $C_{26}H_{42}N_3O_6$ required 492.3069

Prop-2-yn-1-yl 4-(2,3-bis(*tert*-butoxycarbonyl)guanidino)benzoate, Boc-3 (scheme 1)

Compound **Boc-3** was prepared according to ***Method E.1***. (34% yield)

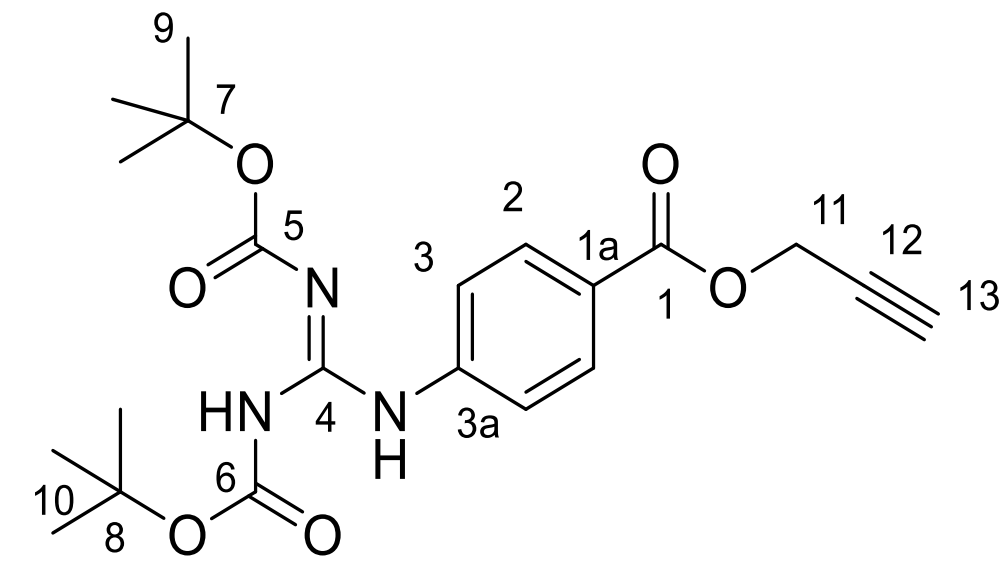

^{1}H NMR (400 MHz, DMSO-d$_6$) δ 11.18 (s, 1H, NHBoc), 10.18 (s, 1H, NHAr), 7.92 (d, 2H, H_3, J_{2-3} = 8.5 Hz), 7.73 (d, 2H, H_2, J_{2-3} = 8.5 Hz), 4.91 (d, 2H, H_{11}, J_{11-13} = 1.4 Hz), 3.29 (s, 1H, H_{13}), 1.49 (s, 9H, $C(CH_3)_3$), 1.40 (s, 9H, $C(CH_3)_3$).

^{13}C NMR (100 MHz, DMSO-d$_6$) δ 164.9 (C_1), 162.5 (C_4), 152.4 (C_5), 152.2 (C_6), 142.3 (C_{5a}), 130.6 (C_3), 124.8 (C_{1a}), 121.9 (C_2), 83.8 (C_7), 79.4 (C_8), 78.9 (C_{12}), 78.3 (C_{13}), 28.3 (C_9), 28.1 (C_{10}).

HRMS (m/z ESI$^+$): m/z found 418.1790 $[M+1]^+$; $C_{21}H_{28}N_3O_6$ required 418.1973

AWR_285: phenyl 4-{[(1E)-{[(tert-butoxy)carbonyl]amino}({[(tert-butoxy)carbonyl]imino})methyl]amino}benzoate, Boc-4 (scheme 1)

Following method **X**, **X** was produced as a white solid (100 mg, 78% yield)

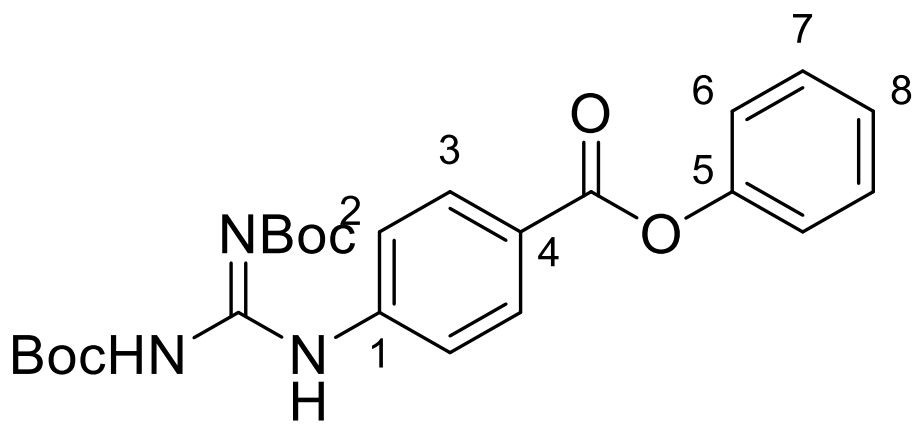

δ$_H$ (400 MHz, *d$_6$*-DMSO); 11.19 (s, 1H, NH), 10.23 (s, 1H, NH), 8.09 (d, J = 8.6 Hz, 2H, H-2 and H-2'), 7.80 (d, J = 8.6 Hz, 2H, H-3 and H-3'), 7.45 (t, J = 7.9 Hz, 2H, H-7 and H-7'), 7.32 – 7.27 (m, 1H, H-8), 7.26 (d, J = 7.9 Hz, 2H, H-6 and H-6'), 1.49 (s, 4H), 1.41 (s, 5H).

δ$_C$ (151 MHz, *d$_6$*-DMSO);

HRMS (m/z ESI+): Found ([M+H]$^+$ $C_{24}H_{29}N_3O_6$ Requires 456.2056)

4-(2-methoxy-2-oxoethyl)phenyl 4-{[(1E)-{[(tert-butoxy)carbonyl]amino}({[(tert-butoxy)carbonyl]imino})methyl]amino}benzoate, Boc-5 (scheme 1)

Following method **X**, **X** was obtained as a white solid (150 mg, 64% yield)

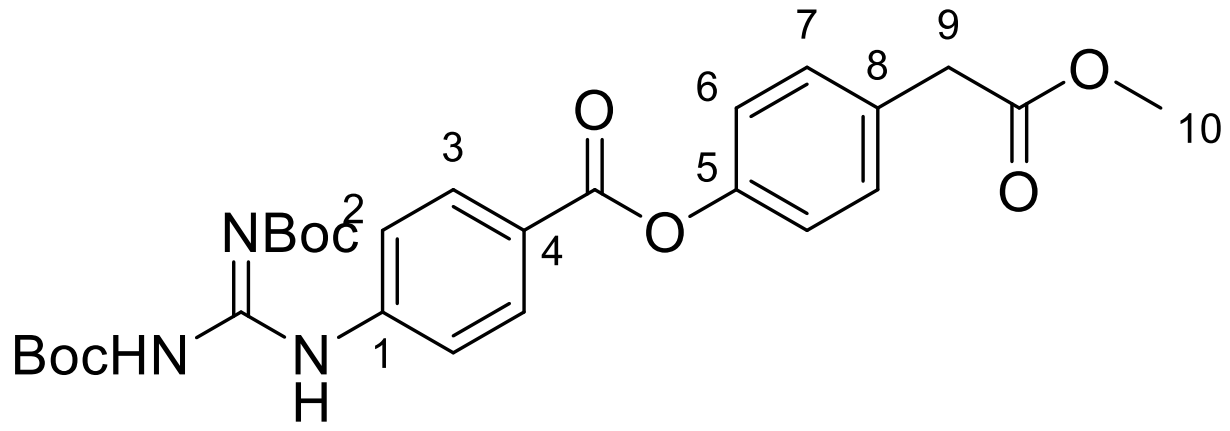

δ$_H$ (400 MHz, $CDCl_3$); 11.22 (s, 1H, NH), 10.27 (s, 1H, NH), 8.10 (d, J = 8.6 Hz, 2H, H-2 and H-2'), 7.82 (d, J = 8.5 Hz, 2H, H-6 and H-6'), 7.36 (d, J = 8.6 Hz, 2H, H-3 and H-3'), 7.23 (d, J = 8.5 Hz, 2H, H-7 and H-7'), 3.74 (s, 2H, H-9), 3.64 (s, 3H, H-10), 1.51 (s, 9H, $C(CH_3)_3$), 1.44 (s, 9H, $C(CH_3)_3$).

δ$_C$ (151 MHz,); 171.60 (s, CO), 164.10 (s, CO), 162.02 (s, CN), 151.93 (s, CO), 151.76 (s, CO), 149.53 (s, C-1), 142.18 (s, C-5), 132.07 (s, C-4), 130.75 (s, C-2), 130.52 (s, C-3), 124.19 (s), 121.80 (s, C-7), 121.47 (s, C-6), 83.37 (s, $\underline{C}(CH_3)_3$), 79.21 (s, $\underline{C}(CH_3)_3$), 51.76 (s, C-10), 39.39 (s, C-9), 27.87 (s, $C(CH_3)_3$), 27.68 (s, $C(CH_3)_3$).

HRMS (m/z ESI+): Found ([M+H]$^+$ $C_{27}H_{33}N_3O_8$ Requires 528.2268)

1,3-di-tert-butyl 2-{[4-(4-{[(1E)-{[(tert-butoxy)carbonyl]amino}({[(tert-butoxy)carbonyl]imino})methyl]amino}benzoyloxy)phenyl]imino}imidazolidine-1,3-dicarboxylate, Boc-6 (scheme 2)

Following method **E.3**, **X**this compound was produced as a white solid (150 mg, 87%yield)

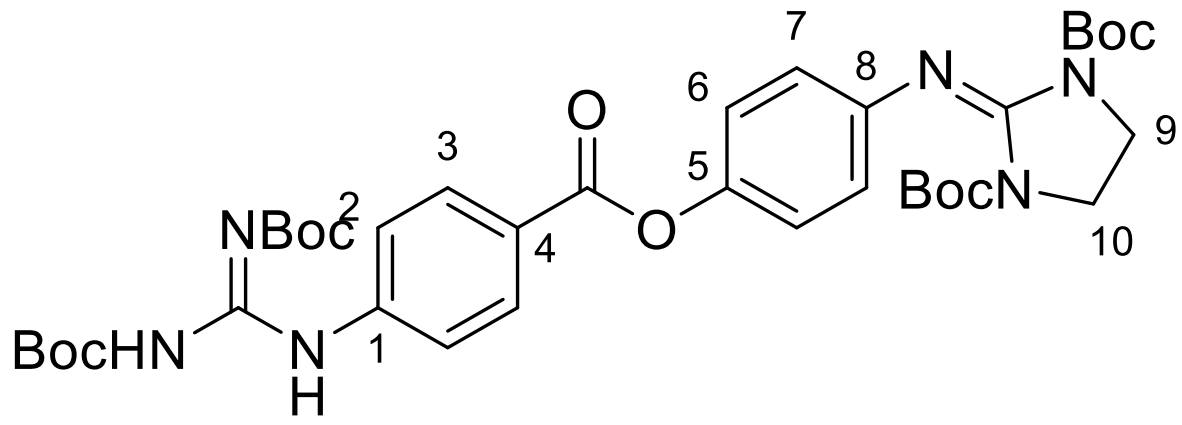

δ_H (400 MHz, $CDCl_3$); 11.63 (s, 1H, NH), 10.63 (s, 1H, NH), 8.16 (d, J = 8.7 Hz, 2H, H-2 and H-2'), 7.81 (d, J = 8.7 Hz, 2H, H-3 and H-3'), 7.07 (td, J = 8.9, 2.2 Hz, 4H), 3.85 (s, 4H), 1.38 (s, 16H).

δ_C (151 MHz, $CDCl_3$); 164.80 (s), 153.20 (s, CO), 150.37 (s, C-1), 146.35 (s, C-8), 145.97 (s, C-5), 141.62 (s, CN), 139.42 (s, CN), 131.18 (s, C-2), 125.40 (s, C-4), 122.17 (s, C-7), 121.78 (s, C-6), 121.11 (s, C-3), 84.18 (s, $\underline{C}(CH_3)_3$), 83.29 (s, $\underline{C}(CH_3)_3$), 83.08 (s, $\underline{C}(CH_3)_3$), 80.06 (s, $\underline{C}(CH_3)_3$), 43.17 (s, C-9 and C-10), 28.09 (s, $C(\underline{C}H_3)_3$), 28.04 (s, $C(\underline{C}H_3)_3$), 27.98 (s, , $C(\underline{C}H_3)_3$), 27.94 (s, $C(\underline{C}H_3)_3$).

HRMS (m/z ESI+): Found ($[M+H]^+$ $C_{37}H_{50}N_6O_{10}$ Requires 739.3588)

Ethyl (4-(2,3-bis(*tert*-butoxycarbonyl)guanidino)phenyl) glycinate, Boc-7 (scheme S3)

N,N'-Boc protected 1-(4-aminophenyl)guanidine (1 eq.) was dissolved in 10 mL of THF. Besides, K_2CO_3 (210 mg, 1.5 mmol, 1.5 eq.) and ethyl bromoacetate (135 µL, 1.2 mmol, 1.2 eq.) were added. The reaction was stirred at room temperature for 16 hours. THF was then removed at low pressure. The crude was dissolved with 50 mL of EtOAc and washed with water (50 mL x 1) and Brine (30 mL x1). The organic phase concentrated under vacuo and purified *via* liquid flash chromatography Eluent: Hexane with a gradient of EtOAc from 0% to 30%). Product **Boc-7** was obtained in an 70% yield.

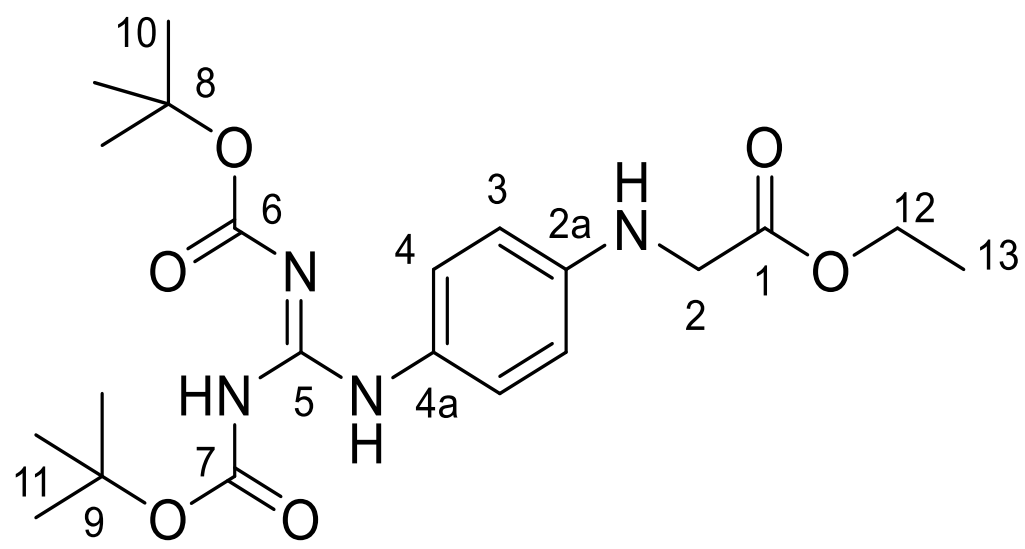

^{1}H NMR (400 MHz, DMSO-d$_6$) δ 11.51 (s, 1H, N<u>H</u>Boc), 9.73 (s, 1H, N<u>H</u>Ar), 7.18 (d, 2H, H_4, J_{3-4} = 8.9 Hz), 6.53 (d, 2H, H_3, J_{3-4} = 8.9 Hz), 6.04 (t, 1H, N<u>H</u>, J_{NH-2} = 5.9 Hz), 4.12 (q, 2H, H_{12}, J_{12-13} = 7.0 Hz), 3.88 (d, 2H, H_2, J_{NH-2} = 5.9 Hz), 1.51 (s, 9H, H_{11}), 1.38 (s, 9H, H_{12}), 1.20 (t, 3H, H_{13}, J_{12-13} = 7.0 Hz).

^{13}C NMR (100 MHz, DMSO-d$_6$) δ 171.3 (C_1), 163.0 (C_5), 153.5 (C_6), 152.3 (C_7), 146.1 (C_{4a}), 125.5 (C_{2a}), 124.8 (C_4), 111.8 (C_3), 83.2 (C_9), 78.4 (C_8), 60.3 (C_{12}), 27.9 (C_{10}), 27.6 (C_{11}), 14.1 (C_{13}).

HRMS (m/z ESI$^+$): m/z found 437.2393 $[M+1]^+$; $C_{21}H_{33}N_4O_6$ required 437.2395

***tert*-butyl N-[(1E)-{[(*tert*-butoxy)carbonyl]imino}({[4-(phenylcarbamoyl)phenyl]amino})methyl]carbamate, Boc-8 (scheme 1)**

Following method **E.2**, this compound was obtained as a (238 mg, 56% yield)

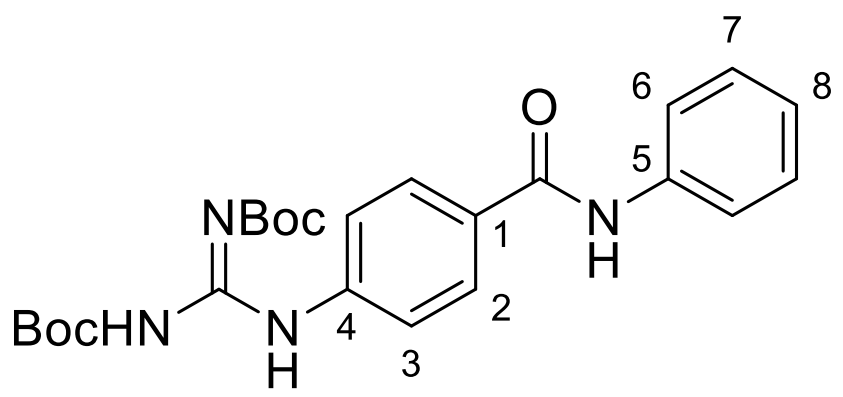

δ_H (400 MHz, $CDCl_3$); 11.65 (s, 1H, NH), 10.55 (s, 1H, NH), 8.05 (s, 1H, NH), 7.83 (d, J = 8.7 Hz, 2H, H-2 and H-2’), 7.75 – 7.67 (m, 4H, H-3, H-3’, H-6 and H-6’), 7.40 (dd, J = 10.8, 5.1 Hz, 2H, H-7 and H-7’), 7.17 (t, J = 7.4 Hz, 1H, H-8), 1.57 (s, 9H, $C(CH_3)_3$), 1.53 (s, 9H, $C(CH_3)_3$).

δ_C (151 MHz, $CDCl_3$); 165.25 (s, CO), 163.27 (s, CN), 153.57 (s, CO), 153.28 (s, CO), 139.96 (s, C-4), 138.17 (s, C-5), 130.85 (s, C-1), 129.06 (s, C-7), 127.99 (s, C-2), 124.38 (s, C-8), 121.97 (s, C-3), 120.12 (s, C-6), 84.18 (s, $\underline{C}(CH_3)_3$), 80.13 (s, $\underline{C}(CH_3)_3$), 28.16 (s, $C(\underline{C}H_3)_3$), 28.08 (s, $C(\underline{C}H_3)_3$).

HRMS (m/z ESI+): Found 454.2293 ($[M+H]^+$ $C_{24}H_{30}N_4O_5$ Requires 454.2216)

Methyl 2-[4-(4-{[(1E)-{[(*tert*-butoxy)carbonyl]amino}({[(*tert*-butoxy)carbonyl]imino})methyl]amino}benzamido)phenyl]acetate, Boc-10 (scheme 1)

Following method **E.2**, this compound was obtained as a white solid (148 mg, 76% yield)

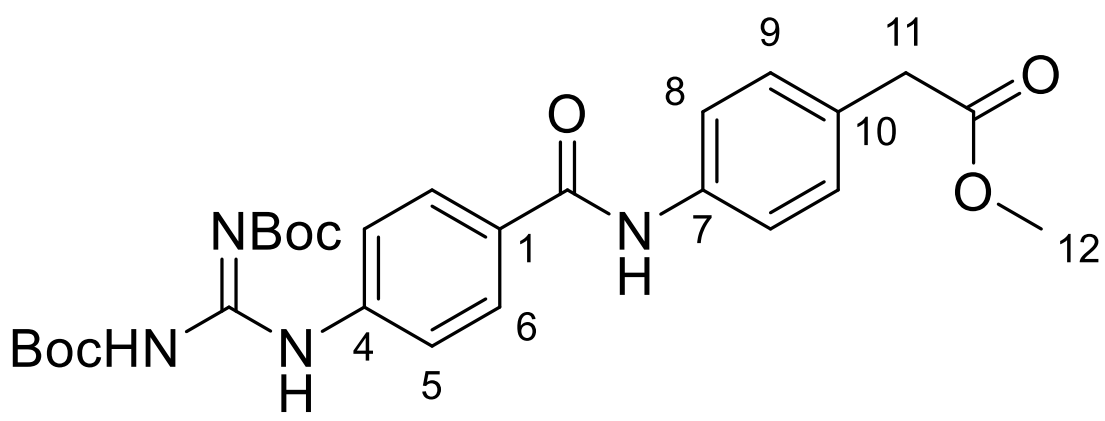

δ_H (400 MHz, *d6*-DMSO); 11.32 (s, 1H, NH), 10.19 (s, 2H, NH), 7.95 (d, J = 8.6 Hz, 2H, H-5 and H-5'), 7.74 (d, J = 8.6 Hz, 2H, H-6 and H-6'), 7.24 (d, J = 8.5 Hz, 2H, H-8 and H-8'), 7.71 (d, J = 8.5 Hz, 2H, H-9 and H-9'), 3.65 (s, 2H, H-11), 3.62 (s, 3H, H-12), 1.52 (s, 9H, $C(C\underline{H}_3)_3$), 1.43 (s, 9H, $C(C\underline{H}_3)_3$).

δ_C (151 MHz, *d6*-DMSO); 172.24 (s, CO), 165.23 (s, CO), 162.80 (s, CN), 152.78 (s, CO), 152.49 (s, CO), 140.31 (s, C-4), 138.38 (s, C-7), 130.98 (s, C-1), 129.99 (s, C-9), 128.82 (s, C-5), 122.10 (s, C-6), 120.82 (s, C-8), 83.94 (s, $\underline{C}(CH_3)_3$), 79.54 (s, $\underline{C}(CH_3)_3$), 52.16 (s, C-12), 39.90 (s, C-11), 28.34 (s, $C(CH_3)_3$), 28.14 (s, $C(CH_3)_3$).

HRMS (m/z ESI+): Found ($[M+H]^+$ $C_{27}H_{34}N_4O_7$ Requires 527.2427)

1,3-di-tert-butyl 2-{[4-(4-{[(1E)-{[(tert-butoxy)carbonyl]amino}({[(tert-butoxy)carbonyl]imino})methyl]amino}benzamido)phenyl]imino}imidazolidine-(1,3)-dicarboxylate, Boc-12 (scheme 2)

Following method E.3, this compound was obtained as a white solid (412 mg, 73% yield)

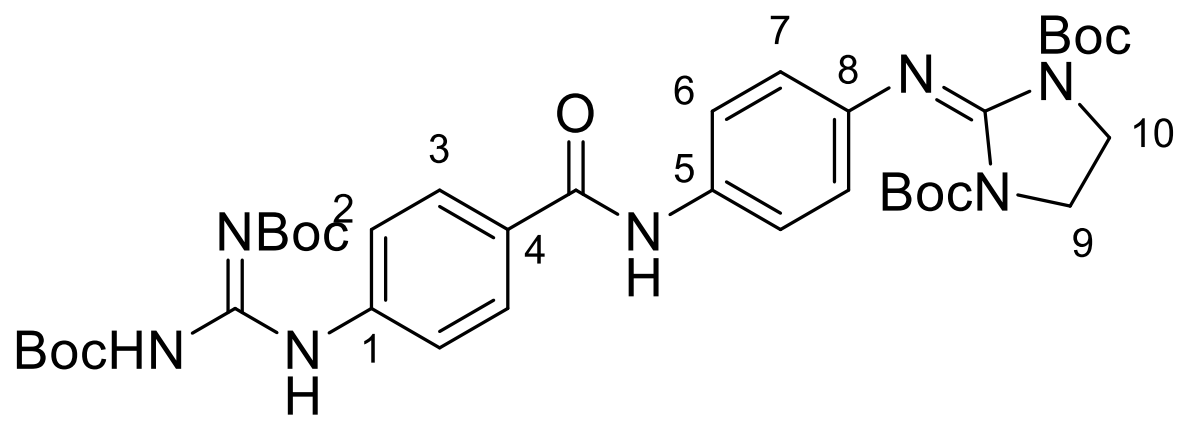

δ_H (600 MHz, *d6*-DMSO); 11.31 (s, 1H, NH), 10.15 (s, 1H, NH), 10.04 (s, 1H, NH), 7.93 (d, J = 8.7 Hz, 2H), 7.71 (d, J = 8.7 Hz, 2H), 7.61 (d, J = 8.8 Hz, 2H), 6.81 (d, J = 8.8 Hz, 2H), 3.73 (s, 4H, H-9 and H-10), 1.50 (s, 9H, $C(CH_3)_3$), 1.41 (s, 9H, $C(CH_3)_3$), 1.26 (s, 18H, 2 x $C(CH_3)_3$).

δ_C (151 MHz, *d6*-DMSO): 164.68 (s, CO), 162.83 (s, CN), 152.79 (s, CO), 152.73 (s, CO), 152.50 (s, CO), 151.55 (s, CO), 150.16 (s), 150.13 (s), 144.91 (s), 140.13 (s), 139.46 (s), 139.38 (s), 134.12 (s), 131.17 (s), 128.74 (s), 122.02 (s), 121.29 (s), 121.20 (s), 83.89 (s), 82.34 (s), 79.48 (s), 43.38 (s), 28.33 (s), 28.12 (s), 27.99 (s), 27.96 (s).

HRMS (m/z ESI+): Found ($[M+H]^+$ $C_{37}H_{51}N_7O_9$ Requires 737.3748)

N-methoxy-N-methyl-4-(2,3-bis(tert-butoxycarbonyl)guanidino)benzamide, Boc-13 (scheme 1)

Compound **Boc-13** was prepared according to ***Method E.1***. (42% yield)

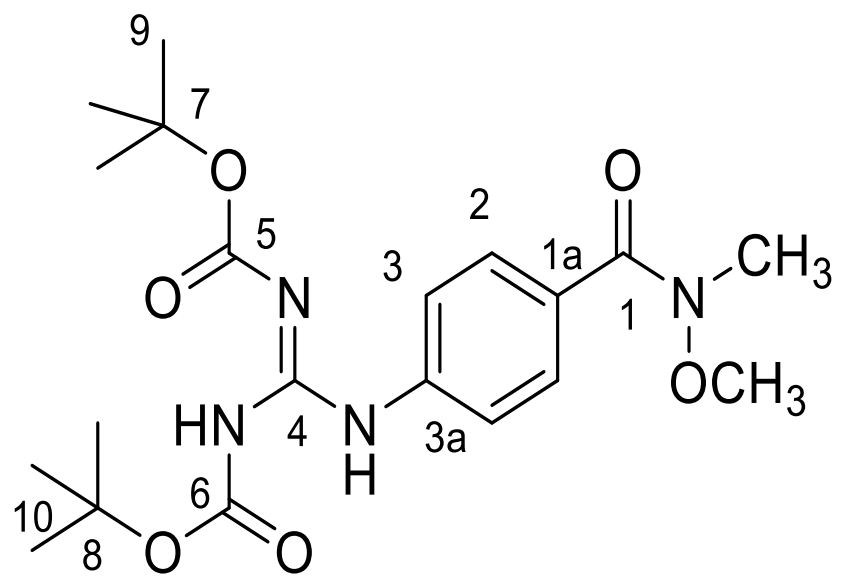

^{1}H NMR (400 MHz, DMSO-d$_6$) δ 11.30 (s, 1H, NHBoc), 10.11 (s, 1H, NHAr), 7.65 (d, 2H, H_3, $J_{2\text{-}3}$ = 9.0 Hz), 7.60 (d, 2H, H_2, $J_{2\text{-}3}$ = 9.0 Hz), 3.56 (s, 3H, OC$\underline{H}_3$), 3.25 (s, 3H, NC$\underline{H}_3$), 1.51 (s, 9H, H_9), 1.42 (s, 9H, H_{10}).

^{13}C NMR (100 MHz, DMSO-d$_6$) δ 168.4 (C_1), 162.3 (C_4), 152.2 (C_5), 152.0 (C_6), 138.8 (C_{3a}), 130.0 (C_{1a}), 128.6 (C_2), 121.3 (C_3), 83.3 (C_8), 79.0 (C_7), 60.7 (O$\underline{C}$H$_3$), 33.3 (N$\underline{C}$H$_3$), 27.9 (C_9), 27.6 (C_{10}).

HRMS (m/z ESI$^+$): m/z found 423.2230 $[M+1]^+$; $C_{20}H_{31}N_4O_6$ required 423.2239

Ethyl 1-methyl-(2,3-bis(*tert*-butoxycarbonyl)guanidino)5-amino-1*H*-indole-2-carboxylate, Boc-15 (scheme 3)

Compound **Boc-15** was prepared according to ***Method E.1***. (41% yield)

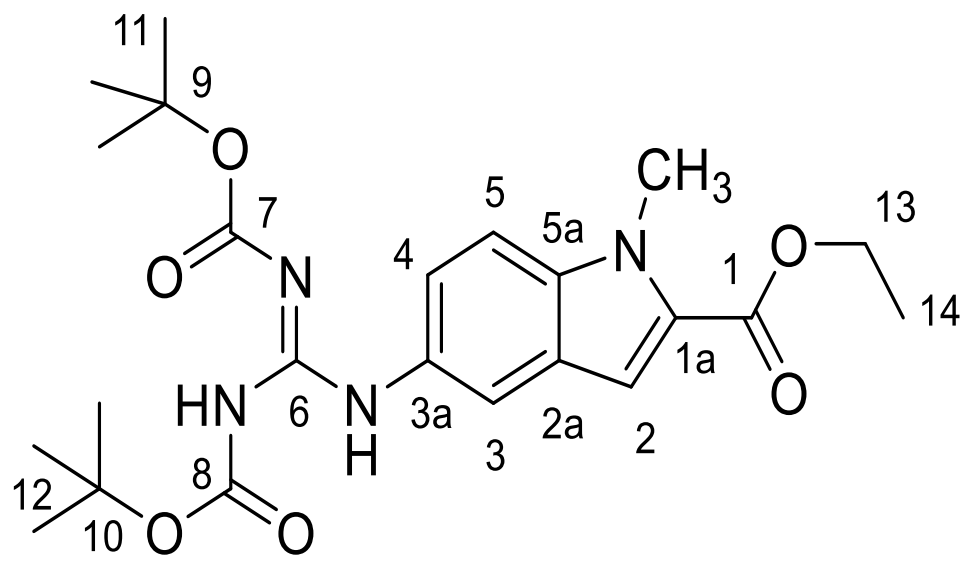

^{1}H NMR (400 MHz, CD_3OD) δ 11.50 (s, 1H, N<u>H</u>Boc), 9.98 (s, 1H, N<u>H</u>Ar), 7.79 (s, 1H, H_3), 7.56 (d, 1H, H_5, $J_{4\text{-}5}$ = 8.7 Hz), 7.40 (d, 1H, H_4, $J_{4\text{-}5}$ = 8.7 Hz), 7.23 (s, 1H, H_2), 4.30 (q, 2H, H_{13}, $J_{13\text{-}14}$ = 7.0 Hz), 4.00 (s, 3H, NC<u>H</u>$_3$), 1.51 (s, 9H, H_{11}), 1.36 (s, 9H, H_{12}), 1.32 (t, 3H, H_{14}, $J_{13\text{-}14}$ = 7.0 Hz).

^{13}C NMR (150 MHz, CD_3OD) δ 163.3 (C_6), 161.7 (C_1), 154.1 (C_7), 152.7 (C_8), 137.6 (C_{2a}), 130.2 (C_{3a}), 128.8 (C_{5a}), 125.4 (C_{1a}), 122.9 (C_2), 117.2 (C_3), 111.5 (C_5), 110.0 (C_2), 83.8 (C_9), 79.0 (C_{10}), 60.9 (C_{13}), 32.2 (N<u>C</u>H$_3$), 28.4 (C_{12}), 28.1 (C_{11}), 14.6 (C_{14}).

HRMS (m/z ESI$^+$): m/z found 461.2399 $[M+1]^+$; $C_{23}H_{33}N_4O_6$ required 461.2395

Ethyl *N*-(2,3-bis(*tert*-butoxycarbonyl)guanidino)-5-amino-1*H*-indole-2-carboxylate, Boc-16 (scheme 3)

Compound **Boc-16** was prepared according to ***Method E.1***. (43% yield)

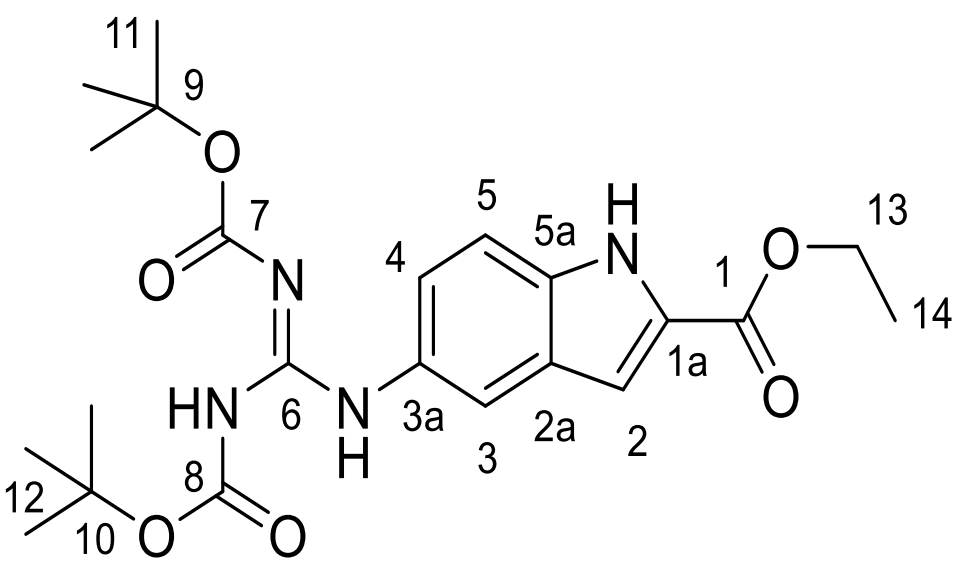

^{1}H NMR (400 MHz, DMSO-d$_6$) δ 11.91 (s, 1H, N<u>H</u>Boc), 11.50 (s, 1H, N<u>H</u>), 9.94 (s, 1H, N<u>H</u>Ar), 7.76 (d, 1H, H_3, J_{3-4} = 1.8 Hz), 7.40 (d, 1H, H_5, J_{4-5} = 8.8 Hz), 7.28 (dd, 1H, H_4, J_{4-5} = 8.8 Hz, J_{3-4} = 1.8 Hz), 7.11 (s, 1H, H_2), 4.32 (q, 2H, H_6, J_{6-7} = 7.2 Hz), 1.50 (s, 9H, H_{11}), 1.35 (s, 9H, H_{12}), 1.32 (t, 3H, H_7, J_{6-7} = 7.2 Hz).

^{13}C NMR (100 MHz, DMSO-d$_6$) δ 163.4 (C_6), 161.6 (C_1), 154.2 (C_7), 152.7 (C_8), 135.7 (C_{3a}), 129.7 (C_{2a}), 128.7 (C_{5a}), 126.9 (C_{1a}), 122.8 (C_4), 117.2 (C_5), 113.0 (C_3), 108.2 (C_2), 83.8 (C_9), 79.0 (C_{10}), 60.9 (C_{13}), 28.4 (C_{11}), 28.1 (C_{12}), 14.7 (C_{14}).

HRMS (m/z ESI$^+$): m/z found 447.2231 $[M+1]^+$; $C_{22}H_{31}N_4O_6$ required 447.2239

2-Azidoethyl *N*-(2,3-bis(*tert*-butoxycarbonyl)guanidino)-5-amino-1*H*-indole-2-carboxylate, Boc-17 (scheme 3)

Compound **Boc-17** was prepared according to ***Method B.2***. (23% yield)

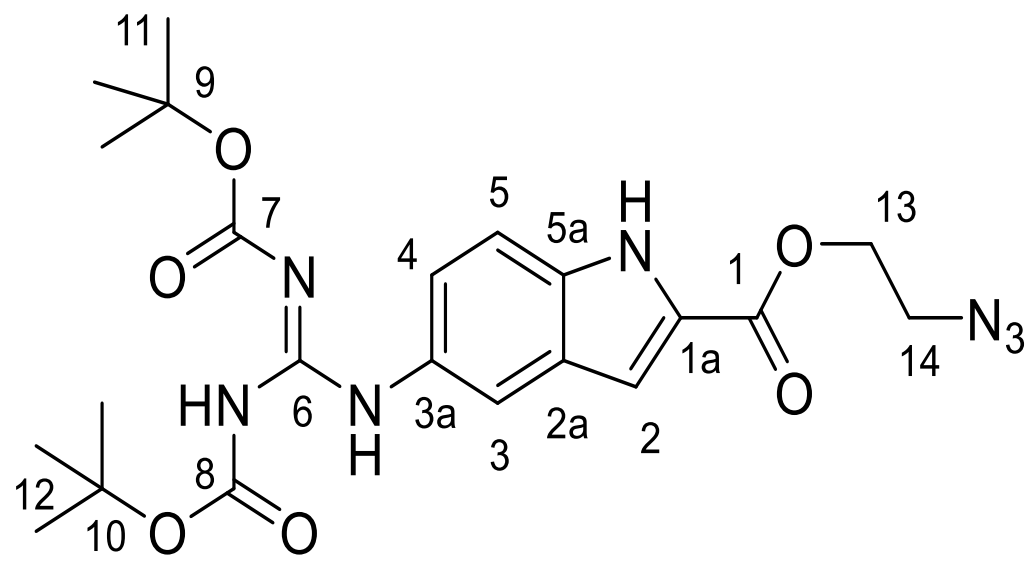

^{1}H NMR (400 MHz, CD$_3$OD) δ 7.79 (d, 1H, H_3, $J_{3\text{-}4}$ = 1.7 Hz), 7.45 (d, 1H, H_5, $J_{4\text{-}5}$ = 8.5 Hz), 7.24 (dd, 1H, H_4, $J_{3\text{-}4}$ = 1.7 Hz, $J_{4\text{-}5}$ = 8.5 Hz), 7.08 (s, 1H, H_2), 3.74 (t, 2H, H_{13}, $J_{13\text{-}14}$ = 5.9 Hz), 3.53 (t, 2H, H_{14}, $J_{13\text{-}14}$ = 5.9 Hz), 1.59 (s, 19H, H_{11}), 1.53 (s, 9H, H_{12}).

^{13}C NMR (100 MHz, CD$_3$OD) δ 162.8 (C_1), 135.0 (C_{5a}), 132.0 (C_{3a}), 127.6 (C_{1a}), 121.1 (C_5), 116.8 (C_3), 112.0 (C_4), 103.2 (C_2), 60.4 (C_{13}), 41.7 (C_{14}), 27.0 (C_{12}, C_{11}).

HRMS (m/z ESI$^+$): m/z found 488.2258 $[M+1]^+$; $C_{22}H_{30}N_7O_6$ required 488.2253

Benzyl *N*-(2,3-bis(*tert*-butoxycarbonyl)guanidino)-5-amino-1*H*-indole-2-carboxylate, Boc-18 (scheme 3)

Compound **Boc-18** was prepared according to ***Method B.2***. (55% yield)

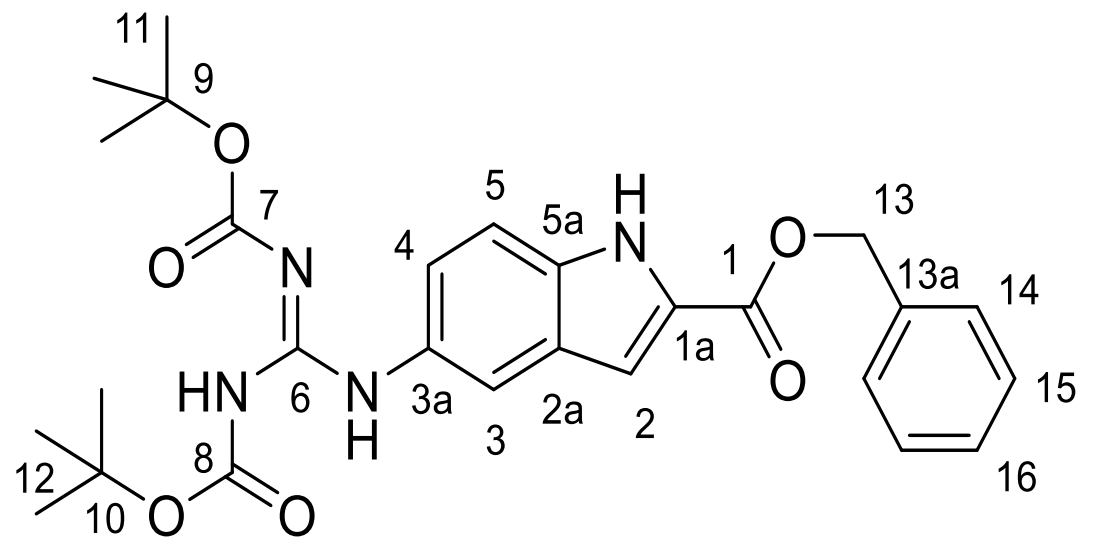

^{1}H NMR (400 MHz, CD_3OD) δ 7.74 (s, 1H, H_3), 7.41 (d, 1H, H_5, J_{4-5} = 9.2 Hz), 7.37 (d, 2H, H_{14}, J_{14-15} = 7.4 Hz), 7.33 (t, 2H, H_{15}, J_{14-15} = J_{15-16} = 7.4 Hz), 7.25 (t, 1H, H_{16}, J_{15-16} = J_{15-16} = 7.4 Hz), 7.20 (dd, 1H, H_4, J_{3-4} = 1.8 Hz, J_{4-5} = 9.2 Hz), 4.58 (s, 2H, H_{13}), 1.56 (s, 9H, H_{11}), 1.41 (s, 9H, H_{12}).

^{13}C NMR (100 MHz, CD_3OD) δ 162.4 (C_1), 158.4 (C_6), 138.8 (C_{13a}), 135.2 (C_{2a}), 131.9 (C_{1a}), 128.1 (C_{15}), 127.6 (C_{5a}), 127.1 (C_{14}), 126.8 (C_{16}), 111.9 (C_5), 103.2 (C_2), 82.5 (C_9), 79.2 (C_{10}), 42.7 (C_{13}), 28.4 (C_{11}), 28.1 (C_{12}).

HRMS (m/z ESI^{+}): m/z found 509.2402 $[M+1]^{+}$; $C_{27}H_{33}N_4O_6$ required 509.2395

N^1-(4-methoxybenzyl)-N^2-(2,3-bis(*tert*-butoxycarbonyl)guanidino)-5-amino-1*H*-indole-2-carboxamide, Boc-19 (scheme 3)

Compound **Boc-19** was prepared according to ***Method B.2***. (87% yield)

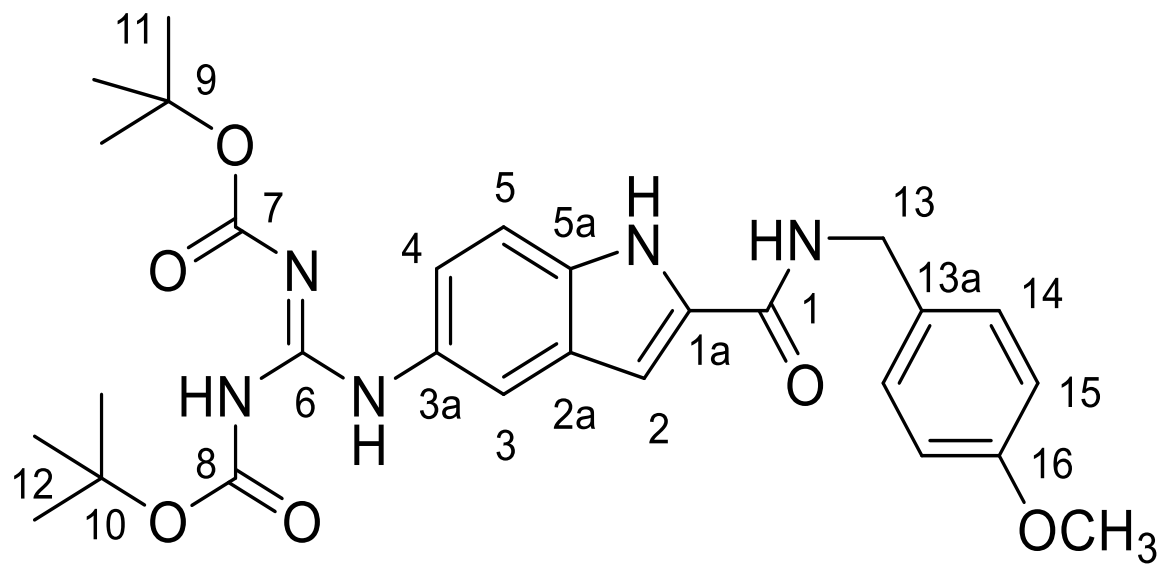

^{1}H NMR (600 MHz, CD$_3$OD) δ 7.73 (d, 1H, H_3, $J_{3\text{-}4}$ = 1.5 Hz), 7.41 (d, 1H, H_5, $J_{4\text{-}5}$ = 8.7 Hz), 7.30 (d, 2H, H_{14}, $J_{14\text{-}15}$ = 8.7 Hz), 7.19 (dd, 1H, H_4, $J_{3\text{-}4}$ = 8.7 Hz, $J_{3\text{-}4}$ = 1.5 Hz), 7.03 (s, 1H, H_2), 6.91 (d, 2H, H_{15}, $J_{14\text{-}15}$ = 8.7 Hz), 4.52 (s, 2H, H_{13}), 3.79 (s, 3H, C$\underline{H}_3$), 1.60 (s, 9H, H_{11}), 1.43 (s, 9H, H_{12}).

^{13}C NMR (150 MHz, CD$_3$OD) δ 162.3 (C_1), 159.1 (C_{16}), 135.1 (C_{3a}), 131.9 (C_{1a}), 130.7 (C_{13a}), 128.6 (C_{14}), 127.5 (C_{2a}), 121.1 (C_4), 117.0 (C_3), 113.5 (C_{15}), 112.0 (C_5), 103.3 (C_2), 83.5 (C_{10}), 79.2 (C_8), 54.3 (O$\underline{C}$H$_3$), 42.2 (C_{13}), 27.0 (C_{12}), 26.9 (C_{11}).

HRMS (m/z ESI$^+$): m/z found 538.2668 $[M+1]^+$; $C_{28}H_{36}N_5O_6$ required 538.2661

***N*[1]-(4-(di(1*H*-indol-3-yl)methyl)phenyl)-*N*[2]-(2,3-bis(*tert*-butoxycarbonyl)guanidino)-5-amino -1*H*-indole-2-carboxamide, Boc-20 (scheme 3)**

Compound **Boc-20** was prepared according to ***Method B.2***. (65% yield)

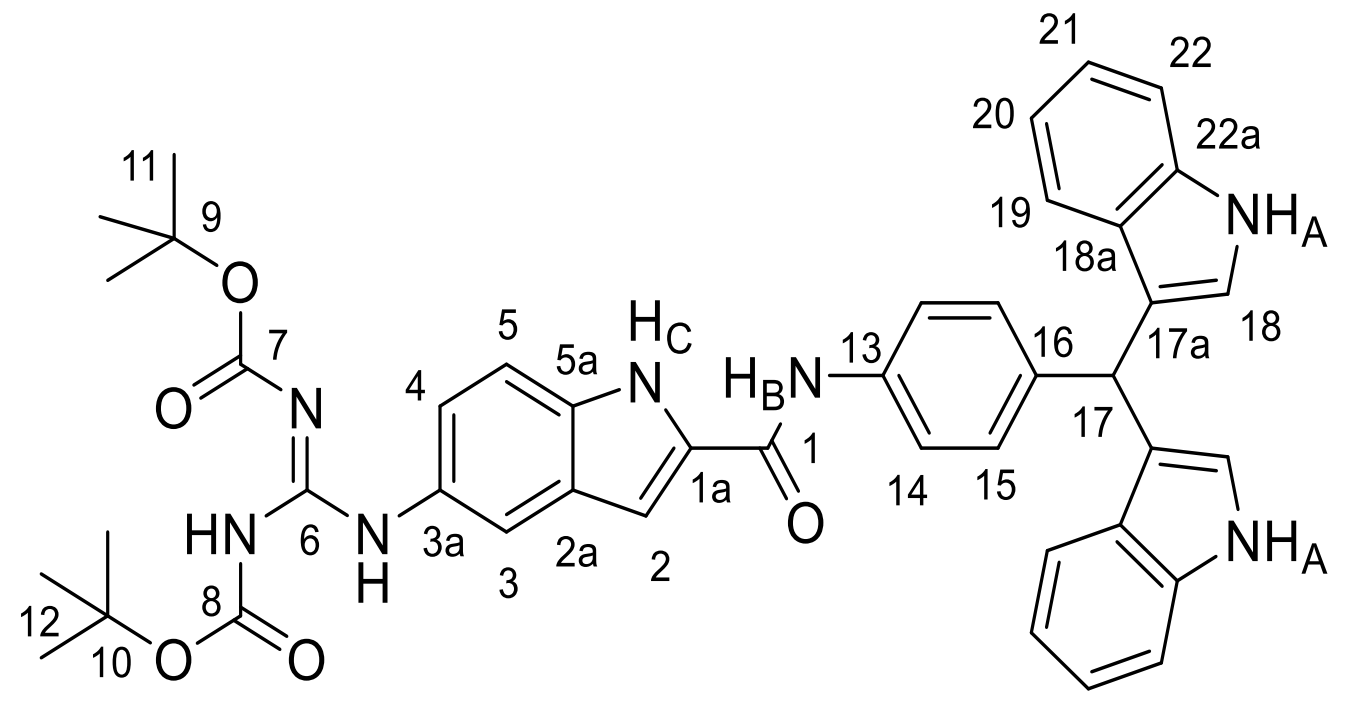

^{1}H NMR (400 MHz, DMSO-d$_6$) δ 11.77 (s, 1H, N<u>H</u>Boc), 11.54 (s, 1H, N-<u>H</u>$_C$), 10.81 (s, 2H, N-<u>H</u>$_A$), 10.15 (s, 1H, N<u>H</u>), 9.99 (s, 1H, N-<u>H</u>$_B$), 7.88 (s, 1H, H_3), 7.70 (d, 2H, H_{14}, J_{14-15} = 8.6 Hz), 7.42 (d, 1H, H_5, J_{4-5} = 8.6 Hz), 7.40 (s, 1H, H_2), 7.38 – 7.32 (m, 4H, H_{15}, H_{22}), 7.30 (d, 2H, H_{19}, J_{19-20} = 8.5 Hz), 7.24 (dd, 1H, H_4, J_{4-5} = 8.7 Hz, J_{3-4} = 1.8 Hz), 7.04 (t, 2H, H_{20}, J_{19-20} = J_{20-21} = 7.3 Hz), 6.87 (t, 2H, H_{21}, J_{20-21} = J_{21-22} = 7.3 Hz), 6.84 (s, 2H, H_{18}), 5.82 (s, 1H, H_{17}), 1.53 (s, 9H, H_{11}), 1.38 (s, 9H, H_{12}).

^{13}C NMR (100 MHz, DMSO-d$_6$) δ 163.4 (C_1), 159.8 (C_6), 154.1 (C_8), 152.8 (C_7), 140.7 (C_{13}), 137.1 (C_{16}, C_{18a}), 135.1 (C_{3a}), 133.0 (C_{5a}), 129.6 (C_{2a}), 128.9 (C_{22}), 127.3 (C_{1a}), 127.1 (C_{17a}), 124.0 (C_{18}), 121.7 (C_4), 121.3 (C_1), 120.4 (C_{3a}), 119.6 (C_{1a}), 118.6 (C_{21}, C_{22a}), 116.8 (C_3), 112.8 (C_5), 111.9 (C_{15}), 104.2 (C_2), 83.8 (C_9), 79.0 (C_{10}), 36.2 (C_{17}), 28.4 (C_9), 28.1 (C_{10}).

HRMS (m/z ESI$^+$): m/z found 738.3391 $[M+1]^+$; $C_{43}H_{14}N_7O_5$ required 738.3399

(E)-N-(4-**(2,3-bis(*****tert*****-butoxycarbonyl)guanidino))but-2-enamide, Boc-21 (scheme 4)**

Compound **Boc-21** was prepared according to ***Method E.1***. (41% yield)

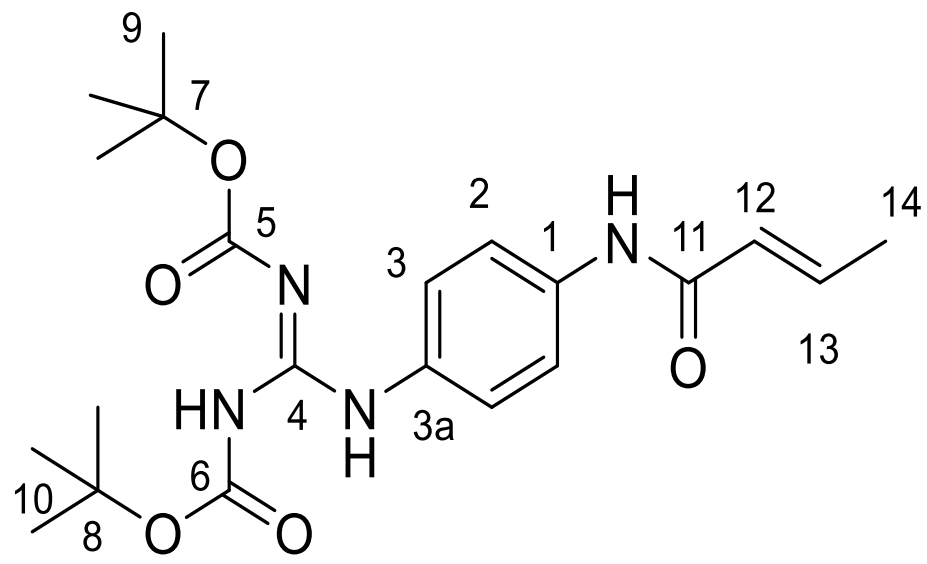

^{1}H NMR (600 MHz, DMSO-d$_6$) δ 11.44 (s, 1H, NHBoc), 9.97 (s, 1H, NHAr), 9.93 (s, 1H, NHC=O), 7.61 (d, 2H, H_3, J_{2-3} = 8.8 Hz), 7.45 (d, 2H, H_2, J_{2-3} = 8.8 Hz), 6.79 (dq, 1H, H_{13}, J_{12-13} = 15.3 Hz, J_{13-14} = 6.9 Hz), 6.11 (dd, 1H, H_{12}, J_{12-14} = 15.3 Hz, J_{13-14} = 1.6Hz), 1.87 (dd, 3H, H_{14}, J_{12-14} = 1.6 Hz, J_{13-14} = 6.9 Hz), 1.51 (s, 9H, H_9), 1.39 (s, 9H, H_{10}).

^{13}C NMR (150 MHz, DMSO-d$_6$) δ 163.3 (C_{11}), 162.7 (C_4), 153.0 (C_5), 152.1 (C_6), 139.8 (C_{13}), 136.3 (C_1), 131.7 (C_{3a}), 125.9 (C_{12}), 123.5 (C_2), 119.3 (C_3), 83.2 (C_7), 78.7 (C_8), 27.9 (C_9), 27.6 (C_{10}), 17.6 (C_{14}).

HRMS (m/z ESI$^+$): m/z found 419.2297 $[M+1]^+$; $C_{21}H_{31}N_4O_5$ required 419.2289

N-(4-(2,3-bis(tert-butoxycarbonyl)guanidino))-3-methylbut-2-enamide, Boc-23 (scheme 4)

Compound **Boc-23** was prepared according to ***Method E.1***. (33% yield)

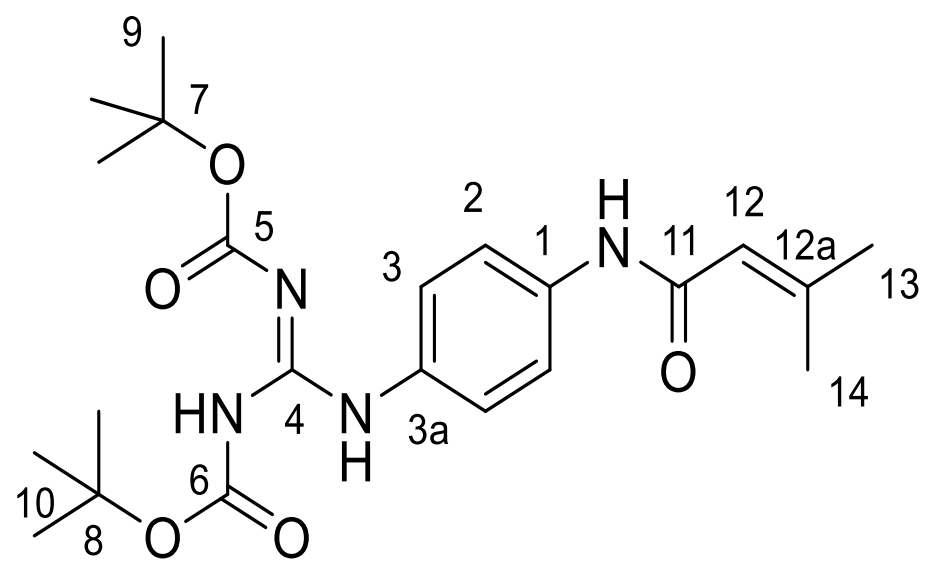

^{1}H NMR (400 MHz, DMSO-d$_6$) δ 11.43 (s, 1H, N<u>H</u>Boc), 9.90 (s, 1H, N<u>H</u>Ar), 9.82 (s, 1H, N<u>H</u>C=O), 7.57 (d, 2H, H_3, J_{2-3} = 8.6 Hz), 7.40 (d, 2H, H_2, J_{2-3} = 8.6 Hz), 5.83 (s, 1H, H_{12}), 2.13 (s, 3H, H_{13}), 1.84 (s, 3H, H_{14}), 1.49 (s, 9H, H_9), 1.38 (s, 9H, H_{10}).

^{13}C NMR (100 MHz, DMSO-d$_6$) δ 165.0 (C_{11}), 165.0 (C_4), 153.5 (C_6), 152.6 (C_5), 151.7 (C_{12a}), 137.1 (C_{3a}), 131.9 (C_1), 123.9 (C_2), 119.6 (C_3, C_{12}), 83.2 (C_7), 78.9 (C_8), 28.4 (C_9), 28.1 (C_{10}), 27.5 (C_{14}), 19.9 (C_{13}).

HRMS (m/z ESI$^+$): m/z found 433.2455 $[M+1]^+$; $C_{22}H_{33}N_4O_5$ required 433.2446

***N*-(4-(2,3-bis(*tert*-butoxycarbonyl)guanidino))cyclohex-1-ene-1-carboxamide, Boc-25 (scheme 4)**

Compound **Boc-25** was prepared according to ***Method E.1***. (24% yield)

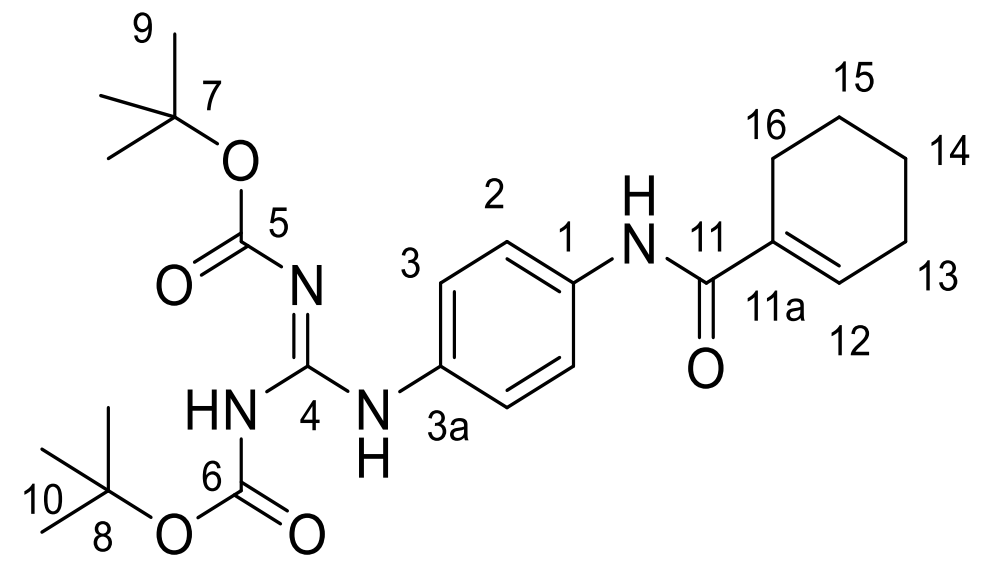

^{1}H NMR (400 MHz, DMSO-d$_6$) δ 11.43 (s, 1H, N<u>H</u>Boc), 10.22 (s, 1H, N<u>H</u>Ar), 9.94 (s, 1H, N<u>H</u>C=O), 7.61 (d, 2H, H_3, $J_{2\text{-}3}$ = 8.3 Hz), 7.41 (d, 2H, H_2, $J_{2\text{-}3}$ = 8.3 Hz), 6.60 (s, 1H, H_{12}), 2.28 – 2.10 (m, 4H, H_{13}, H_{16}), 1.66 – 1.50 (m, 4H, H_{14}, H_{15}), 1.49 (s, 9H, H_9), 1.38 (s, 9H, H_{10}).

^{13}C NMR (100 MHz, DMSO-d$_6$) δ 167.3 (C_7), 163.2 (C_4), 153.5 (C_6), 152.6 (C_5), 136.8 (C_{3a}), 134.5 (C_1), 133.2 (C_{12}), 132.1 (C_{11a}), 123.7 (C_3), 120.6 (C_2), 83.8 (C_8), 79.2 (C_7), 28.4 (C_9), 28.1 (C_{10}), 25.3 (C_{13}), 24.5 (C_{16}), 22.2 (C_{14}), 21.7 (C_{15}).

HRMS (m/z ESI$^+$): m/z found 459.2612 $[M+1]^+$; $C_{24}H_{35}N_4O_5$ required 459.2602

_N_-(4-(2,3-bis(_tert_-butoxycarbonyl)guanidino))-3,4-dihydro-2H-pyran-6-carboxamide, Boc-27 (scheme 4)

Compound **Boc-27** was prepared according to ***Method E.1***. (41% yield)

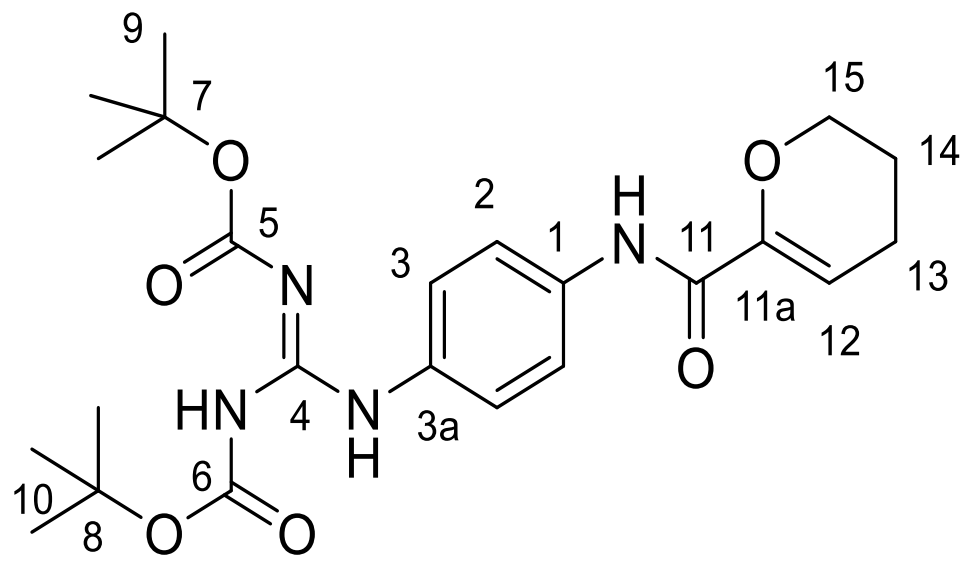

^{1}H NMR (400 MHz, DMSO-d$_6$) δ 11.41 (s, 1H, N<u>H</u>Boc), 9.91 (s, 1H, N<u>H</u>Ar), 9.60 (s, 1H, N<u>H</u>C=O), 7.68 (d, 2H, H_3, $J_{2\text{-}3}$ = 8.4 Hz), 7.42 (d, 2H, H_2, $J_{2\text{-}3}$ = 8.4 Hz), 5.89 (d, 1H, H_2, $J_{7\text{-}8}$ = 3.6 Hz), 4.10 (d, 2H, H_{11}, $J_{11\text{-}12}$ = 4.9 Hz), 2.14 (q, 2H, H_{13}, $J_{12\text{-}13}$ = $J_{13\text{-}14}$ = 4.4 Hz), 1.81 (q, 2H, H_{13}, $J_{13\text{-}14}$ = $J_{14\text{-}15}$ = 5.6 Hz), 1.49 (s, 9H, H_9), 1.38 (s, 9H, H_{10}).

^{13}C NMR (100 MHz, DMSO-d$_6$) δ 163.2 (C_{11}), 160.6 (C_4), 153.4 (C_5), 152.6 (C_6), 146.7 (C_{11a}), 135.8 (C_{3a}), 132.6 (C_1), 123.7 (C_3), 121.1 (C_2), 107.4 (C_{12}), 83.8 (C_7), 79.2 (C_8), 66.7 (C_{15}), 28.4 (C_9), 28.1 (C_{10}), 21.7 (C_{14}), 20.4 (C_{13}).

HRMS (m/z ESI$^+$): m/z found 461.2402 $[M+1]^+$; $C_{23}H_{33}N_4O_6$ required 461.2395

N-(4-(2,3-bis(*tert*-butoxycarbonyl)guanidino))-*N*-methylcinnamamide, Boc-28 (scheme 4)

Compound **Boc-28** was prepared according to ***Method E.1***. (37% yield)

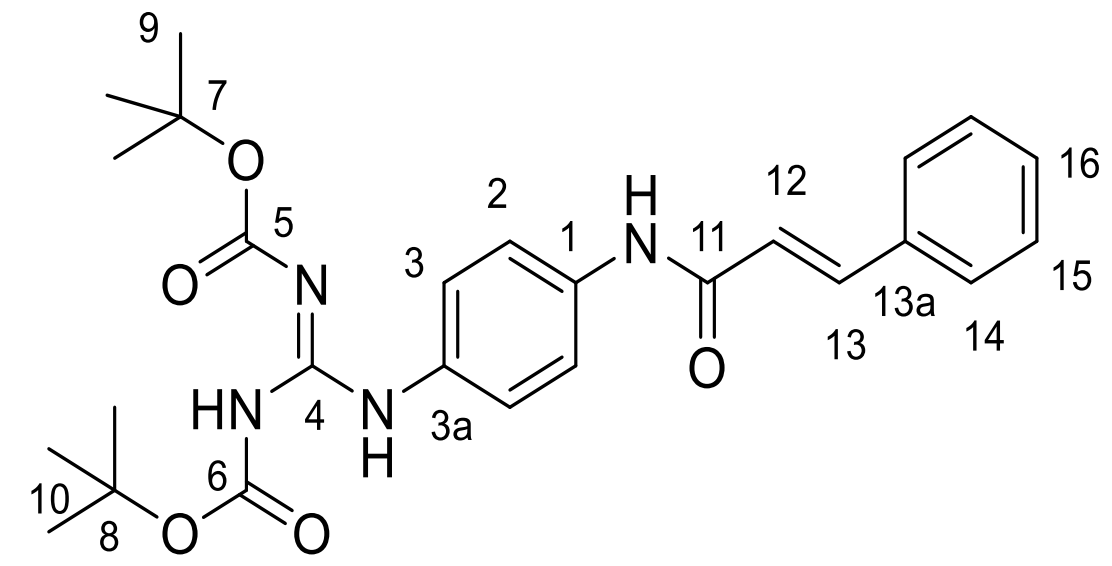

^{1}H NMR (400 MHz, DMSO-d$_6$) δ 11.43 (s, 1H, NHBoc), 10.22 (s, 1H, NHAr), 9.94 (s, 1H, NHC=O), 7.77 – 7.33 (m, 10H, H_2, H_3, H_8, H_{10}, H_{11}, H_{12}), 6.81 (d, 1H, H_9, J_{8-9} = 15.3 Hz), 1.50 (s, 9H, H_9), 1.38 (s, 9H, H_{10}).

^{13}C NMR (100 MHz, DMSO-d$_6$) δ 163.7 (C_{11}), 163.2 (C_4), 153.4 (C_5), 152.6 (C_6), 140.6 (C_{13}), 136.8 (C_1), 135.2 (C_{3a}), 132.4 (C_{13a}), 130.2 (C_{14}), 129.5 (C_2), 128.2 (C_3), 124.0 (C_{12}), 122.7 (C_{16}), 119.8 (C_{15}), 83.3 (C_7), 79.2 (C_8), 28.4 (C_5), 28.1 (C_6).

HRMS (m/z ESI$^+$): m/z found 481.2451 $[M+1]^+$; $C_{26}H_{33}N_4O_5$ required 481.2446

(*E*)-*N*-(4-(2,3-bis(*tert*-butoxycarbonyl)guanidino))-3-(4-methoxyphenyl)acrylamide, Boc-30 (scheme 4)

Compound **Boc-30** was prepared according to ***Method E.1***. (28% yield)

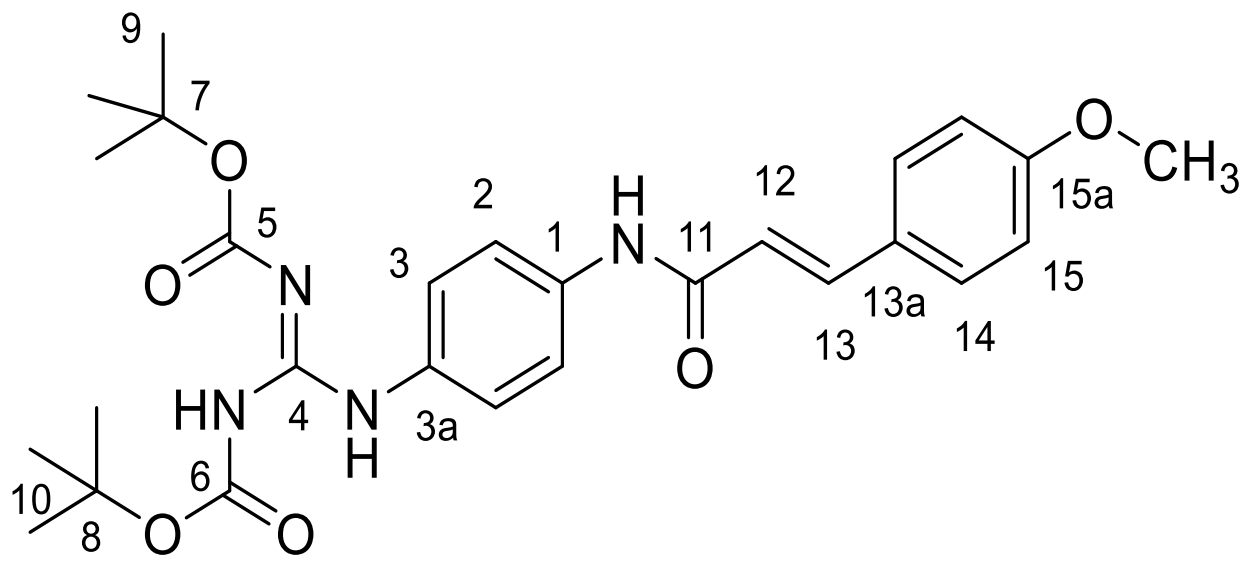

^{1}H NMR (400 MHz, DMSO-d$_6$) δ 11.42 (s, 1H, N<u>H</u>Boc), 10.13 (s, 1H, N<u>H</u>Ar), 9.93 (s, 1H, N<u>H</u>C=O), 7.65 (d, 2H, H_3, J_{2-3} = 8.3 Hz), 7.60 – 7.40 (m, 5H, H_2, H_{12}, H_{14}), 6.99 (d, 2H, H_{15}, J_{14-15} = 8.5 Hz), 6.66 (d, 1H, H_{13}, J_{12-13} = 15.4 Hz), 3.79 (s, 3H, OC<u>H</u>$_3$), 1.50 (s, 9H, H_9), 1.38 (s, 9H, H_{10}).

^{13}C NMR (150 MHz, DMSO-d$_6$) δ 163.7 (C_{11}), 162.7 (C_4), 160.6 (C_{15a}), 153.0 (C_5), 152.1 (C_6), 139.9 (C_{13}), 136.5 (C_{3a}), 131.7 (C_1), 129.3 (C_{15}), 127.3 (C_{13a}), 123.5 (C_{14}), 119.6 (C_{13}), 119.3 (C_{13}), 114.5 (C_2), 83.3 (C_7), 78.7 (C_8), 55.3 (O<u>C</u>H$_3$), 27.9 (C_9), 27.6 (C_{10}).

HRMS (m/z ESI$^+$): m/z found 511.2560 $[M+1]^+$; $C_{27}H_{35}N_4O_6$ required 511.2552

***N*-(4-(2,3-bis(*tert*-butoxycarbonyl)guanidino)-2-ynamide, Boc-32 (scheme 4)**

Compound **Boc-32** was prepared according to ***Method E.1***. (35%. yield)

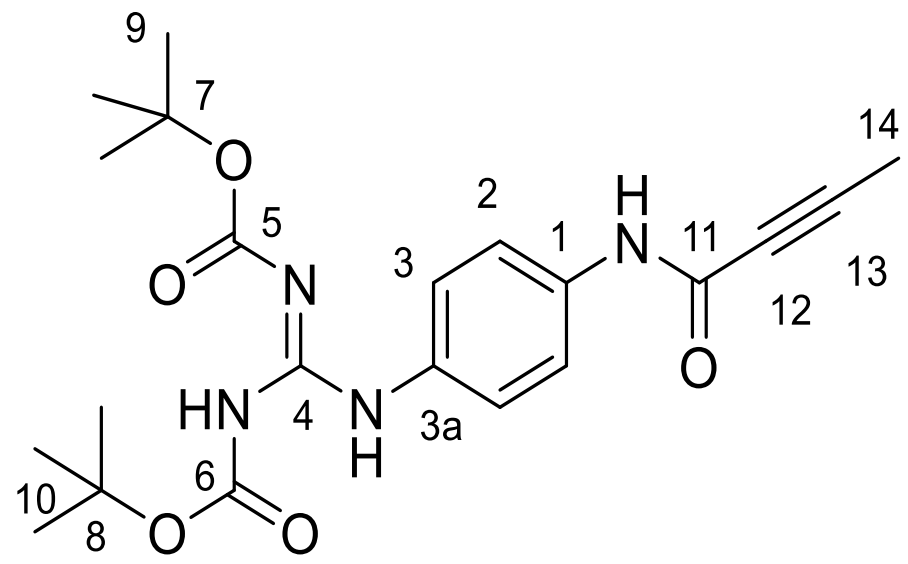

^{1}H NMR (400 MHz, DMSO-d_6) δ 11.40 (s, 1H, N<u>H</u>Boc), 10.60 (s, 1H, N<u>H</u>Ar), 9.91 (s, 1H, N<u>H</u>C=O), 7.53 (d, 2H, H_3, J_{2-3} = 8.6 Hz), 7.43 (d, 2H, H_2, J_{2-3} = 8.6 Hz), 2.02 (s, 3H, H_{14}), 1.49 (s, 9H, H_9), 1.37 (s, 9H, H_{10}).

^{13}C NMR (100 MHz, DMSO-d_6) δ 163.1 (C_4), 153.4 (C_5), 152.6 (C_6), 150.8 (C_{11}), 136.0 (C_{3a}), 132.7 (C_1), 123.9 (C_2), 120.1 (C_3), 84.6 (C_{13}), 83.8 (C_7), 79.2 (C_8), 76.3 (C_{12}), 28.3 (C_9), 28.1 (C_{10}), 3.7 (C_{14}).

HRMS (m/z ESI$^+$): m/z found 417.2138 $[M+1]^+$; $C_{21}H_{29}N_4O_5$ required 417.2133

4-(2,3-Bis(*tert*-butoxycarbonyl)guanidino)-4H-chromen-4-one, Boc-33 (scheme S5)

Compound **Boc-33** was prepared according to ***Method E.1***. (48% yield)

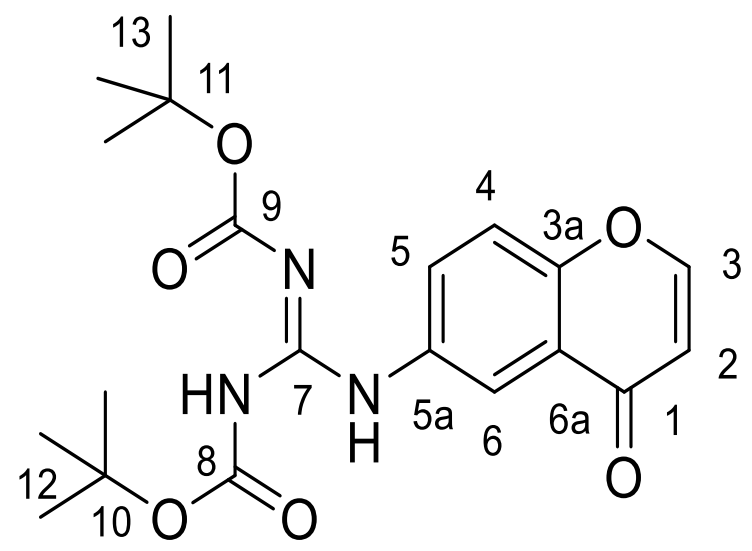

^{1}H NMR (400 MHz, DMSO-d$_6$) δ 11.30 (s, 1H, N<u>H</u>Boc), 10.09 (s, 1H, N<u>H</u>Ar), 8.28 (d, 1H, H_3, $J_{2\text{-}3}$ = 6.3 Hz), 8.11 (d, 1H, H_6, $J_{5\text{-}6}$ = 2.3 Hz), 7.93 (dd, 1H, H_5, $J_{4\text{-}5}$ = 9.0 Hz, $J_{5\text{-}6}$ = 2.3 Hz), 7.65 (d, 1H, H_4, $J_{4\text{-}5}$ = 9.0 Hz), 6.32 (d, 1H, H_2, $J_{2\text{-}3}$ = 6.3 Hz), 1.50 (s, 9H, H_{12}), 1.38 (s, 9H, H_{13}).

^{13}C NMR (100 MHz, DMSO-d$_6$) δ 176.5 (C_1), 162.8 (C_7), 157.4 (C_3), 153.5 (C_{3a}), 153.2 (C_9), 152.3 (C_8), 134.7 (C_{5a}), 130.1 (C_5), 124.7 (C_{1a}), 119.4 (C_4), 118.4 (C_6), 112.3 (C_2), 83.7 (C_{10}), 79.4 (C_{11}), 28.3 (C_{12}), 28.1 (C_{13}).

HRMS (m/z ESI$^+$): m/z found 404.1825 $[M+1]^+$; $C_{20}H_{26}N_3O_6$ required 404.1817

4-(2,3-Bis(*tert*-butoxycarbonyl)guanidino)-2H-chromen-2-one, Boc-34 (scheme S5)

Compound **Boc-34** was prepared according to ***Method E.1***. (45% yield)

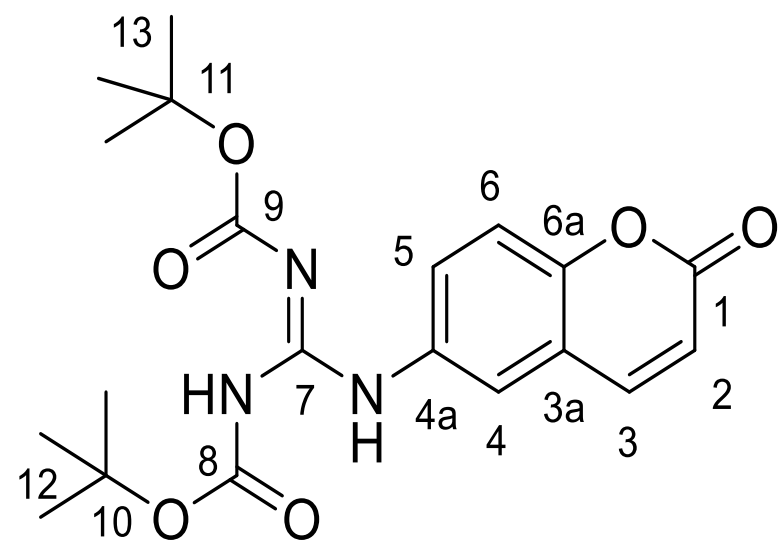

^{1}H NMR (400 MHz, DMSO-d$_6$) δ 11.39 (s, 1H, N<u>H</u>Boc), 10.05 (s, 1H, N<u>H</u>Ar), 8.03 (d, 1H, H_3, $J_{2\text{-}3}$ = 9.6 Hz), 7.86 – 7.74 (m, 2H, H_5, H_6), 7.39 (d, 1H, H_4, $J_{4\text{-}5}$ = 8.8 Hz), 6.49 (d, 1H, H_2, $J_{2\text{-}3}$ = 9.6 Hz), 1.50 (s, 9H, H_{12}), 1.37 (s, 9H, H_{13}).

^{13}C NMR (100 MHz, DMSO-d$_6$) δ 160.4 (C_1), 154.1 (C_7), 151.2 (C_8), 150.0 (C_9), 144.6 (C_2), 134.7 (C_{3a}), 124.3 (C_6), 119.2 (C_5), 118.8 (C_2), 117.1 (C_6), 82.3 (C_{10}), 28.2 (C_{12}, C_{13}).

HRMS (m/z ESI$^+$): m/z found 404.1827 [M+1]$^+$; $C_{20}H_{26}N_3O_6$ required 404.1817

***N*^{6}-(2,3-bis(*tert*-butoxycarbonyl)guanidino)-*N*^{2}-(4-(2,3-bis(*tert*-butoxycarbonyl)guanidino) benzothiazole-2,6-diamine, Boc-39 (scheme S6)**

Compound **Boc-39** was prepared according to ***Method E.1***. (18% yield)

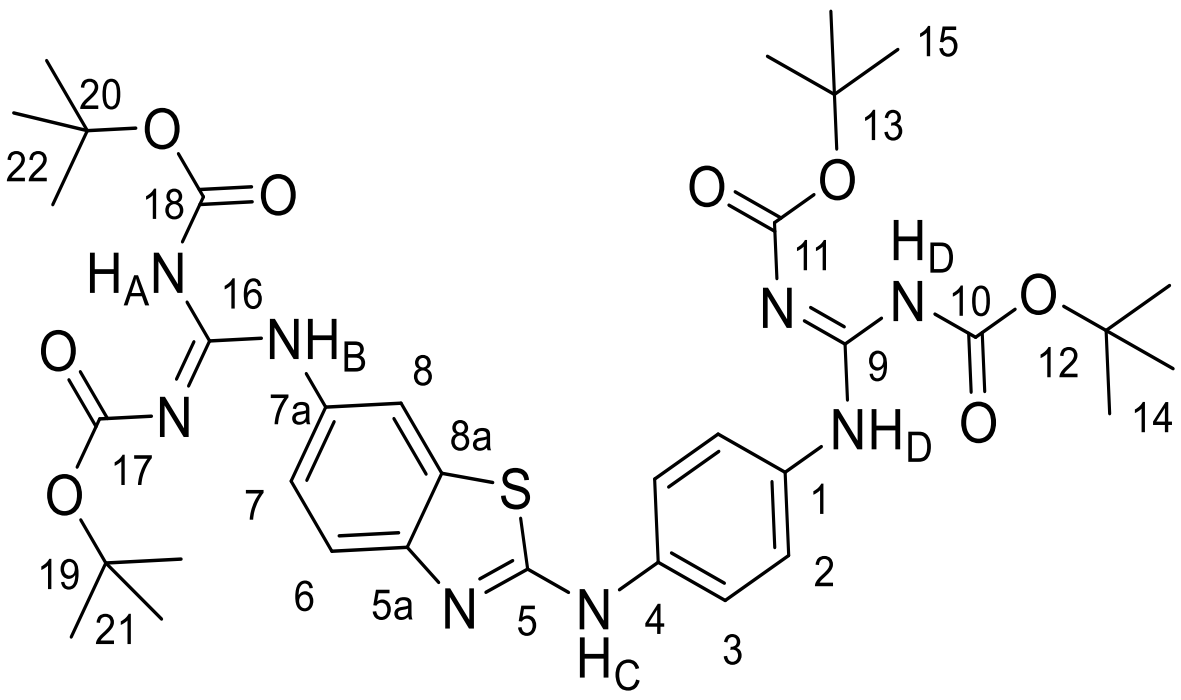

^{1}H NMR (400 MHz, CD_3OD) δ 11.41 (s, 1H, N$\underline{H}_A$), 11.06 (s, 1H, N$\underline{H}_E$), 10.27 (s, 1H, N$\underline{H}_B$), 10.14 (s, 1H, N$\underline{H}_D$), 9.33 (s, 1H, N$\underline{H}_C$), 8.36 (d, 1H, H_8, $J_{7\text{-}8}$ = 1.4 Hz), 8.21 (d, 2H, H_3, $J_{2\text{-}3}$ = 9.1 Hz), 8.03 (d, 1H, H_6, $J_{6\text{-}7}$ = 8.7 Hz), 7.84 (d, 2H, H_2, $J_{2\text{-}3}$ = 9.1 Hz), 7.63 (dd, 1H, H_7, $J_{6\text{-}7}$ = 8.7 Hz, $J_{7\text{-}8}$ = 1.4 Hz), 1.51 (s, 9H, H_{21}), 1.49 (s, 9H, H_{22}), 1.41 (s, 9H, H_{14}), 1.38 (s, 9H, H_{15}).

^{13}C NMR (400 MHz, CD_3OD) δ 163.0 (C_{16}), 162.1 (C_9), 156.3 (C_5), 153.5 (C_{18}), 152.5 (C_{7a}), 152.2 (C_{17}), 151.6 (C_{11}), 150.8 (C_{10}), 144.0 (C_4), 143.3 (C_1), 134.9 (C_{8a}), 134.3 (C_{5a}), 125.1 (C_3), 123.2 (C_6), 122.8 (C_7), 121.9 (C_2), 116.7 (C_8), 83.8 (C_{13}), 83.6 (C_{19}), 79.8 (C_{12}), 79.4 (C_{20}), 28.3 (C_{14}, C_{22}), 28.1 (C_{15}, C_{22}).

HRMS (m/z ESI$^+$): m/z found 741.3398 $[M+1]^+$; $C_{35}H_{49}N_8O_8S$ required 741.3389

N-(2,3-bis(*tert*-butoxycarbonyl)guanidino)-2-benzothiazolamine, Boc-40 (scheme S6)

Compound **Boc-40** was prepared according to **Method *E.1***. (31% yield)

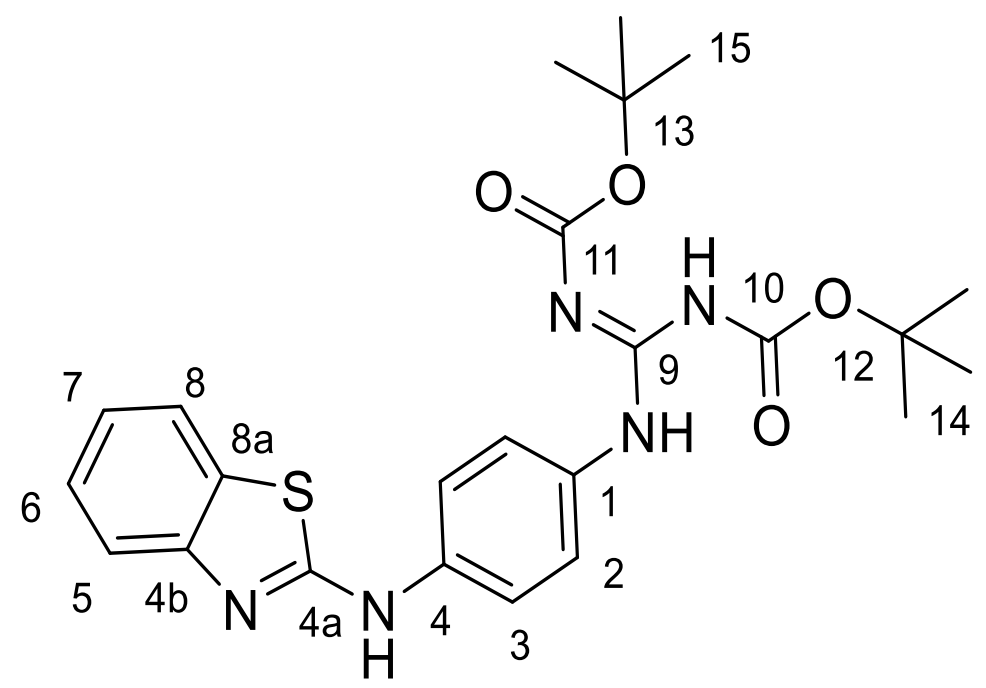

^{1}H NMR (400 MHz, DMSO-d$_6$) δ 11.42 (s, 1H, NHBoc), 9.96 (s, 1H, NHBoc), 9.75 (s, 1H, NH), 7.51 (d, 2H, H_2, J_{2-3} = 8.7 Hz), 7.50 – 7.40 (m, 3H, H_3, H_8), 7.38 – 7.30 (m, 2H, H_5, H_7), 7.30 (t, 1H, H_6, J_{5-6} = J_{6-7} = 7.7 Hz), 1.51 (s, 9H, H_{14}), 1.40 (s, 9H, H_{15}).

^{13}C NMR (100 MHz, DMSO-d$_6$) δ 181.6 (C_{4a}), 163.2 (C_9), 153.3 (C_{11}), 152.6 (C_{10}), 136.6 (C_{8a}), 136.3 (C_{4b}), 133.5 (C_1), 132.3 (C_4), 129.6 (C_3), 129.3 (C_8), 129.0 (C_6), 127.6 (C_7), 124.2 (C_8), 123.5 (C_2), 83.8 (C_{13}), 79.3 (C_{12}), 28.4 (C_{15}), 28.1 (C_{14}).

HRMS (m/z ESI$^+$): m/z found 484.2022 [M+1]$^+$; $C_{24}H_{30}N_5O_4S$ required 484.2014

(Z)-5-(2,3-bis(*tert*-butoxycarbonyl)guanidino)-1*H*-indole-2-carboxylic acid, 52 (scheme 3)

Compound **52** was prepared according to ***Method E.1***. (42% yield)

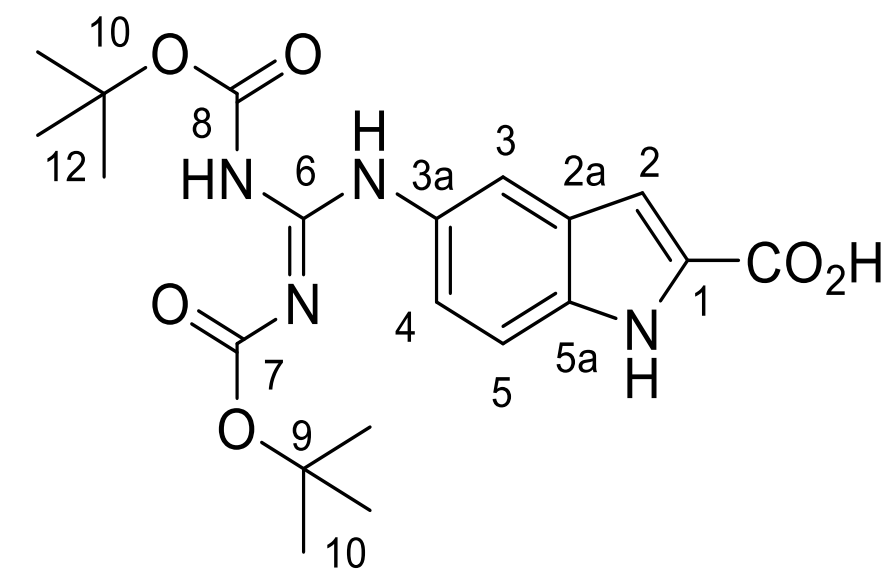

^{1}H NMR (400 MHz, DMSO-d$_6$) δ 12.96 (s, 1H, $CO_2\underline{H}$), 11.77 (s, 1H, N$\underline{H}$Boc), 11.51 (s, 1H, N$\underline{H}$), 9.94 (s, 1H, N$\underline{H}$Ar), 7.77 (s, 1H, H_2), 7.38 (d, 1H, H_5, $J_{4\text{-}5}$ = 8.8 Hz), 7.24 (dd, 2H, H_4, $J_{4\text{-}5}$ = 8.8 Hz, $J_{3\text{-}4}$ = 1.8 Hz), 7.04 (d, 1H, H_3, $J_{3\text{-}4}$ = 1.8 Hz), 1.52 (s, 9H, H_{11}), 1.38 (s, 9H, H_{12}).

^{13}C NMR (100 MHz, DMSO-d$_6$) δ 162.8 ($\underline{C}O_2H$, C_6), 153.8 (C_8), 152.3 (C_7), 135.1 (C_{2a}), 129.6 (C_1), 129.0 (C_{5a}), 126.6 (C_{3a}), 121.9 (C_4), 116.7 (C_2), 112.5 (C_5), 107.2 (C_3), 83.4 (C_9), 78.6 (C_{10}), 27.8 (C_{12}), 27.7 (C_{11}).

HRMS (m/z ESI$^-$): m/z found 417.1767 $[M-1]^-$; $C_{20}H_{25}N_4O_6$ required 417.1779

N^1-4-(2,3-bis(*tert*-butoxycarbonyl)guanidino)-N^2-methyldianiline, 55 (scheme 1)

Compound **60** was prepared according to ***Method E.1***. (35% yield)

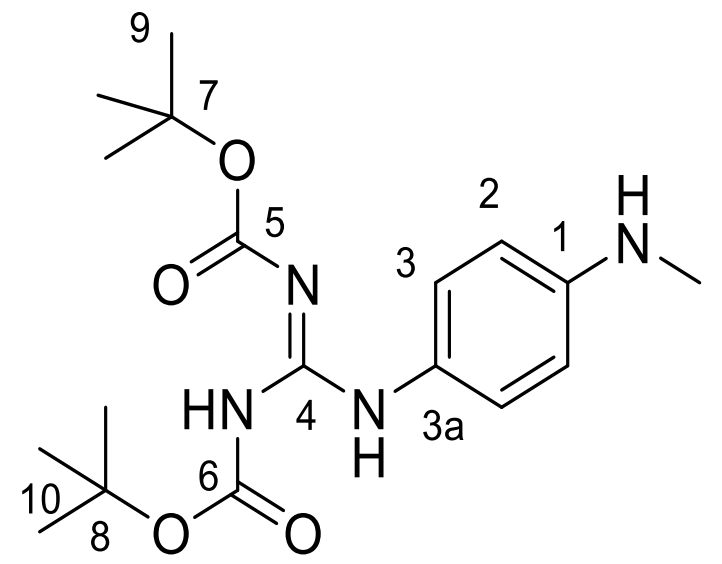

^{1}H NMR (400 MHz, DMSO-d$_6$) δ 11.50 (s, 1H, N<u>H</u>Boc), 9.70 (s, 1H, N<u>H</u>Ar), 7.16 (d, 2H, H_3, $J_{2\text{-}3}$ = 8.9 Hz), 6.49 (d, 2H, H_2, $J_{2\text{-}3}$ = 8.9 Hz), 5.63 (q, 3H, N<u>H</u>CH_3, $J_{NH\text{-}CH3}$ = 5.0 Hz), 2.64 (d, 3H, NHC<u>H</u>$_3$, $J_{NH\text{-}CH3}$ = 5.0 Hz), 1.49 (s, 9H, H_9), 1.36 (s, 9H, H_{10}).

^{13}C NMR (100 MHz, DMSO-d$_6$) δ 163.5 (C_4), 153.9 (C_6), 152.8 (C_5), 148.3 (C_{3a}), 125.3(C_3), 125.2 (C_{3a}), 111.7 (C_2), 83.7 (C_7), 78.8 (C_8), 30.3 (NH<u>C</u>H_3), 28.4 (C_9), 28.1 (C_{10}).

HRMS (m/z ESI$^+$): m/z found 365.2198 $[M+1]^+$; $C_{18}H_{29}N_4O_6$ required 365.2184

(*E*)-*N*-(4-(2,3-bis(*tert*-butoxycarbonyl)guanidino))-*N*-methylbut-2-enamide, 56 (scheme 4)

Compound **56** was prepared according to ***Method A.1***. (quant. yield)

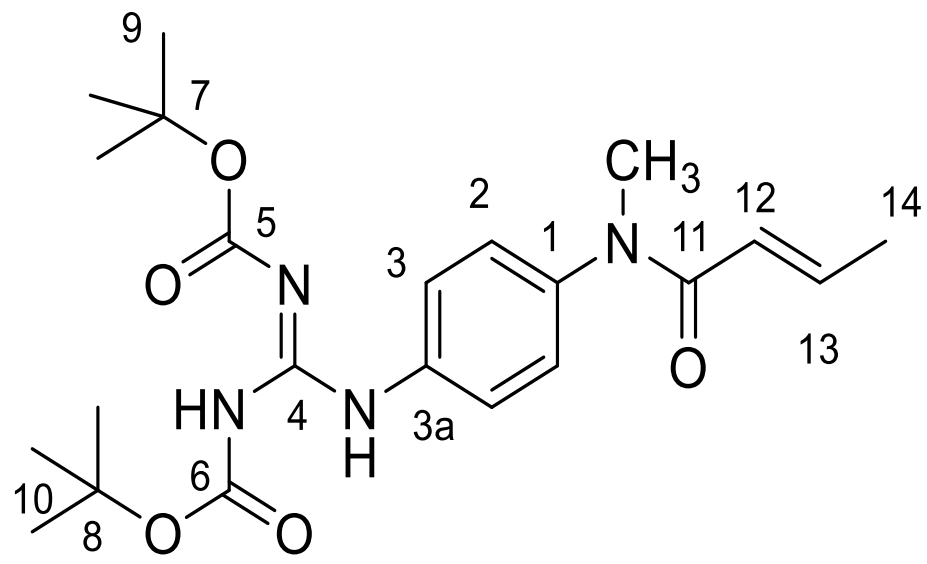

^{1}H NMR (600 MHz, DMSO-d$_6$) δ 11.36 (s, 1H, N<u>H</u>Boc), 10.05 (s, 1H, N<u>H</u>Ar), 7.63 (d, 2H, H_3, $J_{2\text{-}3}$ = 8.7 Hz), 7.25 (d, 2H, H_2, $J_{2\text{-}3}$ = 8.7 Hz), 6.71 (dq, 1H, H_{13}, $J_{12\text{-}14}$ = 15.0 Hz, $J_{13\text{-}14}$ = 6.9 Hz), 5.76 (s, 1H, H_{12}), 3.20 (s, 3H, NC<u>H</u>$_3$), 1.71 (dd, 3H, H_{14}, $J_{12\text{-}14}$ = 1.4 Hz, $J_{13\text{-}14}$ = 6.9 Hz), 1.45 (brd, 18H, H_9, H_{10}).

^{13}C NMR (150 MHz, DMSO-d$_6$) δ 164.5 (C_{11}), 152.5 (C_4), 140.4 (C_1), 139.7 (C_{13}), 135.3 (C_{3a}), 127.5 (C_3), 123.2 (C_2), 122.6 (C_{12}), 36.8 (N<u>C</u>H$_3$), 27.8 (C_9, C_{10}), 17.6 (C_{14}).

HRMS (m/z ESI$^+$): m/z found 433.2434 [M+1]$^+$; $C_{22}H_{33}N_4O_5$ required 433.2446

_N_-(4-(2,3-bis(_tert_-butoxycarbonyl)guanidino))-_N_,3-dimethylbut-2-enamide, 57 (scheme 4)

Compound **57** was prepared according to ***Method A.1***. (quant. yield)

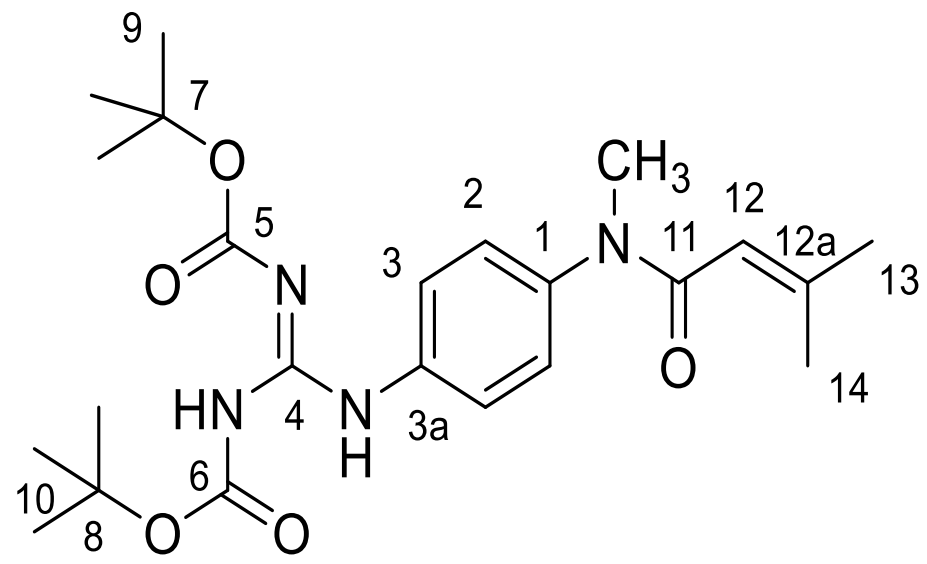

^{1}H NMR (600 MHz, DMSO-d$_6$) δ 11.36 (s, 1H, N<u>H</u>Boc), 10.03 (s, 1H, N<u>H</u>Ar), 7.60 (d, 2H, H_3, $J_{2\text{-}3}$ = 8.6 Hz), 7.22 (d, 2H, H_2, $J_{2\text{-}3}$ = 8.6 Hz), 5.47 (s, 1H, H_{12}), 3.17 (s, 3H, NC<u>H</u>$_3$), 2.00 (s, 3H, H_{13}), 1.65 (s, 3H, H_{14}), 1.51 (s, 9H, H_9), 1.41 (s, 9H, H_{10}).

^{13}C NMR (150 MHz, DMSO-d$_6$) δ 165.8 (C_{11}), 152.5 (C_4), 152.0 (C_6), 148.7 (C_5), 140.3 (C_1), 135.3 (C_{3a}), 127.2 (C_3), 123.2 (C_2), 123.0 (C_{12a}), 117.6 (C_{12}), 83.2 (C_7), 78.9 (C_8), 36.5 (N<u>C</u>H$_3$), 27.9 (C_9), 27.6 (C_{10}), 26.7 (C_{14}), 19.7 (C_{13}).

HRMS (m/z ESI$^+$): m/z found 447.2611 [M+1]$^+$; $C_{25}H_{37}N_4O_5$ required 447.2602

N-(4-(2,3-bis(tert-butoxycarbonyl)guanidino))-N-methylcyclohex-1-ene-1-carboxamide, 58 (scheme 4)

Compound **58** was prepared according to ***Method A.1***. (quant. yield)

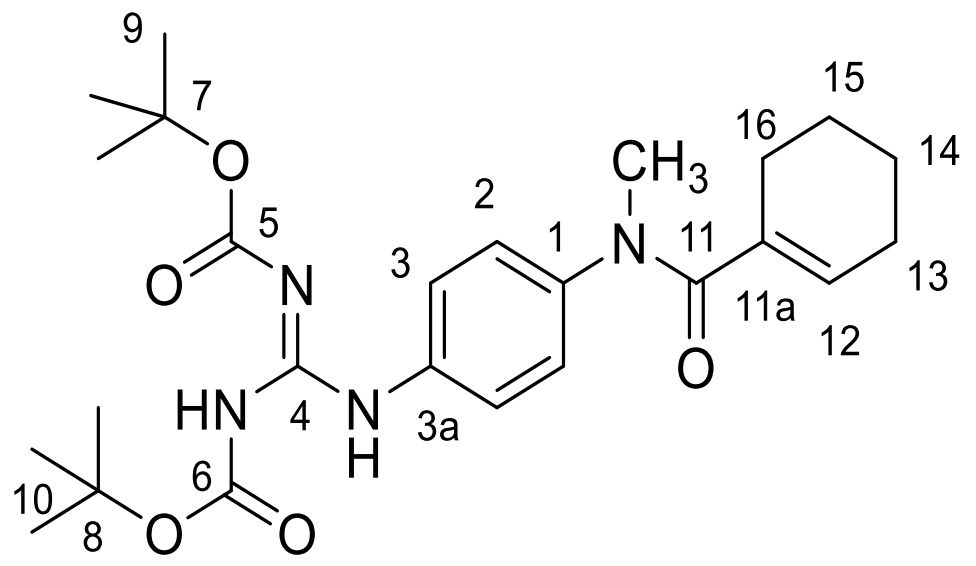

^{1}H NMR (400 MHz, CD_2Cl_2) δ 11.64 (s, 1H, N<u>H</u>Boc), 10.28 (s, 1H, N<u>H</u>Ar), 7.55 (d, 2H, H_3, $J_{2\text{-}3}$ = 8.6 Hz), 7.12 (d, 2H, H_2, $J_{2\text{-}3}$ = 8.6 Hz), 5.78 (s, 1H, H_{12}), 3.09 (s, 3H, NC<u>H</u>$_3$), 2.00 (s, 2H, H_{13}), 1.91 (s, 2H, H_{16}), 1.52 - 1.41 (m, 22H, H_9, H_{10}, H_{14}, H_{15}).

^{13}C NMR (100 MHz, CD_2Cl_2) δ 172.1 (C_{11}), 153.7 (C_4), 144.3 (C_{11a}), 141.8 (C_1), 134.6 (C_{3a}), 131.9 (C_{12}), 126.7 (C_3), 122.9 (C_2), 83.3 (C_7), 78.9 (C_8), 37.4 (N<u>C</u>H$_3$), 27.8 (C_9, C_{10}), 25.9 (C_{13}), 24.8 (C_{16}), 22.1 (C_{14}), 21.5 (C_{15}).

HRMS (m/z ESI$^+$): m/z found 473.2748 $[M+1]^+$; $C_{25}H_{37}N_4O_5$ required 473.2759

_N_-(4-(2,3-bis(_tert_-butoxycarbonyl)guanidino))-_N_-methylcinnamamide, 59 (scheme 4)

Compound **59** was prepared according to ***Method A.1***. (quant. yield)

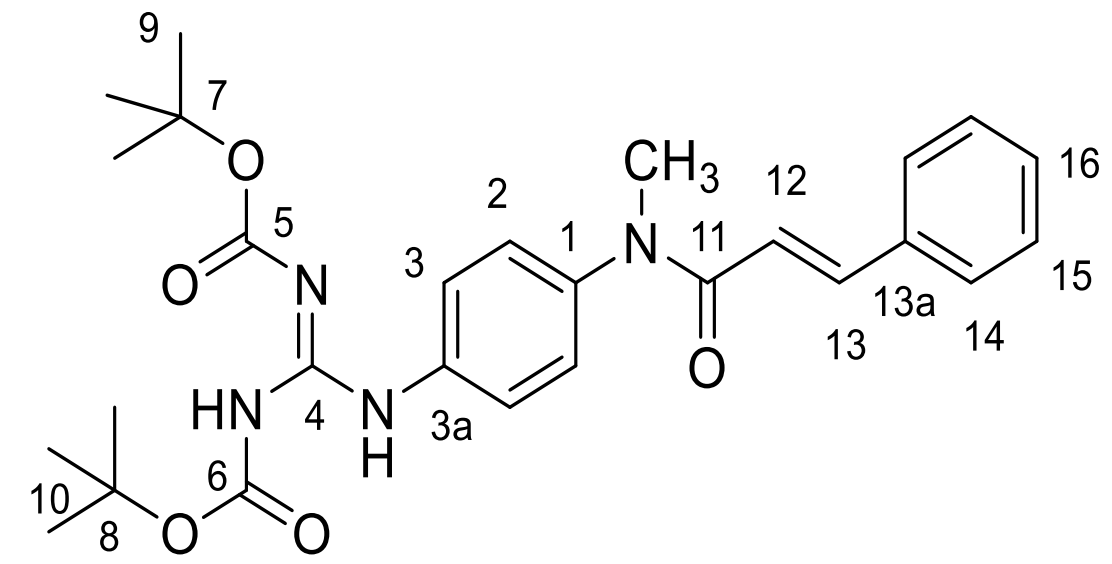

^{1}H NMR (600 MHz, DMSO-d$_6$) δ 11.37 (s, 1H, N<u>H</u>Boc), 10.08 (s, 1H, N<u>H</u>Ar), 7.67 (d, 2H, H_3, J_{2-3} = 8.6 Hz), 7.52 (d, 1H, H_{13}, J_{12-13} = 15.7 Hz), 7.46 – 7.26 (m, 7H, H_2, H_{14}, H_{15}, H_{16}), 6.27 (brd, 1H, H_{12}), 3.30 (s, 3H, NC<u>H</u>$_3$), 1.52 (s, 9H, H_9), 1.41 (s, 9H, H_{10}).

^{13}C NMR (150 MHz, DMSO-d$_6$) δ 164.7 (C_{10}), 162.4 (C_4), 152.5 (C_5), 152.0 (C_6), 140.7 (C_{11}), 139.6 (C_{12a}), 135.8 (C_1), 134.7 (C_{3a}), 129.6 (C_2), 128.9 (C_{14}), 127.6 (C_{13}), 127.5 (C_{15}), 123.2 (C_3), 119.0 (C_{12}), 83.4 (C_7), 78.9 (C_8), 37.0 (N<u>C</u>H$_3$), 27.9 (C_9), 27.6 (C_{10}).

HRMS (m/z ESI$^+$): m/z found 495.2608 $[M+1]^+$; $C_{27}H_{35}N_4O_5$ required 495.2602

(*E*)-*N*-(4-(2,3-bis(*tert*-butoxycarbonyl)guanidino))-3-(4-methoxyphenyl)-*N*-methylacrylamide, 60 (scheme 4)

Compound **60** was prepared according to ***Method A.1***. (quant. yield)

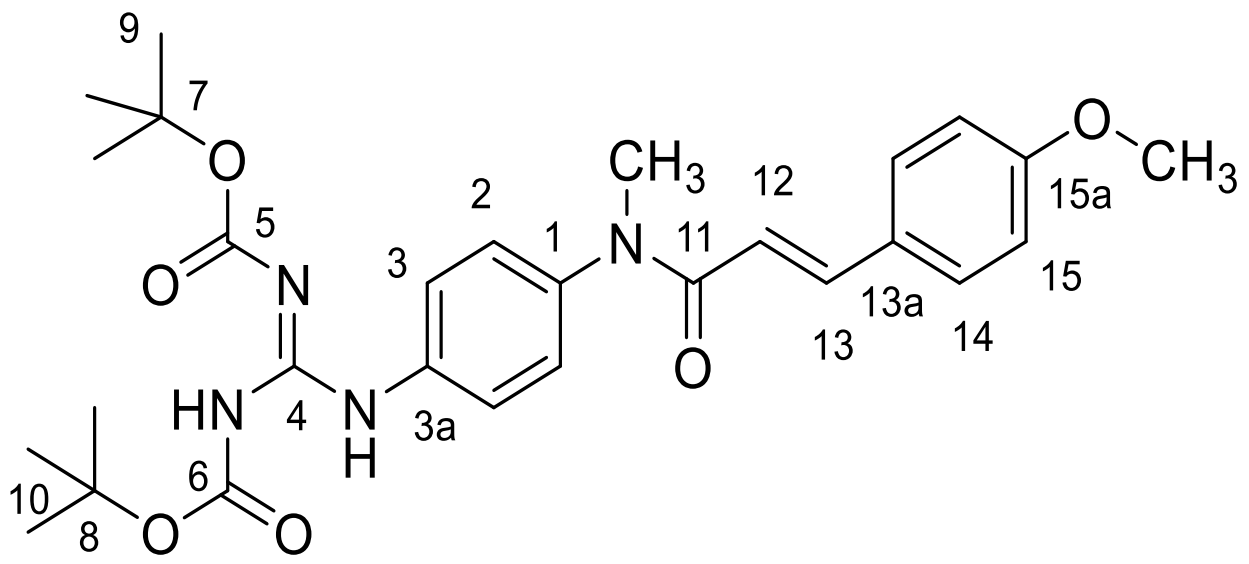

^{1}H NMR (600 MHz, DMSO-d$_6$) δ 11.38 (s, 1H, N<u>H</u>Boc), 10.08 (s, 1H, N<u>H</u>Ar), 7.66 (d, 2H, H_3, $J_{2\text{-}3}$ = 8.5 Hz), 7.48 (d, 1H, H_{13}, $J_{12\text{-}13}$ = 15.3 Hz), 7.35 (d, 2H, H_{14}, $J_{14\text{-}15}$ = 8.6 Hz), 7.31 (d, 2H, H_2, $J_{2\text{-}3}$ = 8.5 Hz), 6.89 (d, 2H, H_{15}, $J_{14\text{-}15}$ = 8.6 Hz), 6.27 (d, 1H, H_{12}, $J_{12\text{-}13}$ = 15.3 Hz), 3.74 (s, 3H, OC<u>H</u>$_3$), 3.28 (s, 3H, NC<u>H</u>$_3$), 1.51 (s, 9H, H_9), 1.42 (s, 9H, H_{10}).

^{13}C NMR (150 MHz, DMSO-d$_6$) δ 165.0 (C_{11}), 160.5 (C_{15a}), 152.5 (C_4), 140.4 (C_{13}), 139.8 (C_1), 135.7 (C_{3a}), 129.3 (C_{14}), 127.4 (C_{13a}), 127.3 (C_2), 123.2 (C_3), 116.4 (C_{12}), 114.3 (C_{15}), 83.3 (C_7), 78.9 (C_8), 55.2 (O<u>C</u>H$_3$), 37.0 (N<u>C</u>H$_3$), 27.7 (C_9, C_{10}).

HRMS (m/z ESI$^+$): m/z found 525.2714 $[M+1]^+$; $C_{28}H_{37}N_4O_6$ required 525.2708

N-(4-(2,3-bis(*tert*-butoxycarbonyl)guanidino)benzothiazol-6-amine, 61 (scheme 5)

Compound **61** was prepared according to ***Method E.1***. (68% yield)

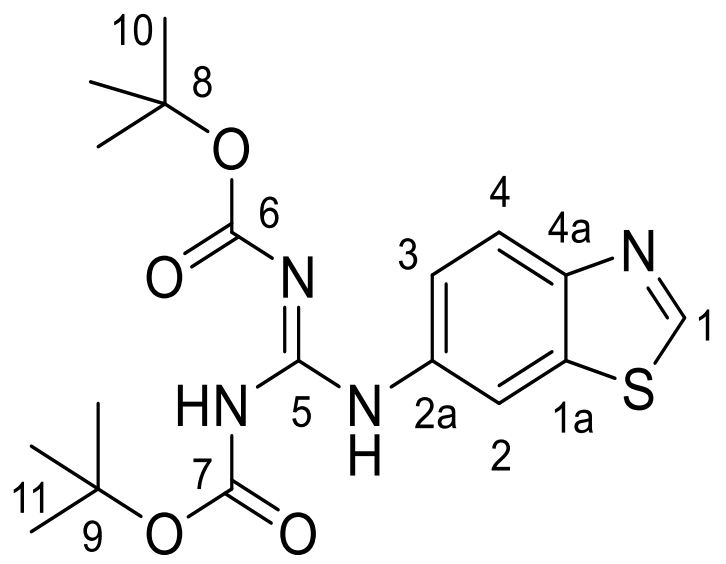

^{1}H NMR (400 MHz, CD_2Cl_2) δ 11.69 (s, 1H, NHBoc), 10.48 (s, 1H, NHAr), 8.96 (s, 1H, H_1), 8.46 (s, 1H, H_2), 8.06 (d, 1H, H_4, J_{3-4} = 8.8 Hz), 7.52 (d, 1H, H_4, J_{3-4} = 8.8 Hz), 1.57 (s, 9H, H_{10}), 1.50 (s, 9H, H_{11}).

^{13}C NMR (100 MHz, CD_2Cl_2) δ 153.9 (C_6), 153.7 (C_1), 153.3 (C_7), 150.6 (C_{2a}), 134.6 (C_{1a}), 134.5 (C_{4a}), 123.3 (C_4), 121.7 (C_3), 115.5 (C_2), 83.9 (C_8), 79.6 (C_9), 27.9 (C_{10}), 27.8 (C_{11}).

HRMS (m/z ESI$^+$): m/z found 393.1598 $[M+1]^+$; $C_{18}H_{25}N_4O_4S$ required 393.1592

***N*-(2,3-bis(*tert*-butoxycarbonyl)guanidino)- 6-amino-2-acetyl benzothiazole, 62 (scheme 5)**

Compound **62** was prepared according to ***Method D***. (23% yield)

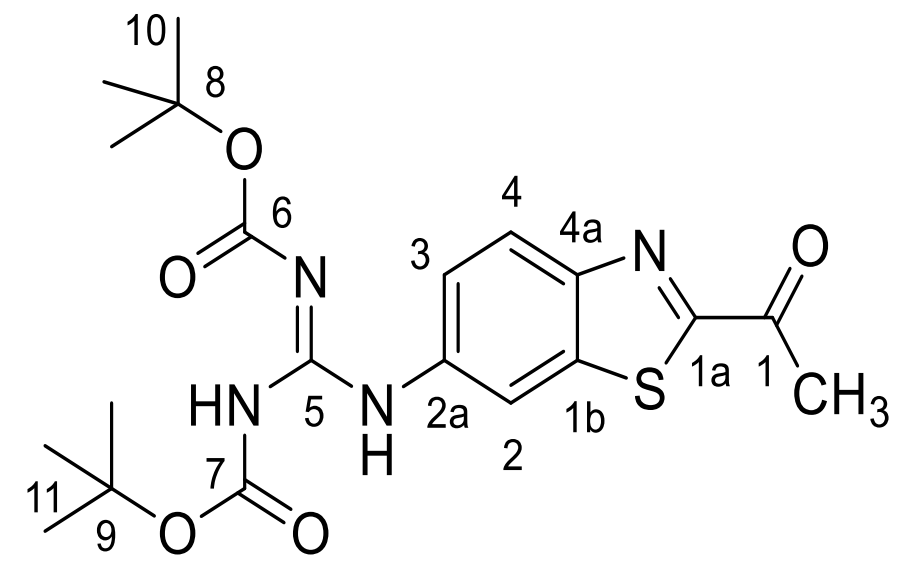

^{1}H NMR (400 MHz, CD_2Cl_2) δ 8.53 (s, 1H, H_2), 8.12 (d, 1H, H_4, J_{3-4} = 8.8 Hz), 7.60 (dd, 1H, H_3, J_{3-4} = 8.8 Hz, J_{2-3} = 1.9 Hz), 2.79 (s, 3H, $C\underline{H}_3$), 1.57 (s, 9H, H_{10}), 1.50 (s, 9H, H_{11}).

^{13}C NMR (100 MHz, CD_2Cl_2) δ 192.8 (C_1), 166.2 (C_{1a}), 161.4 (C_5), 153.5 (C_6), 150.6 (C_7), 138.4 (C_{2a}), 136.6 (C_{1b}), 125.4 (C_4), 122.3 (C_3), 115.0 (C_2), 27.8 (C_{10}, C_{11}), 25.8 ($\underline{C}H_3$).

HRMS (m/z ESI$^+$): m/z found 435.1690 $[M+1]^+$; $C_{20}H_{27}N_4O_5S$ required 435.1697

***N*-(2,3-bis(*tert*-butoxycarbonyl)guanidino)-6-amino-2-(2-phenylacetyl) benzothiazole, 63 (scheme 5)**

Compound **63** was prepared according to ***Method D***. (21% yield)

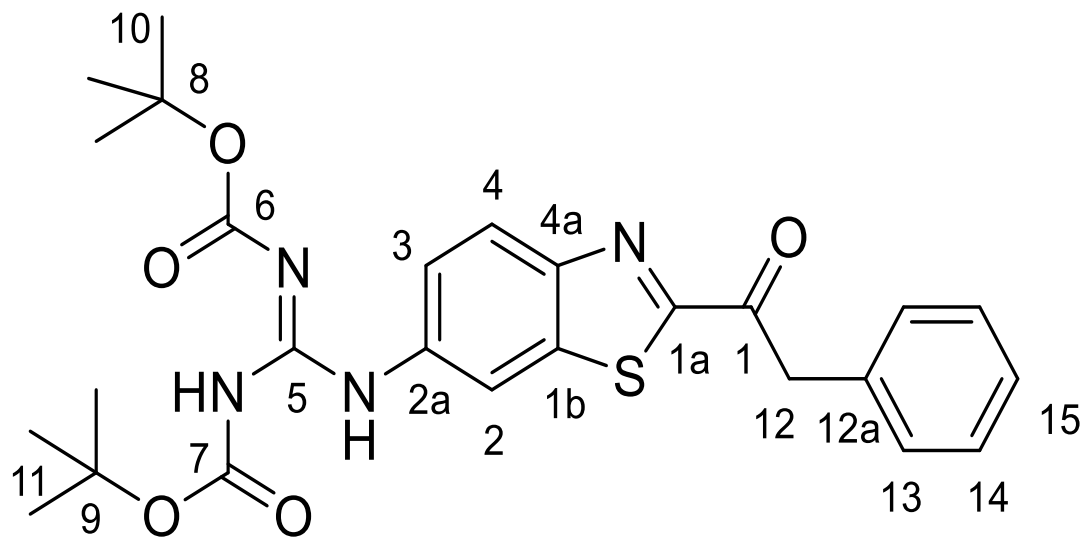

^{1}H NMR (400 MHz, CD_2Cl_2) δ 11.98 (s, 1H, N<u>H</u>Boc), 8.41 (s, 1H, H_2), 8.22 (d, 1H, H_4, $J_{3\text{-}4}$ = 8.6 Hz), 7.61 (dd, 1H, H_3, $J_{3\text{-}4}$ = 8.6 Hz, $J_{2\text{-}3}$ = 2.0 Hz), 7.45 – 7.25 (d, 2H, H_4, $J_{3\text{-}4}$ = 8.3 Hz), 4.60 (s, 2H, H_{12}), 1.61 (s, 9H, H_{10}), 1.46 (s, 9H, H_{11}).

^{13}C NMR (150 MHz, CD_2Cl_2) δ 192.3 (C_1), 166.1 (C_{1a}), 153.4 (C_5), 150.9 (C_{2a}), 138.5 (C_{4a}), 133.7 (C_{12a}), 129.9 (C_{13}), 128.5 (C_{14}), 127.1 (C_{15}), 125.7 (C_4), 122.2 (C_3), 115.2 (C_2), 86.0 (C_8, C_9), 44.7 (C_{12}), 27.7 (C_{10}, C_{11}).

HRMS (m/z ESI$^+$): m/z found 511.2121 $[M+1]^+$; $C_{26}H_{31}N_4O_5S$ required 511.2012

***N*-(2,3-bis(*tert*-butoxycarbonyl)guanidino)-6-amino-2-benzoyl benzothiazole, 64 (scheme 5)**

Compound **64** was prepared according to ***Method D***. (17% yield)

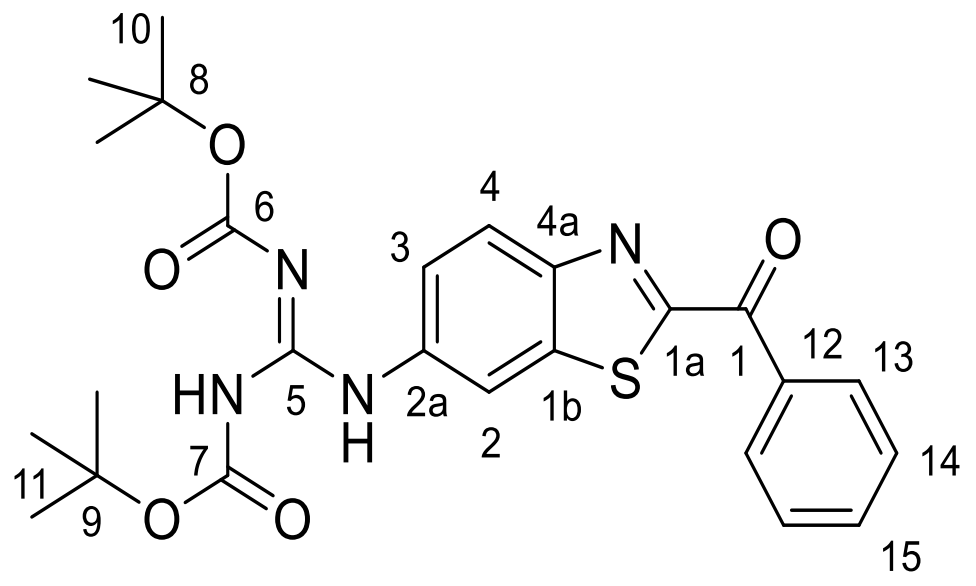

^{1}H NMR (600 MHz, CD_2Cl_2) δ 11.72 (s, 1H, N<u>H</u>Boc), 10.68 (s, 1H, N<u>H</u>Ar), 8.63 (d, 1H, H_2, J_{2-3} = 1.6 Hz), 8.58 (d, 2H, H_{13}, J_{13-14} = 7.5 Hz), 8.21 (d, 1H, H_4, J_{3-4} = 9.0 Hz), 7.73 (t, 1H, H_{15}, J_{14-15} = 7.5 Hz), 7.69 – 7.58 (m, 3H, H_3, H_{14}), 1.60 (s, 9H, H_{10}), 1.55 (s, 9H, H_{11}).

^{13}C NMR (150 MHz, CD_2Cl_2) δ 185.0 (C_1), 166.7 (C_5), 153.6 (C_6), 150.9 (C_7), 138.0 (C_{2a}), 136.8 (C_{4a}), 135.1 (C_{12}), 133.8 (C_{15}), 131.1 (C_{13}), 128.4 (C_{14}), 125.6 (C_4), 122.3 (C_3), 114.6 (C_2), 84.2 (C_8), 79.9 (C_9), 27.9 (C_{10}), 27.8 (C_{11}),

HRMS (m/z ESI$^+$): m/z found 497.1862 $[M+1]^+$; $C_{25}H_{29}N_4O_5S$ required 497.1854

N-4-(2,3-bis(*tert*-butoxycarbonyl)guanidino) nitrobenzene (scheme 4)

p-Nitroaniline was guanidylated according to ***Method E.1*** affording the aimed compound (61% yield)

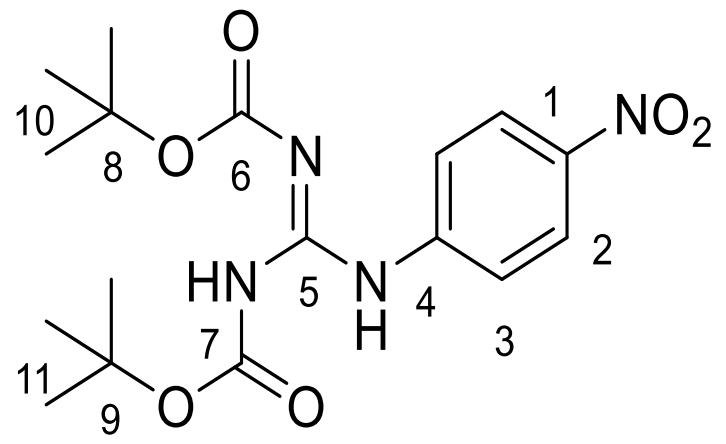

^{1}H NMR (400 MHz, DMSO-d$_6$) δ 11.05 (s, 1H, N<u>H</u>Boc), 10.27 (s, 1H, N<u>H</u>Ar), 8.21 (d, 2H, H_2, $J_{2\text{-}3}$ = 8.6 Hz), 7.83 (d, 2H, H_3, $J_{2\text{-}3}$ = 8.6 Hz), 1.48 (s, 9H, H_9), 1.41 (s, 9H, H_{10}).

^{13}C NMR (100 MHz, DMSO-d$_6$) δ 162.0 (C_5), 152.1 (C_6), 151.5 (C_7), 144.0 (C_1), 144.0 (C_4), 125.1 (C_2), 121.9 (C_3), 83.6 (C_8), 79.8 (C_9), 28.1 (C_{10}, C_{11}).

N^1-4-(2,3-bis(*tert*-butoxycarbonyl)guanidino)dianiline (scheme 4)

N-4-(2,3-bis(*tert*-butoxycarbonyl)guanidino)nitrobenzene was reduced according to ***Method C.1*** affording the aimed compound in quantitative yield.

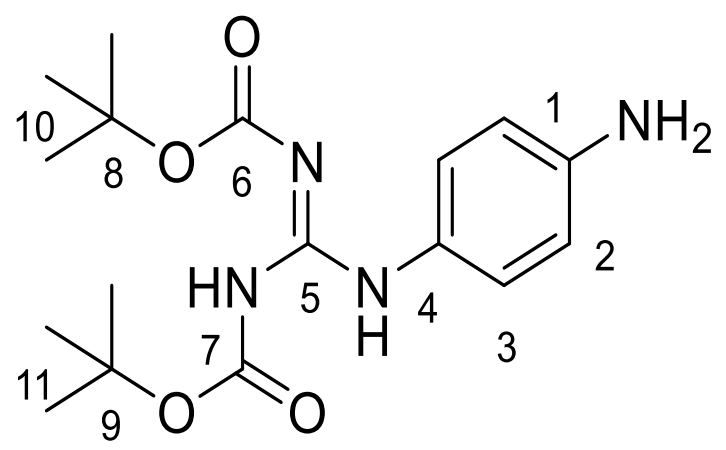

^{1}H NMR (400 MHz, DMSO-d$_6$) δ 11.11 (brd, 1H, N<u>H</u>Boc), 9.68 (s, 1H, N<u>H</u>Ar), 7.08 (d, 2H, H_3, $J_{2\text{-}3}$ = 8.7 Hz), 6.52 (d, 2H, H_2, $J_{2\text{-}3}$ = 8.7 Hz), 5.06 (s, 2H, N<u>H</u>$_2$), 1.44 (s, 18H, H_9, H_{10}).

^{13}C NMR (100 MHz, DMSO-d$_6$) δ 154.0 (C_6), 147.1 (C_1), 125.3 (C_3), 114.0 (C_2), 83.68(C_8), 79.3 (C_9), 28.5 (C_{11}), 28.1 (C_{10}).

Mono-Boc derivatives

tert-Butyl (E)-(4-(but-2-enamido)phenyl)carbamate (scheme 4)

This compound was prepared according to ***Method A.2***. (quant. yield)

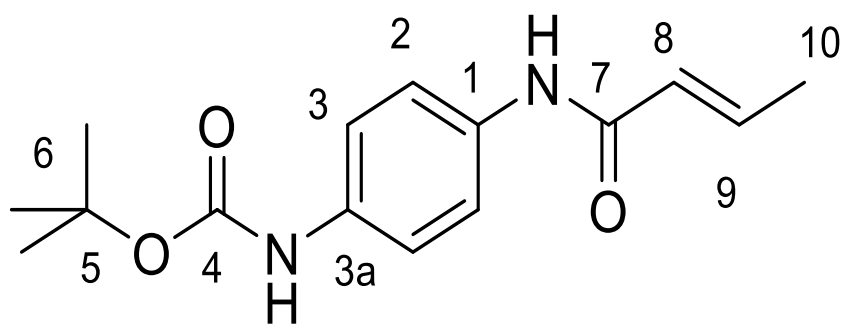

^{1}H NMR (400 MHz, DMSO-d_6) δ 9.79 (s, 1H, N<u>H</u>Boc), 9.21 (s, 1H, N<u>H</u>C=O), 7.48 (d, 2H, H_3, $J_{2\text{-}3}$ = 8.7 Hz), 7.33 (d, 2H, H_2, $J_{2\text{-}3}$ = 8.7 Hz), 6.72 (dq, 1H, H_9, $J_{8\text{-}9}$ = 15.0 Hz, $J_{9\text{-}10}$ = 7.0 Hz), 6.06 (d, 1H, H_8, $J_{8\text{-}9}$ = 15.0 Hz), 1.71 (d, 3H, H_{10}, $J_{9\text{-}10}$ = 7.0 Hz), 1.44 (s, 9H, H_6).

^{13}C NMR (100 MHz, DMSO-d_6) δ 163.5 (C_7), 153.3 (C_4), 139.6 (C_8), 135.4 (C_1), 134.2 (C_{3a}), 126.5 (C_9), 120.1 (C_3), 118.9 (C_2), 79.3 (C_5), 28.6 (C_6), 17.9 (C_{10}).

HRMS (m/z ESI$^+$): m/z found 277.1551 $[M+1]^+$; $C_{15}H_{21}N_2O_3$ required 277.1547

***tert*-Butyl (4-(3-methylbut-2-enamido)phenyl)carbamate (scheme 4)**

This compound was prepared according to ***Method A.2***. (quant. yield)

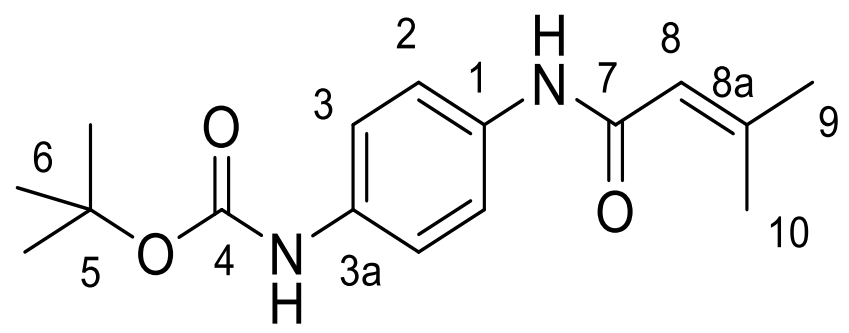

^{1}H NMR (400 MHz, DMSO-d$_6$) δ 9.66 (s, 1H, NHBoc), 9.18 (s, 1H, NHC=O), 7.46 (d, 2H, H_3, $J_{2\text{-}3}$ = 8.7 Hz), 7.31 (d, 2H, H_2, $J_{2\text{-}3}$ = 8.7 Hz), 5.81 (s, 1H, H_8), 2.11 (s, 3H, H_9), 1.82 (s, 3H, H_{10}), 1.44 (s, 9H, H_6).

^{13}C NMR (100 MHz, DMSO-d$_6$) δ 164.7 (C_7), 153.3 (C_4), 151.0 (C_{8a}), 135.1 (C_{3a}), 134.5 (C_1), 119.9 (C_3), 119.7 (C_8), 118.9 (C_2), 79.2 (C_5), 28.6 (C_6), 27.5 (C_{11}), 19.9 (C_{10}).

HRMS (m/z ESI$^+$): m/z found 291.1711 $[M+1]^+$; $C_{16}H_{23}N_2O_3$ required 291.1704

***tert*-Butyl (4-(cyclohex-1-ene-1-carboxamido)phenyl)carbamate (scheme 4)**

This compound was prepared according to ***Method A.2***. (quant. yield)

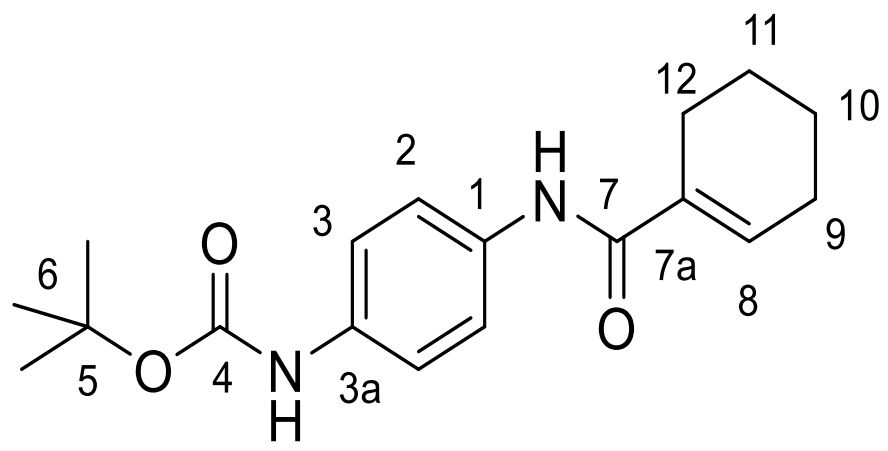

^{1}H NMR (400 MHz, DMSO-d$_6$) δ 9.42 (s, 1H, N<u>H</u>Boc), 9.19 (s, 1H, N<u>H</u>C=O), 7.49 (d, 2H, H_3, $J_{2\text{-}3}$ = 8.3 Hz), 7.32 (d, 2H, H_2, $J_{2\text{-}3}$ = 8.3 Hz), 6.60 (s, 1H, H_8), 2.22 – 2.08 (m, 4H, H_9, H_{12}), 1.66 – 1.50 (m, 4H, H_{10}, H_{11}), 1.46 (s, 9H, H_6).

^{13}C NMR (100 MHz DMSO-d$_6$) δ 167.0 (C_7), 153.2 (C_4), 135.4 (C_{3a}), 134.5 (C_1), 134.2 (C_{7a}), 132.9 (C_8), 121.0 (C_3), 118.7 (C_2), 78.9 (C_5), 27.7 (C_6), 25.3 (C_9), 24.5 (C_{12}), 22.2 (C_{10}), 21.7 (C_{11}).

HRMS (m/z ESI$^+$): m/z found 317.1799 [M+1]$^+$; $C_{18}H_{25}N_2O_3$ required 317.1806

tert-Butyl (4-(3,4-dihydro-2H-pyran-6-carboxamido)phenyl)carbamate (scheme 4)

This compound was prepared according to ***Method A.1***. (quant. yield)

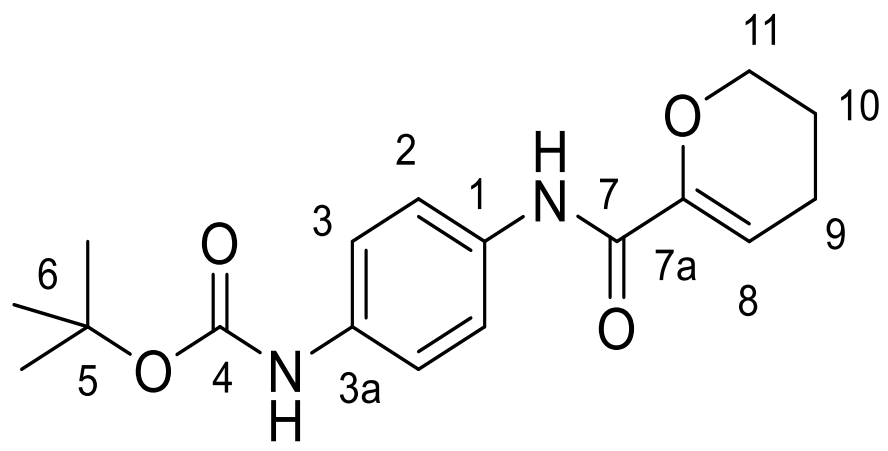

^{1}H NMR (400 MHz, DMSO-d$_6$) δ 9.45 (s, 1H, N<u>H</u>Boc), 9.25 (s, 1H, N<u>H</u>C=O), 7.58 (d, 2H, H_3, $J_{2\text{-}3}$ = 8.4 Hz), 7.34 (d, 2H, H_2, $J_{2\text{-}3}$ = 8.4 Hz), 5.88 (d, 1H, H_2, $J_{7\text{-}8}$ = 5.6 Hz), 4.10 (d, 2H, H_{11}, $J_{11\text{-}12}$ = 5.0 Hz), 2.20 – 2.10 (m, 2H, H_9), 1.86 – 1.76 (m, 2H, H_{10}), 1.47 (s, 9H, H_6).

^{13}C NMR (150 MHz DMSO-d$_6$) δ 159.8 (C_7), 152.8 (C_4), 146.3 (C_{7a}), 135.4 (C_{3a}), 132.7 (C_1), 120.9 (C_3), 118.2 (C_2), 106.5 (C_8), 78.8 (C_5), 66.2 (C_{11}), 28.1 (C_6), 21.3 (C_{10}), 19.9 (C_9).

HRMS (m/z ESI$^+$): m/z found 319.1660 $[M+1]^+$; $C_{17}H_{23}N_2O_4$ required 319.1653

***tert*-butyl (4-cinnamamidophenyl)carbamate (scheme 4)**

This compound was prepared according to ***Method A.2***. (quant. yield)

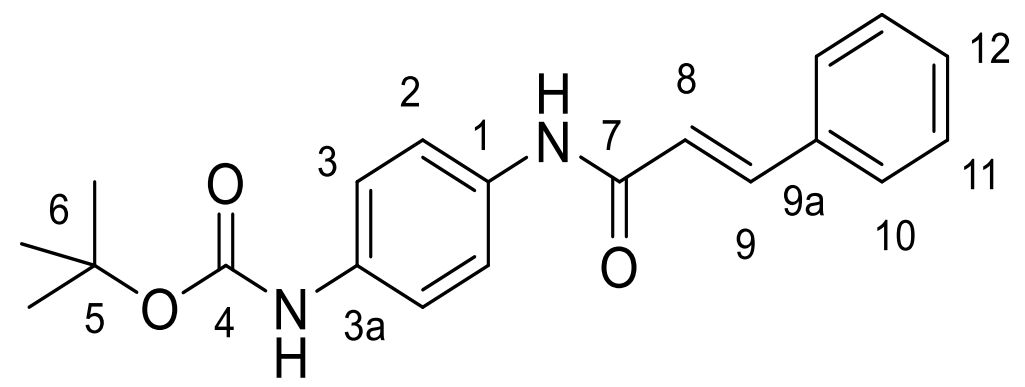

^{1}H NMR (400 MHz, DMSO-d$_6$) δ 10.09 (s, 1H, N<u>H</u>Boc), 10.08 (s, 1H, N<u>H</u>C=O), 7.46 – 7.26 (m, 10H, H_2, H_3, H_8, H_{10}, H_{11}, H_{12}), 6.80 (d, 1H, H_9, $J_{8\text{-}9}$ = 15.5 Hz), 1.45 (s, 9H, H_{16}).

^{13}C NMR (100 MHz, DMSO-d$_6$) δ 163.60 (C_7), 153.3 (C_4), 140.1 (C_9), 135.6 (C_{3a}), 135.3 (C_1), 134.2 (C_{9a}), 130.1 (C_{10}), 129.4 (C_{11}), 128.1 (C_{12}), 122.9 (C_8), 120.1 (C_2), 119.0 (C_3), 79.3 (C_5), 28.6 (C_6).

HRMS (m/z ESI$^+$): m/z found 339.1697 $[M+1]^+$; $C_{20}H_{23}N_2O_3$ required 339.1704

tert-Butyl (_E_)-(4-(3-(4-methoxyphenyl)acrylamido)phenyl)carbamate (scheme 4)

This compound was prepared according to ***Method A.2***. (quant. yield)

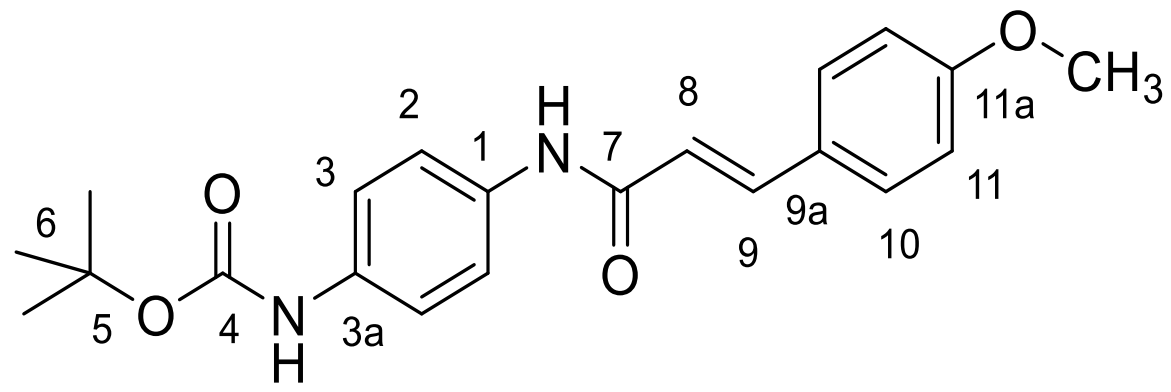

^{1}H NMR (400 MHz, DMSO-d$_6$) δ 9.97 (s, 1H, N$\underline{H}$Boc), 9.23 (s, 1H, N$\underline{H}$C=O), 7.58 – 7.50 (m, 4H, H_3, H_{10}), 7.47 (d, 1H, H_9, $J_{8\text{-}9}$ = 15.7 Hz), 7.36 (d, 2H, H_3, $J_{2\text{-}3}$ = 8.5 Hz), 6.98 (d, 2H, H_{10}, $J_{10\text{-}11}$ = 8.0 Hz), 6.64 (d, 1H, H_8, $J_{8\text{-}9}$ = 15.7 Hz), 3.78 (s, 3H, OC$\underline{H}_3$), 1.45 (s, 9H, H_6).

^{13}C NMR (100 MHz, DMSO-d$_6$) δ 163.9 (C_7), 160.9 (C_{14}), 153.23 (C_{3a}), 139.9 (C_9), 134.4 (C_1), 129.7 (C_3), 127.8 (C_{9a}), 120.3 (C_{10}), 120.0 (C_2), 119.0 (C_8), 114.9 (C_{11}), 79.3 (C_5), 55.7 (O$\underline{C}H_3$), 28.6 (C_6).

HRMS (m/z ESI$^+$): m/z found 369.1812 $[M+1]^+$; $C_{21}H_{25}N_2O_6$ required 369.1809

tert-Butyl (4-(but-2-ynamido)phenyl)carbamate (scheme 4)

This compound was prepared according to ***Method A.2***. (quant. yield)

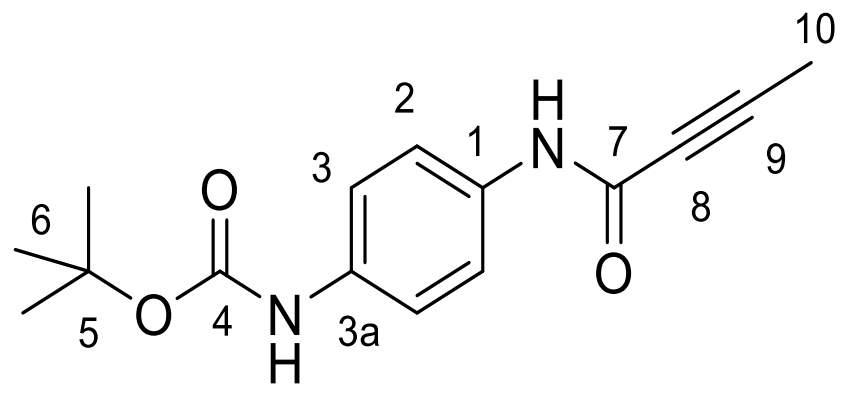

^{1}H NMR (400 MHz, DMSO-d$_6$) δ 10.42 (s, 1H, N<u>H</u>Boc), 9.24 (s, 1H, N<u>H</u>C=O), 7.42 (d, 2H, H_3, $J_{2\text{-}3}$ = 9.0 Hz), 7.31 (d, 2H, H_2, $J_{2\text{-}3}$ = 9.0 Hz), 2.00 (s, 3H, H_{10}), 1.44 (s, 9H, H_6).

^{13}C NMR (100 MHz, DMSO-d$_6$) δ 153.2 (C_4), 150.6 (C_7), 135.9 (C_{3a}), 133.5 (C_1), 120.4 (C_2), 118.8 (C_3), 84.1 (C_8), 79.3 (C_5), 76.4 (C_9), 28.6 (C_6), 3.6 (C_{10}).

HRMS (m/z ESI$^+$): m/z found 275.1321 $[M+1]^+$; $C_{15}H_{19}N_2O_3$ required 275.1319

Starting compounds

Octyl 4-aminobenzoate (scheme S1)

Commercially available 4-aminobenzoic acid (1.37 g, 10 mmol, 1 eq.) was dissolved in 12.5 mL of *n*-Octanol (0.4 M). Upon dissolution, H_2SO_4 (650 µL, 12 mmol, 1.2 eq.) was added and the mixture was heated at 110° C for 16 hours. The solution is stirred at room temperature for 16 hours. The crude was diluted with 50 mL of CH_2Cl_2 and washed with water (1 x 50 mL) and sat. $NaHCO_3$ (1 x 50 mL). The organic phase concentrated under vacuo and purified *via* liquid flash chromatography Eluent: Hexane with a gradient of EtOAc from 0% to 40%). Product (colourless oil) was obtained in an 82% yield.

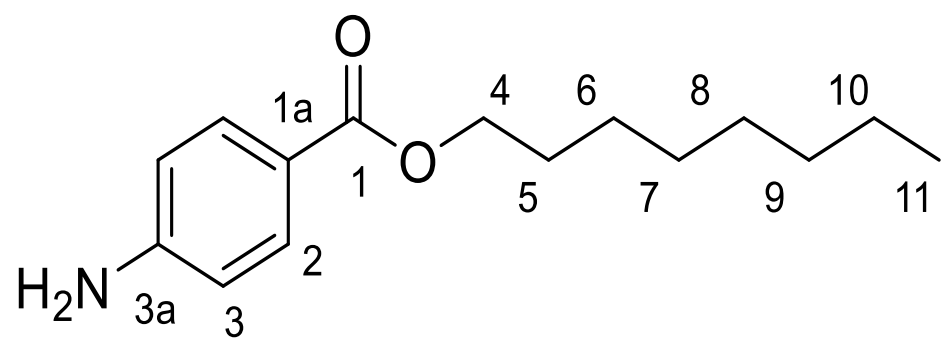

^{1}H NMR (400 MHz, DMSO-d$_6$) δ 7.60 (d, 2H, H_2, $J_{2\text{-}3}$ = 8.1 Hz), 6.53 (d, 2H, H_3, $J_{2\text{-}3}$ = 8.1 Hz), 5.91 (s, 2H, N$\underline{H}_2$), 4.11 (t, 2H, H_4, $J_{4\text{-}5}$ = 6.9 Hz), 1.62 (q, 2H, H_{12}, $J_{4\text{-}5}$ = $J_{5\text{-}6}$ = 7.0 Hz), 1.40 – 1.16 (m, 10H, H_6, H_7, H_8, H_9, H_{10}), 0.83 (t, 3H, H_{11}, $J_{10\text{-}11}$ = 6.7 Hz).

^{13}C NMR (100 MHz, DMSO-d$_6$) δ 166.3 (C_1), 153.9 (C_{3a}), 131.4 (C_2), 116.5 (C_{1a}), 113.1 (C_3), 63.9 (C_4), 31.7 (C_5), 29.1 (C_6), 29.0 (C_7), 28.8 (C_8), 26.0 (9_9), 22.5 (C_{10}), 14.4 (C_{11}).

HRMS (m/z ESI$^+$): m/z found 250.1811 $[M+1]^+$; $C_{15}H_{24}NO_2$ required 250.1802

Prop-2-yn-1-yl 4-nitrobenzoate, 42 (scheme S1)

Commercially available 4-nitrobenzoic acid (1.37 g, 10 mmol, 1 eq.) was dissolved in 12.5 mL of Propargyl alcohol (0.4 M). Upon dissolution, H_2SO_4 (650 µL, 12 mmol, 1.2 eq.) was added and the mixture was heated at 110° C for 16 hours. The solution is stirred at room temperature for 16 hours. The crude was diluted with 50 mL of CH_2Cl_2 and washed with water (1 x 50 mL) and sat. $NaHCO_3$ (1 x 50 mL). The organic phase concentrated under vacuo and purified *via* liquid flash chromatography Eluent: Hexane with a gradient of EtOAc from 0% to 30%). Product **42** (orange solid) was obtained in an 63% yield.

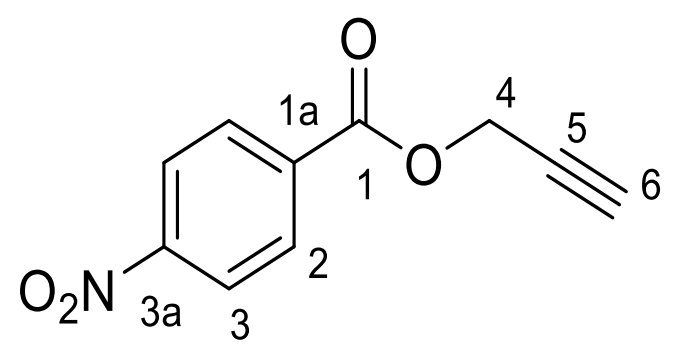

^{1}H NMR (400 MHz, DMSO-d$_6$) δ 8.34 (d, 2H, H_3, $J_{2\text{-}3}$ = 8.7 Hz), 8.18 (d, 2H, H_2, $J_{2\text{-}3}$ = 8.7 Hz), 5.00 (d, 2H, H_4, $J_{4\text{-}6}$ = 2.2 Hz), 3.65 (s, 1H, H_6).

^{13}C NMR (100 MHz, DMSO-d$_6$) δ 164.0 (C_1), 150.9 (C_{3a}), 134.8 (C_{1a}), 131.2 (C_2), 124.4 (C_3), 78.9 (C), 78.4 (C_5), 53.8 (C_4).

HRMS (m/z ESI$^+$): m/z found 206.0449 $[M+1]^+$; $C_{10}H_8NO_4$ required 206.0448

N-methoxy-*N*-methyl-4-nitrobenzamide, 43 (scheme S1)

Compound **43** was prepared according to ***Method B.1*** from 4-nitrobenzoic acid. (91% yield)

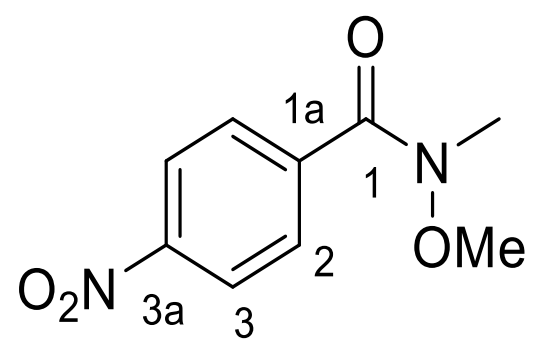

^{1}H NMR (400 MHz, DMSO-d$_6$) δ 8.26 (d, 2H, H_3, $J_{2\text{-}3}$ = 8.3 Hz), 7.80 (d, 2H, H_2, $J_{2\text{-}3}$ = 8.3 Hz), 3.52 (s, 3H, $OC\underline{H}_3$), 3.27 (s, 3H, $NC\underline{H}_3$).

^{13}C NMR (100 MHz, DMSO-d$_6$) δ 167.8 (C_1), 148.7 (C_{3a}), 141.2 (C_{1a}), 129.3 (C_2), 123.8 (C_3), 61.5 ($O\underline{C}H_3$), 55.3 ($N\underline{C}H_3$).

HRMS (m/z ESI$^+$): m/z found 211.0707 $[M+1]^+$; $C_9H_{11}N_2O_4$ required 211.0714

5-Nitro-1*H*-indole-2-carboxylic acid, 51 (scheme 3)

Commercially available ethyl 5-nitro-1*H*-indole-2-carboxylate (2.34 g, 10 mmol, 1 eq.) was dissolved in 100 mL of EtOH (0.1 M). LiOH (265 mg, 11 mmol, 1.1 eq.) was dissolved in 100 mL of water. The two solutions are combined, and the reaction is stirred at 40° C for 6 h. Most of the ethanol is removed at low pressure. The aqueous phase is acidified at pH 2 with 1M HCl. The product is extracted with EtOAc (100 mL x 2). The organic phase dried over Na_2SO_4 and concentrated under vacuo. Product **51** (orange solid) was obtained in quantitative yield. No further purification is needed.

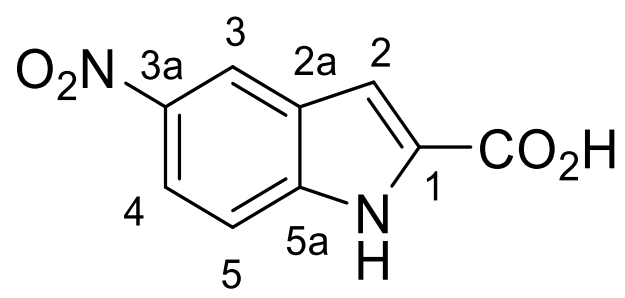

^{1}H NMR (400 MHz, DMSO-d$_6$) δ 13.37 (s, 1H, $CO_2\underline{H}$), 12.45 (s, 1H, N$\underline{H}$), 8.69 (d, 1H, H_3, $J_{3\text{-}4}$ = 2.2 Hz), 8.09 (dd, 1H, H_4, $J_{4\text{-}5}$ = 8.9 Hz, $J_{3\text{-}4}$ = 2.2 Hz), 7.56 (d, 1H, H_5, $J_{4\text{-}5}$ = 8.9 Hz), 7.35 (d, 1H, H_2).

^{13}C NMR (100 MHz, DMSO-d$_6$) δ 162.6 ($\underline{C}O_2H$), 141.8 (C_{3a}), 140.8 (C_{5a}), 132.5 (C_{2a}), 126.5 (C_{1a}), 120.0 (C_3), 119.6 (C_4), 113.5 (C_5), 110.2 (C_2).

2-Azideethanol, 58 (scheme S4)

2-Chloroethanol (4 mL, 60 mmol, 1.2 eq.) and NaN_3 (3.9 g, 50 mmol, 1 eq.) were dissolved in 50 mL of water. The reaction was stirred for 3 days at 50° C. Upon completion (accessed *via* ^{1}H-NMR – D_2O) The reaction mixture was diluted with 50 mL of brine, and the product was extracted with DCM (50 mL x 3). The combined organic phases were dried over MgSO4 and evaporated at low pressure. A 60% w/w DCM solution of 2-Azidoethanol was obtained (accessed *via* ^{1}H-NMR – DMSO-d^6).

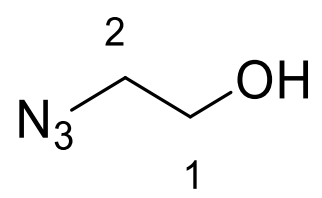

^{1}H NMR (400 MHz, DMSO-d_6) δ 4.97 (t, 1H, O<u>H</u>, $J_{1\text{-OH}}$ = 5.3 Hz), 3.56 (q, 1H, H_1, $J_{1\text{-OH}}$ = 5.3 Hz, $J_{1\text{-}2}$ = 5.1 Hz), 3.26 (t, 1H, H_1, $J_{1\text{-}2}$ = 5.1 Hz).

2,2'-((4-nitrophenyl)methylene)bis(1*H*-indole) (scheme S4)

Commercially available 4-nitro benzaldehyde (1.5 g, 10 mmol, 1 eq.) and indole (2.3 g, 20 mmol, 2 eq.) were dissolved in 50 mL of EtOH, [0.2] M. *p*-TSA*H_2O (190 mg, 1 mmol, 0.1 eq.) was added to the mixture. The solution is refluxed for 16 hours. Most of the ethanol is removed at low pressure. The crude mixture was diluted with 100 mL of dichloromethane and was washed with water (2 x 100 mL) and brine (1 x 50 mL). The organic phase was dried over Na_2SO_4 and concentrated under vacuo. Product (deep purple solid) was obtained in an 73% yield, pure enough to be directly used in the next step.

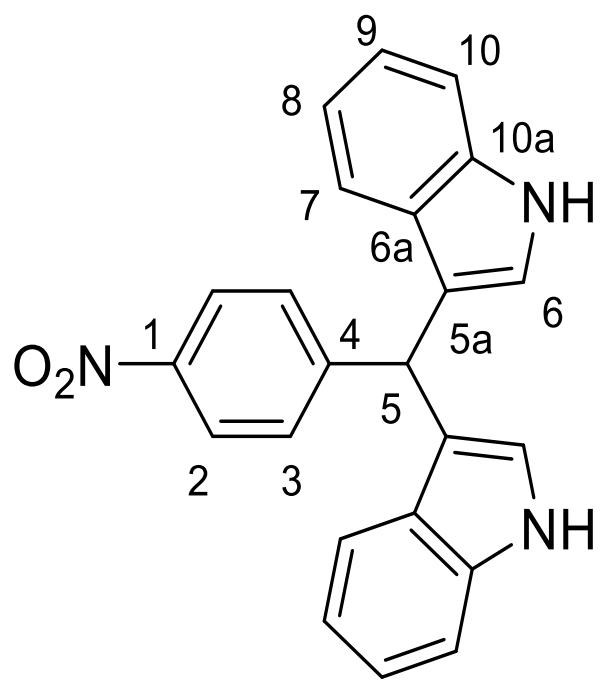

^{1}H NMR (400 MHz, DMSO-d$_6$) δ 10.91 (s, 2H, N<u>H</u>), 8.13 (d, 2H, H_2, $J_{2\text{-}3}$ = 8.3 Hz), 7.59 (d, 2H, H_3, $J_{2\text{-}3}$ = 8.3 Hz), 7.35 (d, 2H, H_7, $J_{7\text{-}8}$ = 8.3 Hz), 7.27 (d, 1H, H_{10}, $J_{9\text{-}10}$ = 8.3 Hz), 7.03 (t, 2H, H_8, $J_{7\text{-}8}$ = $J_{8\text{-}9}$ = 8.3 Hz), 6.95 – 6.80 (m, 4H, H_6, H_9), 6.01 (s, 1H, H_5).

^{13}C NMR (100 MHz, DMSO-d$_6$) δ 153.6 (C_1), 146.2 (C_4), 137.0 (C_{5a}), 129.9 (C_3), 126.8 (C_{10a}), 124.3 (C_9), 123.9 (C_2), 121.5 (C_7), 119.4 (C_8), 118.9 (C_{10}), 117.1 (C_{6a}), 112.0 (C_3), 5 (C_5).

HRMS (m/z ESI$^+$): m/z found 368.1401 $[M+1]^+$; $C_{23}H_{18}N_3O_2$ required 368.1394

Ethyl 5-amino-1*H*-indole-2-carboxylate (scheme 3)

Reduction of commercially available **Ethyl 5-nitro-1*H*-indole-2-carboxylate** was carried out according to ***Method C.2***. (92% yield)

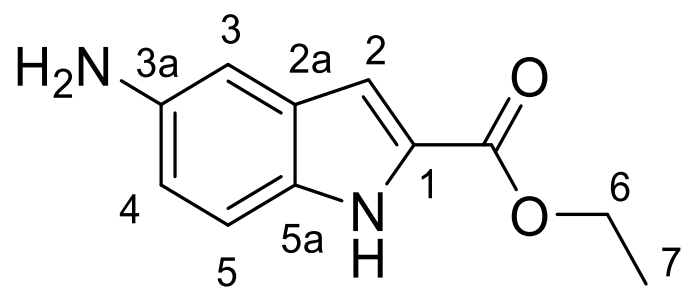

^{1}H NMR (400 MHz, $CDCl_3$) δ 8.73 (s, 1H, NH), 7.25 (d, 1H, H_5, J_{4-5} = 8.6 Hz), 7.06 (s, 1H, H_2), 6.96 (d, 1H, H_3, J_{3-4} = 2.0 Hz), 6.83 (dd, 1H, J_{4-5} = 8.6 Hz, J_{3-4} = 2.0 Hz), 4.41 (q, 2H, H_6, J_{6-7} = 7.0 Hz), 3.61 (s, 2H, NH_2), 1.42 (t, 3H, H_7, J_{6-7} = 7.0 Hz).

^{13}C NMR (100 MHz, $CDCl_3$) δ 162.0 (C_1), 140.3 (C_{5a}), 131.8 (C_{3a}), 128.5 (C_{2a}), 127.8 (C_{1a}), 117.1 (C_4), 112.5 (C_5), 107.4 (C_2), 105.9 (C_3), 60.9 (C_6), 14.4 (C_7).

HRMS (m/z ESI$^+$): m/z found 205.0983 $[M+1]^+$; $C_{11}H_{13}N_2O_2$ required 205.0972

N^1-methylbenzene-1,4-diamine (scheme 4)

N*-methyl-4-nitroaniline** was reduced according to ***Method C.1 affording the desired dianiline in quantitative yield.

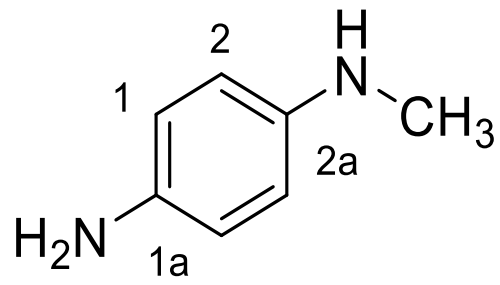

^{1}H NMR (400 MHz, DMSO-d$_6$) δ 6.42 (s, 2H, H_1), 6.32 (d, 2H, H_2), 4.36 (s, 3H, N$\underline{H}$CH$_3$, N$\underline{H}_2$), 2.55 (s, 3H, NHC$\underline{H}_3$).

^{13}C NMR (100 MHz, DMSO-d$_6$) δ 142.0 (C_{1a}), 139.2 (C_{2a}), 116.0 (C_1), 113.5 (C_2), 32.4 (NH$\underline{C}$H$_3$).

HRMS (m/z ESI$^+$): m/z found 123.0902 $[M+1]^+$; $C_7H_{11}N_2$ required 123.0917

N-methoxy-N-methyl acetamide (scheme 5)

Acetic acid was converted into compound *N*-methoxy-*N*-methylacetamide according to ***Method A.3***. (30% yield)

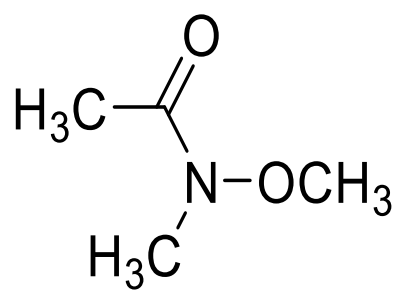

^{1}H NMR (400 MHz, DMSO-d$_6$) δ 3.63 (s, 3H, OC$\underline{H}_3$), 3.04 (s, 3H, NC$\underline{H}_3$), 1.99 (s, 3H, C$\underline{H}_3$).

^{13}C NMR (100 MHz, DMSO-d$_6$) δ 171.5 (O=$\underline{C}$CH$_3$), 61.3 (O$\underline{C}$H$_3$), 32.0 (N$\underline{C}$H$_3$), 20.1 (O=C$\underline{C}$H$_3$).

N-methoxy-N-methyl-2-phenylacetamide (scheme 5)

Phenylacetic acid was converted into compound *N*-methoxy-*N*-methyl-2-phenylacetamide according to ***Method A.3***. (quant. yield)

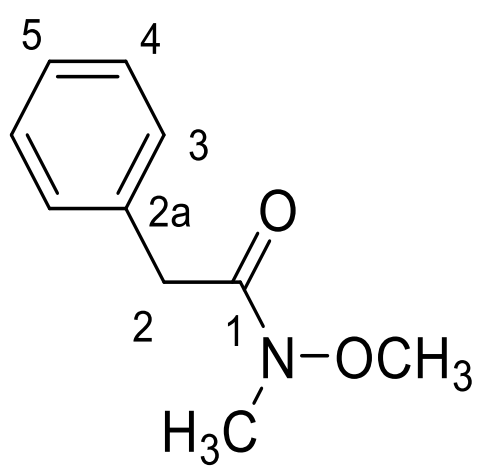

^{1}H NMR (400 MHz, DMSO-d$_6$) δ 7.40 – 7.15 (m, 5H, H_3, H_4, H_5), 3.70 (s, 2H, H_2), 3.64 (s, 3H, $OC\underline{H}_3$), 3.09 (s, 3H, $NC\underline{H}_3$).

^{13}C NMR (100 MHz, DMSO-d$_6$) δ 135.8 (C_{2a}), 129.9 (C_3), 128.6 (C_4), 126.9 (C_5), 61.5 ($O\underline{C}H_3$), 38.8 (C_2), 32.2 ($N\underline{C}H_3$).

***N*-methoxy-*N*-methyl benzamide (scheme 5)**

Benzoic acid was converted into compound *N*-methoxy-*N*-methyl benzamide according to ***Method A.3***. (quant. yield)

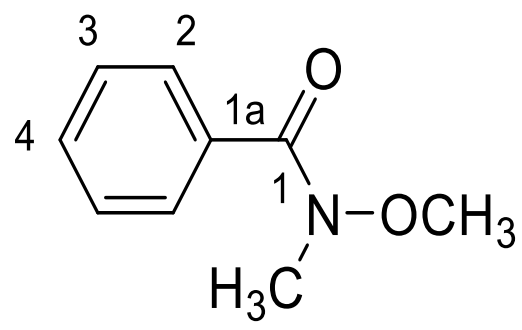

^{1}H NMR (400 MHz, DMSO-d$_6$) δ 7.70 – 7.35 (m, 5H, H_2, H_3, H_4), 3.51 (s, 3H, OC<u>H</u>$_3$), 3.23 (s, 3H, NC<u>H</u>$_3$).

^{13}C NMR (100 MHz, DMSO-d$_6$) δ 169.6 (C_1), 134.9 (C_{1a}), 130.8 (C_4), 128.5 (C_2), 128.0 (C_3), 61.1 (O<u>C</u>H$_3$), 33.7 (N<u>C</u>H$_3$).

***N*-(4-Nitrophenyl)-2-benzothiazolamine (scheme S6)**

2-Chloro benzothiazole (1 eq.) and *p*-nitro aniline (2 eq.) are dissolved in 1 mL of DMSO, [1] M. The mixture is stirred at 100 °C for 48 hours. Upon completion, 20 mL of CH_2Cl_2 are added to crush the product out, which was recovered by Buchner filtration. (40% yield)

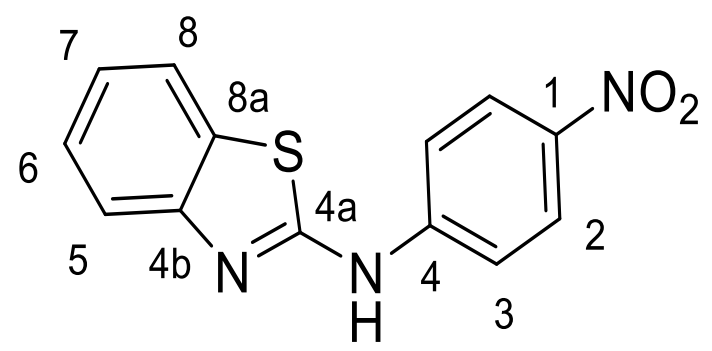

^{1}H NMR (600 MHz, DMSO-d$_6$) δ 11.36 (s, 1H, N<u>H</u>), 8.29 (d, 2H, H_2, $J_{2\text{-}3}$ = 9.5 Hz), 8.05 (d, 2H, H_3, $J_{2\text{-}3}$ = 9.5 Hz), 7.90 (d, 1H, H_5, $J_{5\text{-}6}$ = 6.6 Hz), 7.72 (d, 1H, H_8, $J_{7\text{-}8}$ = 6.6 Hz), 7.41 (t, 1H, H_6, $J_{5\text{-}6}$ = $J_{6\text{-}7}$ =6.6 Hz), 7.26 (t, 1H, H_7, $J_{6\text{-}7}$ = $J_{7\text{-}8}$ =6.6 Hz).

^{13}C NMR (150 MHz, DMSO-d$_6$) δ 161.2 (C_{4a}), 152.0 (C_{4b}), 146.9 (C_1), 141.4 (C_4), 130.8 (C_{8a}), 126.7 (C_7), 125.9 (C_3), 123.7 (C_6), 121.9 (C_8), 120.5 (C_5), 117.6 (C_2).

6-nitro-*N*-(4-Nitrophenyl)-2-benzothiazolamine (scheme S6)

2-Chloro-6-nitrobenzothiazole (1 eq.) and *p*-nitro aniline (2 eq.) were dissolved in 1 mL of DMSO, [1M]. The reaction is stirred for 48 hours at 100 °C. The precipitate is filtered over Buchner funnel and rinsed with cold dichloromethane. (45% yield)

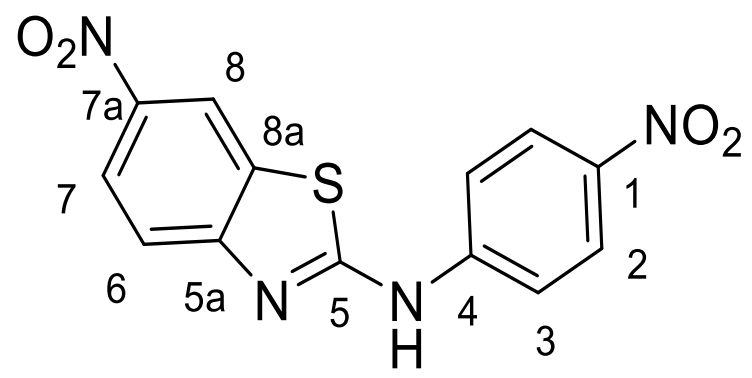

^{1}H NMR (600 MHz, DMSO-d$_6$) δ 11.61 (s, 1H, N<u>H</u>), 8.93 (d, 1H, H_8, J_{7-8} = 2.5 Hz), 8.30 (d, 2H, H_2, J_{2-3} = 9.3 Hz), 8.23 (dd, 1H, H_7, J_{6-7} = 8.9 Hz, J_{7-8} = 2.5 Hz), 8.03 (d, 2H, H_3, J_{2-3} = 9.3 Hz), 7.82 (d, 1H, H_6, J_{6-7} = 8.9 Hz).

^{13}C NMR (150 MHz, DMSO-d$_6$) δ 166.4 (C_5), 157.1 (C_{7a}), 146.0 (C_1), 143.1 (C_{5a}), 142.2 (C_4), 131.7 (C_{8a}), 125.9 (C_2), 122.6 (C_7), 120.1 (C_6), 118.9 (C_8), 118.5 (C_3).

S3. Biophysical experiments

S3.1. UV thermal denaturation experiments

Thermal melting (T_m) experiments were conducted with a Varian Cary 300 Bio spectrophotometer equipped with a 6×6 multicell temperature-controlled block. Temperatures were monitored with a thermistor inserted into a 1 cm quartz cuvette containing the same volume of water as in the sample cells. Absorbance changes at 260 nm were monitored from a range of 30 °C to 90 °C with a heating rate of 1 °C min^{-1} and a data collection rate of five points per °C. The salmon sperm DNA (st-DNA) was purchased from Sigma Aldrich (extinction coefficient ε_{260}= 6600 M^{-1} cm^{-1}). A quartz cell with a 1 cm path length was filled with a 1 mL solution of DNA or DNA-compound complex. The DNA (150 µM base) and the compound (**3**, **16**, **31** and **36**) solution (15 µM – DMSO stock) were prepared in a phosphate buffer ([10 mM K_2HPO_4/KH_2PO_4], adjusted to pH 7) so that a compound to DNA base ratio of 0.1 was obtained. The thermal melting temperatures of the duplex or duplex-compound complex obtained from the first derivative of the melting curves are reported.

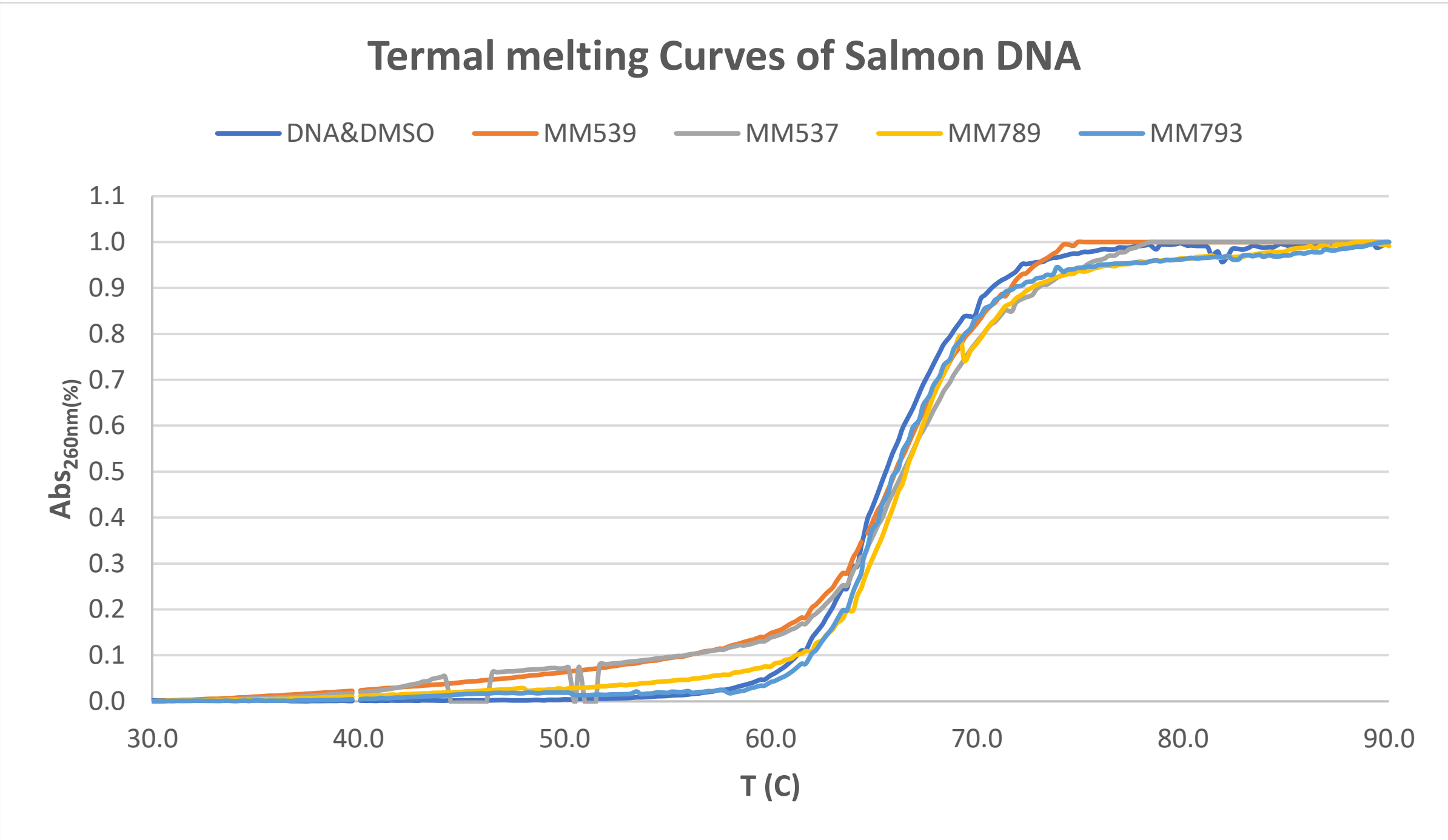

	ΔT (°C)
$T_{m\text{-Ref}}$ = 65.8° C	0.0
$T_{m\text{-MM539}}$ = 66.2° C	0.4
$T_{m\text{-MM537}}$ = 66.2° C	0.4
$T_{m\text{-MM789}}$ = 66.8° C	1.0
$T_{m\text{-MM793}}$ = 66.0° C	0.2

Figure S1. Thermal melting of st-DNA was measured with 15 µL of DMSO as reference. The normalized T_m curves of compounds **3** (**MM793**), **16** (**MM537**), **31** (**MM789**) and **36** (**MM539**) are displayed. On the table on the left, the results are reported. Almost negligible variations were found for all compounds.

S4. Cell viability assays

MCF-10 cells were seeded at $2.5x10^4$cells/well. The following day, cells were treated with either compound (0.1% (v/v) DMSO) at 0.1 µM, 1 µM, 5 µM, 10 µM, 25 µM, 50 µM and 100 µM in triplicate. After 72 hours 10% AlamarBlue (*i.e.* 20 µL) was added to each well. Plates were incubated in the dark for 6 hours at 37 °C. Fluorescence was measured using a SpectraMax Gemini plate reader (excitation wavelength 544 nm - emission wavelength 590 nm). The procedure was repeated three times. It appeared that no significant killing was achieved, indicating no cytotoxicity is exerted by compounds **3** (**MM793)**, **16** (**MM537)**, **31** (**MM789)** and **36 (MM539)** on the MCF-10 cell line.

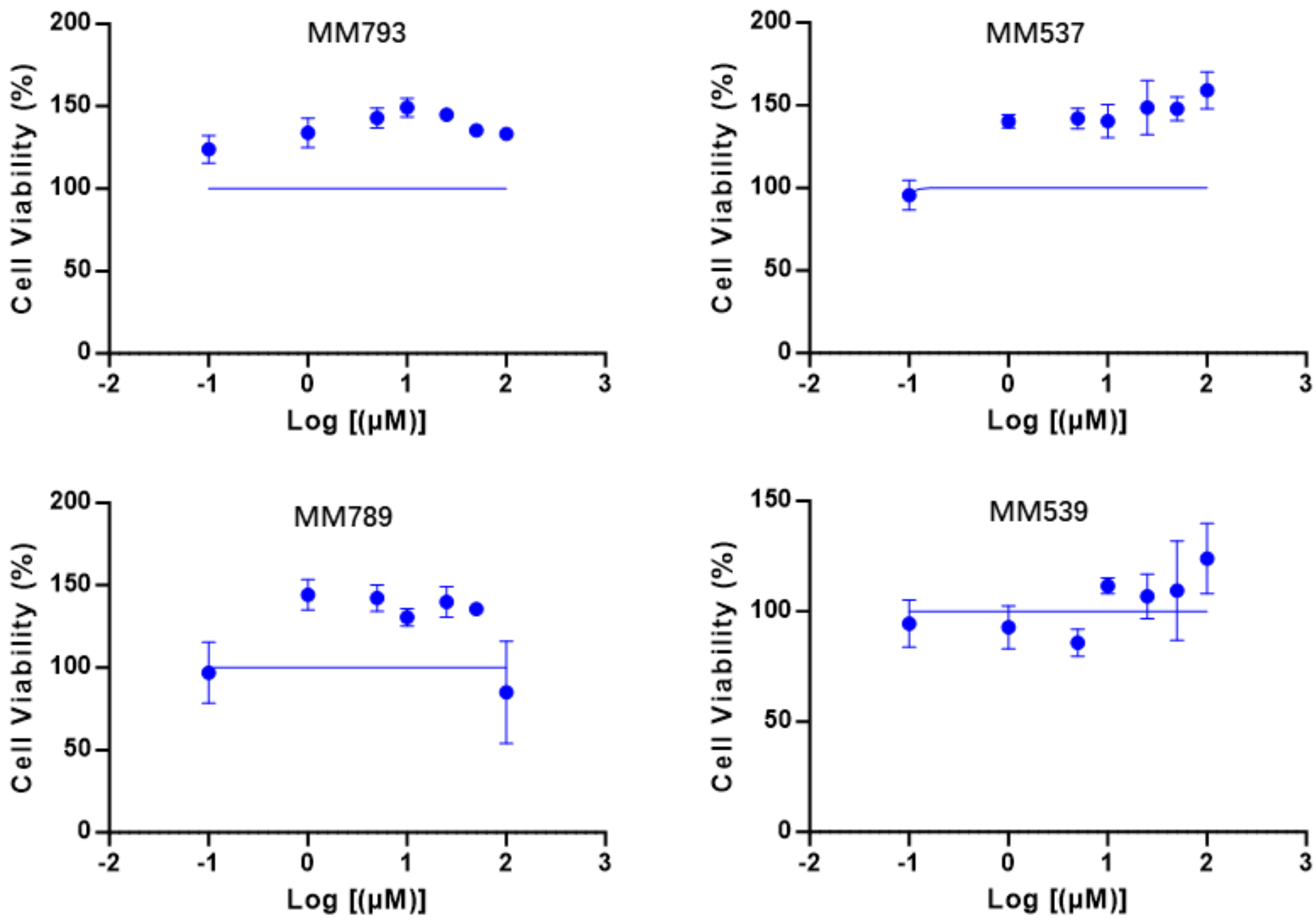

Figure S2. Dose-dependent cell viability curve obtained for compounds **3** (**MM793)**, **16** (**MM537)**, **31** (**MM789)** and **36 (MM539)** in the AlamarBlue assay.

S5. Analysis of SARS-CoV-2 spike protein-driven entry into cells and its inhibition

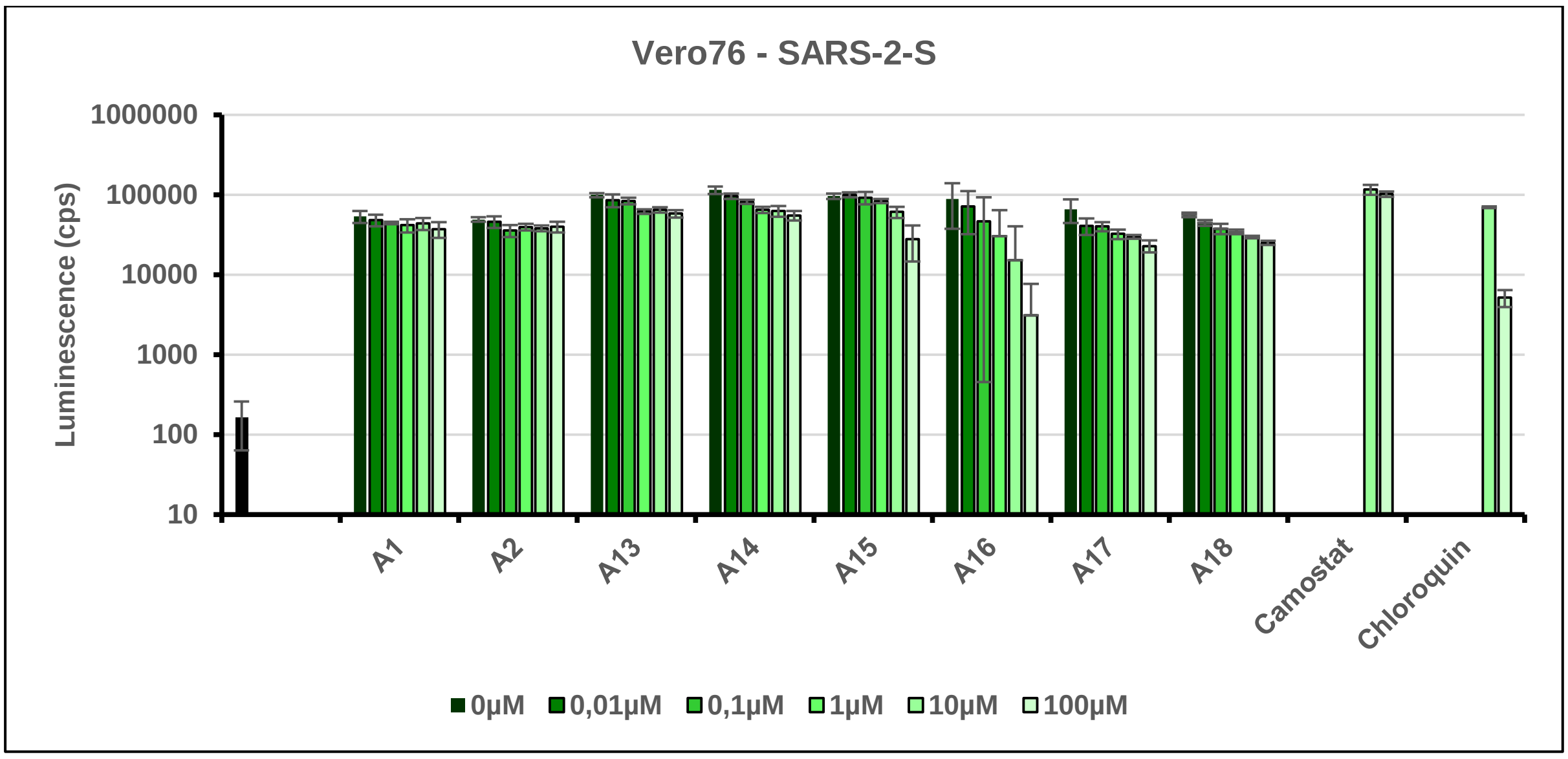

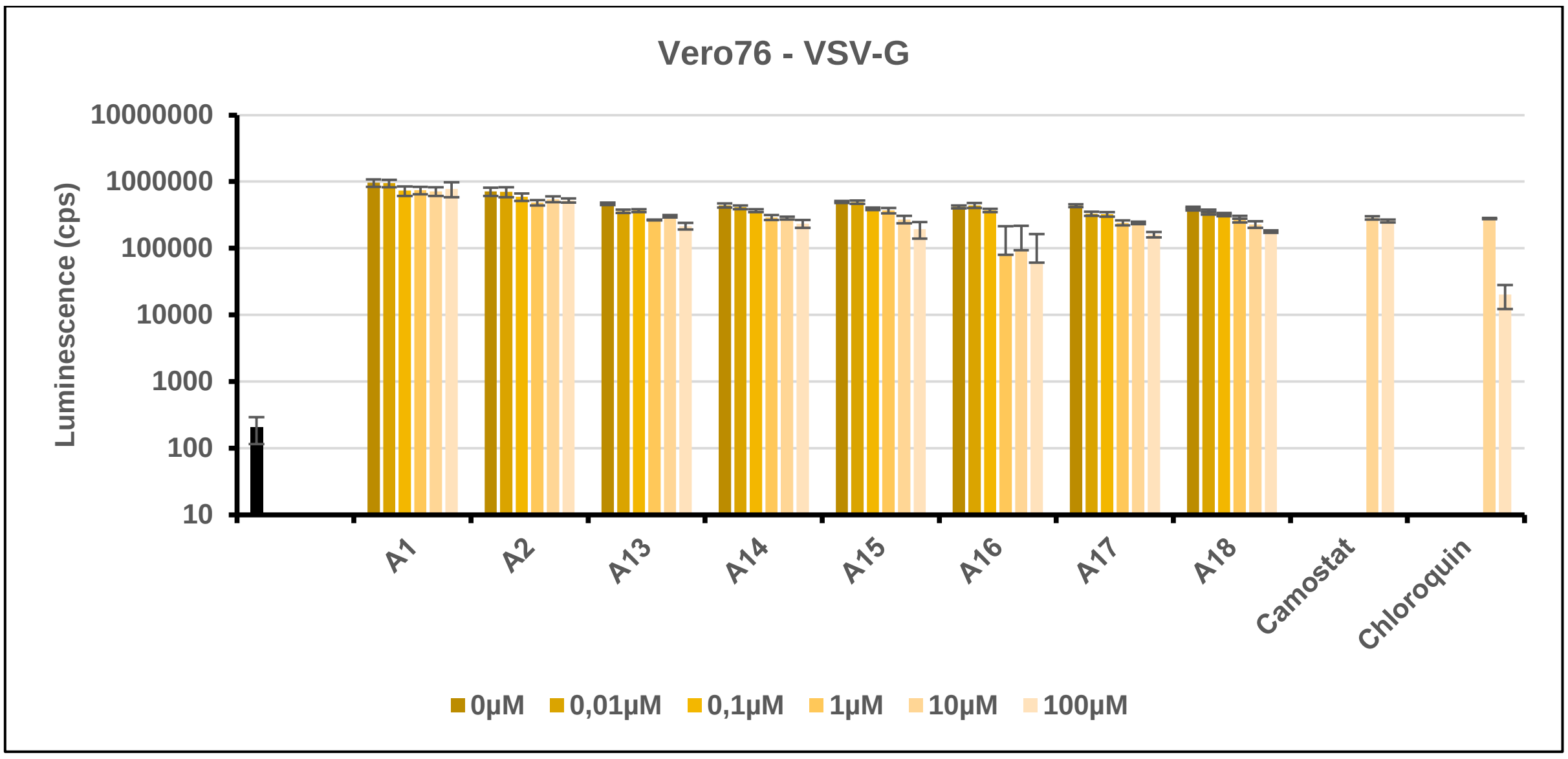

Figure S3. Vero76 cells seeded in 96-well plates were incubated with the indicated compounds at the indicated concentrations and inoculated with vesicular stomatitis virus (VSV) pseudoparticles bearing SARS-CoV-2 S protein (SARS-2-S, top panel) or the VSV glycoprotein (VSV-G, bottom panel) or control inoculated with medium (black bar). Luciferase activity in cell lysates was determined at 16-18 h post infection. The results of a single experiment carried out with technical quadruplicates are shown. Error bars indicate SD. Compounds **A1** (**10**), **A2** (**12**), **A13** (**9**), **A14** (**11**), **A15** (**13**), **A16** (**6**) **A17** (**5**) and **A18** (**7**) jointly with camostat and chloroquine were analyzed in separate experiments.

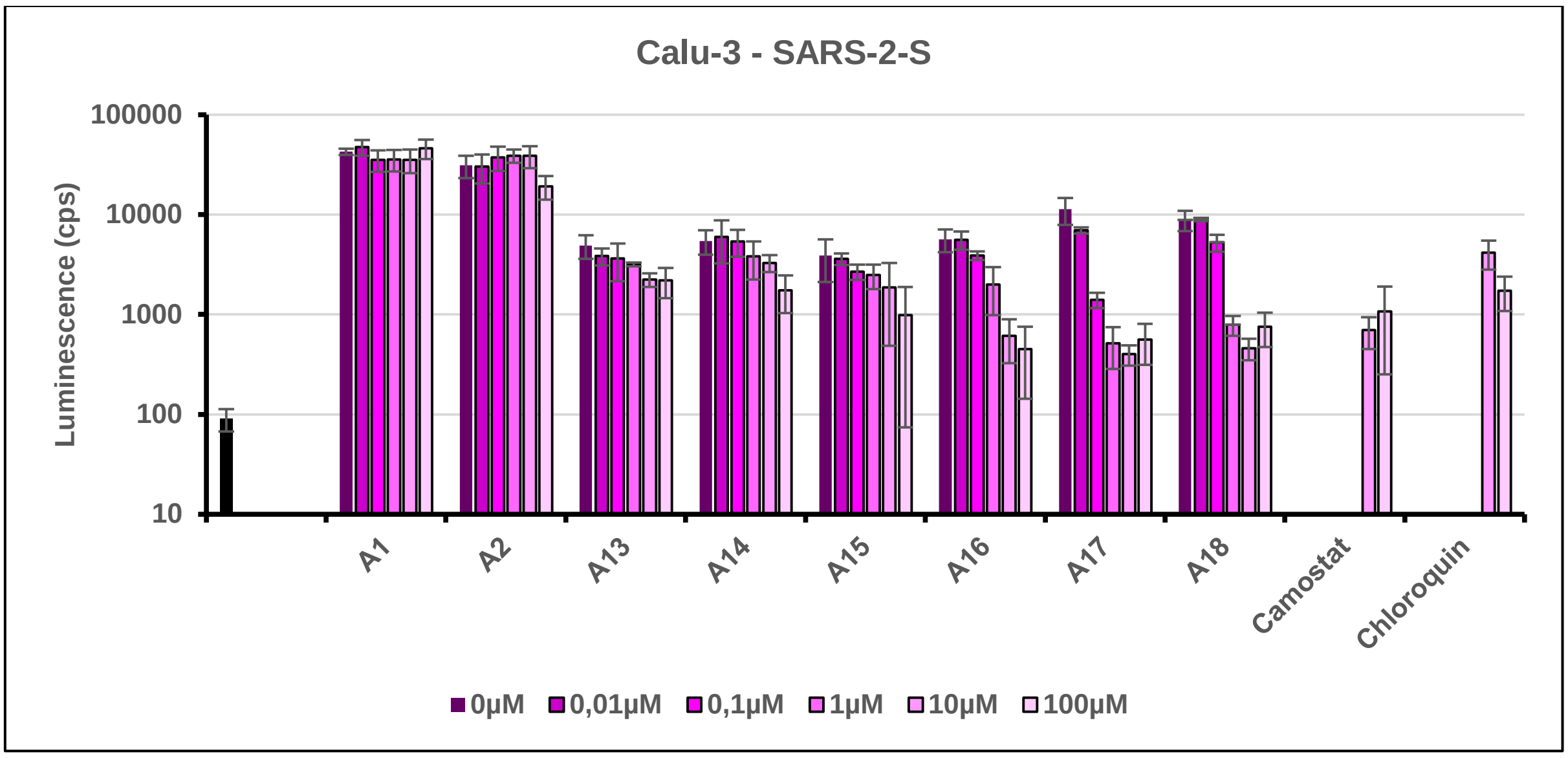

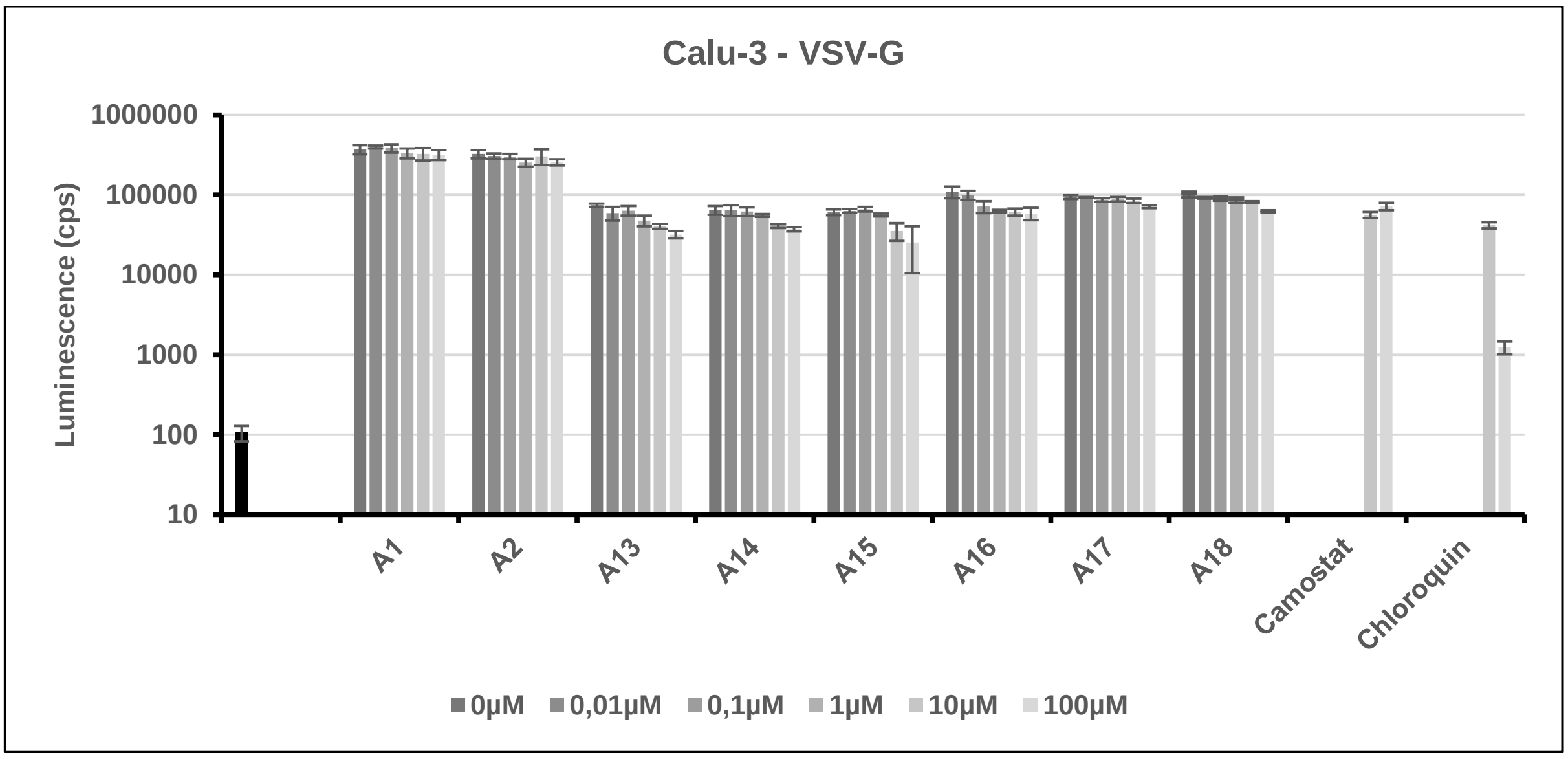

Figure S4. Calu-3 cells seeded in 96-well plates were incubated with the indicated compounds at the indicated concentrations and inoculated with vesicular stomatitis virus (VSV) pseudoparticles bearing SARS-CoV-2 S protein (SARS-2-S, top panel) or the VSV glycoprotein (VSV-G, bottom panel) or control inoculated with medium (black bar). Luciferase activity in cell lysates was determined at 16-18 h post infection. The results of a single experiment carried out with technical quadruplicates are shown. Error bars indicate SD. Compounds **A1** (**10**), **A2** (**12**), **A13** (**9**), **A14** (**11**), **A15** (**13**), **A16** (**6**) **A17** (**5**) and **A18** (**7**) jointly with camostat and chloroquine were analyzed in separate experiments.

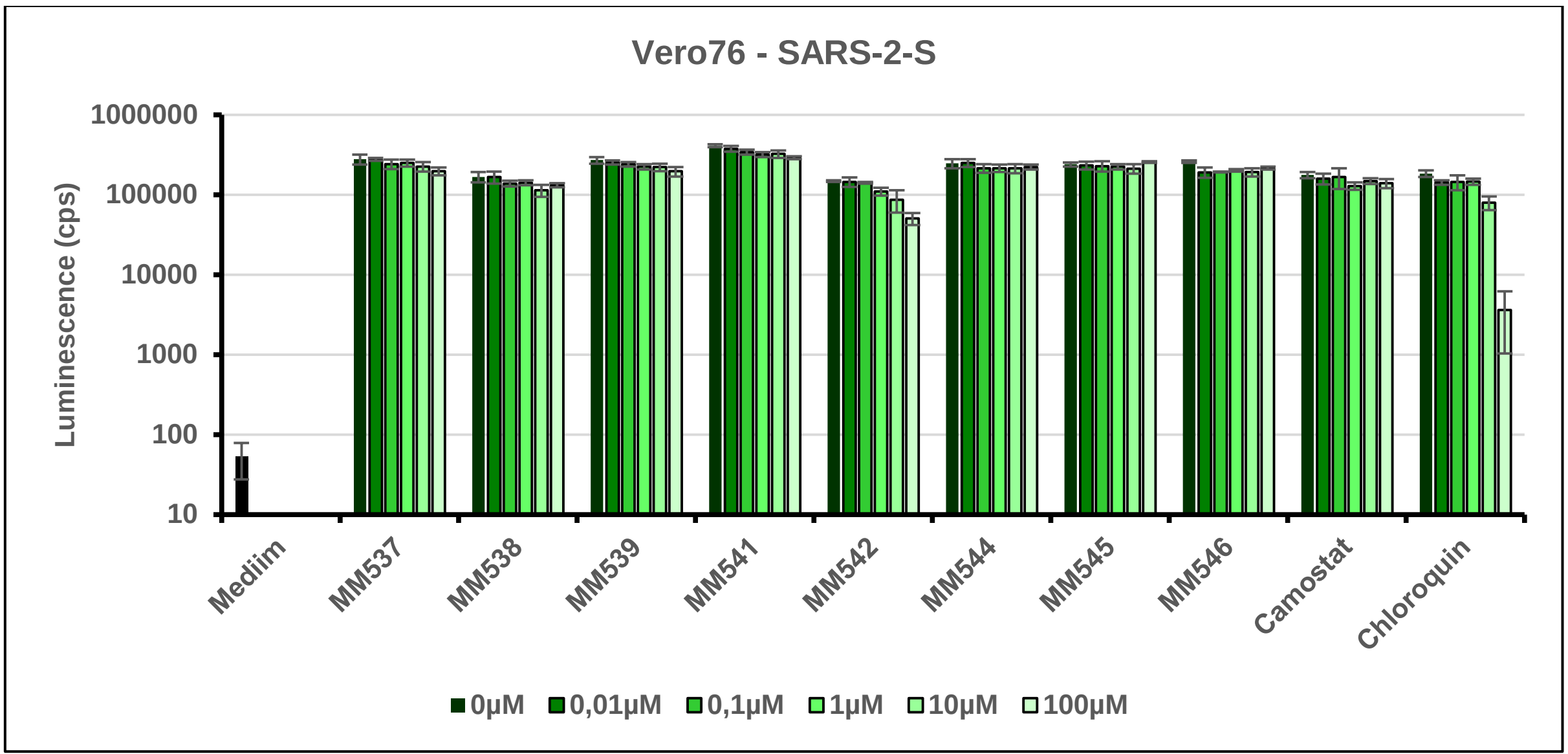

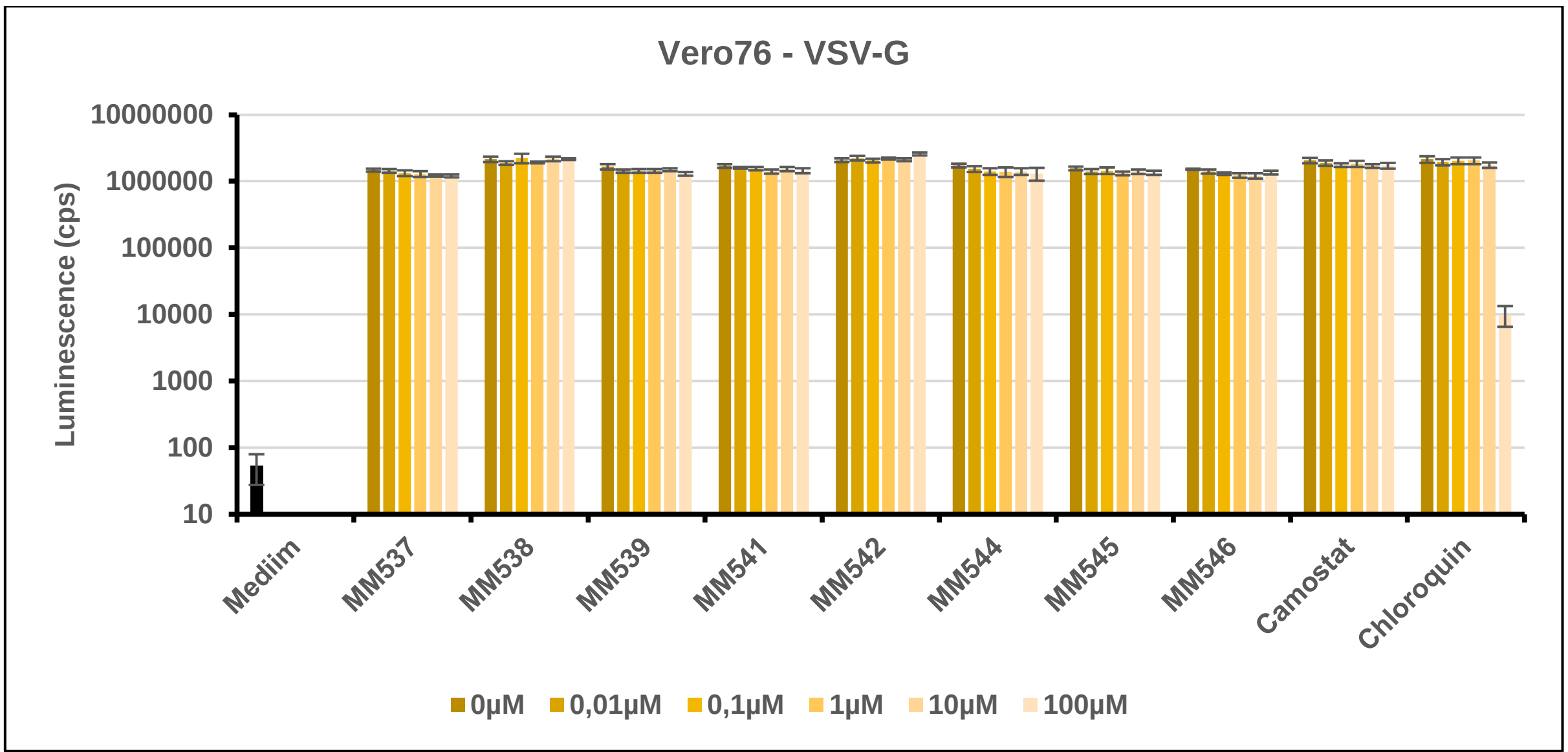

Figure S5. Vero76 cells seeded in 96-well plates were incubated with the indicated compounds at the indicated concentrations and inoculated with vesicular stomatitis virus (VSV) pseudoparticles bearing SARS-CoV-2 S protein (SARS-2-S, top panel) or the VSV glycoprotein (VSV-G, bottom panel) or control inoculated with medium (black bar). Luciferase activity in cell lysates was determined at 16-18 h post infection. The results of a single experiment carried out with technical quadruplicates are shown. Error bars indicate SD. Compounds **MM537** (**16**), **MM538** (**8**), **MM539** (**36**), **MM541** (**21**), **MM542** (**38**), **MM544** (**19**), **MM545** (**41**) and **MM546** (**37**) jointly with camostat and chloroquine were analyzed in separate experiments.

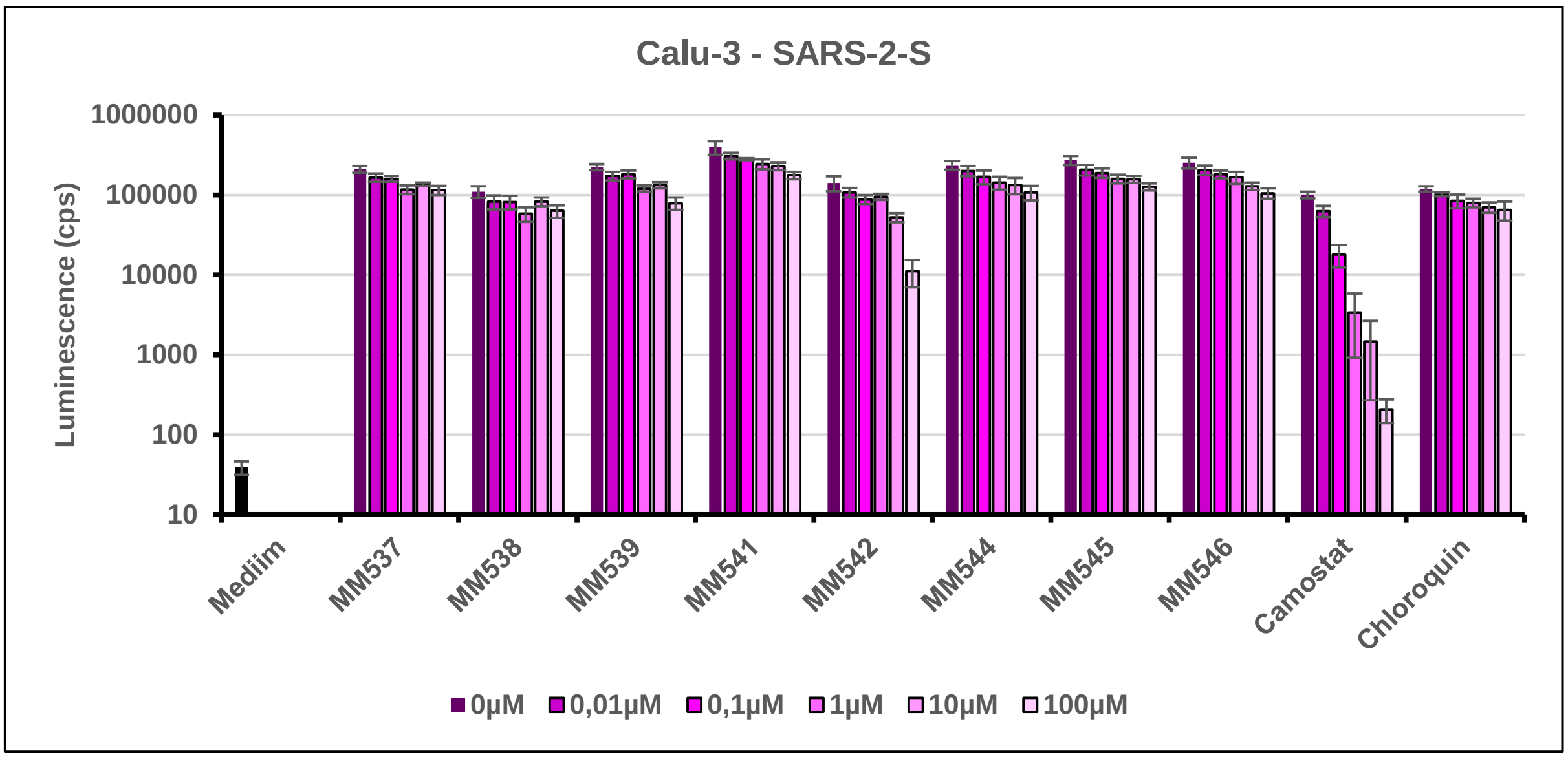

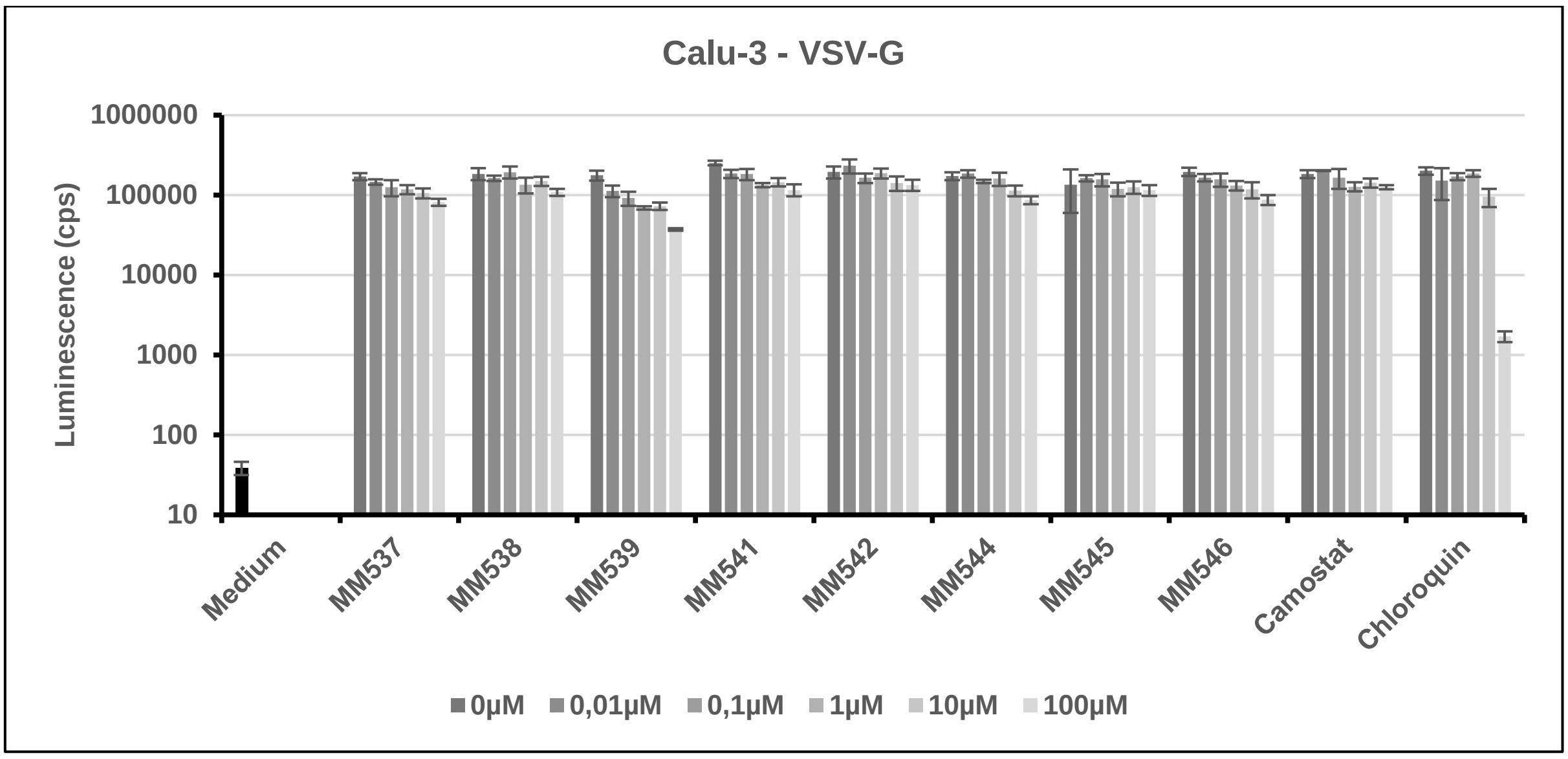

Figure S6. Calu-3 cells seeded in 96-well plates were incubated with the indicated compounds at the indicated concentrations and inoculated with vesicular stomatitis virus (VSV) pseudoparticles bearing SARS-CoV-2 S protein (SARS-2-S, top panel) or the VSV glycoprotein (VSV-G, bottom panel) or control inoculated with medium (black bar). Luciferase activity in cell lysates was determined at 16-18 h post infection. The results of a single experiment carried out with technical quadruplicates are shown. Error bars indicate SD. Compounds **MM537** (**16**), **MM538** (**8**), **MM539** (**36**), **MM541** (**21**), **MM542** (**38**), **MM544** (**19**), **MM545** (**41**) and **MM546** (**37**) jointly with camostat and chloroquine were analyzed in separate experiments.

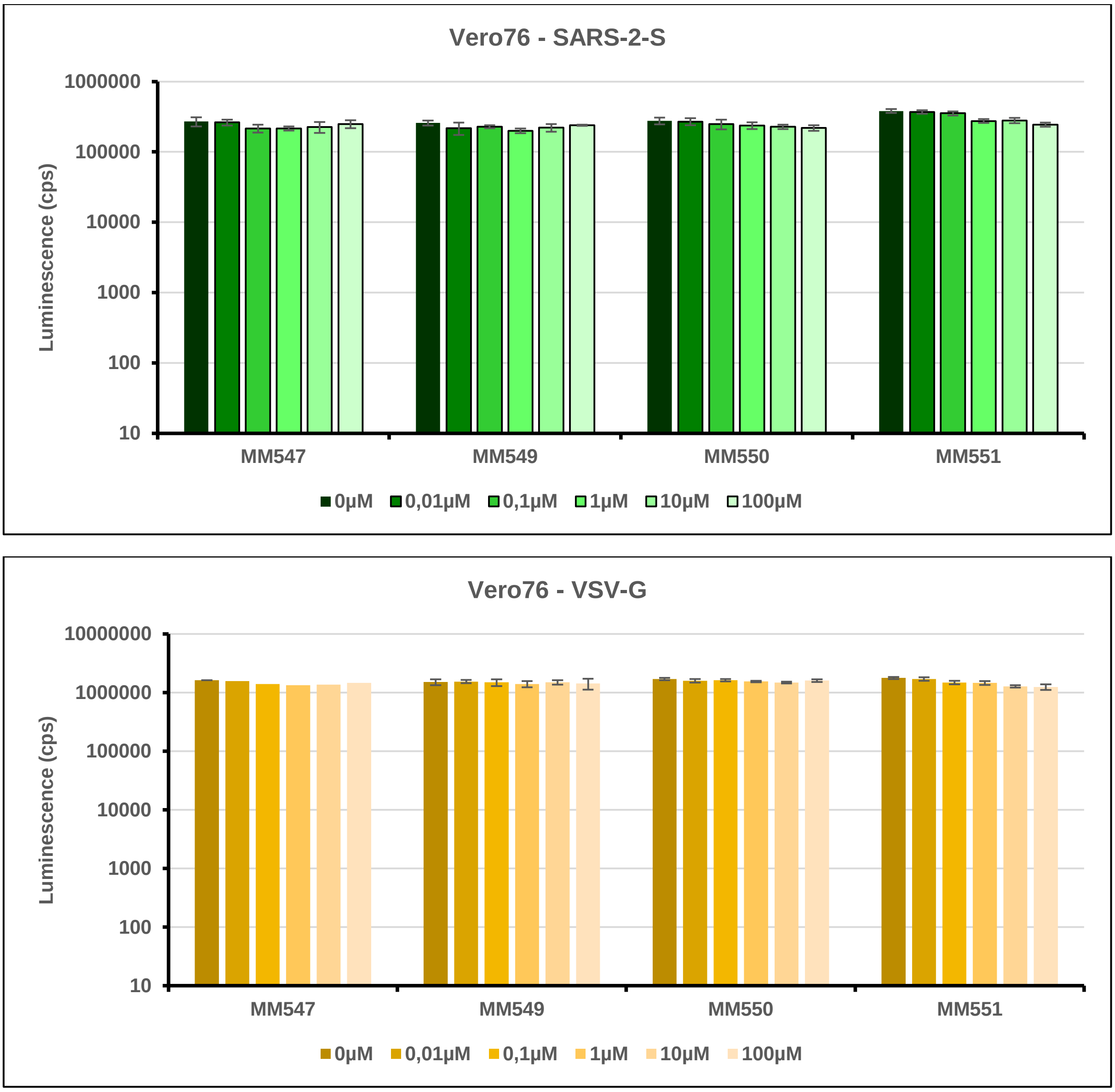

Figure S7. Vero76 cells seeded in 96-well plates were incubated with the indicated compounds at the indicated concentrations and inoculated with vesicular stomatitis virus (VSV) pseudoparticles bearing SARS-CoV-2 S protein (SARS-2-S, top panel) or the VSV glycoprotein (VSV-G, bottom panel) or control inoculated with medium (black bar). Luciferase activity in cell lysates was determined at 16-18 h post infection. The results of a single experiment carried out with technical quadruplicates are shown. Compounds **MM547** (**15**), **MM549** (**18**), **MM550** (**17**) and **MM551** (**40**) were analysed in separate experiments. Error bars indicate SD. Controls are shown in Figure S5.

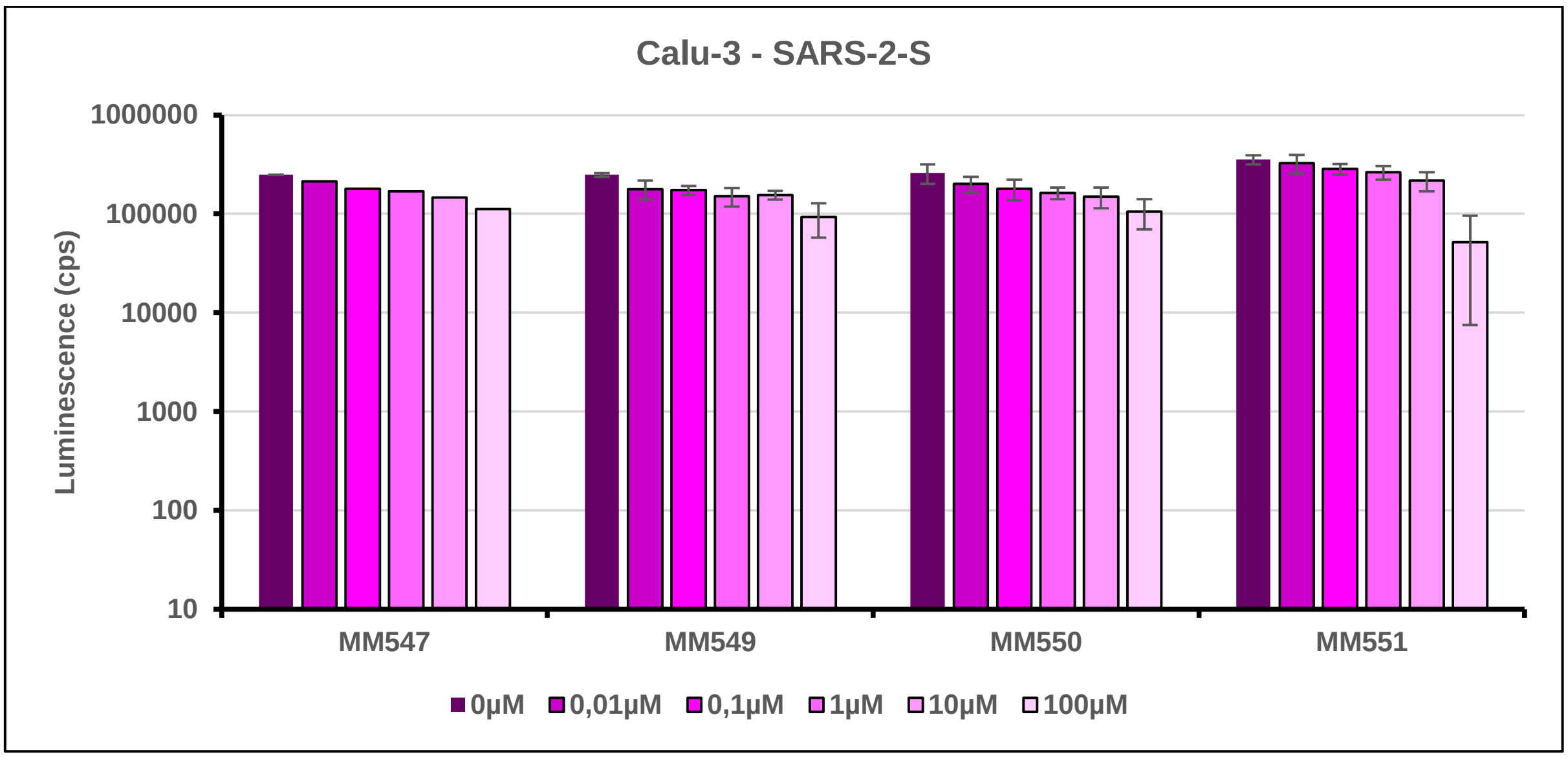

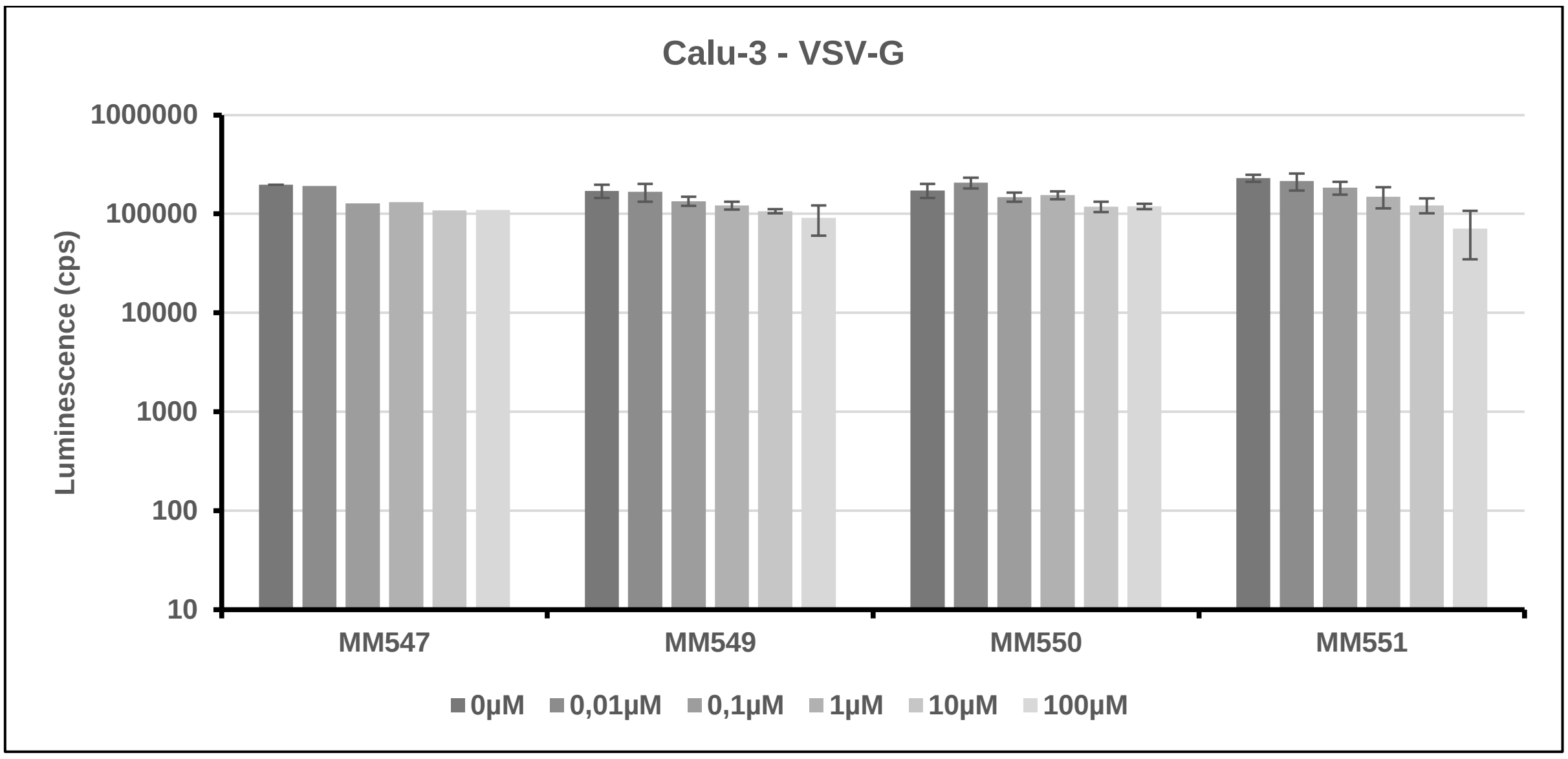

Figure S8. Calu-3 cells seeded in 96-well plates were incubated with the indicated compounds at the indicated concentrations and inoculated with vesicular stomatitis virus (VSV) pseudoparticles bearing SARS-CoV-2 S protein (SARS-2-S, top panel) or the VSV glycoprotein (VSV-G, bottom panel) or control inoculated with medium (black bar). Luciferase activity in cell lysates was determined at 16-18 h post infection. The results of a single experiment carried out with technical quadruplicates are shown. Compounds **MM547** (**15**), **MM549** (**18**), **MM550** (**17**) and **MM551** (**40**) were analysed in separate experiments. Error bars indicate SD. Controls are shown in Figure S6.

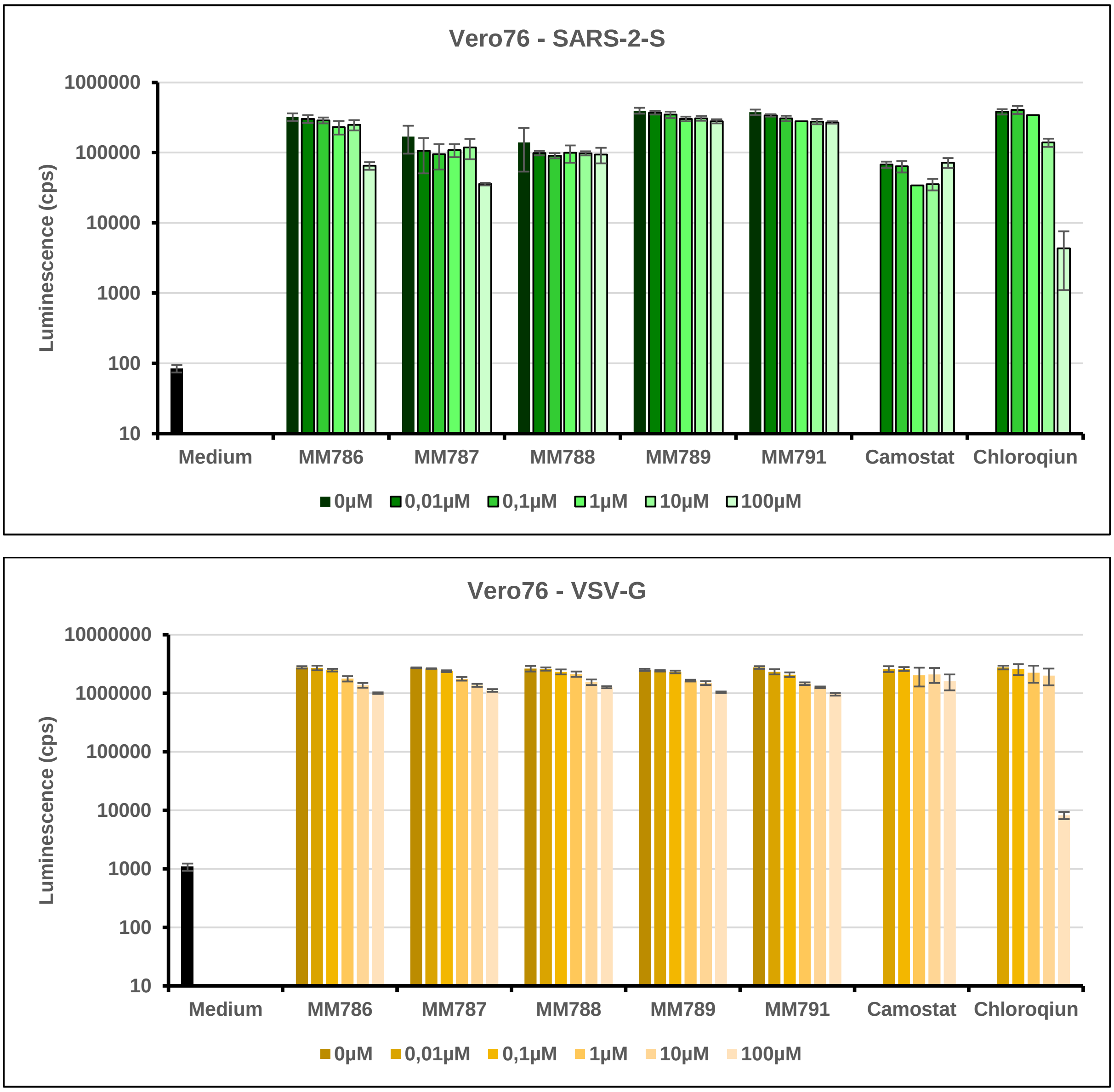

Figure S9. Vero76 cells seeded in 96-well plates were incubated with the indicated compounds at the indicated concentrations and inoculated with vesicular stomatitis virus (VSV) pseudoparticles bearing SARS-CoV-2 S protein (SARS-2-S, top panel) or the VSV glycoprotein (VSV-G, bottom panel) or control inoculated with medium (black bar). Luciferase activity in cell lysates was determined at 16-18 h post infection. The results of a single experiment carried out with technical quadruplicates are shown. Compounds **MM786** (**39**), **MM787** (**34**), **MM788** (**35**), **MM789** (**31**) and **MM791** (**14**) were analysed in separate experiments. Error bars indicate SD.

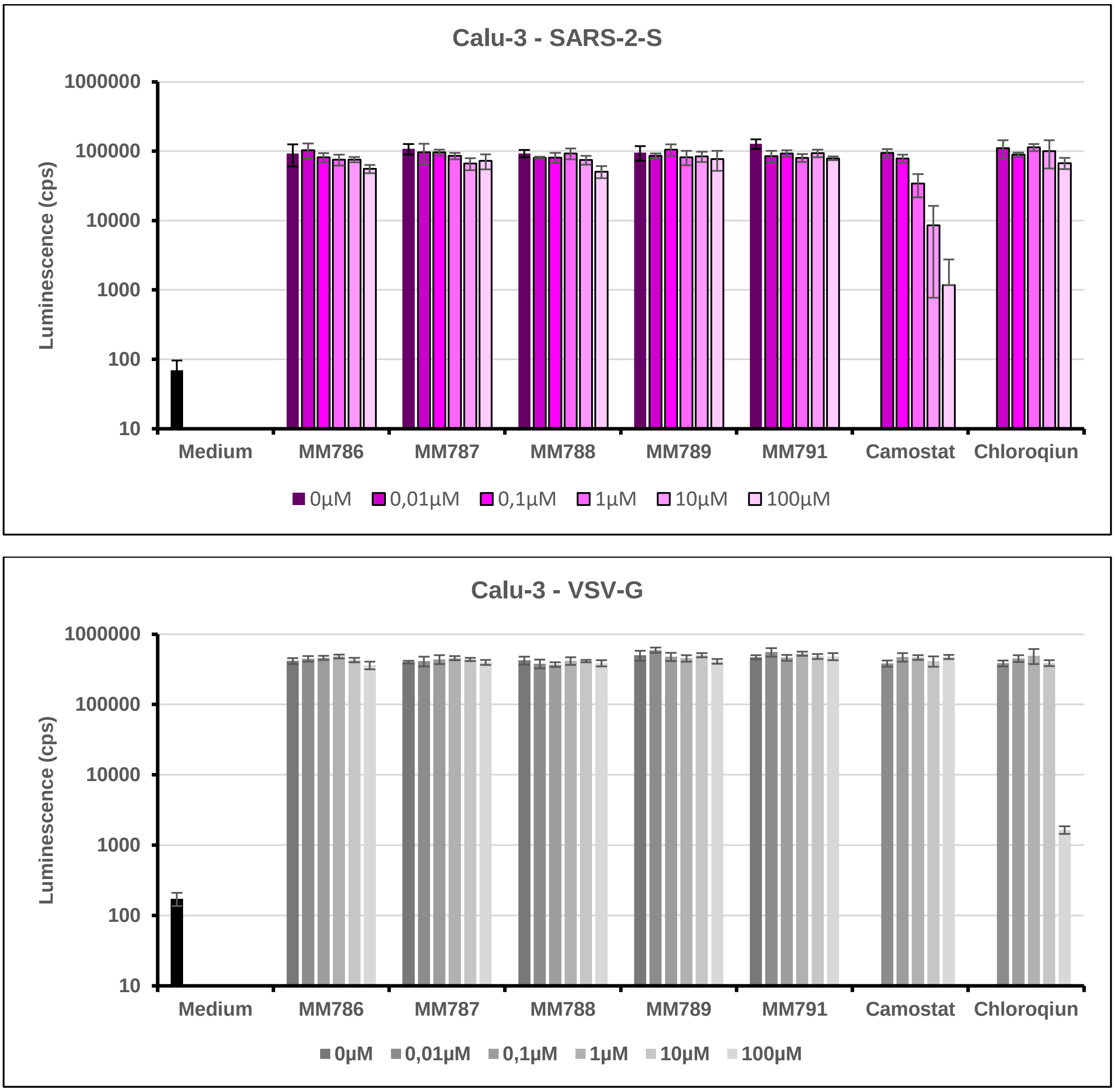

Figure S10. Calu-3 cells seeded in 96-well plates were incubated with the indicated compounds at the indicated concentrations and inoculated with vesicular stomatitis virus (VSV) pseudoparticles bearing SARS-CoV-2 S protein (SARS-2-S, top panel) or the VSV glycoprotein (VSV-G, bottom panel) or control inoculated with medium (black bar). Luciferase activity in cell lysates was determined at 16-18 h post infection. The results of a single experiment carried out with technical quadruplicates are shown. Compounds **MM786** (**39**), **MM787** (**34**), **MM788** (**35**), **MM789** (**31**) and **MM791** (**14**) were analysed in separate experiments. Error bars indicate SD.

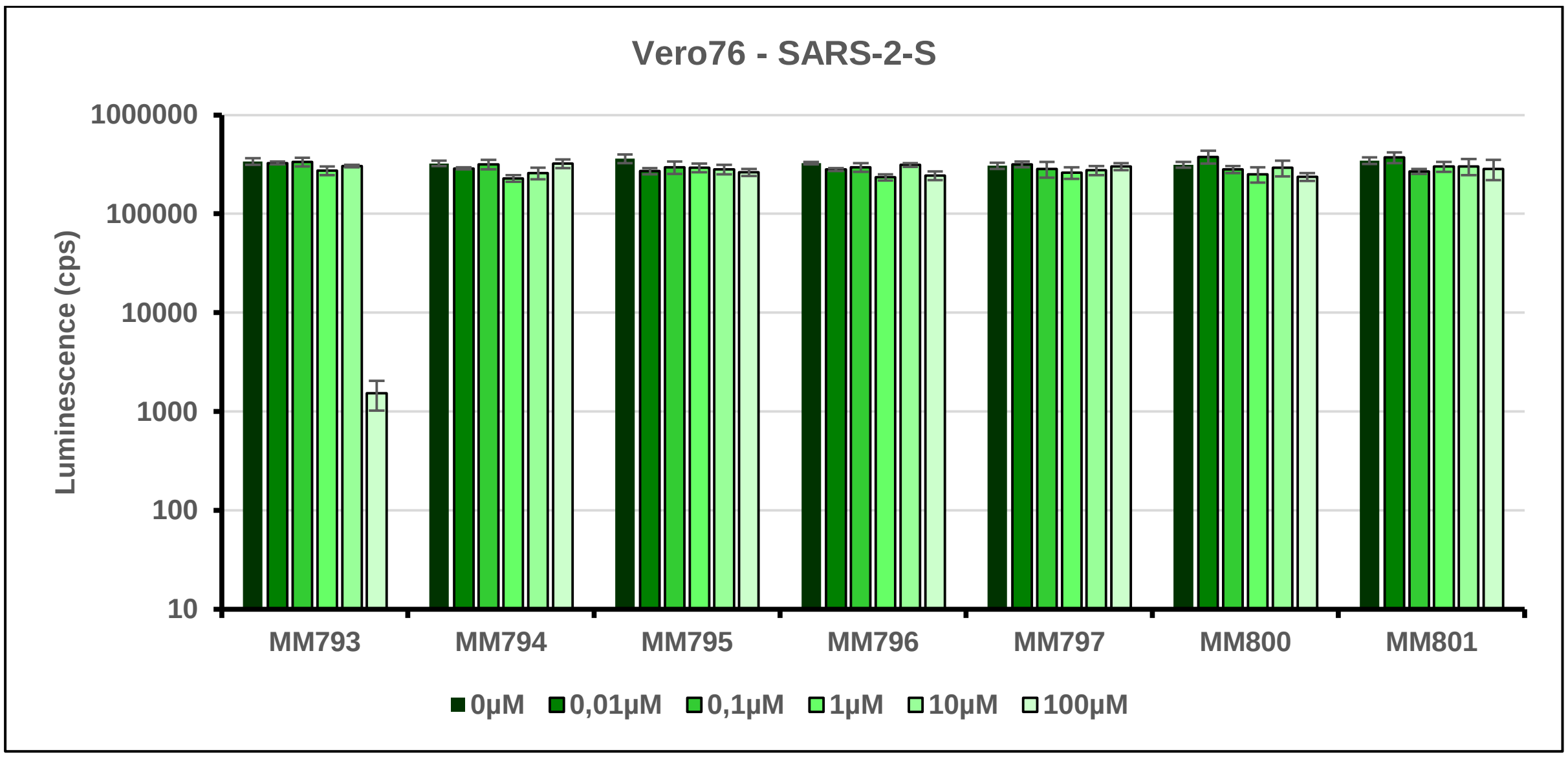

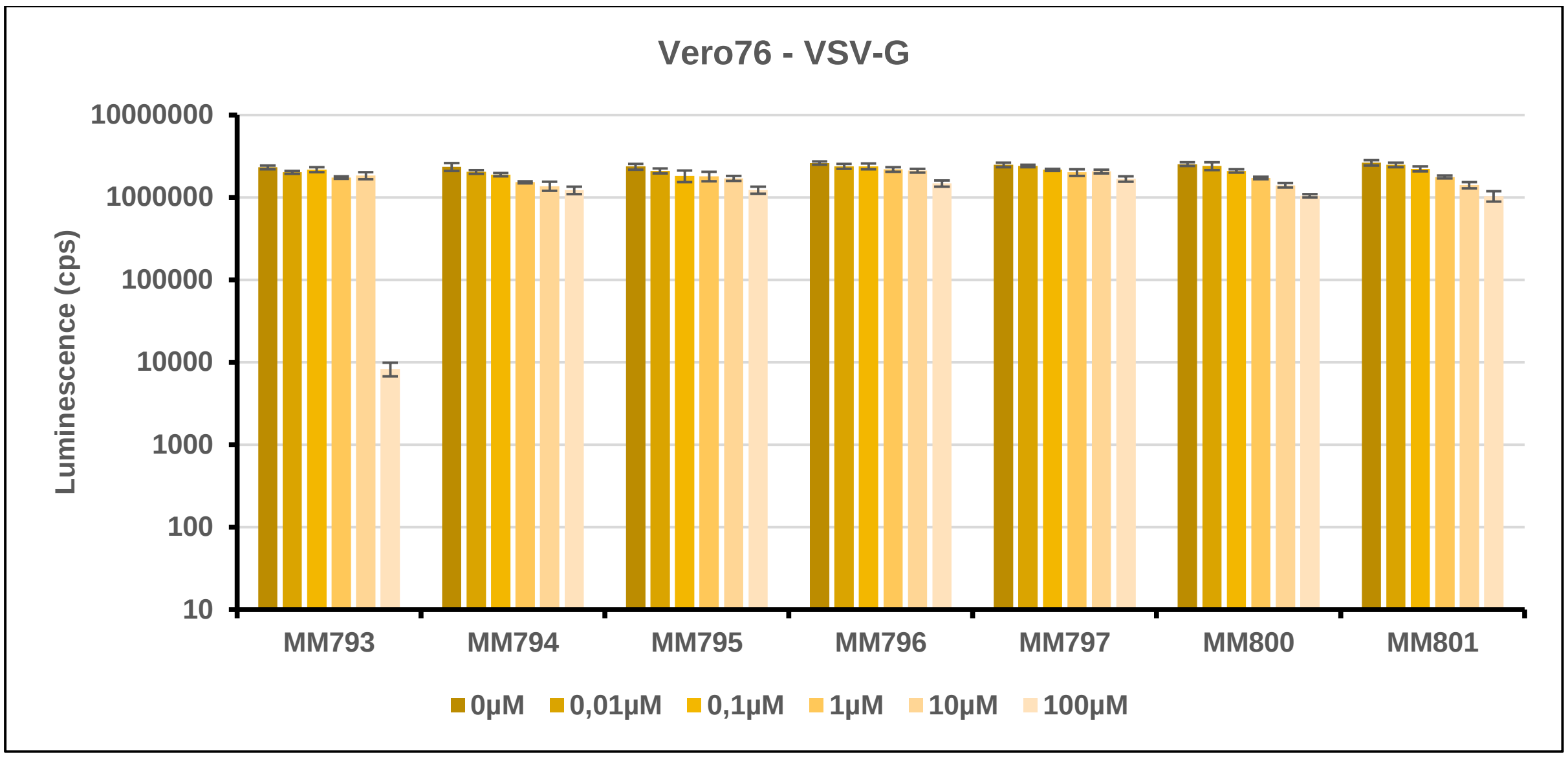

Figure S11. Vero76 cells seeded in 96-well plates were incubated with the indicated compounds at the indicated concentrations and inoculated with vesicular stomatitis virus (VSV) pseudoparticles bearing SARS-CoV-2 S protein (SARS-2-S, top panel) or the VSV glycoprotein (VSV-G, bottom panel) or control inoculated with medium (black bar). Luciferase activity in cell lysates was determined at 16-18 h post infection. The results of a single experiment carried out with technical quadruplicates are shown. Compounds **MM793** (**3**), **MM794** (**22**), **MM795** (**24**), **MM796** (**1**), **MM797** (**4**), **MM800** (**32**) and **MM801** (**30**) were analysed in separate experiments. Error bars indicate SD. Controls are shown in Figure S9.

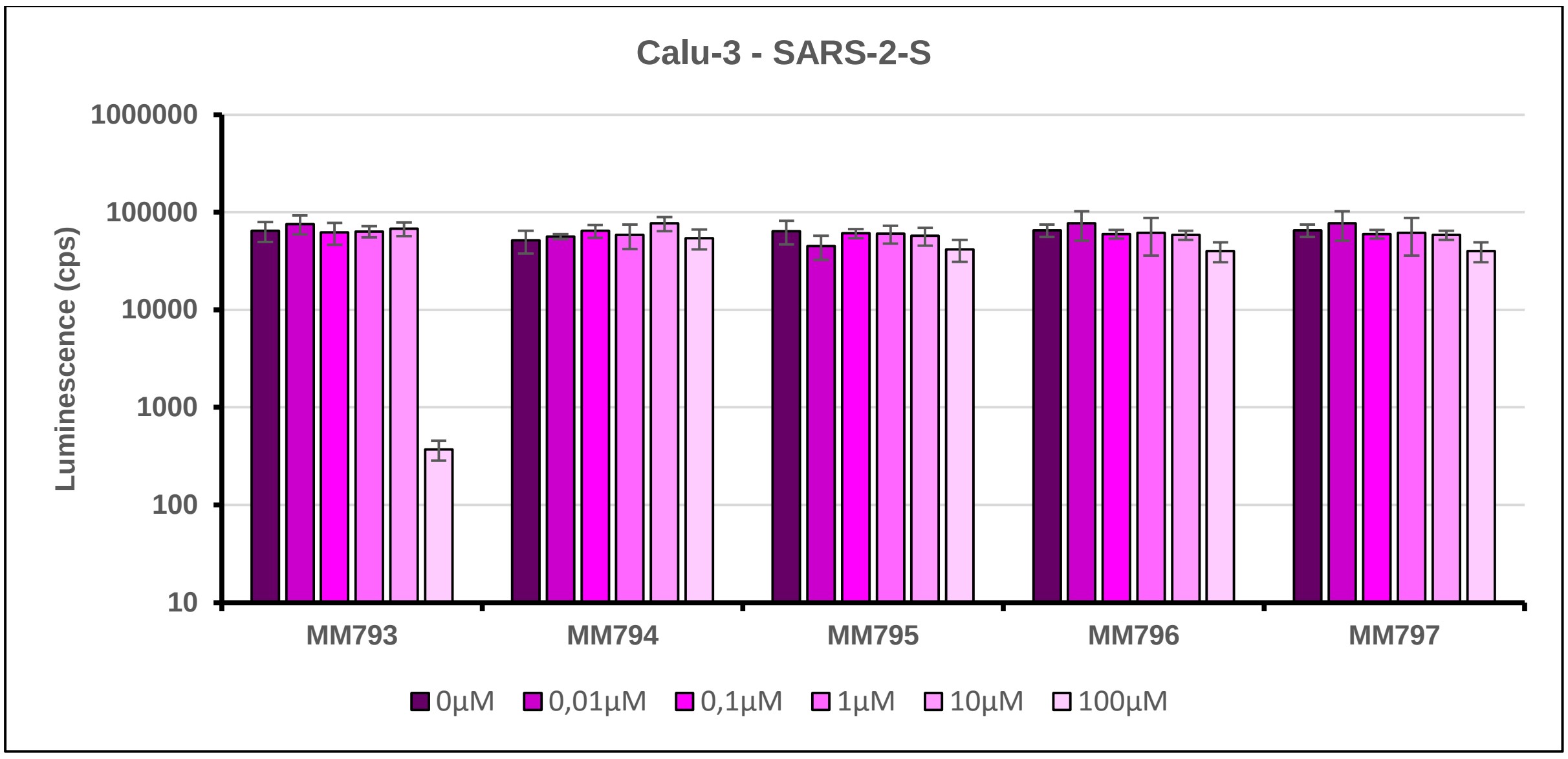

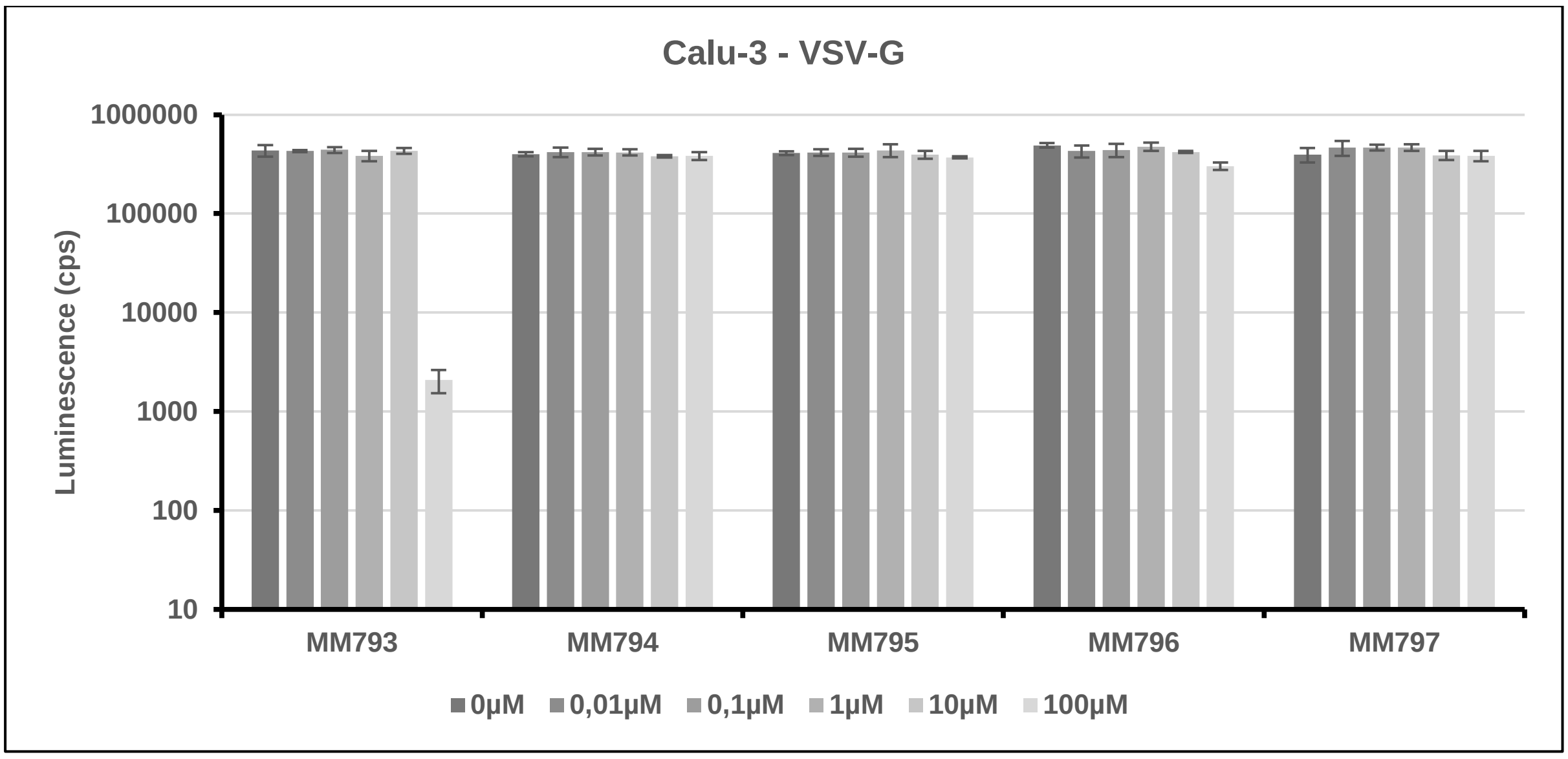

Figure S12. Calu-3 cells seeded in 96-well plates were incubated with the indicated compounds at the indicated concentrations and inoculated with vesicular stomatitis virus (VSV) pseudoparticles bearing SARS-CoV-2 S protein (SARS-2-S, top panel) or the VSV glycoprotein (VSV-G, bottom panel) or control inoculated with medium (black bar). Luciferase activity in cell lysates was determined at 16-18 h post infection. The results of a single experiment carried out with technical quadruplicates are shown. Compounds **MM793** (**3**), **MM794** (**22**), **MM795** (**24**), **MM796** (**1**), and **MM797** (**4**), were analysed in separate experiments. Error bars indicate SD. Controls are shown in Figure S10.

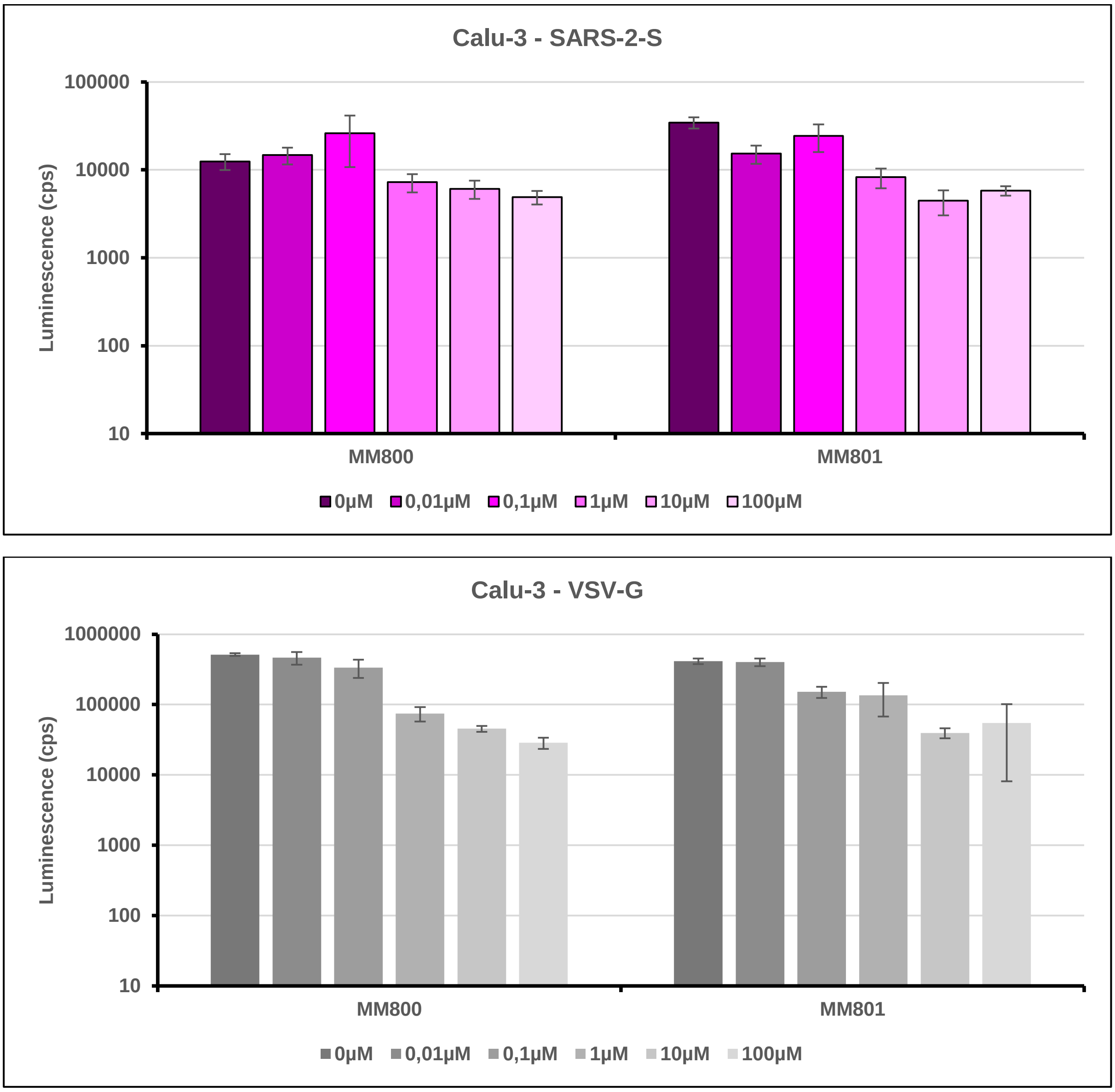

Figure S13. Calu-3 cells seeded in 96-well plates were incubated with the indicated compounds at the indicated concentrations and inoculated with vesicular stomatitis virus (VSV) pseudoparticles bearing SARS-CoV-2 S protein (SARS-2-S, top panel) or the VSV glycoprotein (VSV-G, bottom panel) or control inoculated with medium (black bar). Luciferase activity in cell lysates was determined at 16-18 h post infection. The results of a single experiment carried out with technical quadruplicates are shown. Error bars indicate SD. Compounds **MM800** (**32**) and **MM801** (**30**) were analysed in separate experiments. Controls are shown in Figure S10.

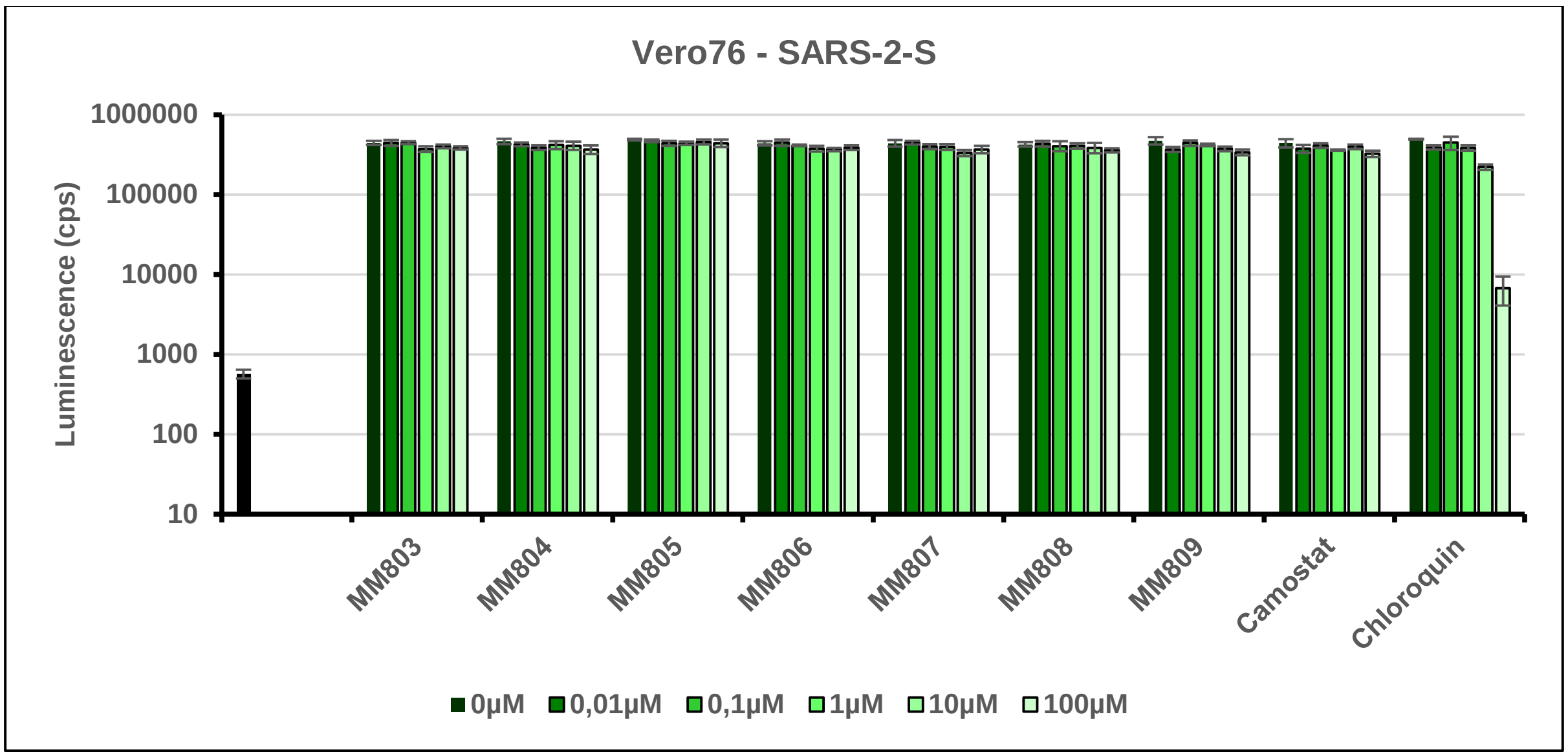

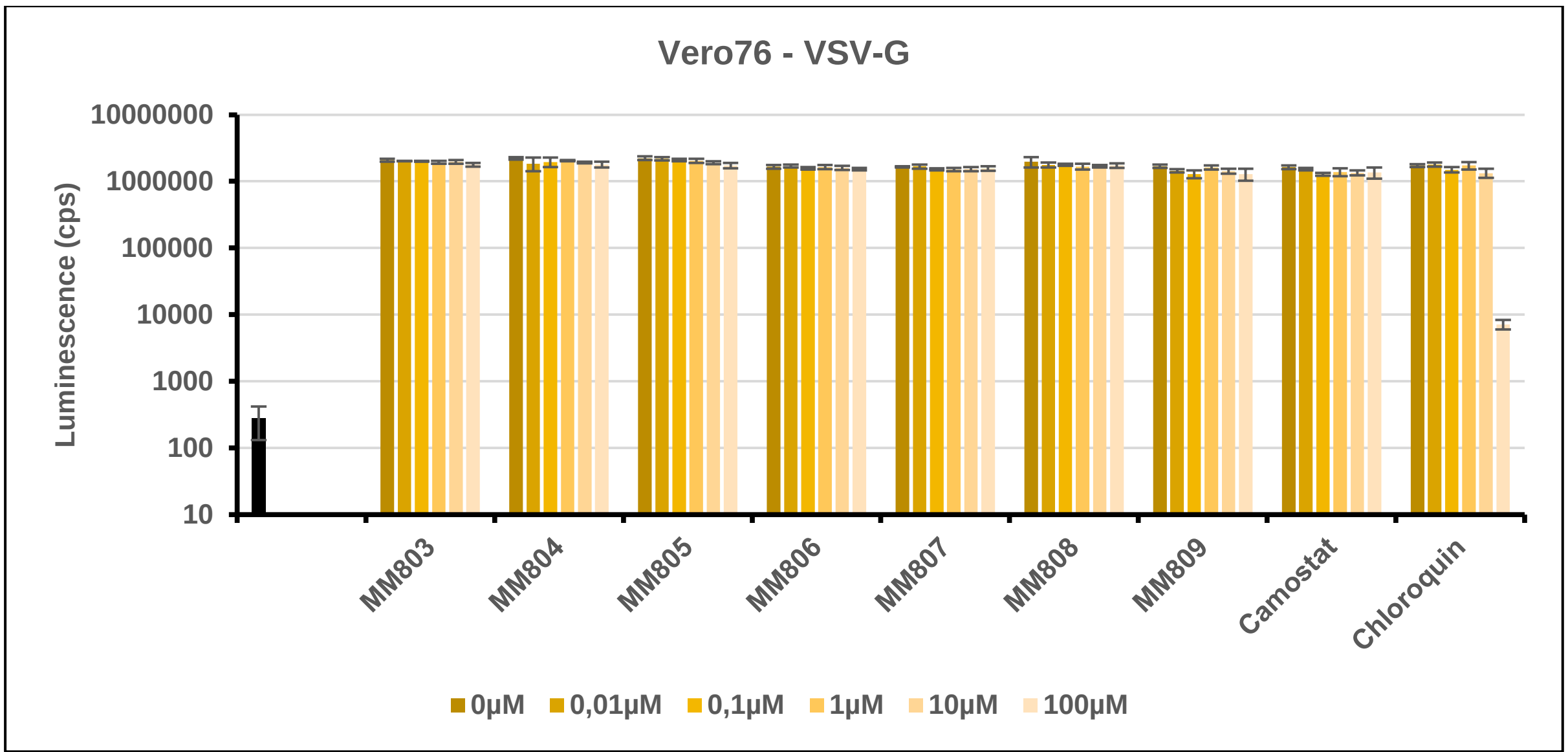

Figure S14. Vero76 cells seeded in 96-well plates were incubated with the indicated compounds at the indicated concentrations and inoculated with vesicular stomatitis virus (VSV) pseudoparticles bearing SARS-CoV-2 S protein (SARS-2-S, top panel) or the VSV glycoprotein (VSV-G, bottom panel) or control inoculated with medium (black bar). Luciferase activity in cell lysates was determined at 16-18 h post infection. The results of a single experiment carried out with technical quadruplicates

are shown. Compounds **MM803** (**27**), **MM804** (**25**), **MM805** (**23**), **MM806** (**29**), **MM807** (**26**), **MM808** (**28**) and **MM809** (**33**) were analysed in separate experiments. Error bars indicate SD.

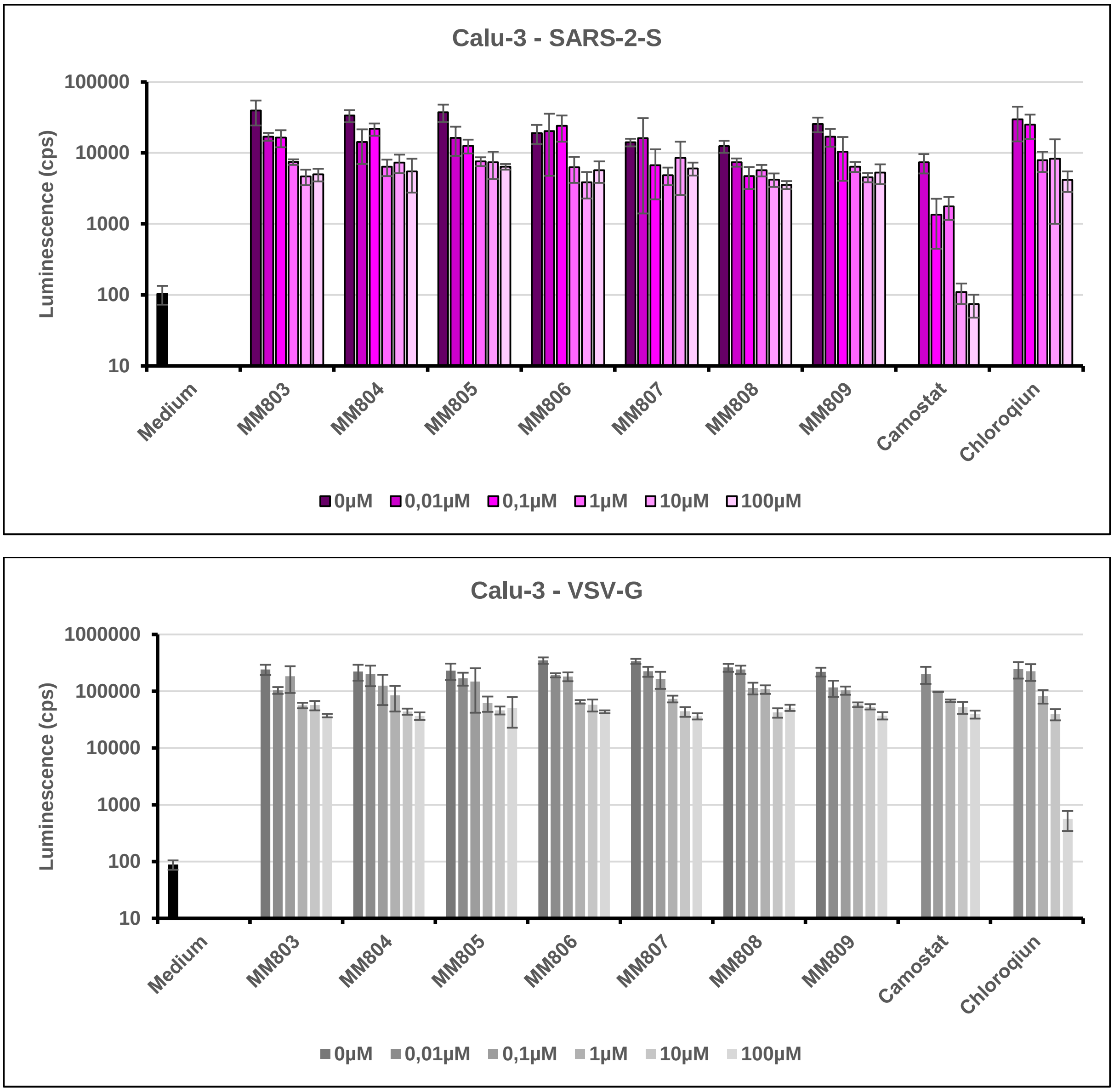

Figure S15. Calu-3 cells seeded in 96-well plates were incubated with the indicated compounds at the indicated concentrations and inoculated with vesicular stomatitis virus (VSV) pseudoparticles bearing SARS-CoV-2 S protein (SARS-2-S, top panel) or the VSV glycoprotein (VSV-G, bottom panel) or control inoculated with medium (black bar). Luciferase activity in cell lysates was determined at 16-18 h post infection. The results of a single experiment carried out with technical quadruplicates

are shown. Compounds **MM803** (**27**), **MM804** (**25**), **MM805** (**23**), **MM806** (**29**), **MM807** (**26**), **MM808** (**28**) and **MM809** (**33**) were analysed in separate experiments. Error bars indicate SD.

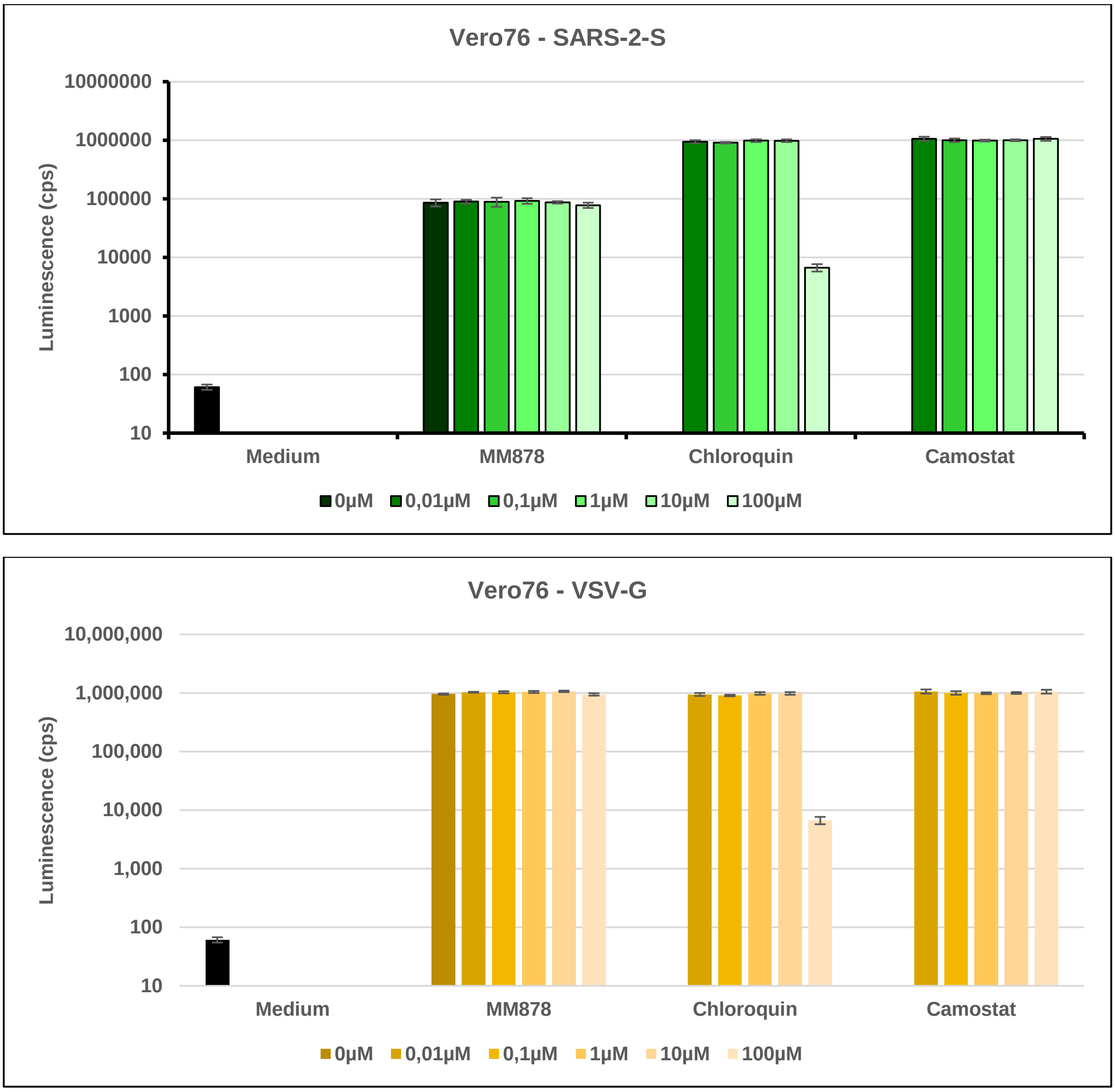

Figure S16. Vero76 cells seeded in 96-well plates were incubated with the indicated compounds at the indicated concentrations and inoculated with vesicular stomatitis virus (VSV) pseudoparticles bearing SARS-CoV-2 S protein (SARS-2-S, top panel) or the VSV glycoprotein (VSV-G, bottom panel)

or control inoculated with medium (black bar). Luciferase activity in cell lysates was determined at 16-18 h post infection. The results of a single experiment carried out with technical quadruplicates are shown. Compound **MM878** (**20**), as well as chloroquine and camostat were analysed in separate experiments. Error bars indicate SD.

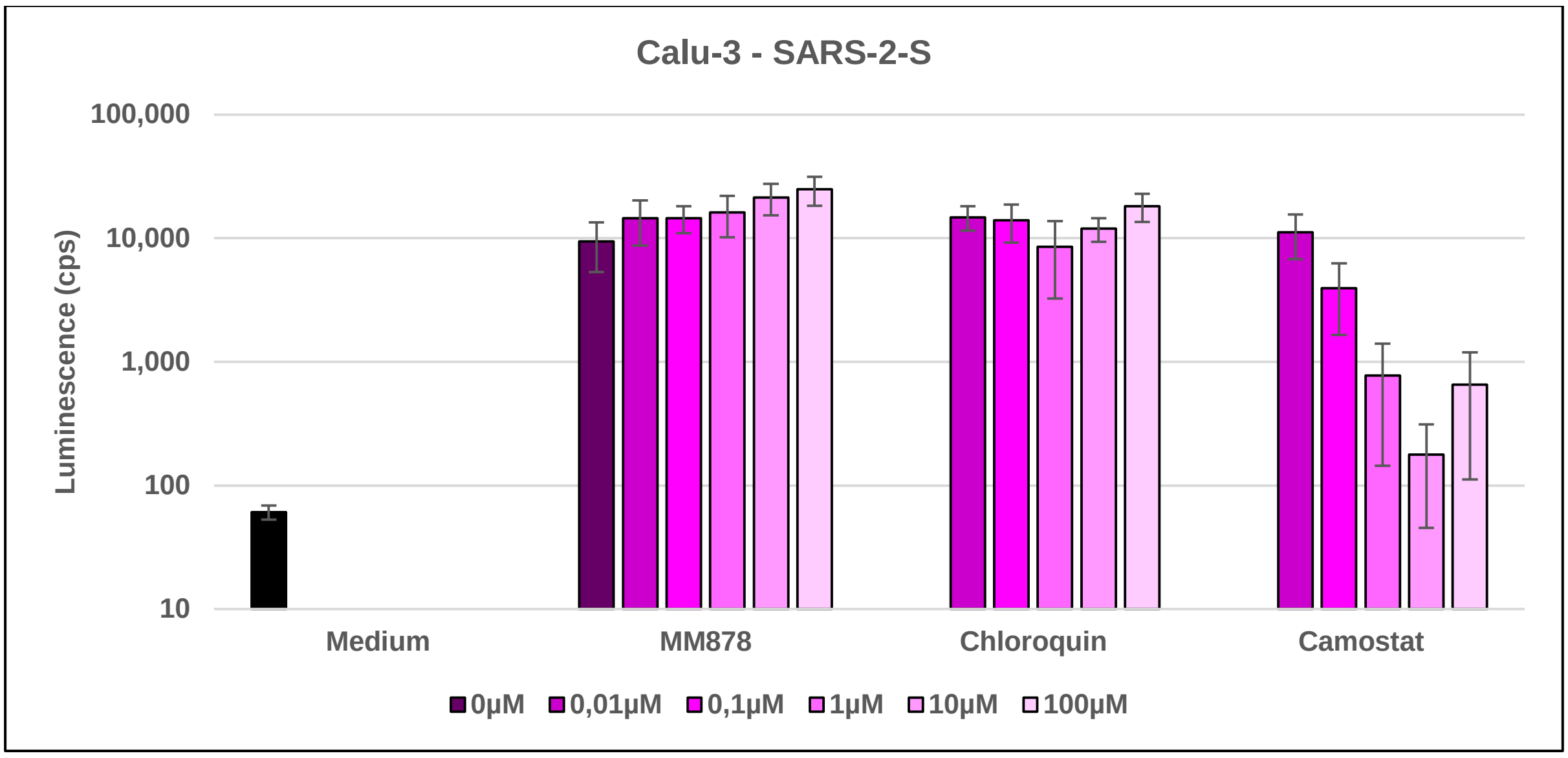

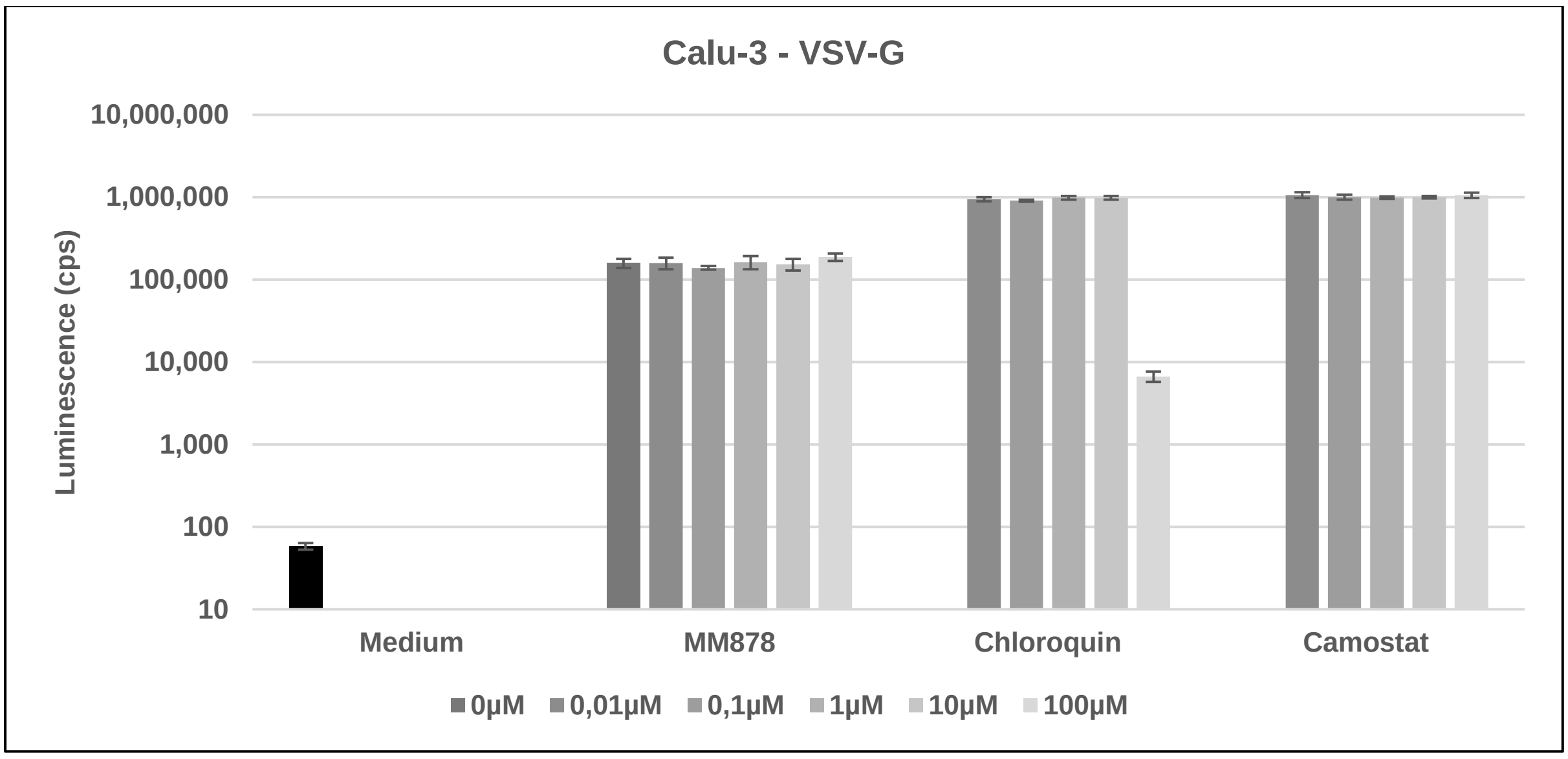

Figure S17. Calu-3 cells seeded in 96-well plates were incubated with the indicated compounds at the indicated concentrations and inoculated with vesicular stomatitis virus (VSV) pseudoparticles bearing SARS-CoV-2 S protein (SARS-2-S, top panel) or the VSV glycoprotein (VSV-G, bottom panel) or control inoculated with medium (black bar). Luciferase activity in cell lysates was determined at 16-18 h post infection. The results of a single experiment carried out with technical quadruplicates are shown. Compound **MM878** (**20**), as well as chloroquine and camostat were analysed in separate experiments. Error bars indicate SD.

S6. ^{1}H and C NMR spectra

Compound **1**

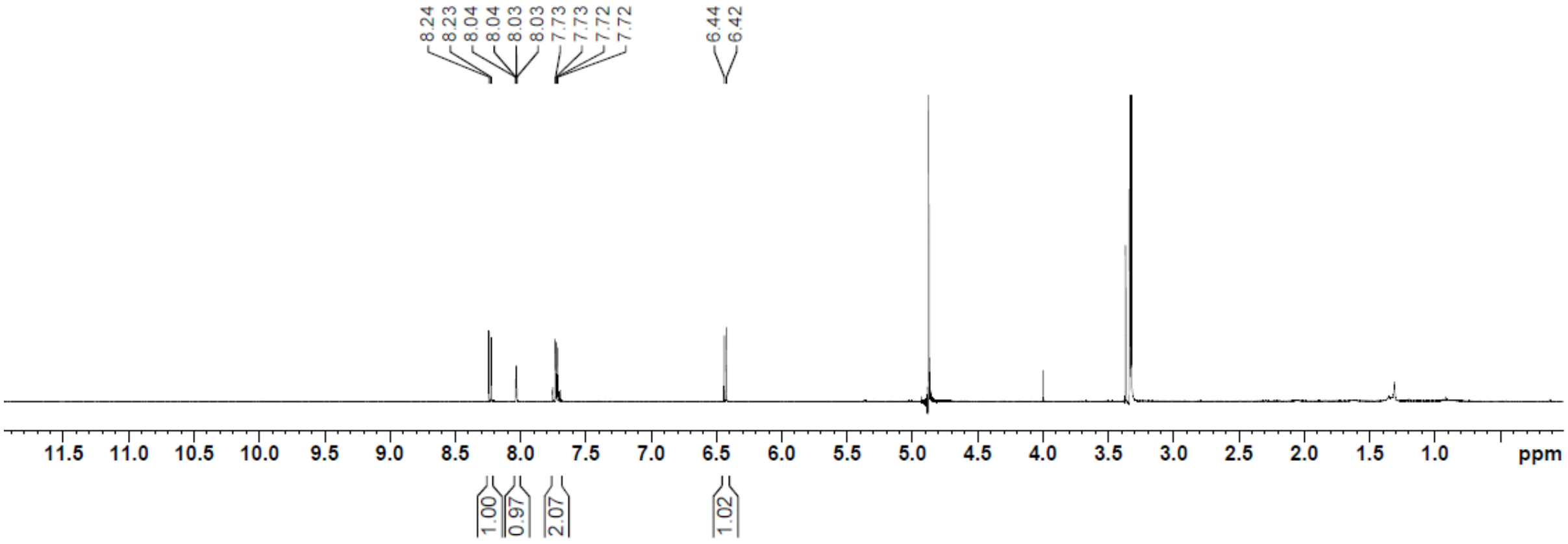

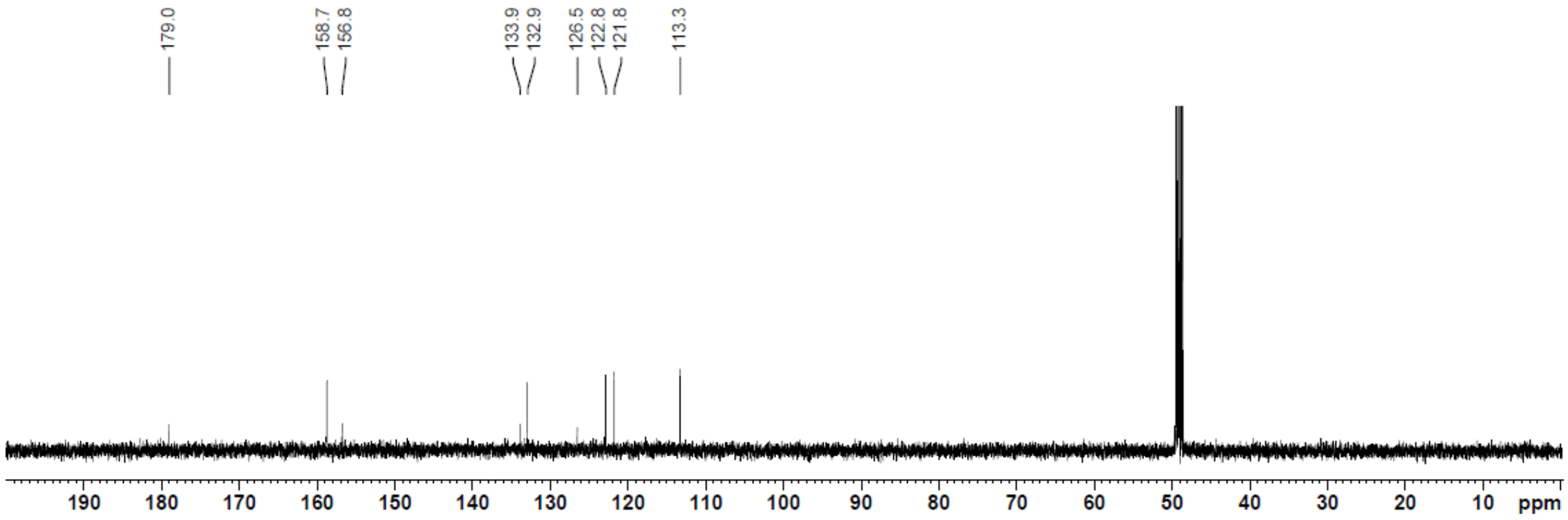

Compound **2**

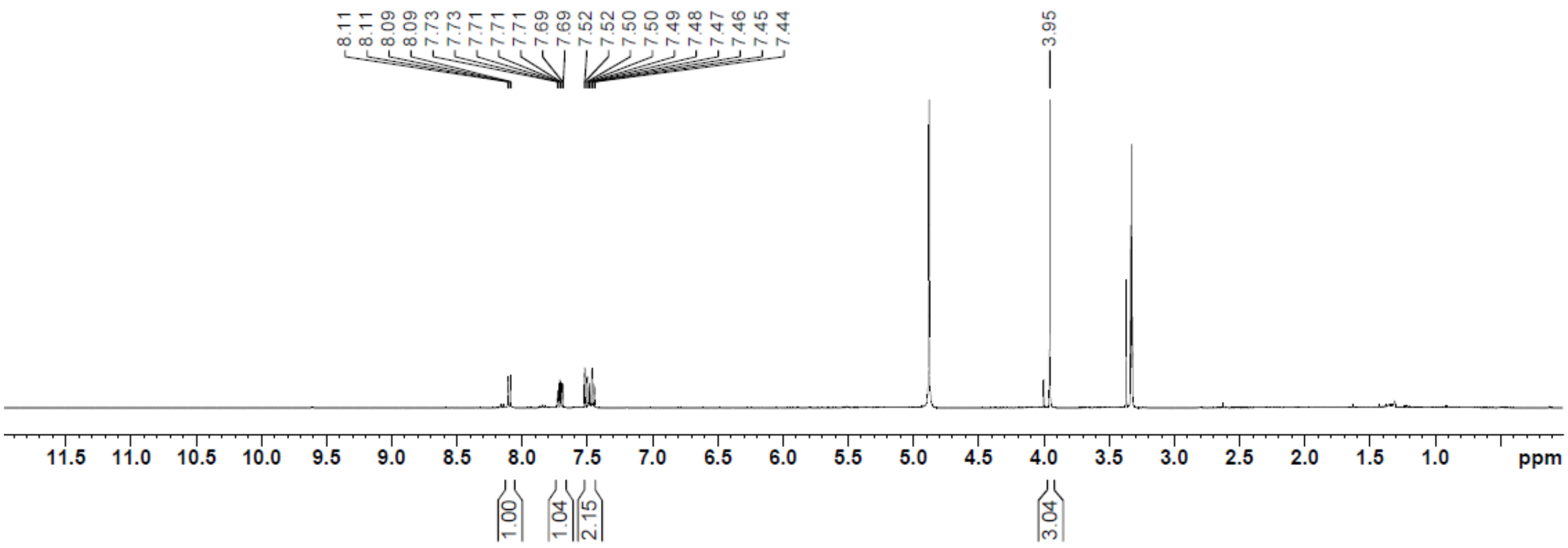
8.11
8.11
8.09
8.09
7.73
7.73
7.71
7.71
7.69
7.69
7.52
7.52
7.50
7.50
7.49
7.48
7.47
7.46
7.45
7.44
3.95
11.5 11.0 10.5 10.0 9.5 9.0 8.5 8.0 7.5 7.0 6.5 6.0 5.5 5.0 4.5 4.0 3.5 3.0 2.5 2.0 1.5 1.0 ppm
1.00
1.04
2.15
3.04

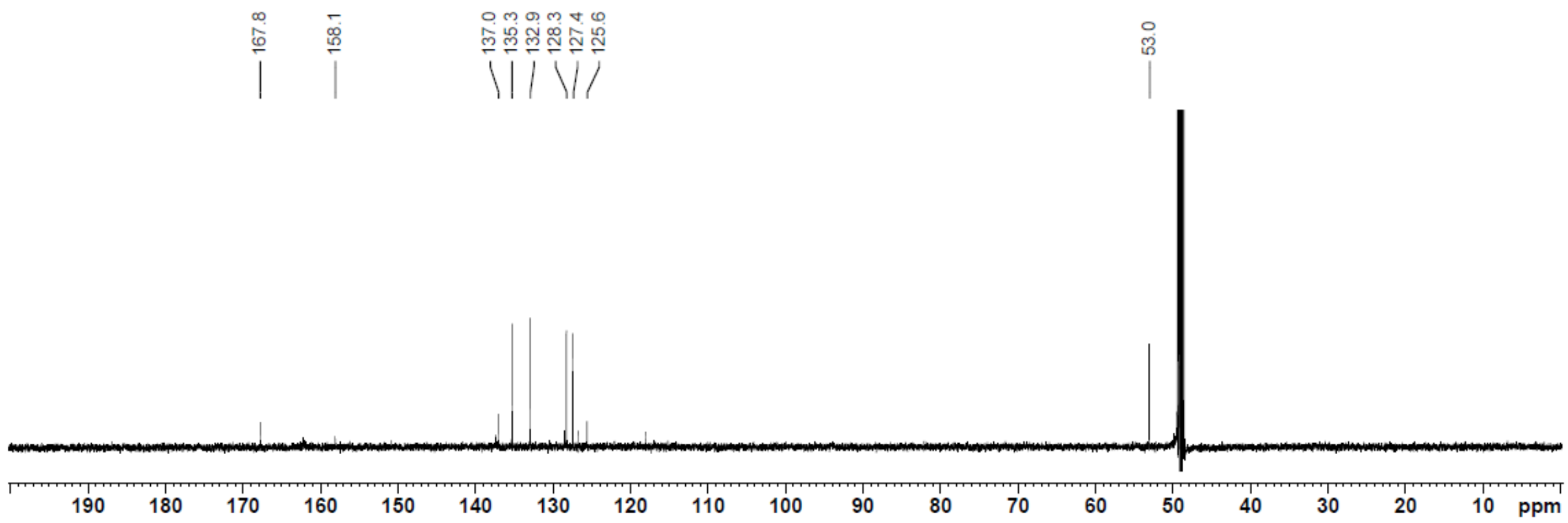
167.8
158.1
137.0
135.3
132.9
128.3
127.4
125.6
53.0
190 180 170 160 150 140 130 120 110 100 90 80 70 60 50 40 30 20 10 ppm

Compound **3**

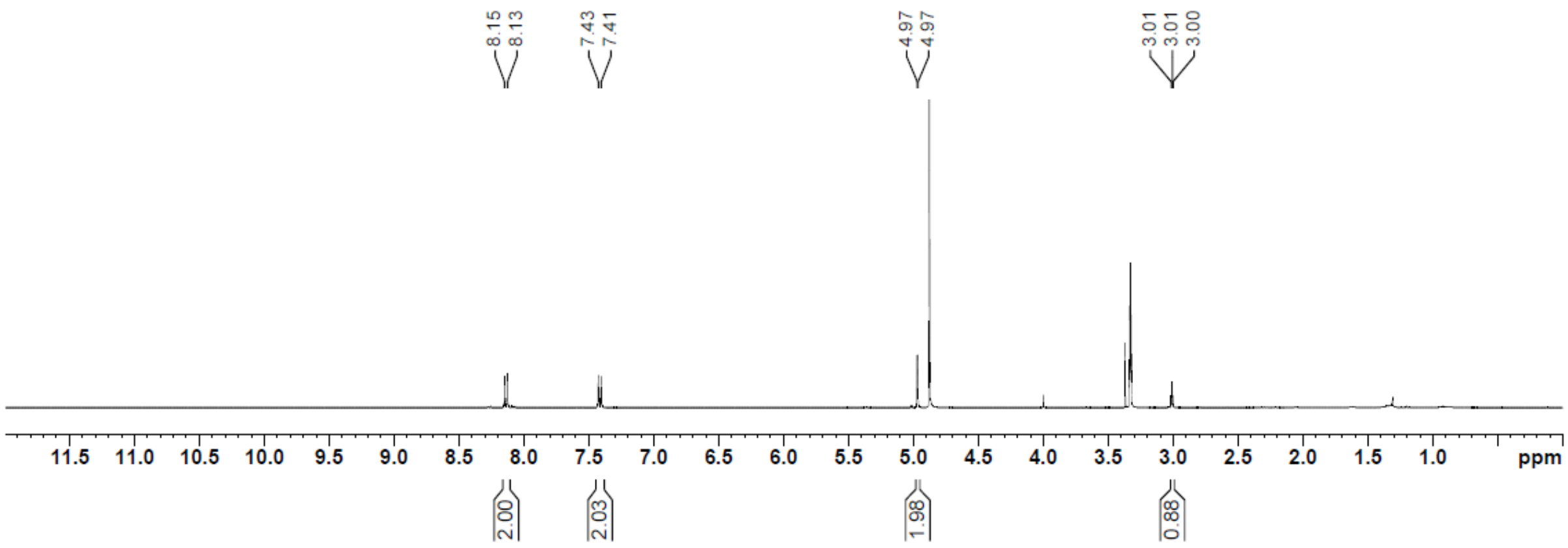

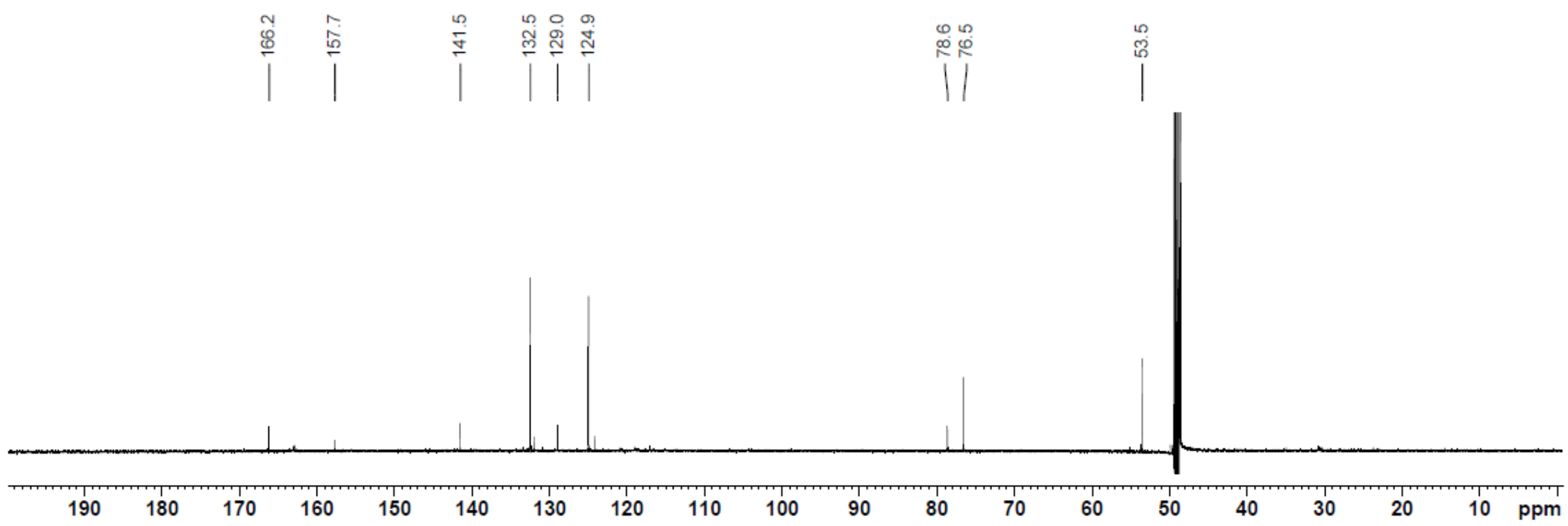

Compound **7**

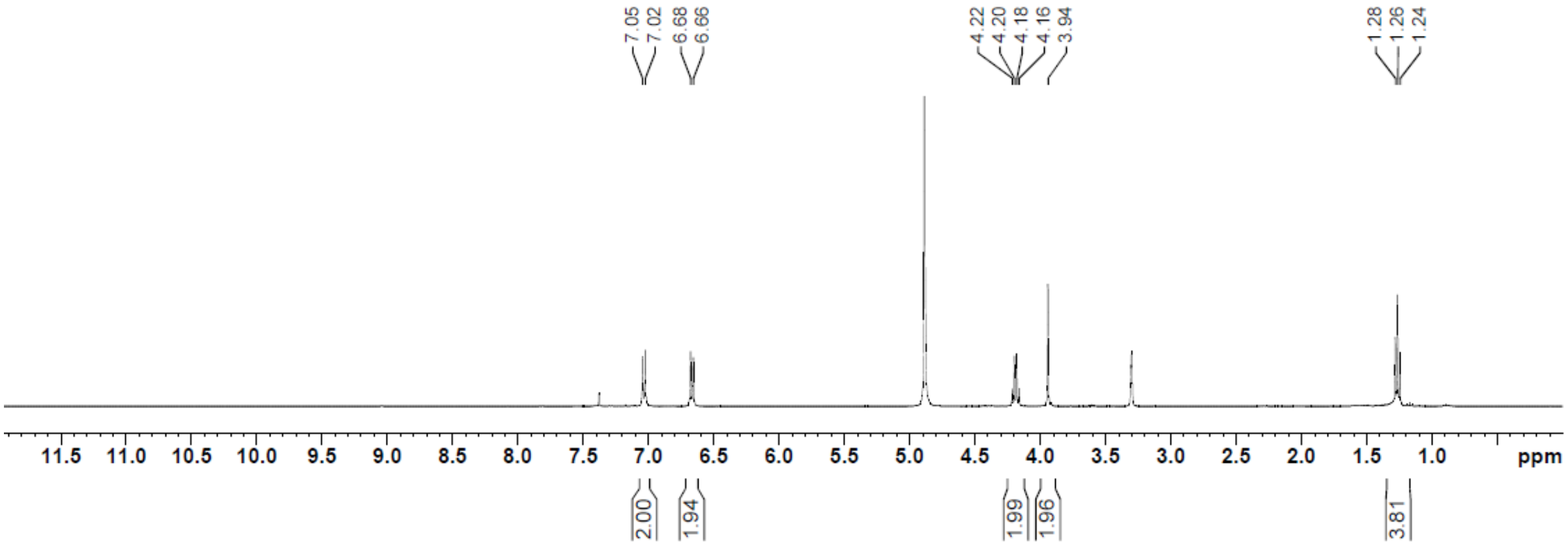

7.05
7.02
6.68
6.66
4.22
4.20
4.18
4.16
3.94
1.28
1.26
1.24
11.5 11.0 10.5 10.0 9.5 9.0 8.5 8.0 7.5 7.0 6.5 6.0 5.5 5.0 4.5 4.0 3.5 3.0 2.5 2.0 1.5 1.0 ppm
2.00
1.94
1.99
1.96
3.81

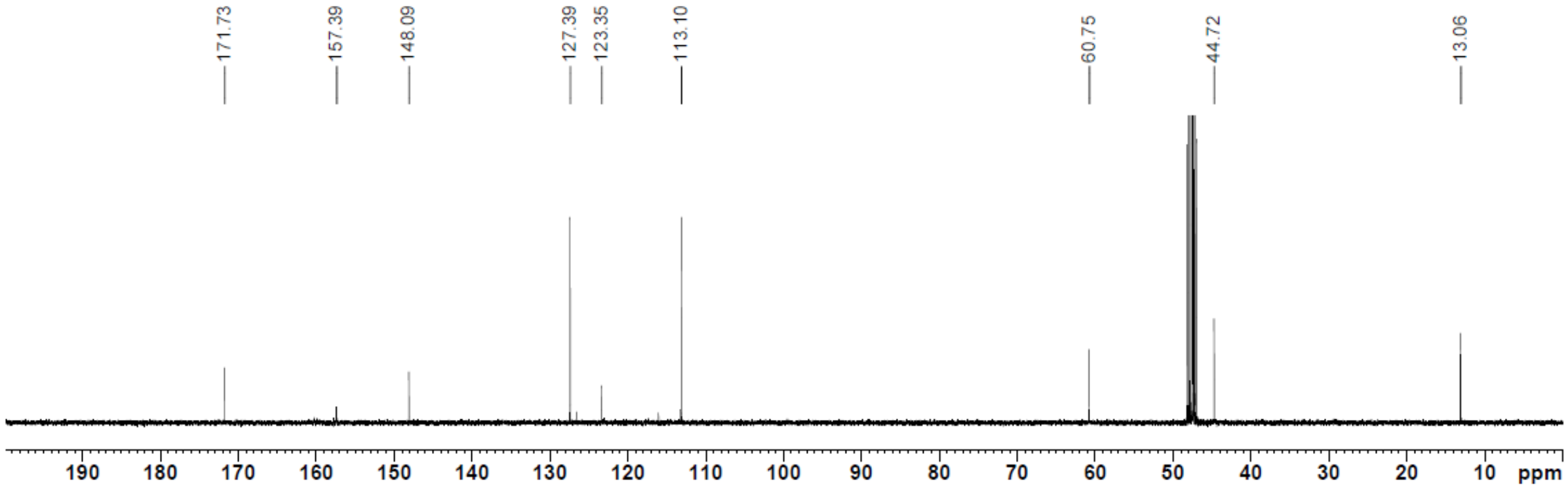

171.73
157.39
148.09
127.39
123.35
113.10
60.75
44.72
13.06
190 180 170 160 150 140 130 120 110 100 90 80 70 60 50 40 30 20 10 ppm

Compound 9

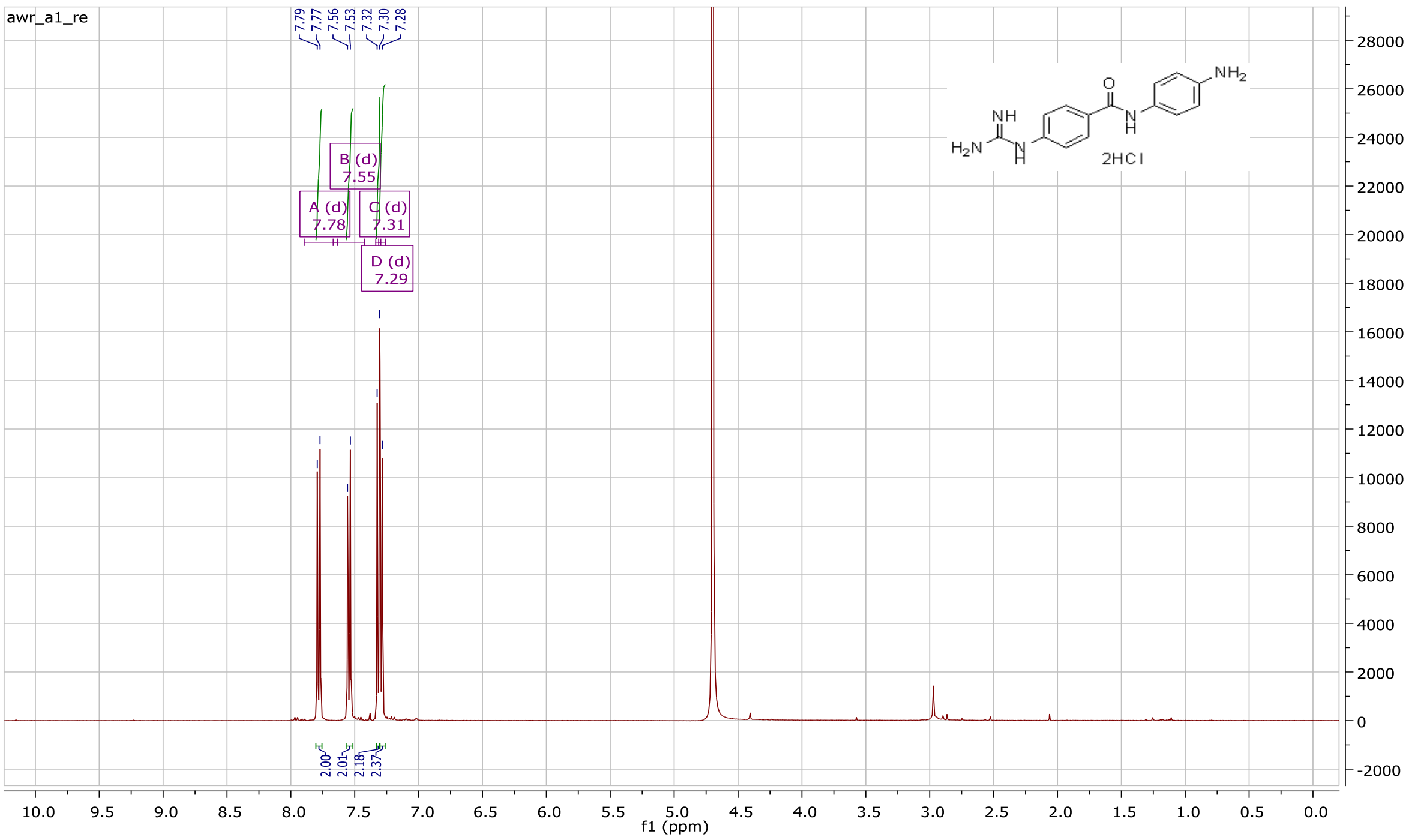
awr_a1_re
7.79
7.77
7.56
7.53
7.32
7.30
7.28
A (d)
7.78
B (d)
7.55
C (d)
7.31
D (d)
7.29
2.00
2.01
2.18
2.37
NH
H2N
N
H
O
N
H
NH2
2HCl
f1 (ppm)

Compound 11

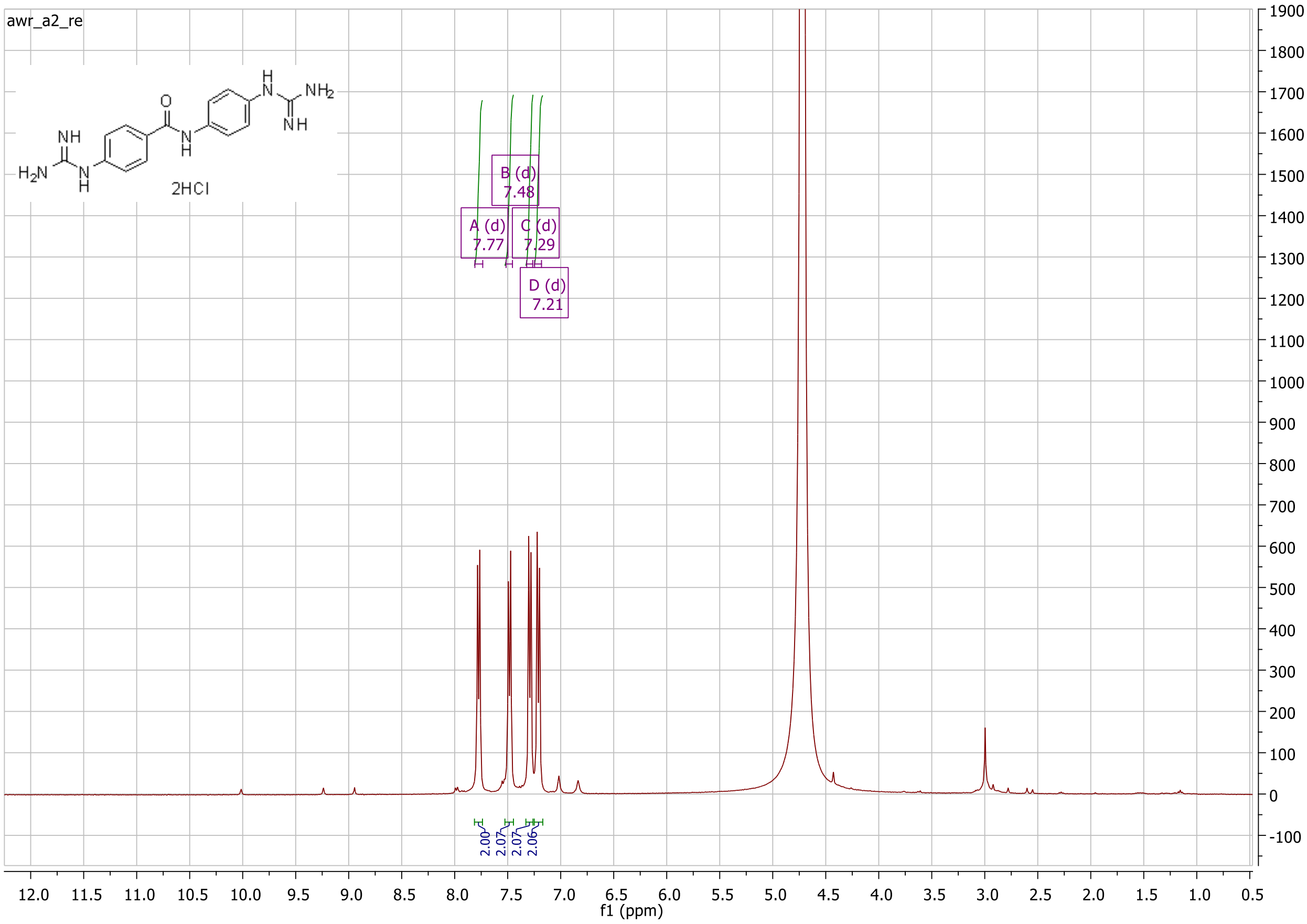

Compound **13**

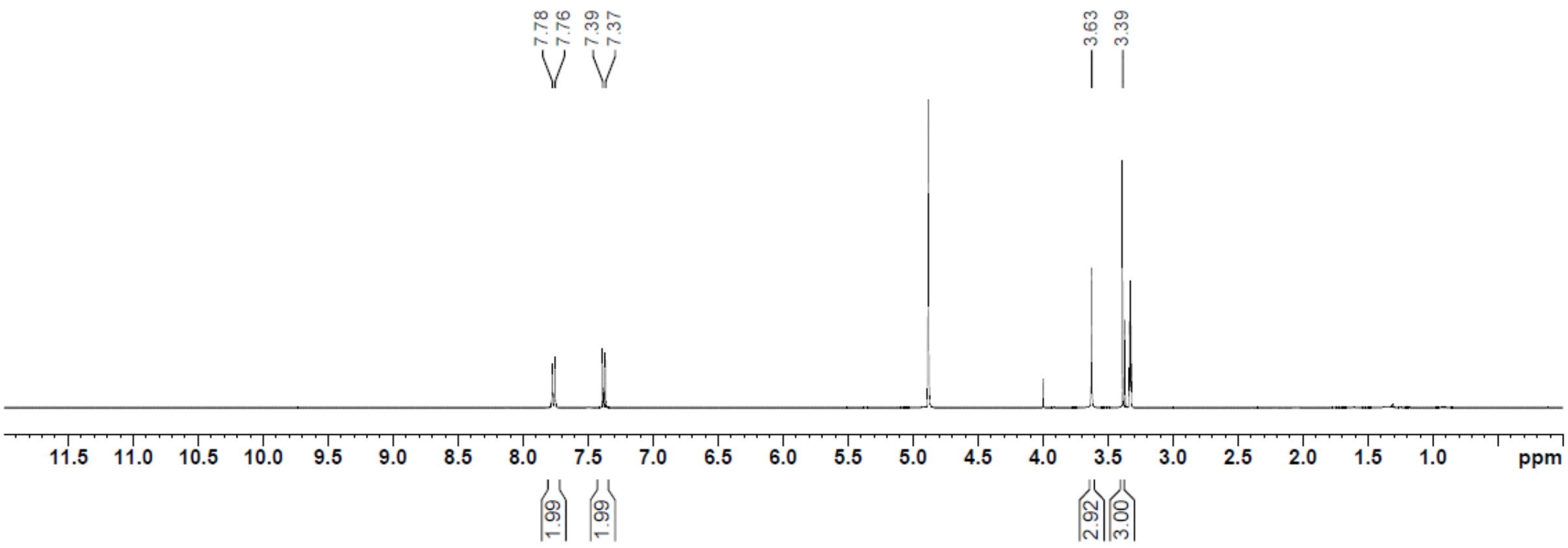
7.78
7.76
7.39
7.37
3.63
3.39
11.5 11.0 10.5 10.0 9.5 9.0 8.5 8.0 7.5 7.0 6.5 6.0 5.5 5.0 4.5 4.0 3.5 3.0 2.5 2.0 1.5 1.0 ppm
1.99
1.99
2.92
3.00

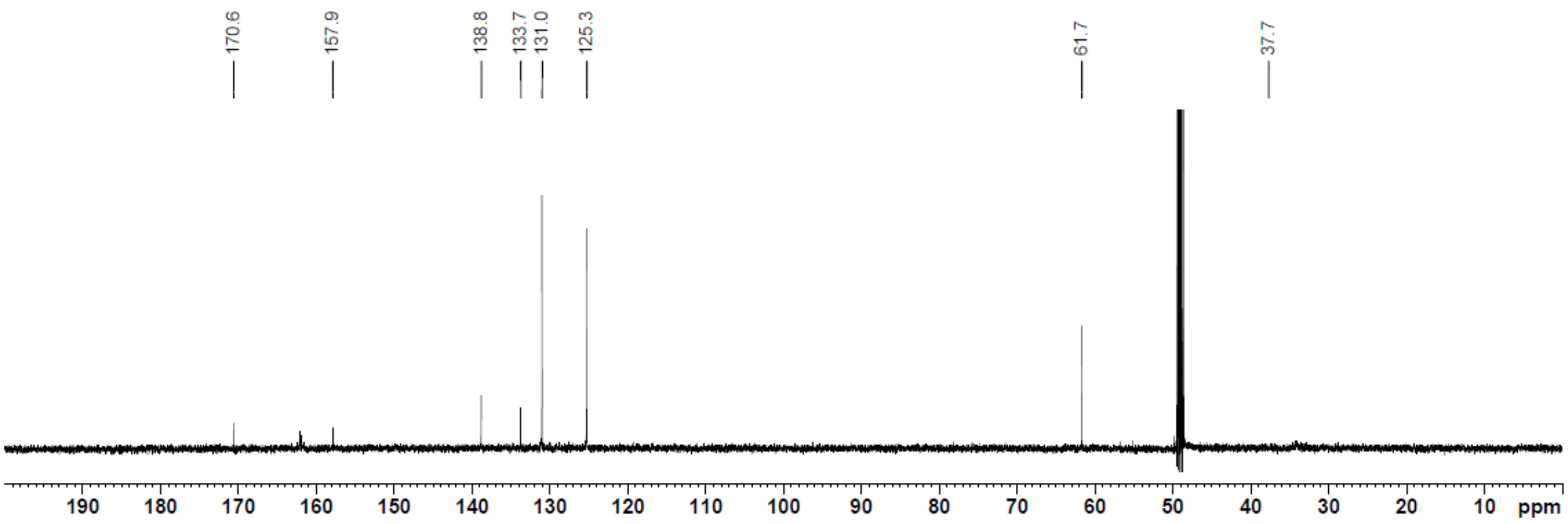
170.6
157.9
138.8
133.7
131.0
125.3
61.7
37.7
190 180 170 160 150 140 130 120 110 100 90 80 70 60 50 40 30 20 10 ppm

Compound **14**

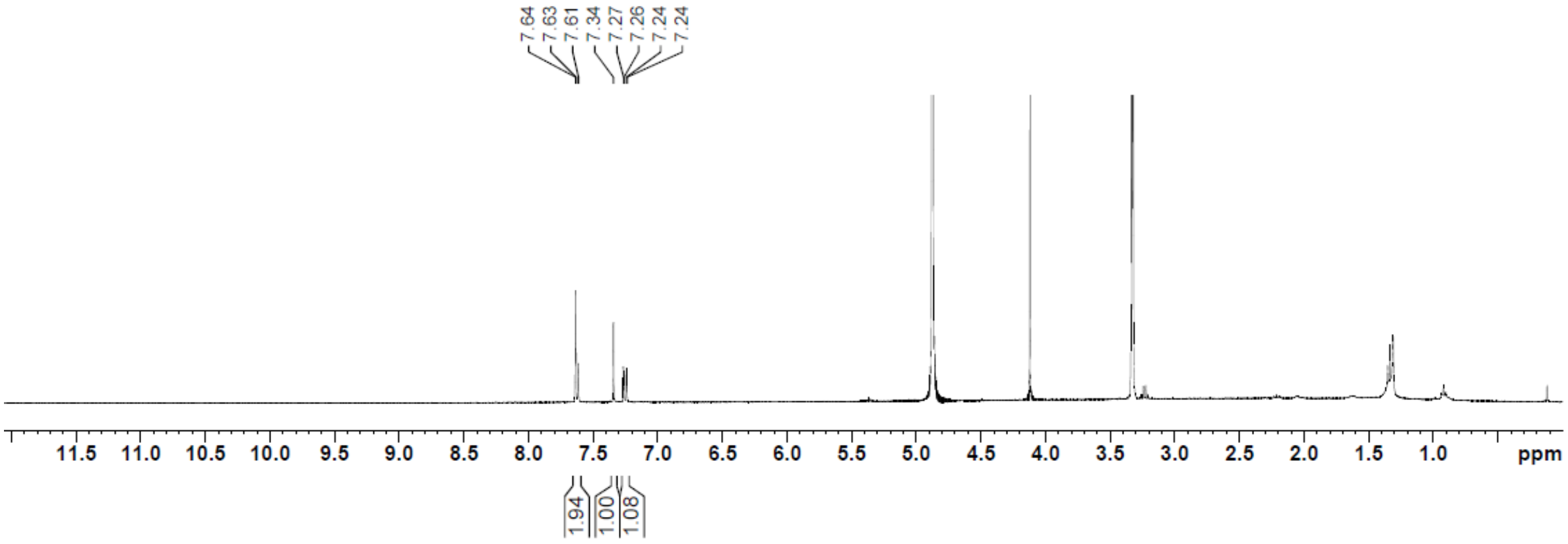

7.64
7.63
7.61
7.34
7.27
7.26
7.24
7.24
11.5 11.0 10.5 10.0 9.5 9.0 8.5 8.0 7.5 7.0 6.5 6.0 5.5 5.0 4.5 4.0 3.5 3.0 2.5 2.0 1.5 1.0 ppm
1.94
1.00
1.08

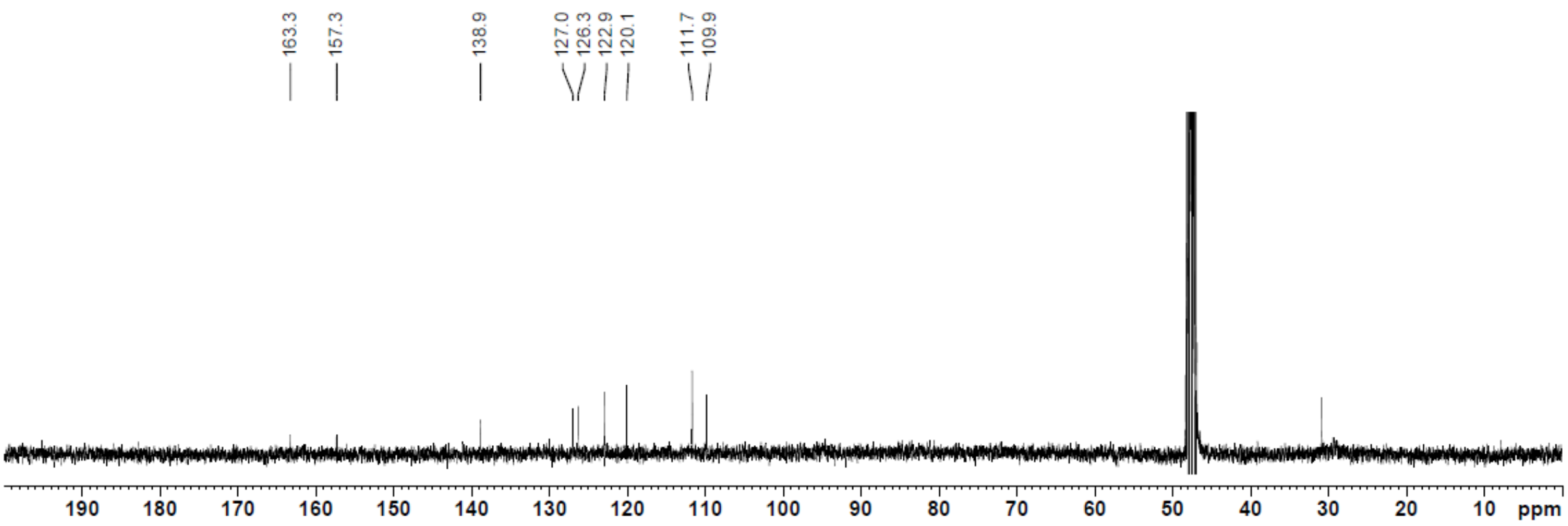

163.3
157.3
138.9
127.0
126.3
122.9
120.1
111.7
109.9
190 180 170 160 150 140 130 120 110 100 90 80 70 60 50 40 30 20 10 ppm

Compound **15**

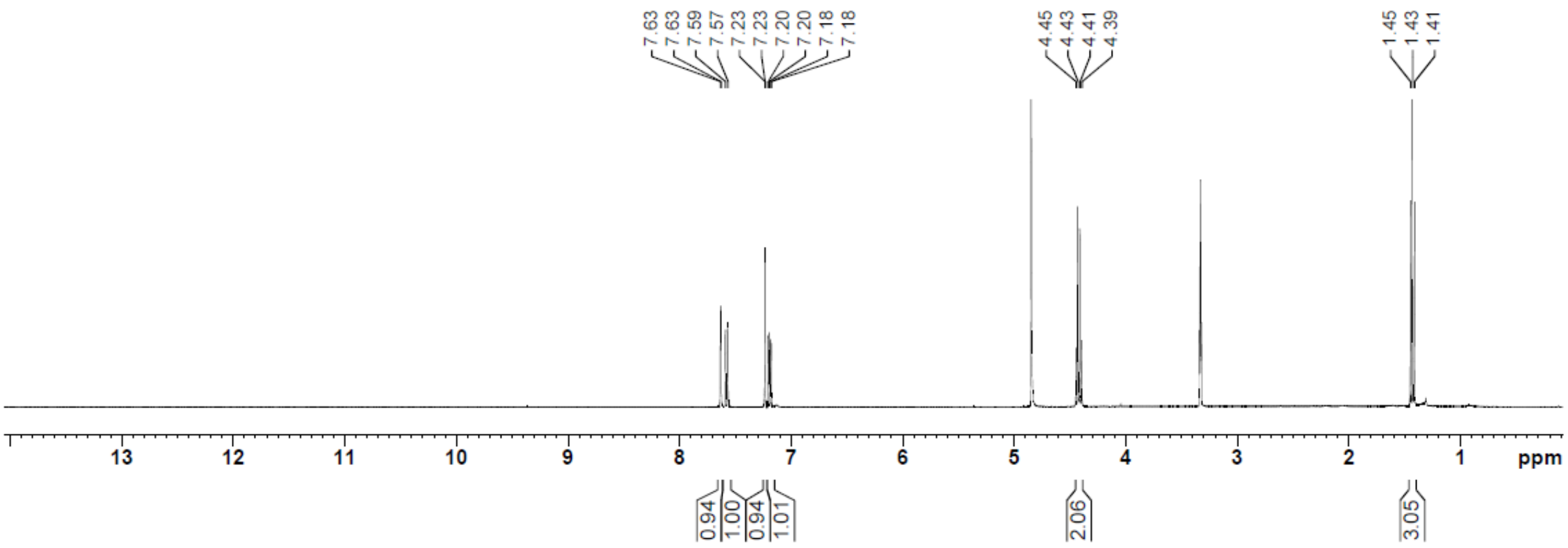

7.63
7.63
7.59
7.57
7.23
7.23
7.20
7.20
7.18
7.18
4.45
4.43
4.41
4.39
1.45
1.43
1.41
13
12
11
10
9
8
7
6
5
4
3
2
1
ppm
0.94
1.00
0.94
1.01
2.06
3.05

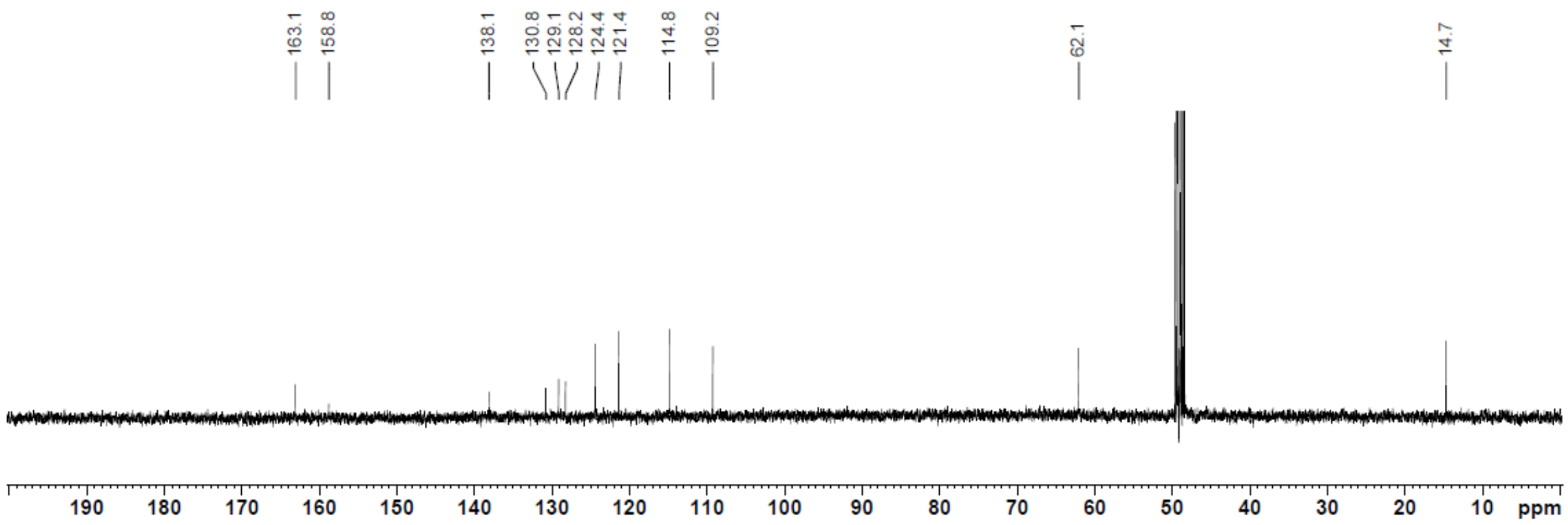

163.1
158.8
138.1
130.8
129.1
128.2
124.4
121.4
114.8
109.2
62.1
14.7
190
180
170
160
150
140
130
120
110
100
90
80
70
60
50
40
30
20
10
ppm

Compound **16**

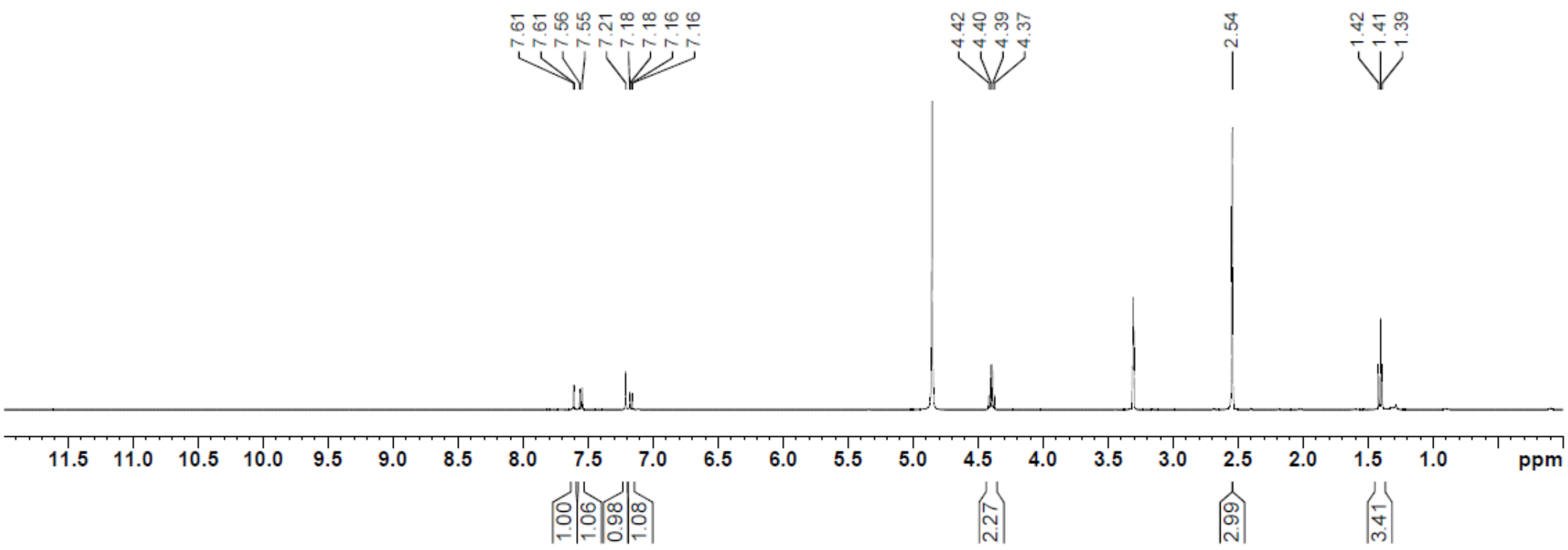

7.61
7.61
7.56
7.55
7.21
7.18
7.18
7.16
7.16
4.42
4.40
4.39
4.37
2.54
1.42
1.41
1.39
11.5 11.0 10.5 10.0 9.5 9.0 8.5 8.0 7.5 7.0 6.5 6.0 5.5 5.0 4.5 4.0 3.5 3.0 2.5 2.0 1.5 1.0 ppm
1.00
1.06
0.98
1.08
2.27
2.99
3.41

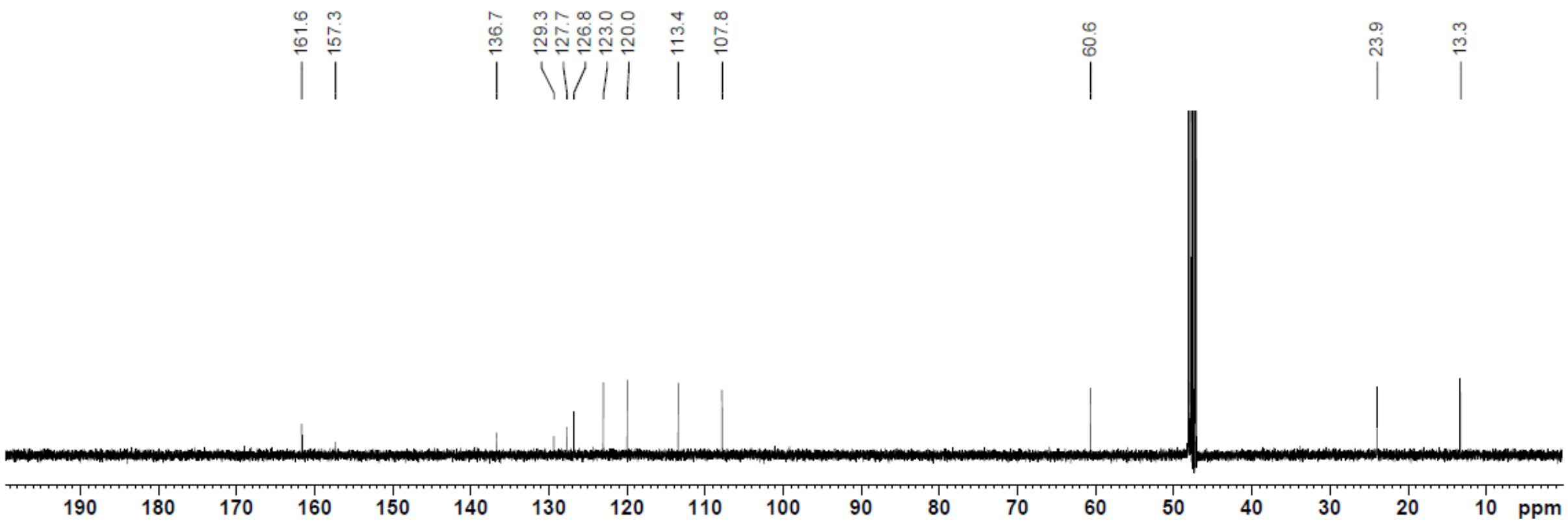

161.6
157.3
136.7
129.3
127.7
126.8
123.0
120.0
113.4
107.8
60.6
23.9
13.3
190 180 170 160 150 140 130 120 110 100 90 80 70 60 50 40 30 20 10 ppm

Compound **17**

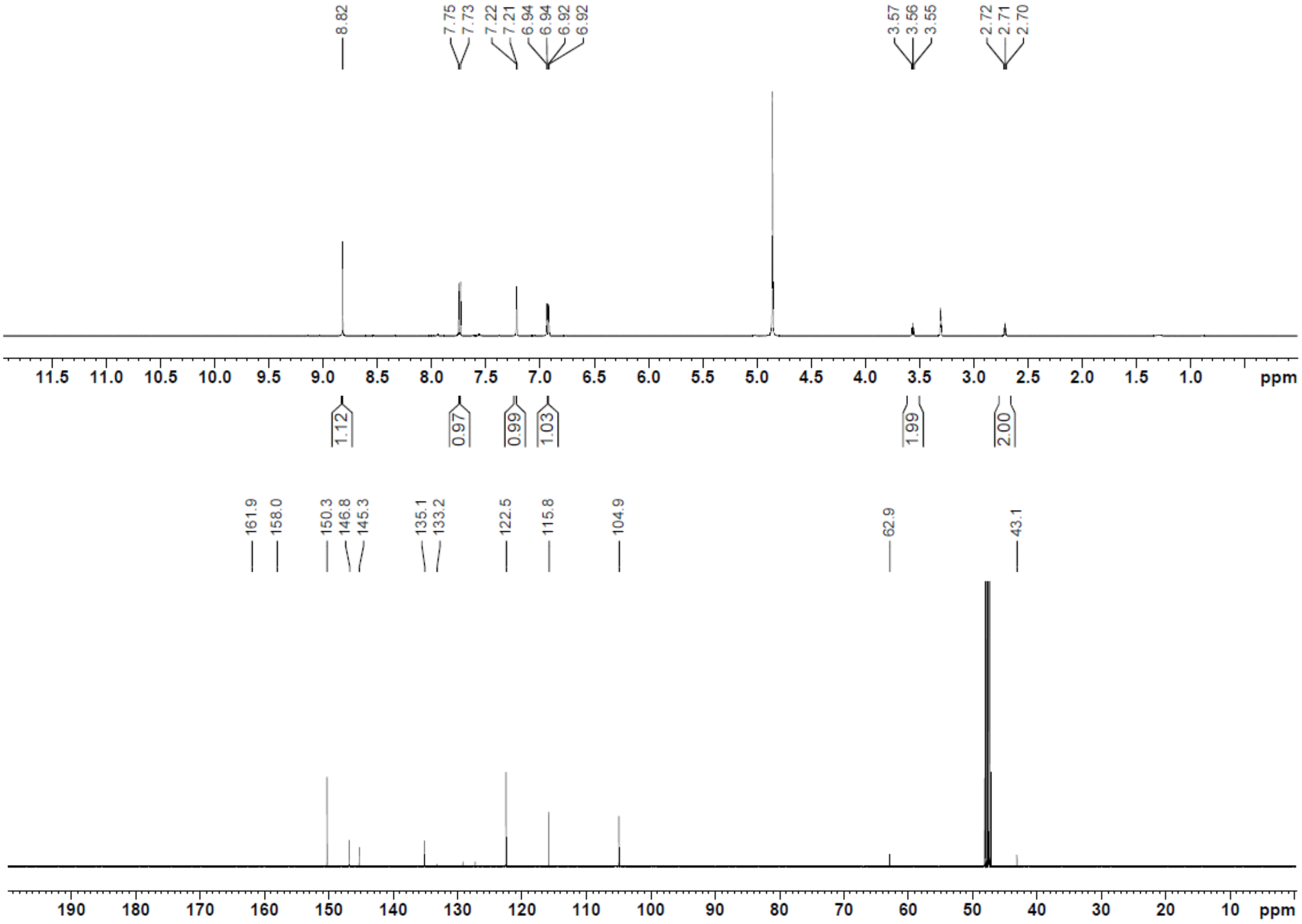
8.82
7.75
7.73
7.22
7.21
6.94
6.94
6.92
6.92
3.57
3.56
3.55
2.72
2.71
2.70
11.5 11.0 10.5 10.0 9.5 9.0 8.5 8.0 7.5 7.0 6.5 6.0 5.5 5.0 4.5 4.0 3.5 3.0 2.5 2.0 1.5 1.0 ppm
1.12
0.97
0.99
1.03
1.99
2.00
161.9
158.0
150.3
146.8
145.3
135.1
133.2
122.5
115.8
104.9
62.9
43.1
190 180 170 160 150 140 130 120 110 100 90 80 70 60 50 40 30 20 10 ppm

Compound **18**

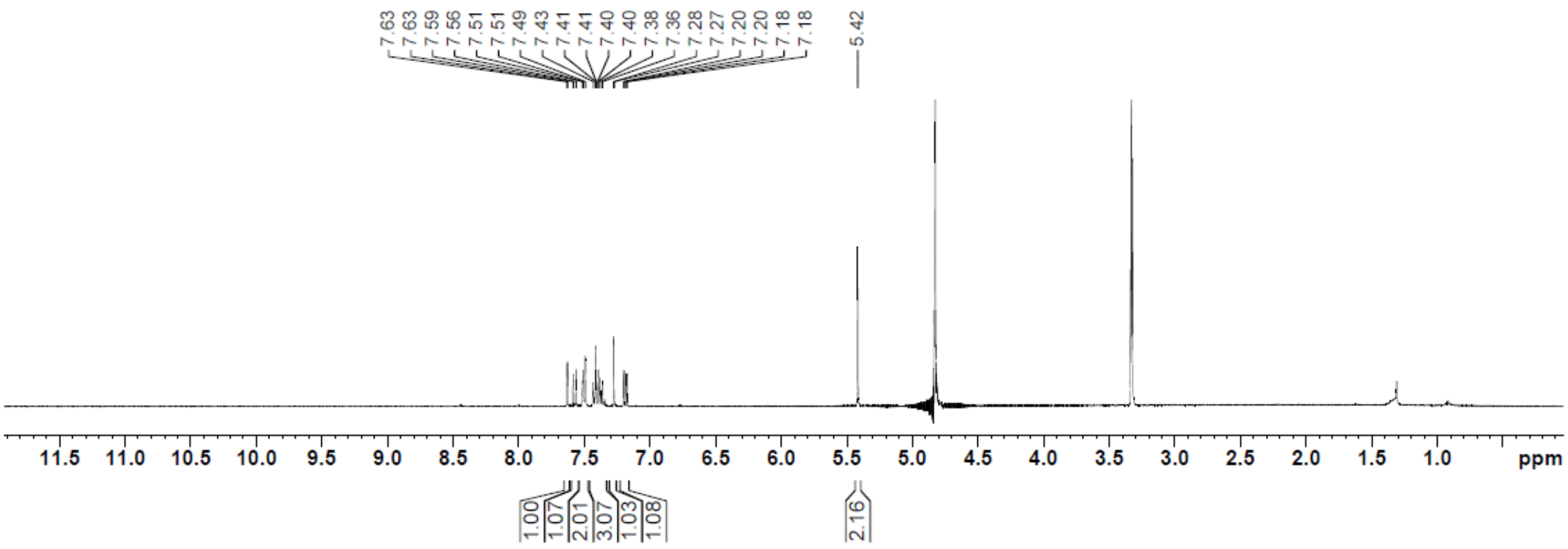

7.63
7.63
7.59
7.56
7.51
7.51
7.49
7.43
7.41
7.41
7.40
7.40
7.38
7.36
7.28
7.27
7.20
7.20
7.18
7.18
5.42
11.5 11.0 10.5 10.0 9.5 9.0 8.5 8.0 7.5 7.0 6.5 6.0 5.5 5.0 4.5 4.0 3.5 3.0 2.5 2.0 1.5 1.0 ppm
1.00
1.07
2.01
3.07
1.03
1.08
2.16

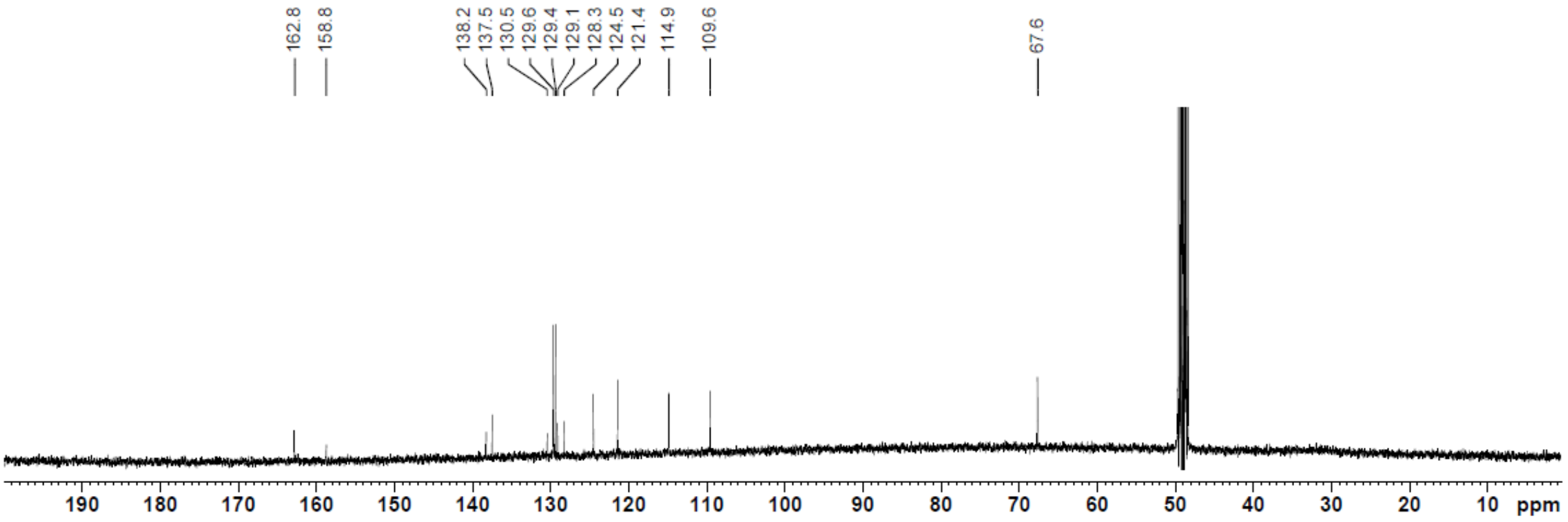

162.8
158.8
138.2
137.5
130.5
129.6
129.4
129.1
128.3
124.5
121.4
114.9
109.6
67.6
190 180 170 160 150 140 130 120 110 100 90 80 70 60 50 40 30 20 10 ppm

Compound **19**

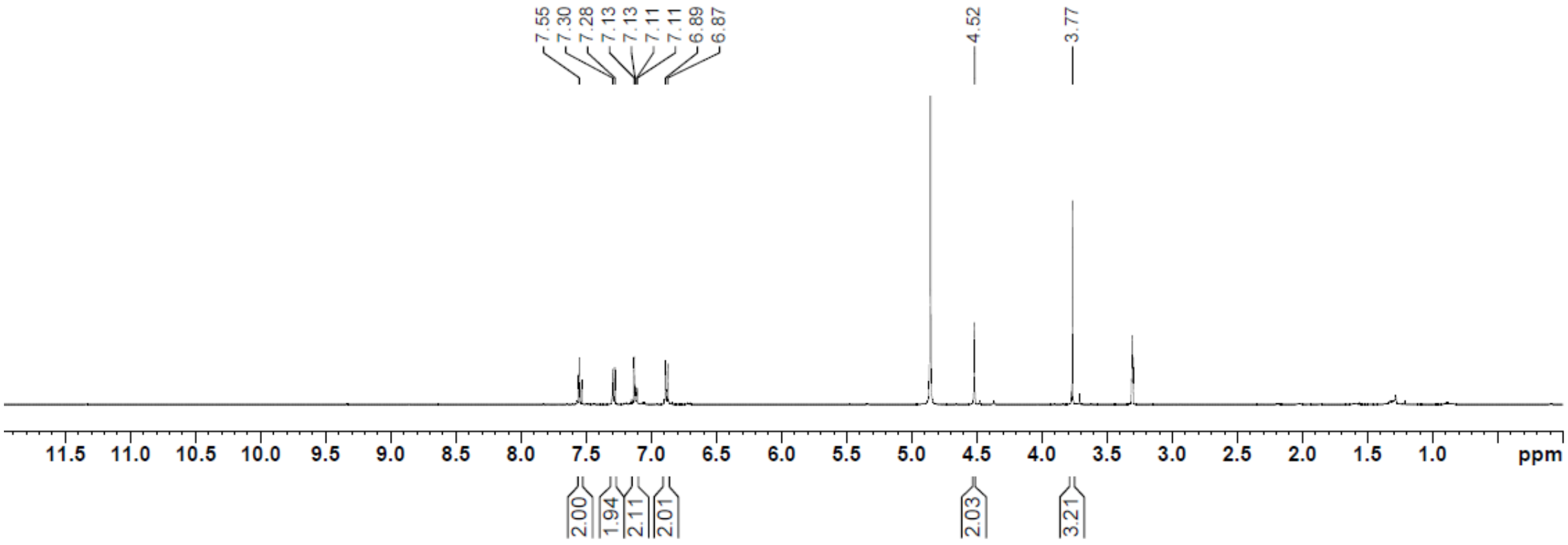
7.55
7.30
7.28
7.13
7.13
7.11
7.11
6.89
6.87
4.52
3.77
11.5 11.0 10.5 10.0 9.5 9.0 8.5 8.0 7.5 7.0 6.5 6.0 5.5 5.0 4.5 4.0 3.5 3.0 2.5 2.0 1.5 1.0 ppm
2.00
1.94
2.11
2.01
2.03
3.21

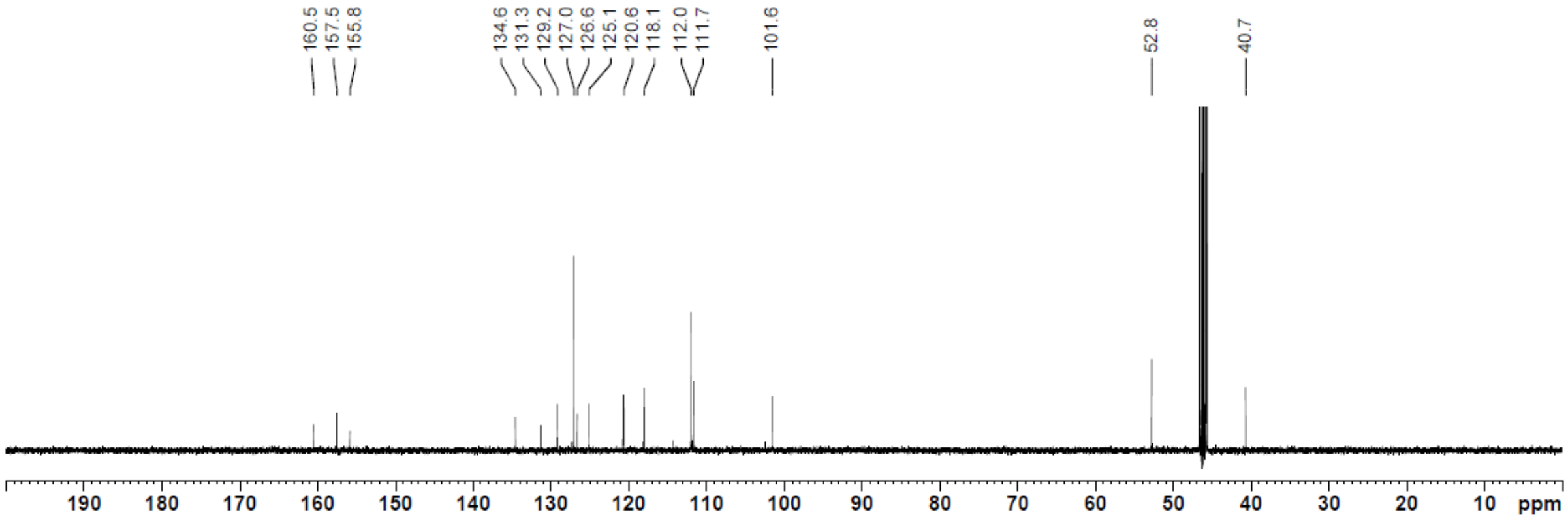
160.5
157.5
155.8
134.6
131.3
129.2
127.0
126.6
125.1
120.6
118.1
112.0
111.7
101.6
52.8
40.7
190 180 170 160 150 140 130 120 110 100 90 80 70 60 50 40 30 20 10 ppm

Compound **20**

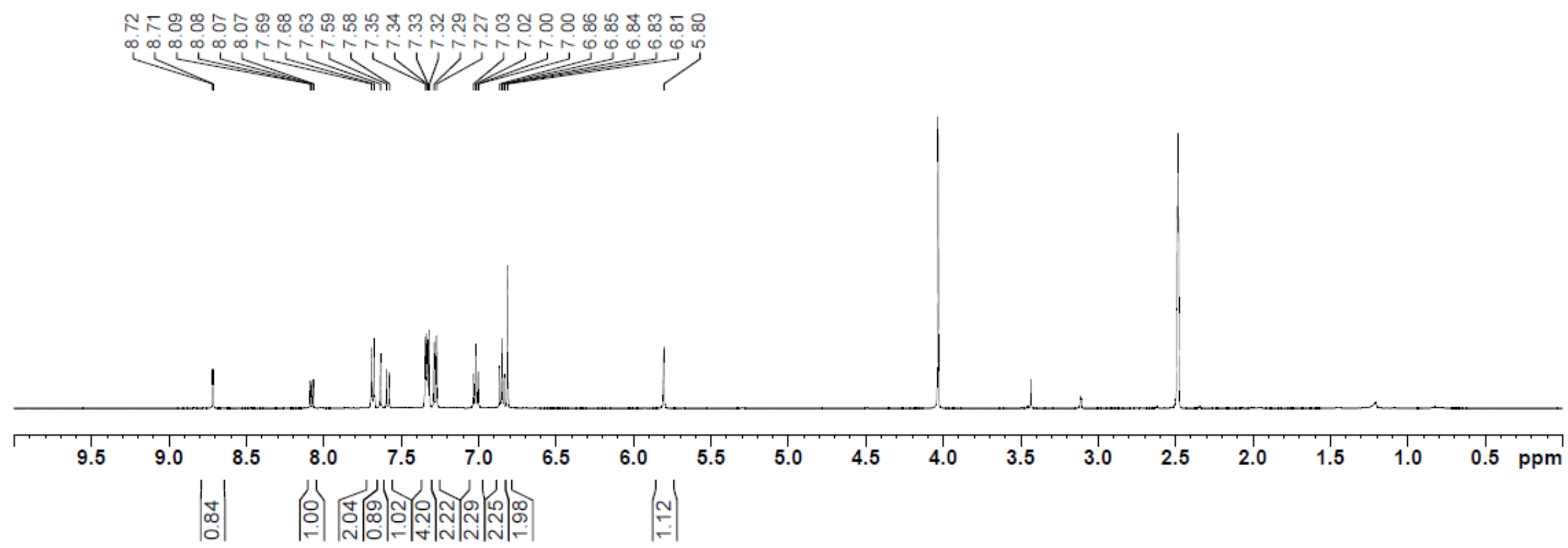
8.72
8.71
8.09
8.08
8.07
8.07
7.69
7.68
7.63
7.59
7.58
7.35
7.34
7.33
7.32
7.29
7.27
7.03
7.02
7.00
7.00
6.86
6.85
6.84
6.83
6.81
5.80
0.84
1.00
2.04
0.89
1.02
4.20
2.22
2.22
2.29
2.25
1.98
1.12
ppm

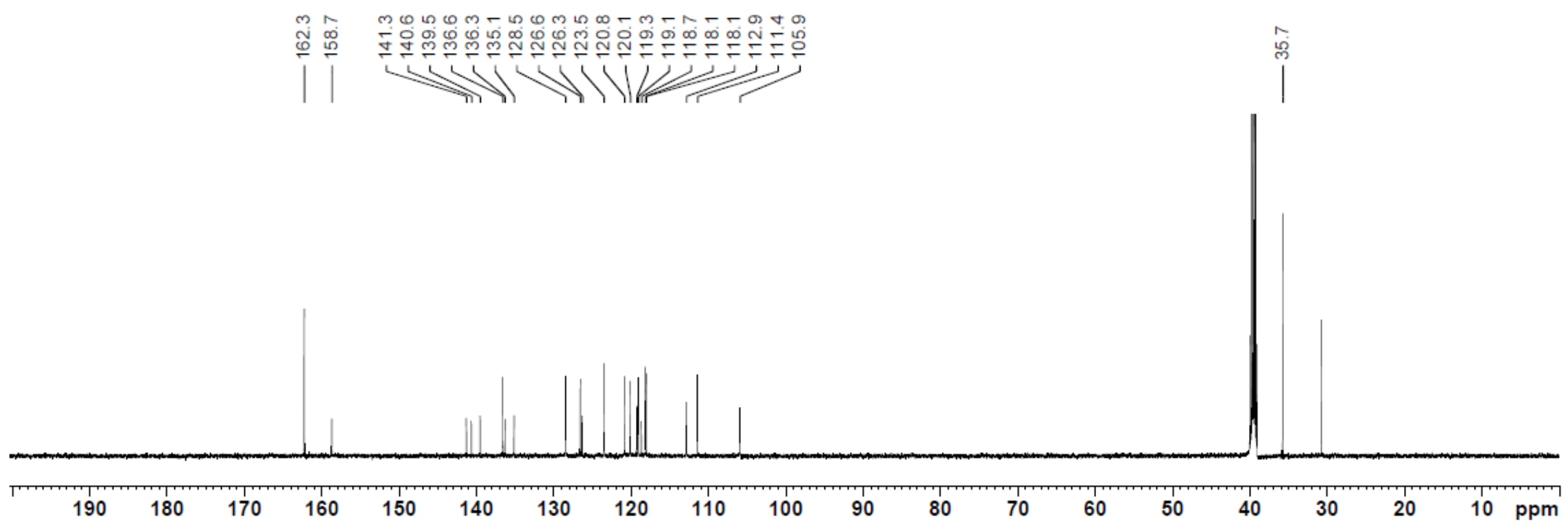
162.3
158.7
141.3
140.6
139.5
136.6
136.3
135.1
128.5
126.6
126.3
123.5
120.8
120.1
119.3
119.1
118.7
118.1
118.1
112.9
111.4
105.9
35.7
ppm

Compound **21**

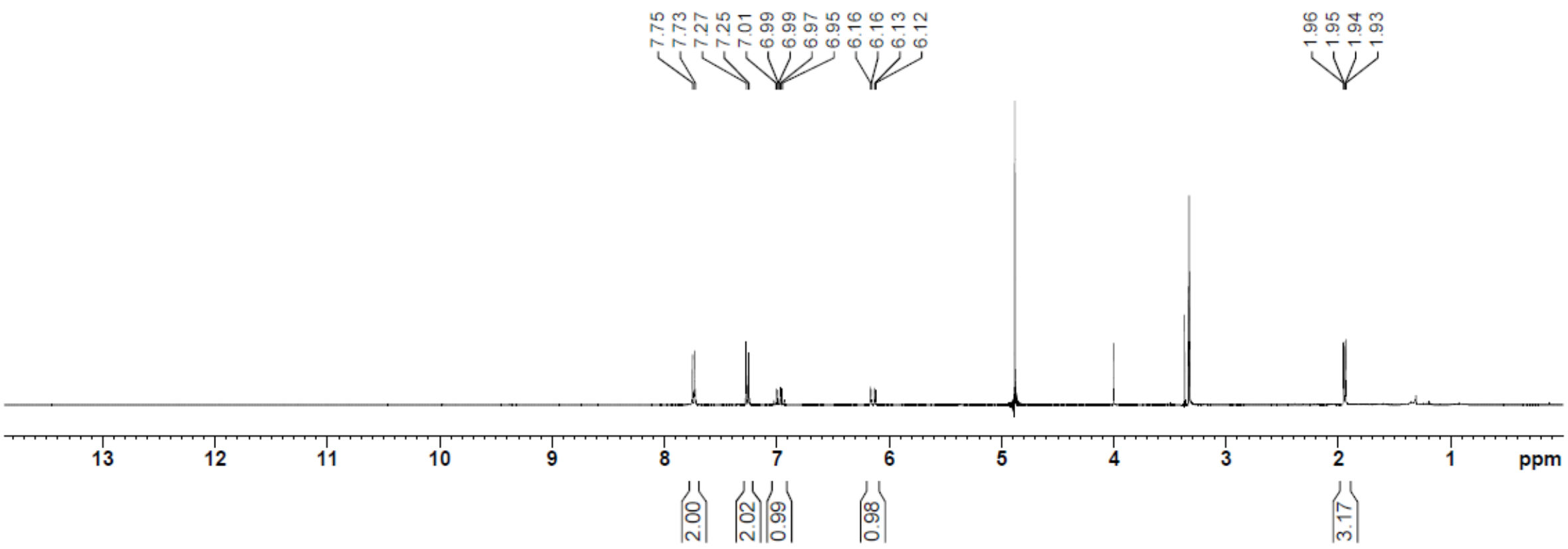

7.75
7.73
7.27
7.25
7.01
6.99
6.99
6.97
6.95
6.16
6.16
6.13
6.12
1.96
1.95
1.94
1.93
13 12 11 10 9 8 7 6 5 4 3 2 1 ppm
2.00
2.02
0.99
0.98
3.17

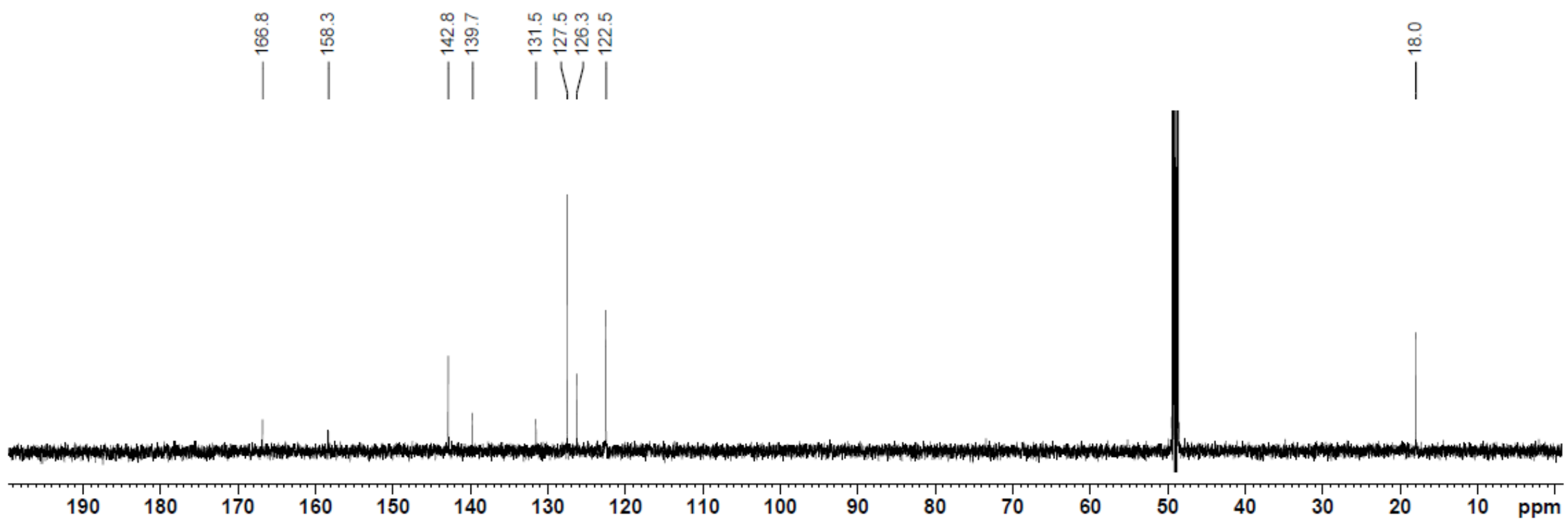

166.8
158.3
142.8
139.7
131.5
127.5
126.3
122.5
18.0
190 180 170 160 150 140 130 120 110 100 90 80 70 60 50 40 30 20 10 ppm

Compound **22**

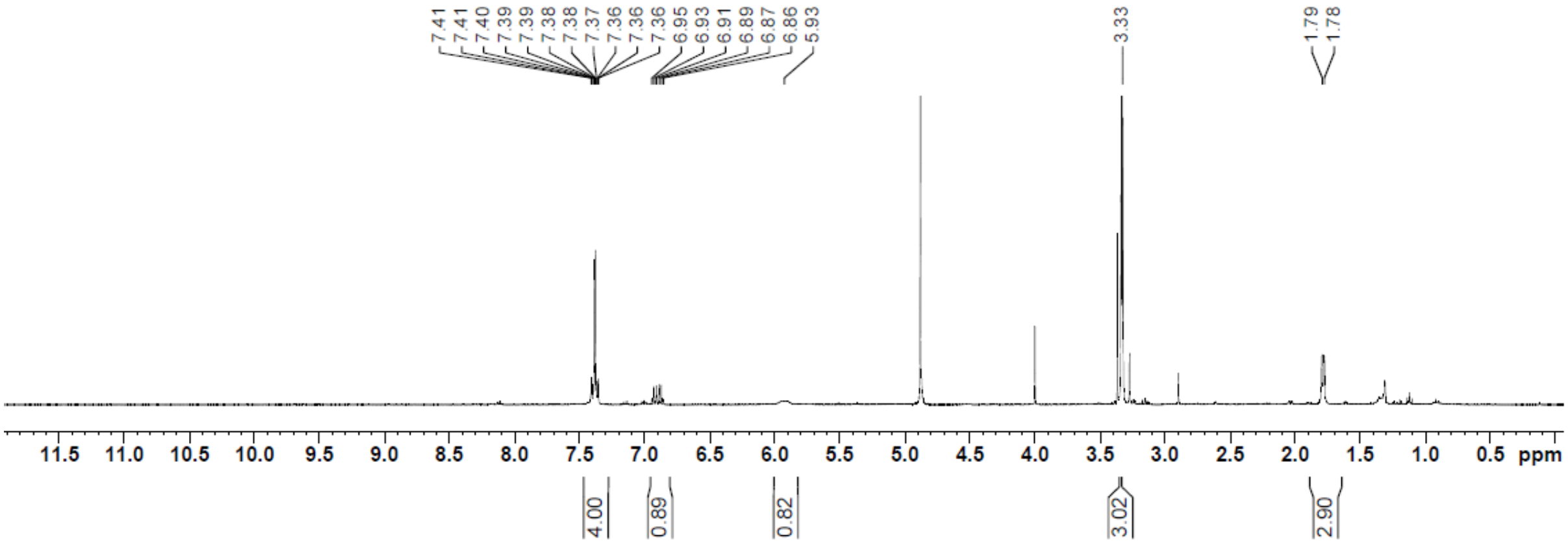

7.41
7.41
7.40
7.39
7.39
7.38
7.38
7.37
7.36
7.36
7.36
6.95
6.93
6.91
6.89
6.87
6.86
5.93
3.33
1.79
1.78
11.5 11.0 10.5 10.0 9.5 9.0 8.5 8.0 7.5 7.0 6.5 6.0 5.5 5.0 4.5 4.0 3.5 3.0 2.5 2.0 1.5 1.0 0.5 ppm
4.00
0.89
0.82
3.02
2.90

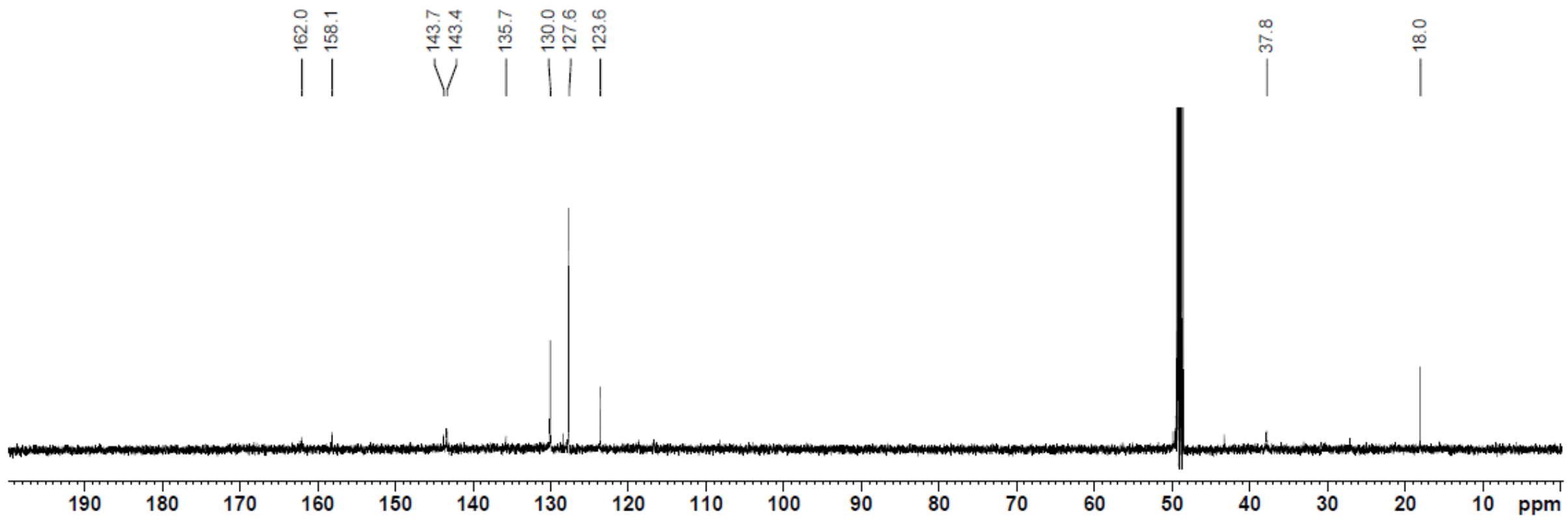

162.0
158.1
143.7
143.4
135.7
130.0
127.6
123.6
37.8
18.0
190 180 170 160 150 140 130 120 110 100 90 80 70 60 50 40 30 20 10 ppm

Compound **23**

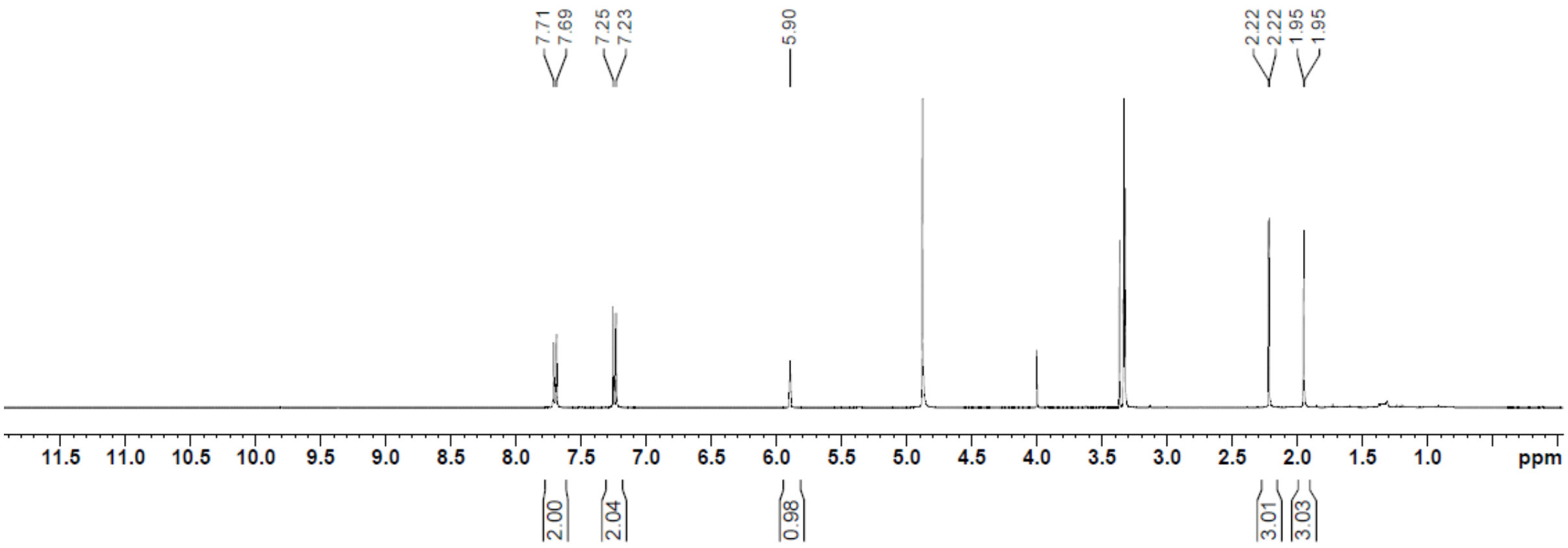
7.71
7.69
7.25
7.23
5.90
2.22
2.22
1.95
1.95
11.5 11.0 10.5 10.0 9.5 9.0 8.5 8.0 7.5 7.0 6.5 6.0 5.5 5.0 4.5 4.0 3.5 3.0 2.5 2.0 1.5 1.0 ppm
2.00
2.04
0.98
3.01
3.03

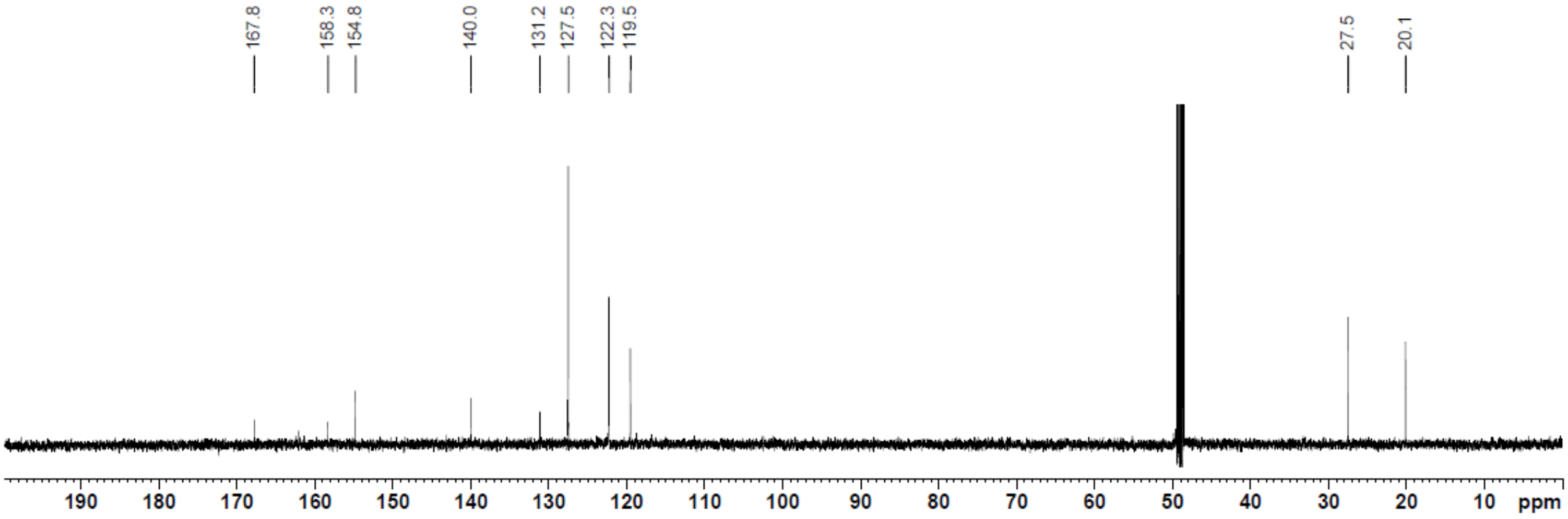
167.8
158.3
154.8
140.0
131.2
127.5
122.3
119.5
27.5
20.1
190 180 170 160 150 140 130 120 110 100 90 80 70 60 50 40 30 20 10 ppm

Compound **24**

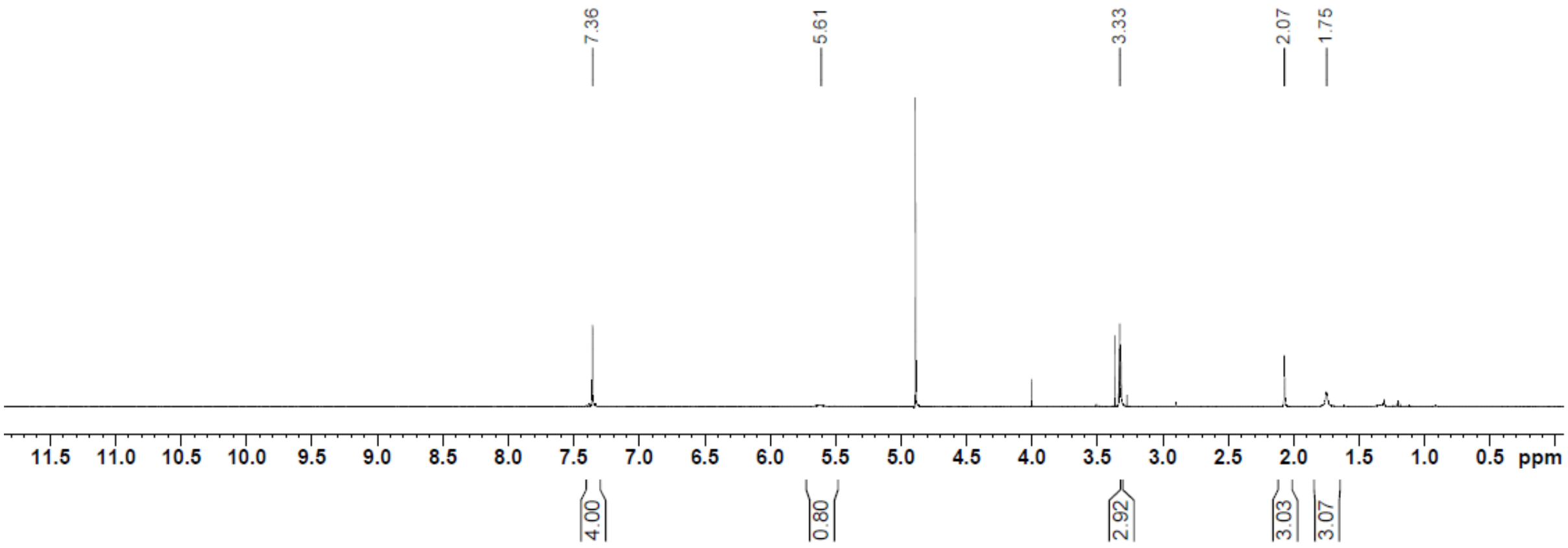
7.36
5.61
3.33
2.07
1.75
11.5 11.0 10.5 10.0 9.5 9.0 8.5 8.0 7.5 7.0 6.5 6.0 5.5 5.0 4.5 4.0 3.5 3.0 2.5 2.0 1.5 1.0 0.5 ppm
4.00
0.80
2.92
3.03
3.07

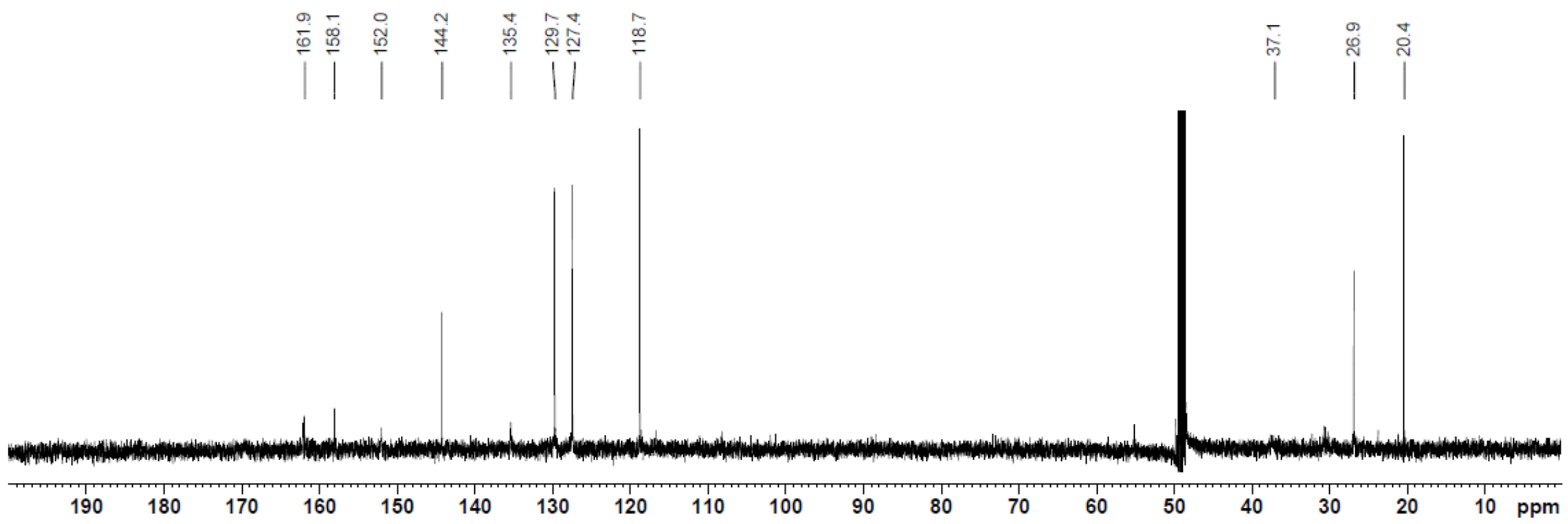
161.9
158.1
152.0
144.2
135.4
129.7
127.4
118.7
37.1
26.9
20.4
190 180 170 160 150 140 130 120 110 100 90 80 70 60 50 40 30 20 10 ppm

Compound **25**

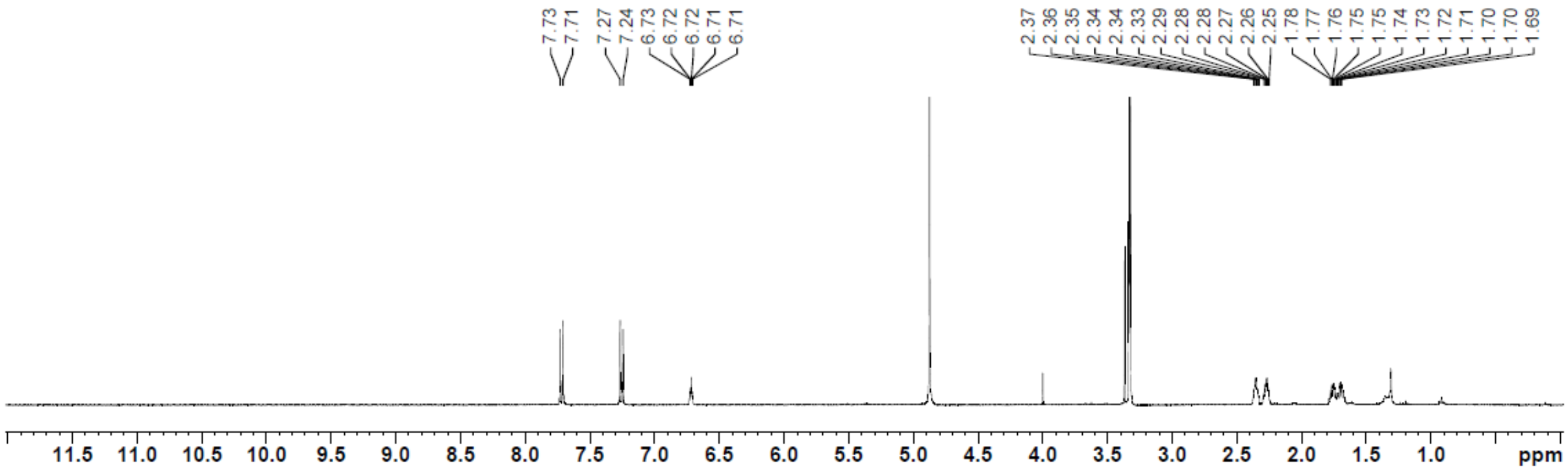
7.73
7.71
7.27
7.24
6.73
6.72
6.72
6.71
6.71
2.37
2.36
2.35
2.34
2.34
2.33
2.29
2.28
2.28
2.27
2.26
2.25
1.78
1.77
1.76
1.75
1.75
1.74
1.73
1.72
1.71
1.70
1.69
11.5 11.0 10.5 10.0 9.5 9.0 8.5 8.0 7.5 7.0 6.5 6.0 5.5 5.0 4.5 4.0 3.5 3.0 2.5 2.0 1.5 1.0 ppm

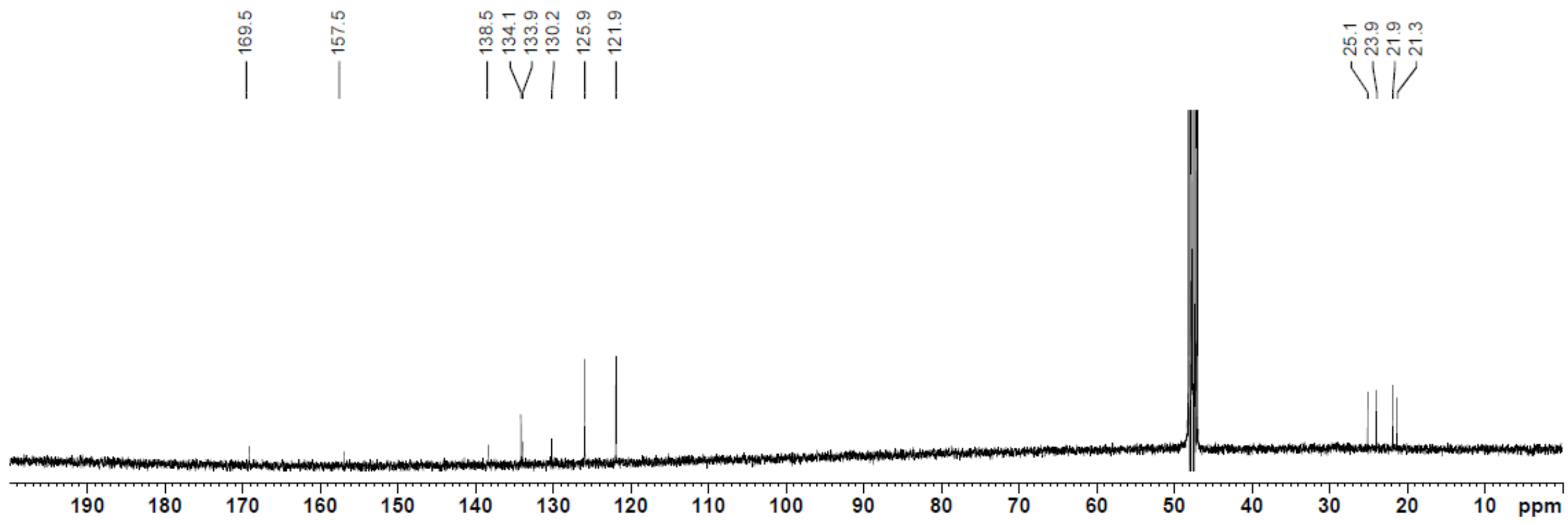
169.5
157.5
138.5
134.1
133.9
130.2
125.9
121.9
25.1
23.9
21.9
21.3
190 180 170 160 150 140 130 120 110 100 90 80 70 60 50 40 30 20 10 ppm

Compound **26**

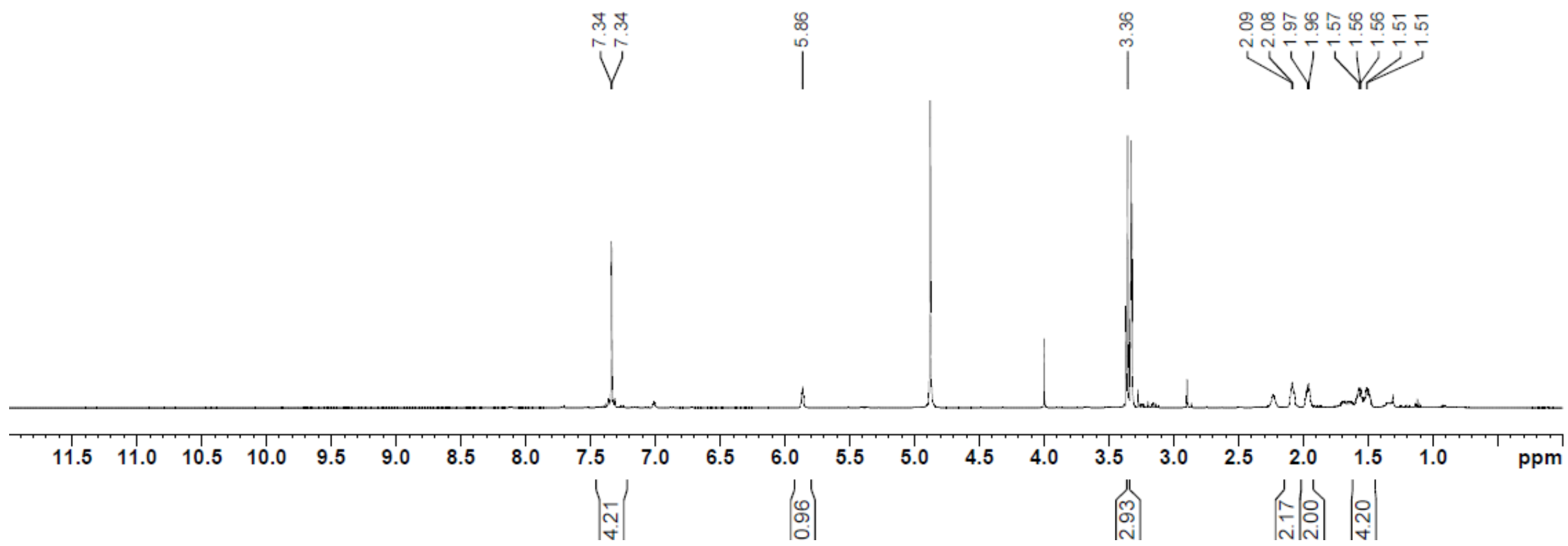
7.34
7.34
5.86
3.36
2.09
2.08
1.97
1.96
1.57
1.56
1.56
1.51
1.51
11.5 11.0 10.5 10.0 9.5 9.0 8.5 8.0 7.5 7.0 6.5 6.0 5.5 5.0 4.5 4.0 3.5 3.0 2.5 2.0 1.5 1.0 ppm
4.21
0.96
2.93
2.17
2.00
4.20

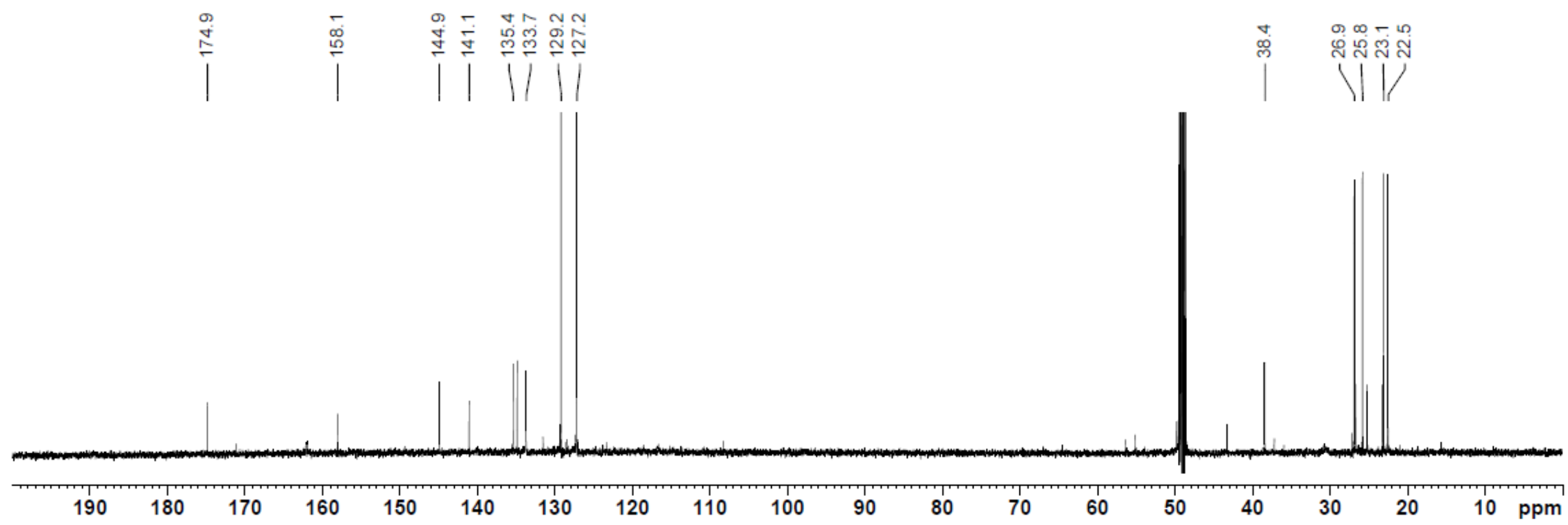
174.9
158.1
144.9
141.1
135.4
133.7
129.2
127.2
38.4
26.9
25.8
23.1
22.5
190 180 170 160 150 140 130 120 110 100 90 80 70 60 50 40 30 20 10 ppm

Compound **27**

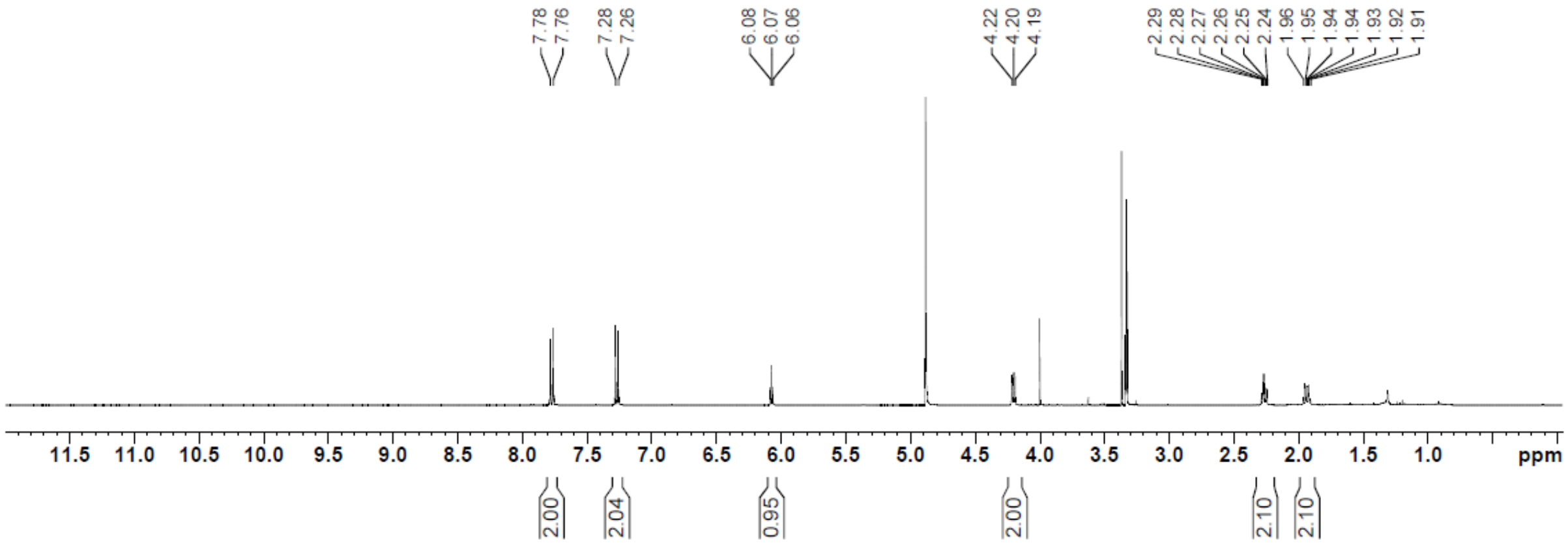

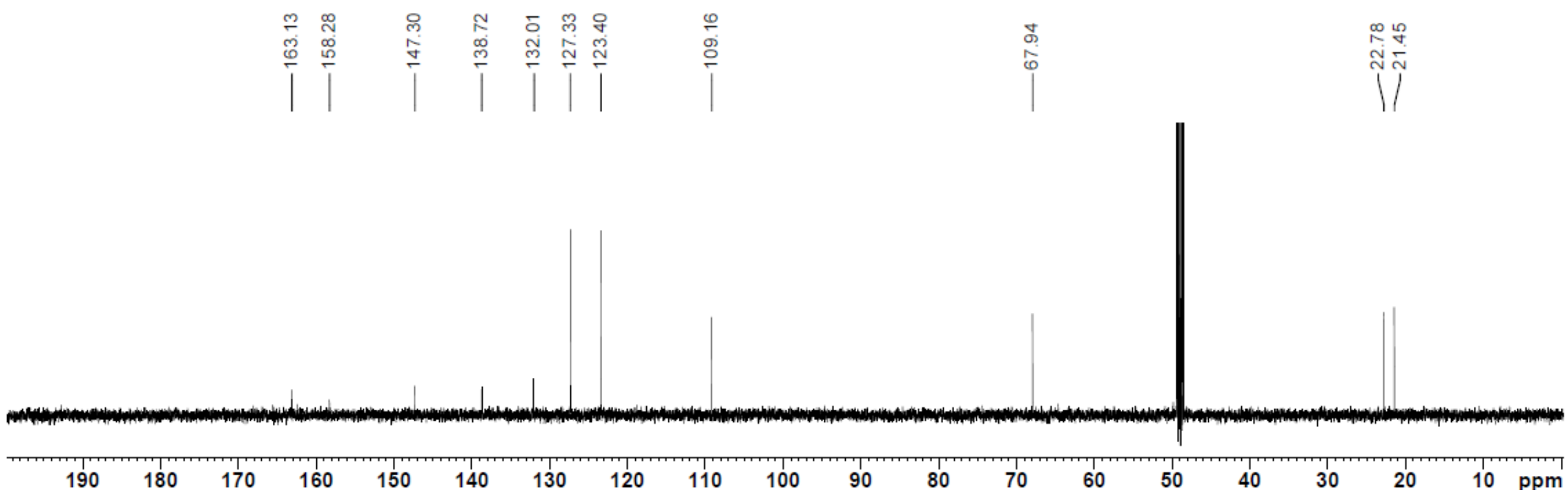

Compound **28**

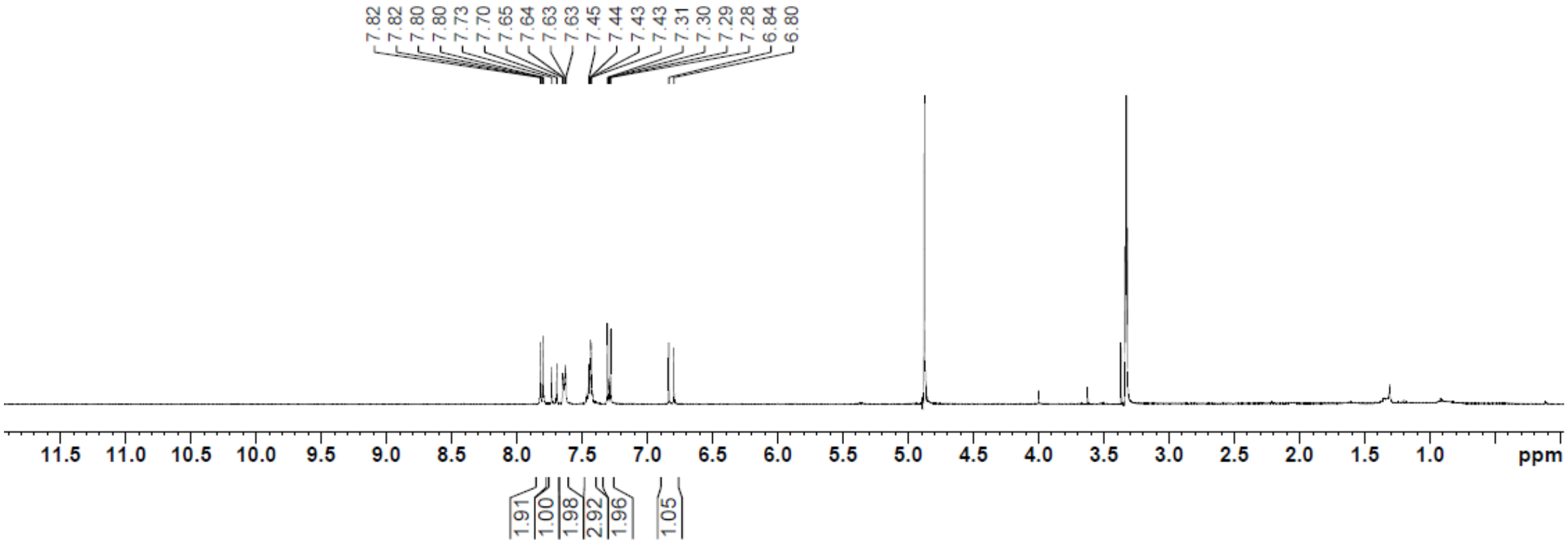

7.82 7.82 7.80 7.80 7.73 7.70 7.65 7.64 7.63 7.63 7.45 7.44 7.43 7.43 7.31 7.30 7.29 7.28 6.84 6.80
11.5 11.0 10.5 10.0 9.5 9.0 8.5 8.0 7.5 7.0 6.5 6.0 5.5 5.0 4.5 4.0 3.5 3.0 2.5 2.0 1.5 1.0 ppm
1.91 1.00 1.98 2.92 1.96 1.05

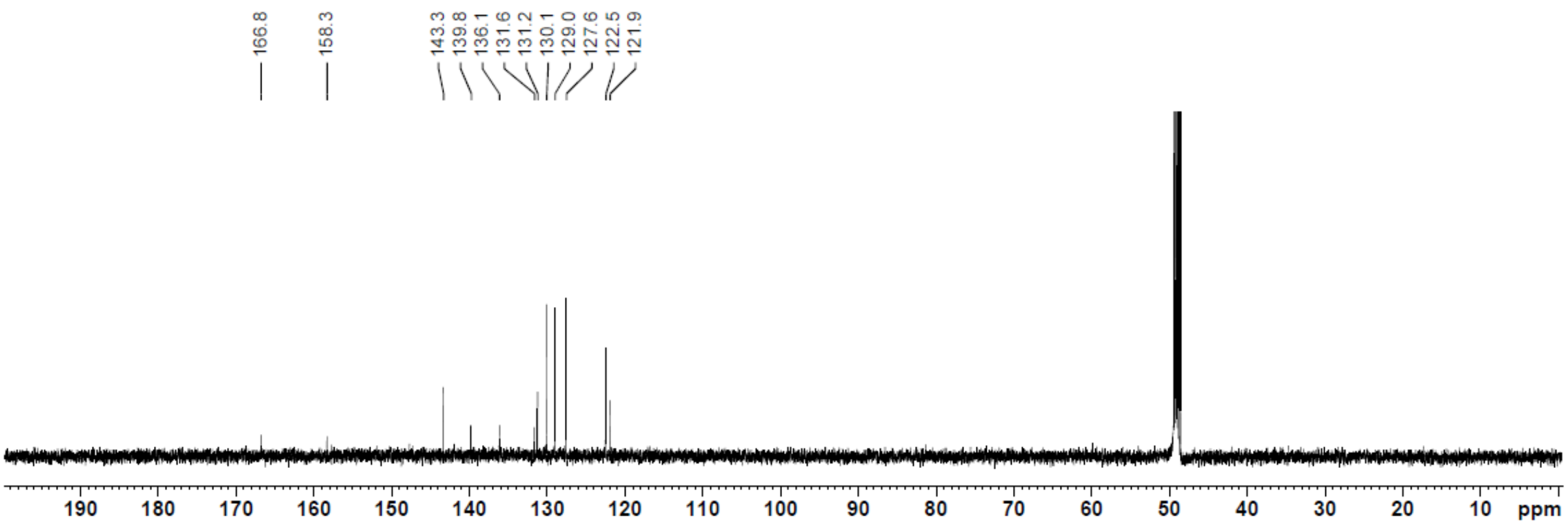

166.8 158.3 143.3 139.8 136.1 131.6 131.2 130.1 129.0 127.6 122.5 121.9
190 180 170 160 150 140 130 120 110 100 90 80 70 60 50 40 30 20 10 ppm

Compound **29**

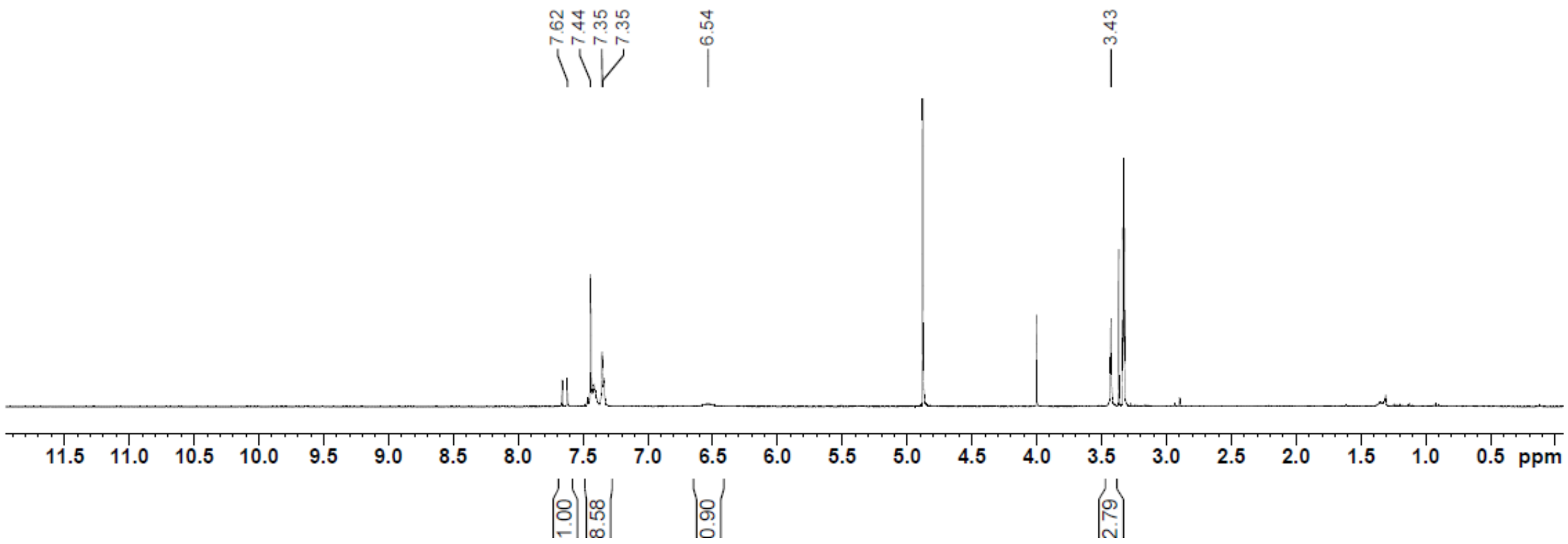
7.62
7.44
7.35
7.35
6.54
3.43
11.5 11.0 10.5 10.0 9.5 9.0 8.5 8.0 7.5 7.0 6.5 6.0 5.5 5.0 4.5 4.0 3.5 3.0 2.5 2.0 1.5 1.0 0.5 ppm
1.00
8.58
0.90
2.79

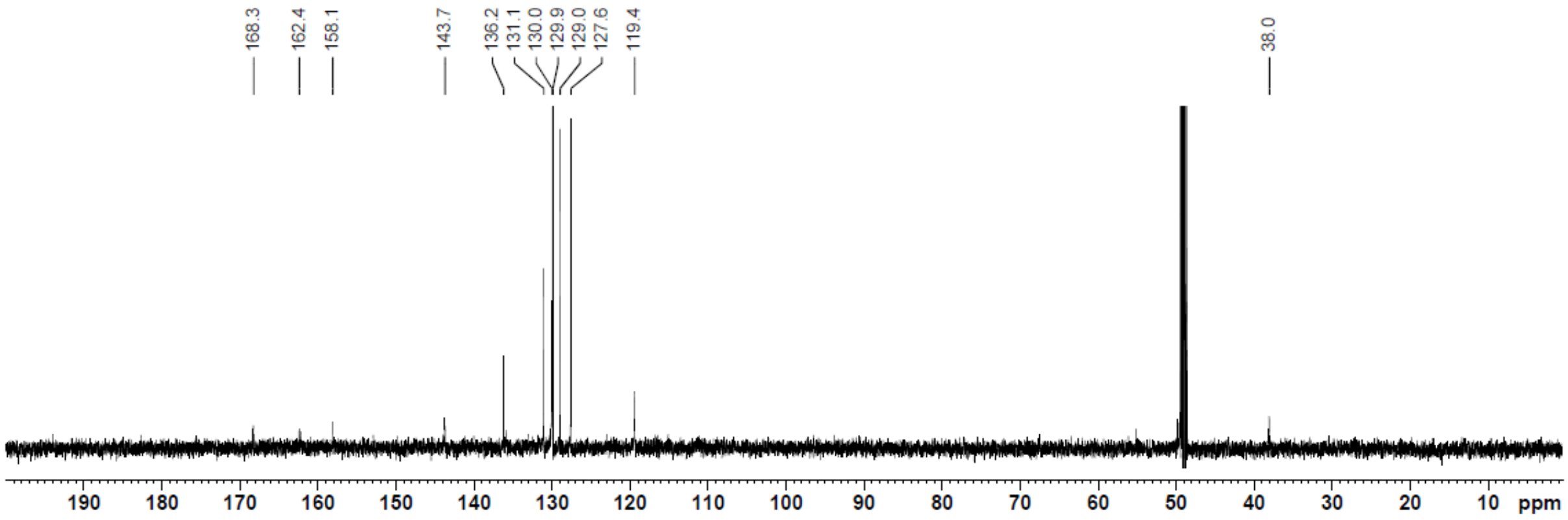
168.3
162.4
158.1
143.7
136.2
131.1
130.0
129.9
129.0
127.6
119.4
38.0
190 180 170 160 150 140 130 120 110 100 90 80 70 60 50 40 30 20 10 ppm

Compound **30**

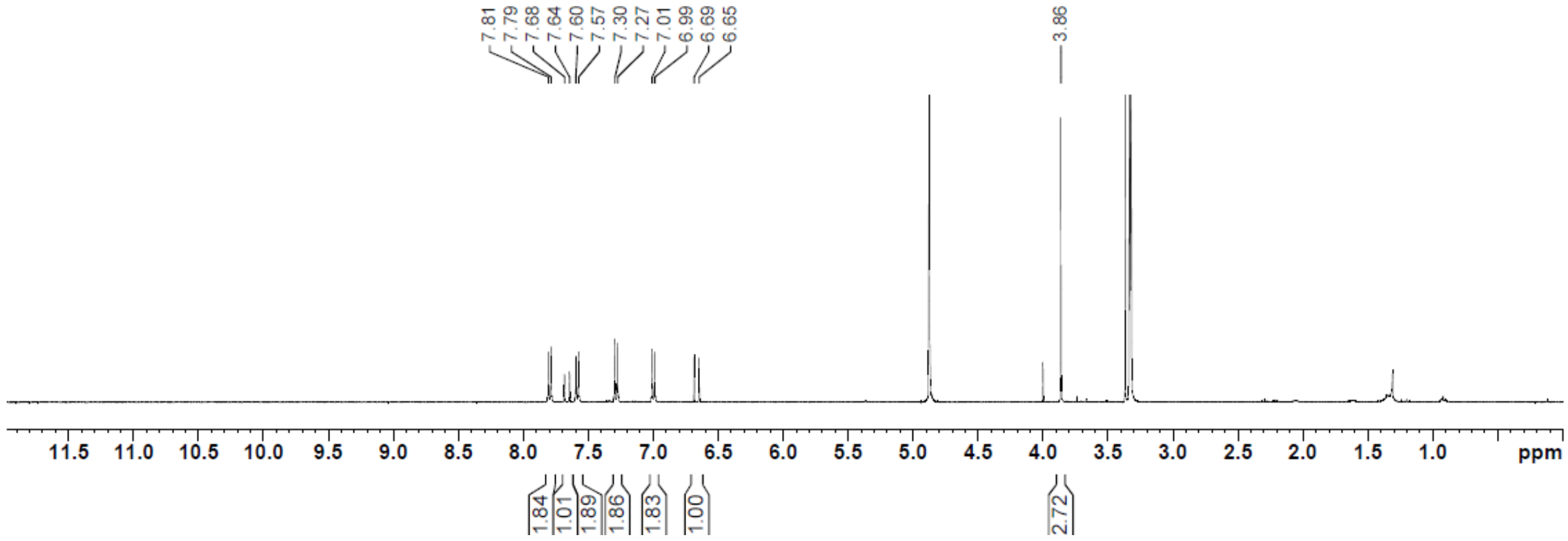
7.81
7.79
7.68
7.64
7.60
7.57
7.30
7.27
7.01
6.99
6.69
6.65
3.86
11.5 11.0 10.5 10.0 9.5 9.0 8.5 8.0 7.5 7.0 6.5 6.0 5.5 5.0 4.5 4.0 3.5 3.0 2.5 2.0 1.5 1.0 ppm
1.84
1.01
1.89
1.86
1.83
1.00
2.72

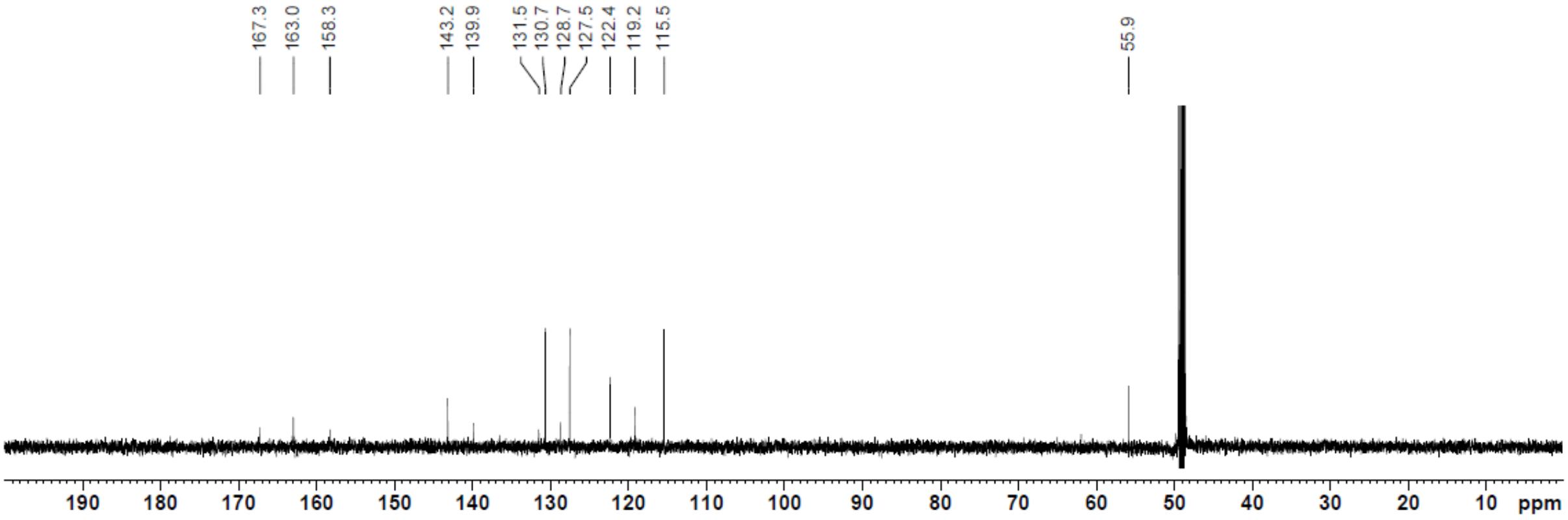
167.3
163.0
158.3
143.2
139.9
131.5
130.7
128.7
127.5
122.4
119.2
115.5
55.9
190 180 170 160 150 140 130 120 110 100 90 80 70 60 50 40 30 20 10 ppm

Compound **31**

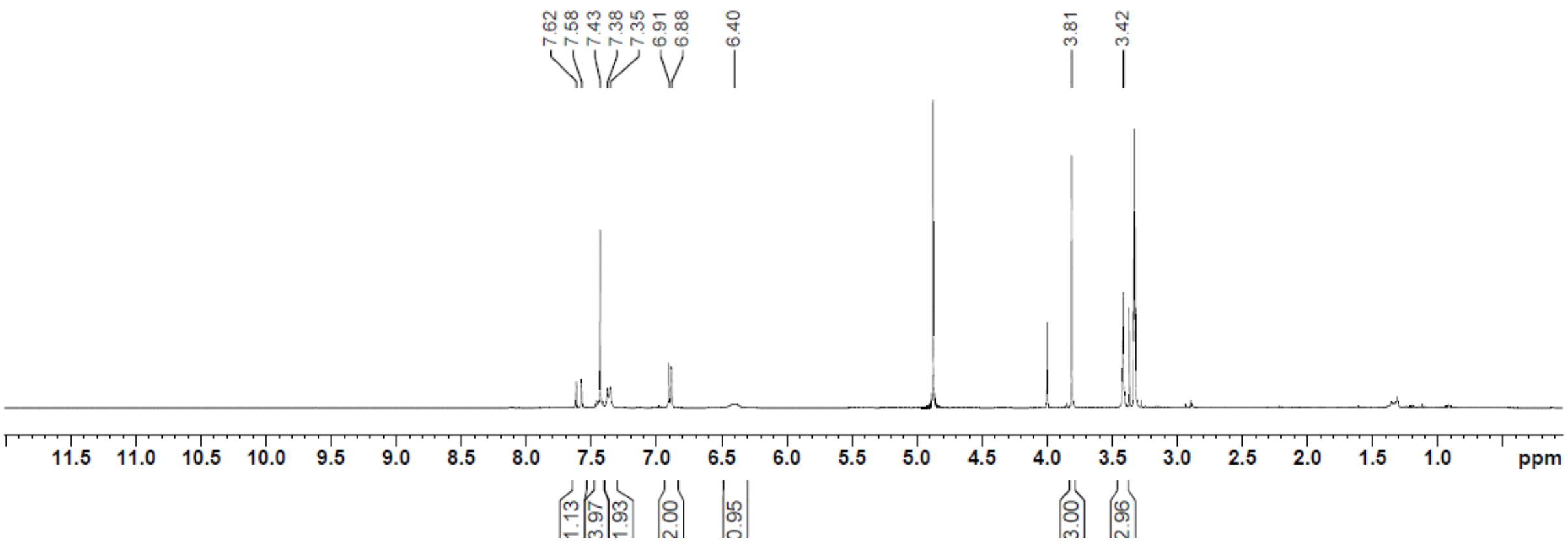
7.62
7.58
7.43
7.38
7.35
6.91
6.88
6.40
3.81
3.42
11.5 11.0 10.5 10.0 9.5 9.0 8.5 8.0 7.5 7.0 6.5 6.0 5.5 5.0 4.5 4.0 3.5 3.0 2.5 2.0 1.5 1.0 ppm
1.13
3.97
1.93
2.00
0.95
3.00
2.96

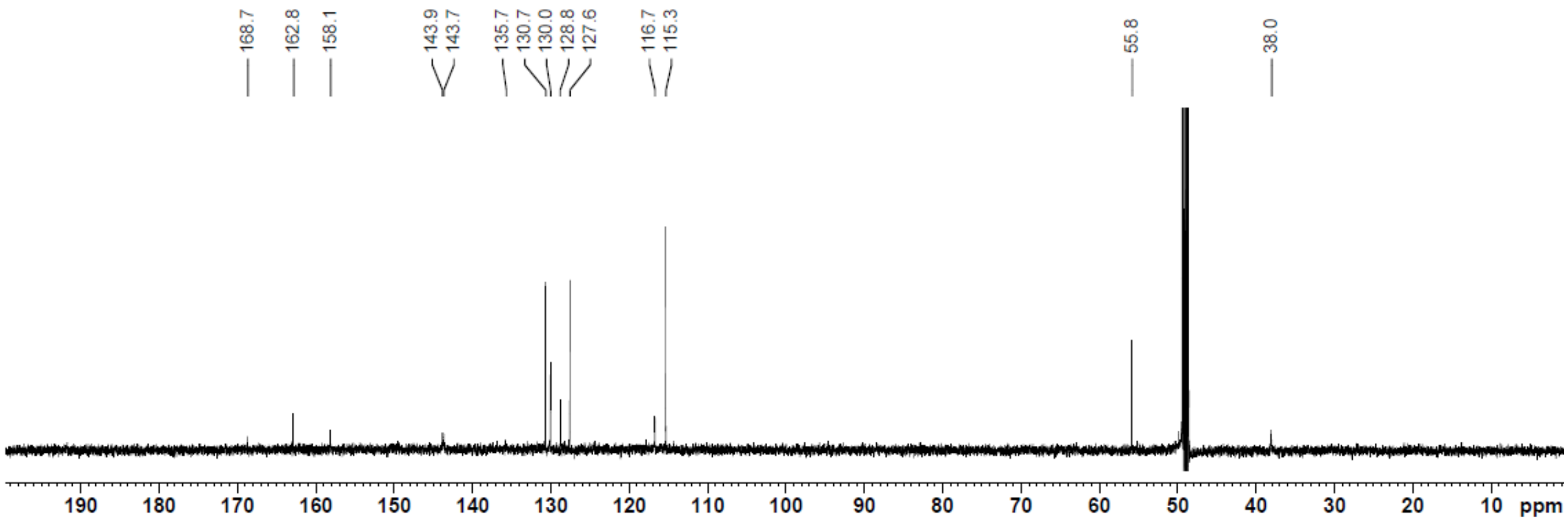
168.7
162.8
158.1
143.9
143.7
135.7
130.7
130.0
128.8
127.6
116.7
115.3
55.8
38.0
190 180 170 160 150 140 130 120 110 100 90 80 70 60 50 40 30 20 10 ppm

Compound **32**

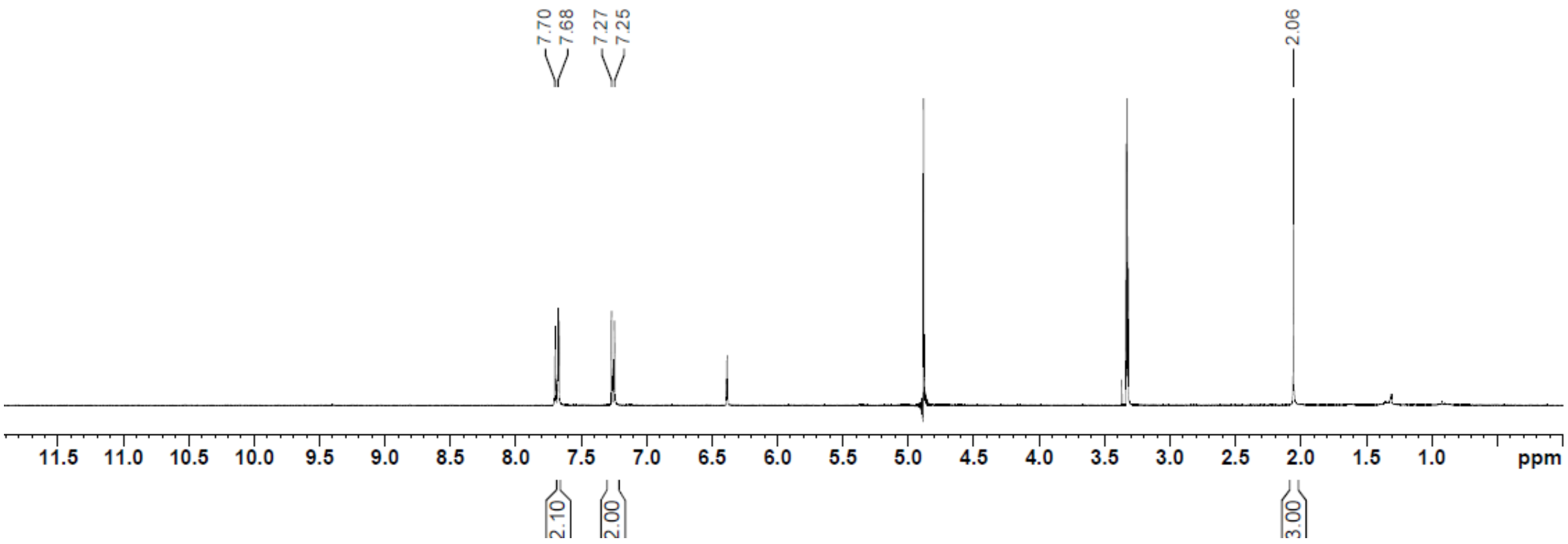
7.70
7.68
7.27
7.25
2.06
11.5 11.0 10.5 10.0 9.5 9.0 8.5 8.0 7.5 7.0 6.5 6.0 5.5 5.0 4.5 4.0 3.5 3.0 2.5 2.0 1.5 1.0 ppm
2.10
2.00
3.00

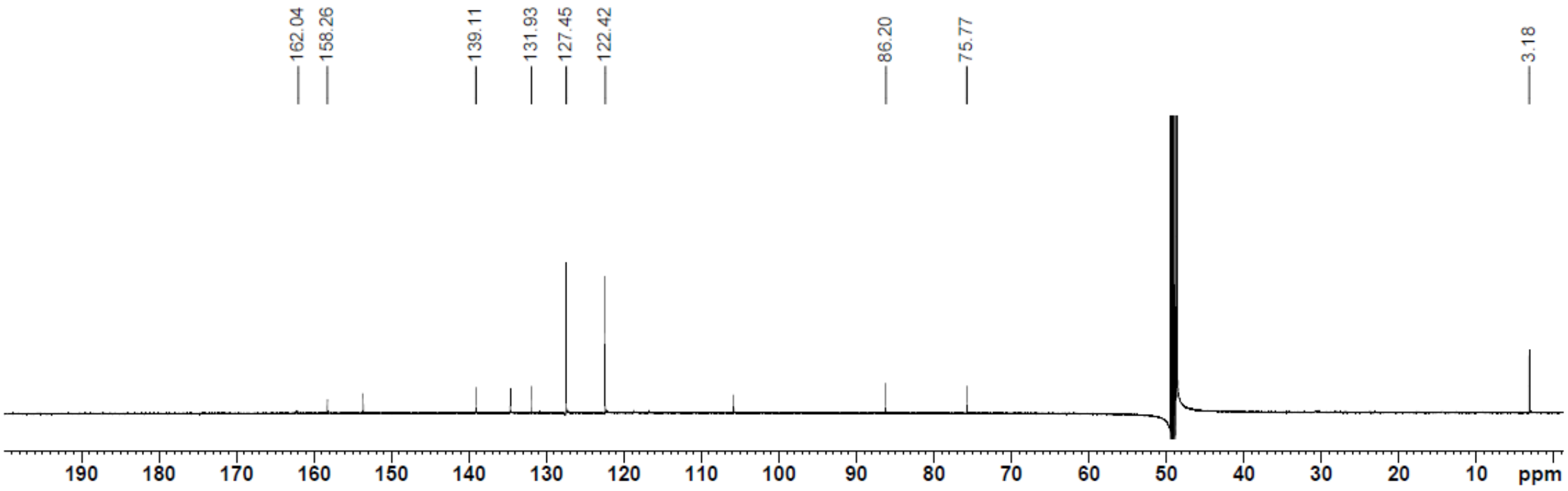
162.04
158.26
139.11
131.93
127.45
122.42
86.20
75.77
3.18
190 180 170 160 150 140 130 120 110 100 90 80 70 60 50 40 30 20 10 ppm

Compound **33**

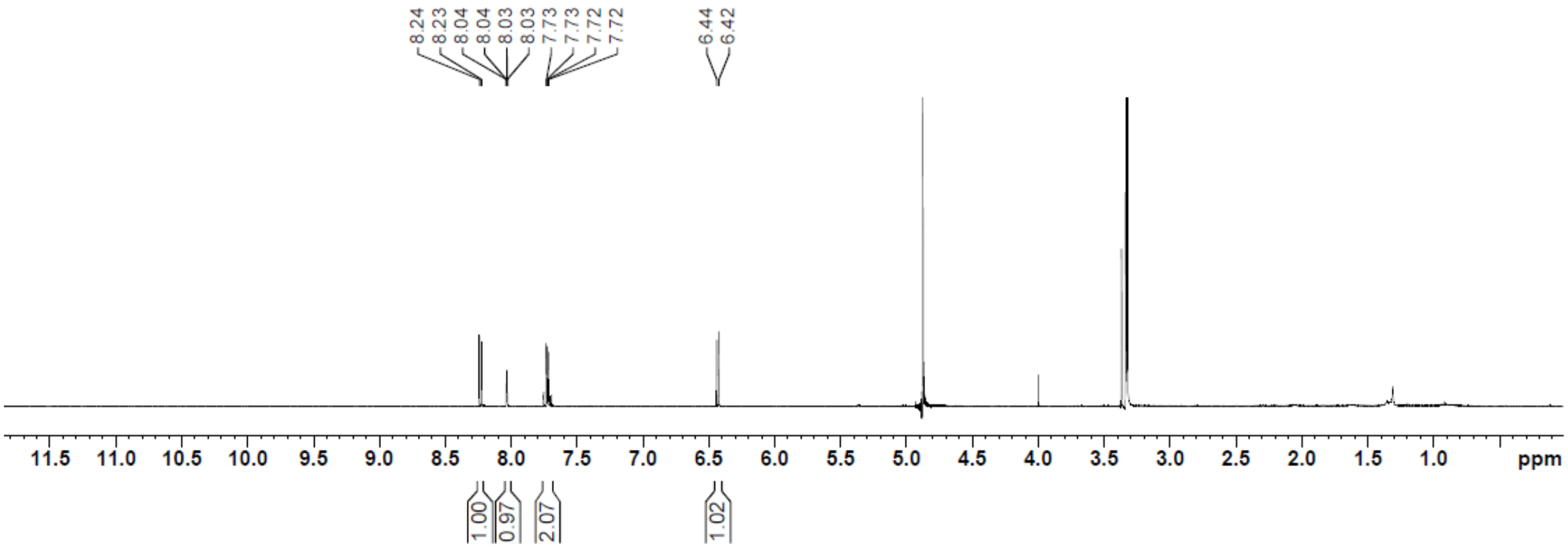

8.24
8.23
8.04
8.04
8.03
8.03
7.73
7.73
7.72
7.72
6.44
6.42
11.5 11.0 10.5 10.0 9.5 9.0 8.5 8.0 7.5 7.0 6.5 6.0 5.5 5.0 4.5 4.0 3.5 3.0 2.5 2.0 1.5 1.0 ppm
1.00
0.97
2.07
1.02

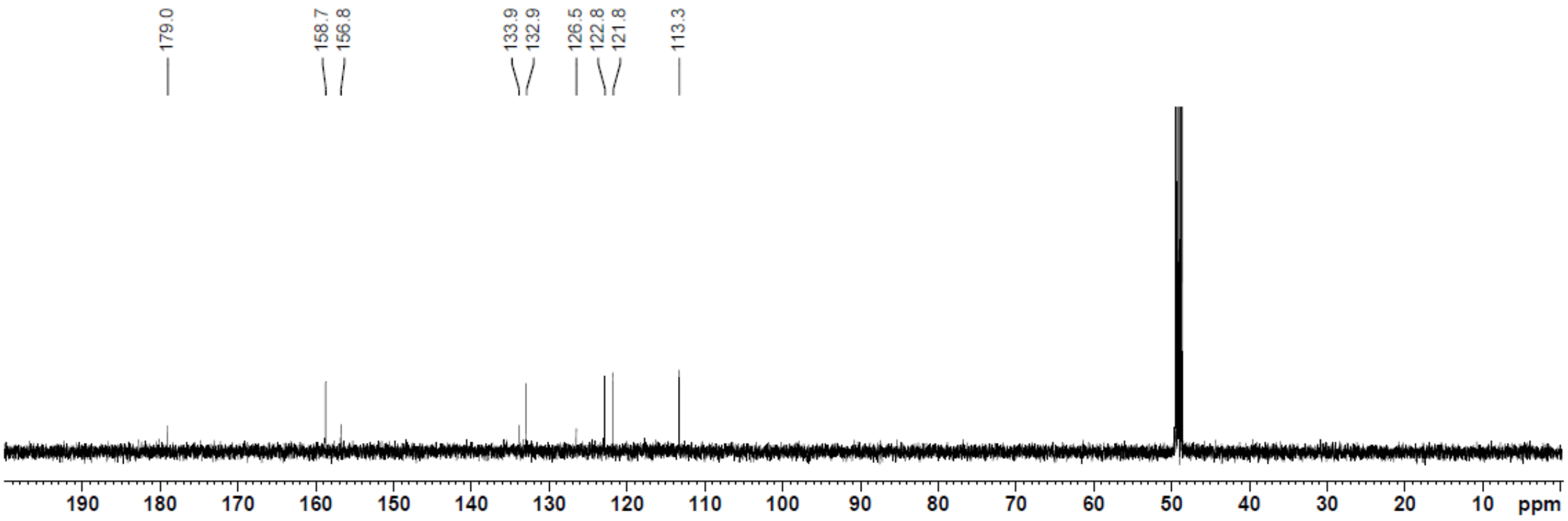

179.0
158.7
156.8
133.9
132.9
126.5
122.8
121.8
113.3
190 180 170 160 150 140 130 120 110 100 90 80 70 60 50 40 30 20 10 ppm

Compound **34**

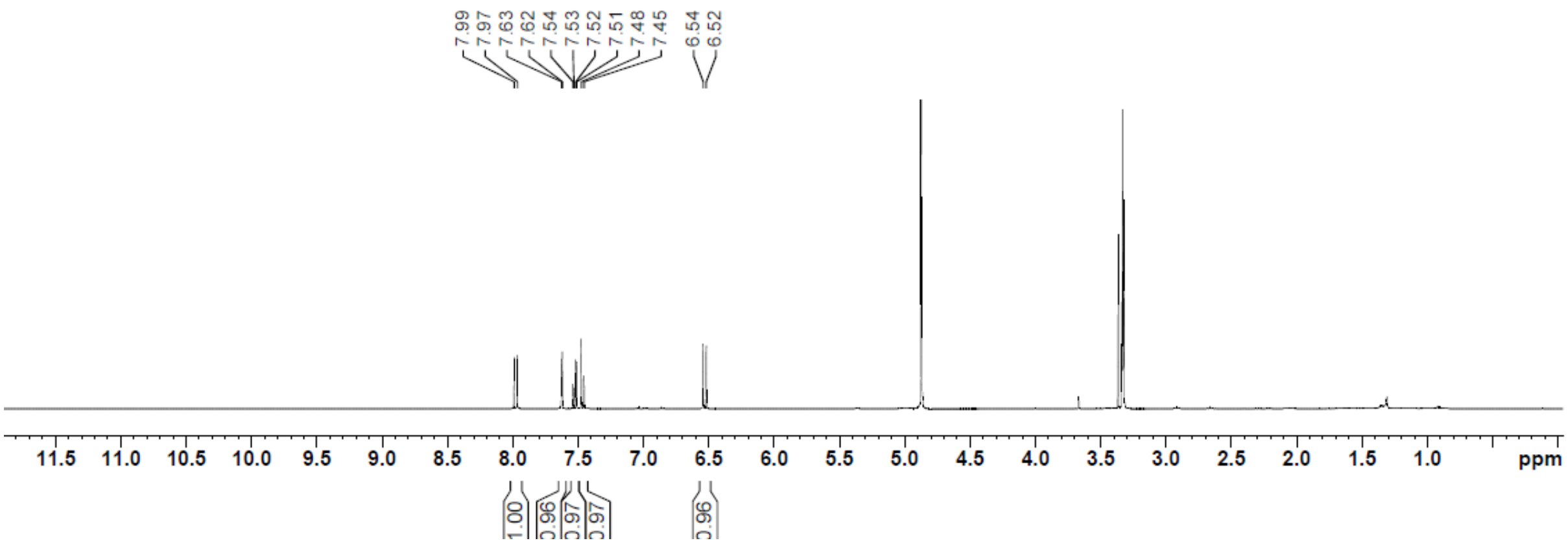
7.99
7.97
7.63
7.62
7.54
7.53
7.52
7.51
7.48
7.45
6.54
6.52
11.5 11.0 10.5 10.0 9.5 9.0 8.5 8.0 7.5 7.0 6.5 6.0 5.5 5.0 4.5 4.0 3.5 3.0 2.5 2.0 1.5 1.0 ppm
1.00
0.96
0.97
0.97
0.96

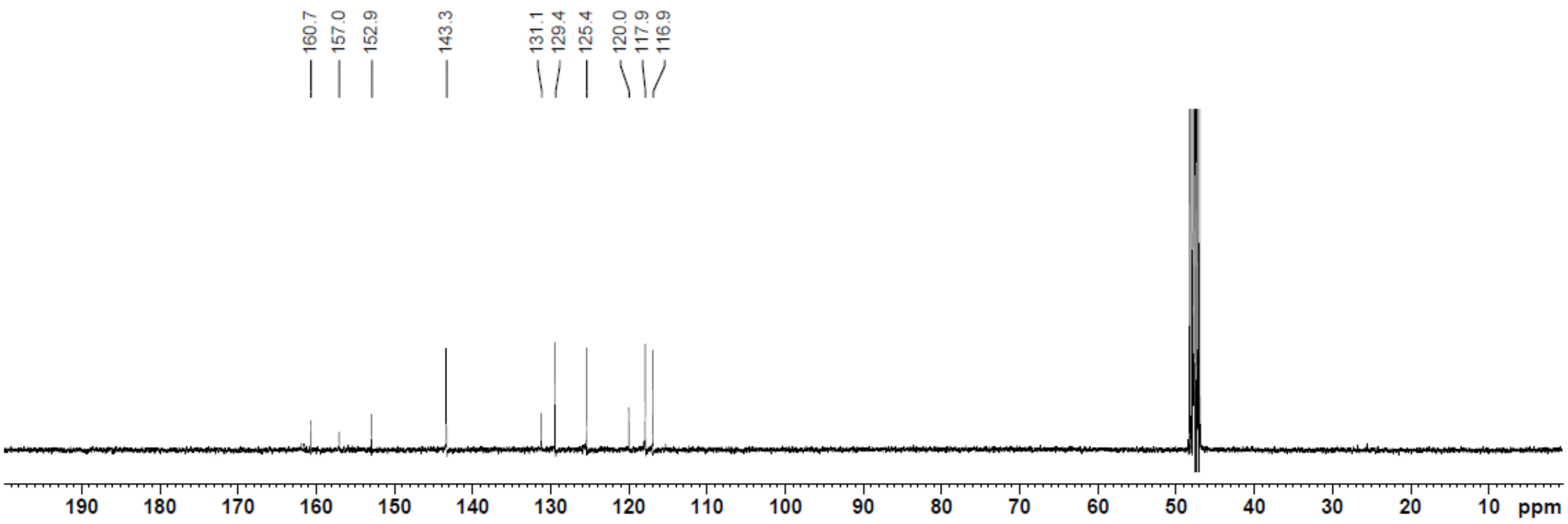
160.7
157.0
152.9
143.3
131.1
129.4
125.4
120.0
117.9
116.9
190 180 170 160 150 140 130 120 110 100 90 80 70 60 50 40 30 20 10 ppm

Compound **35**

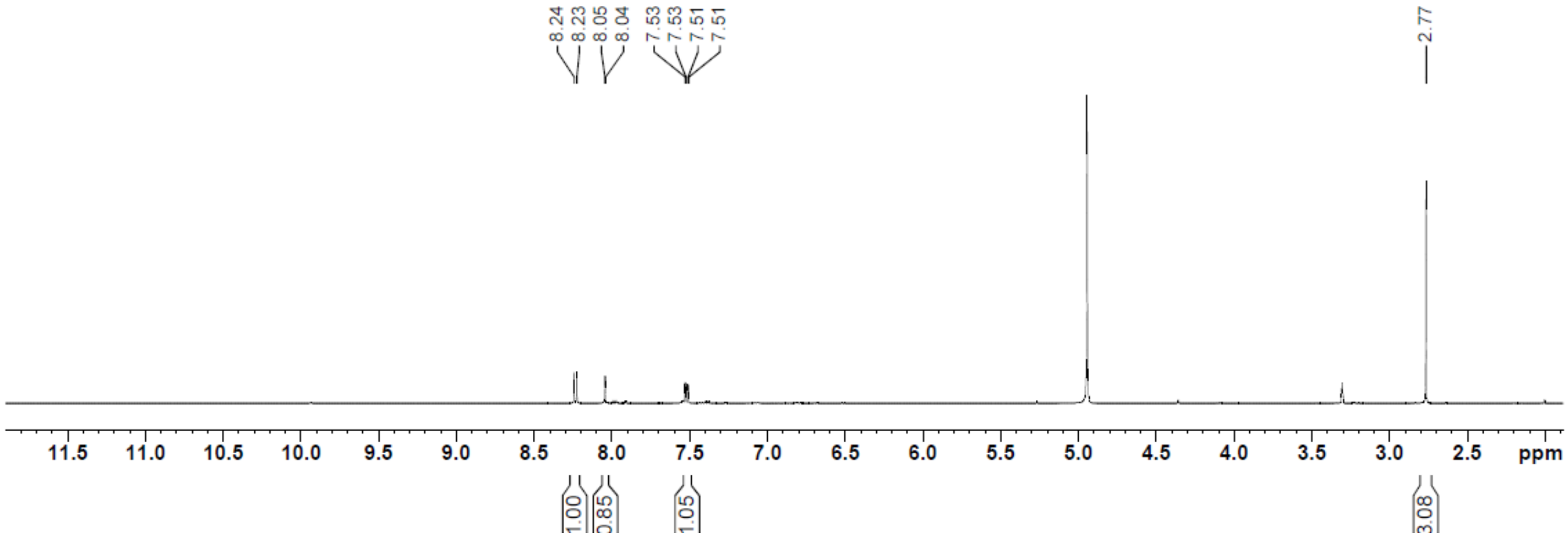

8.24
8.23
8.05
8.04
7.53
7.53
7.51
7.51
2.77
11.5 11.0 10.5 10.0 9.5 9.0 8.5 8.0 7.5 7.0 6.5 6.0 5.5 5.0 4.5 4.0 3.5 3.0 2.5 ppm
1.00
0.85
1.05
3.08

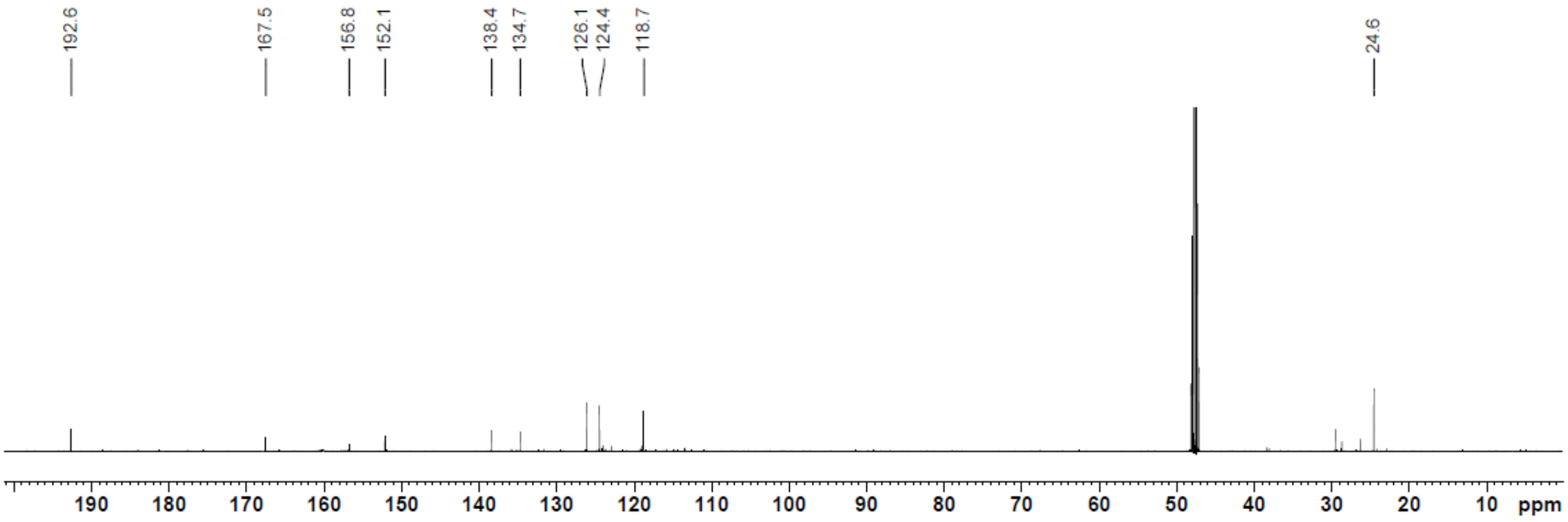

192.6
167.5
156.8
152.1
138.4
134.7
126.1
124.4
118.7
24.6
190 180 170 160 150 140 130 120 110 100 90 80 70 60 50 40 30 20 10 ppm

Compound **36**

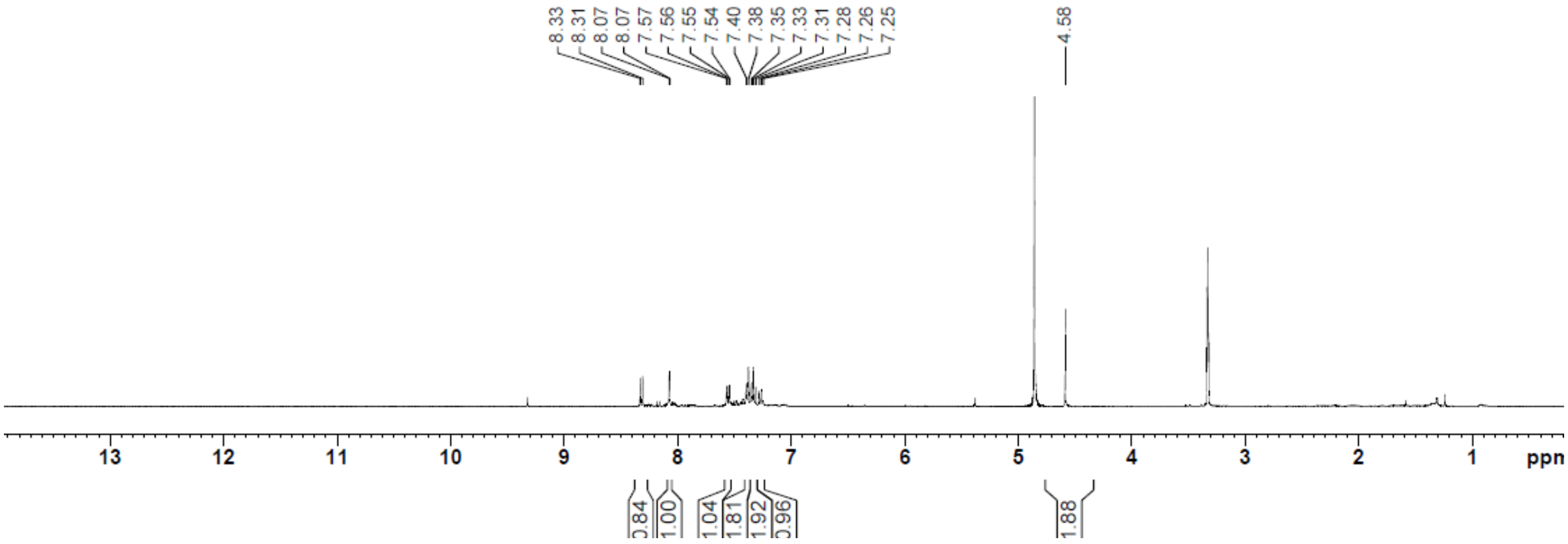
8.33
8.31
8.07
8.07
7.57
7.56
7.55
7.54
7.40
7.38
7.35
7.33
7.31
7.28
7.26
7.25
4.58
0.84
1.00
1.04
1.81
1.92
0.96
1.88
13
12
11
10
9
8
7
6
5
4
3
2
1
ppm

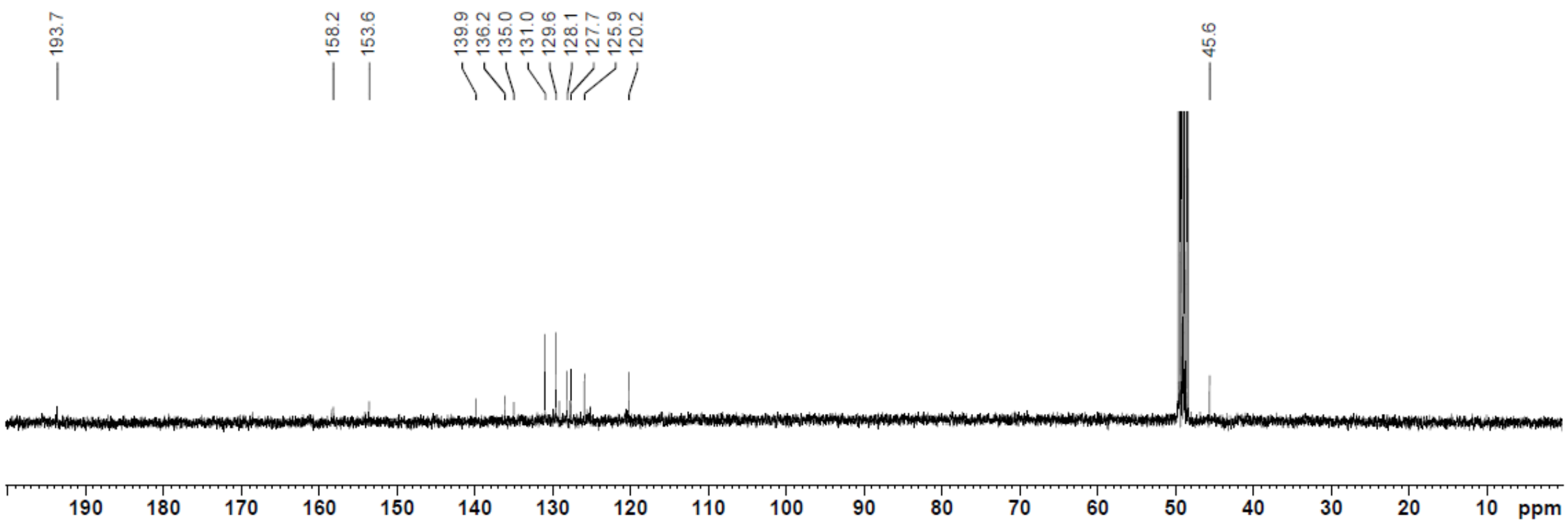
193.7
158.2
153.6
139.9
136.2
135.0
131.0
129.6
128.1
127.7
125.9
120.2
45.6
190
180
170
160
150
140
130
120
110
100
90
80
70
60
50
40
30
20
10
ppm

Compound **37**

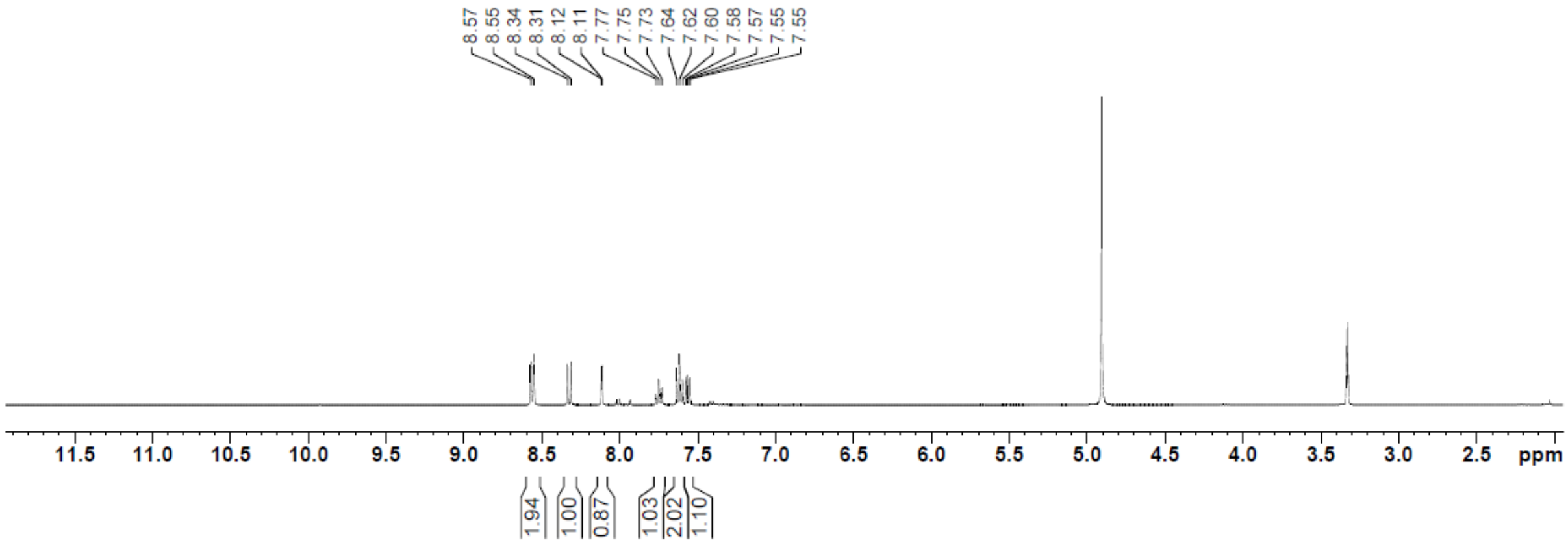
8.57
8.55
8.34
8.31
8.12
8.11
7.77
7.75
7.73
7.64
7.62
7.60
7.58
7.57
7.55
7.55
11.5 11.0 10.5 10.0 9.5 9.0 8.5 8.0 7.5 7.0 6.5 6.0 5.5 5.0 4.5 4.0 3.5 3.0 2.5 ppm
1.94
1.00
0.87
1.03
2.02
1.10

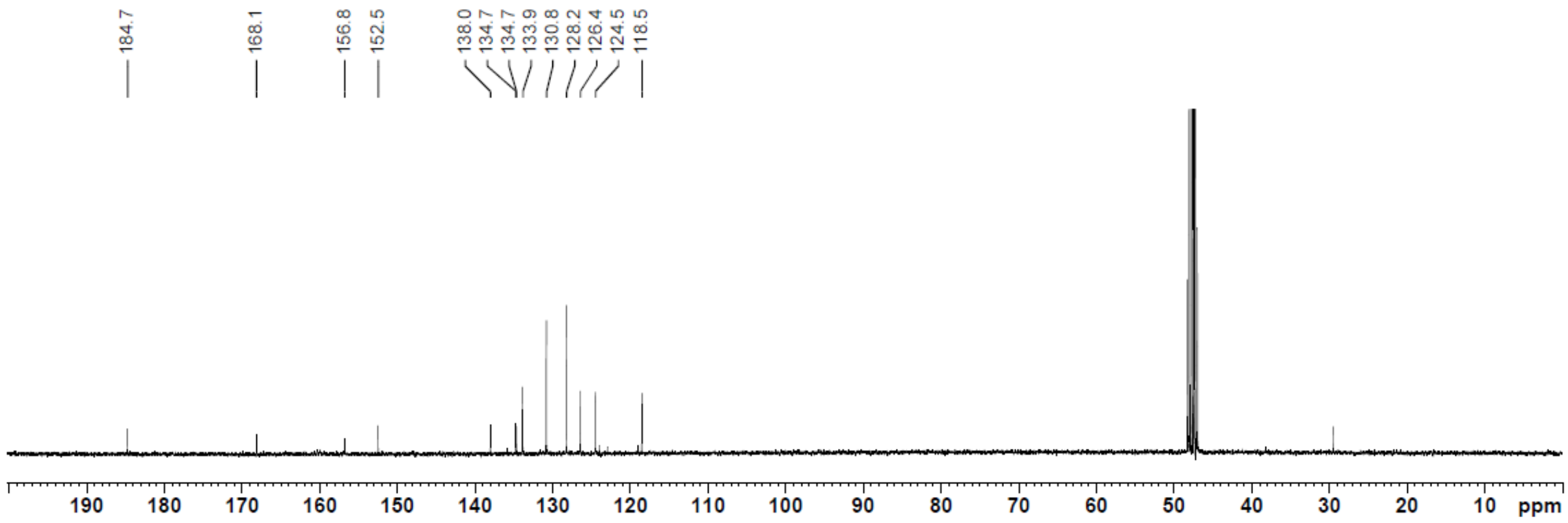
184.7
168.1
156.8
152.5
138.0
134.7
134.7
133.9
130.8
128.2
126.4
124.5
118.5
190 180 170 160 150 140 130 120 110 100 90 80 70 60 50 40 30 20 10 ppm

Compound **38**

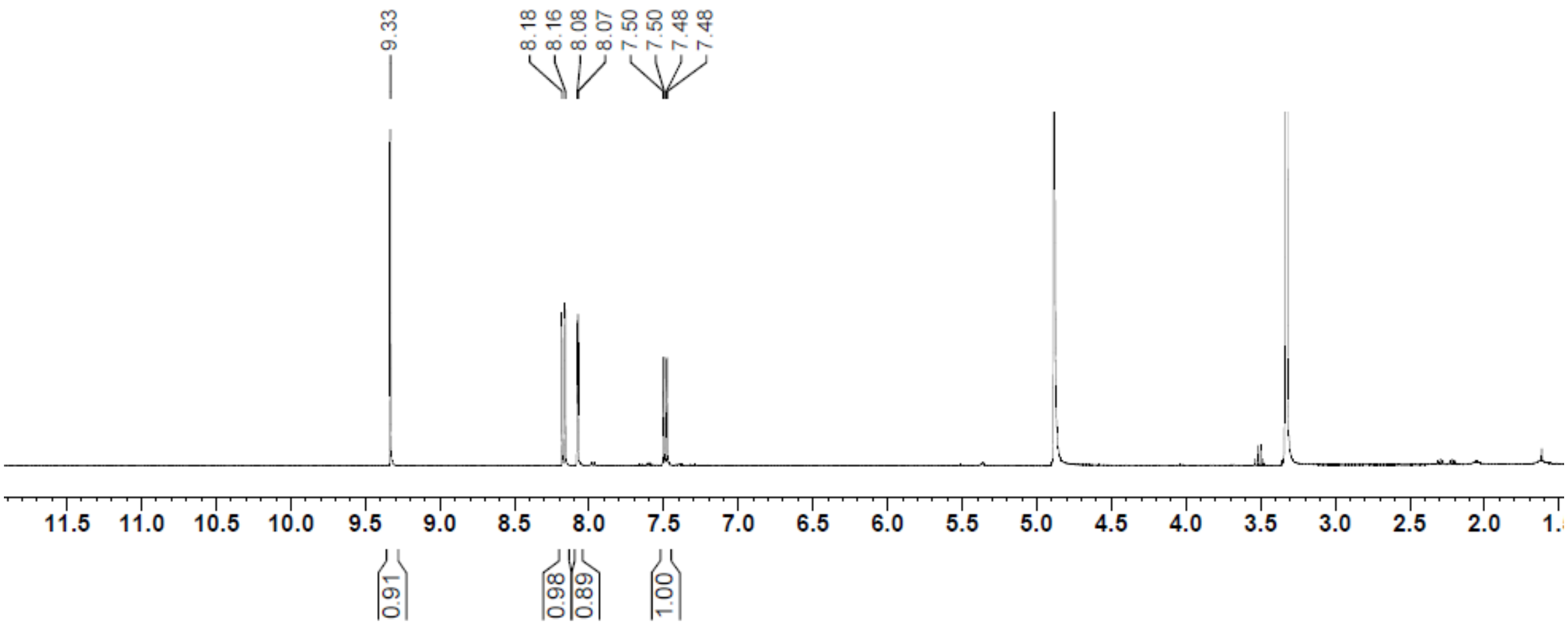
9.33
8.18
8.16
8.08
8.07
7.50
7.50
7.48
7.48
11.5 11.0 10.5 10.0 9.5 9.0 8.5 8.0 7.5 7.0 6.5 6.0 5.5 5.0 4.5 4.0 3.5 3.0 2.5 2.0
0.91
0.98
0.89
1.00

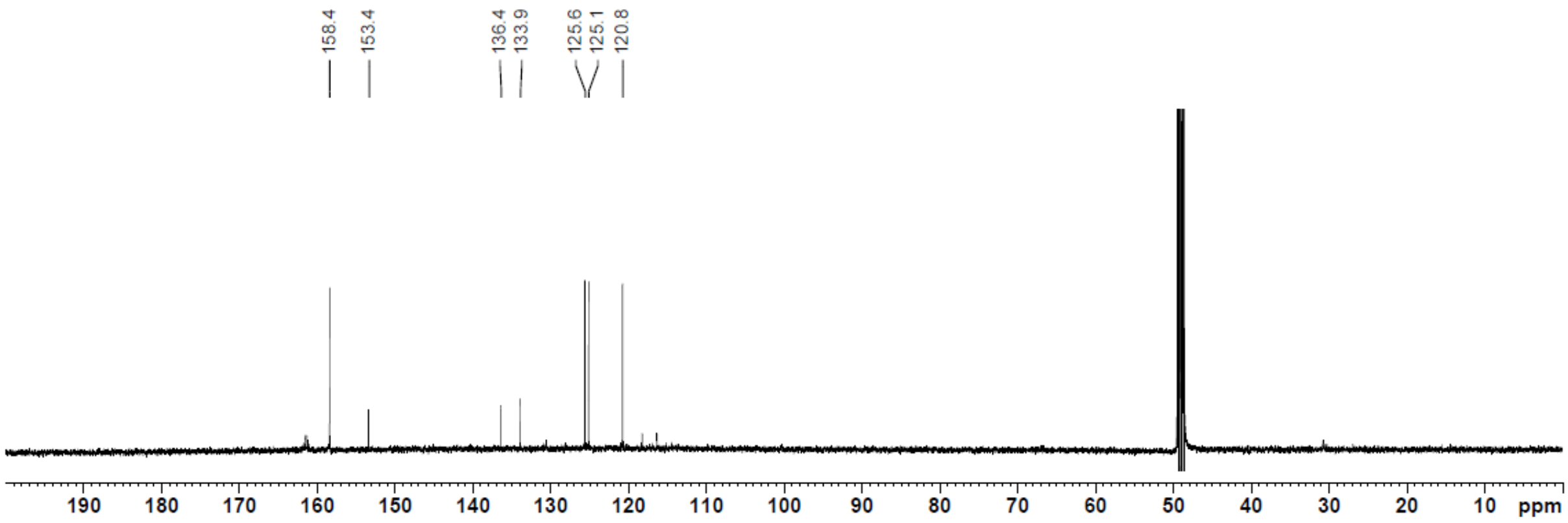
158.4
153.4
136.4
133.9
125.6
125.1
120.8
190 180 170 160 150 140 130 120 110 100 90 80 70 60 50 40 30 20 10 ppm

Compound **39**

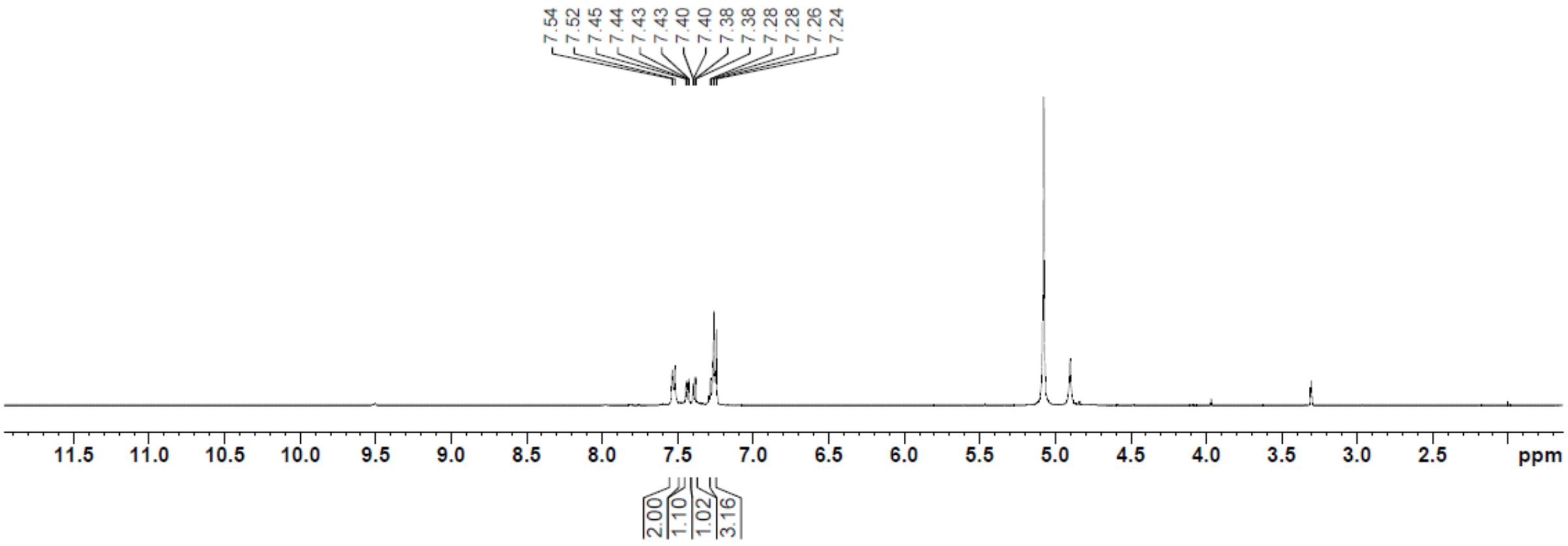
7.54
7.52
7.45
7.44
7.43
7.43
7.40
7.40
7.38
7.38
7.28
7.28
7.26
7.24
2.00
1.10
1.02
3.16
11.5
11.0
10.5
10.0
9.5
9.0
8.5
8.0
7.5
7.0
6.5
6.0
5.5
5.0
4.5
4.0
3.5
3.0
2.5
ppm

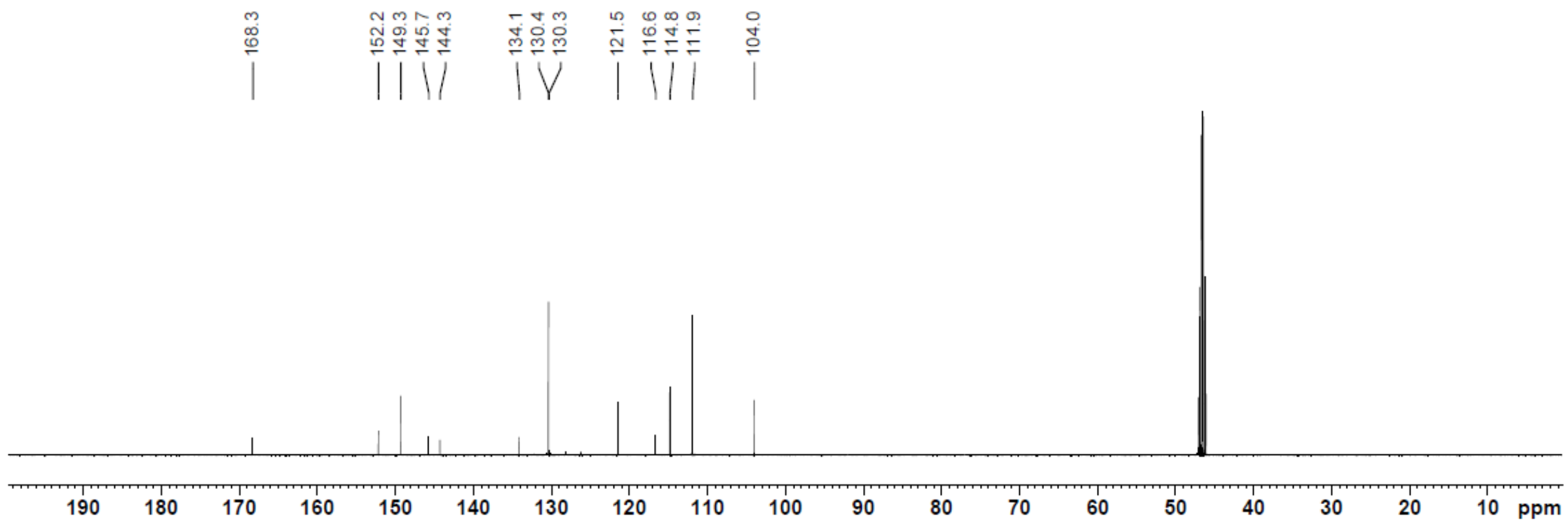
168.3
152.2
149.3
145.7
144.3
134.1
130.4
130.3
121.5
116.6
114.8
111.9
104.0
190
180
170
160
150
140
130
120
110
100
90
80
70
60
50
40
30
20
10
ppm

Compound **40**

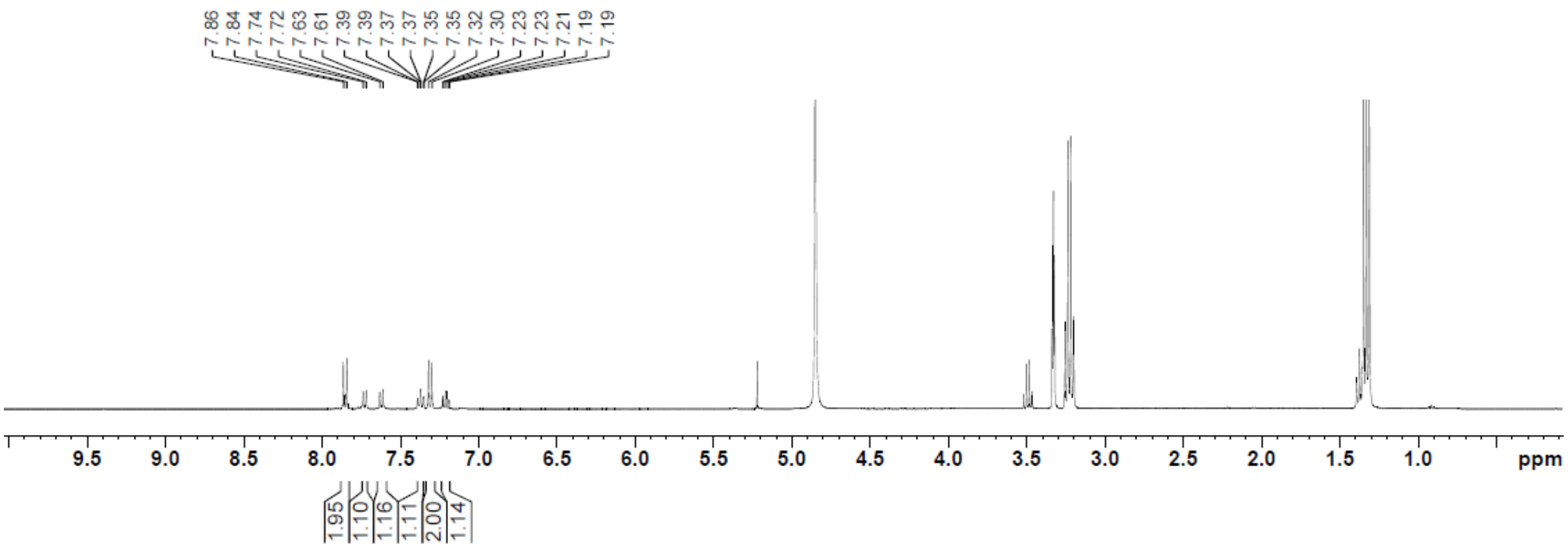
7.86
7.84
7.74
7.72
7.63
7.61
7.39
7.39
7.37
7.37
7.35
7.35
7.32
7.30
7.23
7.23
7.21
7.19
7.19
9.5 9.0 8.5 8.0 7.5 7.0 6.5 6.0 5.5 5.0 4.5 4.0 3.5 3.0 2.5 2.0 1.5 1.0 ppm
1.95
1.10
1.16
1.11
2.00
1.14

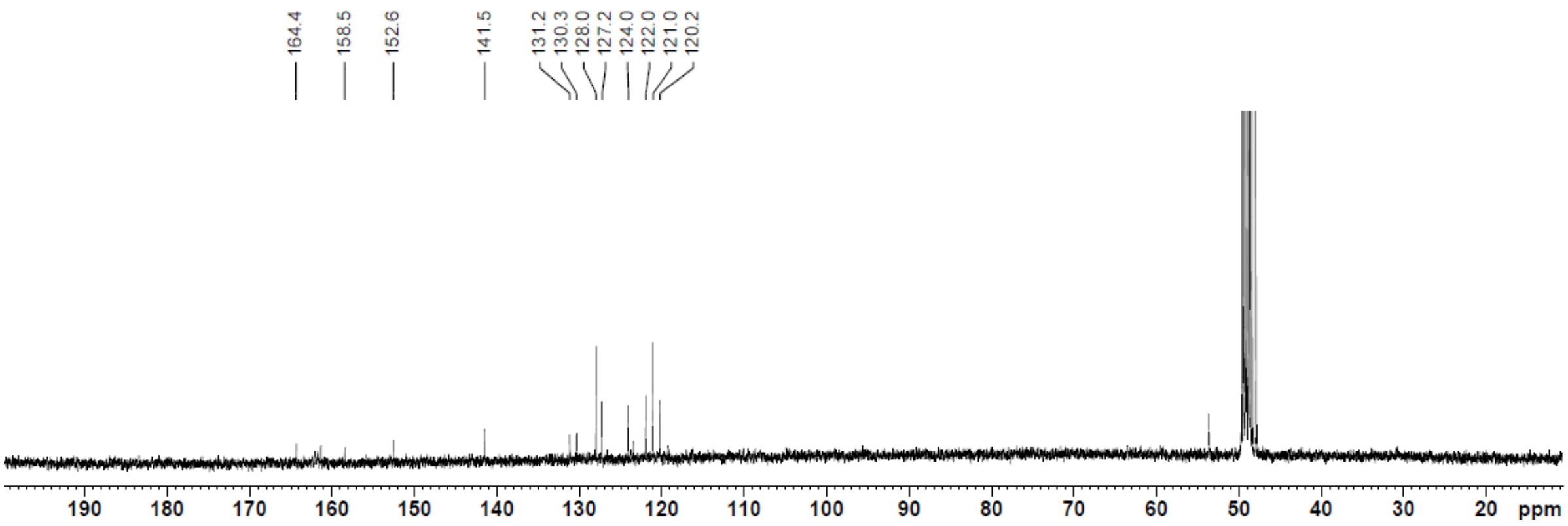
164.4
158.5
152.6
141.5
131.2
130.3
128.0
127.2
124.0
122.0
121.0
120.2
190 180 170 160 150 140 130 120 110 100 90 80 70 60 50 40 30 20 ppm

Compound **Boc-1**

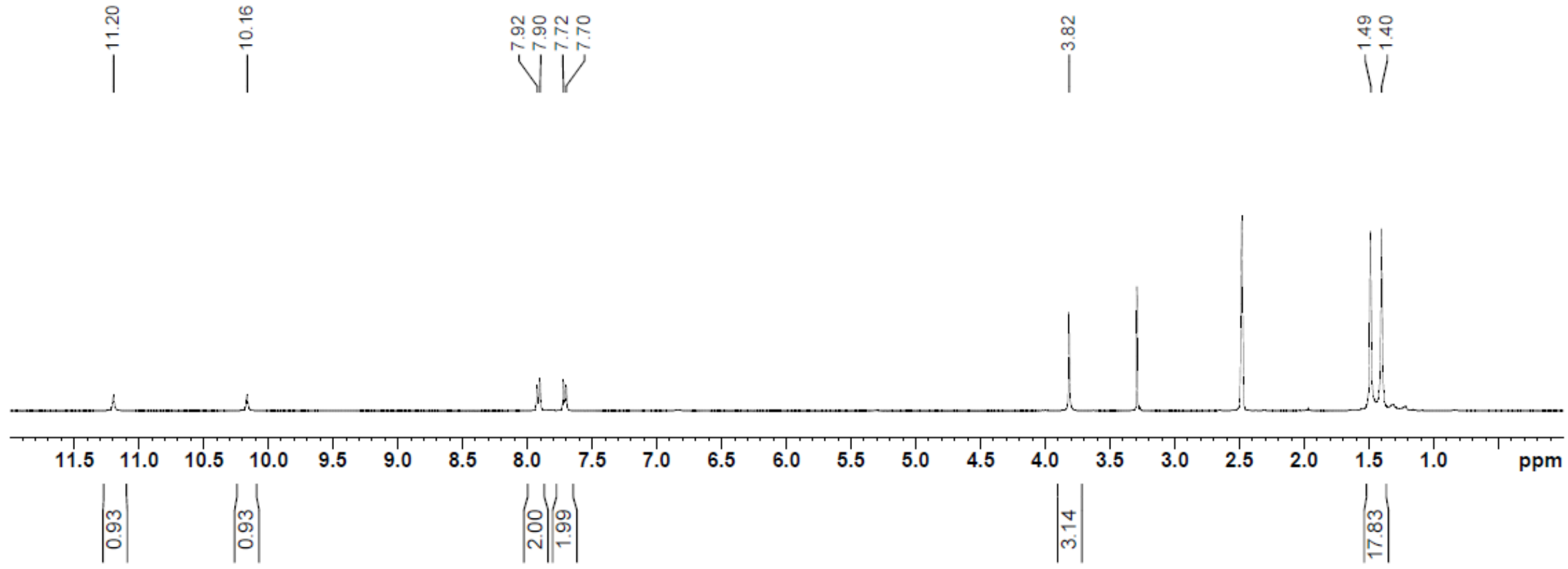

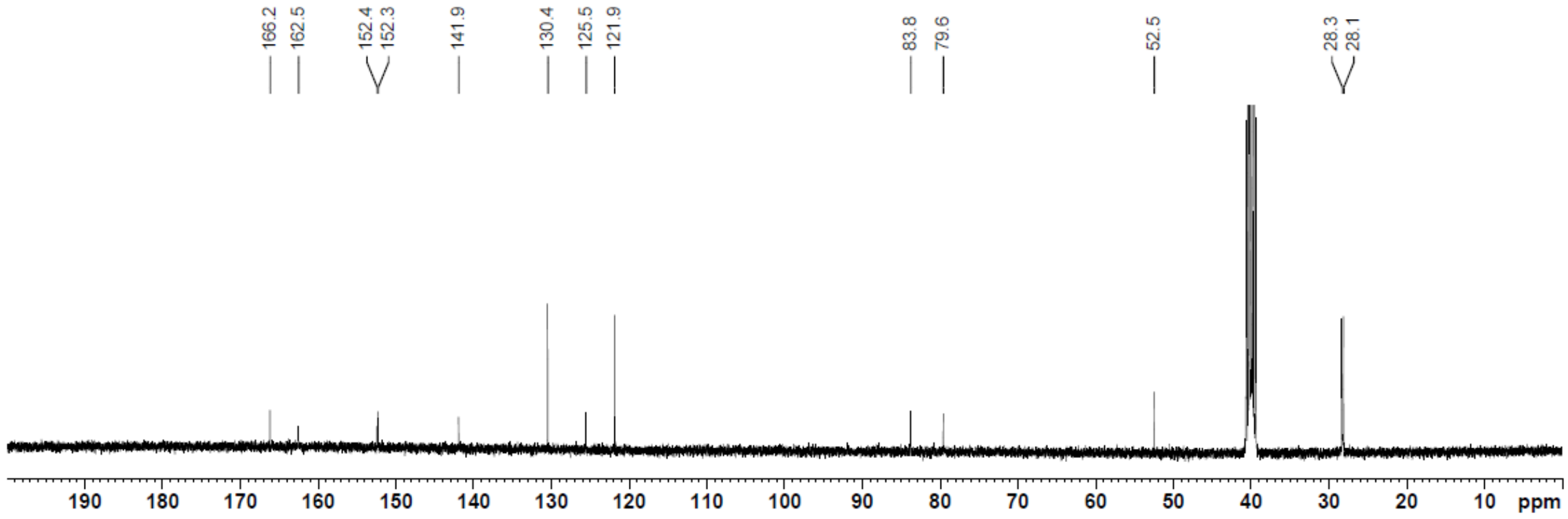

Octyl 4-aminobenzoate

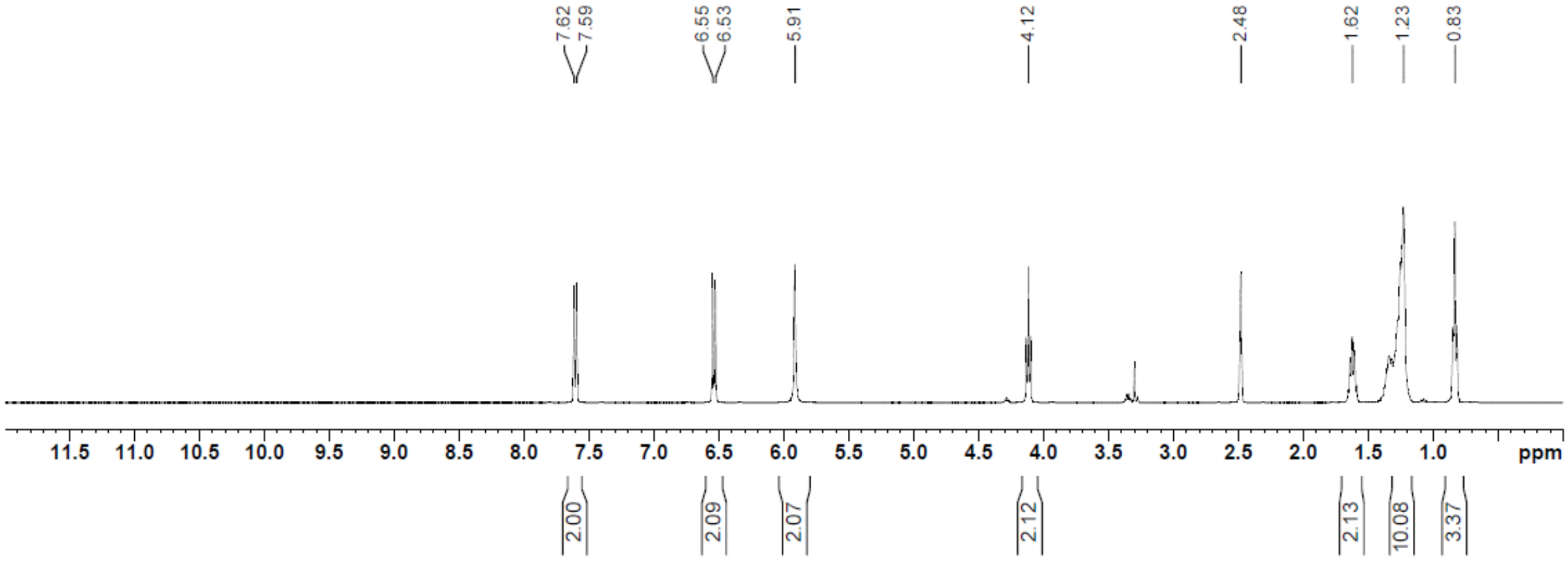

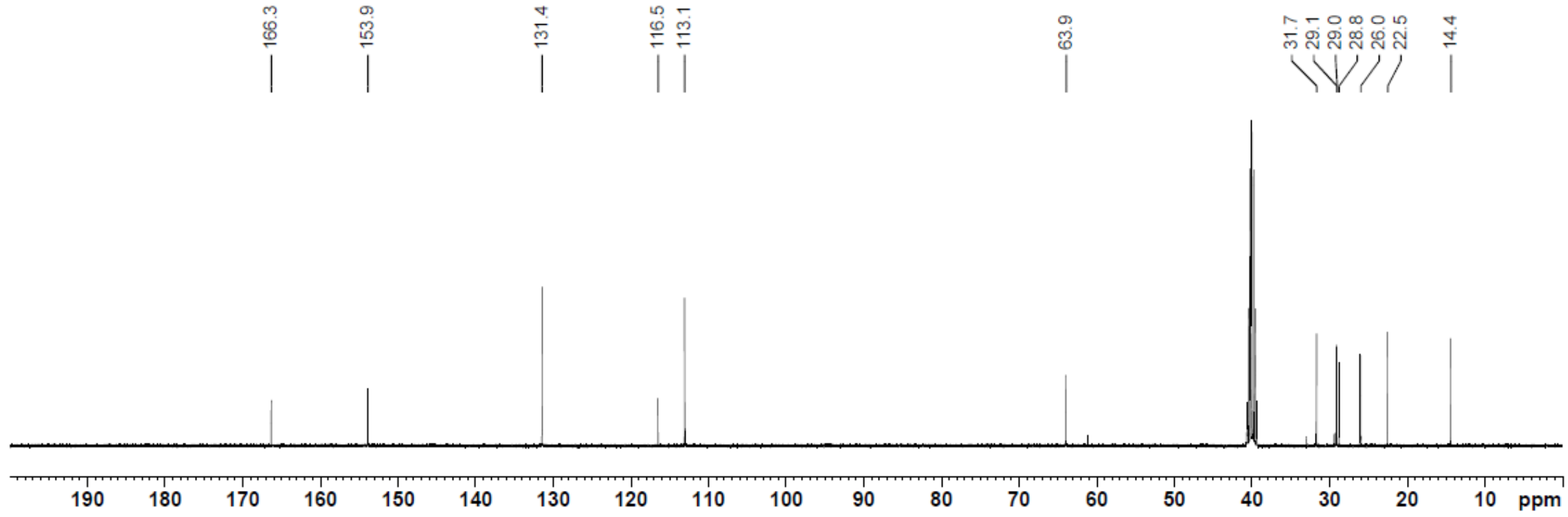

Compound **Boc-2**

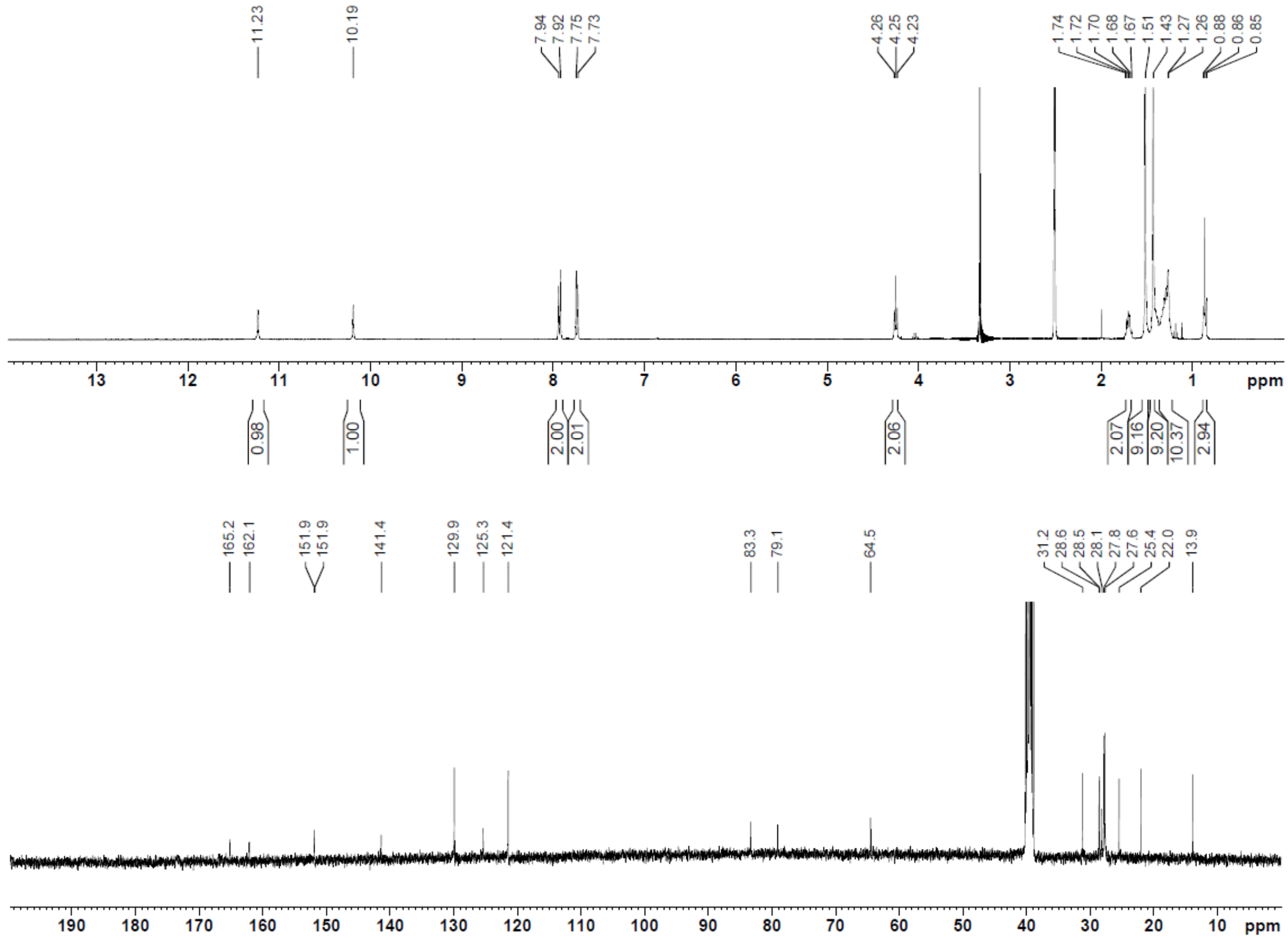

Compound **42**

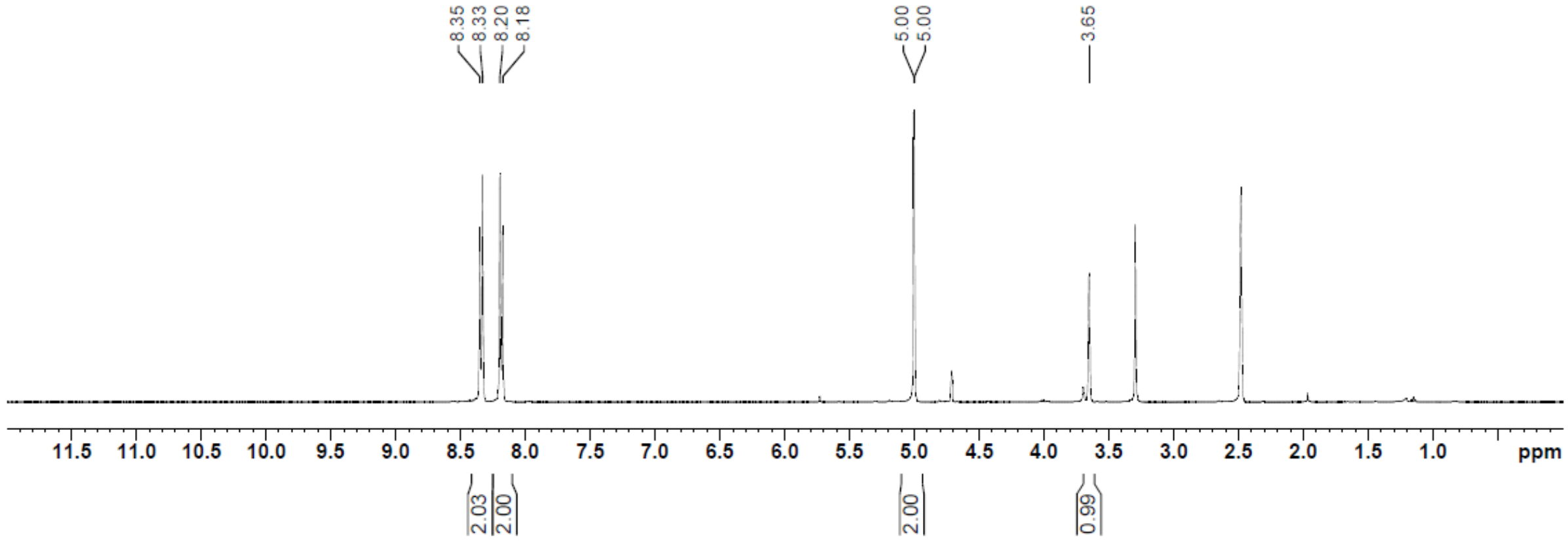
8.35
8.33
8.20
8.18
5.00
5.00
3.65
11.5 11.0 10.5 10.0 9.5 9.0 8.5 8.0 7.5 7.0 6.5 6.0 5.5 5.0 4.5 4.0 3.5 3.0 2.5 2.0 1.5 1.0 ppm
2.03
2.00
2.00
0.99

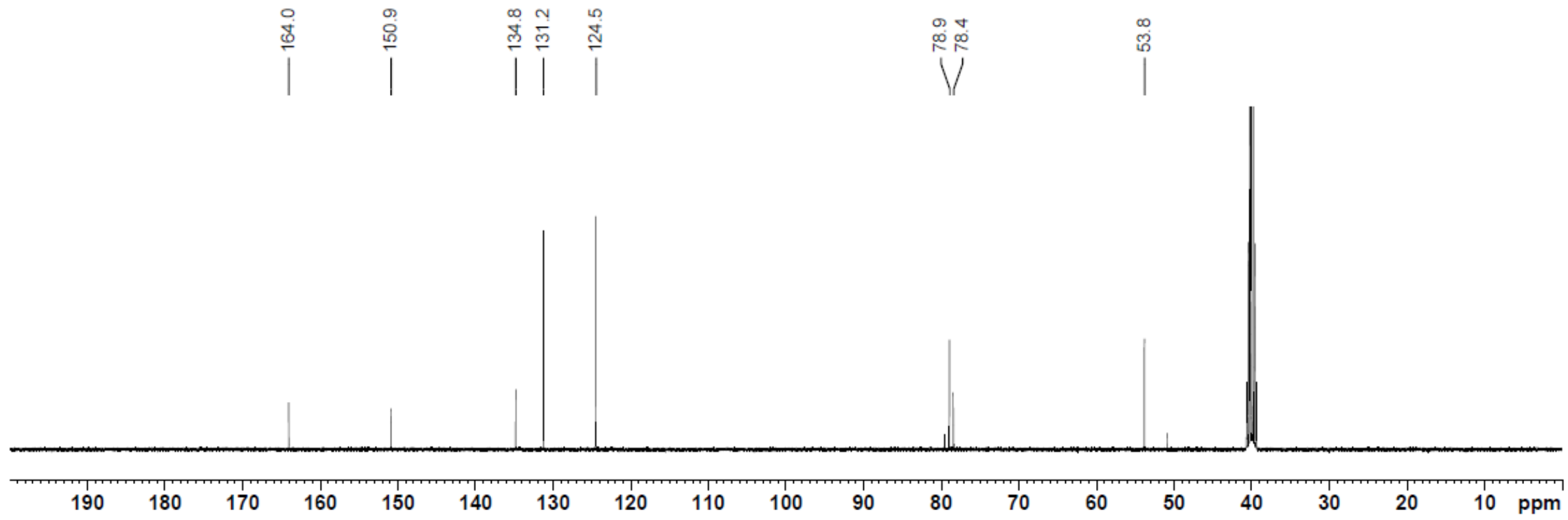
164.0
150.9
134.8
131.2
124.5
78.9
78.4
53.8
190 180 170 160 150 140 130 120 110 100 90 80 70 60 50 40 30 20 10 ppm

Compound **Boc-4**

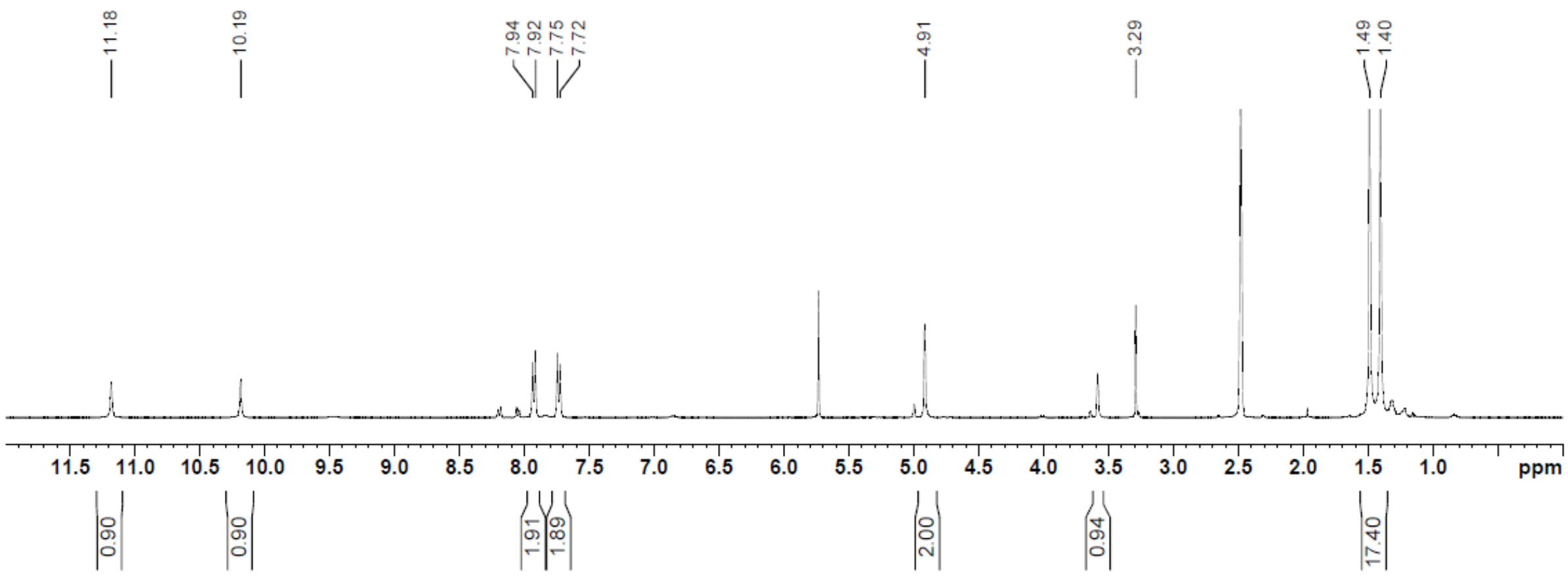

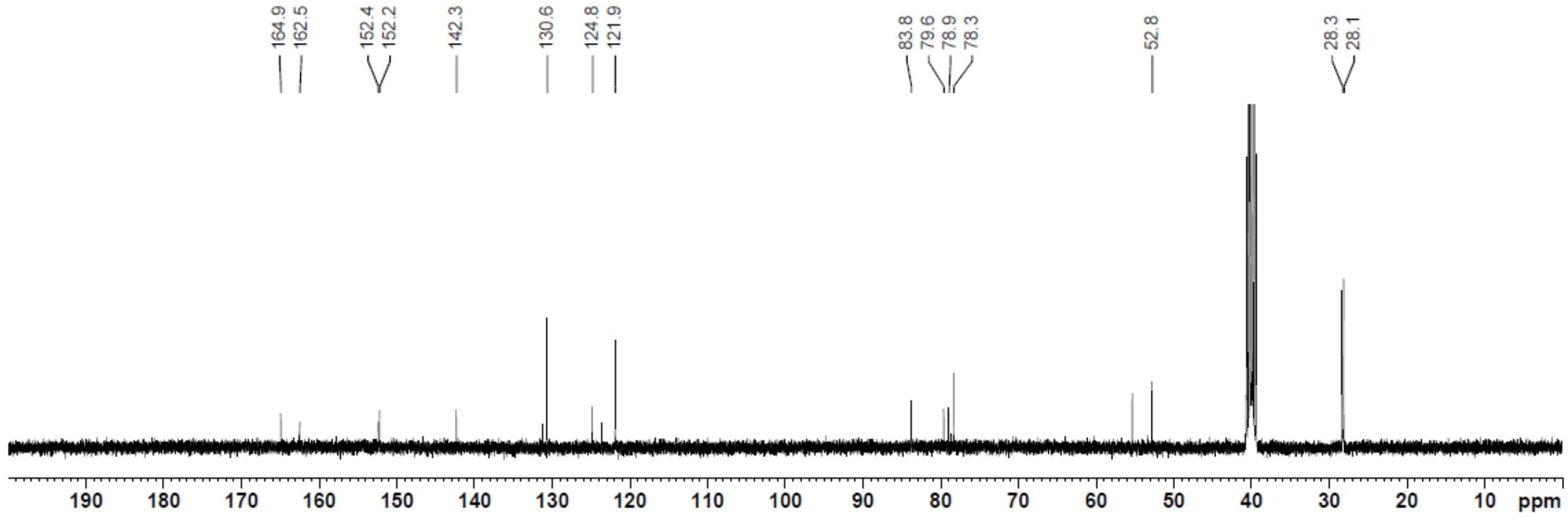

Compound **43**

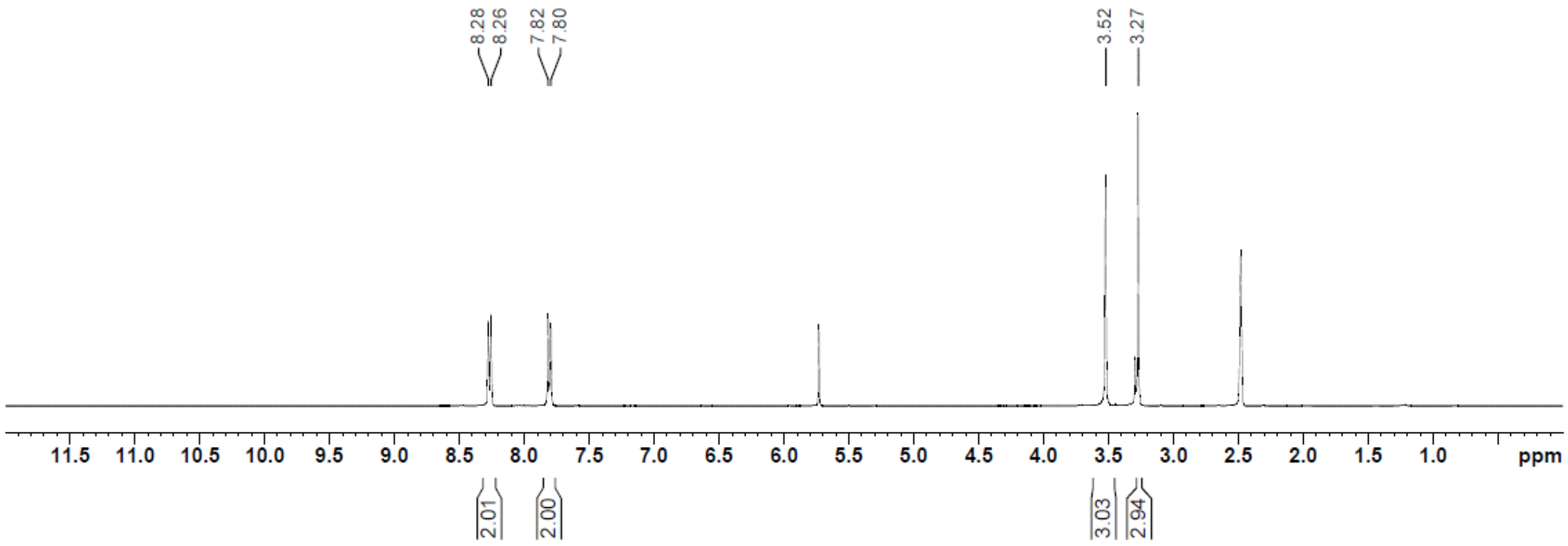
8.28
8.26
7.82
7.80
3.52
3.27
11.5
11.0
10.5
10.0
9.5
9.0
8.5
8.0
7.5
7.0
6.5
6.0
5.5
5.0
4.5
4.0
3.5
3.0
2.5
2.0
1.5
1.0
ppm
2.01
2.00
3.03
2.94

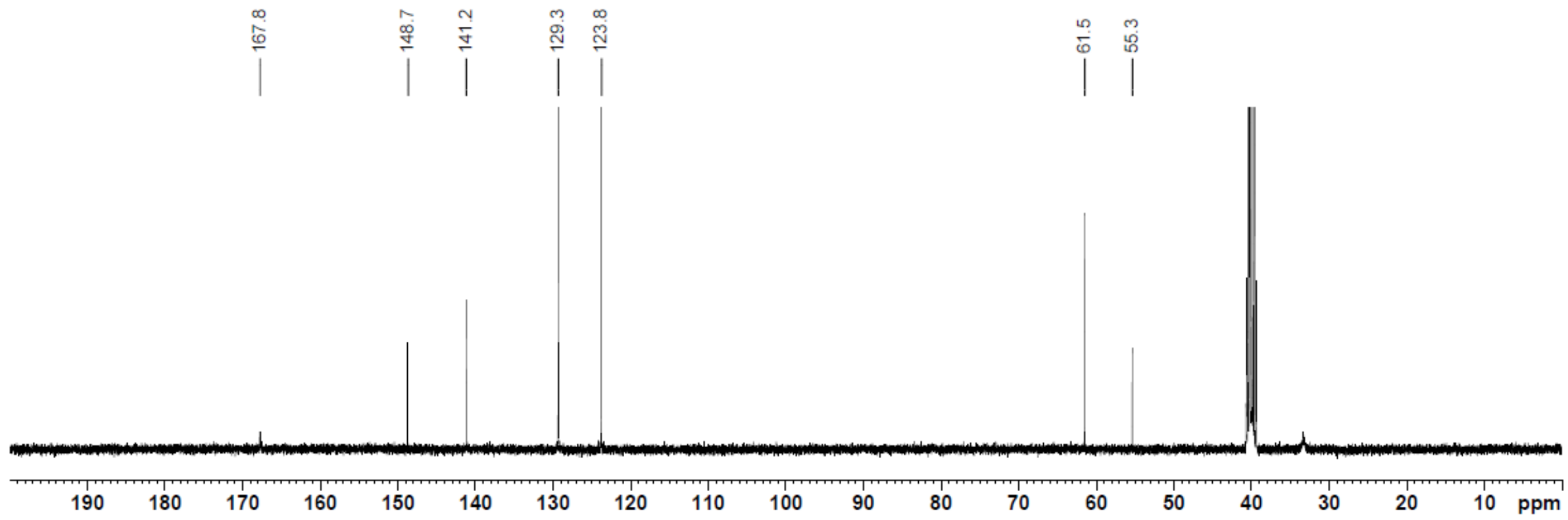
167.8
148.7
141.2
129.3
123.8
61.5
55.3
190
180
170
160
150
140
130
120
110
100
90
80
70
60
50
40
30
20
10
ppm

Compound **Boc-14**

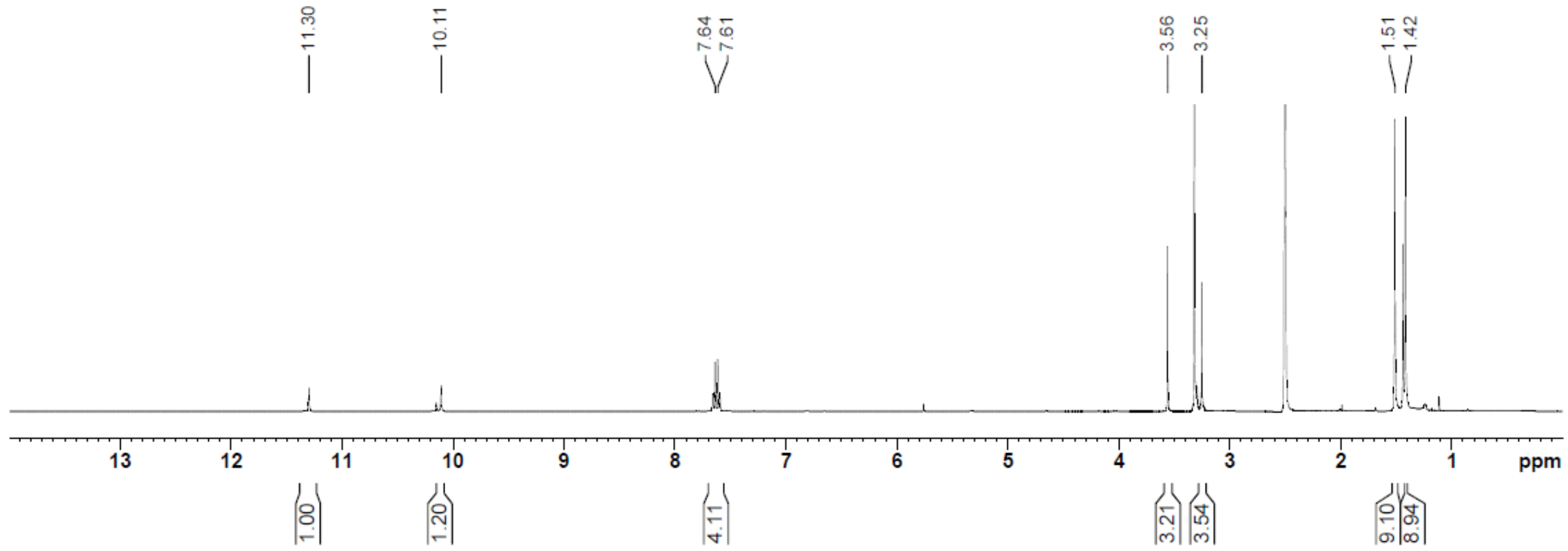

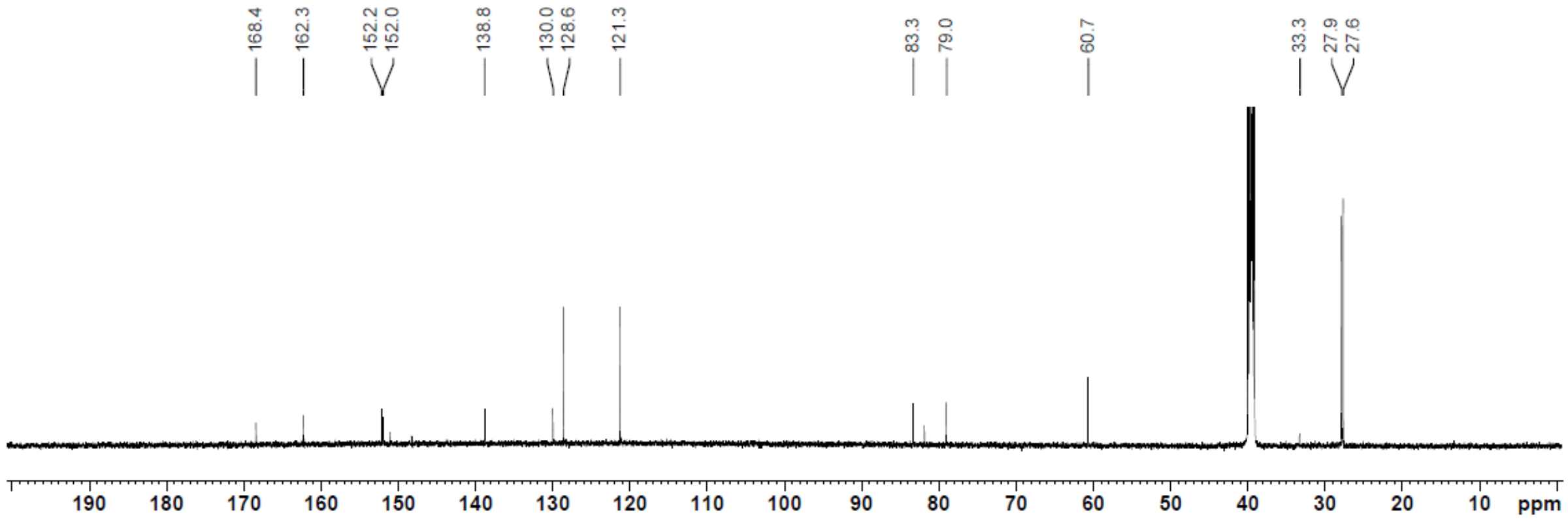

Compound **Boc-33**

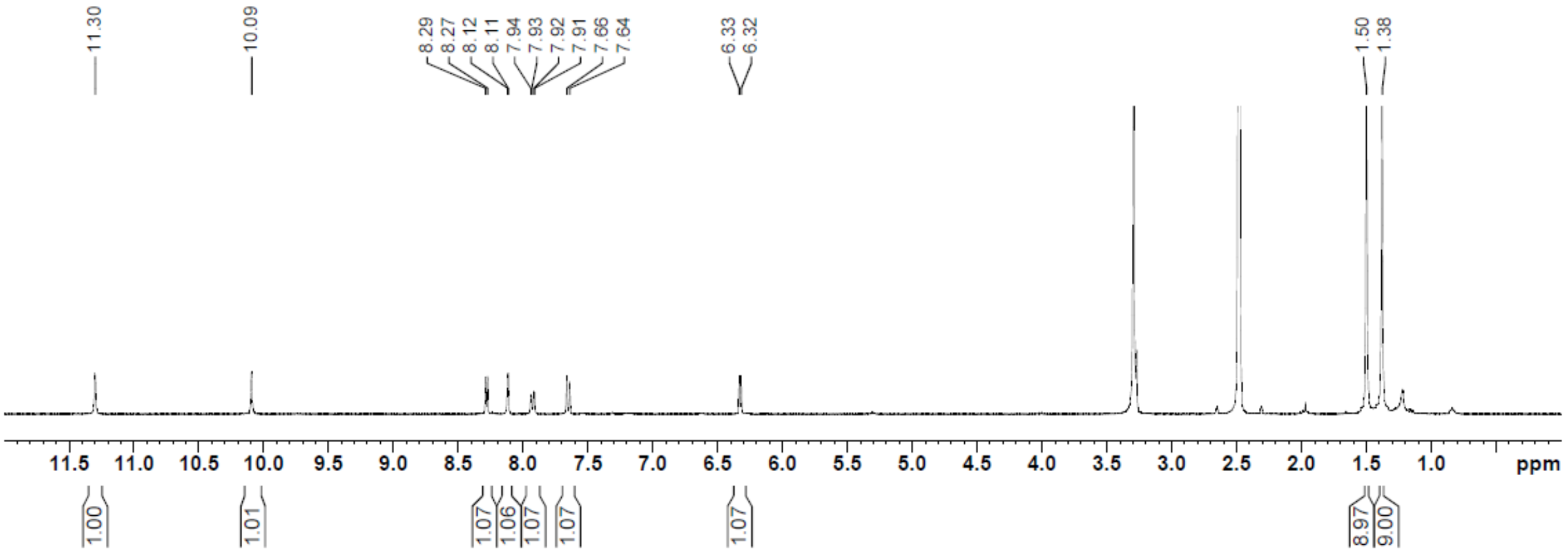

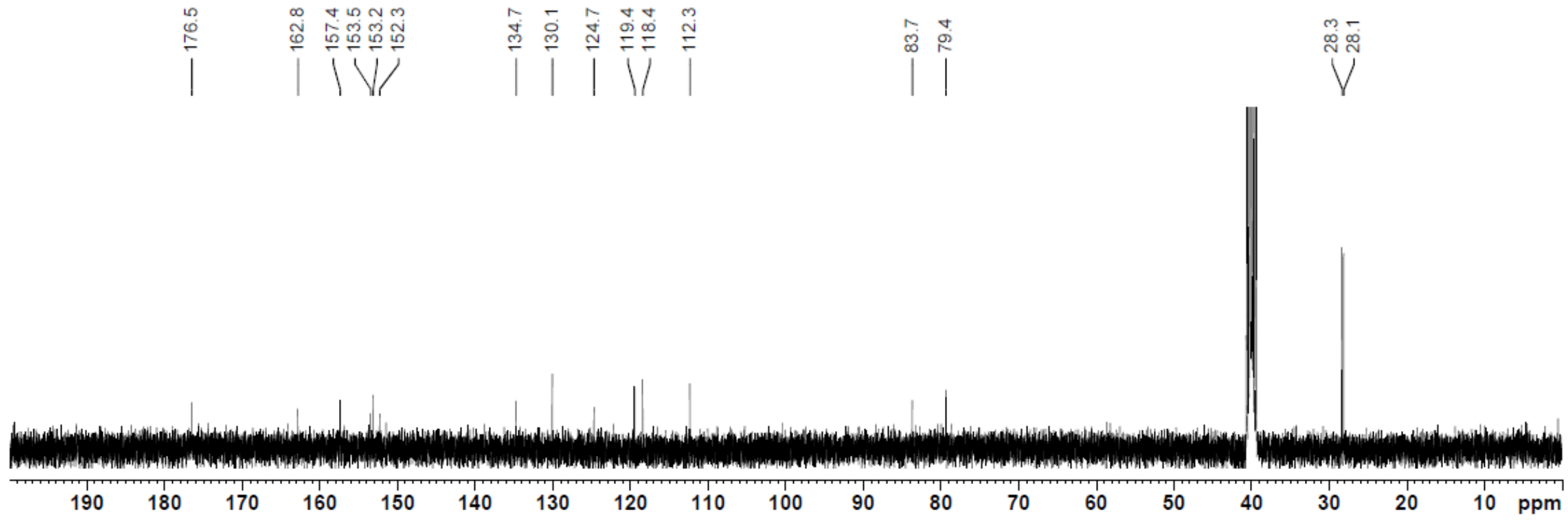

Compound **Boc-34**

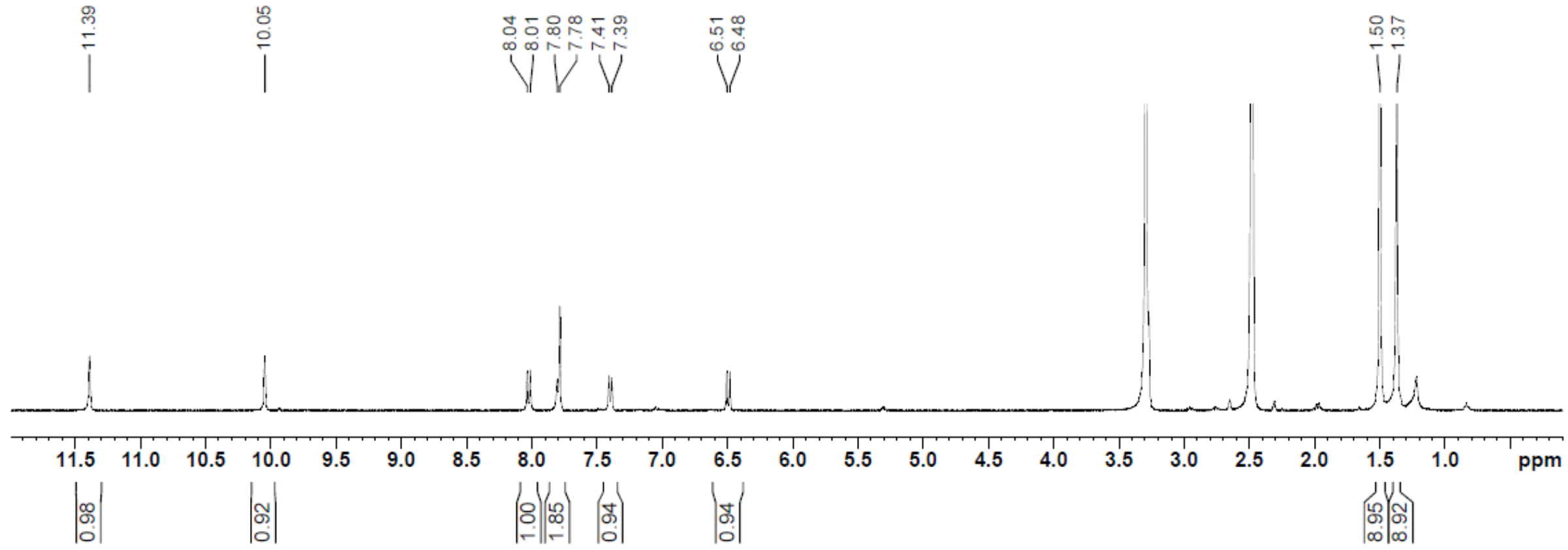

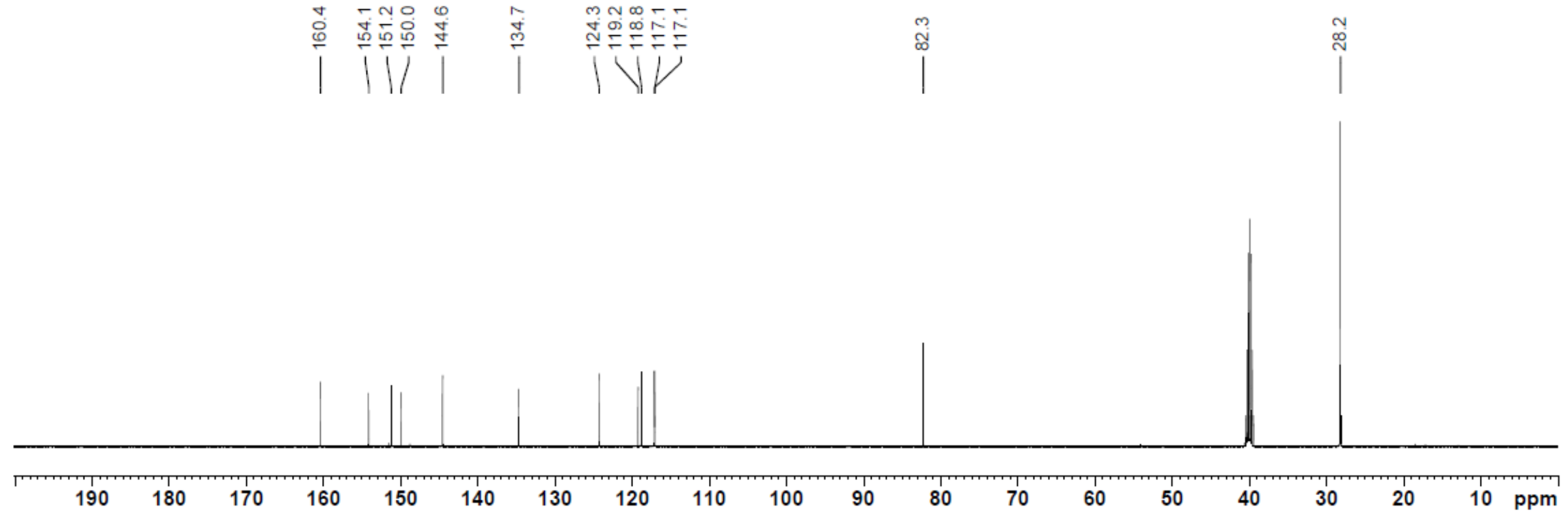

***N*-(4-Nitrophenyl)-2-benzothiazolamine**

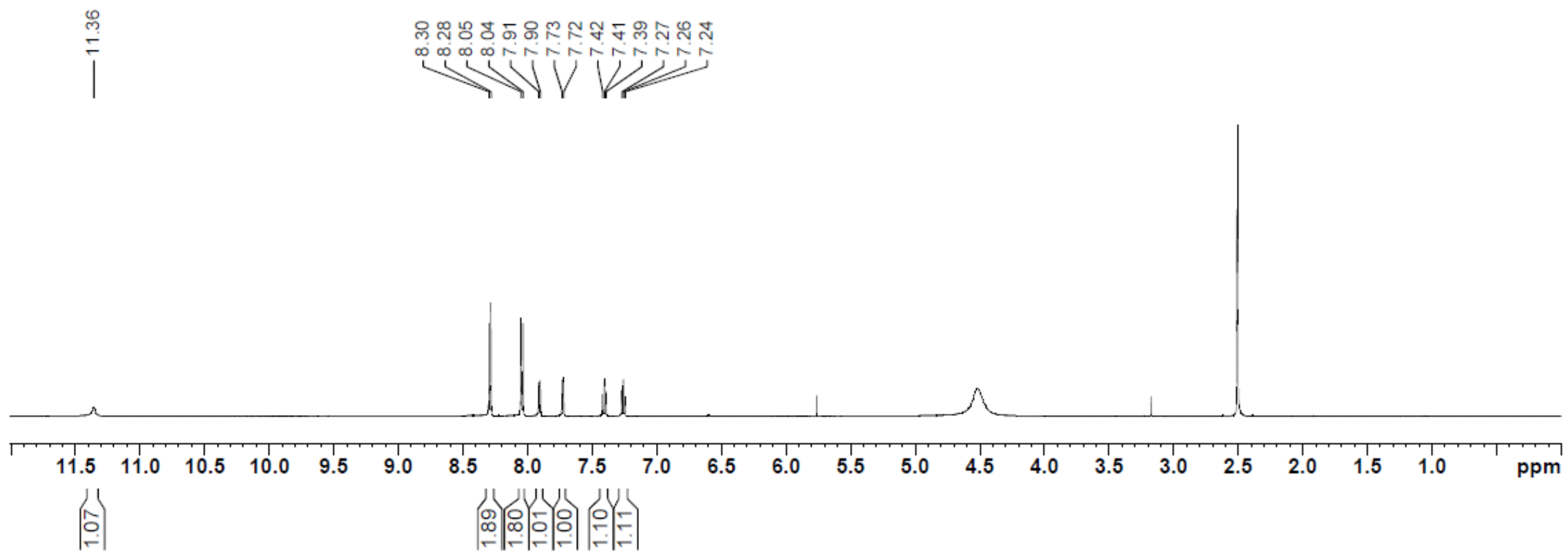

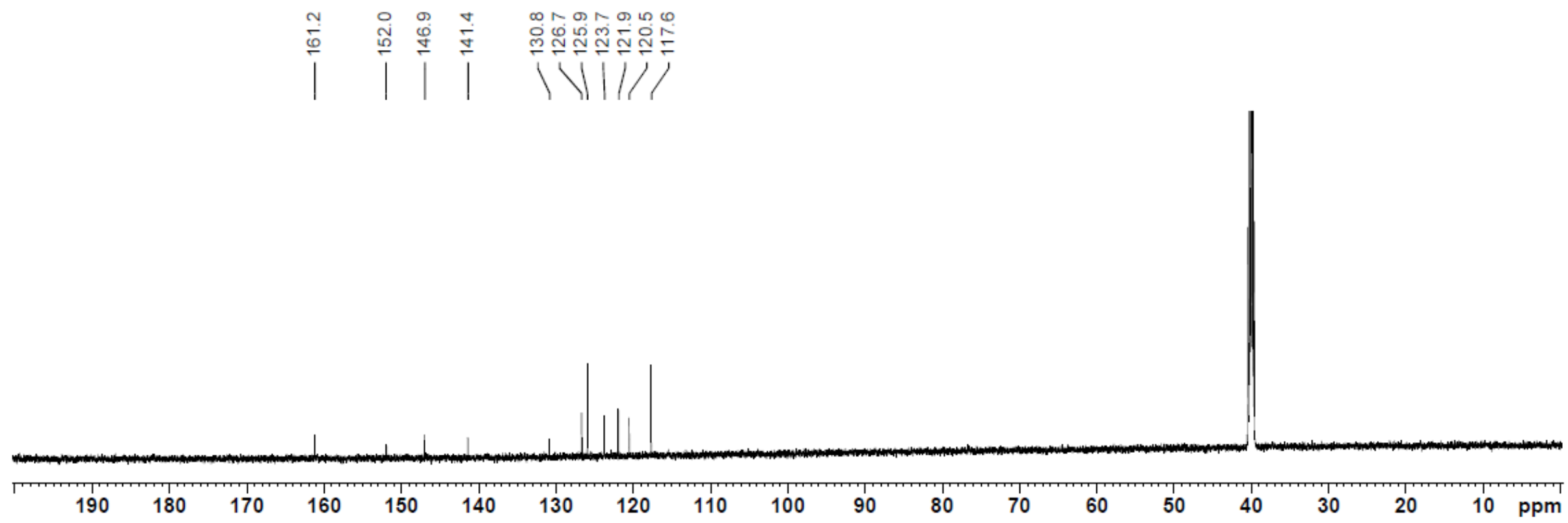

Compound **Boc-40**

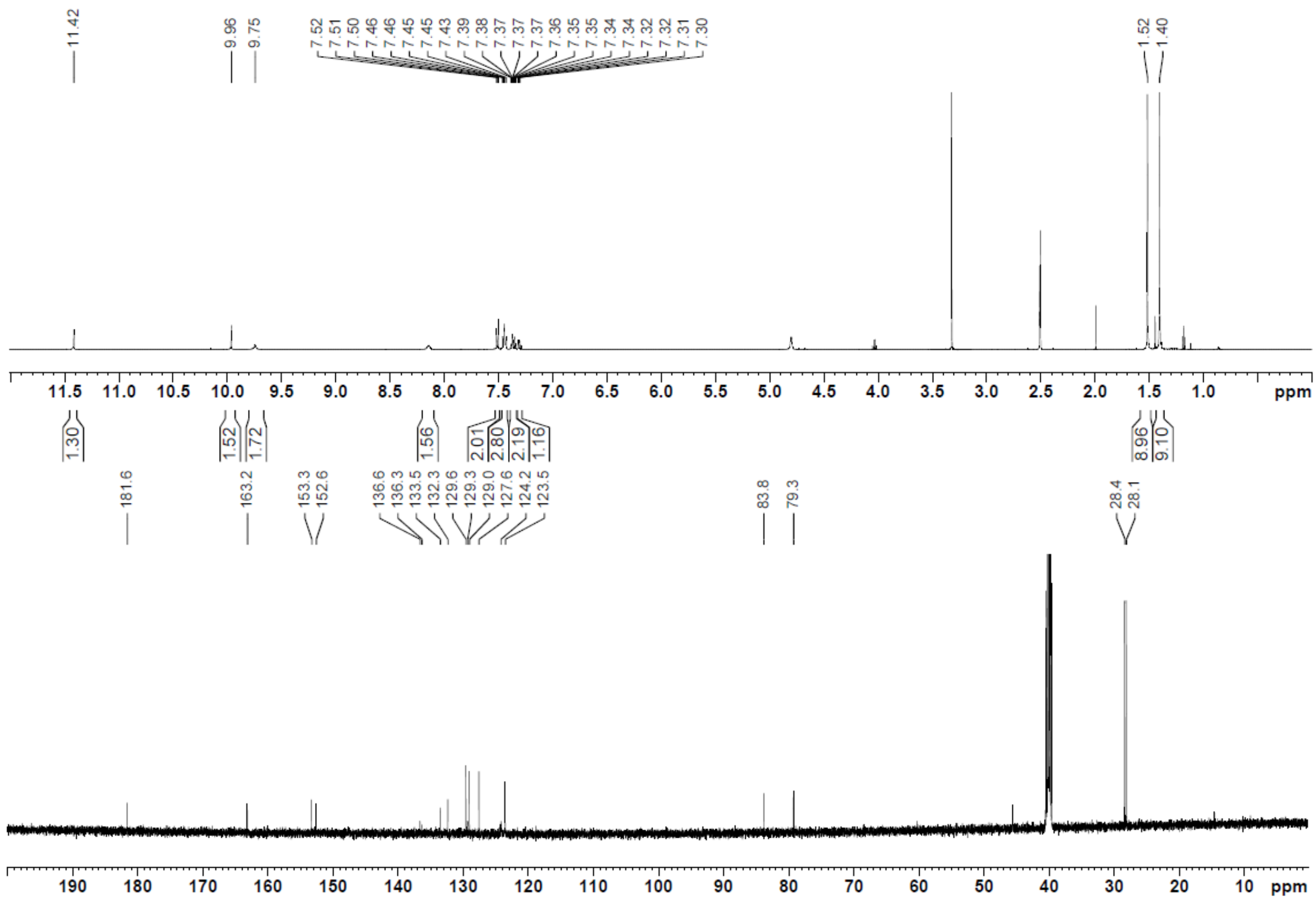

Compound **51**

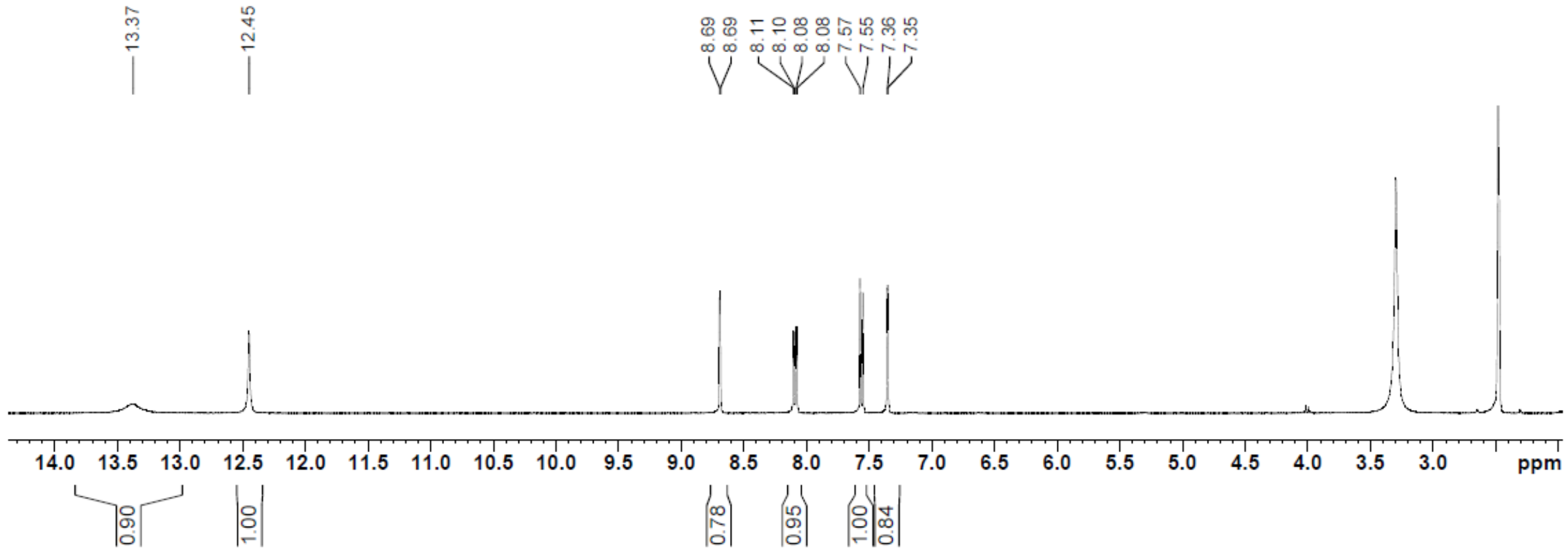

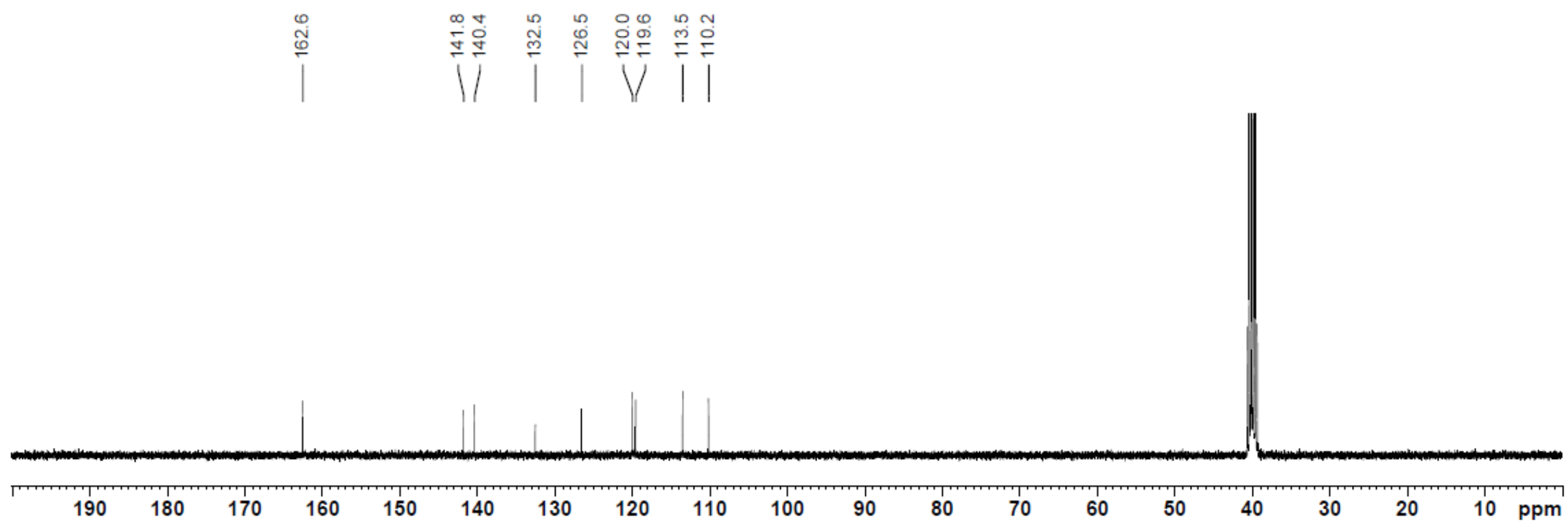

Compound **52**

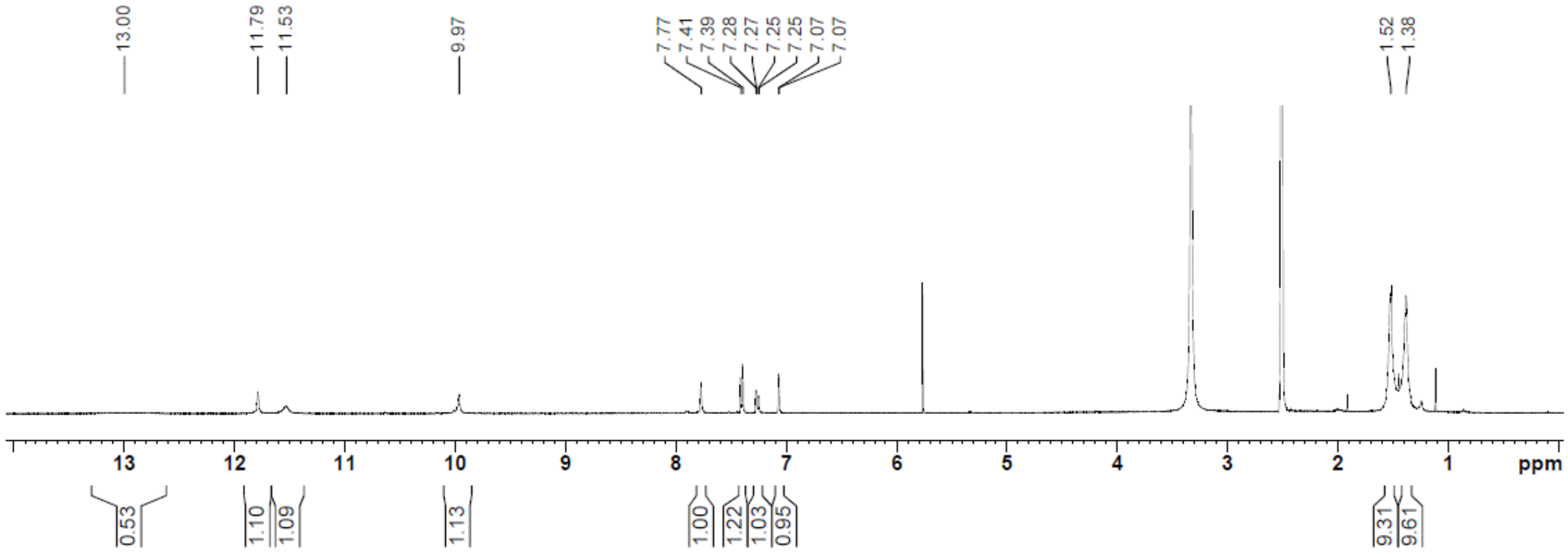

13.00
11.79
11.53
9.97
7.77
7.41
7.39
7.28
7.27
7.25
7.25
7.07
7.07
1.52
1.38
0.53
1.10
1.09
1.13
1.00
1.22
1.03
0.95
9.31
9.61
13 12 11 10 9 8 7 6 5 4 3 2 1 ppm

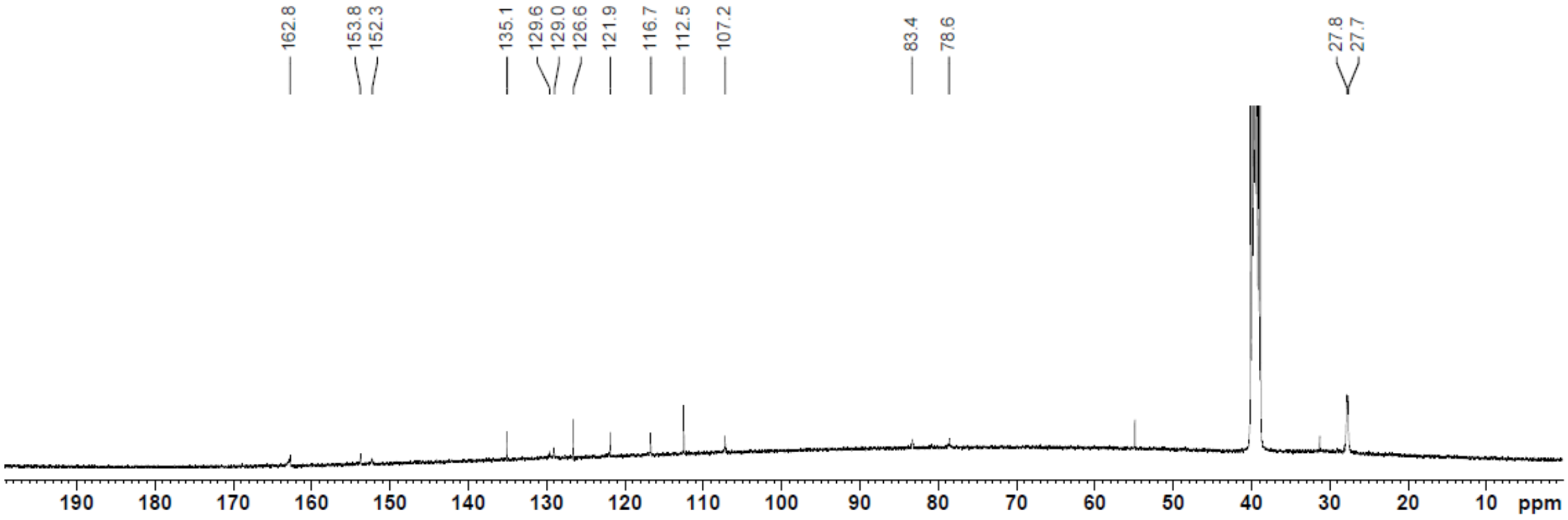

162.8
153.8
152.3
135.1
129.6
129.0
126.6
121.9
116.7
112.5
107.2
83.4
78.6
27.8
27.7
190 180 170 160 150 140 130 120 110 100 90 80 70 60 50 40 30 20 10 ppm

Ethyl 5-amino-1*H*-indole-2-carboxylate

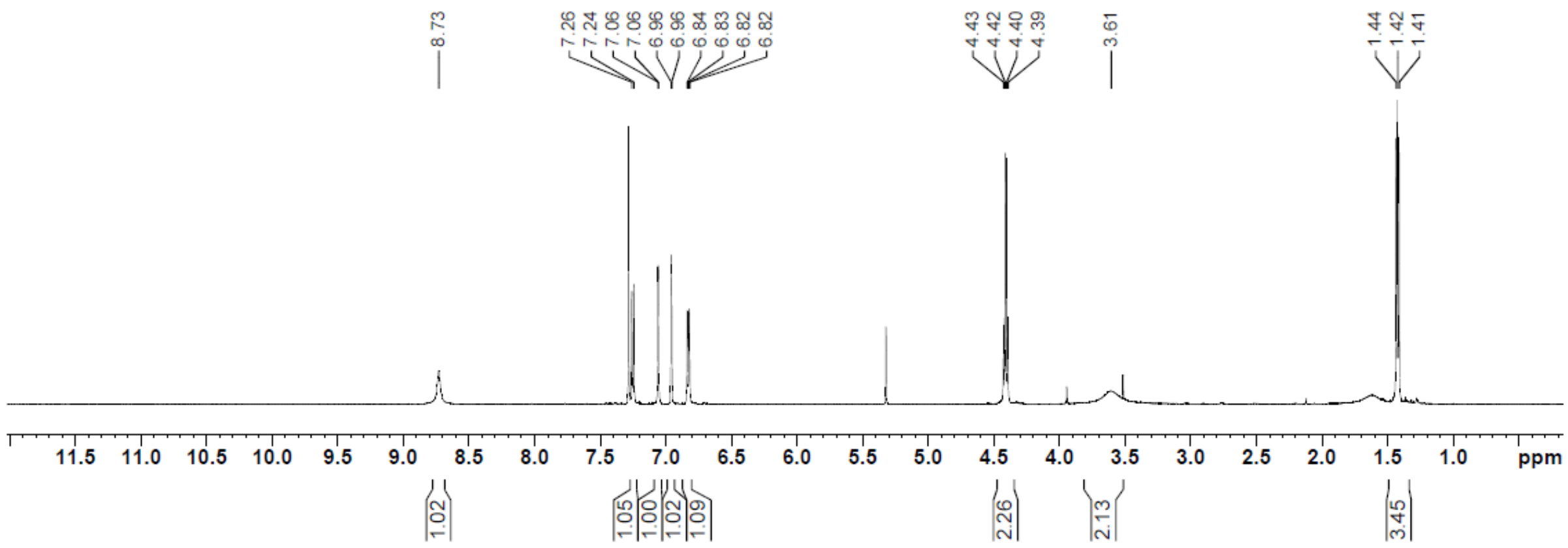

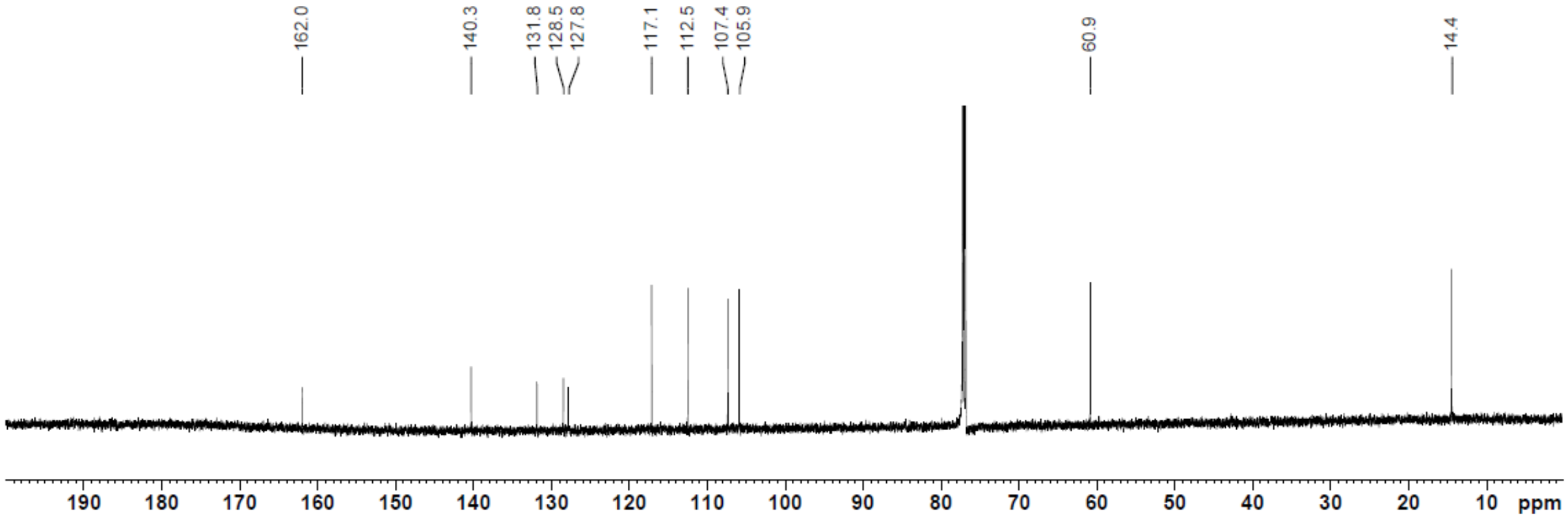

***N*[1]-methylbenzene-1,4-diamine**

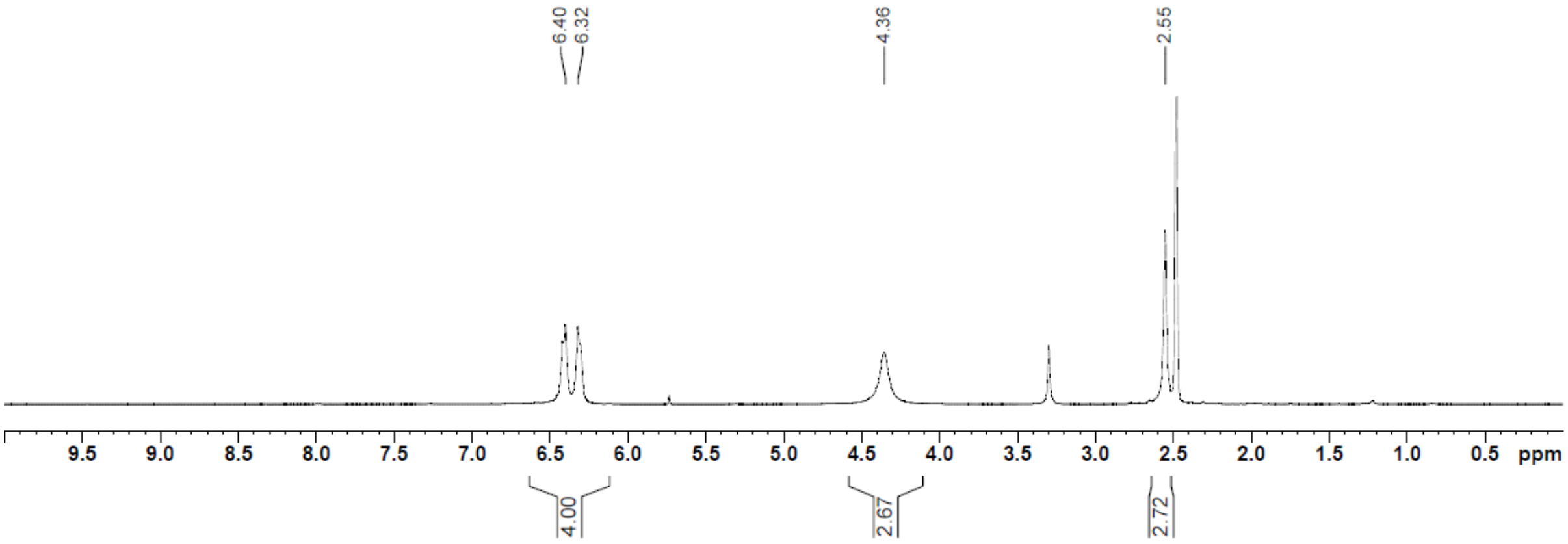

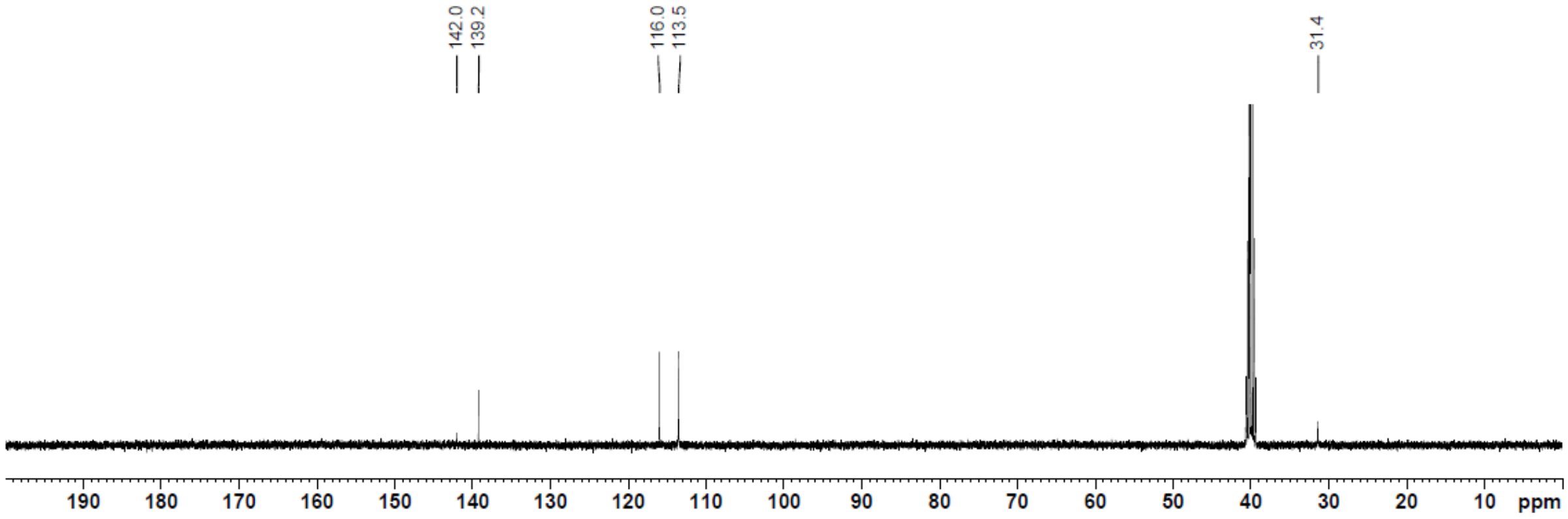

Compound **55**

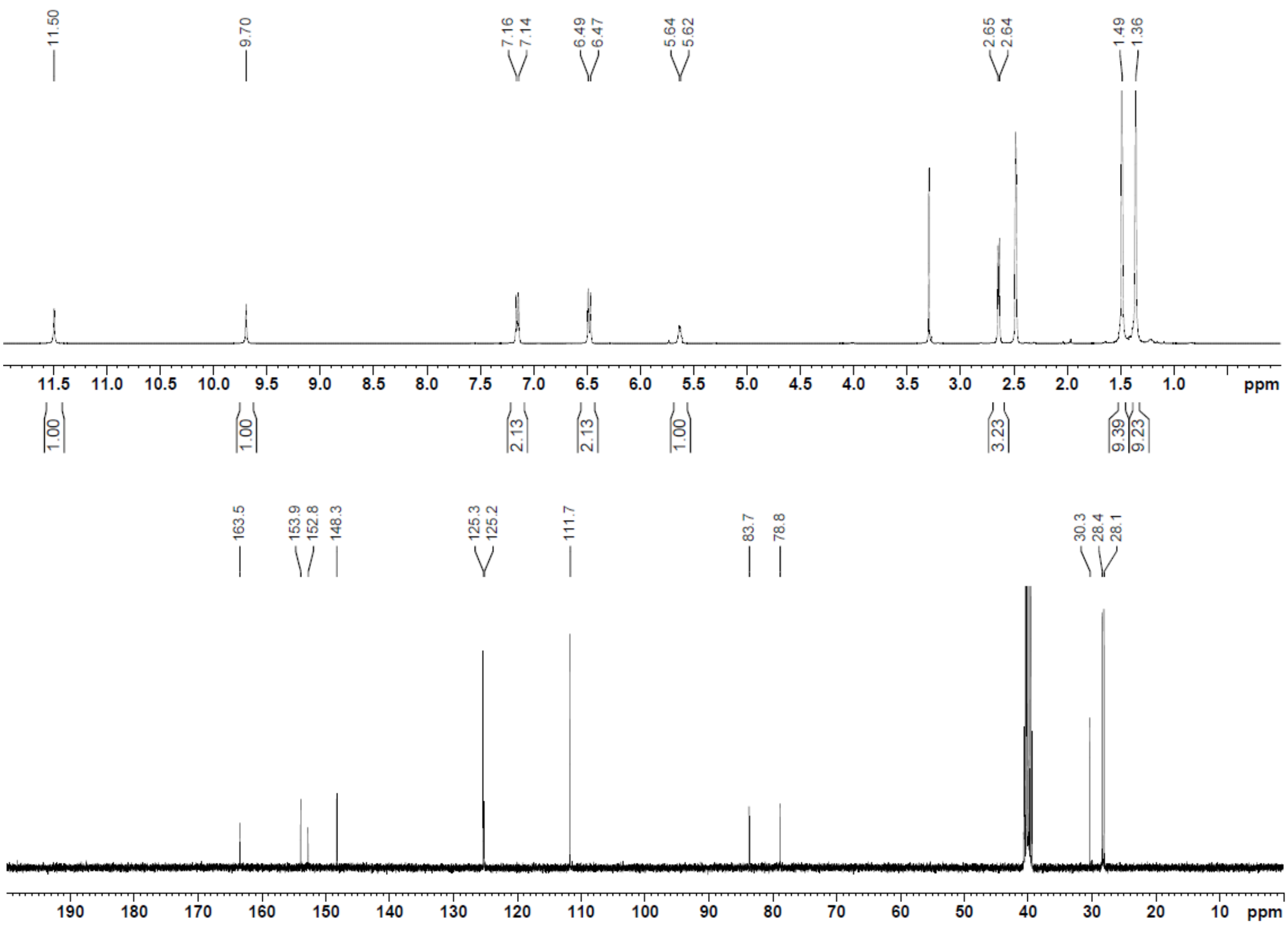

11.50
9.70
7.16
7.14
6.49
6.47
5.64
5.62
2.65
2.64
1.49
1.36
ppm
1.00
1.00
2.13
2.13
1.00
3.23
9.39
9.23
163.5
153.9
152.8
148.3
125.3
125.2
111.7
83.7
78.8
30.3
28.4
28.1
ppm

Compound **60**

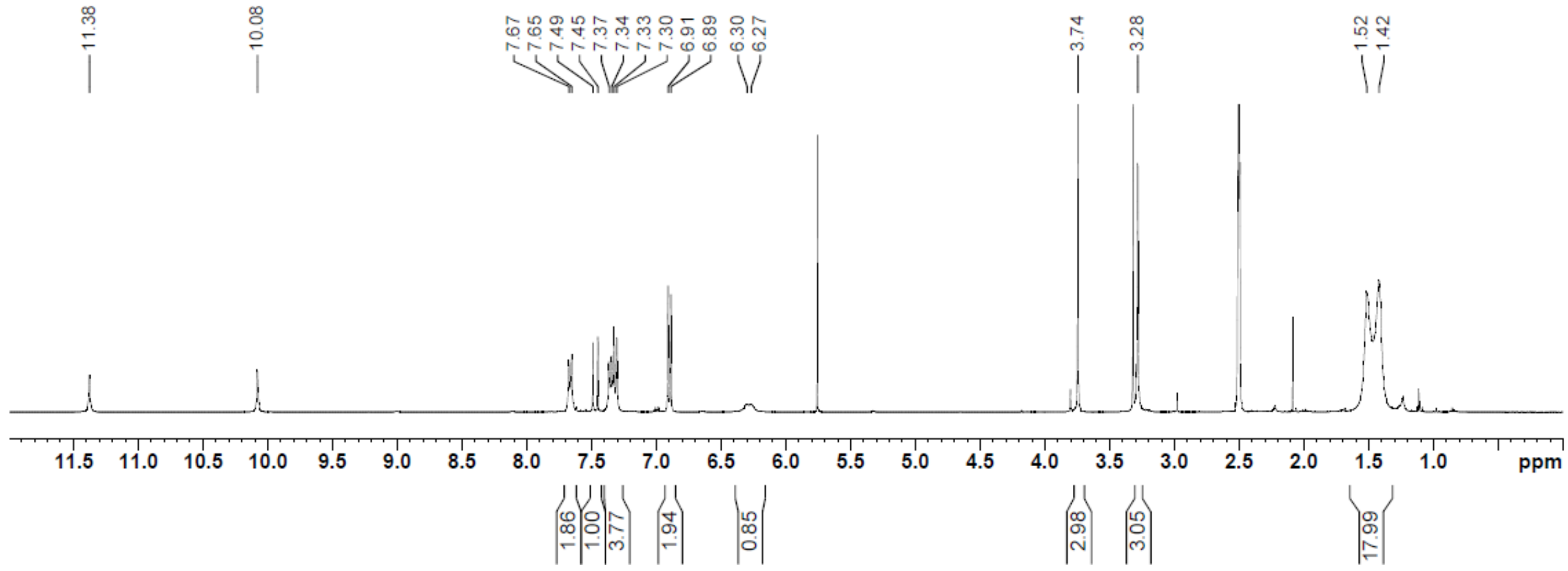

11.38
10.08
7.67
7.65
7.49
7.45
7.37
7.34
7.33
7.30
6.91
6.89
6.30
6.27
3.74
3.28
1.52
1.42
11.5
11.0
10.5
10.0
9.5
9.0
8.5
8.0
7.5
7.0
6.5
6.0
5.5
5.0
4.5
4.0
3.5
3.0
2.5
2.0
1.5
1.0
ppm
1.86
1.00
3.77
1.94
0.85
2.98
3.05
17.99

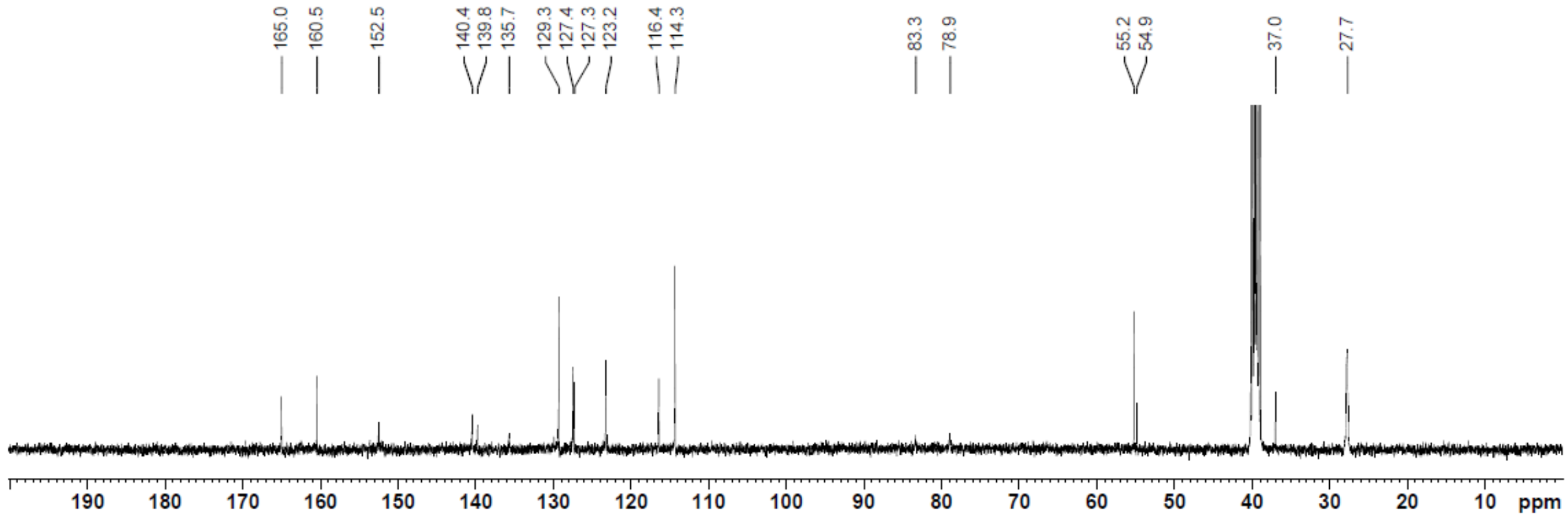

165.0
160.5
152.5
140.4
139.8
135.7
129.3
127.4
127.3
123.2
116.4
114.3
83.3
78.9
55.2
54.9
37.0
27.7
190
180
170
160
150
140
130
120
110
100
90
80
70
60
50
40
30
20
10
ppm

Compound **59**

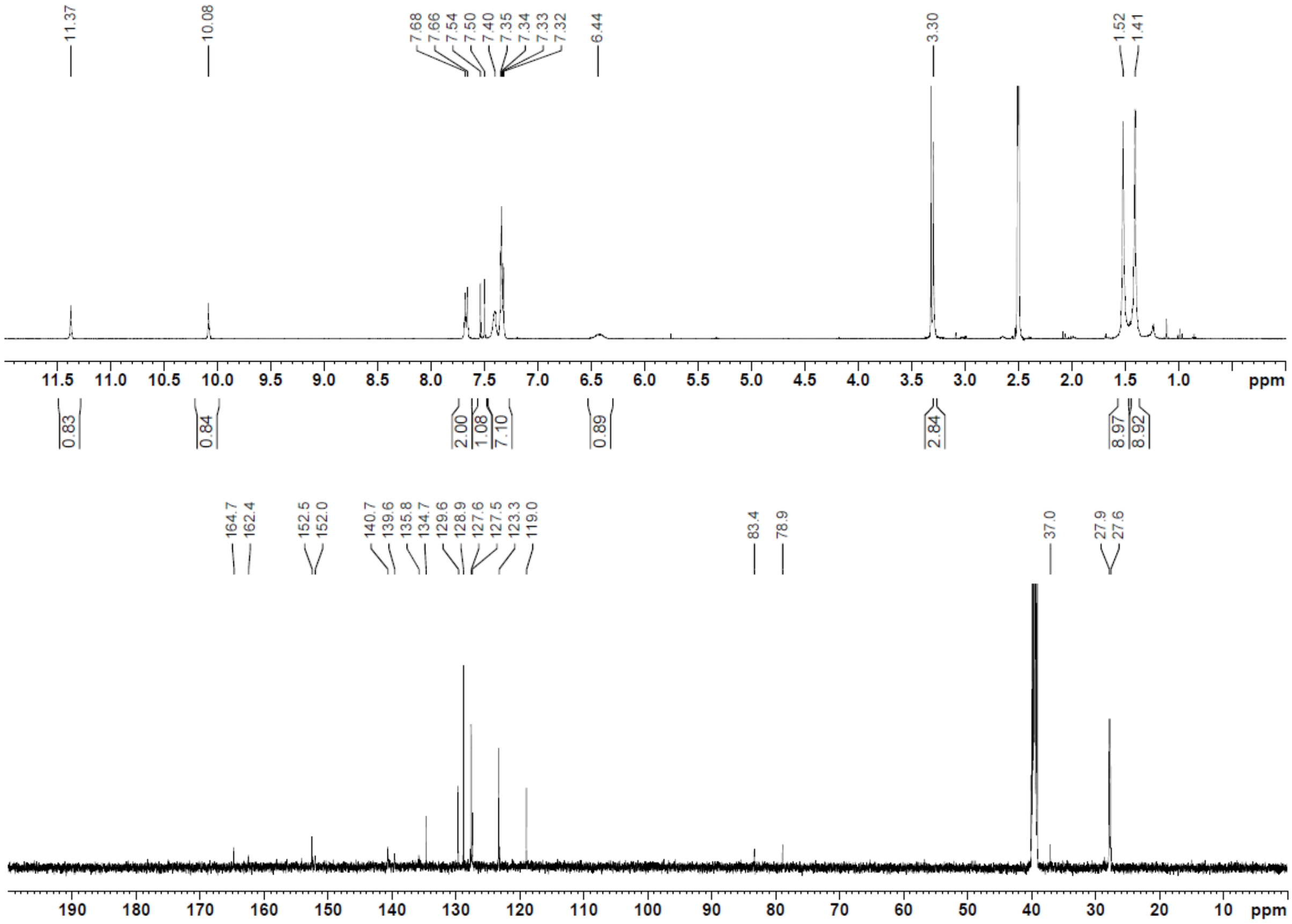

11.37
10.08
7.68
7.66
7.54
7.50
7.40
7.35
7.34
7.33
7.32
6.44
3.30
1.52
1.41
11.5 11.0 10.5 10.0 9.5 9.0 8.5 8.0 7.5 7.0 6.5 6.0 5.5 5.0 4.5 4.0 3.5 3.0 2.5 2.0 1.5 1.0 ppm
0.83
0.84
2.00
1.08
7.10
0.89
2.84
8.97
8.92
164.7
162.4
152.5
152.0
140.7
139.6
135.8
134.7
129.6
128.9
127.6
127.5
123.3
119.0
83.4
78.9
37.0
27.9
27.6
190 180 170 160 150 140 130 120 110 100 90 80 70 60 50 40 30 20 10 ppm

Compound **58**

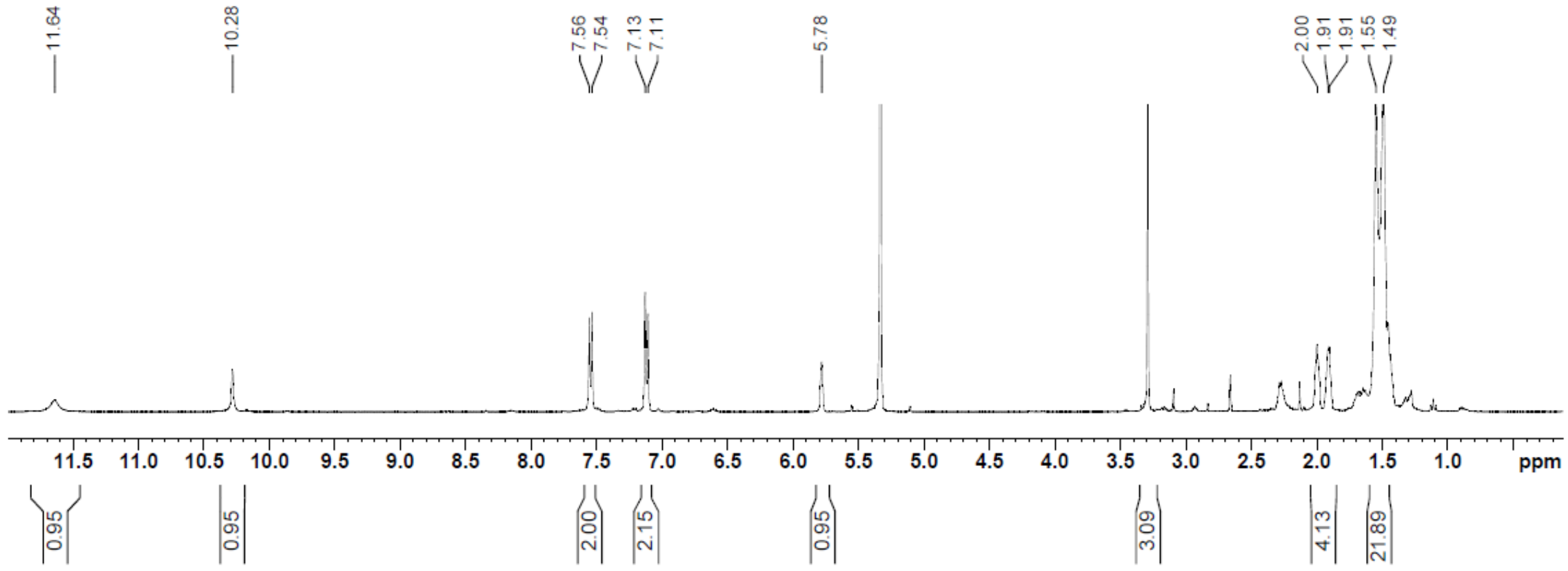

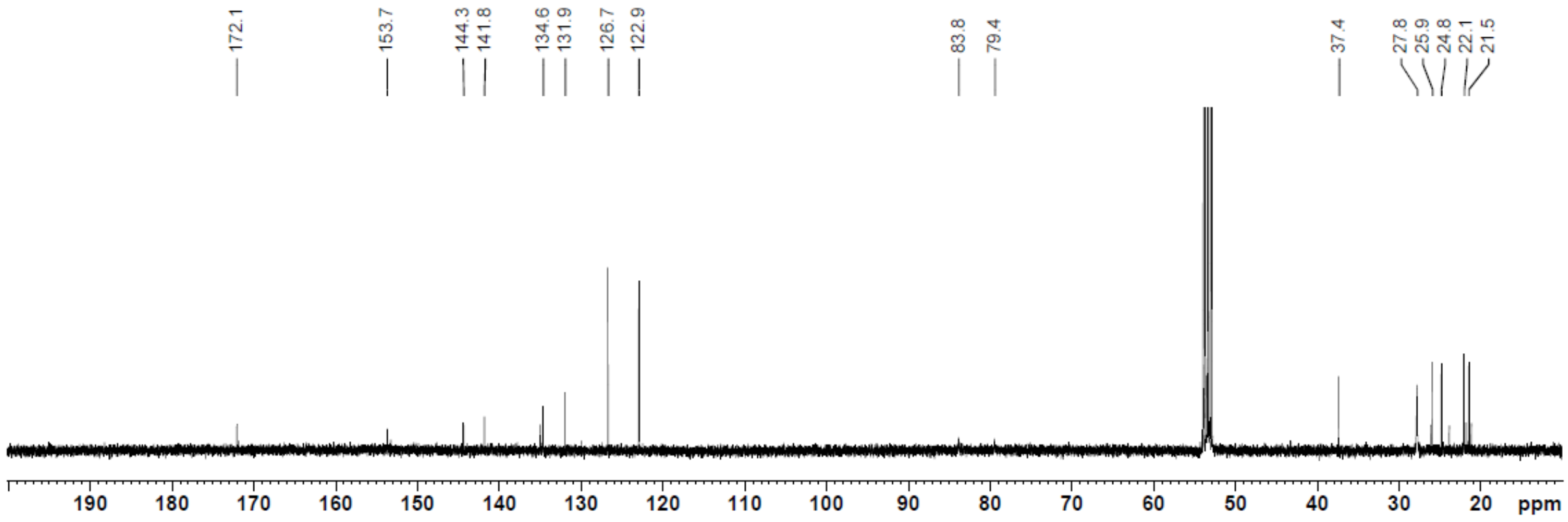

Compound **57**

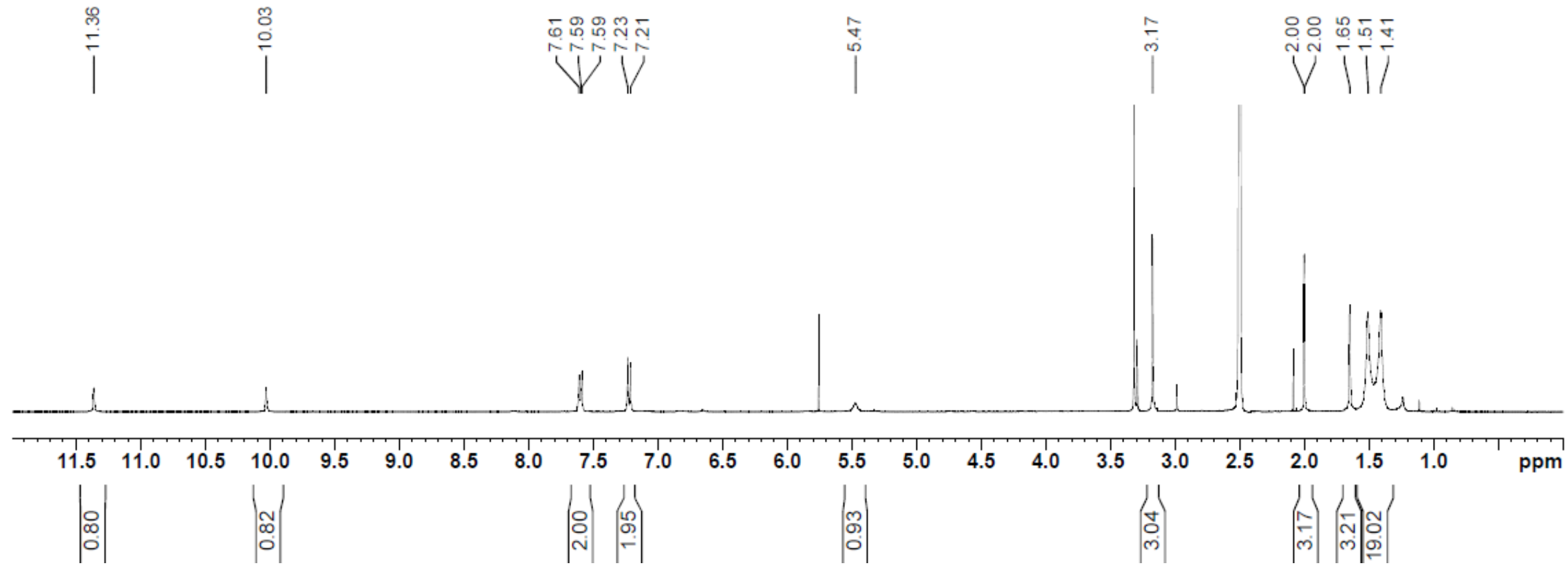
11.36
10.03
7.61
7.59
7.59
7.23
7.21
5.47
3.17
2.00
2.00
1.65
1.51
1.41
0.80
0.82
2.00
1.95
0.93
3.04
3.17
3.21
19.02
ppm

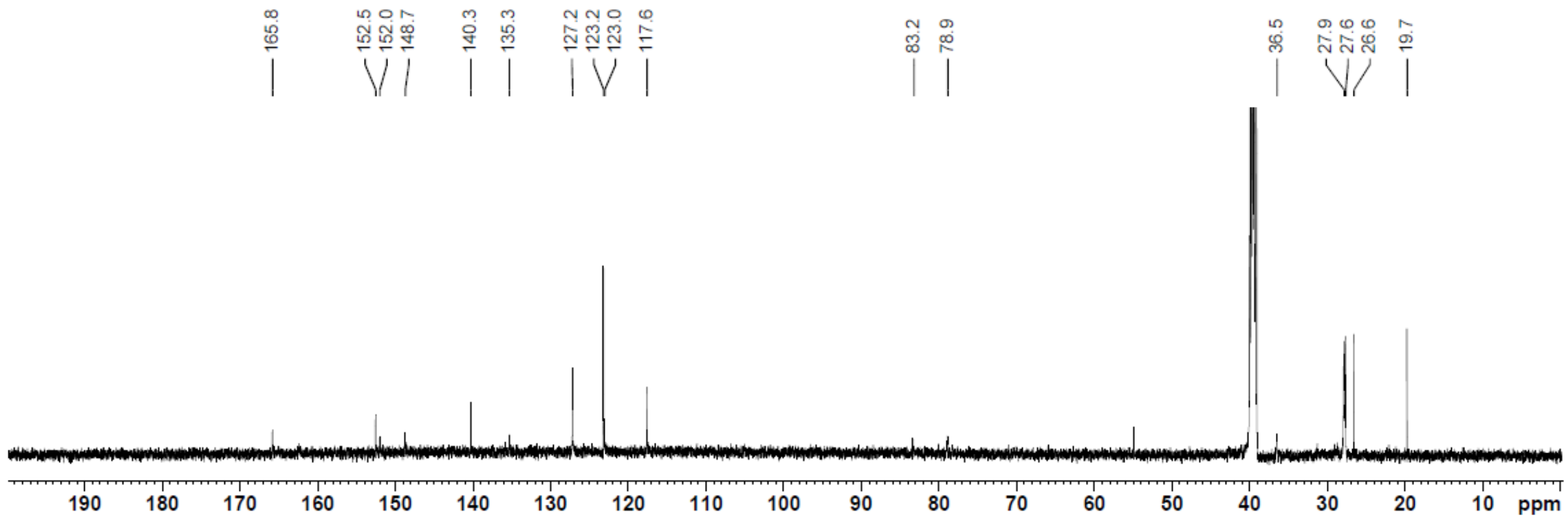
165.8
152.5
152.0
148.7
140.3
135.3
127.2
123.2
123.0
117.6
83.2
78.9
36.5
27.9
27.6
26.6
19.7
ppm

Compound **56**

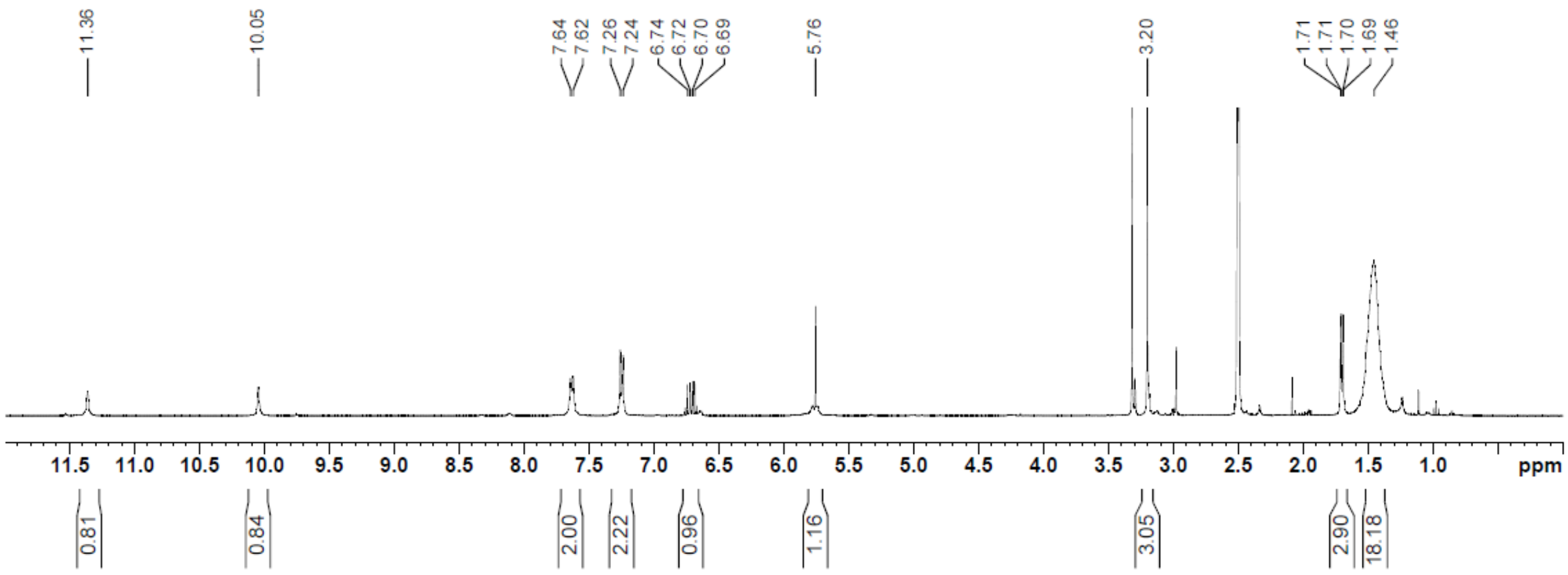
11.36
10.05
7.64
7.62
7.26
7.24
6.74
6.72
6.70
6.69
5.76
3.20
1.71
1.71
1.70
1.69
1.46
11.5 11.0 10.5 10.0 9.5 9.0 8.5 8.0 7.5 7.0 6.5 6.0 5.5 5.0 4.5 4.0 3.5 3.0 2.5 2.0 1.5 1.0 ppm
0.81
0.84
2.00
2.22
0.96
1.16
3.05
2.90
18.18

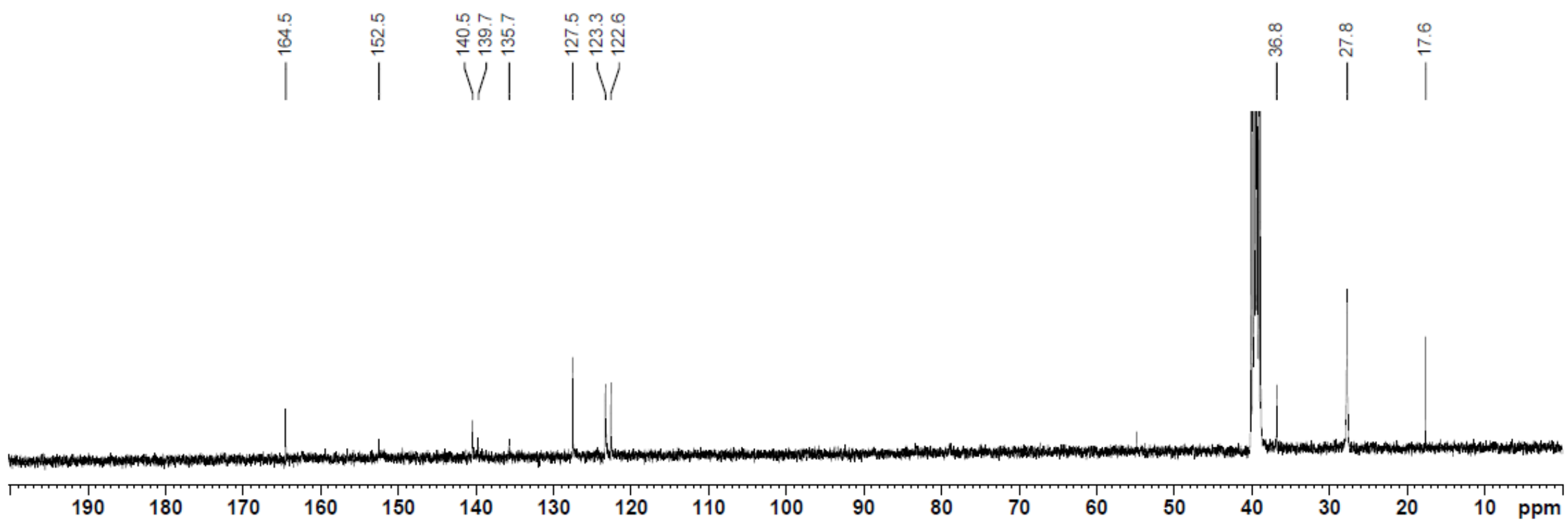
164.5
152.5
140.5
139.7
135.7
127.5
123.3
122.6
36.8
27.8
17.6
190 180 170 160 150 140 130 120 110 100 90 80 70 60 50 40 30 20 10 ppm

***tert*-butyl (*E*)-(4-(3-(4-methoxyphenyl)acrylamido)phenyl)carbamate**

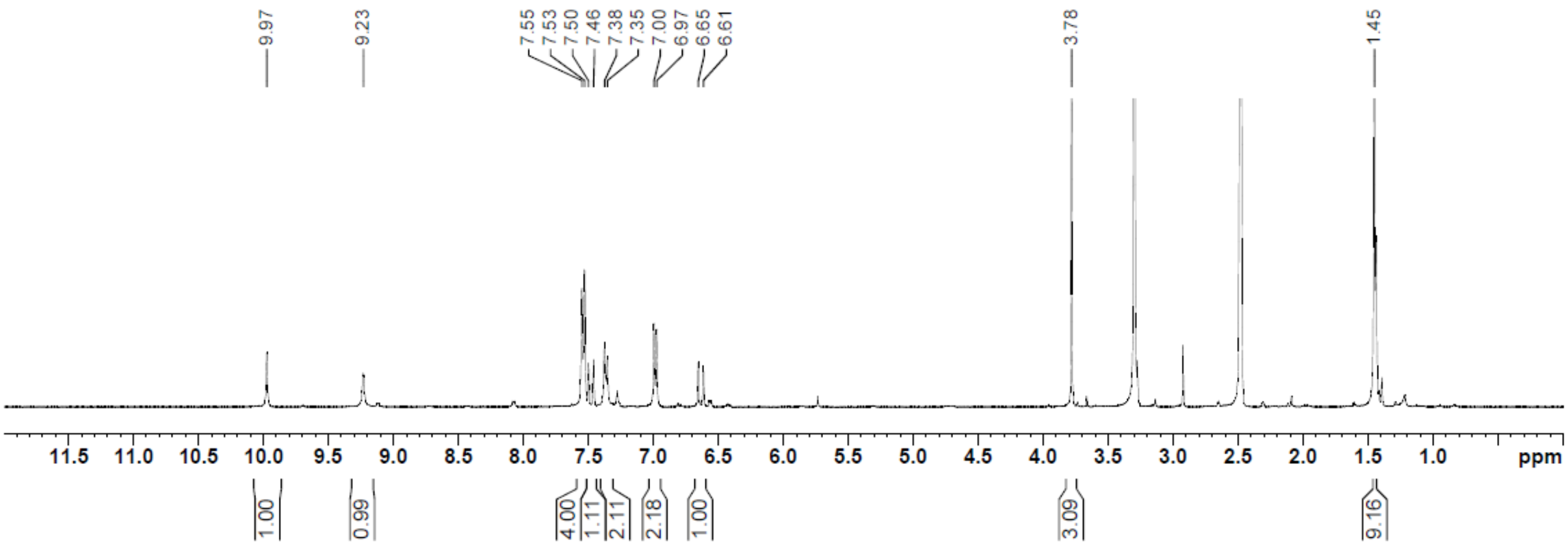

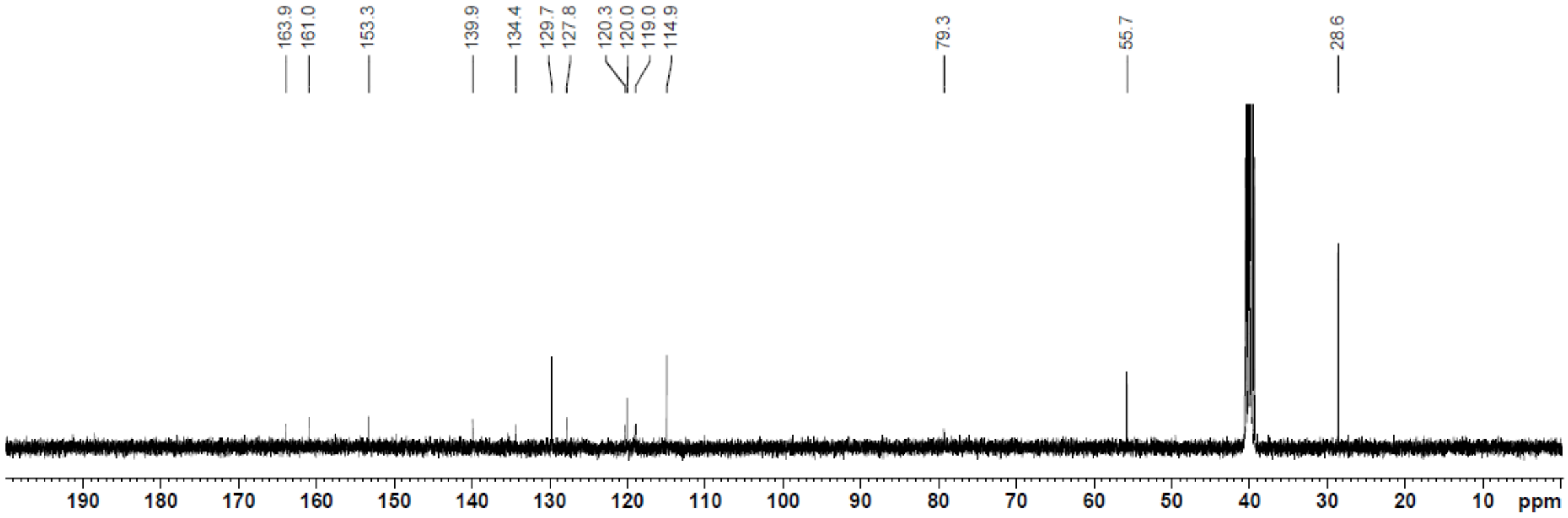

Compound **Boc-30**

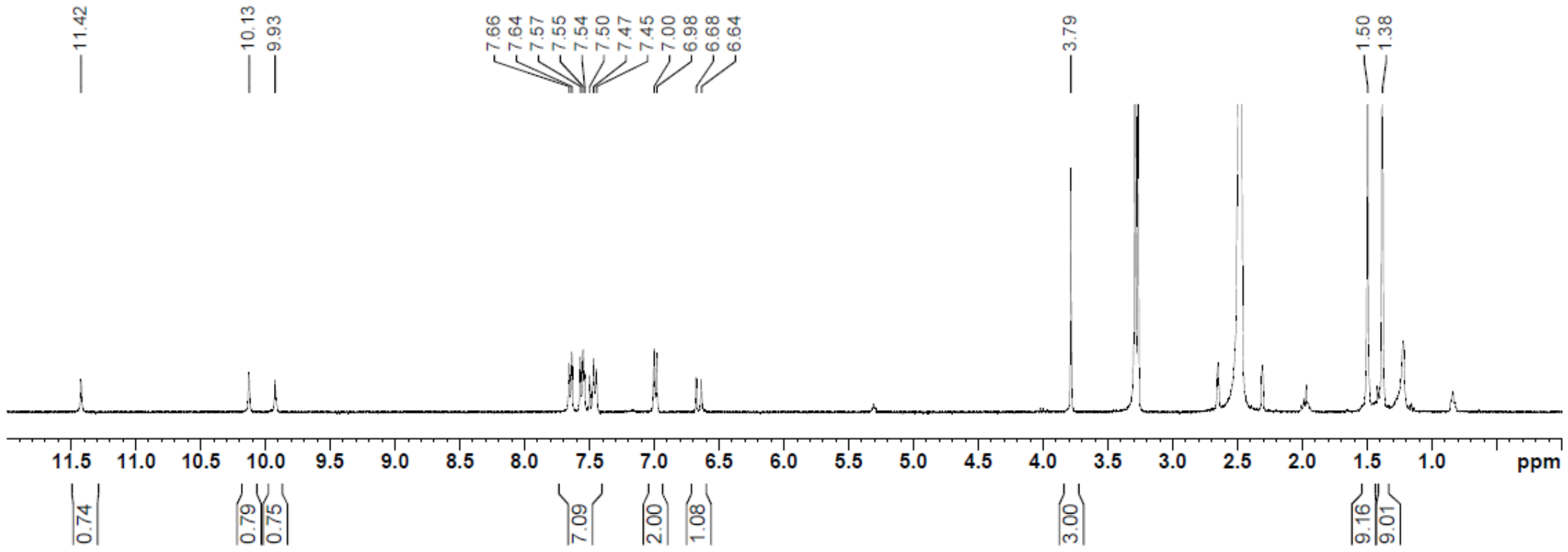

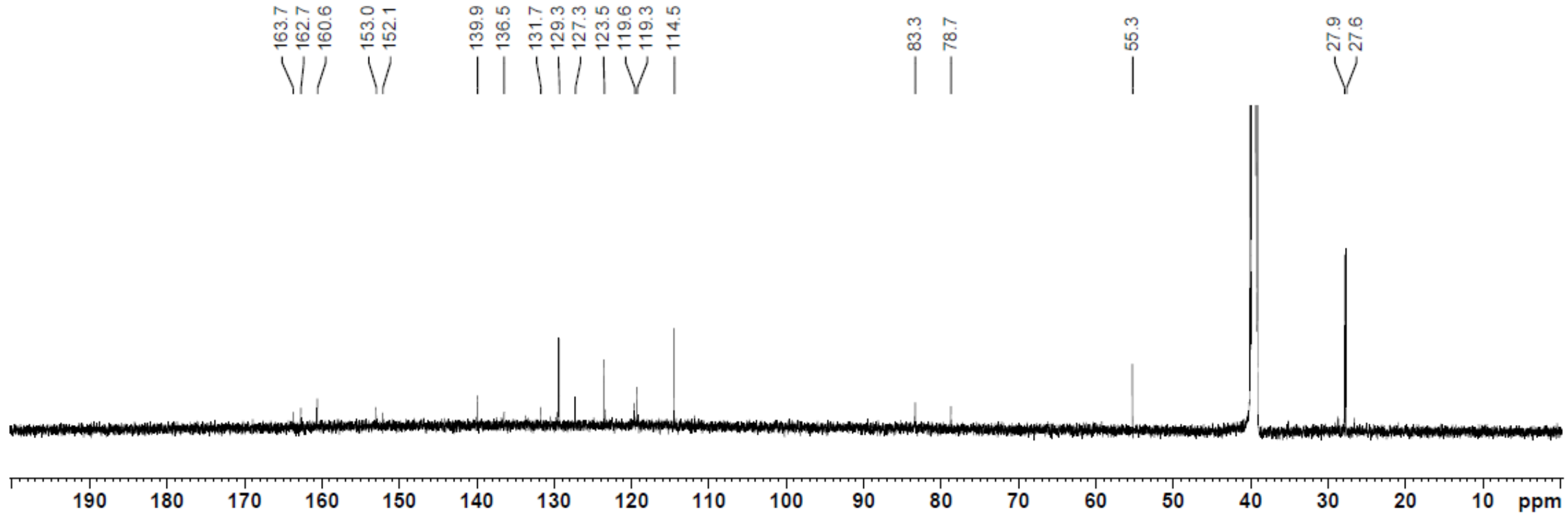

***tert*-butyl (4-cinnamamidophenyl)carbamate**

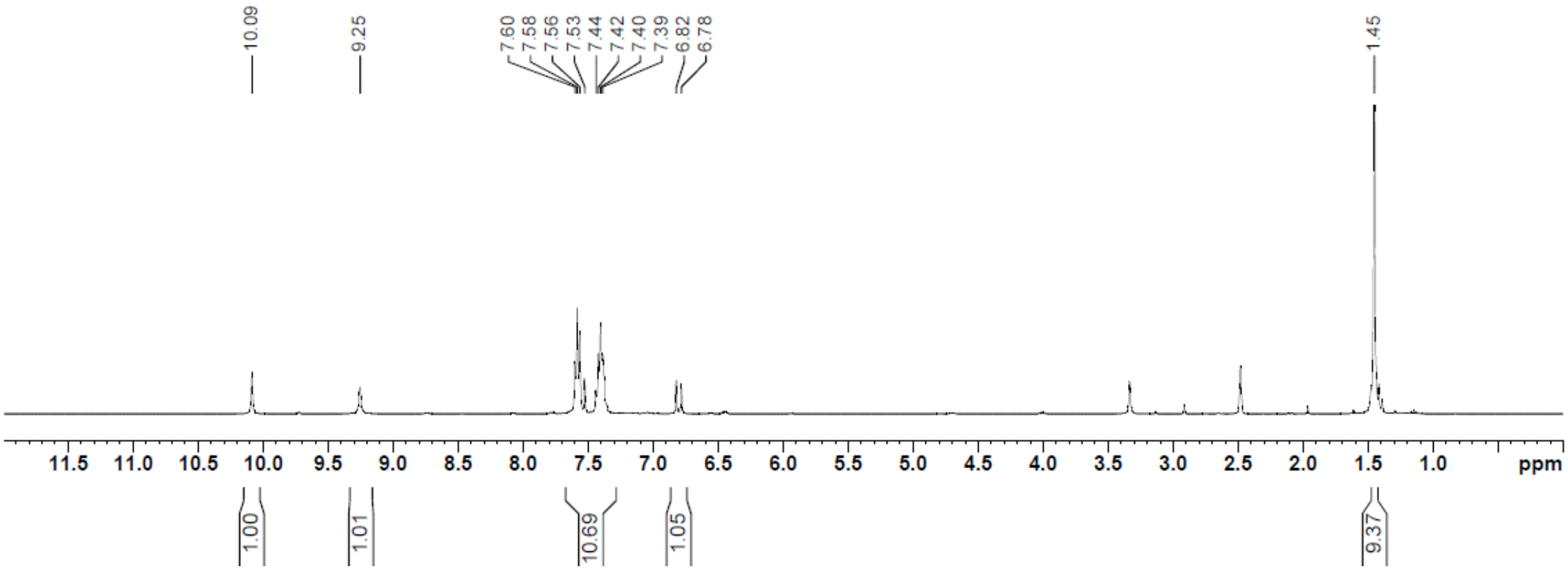

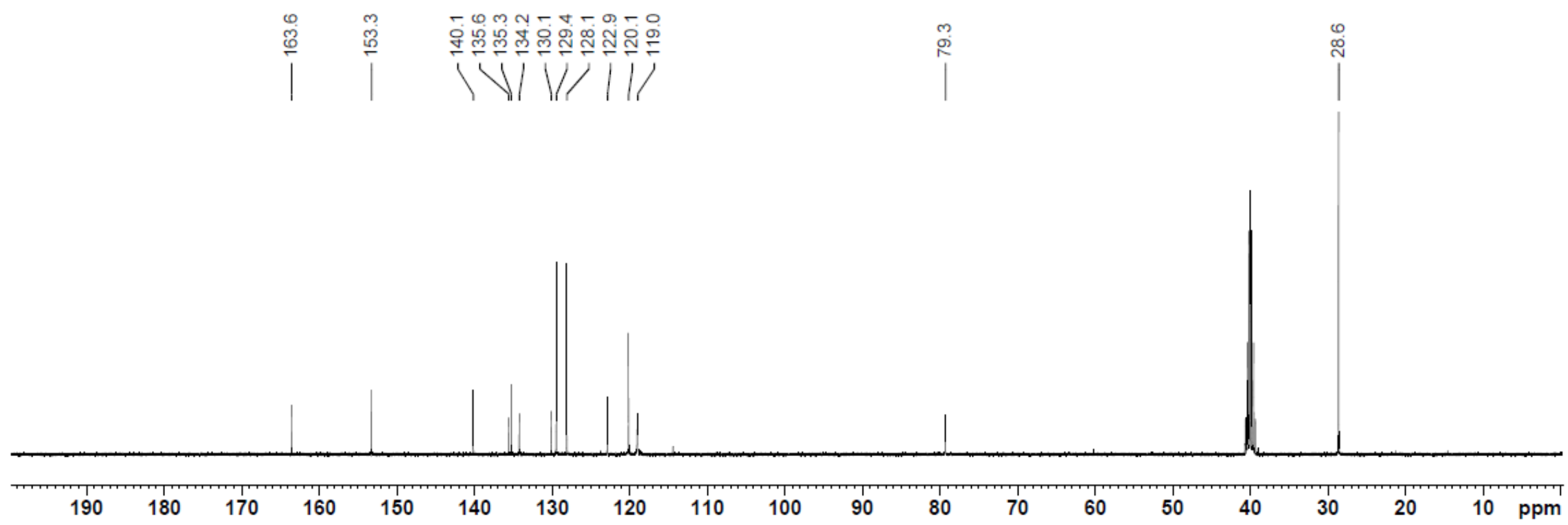

Compound **Boc-28**

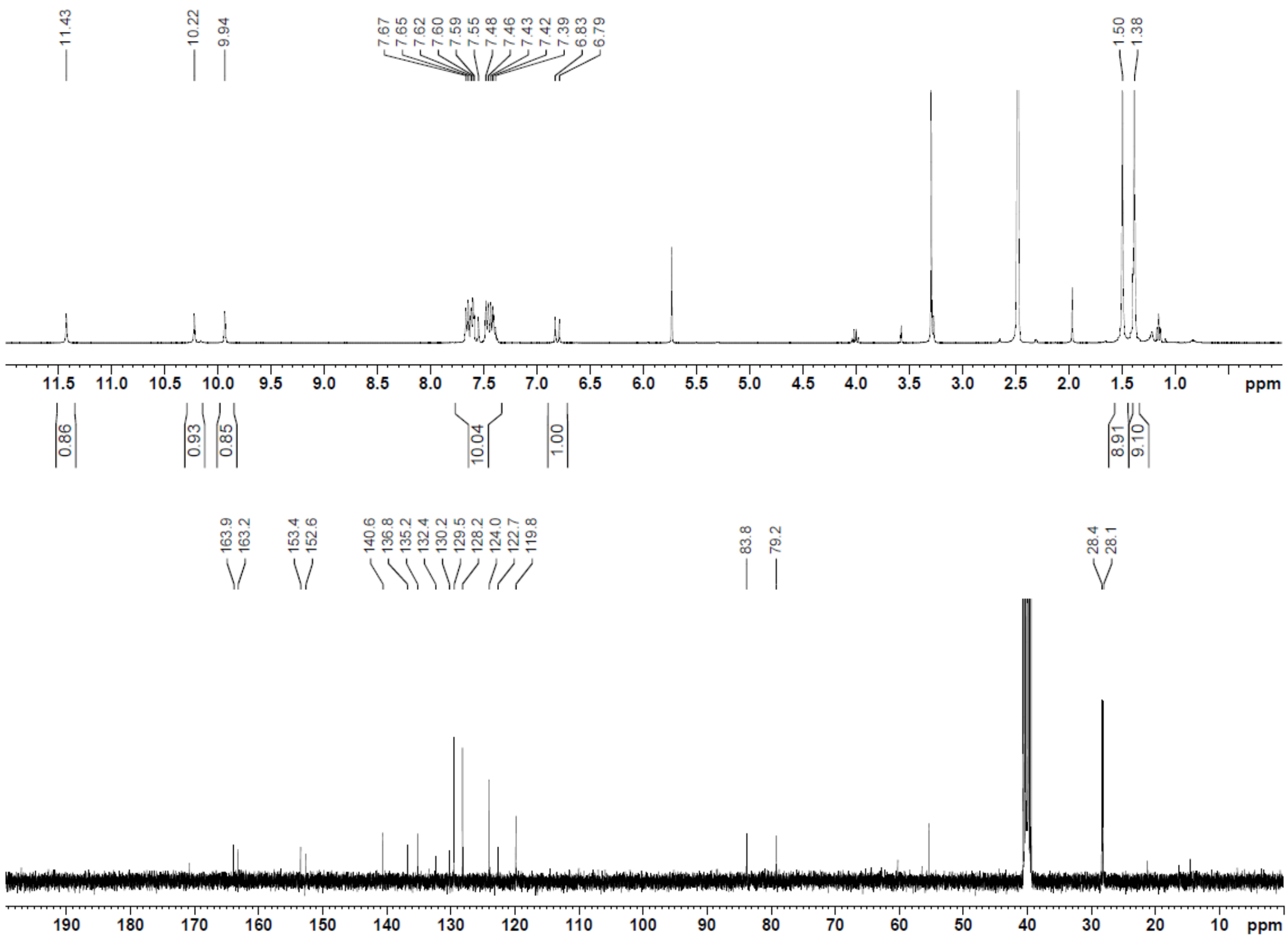

***tert*-Butyl (4-(cyclohex-1-ene-1-carboxamido)phenyl)carbamate**

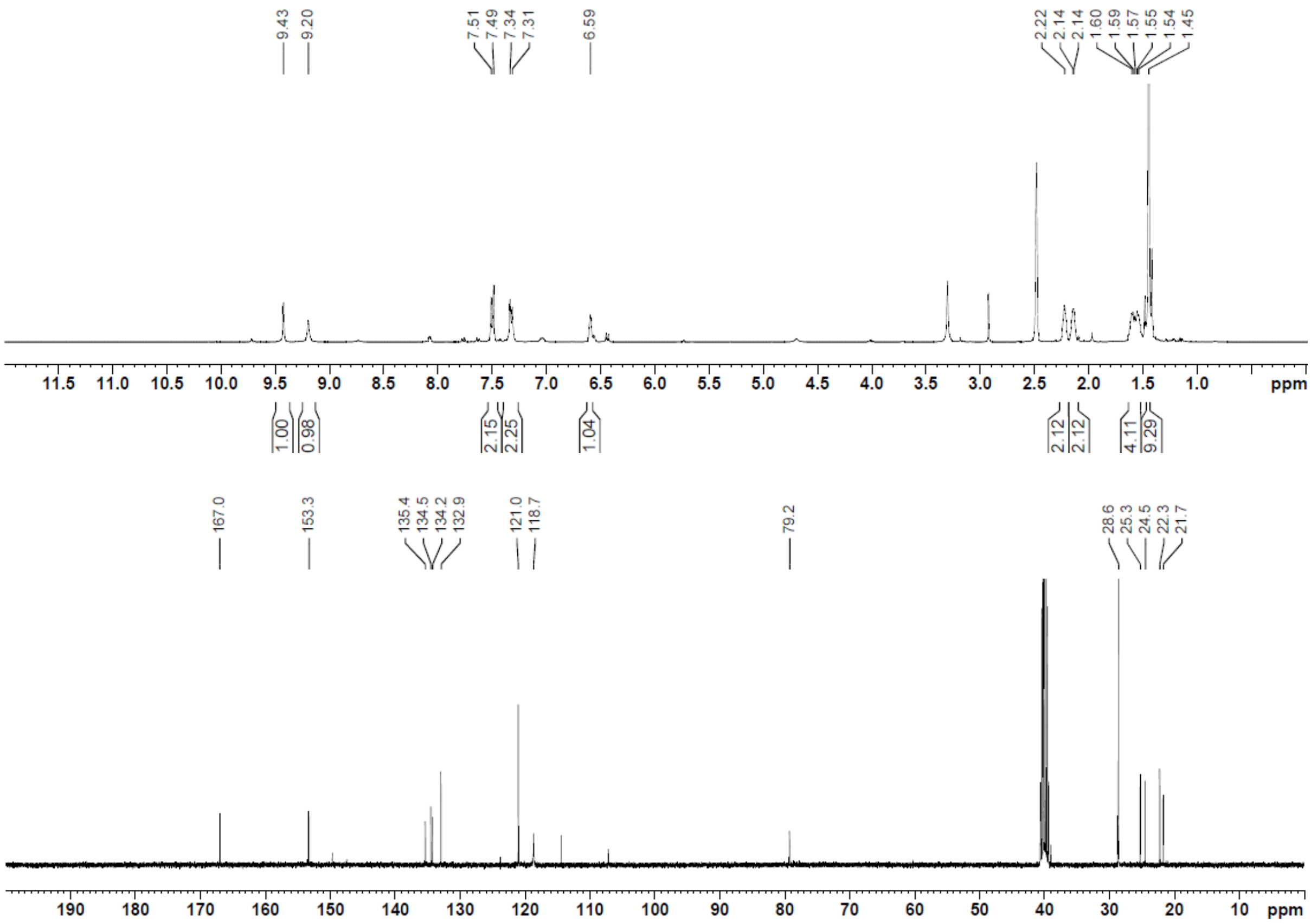

Compound **Boc-25**

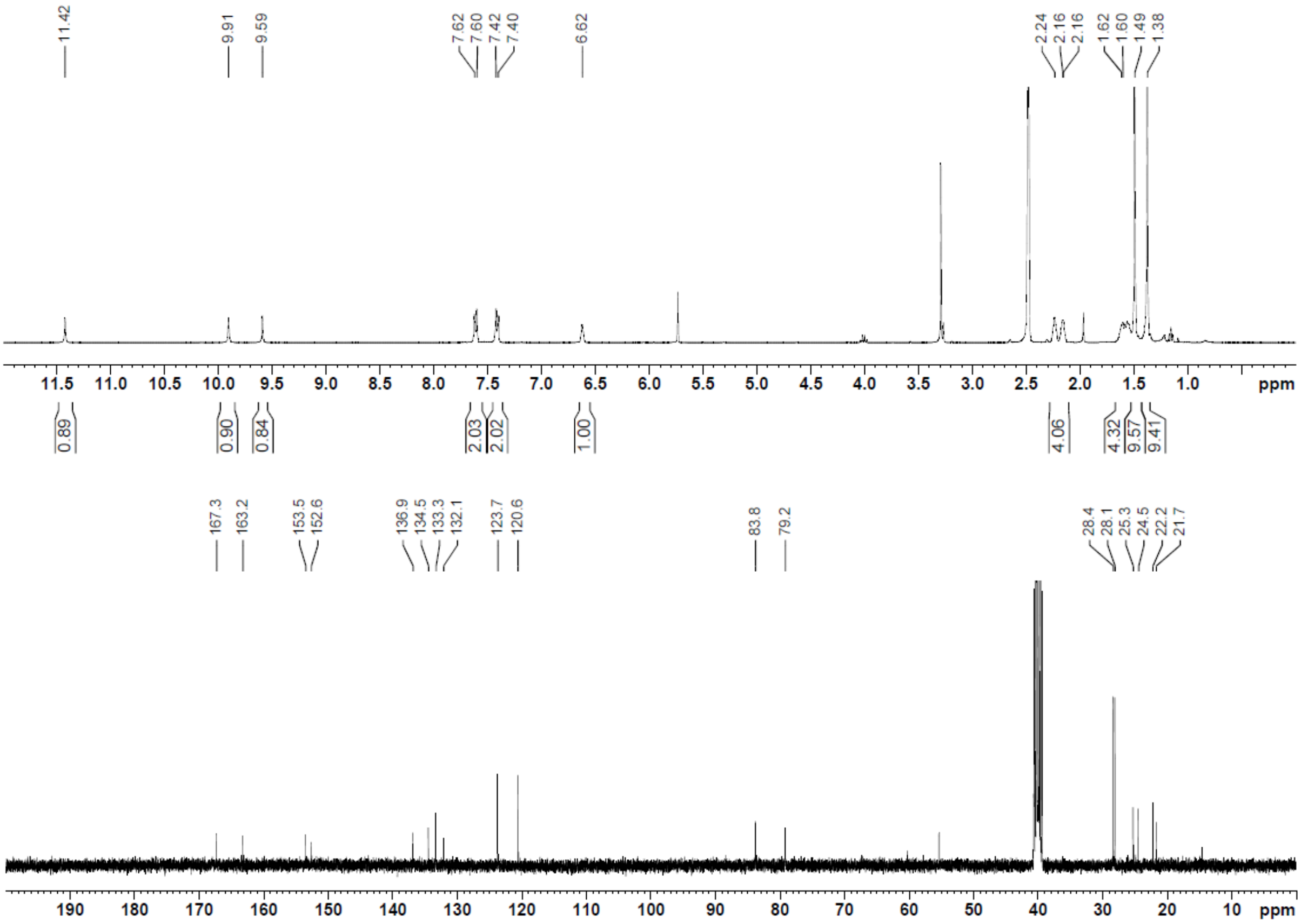

***tert*-Butyl (4-(3,4-dihydro-2H-pyran-6-carboxamido)phenyl)carbamate**

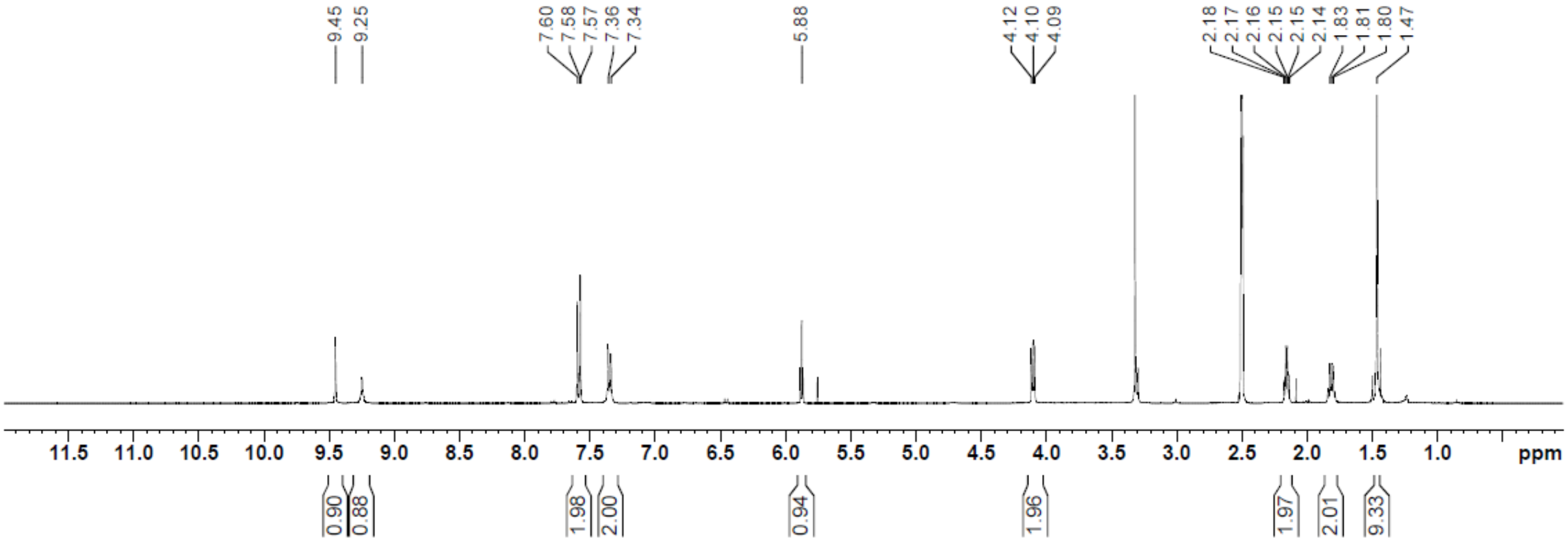

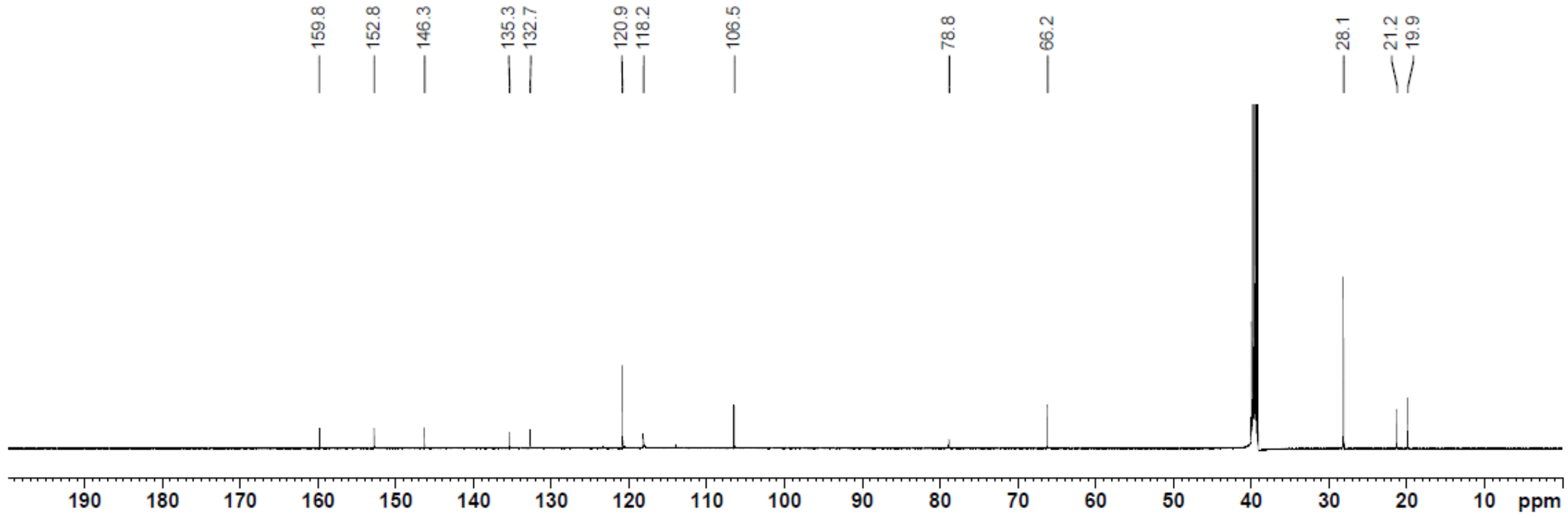

Compound **Boc-27**

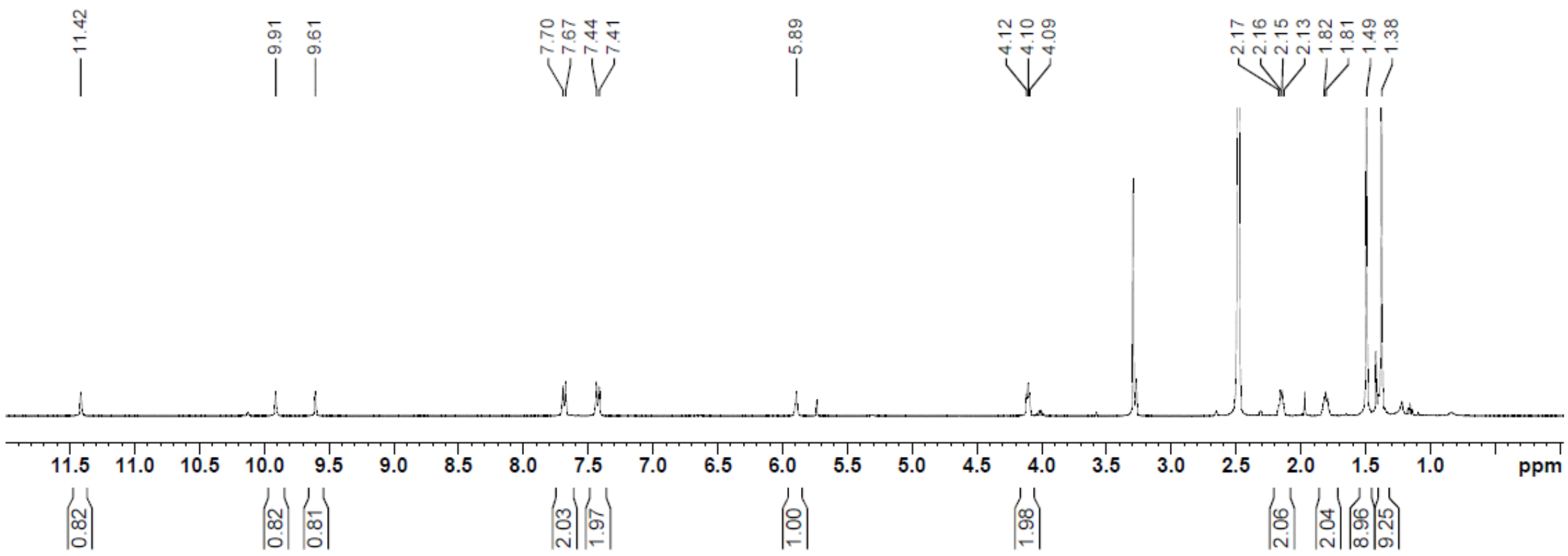

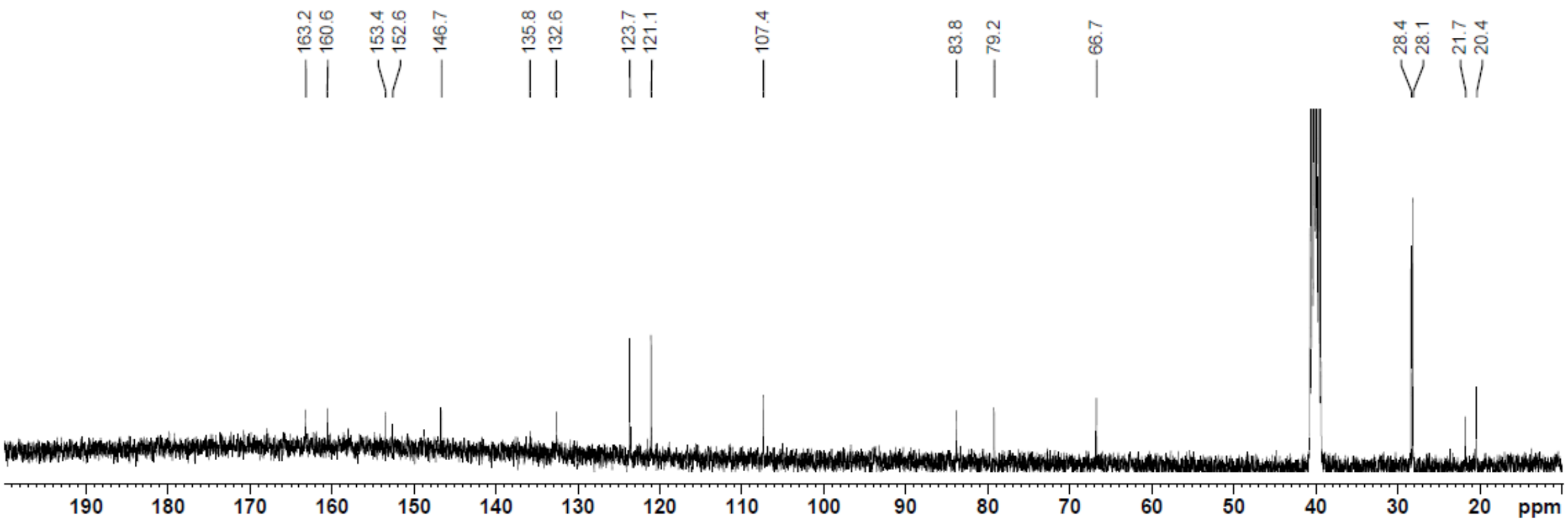

tert-Butyl (4-(3-methylbut-2-enamido)phenyl)carbamate

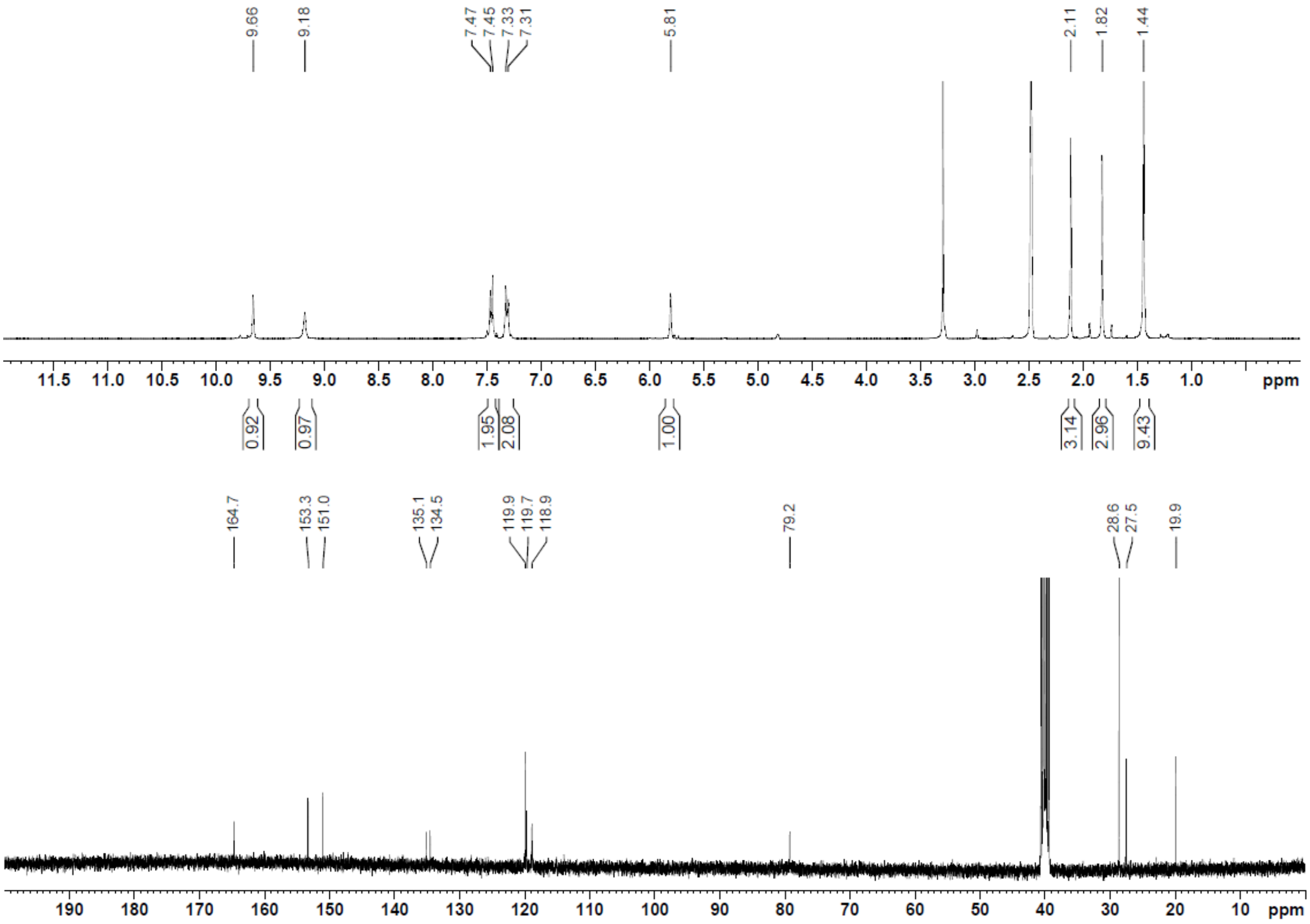

Compound **Boc-24**

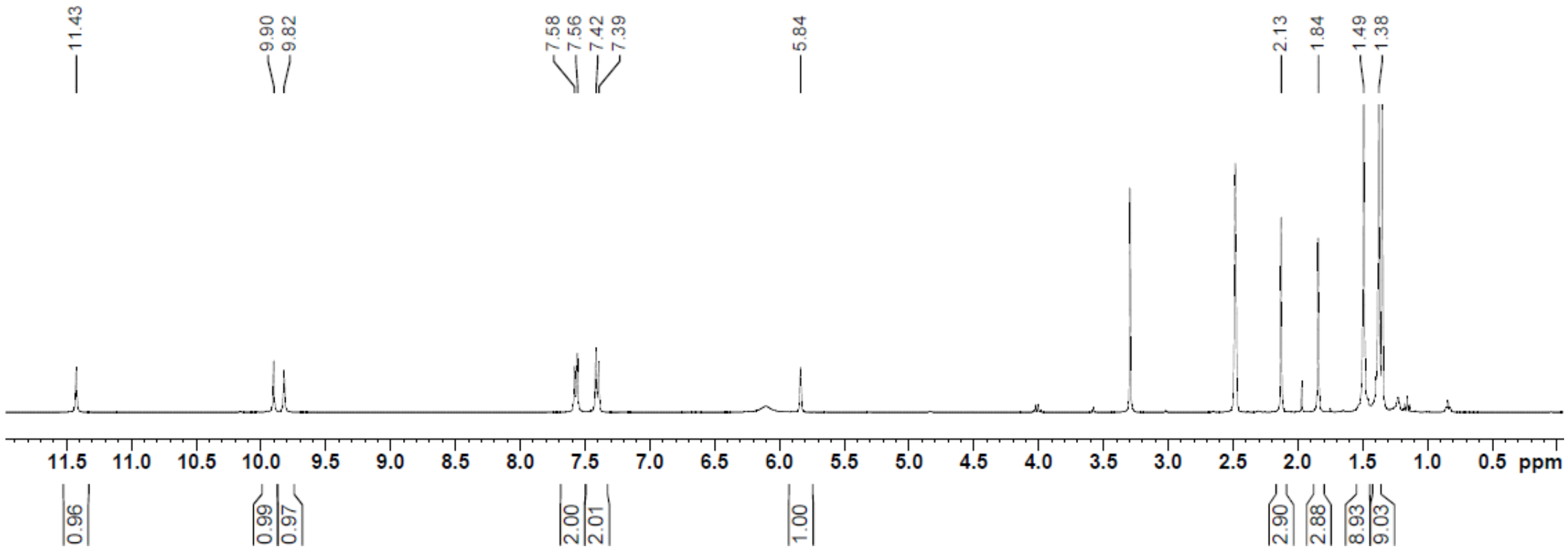

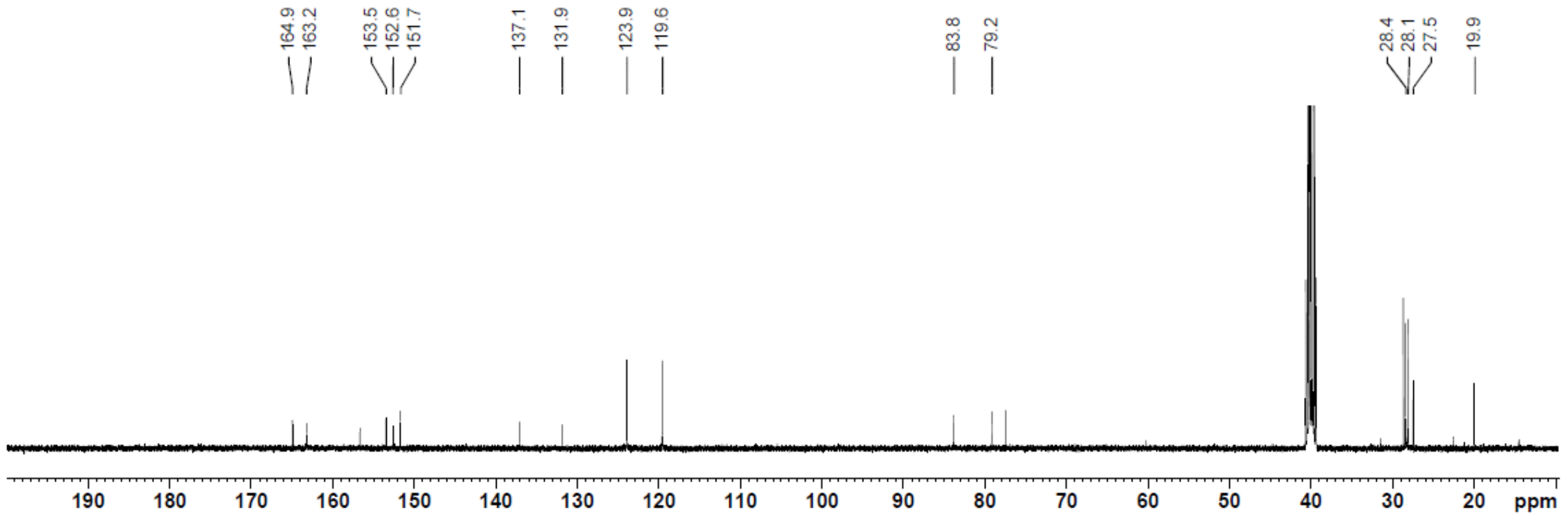

tert-Butyl (E)-(4-(but-2-enamido)phenyl)carbamate

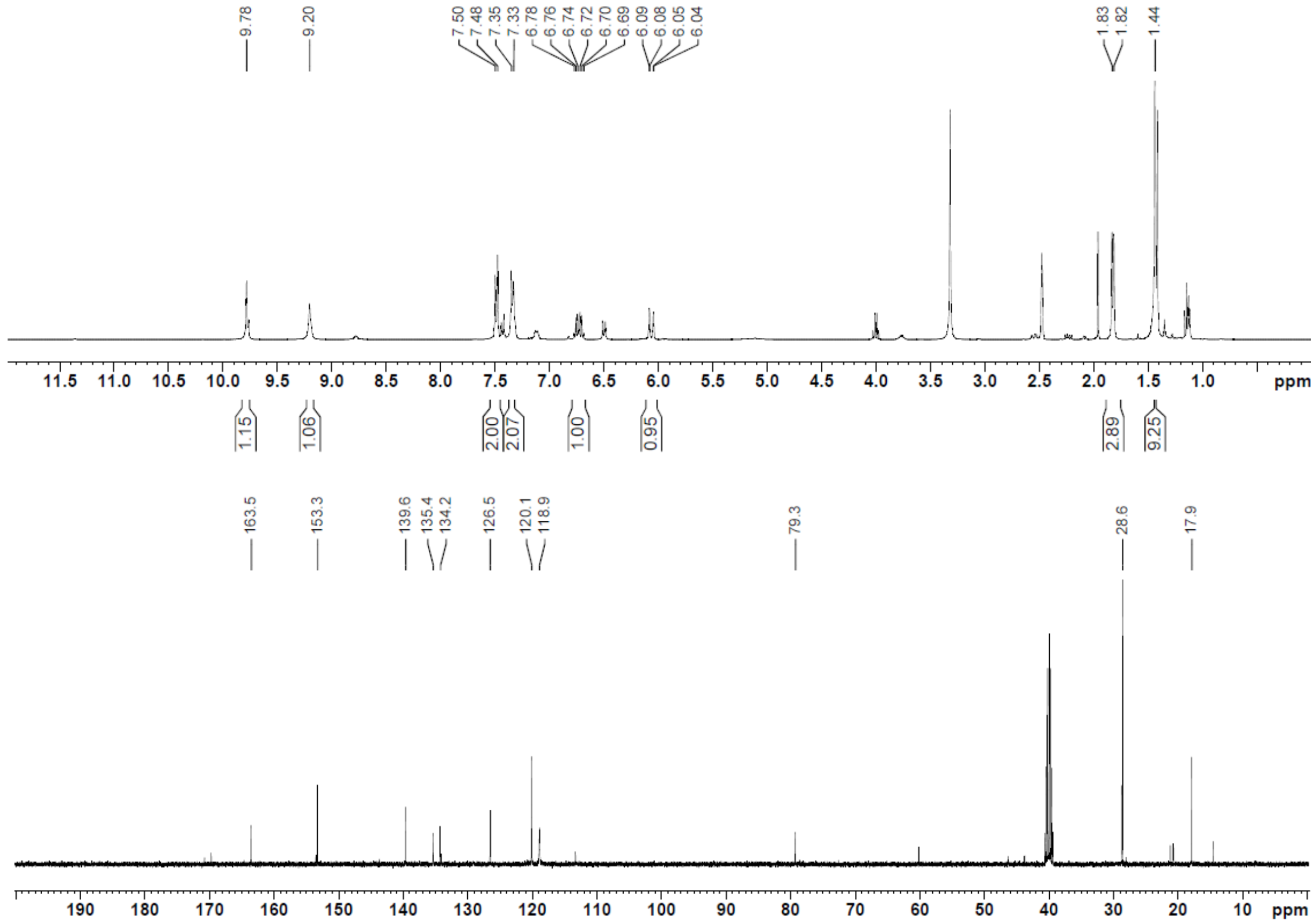

Compound **Boc-21**

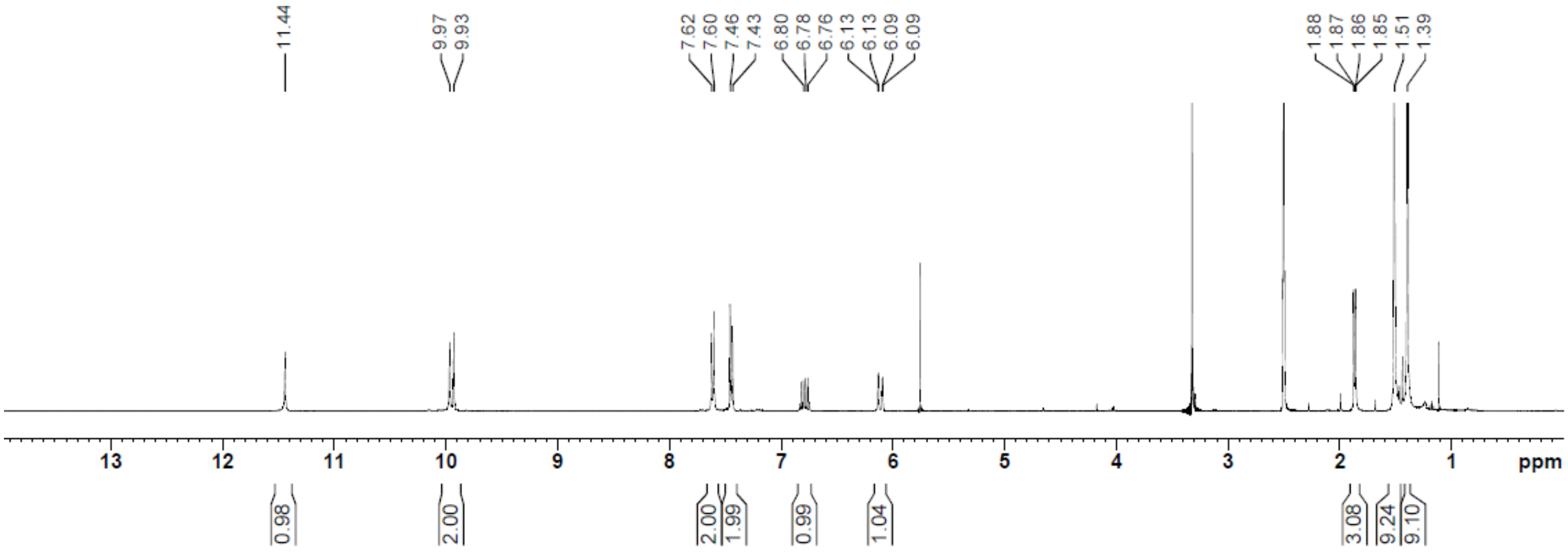

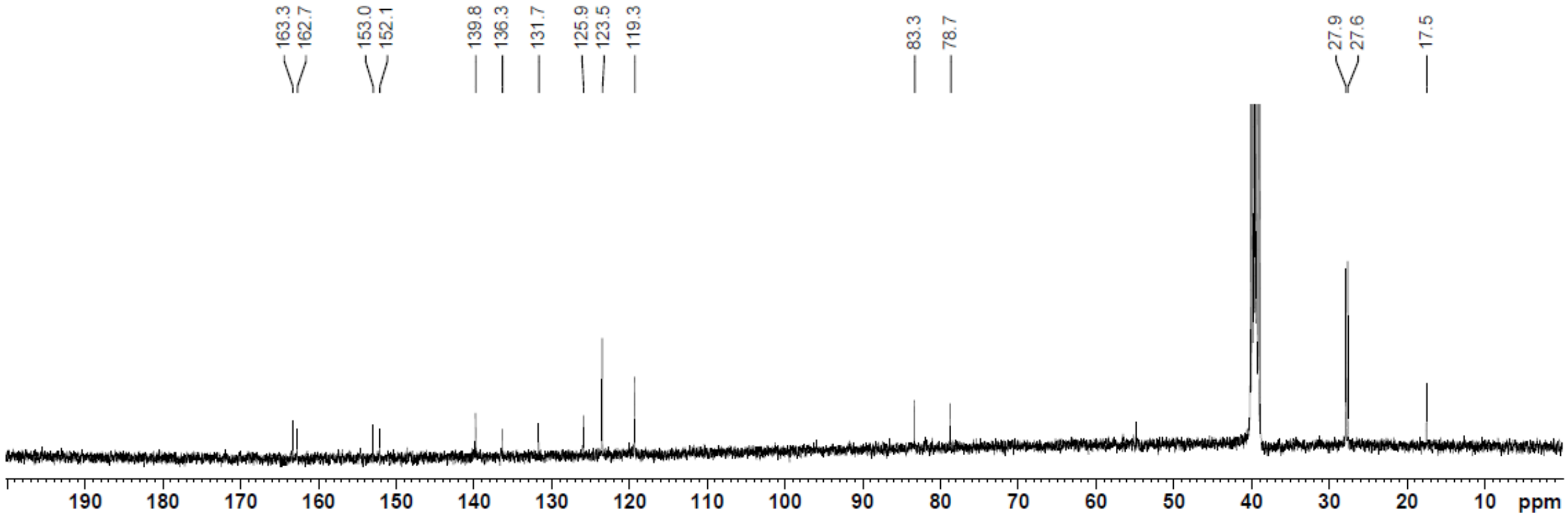

***tert*-Butyl (4-(but-2-ynamido)phenyl)carbamate**

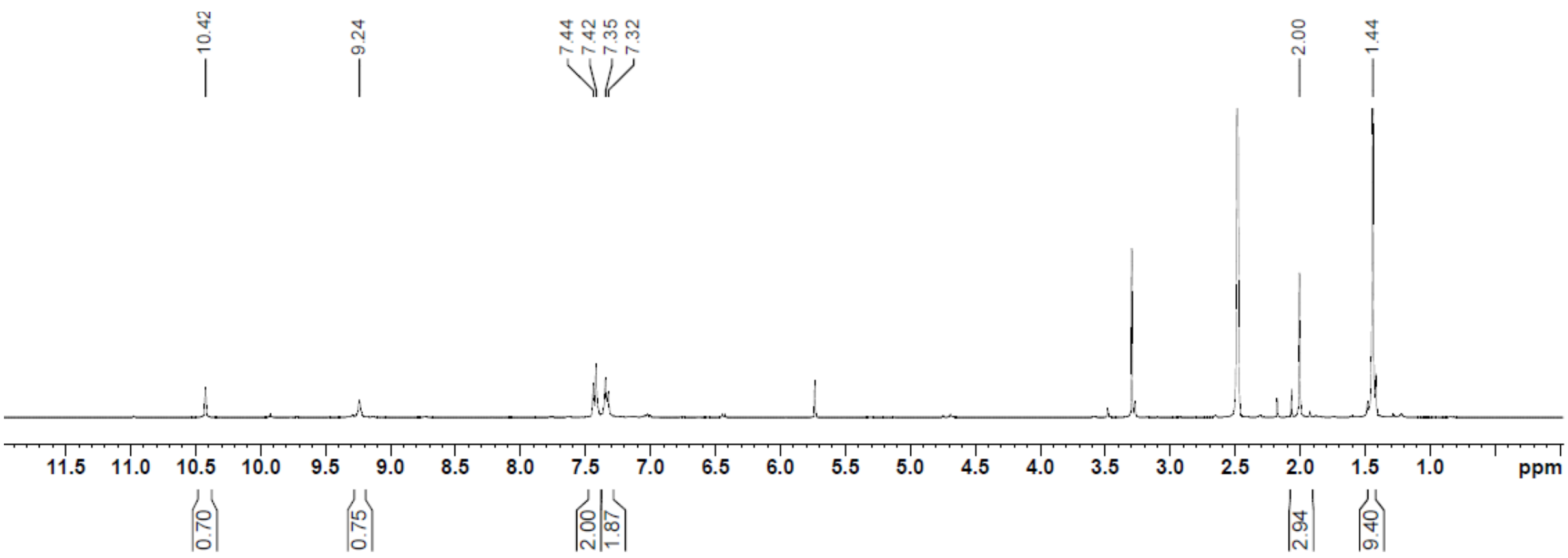

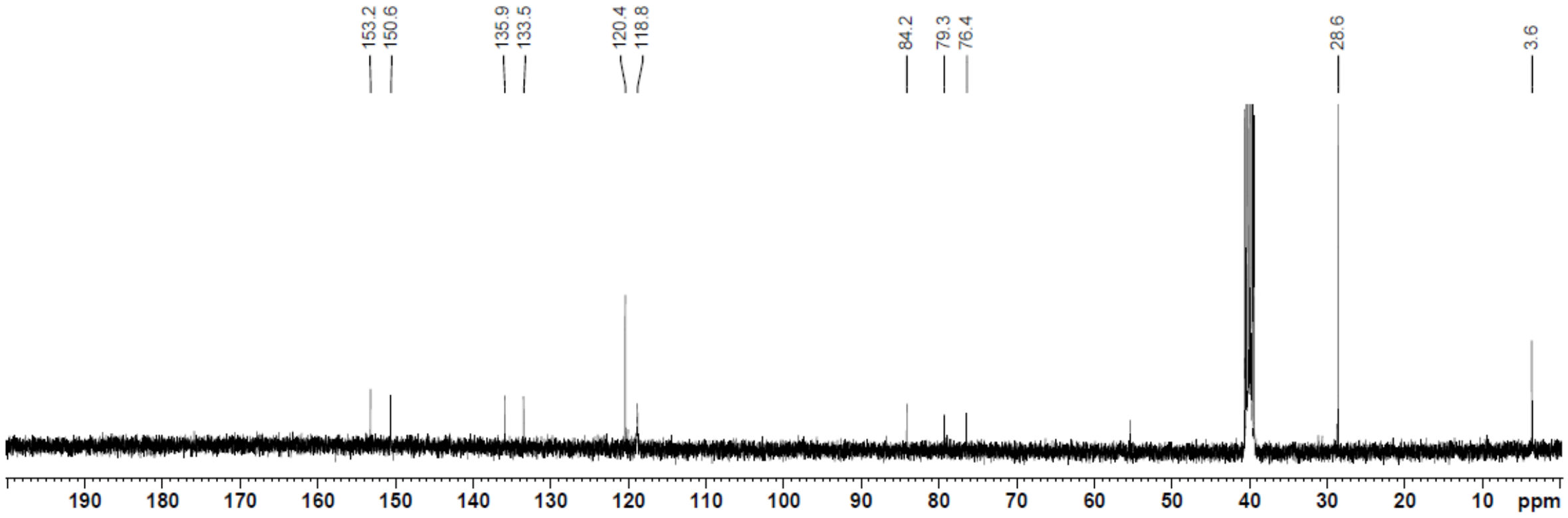

Compound **Boc-32**

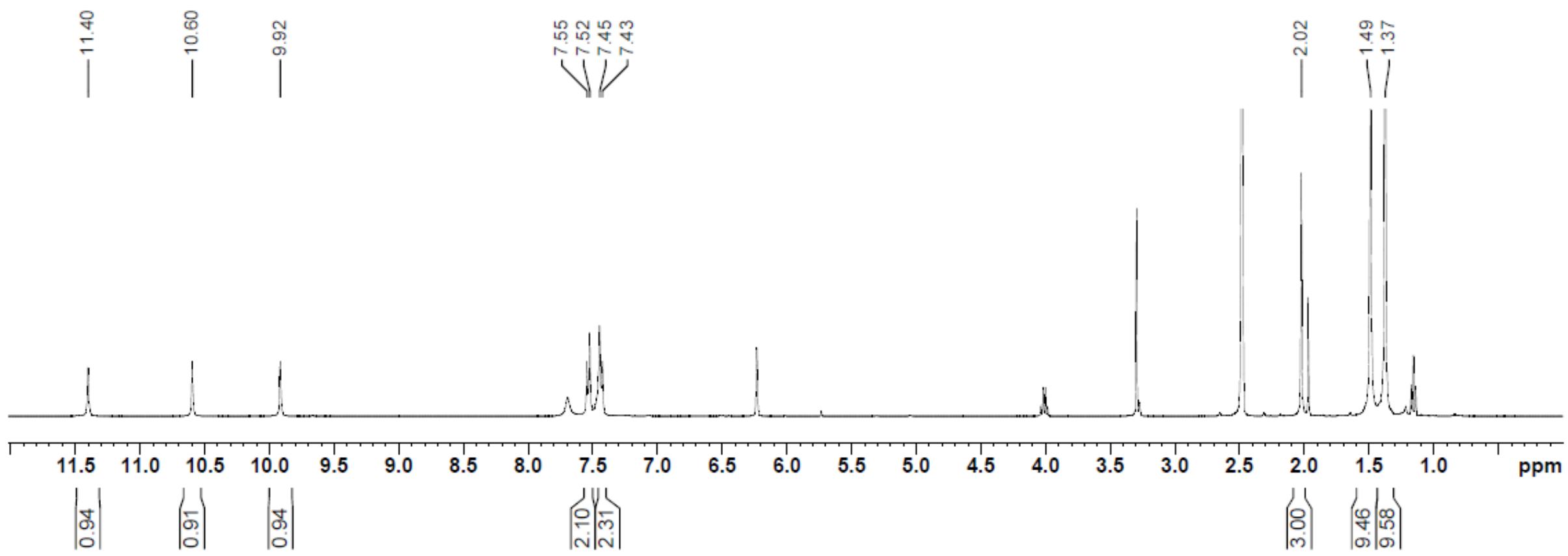

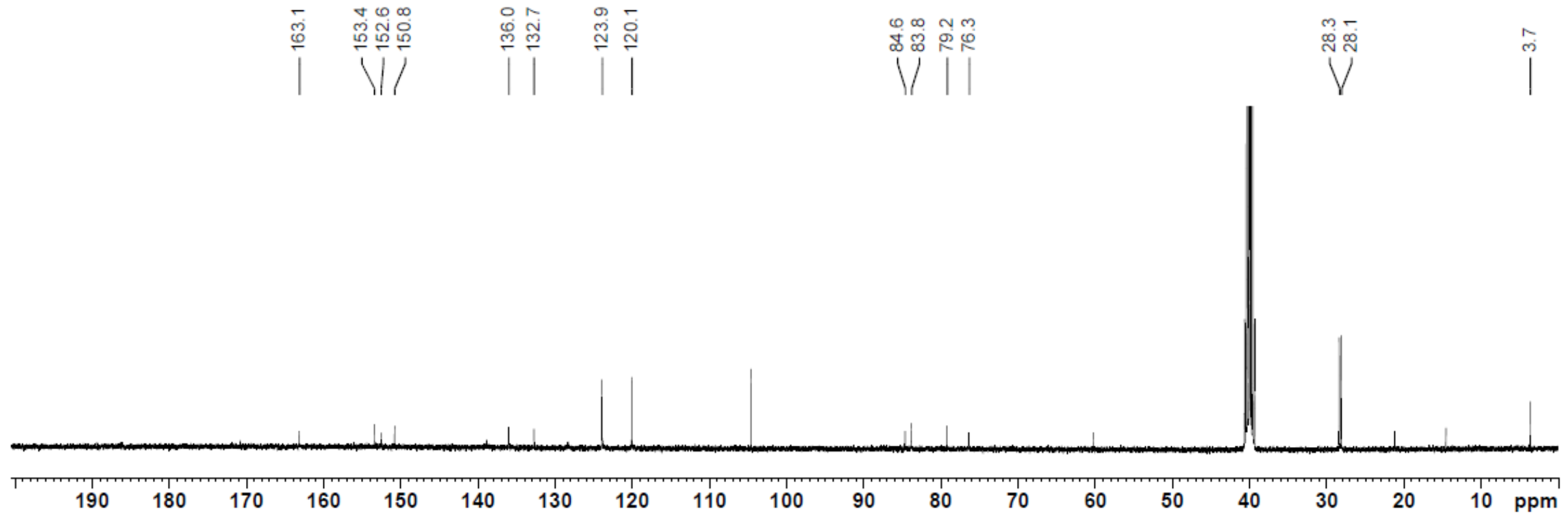

Compound **61**

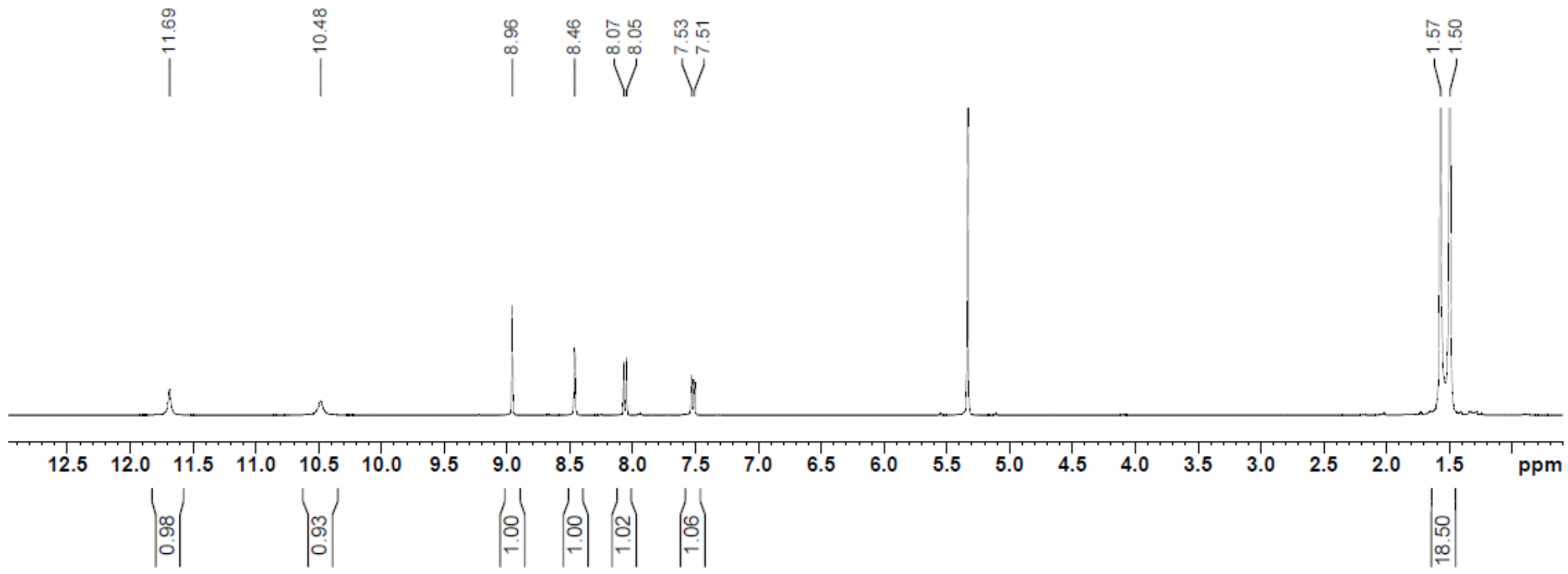

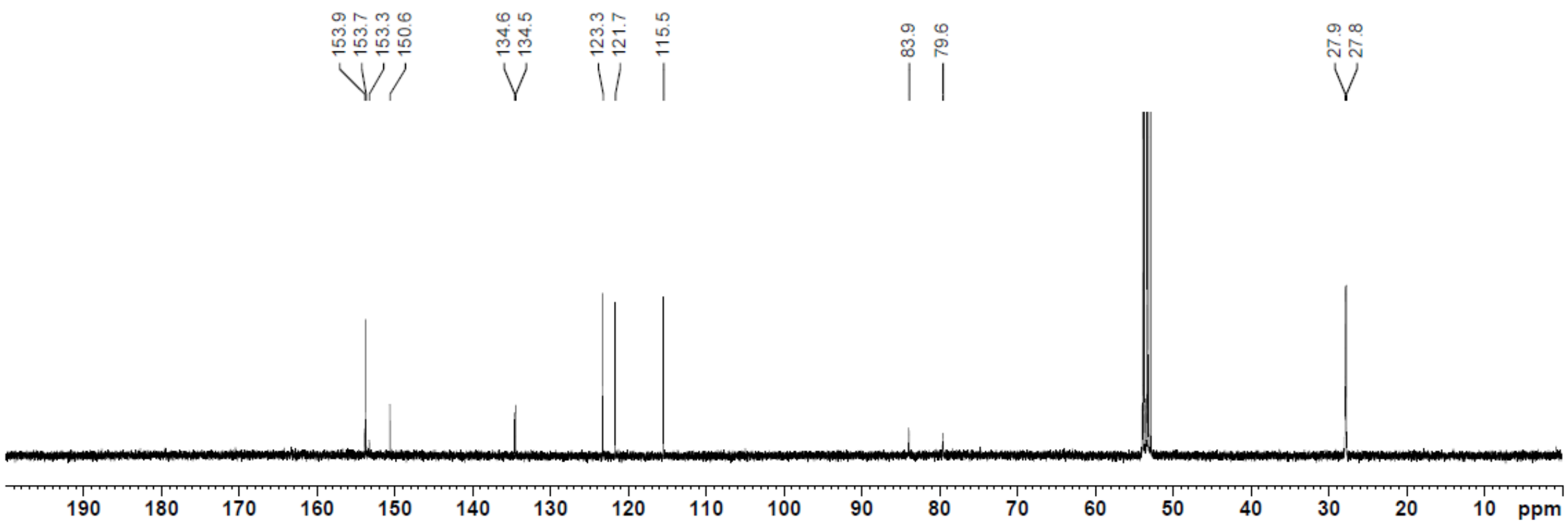

6-nitro-*N*-(4-Nitrophenyl)-2-benzothiazolamine

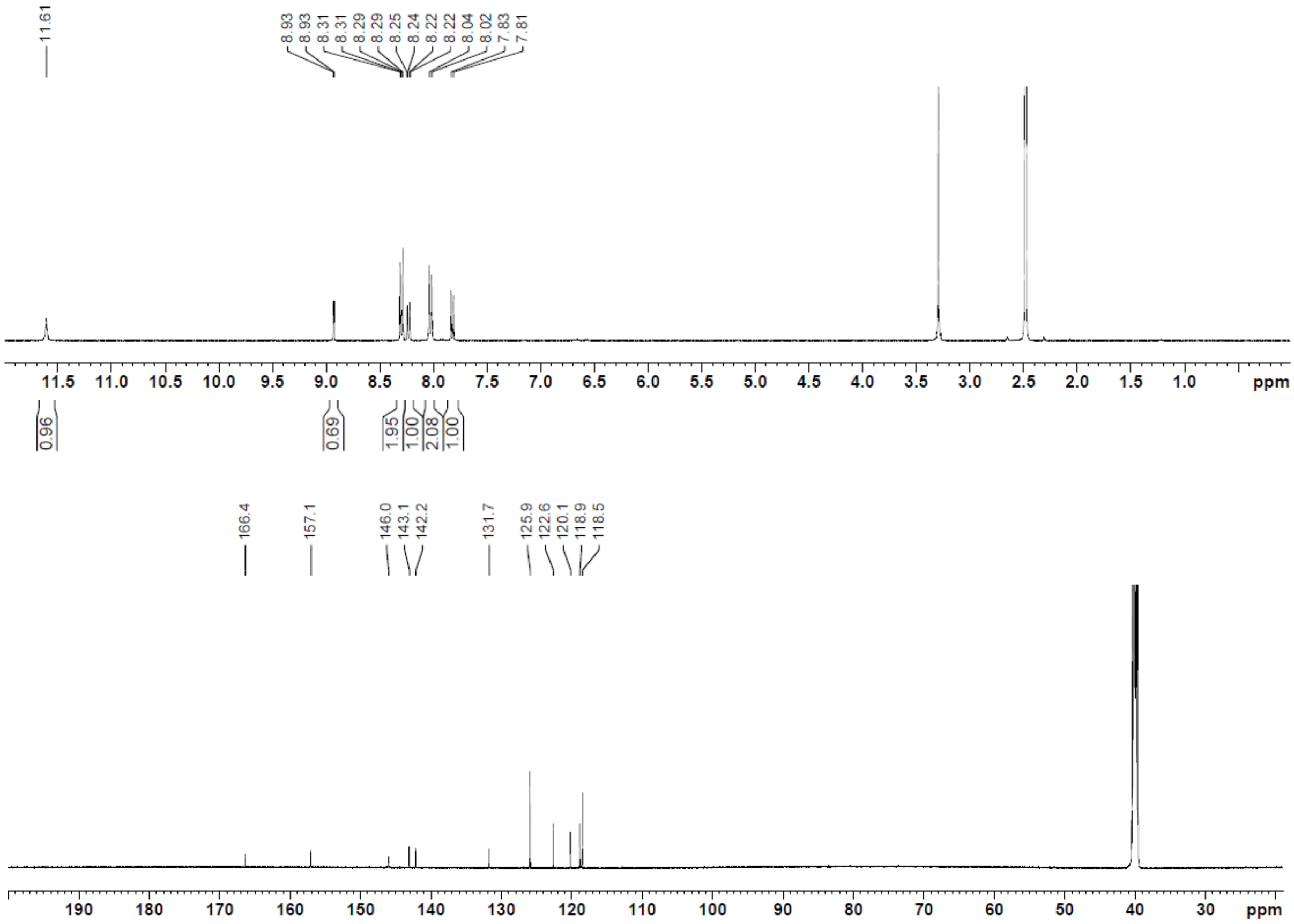

Compound **Boc-39**

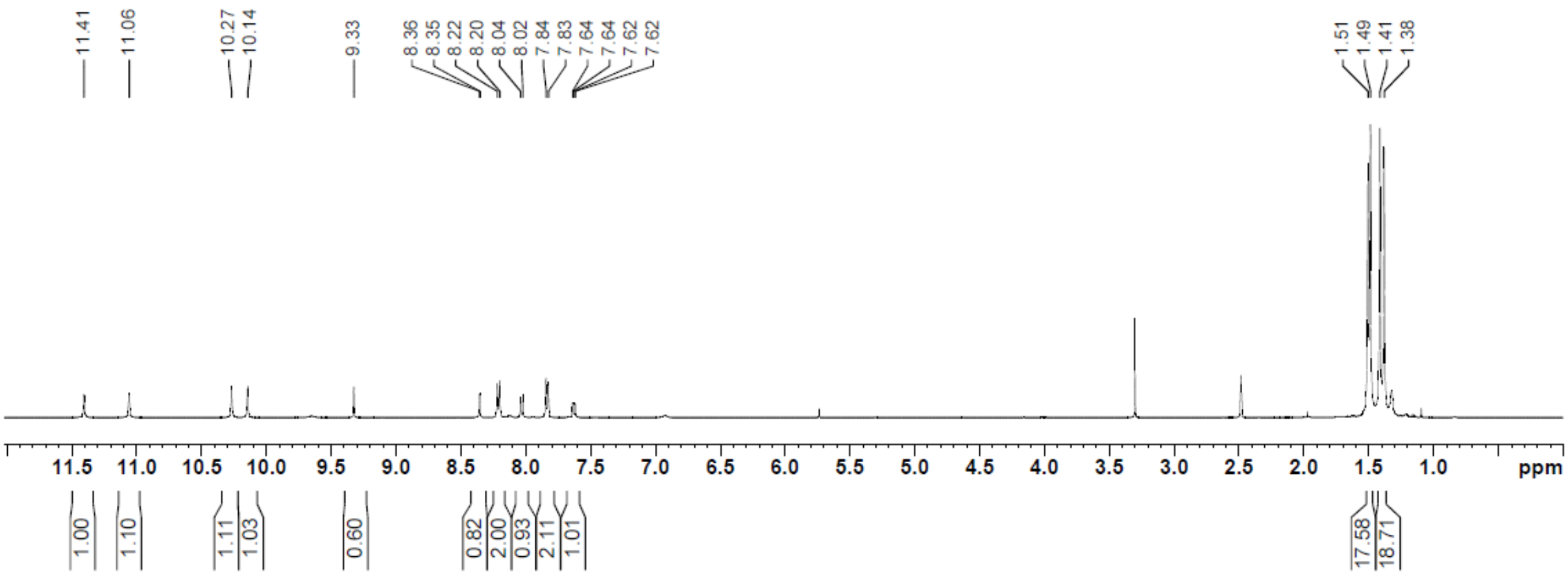

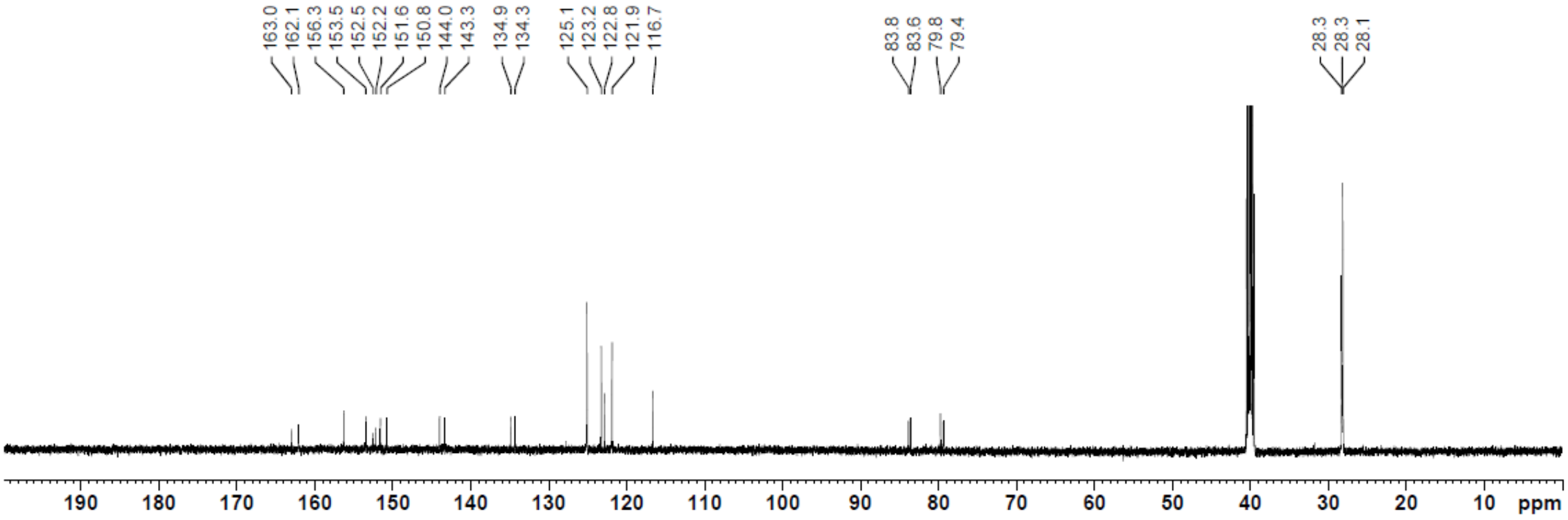

Compound **Boc-15**

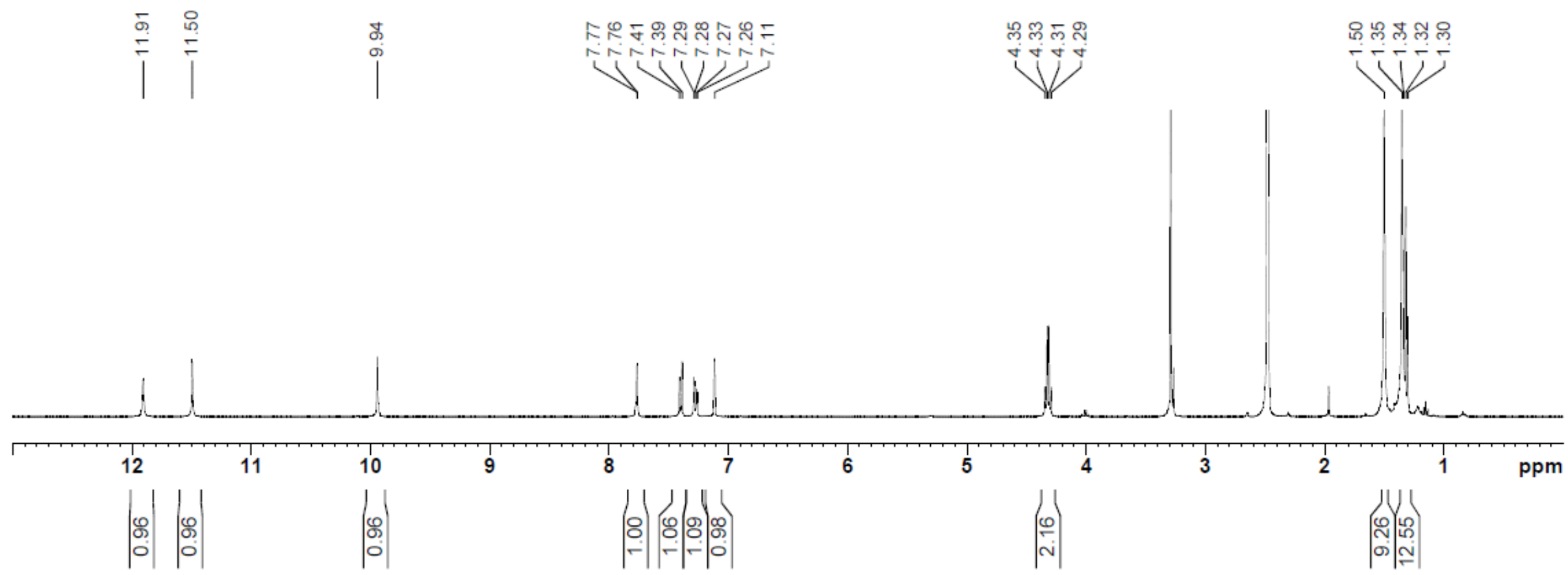

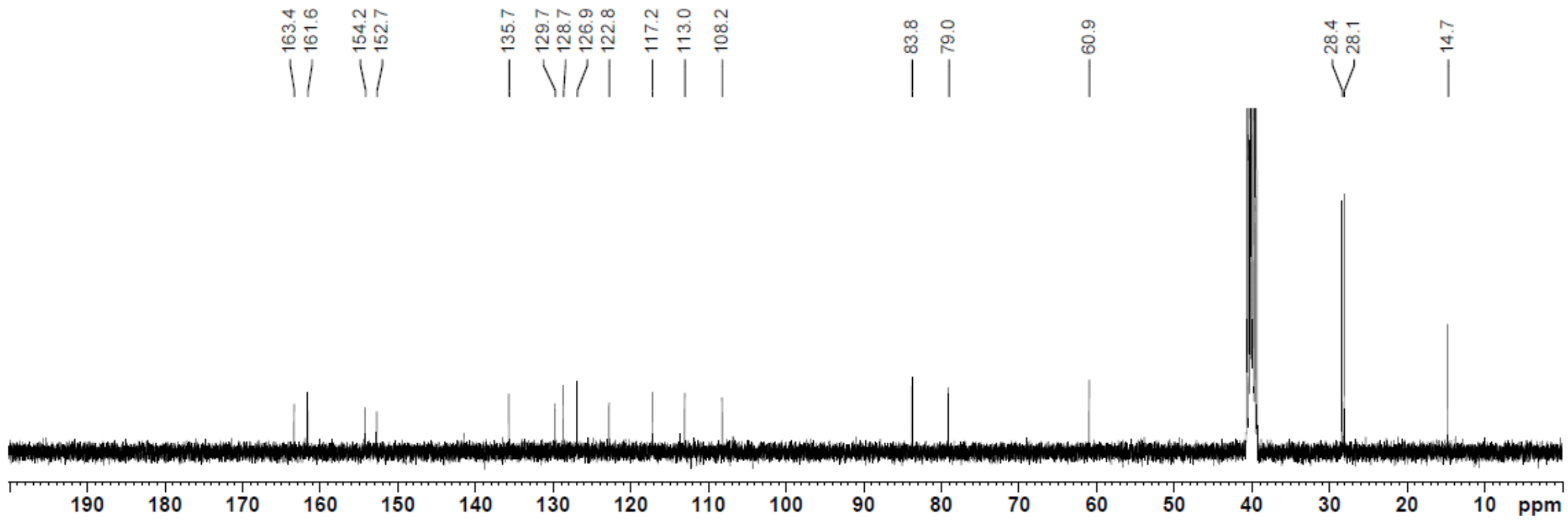

Compound **Boc-16**

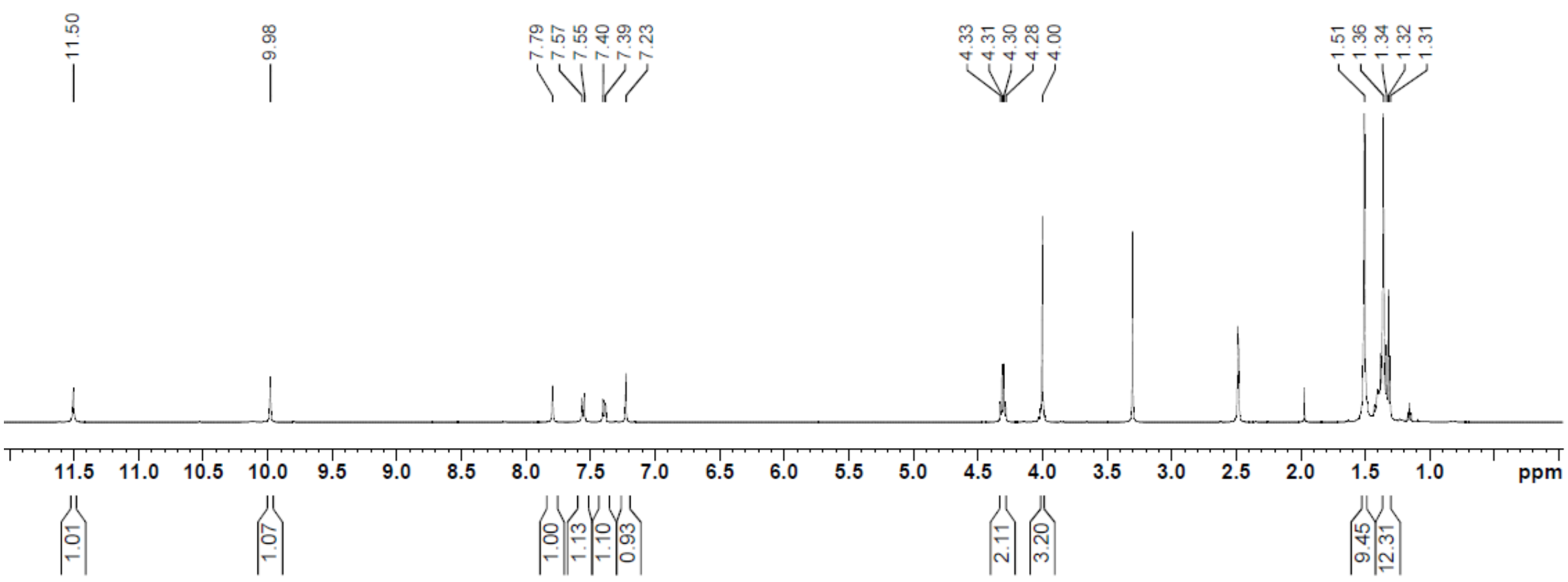

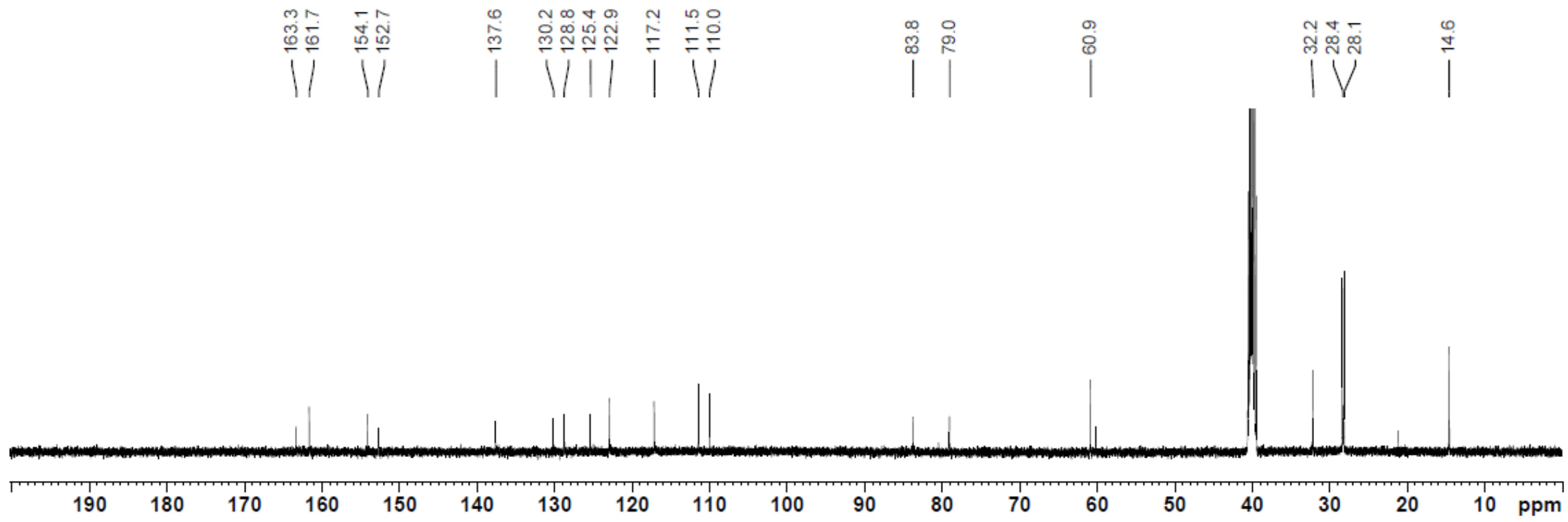

Compound **Boc-18**

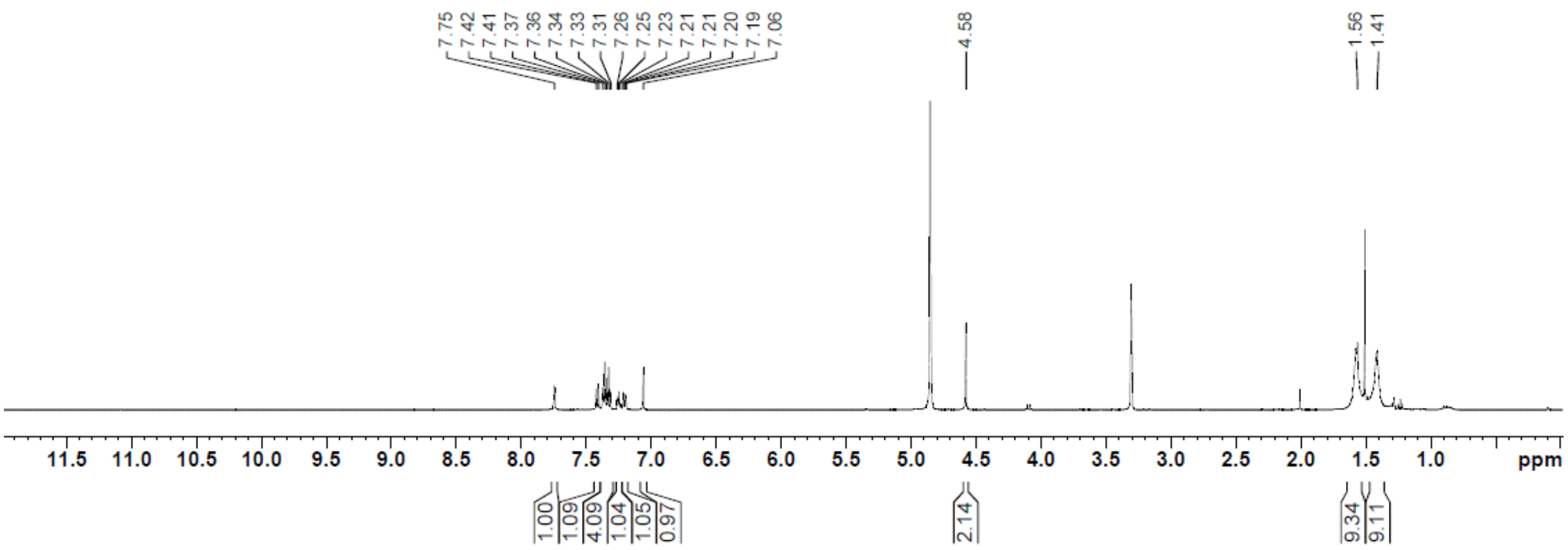

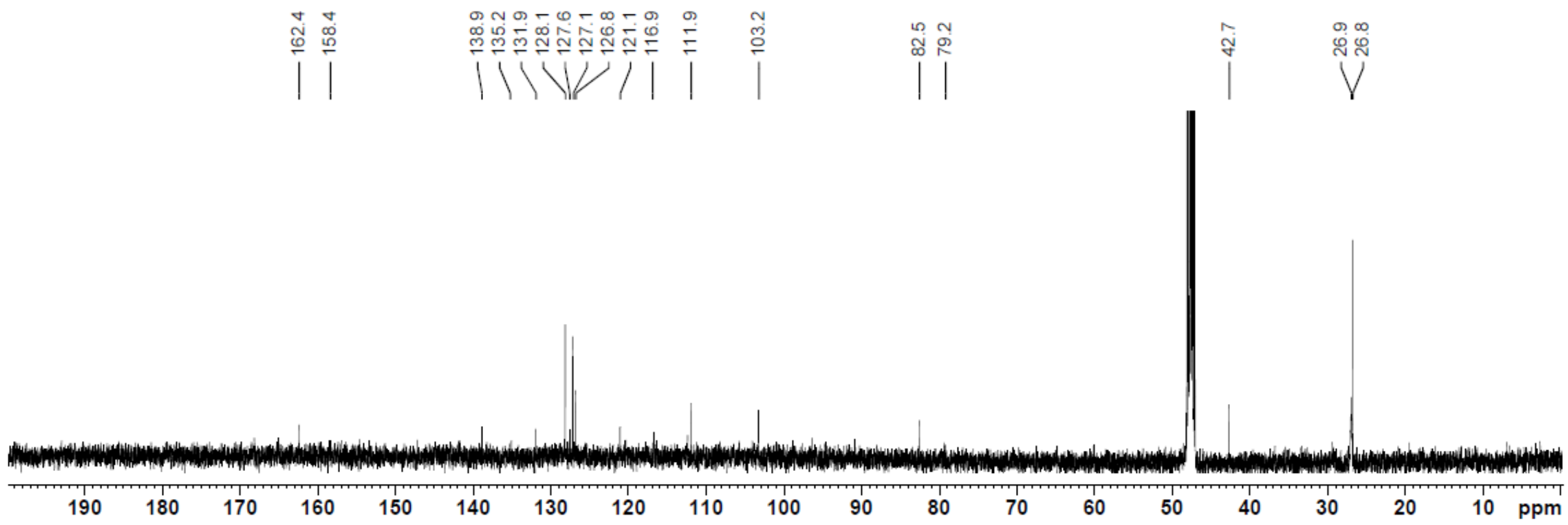

Compound **Boc-17**

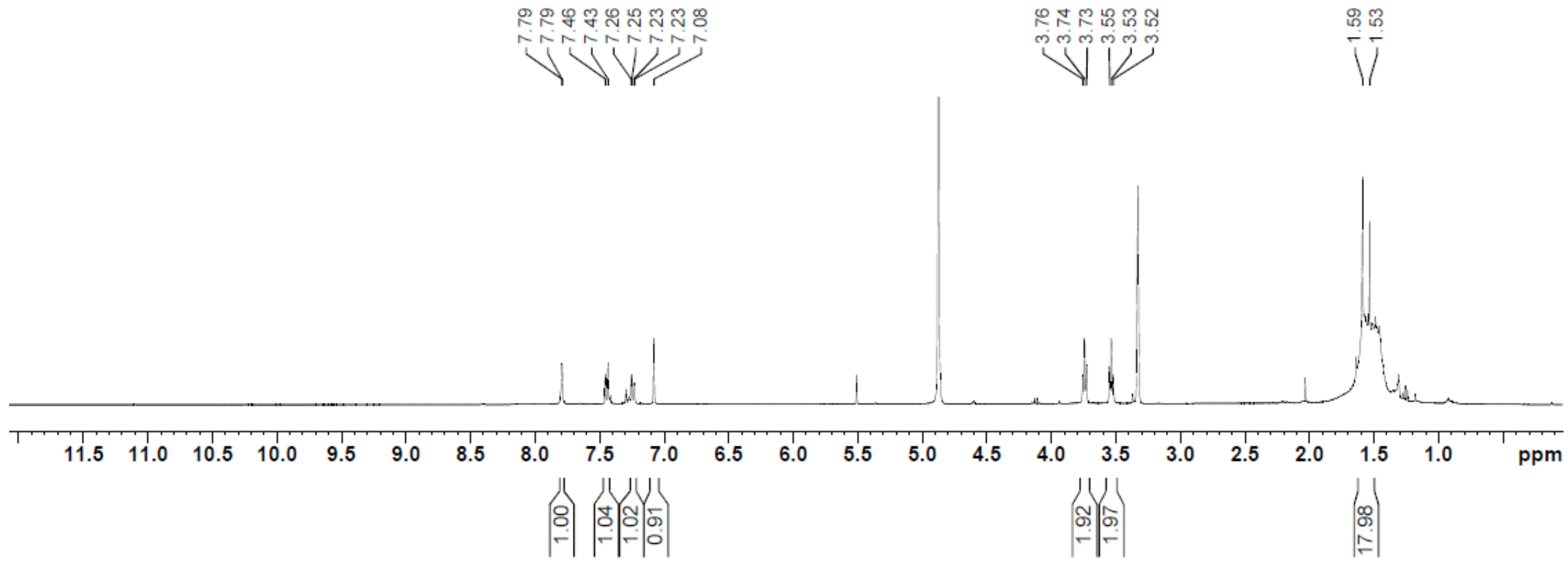

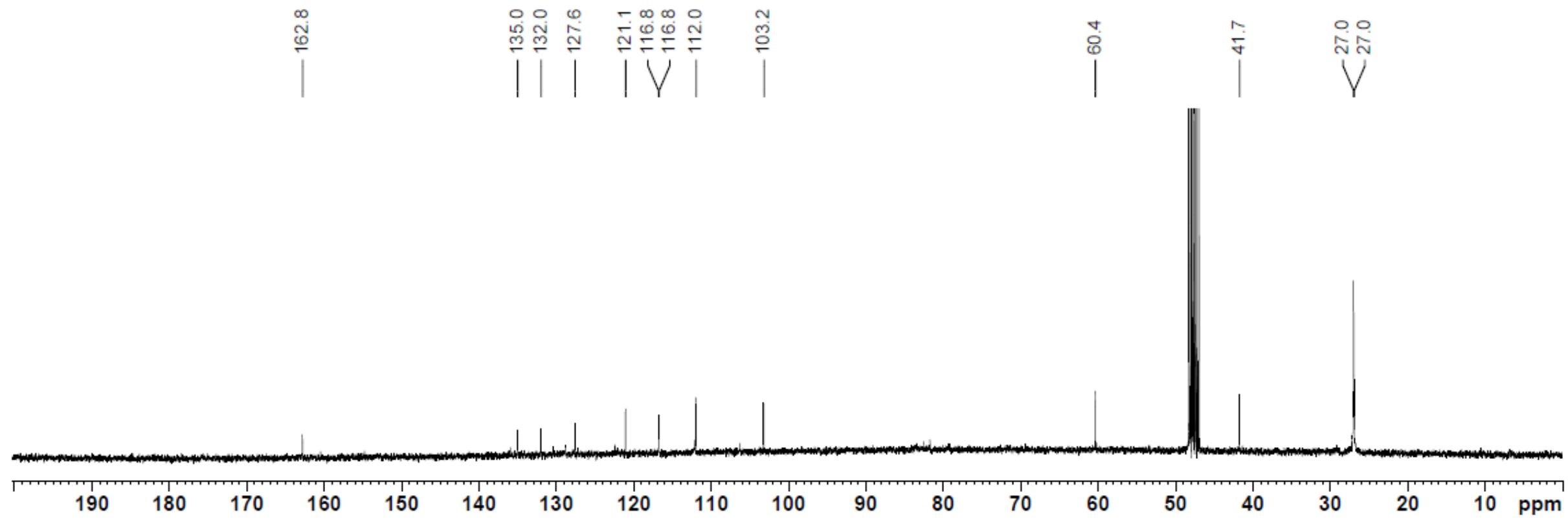

2,2'-((4-nitrophenyl)methylene)bis(1*H*-indole)

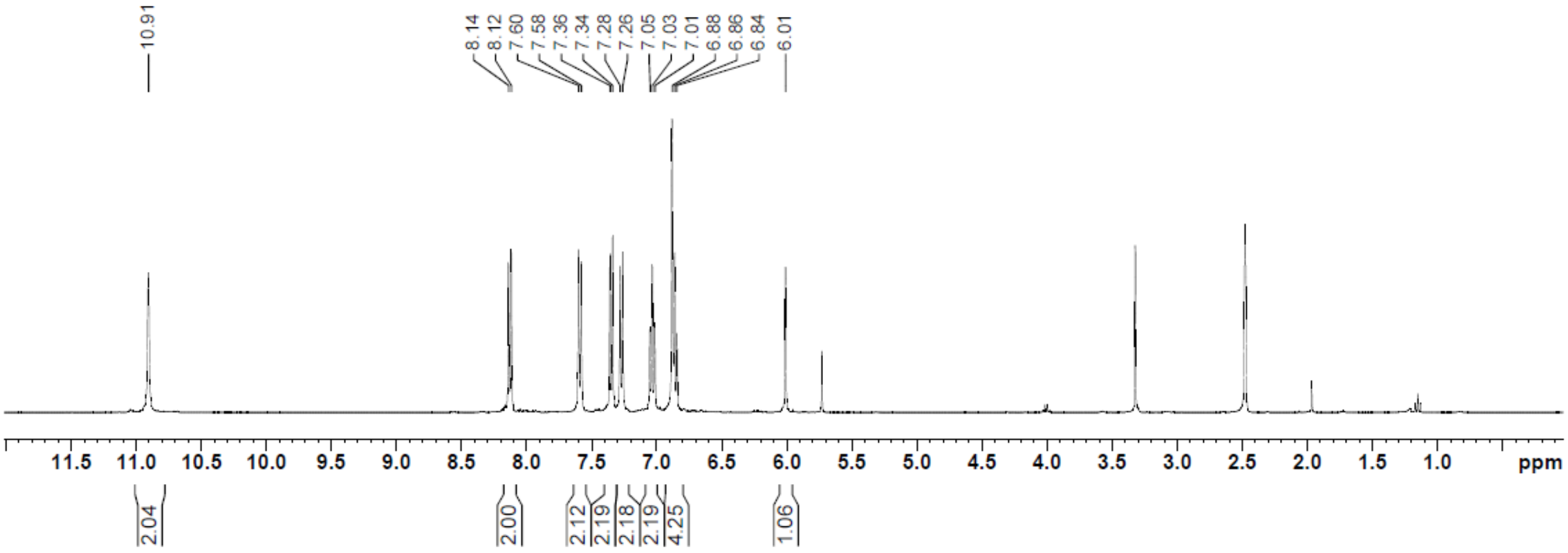

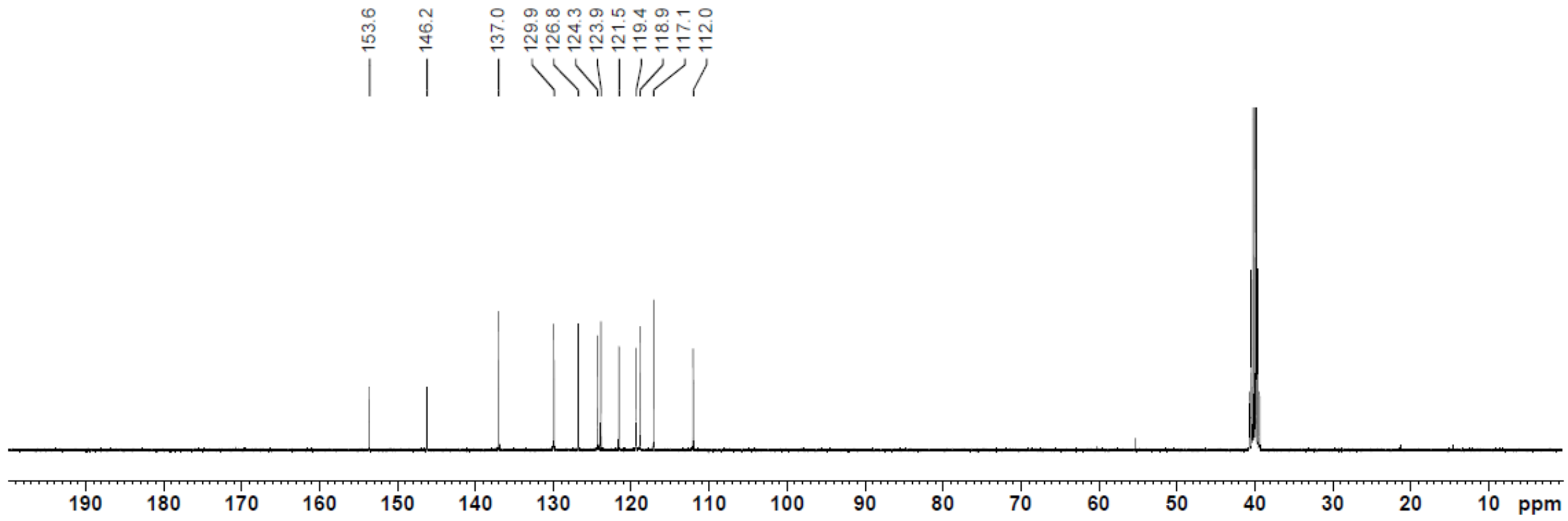

Compound **Boc-20**

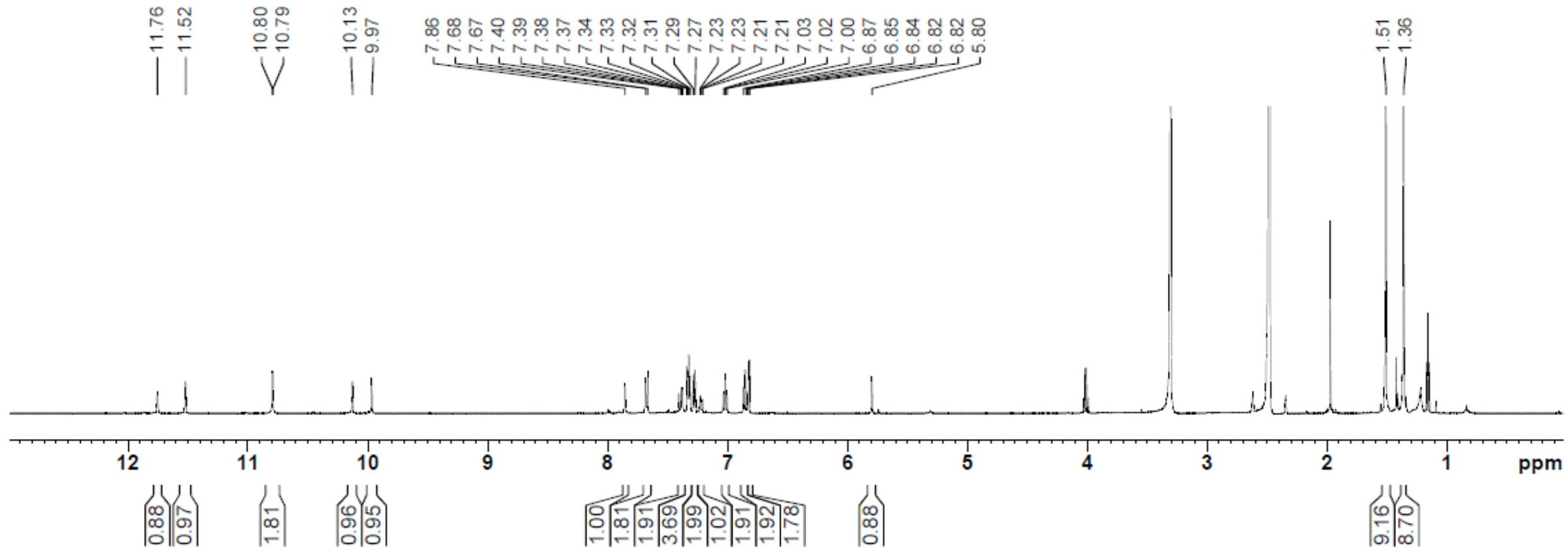

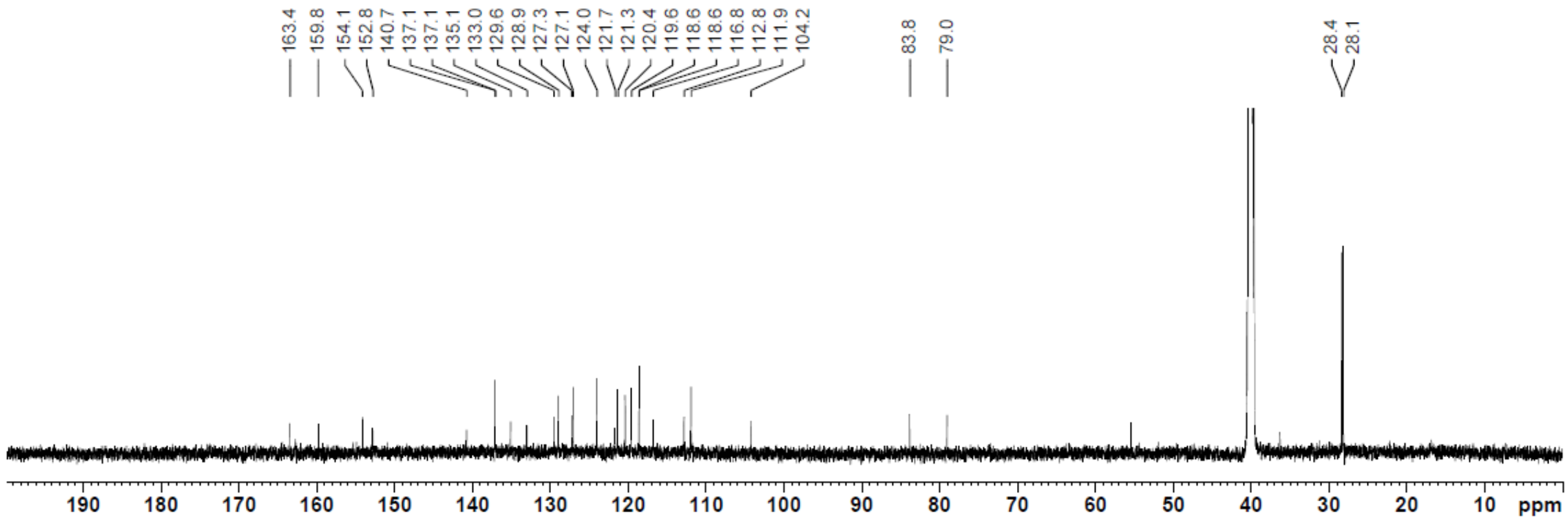

Compound **Boc-7**

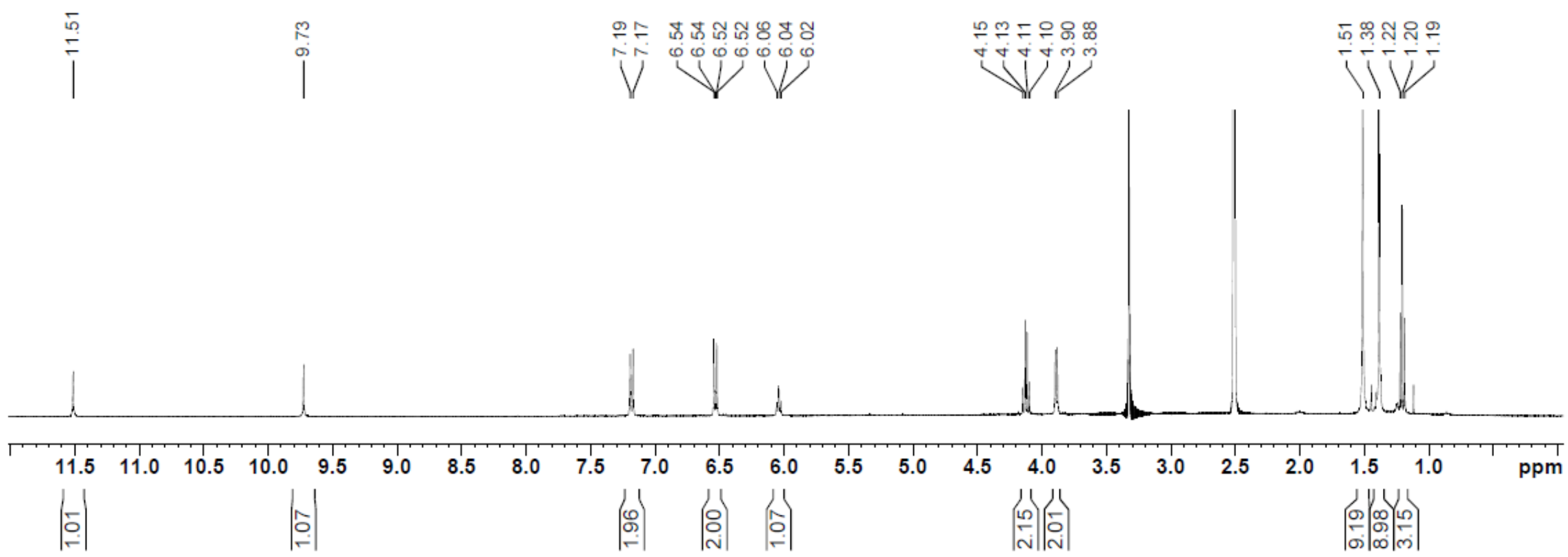

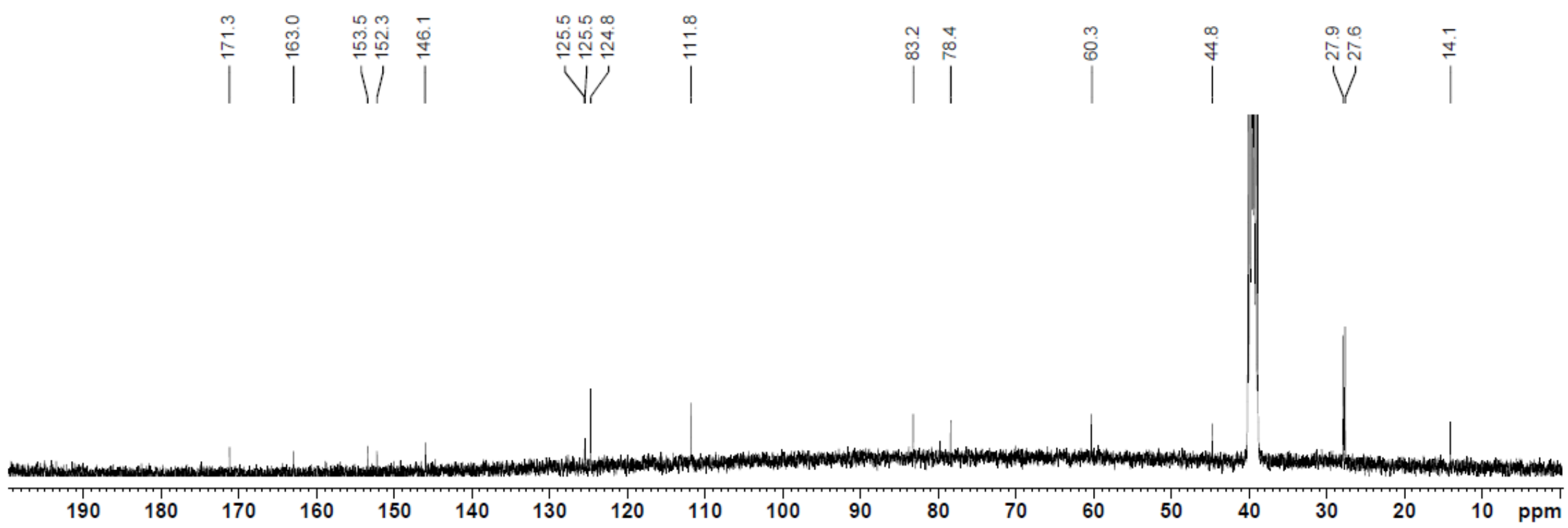

***N*-methoxy-*N*-methyl acetamide**

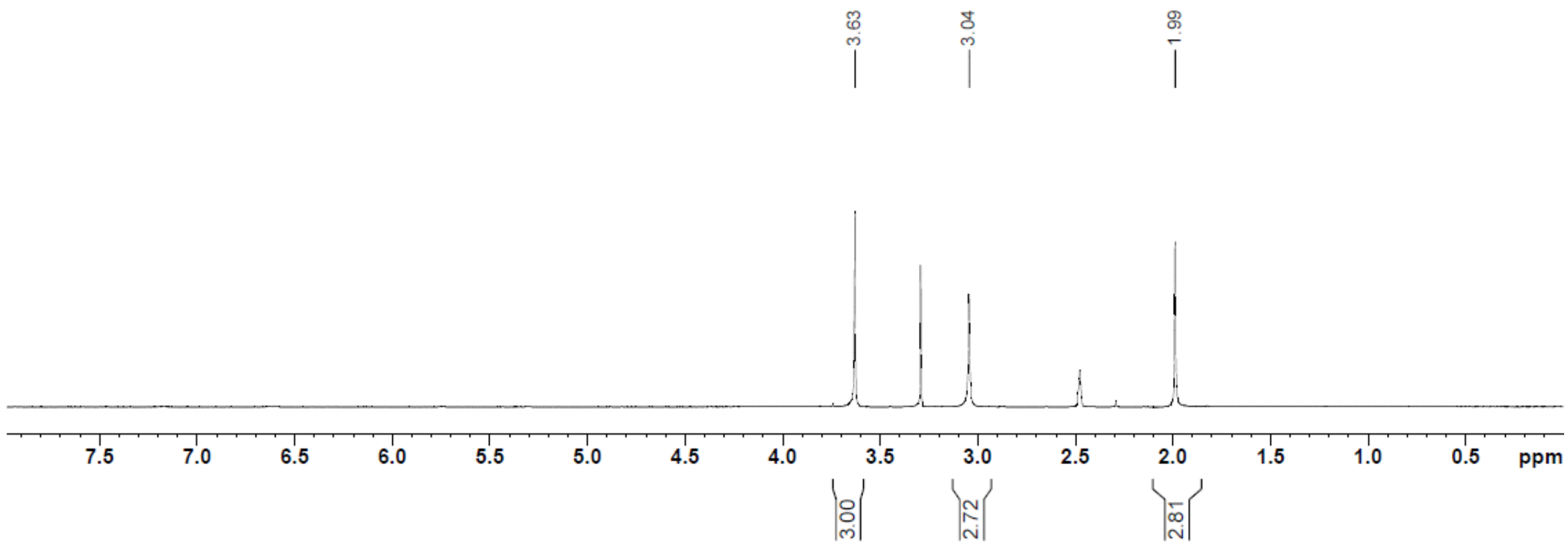

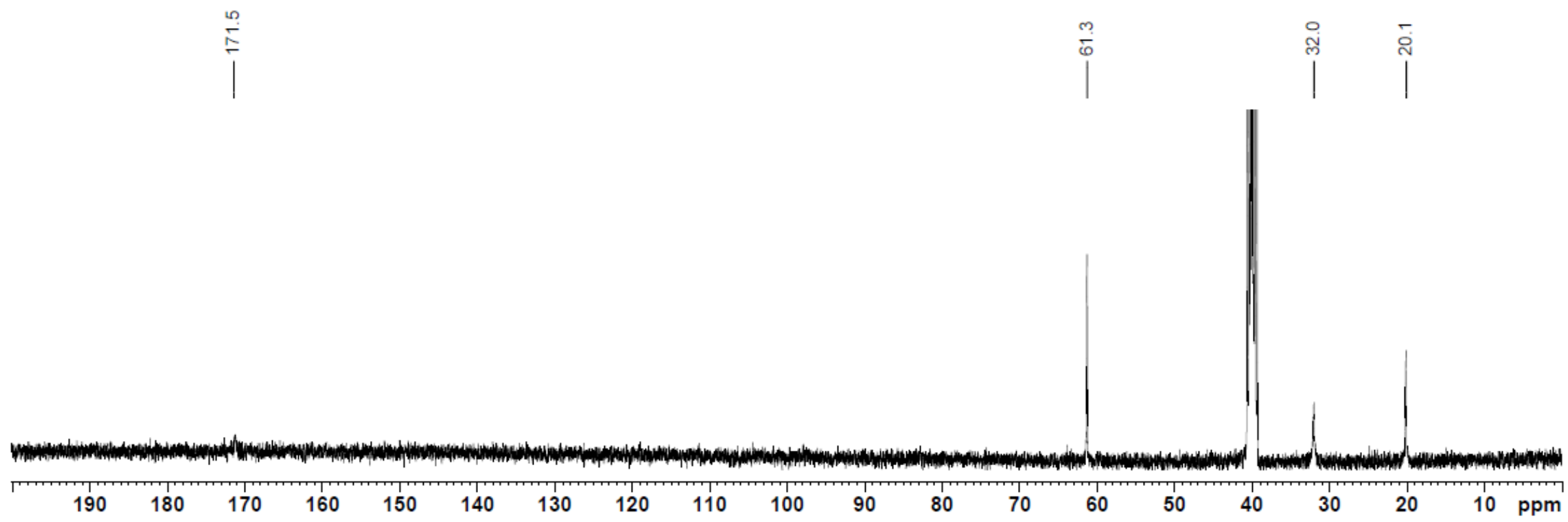

***N*-methoxy-*N*-methyl benzamide**

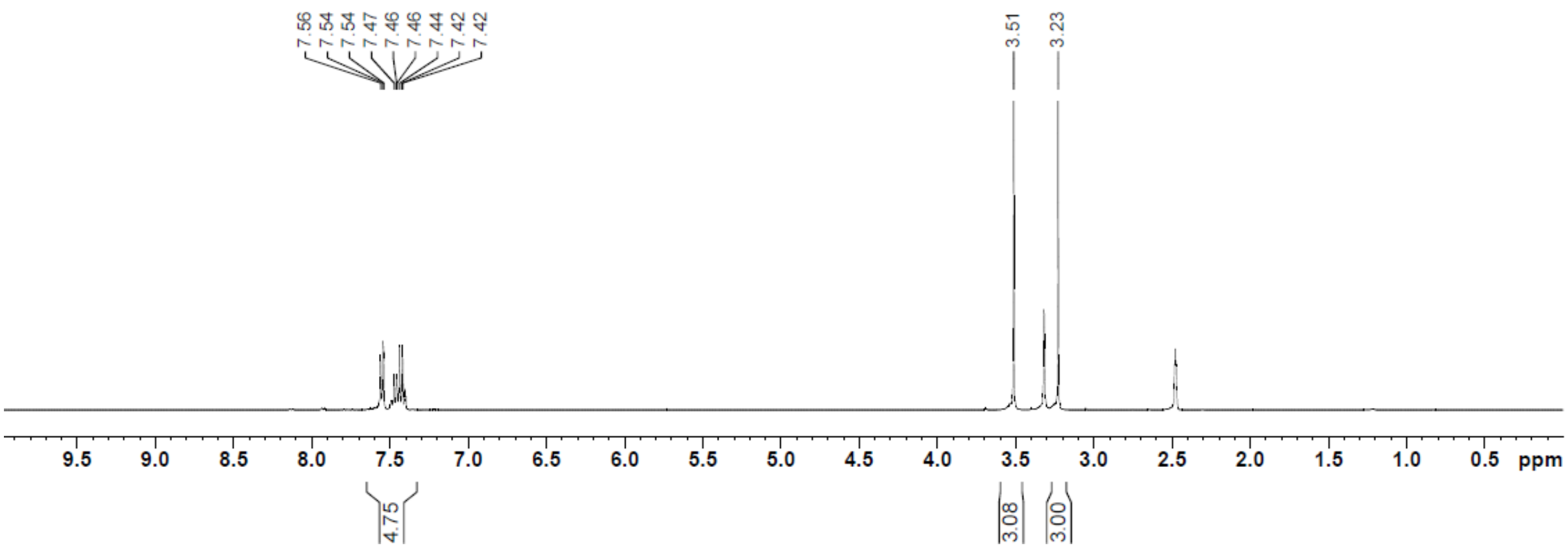

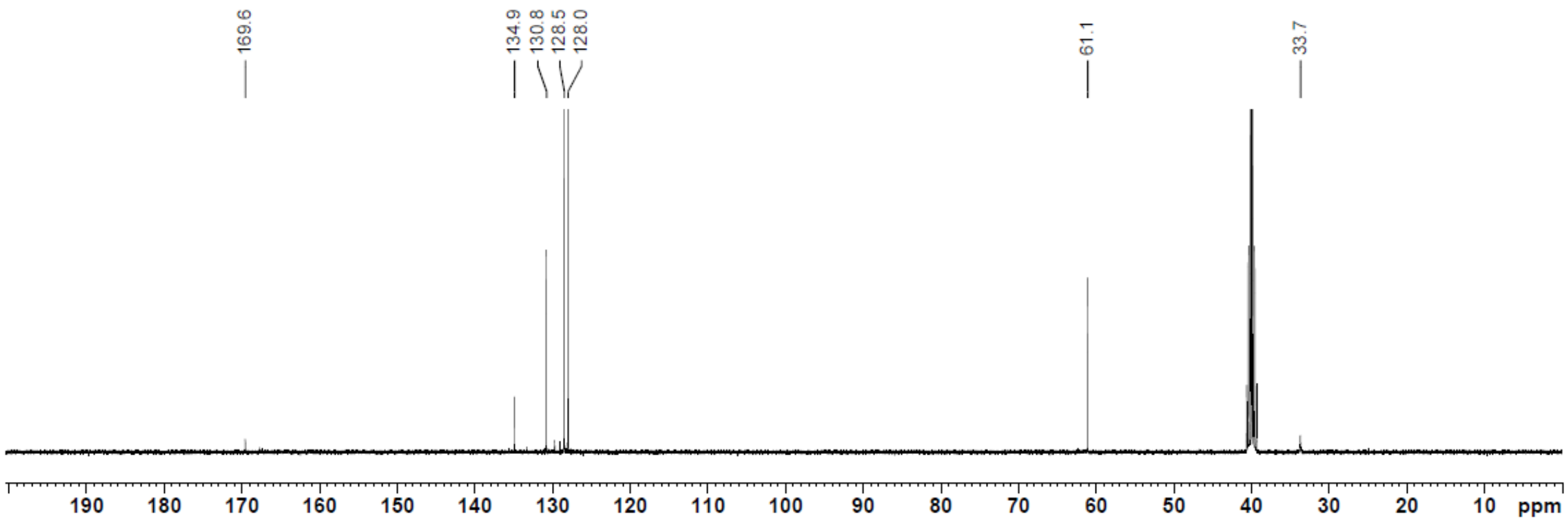

***N*-methoxy-*N*-methyl 2-phenylacetamide**

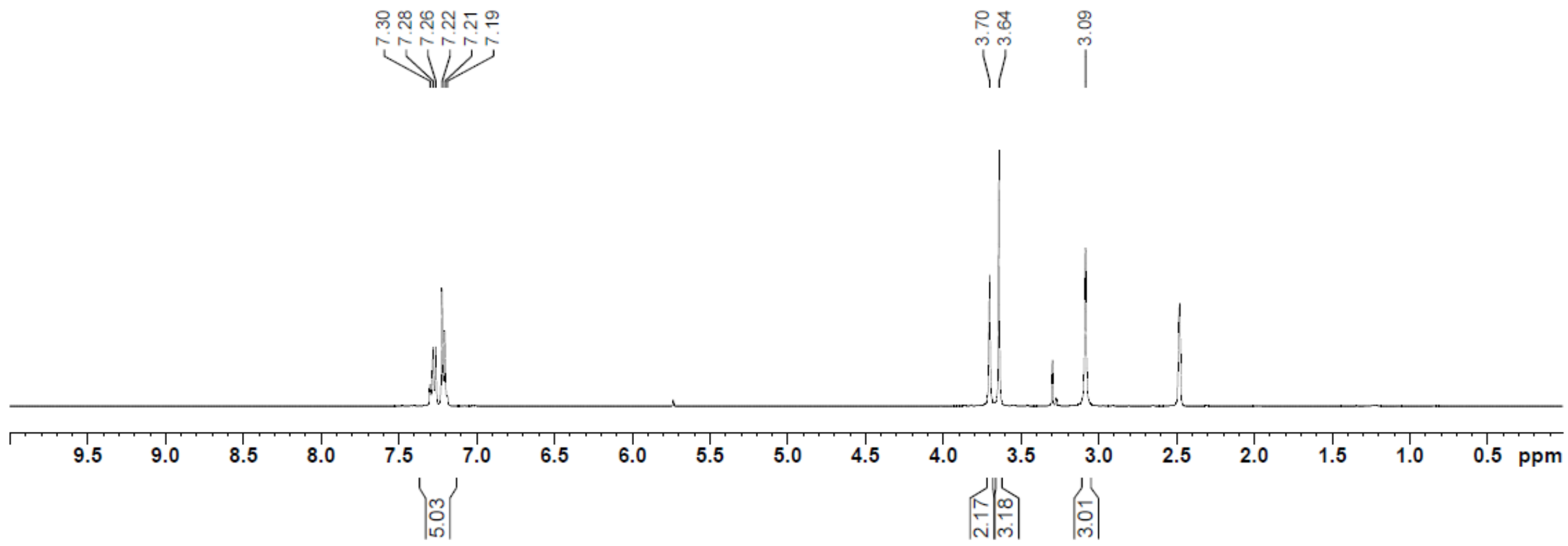

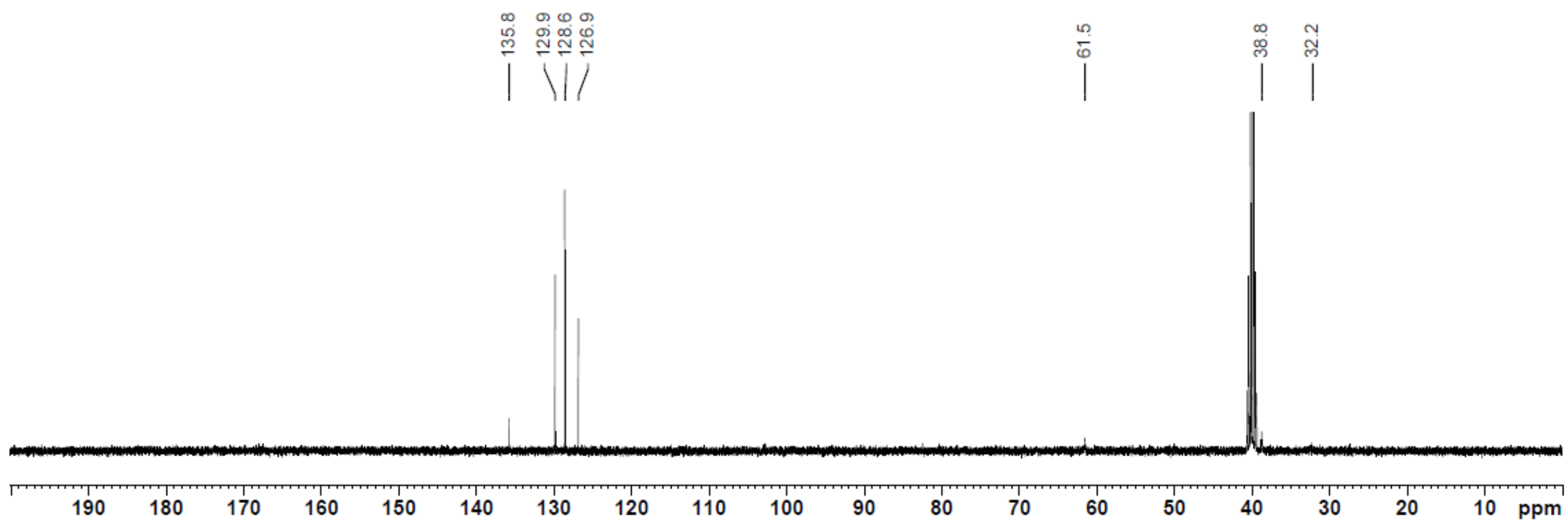

Compound **63**

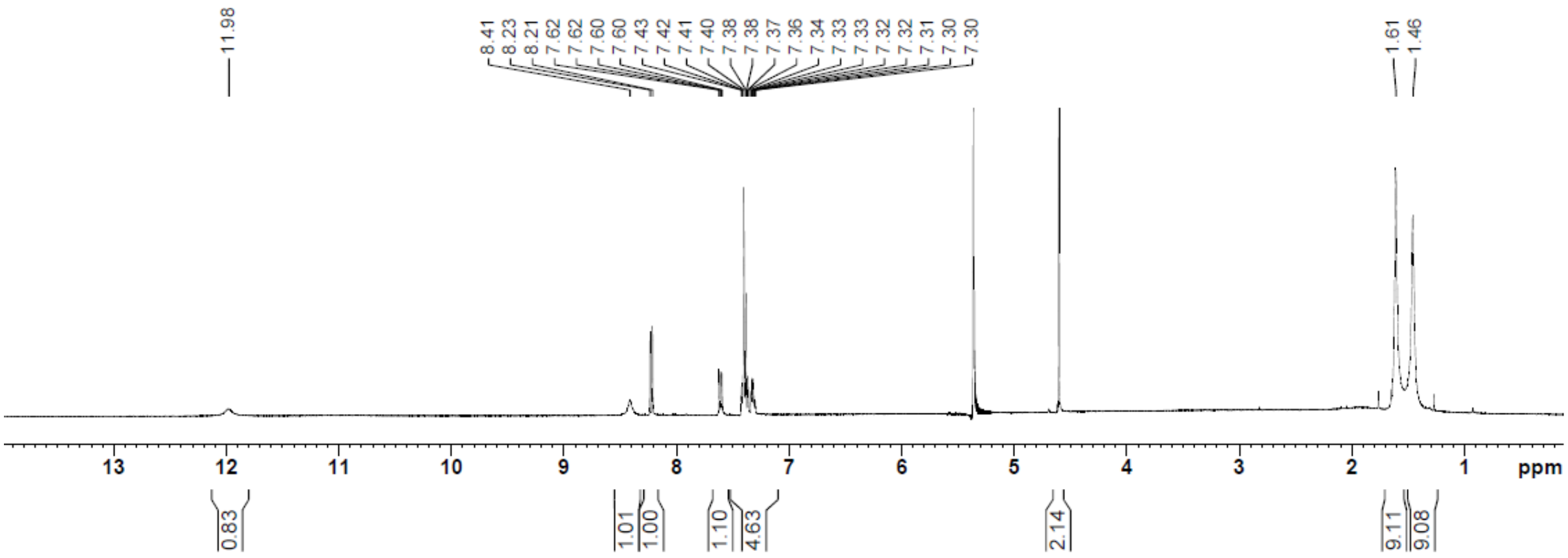

11.98
8.41
8.23
8.21
7.62
7.62
7.60
7.60
7.43
7.42
7.41
7.40
7.38
7.38
7.37
7.36
7.34
7.33
7.33
7.32
7.32
7.31
7.30
7.30
1.61
1.46
0.83
1.01
1.00
1.10
4.63
2.14
9.11
9.08
ppm

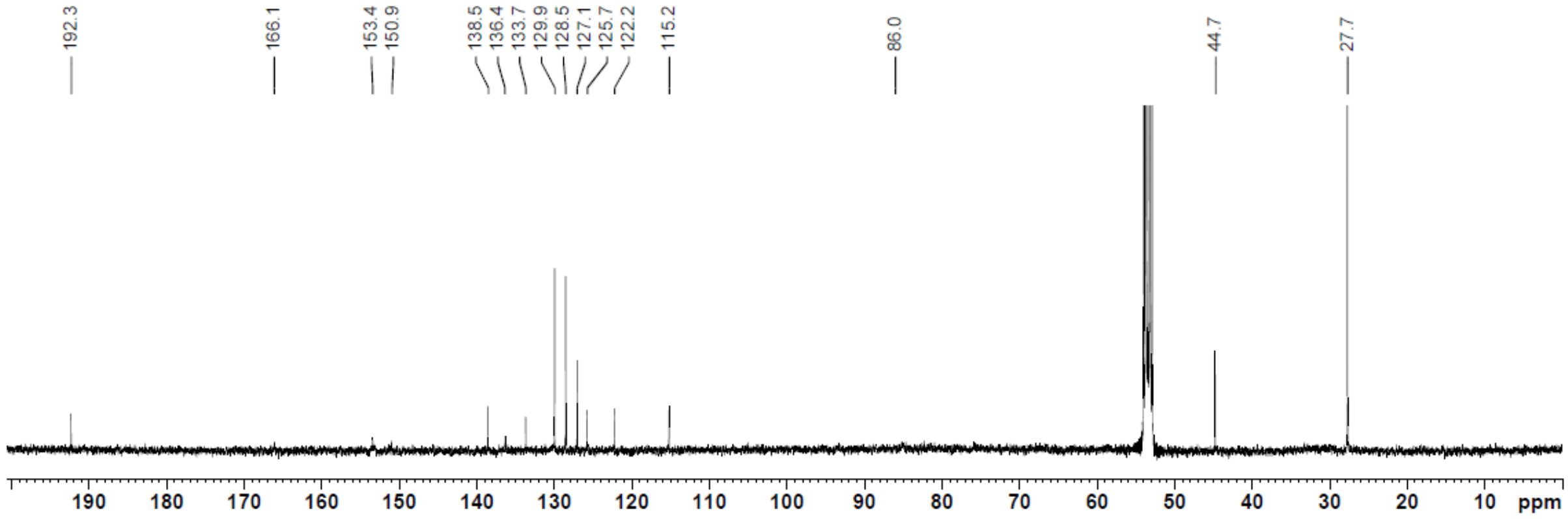

192.3
166.1
153.4
150.9
138.5
136.4
133.7
129.9
128.5
127.1
125.7
122.2
115.2
86.0
44.7
27.7
ppm

Compound **Boc-19**

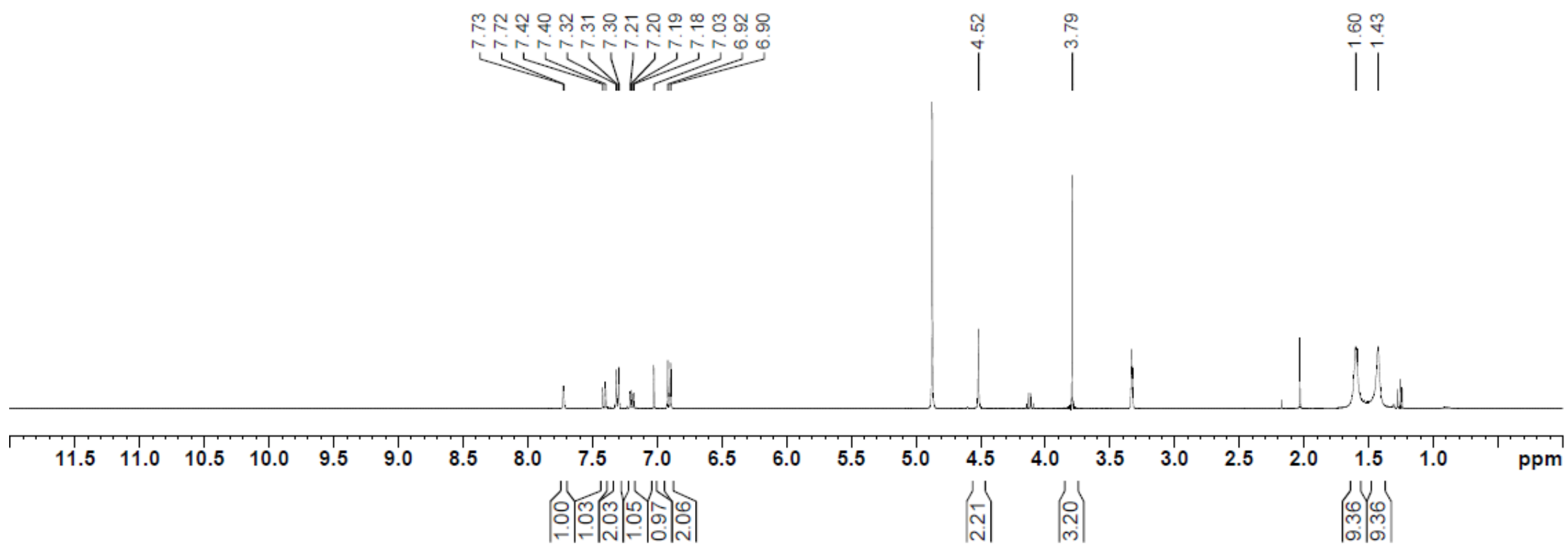

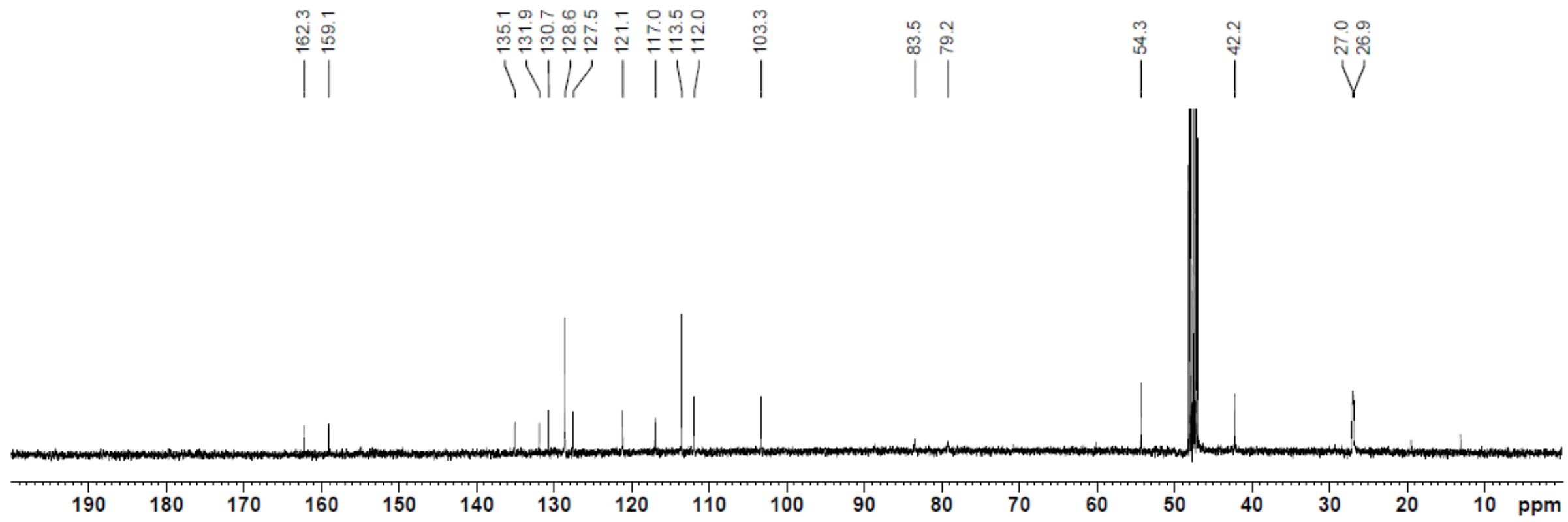

Compound **62**

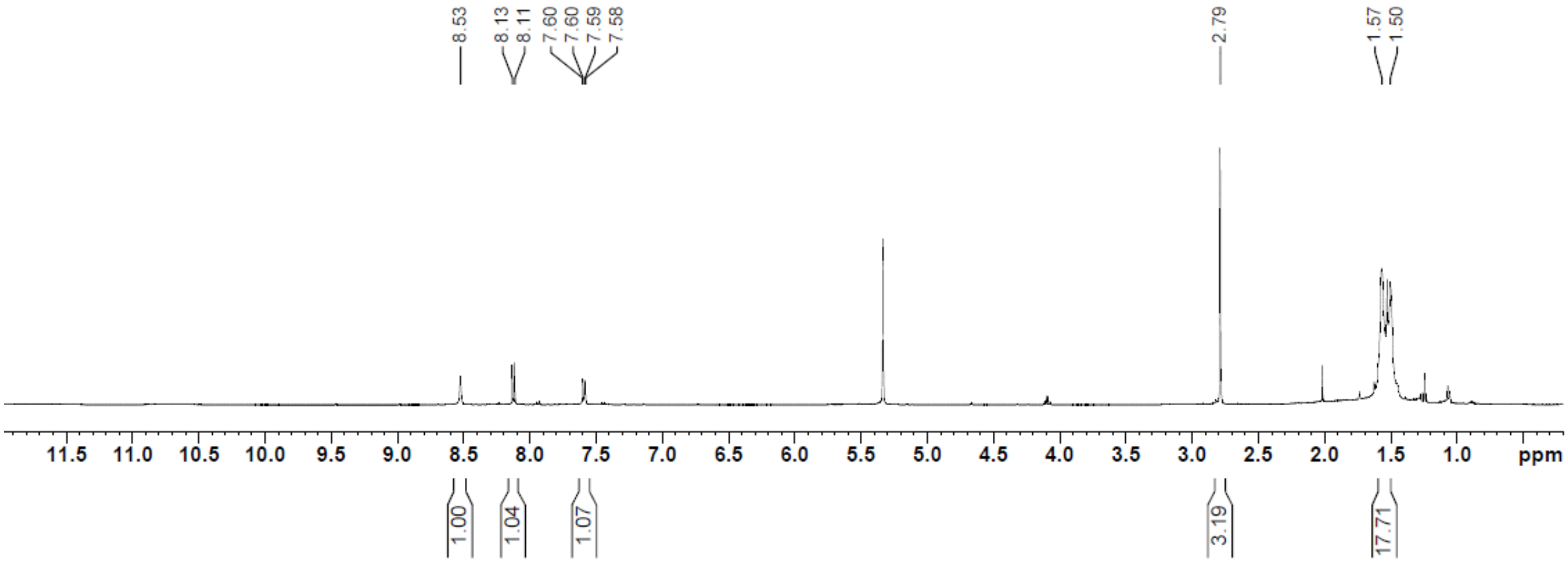
8.53
8.13
8.11
7.60
7.60
7.59
7.58
2.79
1.57
1.50
11.5 11.0 10.5 10.0 9.5 9.0 8.5 8.0 7.5 7.0 6.5 6.0 5.5 5.0 4.5 4.0 3.5 3.0 2.5 2.0 1.5 1.0 ppm
1.00
1.04
1.07
3.19
17.71

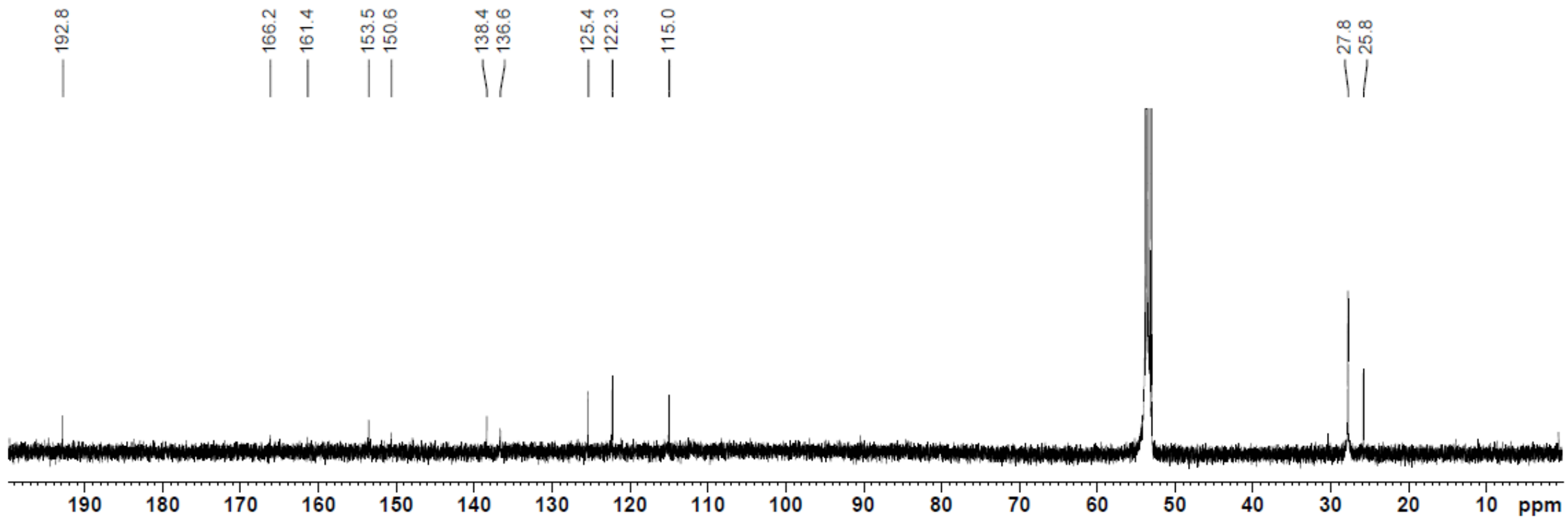
192.8
166.2
161.4
153.5
150.6
138.4
136.6
125.4
122.3
115.0
27.8
25.8
190 180 170 160 150 140 130 120 110 100 90 80 70 60 50 40 30 20 10 ppm

Compound **64**

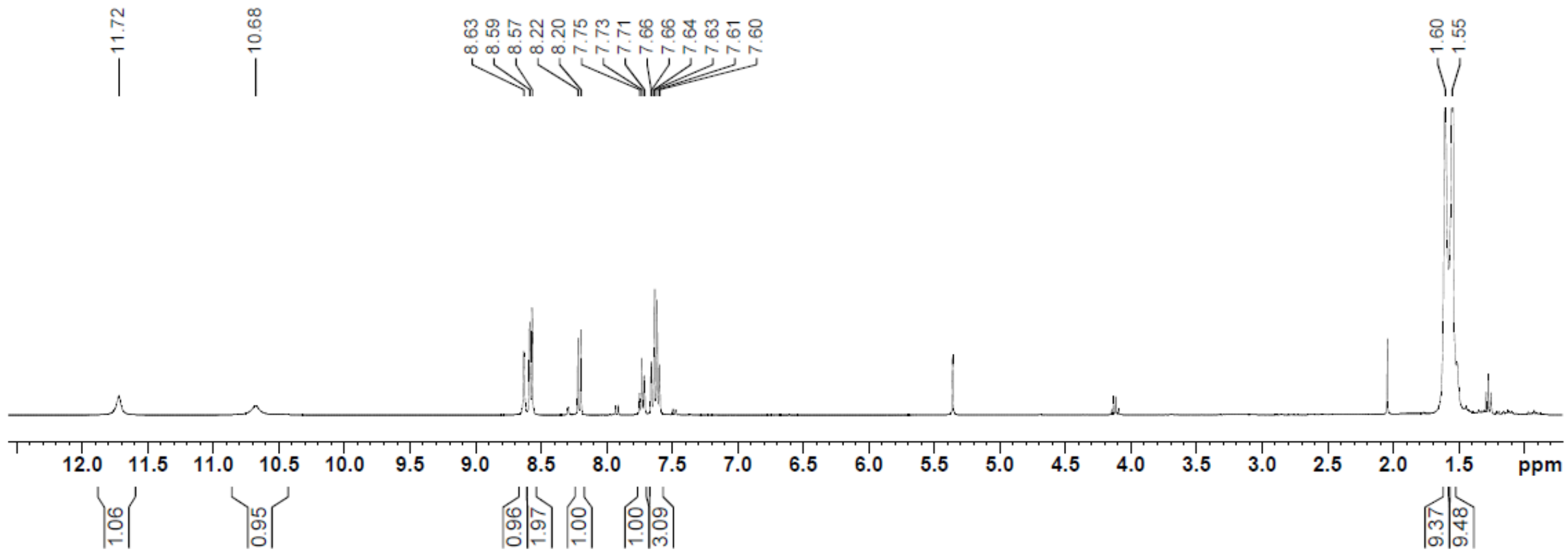

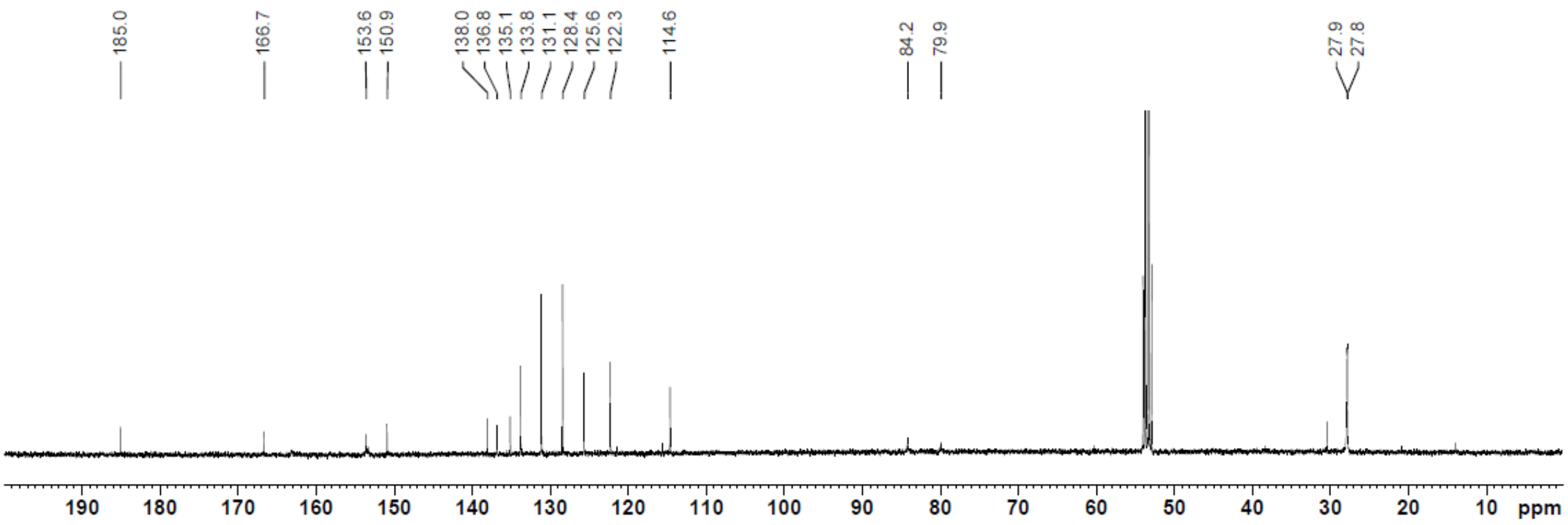

Compound **53**

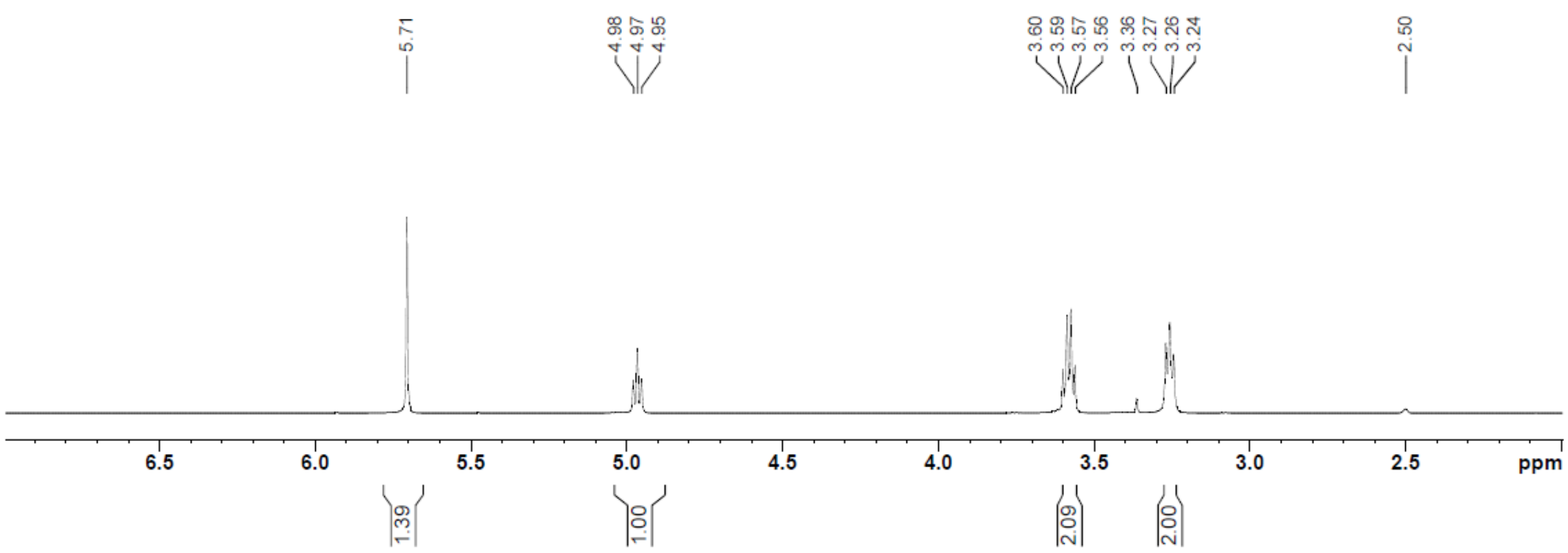

***N*-4-(2,3-bis(*tert*-butoxycarbonyl)guanidino) nitrobenzene**

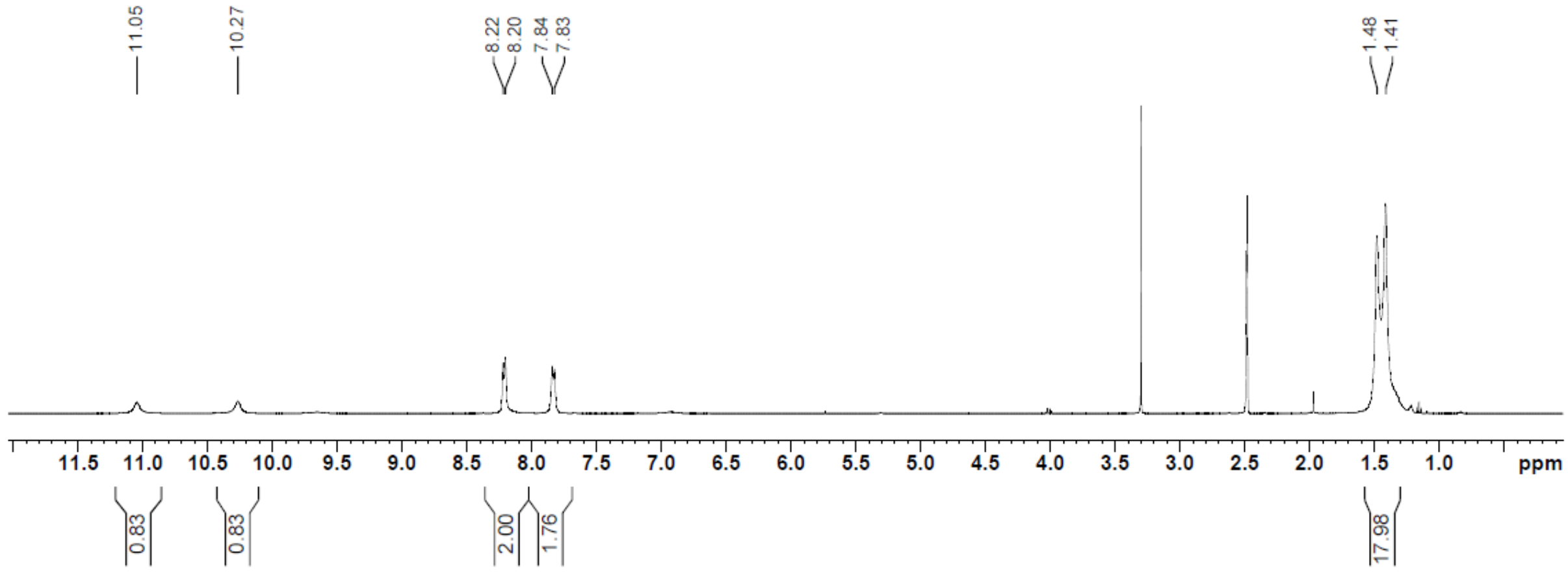

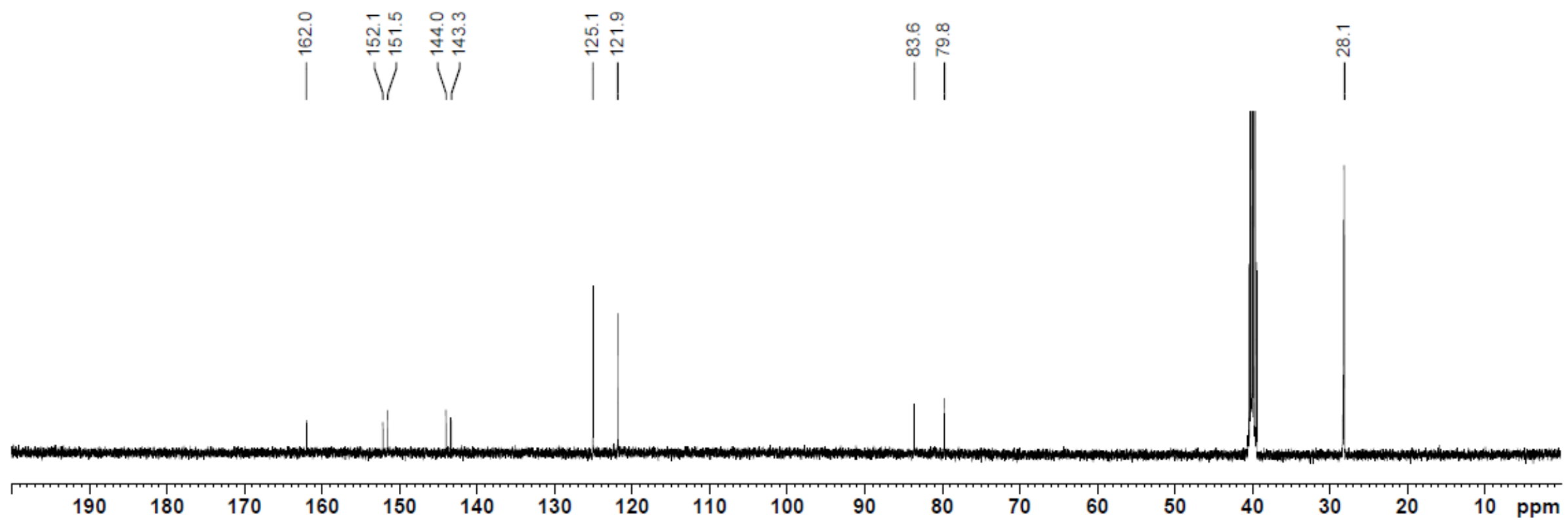

***N*[1]-4-(2,3-bis(*tert*-butoxycarbonyl)guanidino) dianiline**

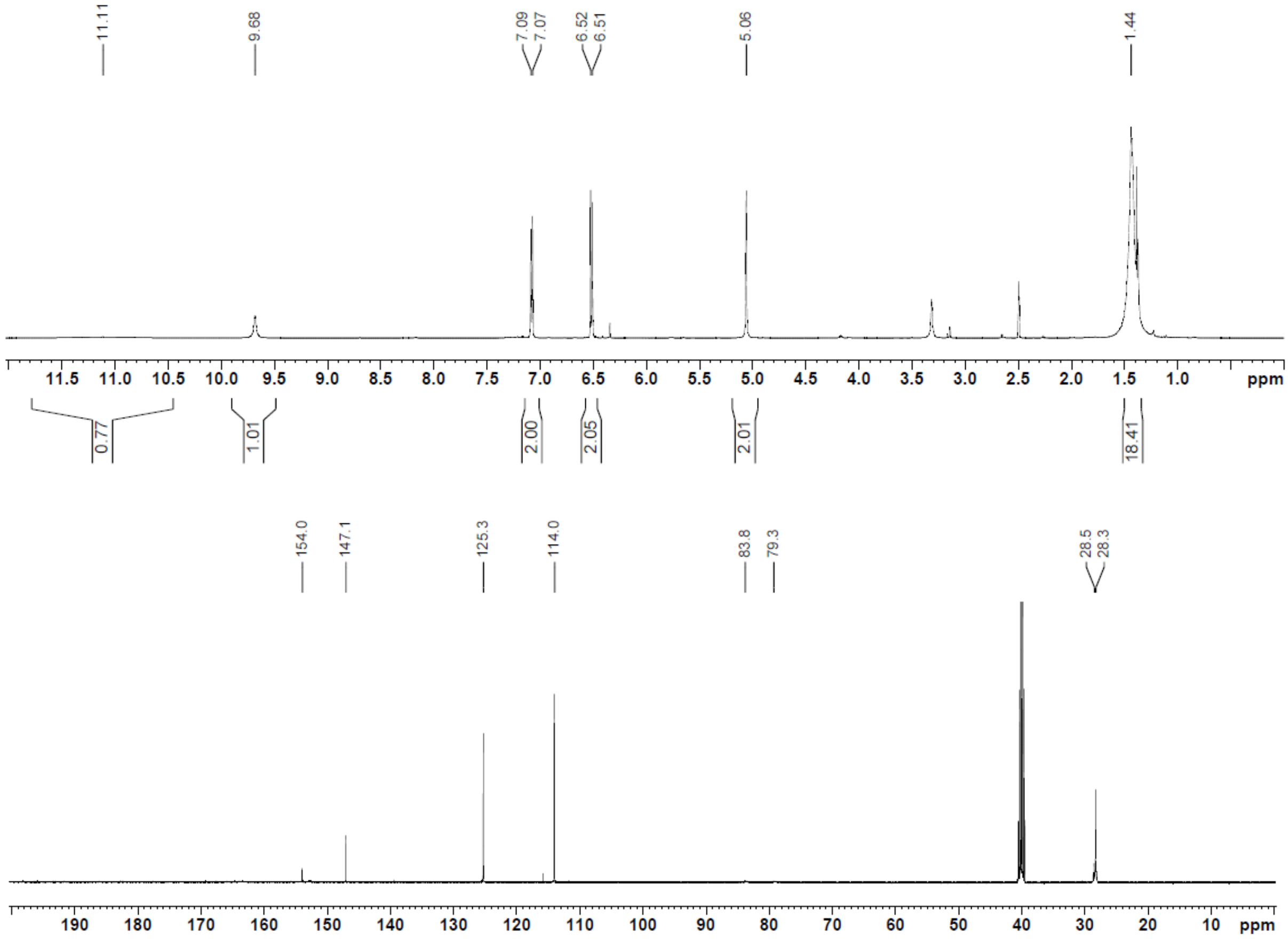

S7. SwissADME results for all compounds modelled

Both the neutral and cationic species were considered since according to SwissADME some properties such as the calculated logP are only trustable when calculated in the neutral species.

Table S2. Potential of the compounds to be inhibitors of different cytochrome P450 isoenzymes and bioavailability score calculated with SwissADME.

Cmpd.	CYP(1A2/2C19/2C9/2D6/3A4) inhibitor	Bio-availability score
**1**	No	0.55
**2**	No	0.55
**3**	No	0.55
**4**	No	0.55
**5**	No	0.55
**6**	No	0.55
**7**	No	0.55
**8**	No	0.55
**9**	No	0.55
**10**	No	0.55
**11**	No	0.55
**12**	No	0.55
**13**	No	0.55
**14**	No	0.55
**15**	No	0.55
**16**	No	0.55
**17**	No	0.55
**18**	Yes: CYP1A2/CYP2D6	0.55
**19**	Yes: CYP1A2/CYP2D6	0.55
**20**	Yes: CYP1A2/CYP2C19	0.17
**21**	No	0.55
**22**	No	0.55
**23**	No	0.55
**24**	No	0.55
**25**	No	0.55
**26**	No	0.55
**27**	No	0.55
**28**	No	0.55
**29**	No	0.55
**30**	Yes: CYP2D6	0.55
**31**	No	0.55
**32**	No	0.55
**33**	No	0.55
**34**	No	0.55
**35**	No	0.55
**36**	Yes: CYP2D6/CYP3A4	0.55
**37**	No	0.55
**38**	No	0.55
**39**	Yes: CYP1A2	0.55
**40**	Yes: CYP1A2/CYP3A4	0.55

Compound 1

Molecule 1

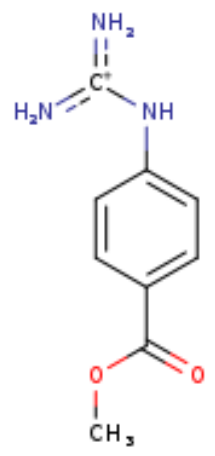

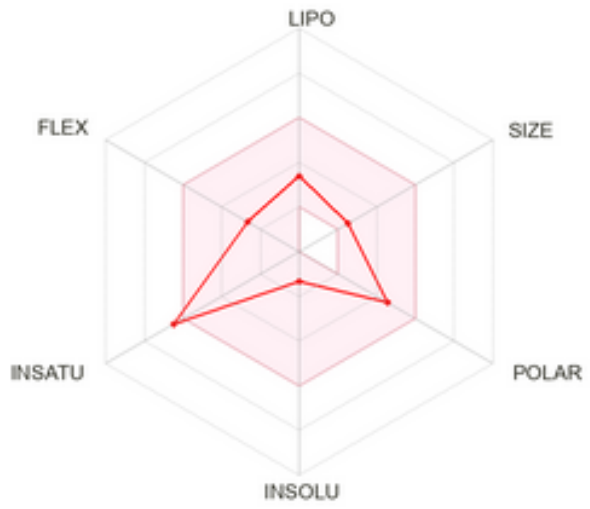

SMILES COC(=O)c1ccc(cc1)N[C+](N)N

Physicochemical Properties	
Formula	C9H12N3O2+
Molecular weight	194.21 g/mol
Num. heavy atoms	14
Num. arom. heavy atoms	6
Fraction Csp3	0.22
Num. rotatable bonds	4
Num. H-bond acceptors	4
Num. H-bond donors	3
Molar Refractivity	51.94
TPSA	90.37 Å²

Lipophilicity	
Log $P_{o/w}$ (iLOGP)	0.00
Log $P_{o/w}$ (XLOGP3)	0.39
Log $P_{o/w}$ (WLOGP)	0.06
Log $P_{o/w}$ (MLOGP)	0.77
Log $P_{o/w}$ (SILICOS-IT)	-0.46
Consensus Log $P_{o/w}$	0.15

Water Solubility	
Log *S* (ESOL)	-1.34
Solubility	8.82e+00 mg/ml ; 4.54e-02 mol/l
Class	Very soluble
Log *S* (Ali)	-1.85
Solubility	2.72e+00 mg/ml ; 1.40e-02 mol/l
Class	Very soluble
Log *S* (SILICOS-IT)	-1.86
Solubility	2.68e+00 mg/ml ; 1.38e-02 mol/l
Class	Soluble

Pharmacokinetics	
GI absorption	High
BBB permeant	No
P-gp substrate	No
CYP1A2 inhibitor	No
CYP2C19 inhibitor	No
CYP2C9 inhibitor	No
CYP2D6 inhibitor	No
CYP3A4 inhibitor	No
Log K_p (skin permeation)	-7.21 cm/s

Druglikeness	
Lipinski	Yes; 0 violation
Ghose	Yes
Veber	Yes
Egan	Yes
Muegge	No; 1 violation: MW<200
Bioavailability Score	0.55

Medicinal Chemistry	
PAINS	0 alert
Brenk	1 alert: carbo_cation_anion
Leadlikeness	No; 1 violation: MW<250
Synthetic accessibility	1.41

Molecule 1

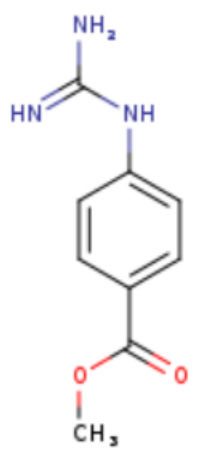

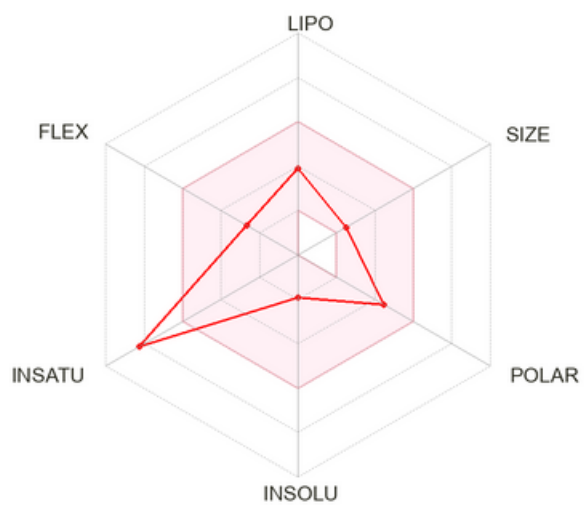

SMILES COC(=O)c1ccc(cc1)NC(=N)N

Physicochemical Properties	
Formula	C9H11N3O2
Molecular weight	193.20 g/mol
Num. heavy atoms	14
Num. arom. heavy atoms	6
Fraction Csp3	0.11
Num. rotatable bonds	4
Num. H-bond acceptors	3
Num. H-bond donors	3
Molar Refractivity	53.33
TPSA	88.20 Å²

Lipophilicity	
Log $P_{o/w}$ (iLOGP)	1.51
Log $P_{o/w}$ (XLOGP3)	1.31
Log $P_{o/w}$ (WLOGP)	0.59
Log $P_{o/w}$ (MLOGP)	1.09
Log $P_{o/w}$ (SILICOS-IT)	0.23
Consensus Log $P_{o/w}$	0.95

Water Solubility	
Log *S* (ESOL)	-1.92
Solubility	2.34e+00 mg/ml ; 1.21e-02 mol/l
Class	Very soluble
Log *S* (Ali)	-2.76
Solubility	3.34e-01 mg/ml ; 1.73e-03 mol/l
Class	Soluble
Log *S* (SILICOS-IT)	-2.26
Solubility	1.05e+00 mg/ml ; 5.46e-03 mol/l
Class	Soluble

Pharmacokinetics	
GI absorption	High
BBB permeant	No
P-gp substrate	No
CYP1A2 inhibitor	No
CYP2C19 inhibitor	No
CYP2C9 inhibitor	No
CYP2D6 inhibitor	No
CYP3A4 inhibitor	No
Log K_p (skin permeation)	-6.55 cm/s

Druglikeness	
Lipinski	Yes; 0 violation
Ghose	Yes
Veber	Yes
Egan	Yes
Muegge	No; 1 violation: MW<200
Bioavailability Score	0.55

Medicinal Chemistry	
PAINS	0 alert
Brenk	2 alerts: imine_1, imine_2
Leadlikeness	No; 1 violation: MW<250
Synthetic accessibility	1.59

Compound 2

Molecule 1

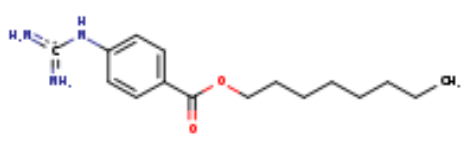

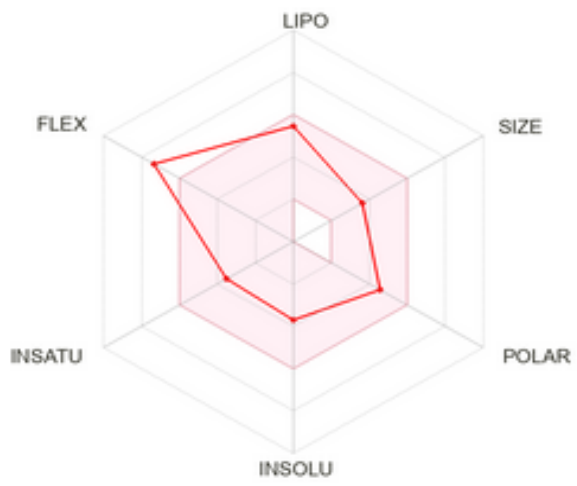

SMILES CCCCCCCCOC(=O)c1ccc(cc1)N[C+](N)N

Physicochemical Properties	
Formula	C16H26N3O2+
Molecular weight	292.40 g/mol
Num. heavy atoms	21
Num. arom. heavy atoms	6
Fraction Csp3	0.56
Num. rotatable bonds	11
Num. H-bond acceptors	4
Num. H-bond donors	3
Molar Refractivity	85.58
TPSA	90.37 Å²

Lipophilicity	
Log $P_{o/w}$ (iLOGP)	0.00
Log $P_{o/w}$ (XLOGP3)	4.06
Log $P_{o/w}$ (WLOGP)	2.79
Log $P_{o/w}$ (MLOGP)	2.69
Log $P_{o/w}$ (SILICOS-IT)	2.27
Consensus Log $P_{o/w}$	2.36

Water Solubility	
Log *S* (ESOL)	-3.70
Solubility	5.89e-02 mg/ml ; 2.01e-04 mol/l
Class	Soluble
Log *S* (Ali)	-5.66
Solubility	6.37e-04 mg/ml ; 2.18e-06 mol/l
Class	Moderately soluble
Log *S* (SILICOS-IT)	-4.68
Solubility	6.08e-03 mg/ml ; 2.08e-05 mol/l
Class	Moderately soluble

Pharmacokinetics	
GI absorption	High
BBB permeant	No
P-gp substrate	Yes
CYP1A2 inhibitor	No
CYP2C19 inhibitor	No
CYP2C9 inhibitor	No
CYP2D6 inhibitor	No
CYP3A4 inhibitor	No
Log K_p (skin permeation)	-5.20 cm/s

Druglikeness	
Lipinski	Yes; 0 violation
Ghose	Yes
Veber	No; 1 violation: Rotors>10
Egan	Yes
Muegge	Yes
Bioavailability Score	0.55

Medicinal Chemistry	
PAINS	0 alert
Brenk	1 alert: carbo_cation_anion
Leadlikeness	No; 2 violations: Rotors>7, XLOGP3>3.5
Synthetic accessibility	2.44

Molecule 1

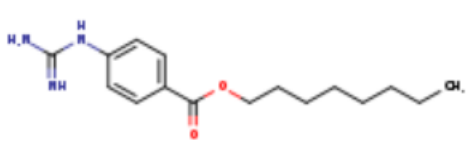

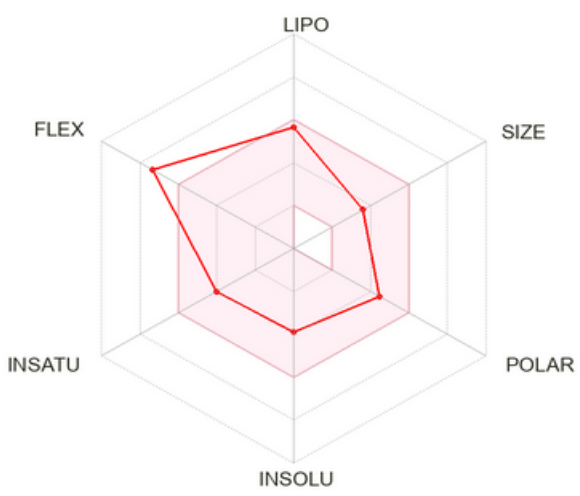

SMILES CCCCCCCCOC(=O)c1ccc(cc1)NC(=N)N

Physicochemical Properties	
Formula	C16H25N3O2
Molecular weight	291.39 g/mol
Num. heavy atoms	21
Num. arom. heavy atoms	6
Fraction Csp3	0.50
Num. rotatable bonds	11
Num. H-bond acceptors	3
Num. H-bond donors	3
Molar Refractivity	86.97
TPSA	88.20 Å²

Lipophilicity	
Log $P_{o/w}$ (iLOGP)	2.89
Log $P_{o/w}$ (XLOGP3)	4.39
Log $P_{o/w}$ (WLOGP)	3.32
Log $P_{o/w}$ (MLOGP)	3.01
Log $P_{o/w}$ (SILICOS-IT)	2.96
Consensus Log $P_{o/w}$	3.31

Water Solubility	
Log *S* (ESOL)	-3.90
Solubility	3.69e-02 mg/ml ; 1.27e-04 mol/l
Class	Soluble
Log *S* (Ali)	-5.96
Solubility	3.20e-04 mg/ml ; 1.10e-06 mol/l
Class	Moderately soluble
Log *S* (SILICOS-IT)	-5.09
Solubility	2.39e-03 mg/ml ; 8.20e-06 mol/l
Class	Moderately soluble

Pharmacokinetics	
GI absorption	High
BBB permeant	No
P-gp substrate	No
CYP1A2 inhibitor	No
CYP2C19 inhibitor	Yes
CYP2C9 inhibitor	Yes
CYP2D6 inhibitor	No
CYP3A4 inhibitor	No
Log K_p (skin permeation)	-4.96 cm/s

Druglikeness	
Lipinski	Yes; 0 violation
Ghose	Yes
Veber	No; 1 violation: Rotors>10
Egan	Yes
Muegge	Yes
Bioavailability Score	0.55

Medicinal Chemistry	
PAINS	0 alert
Brenk	2 alerts: imine_1, imine_2
Leadlikeness	No; 2 violations: Rotors>7, XLOGP3>3.5
Synthetic accessibility	2.54

Compound 3

Molecule 1

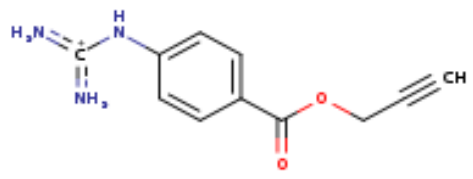

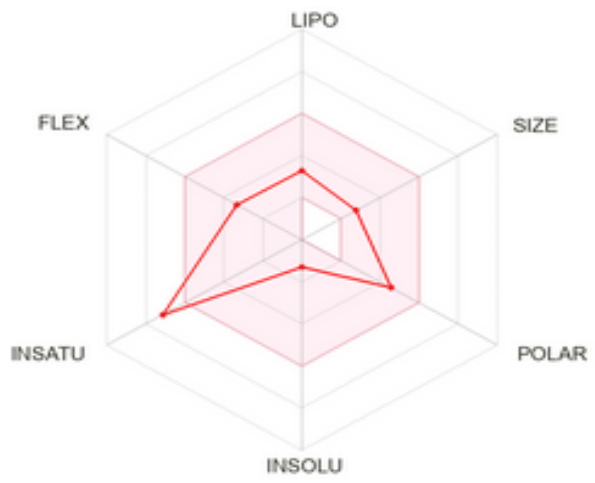

SMILES C#CCOC(=O)c1ccc(cc1)N[C+](N)N

Physicochemical Properties	
Formula	C11H12N3O2+
Molecular weight	218.23 g/mol
Num. heavy atoms	16
Num. arom. heavy atoms	6
Fraction Csp3	0.18
Num. rotatable bonds	5
Num. H-bond acceptors	4
Num. H-bond donors	3
Molar Refractivity	59.71
TPSA	90.37 Å²

Lipophilicity	
Log $P_{o/w}$ (iLOGP)	0.00
Log $P_{o/w}$ (XLOGP3)	0.25
Log $P_{o/w}$ (WLOGP)	0.14
Log $P_{o/w}$ (MLOGP)	1.28
Log $P_{o/w}$ (SILICOS-IT)	0.11
Consensus Log $P_{o/w}$	0.36

Water Solubility	
Log *S* (ESOL)	-1.30
Solubility	1.10e+01 mg/ml ; 5.03e-02 mol/l
Class	Very soluble
Log *S* (Ali)	-1.71
Solubility	4.27e+00 mg/ml ; 1.96e-02 mol/l
Class	Very soluble
Log *S* (SILICOS-IT)	-1.96
Solubility	2.37e+00 mg/ml ; 1.09e-02 mol/l
Class	Soluble

Pharmacokinetics	
GI absorption	High
BBB permeant	No
P-gp substrate	No
CYP1A2 inhibitor	No
CYP2C19 inhibitor	No
CYP2C9 inhibitor	No
CYP2D6 inhibitor	No
CYP3A4 inhibitor	No
Log K_p (skin permeation)	-7.45 cm/s

Druglikeness	
Lipinski	Yes; 0 violation
Ghose	Yes
Veber	Yes
Egan	Yes
Muegge	Yes
Bioavailability Score	0.55

Medicinal Chemistry	
PAINS	0 alert
Brenk	2 alerts: carbo_cation_anion, triple_bond
Leadlikeness	No; 1 violation: MW<250
Synthetic accessibility	2.04

Molecule 1

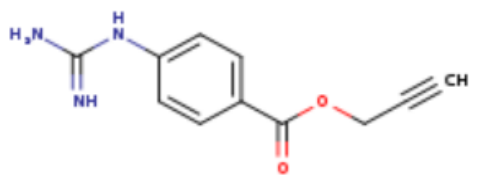

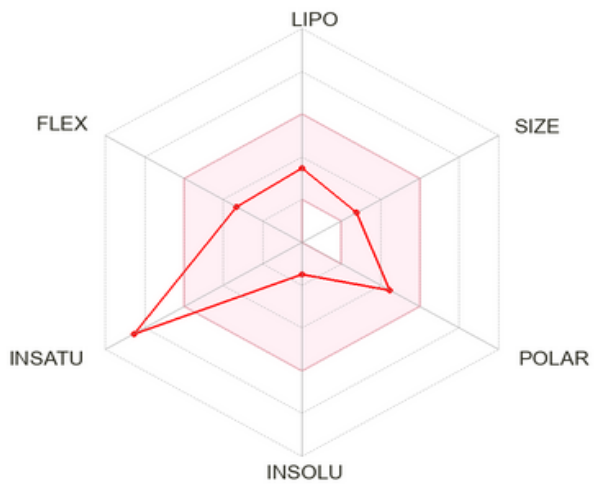

SMILES C#CCOC(=O)c1ccc(cc1)NC(=N)N

Physicochemical Properties	
Formula	C11H11N3O2
Molecular weight	217.22 g/mol
Num. heavy atoms	16
Num. arom. heavy atoms	6
Fraction Csp3	0.09
Num. rotatable bonds	5
Num. H-bond acceptors	3
Num. H-bond donors	3
Molar Refractivity	61.10
TPSA	88.20 Å²

Lipophilicity	
Log $P_{o/w}$ (iLOGP)	1.59
Log $P_{o/w}$ (XLOGP3)	0.58
Log $P_{o/w}$ (WLOGP)	0.67
Log $P_{o/w}$ (MLOGP)	1.60
Log $P_{o/w}$ (SILICOS-IT)	0.80
Consensus Log $P_{o/w}$	1.05

Water Solubility	
Log *S* (ESOL)	-1.50
Solubility	6.87e+00 mg/ml ; 3.16e-02 mol/l
Class	Very soluble
Log *S* (Ali)	-2.01
Solubility	2.15e+00 mg/ml ; 9.88e-03 mol/l
Class	Soluble
Log *S* (SILICOS-IT)	-2.37
Solubility	9.33e-01 mg/ml ; 4.30e-03 mol/l
Class	Soluble

Pharmacokinetics	
GI absorption	High
BBB permeant	No
P-gp substrate	No
CYP1A2 inhibitor	No
CYP2C19 inhibitor	No
CYP2C9 inhibitor	No
CYP2D6 inhibitor	No
CYP3A4 inhibitor	No
Log K_p (skin permeation)	-7.21 cm/s

Druglikeness	
Lipinski	Yes; 0 violation
Ghose	Yes
Veber	Yes
Egan	Yes
Muegge	Yes
Bioavailability Score	0.55

Medicinal Chemistry	
PAINS	0 alert
Brenk	3 alerts: imine_1, imine_2, triple_bond
Leadlikeness	No; 1 violation: MW<250
Synthetic accessibility	2.14

Compound 4

Molecule 1

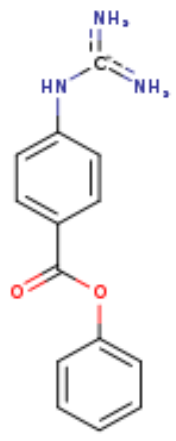

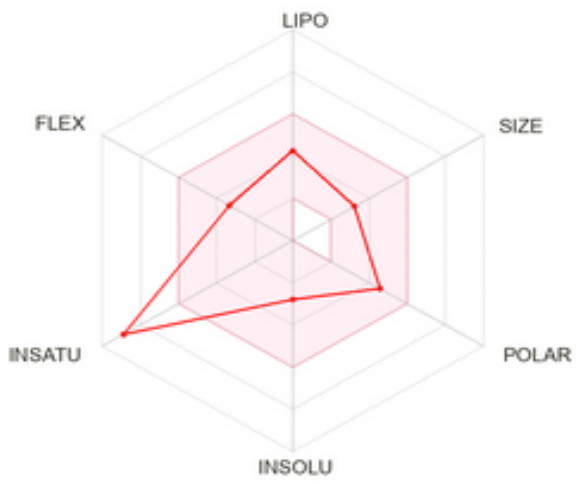

SMILES N[C+](Nc1ccc(cc1)C(=O)Oc1ccccc1)N

Physicochemical Properties	
Formula	C14H14N3O2+
Molecular weight	256.28 g/mol
Num. heavy atoms	19
Num. arom. heavy atoms	12
Fraction Csp3	0.07
Num. rotatable bonds	5
Num. H-bond acceptors	4
Num. H-bond donors	3
Molar Refractivity	72.06
TPSA	90.37 Å²

Lipophilicity	
Log $P_{o/w}$ (iLOGP)	0.00
Log $P_{o/w}$ (XLOGP3)	1.95
Log $P_{o/w}$ (WLOGP)	1.49
Log $P_{o/w}$ (MLOGP)	2.20
Log $P_{o/w}$ (SILICOS-IT)	0.74
Consensus Log $P_{o/w}$	1.28

Water Solubility	
Log *S* (ESOL)	-2.79
Solubility	4.11e-01 mg/ml ; 1.60e-03 mol/l
Class	Soluble
Log *S* (Ali)	-3.47
Solubility	8.63e-02 mg/ml ; 3.37e-04 mol/l
Class	Soluble
Log *S* (SILICOS-IT)	-3.99
Solubility	2.62e-02 mg/ml ; 1.02e-04 mol/l
Class	Soluble

Pharmacokinetics	
GI absorption	High
BBB permeant	No
P-gp substrate	No
CYP1A2 inhibitor	No
CYP2C19 inhibitor	No
CYP2C9 inhibitor	No
CYP2D6 inhibitor	No
CYP3A4 inhibitor	No
Log K_p (skin permeation)	-6.48 cm/s

Druglikeness	
Lipinski	Yes; 0 violation
Ghose	Yes
Veber	Yes
Egan	Yes
Muegge	Yes
Bioavailability Score	0.55

Medicinal Chemistry	
PAINS	0 alert
Brenk	2 alerts: carbo_cation_anion, phenol_ester
Leadlikeness	Yes
Synthetic accessibility	1.90

Molecule 1

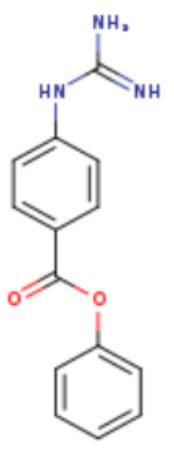

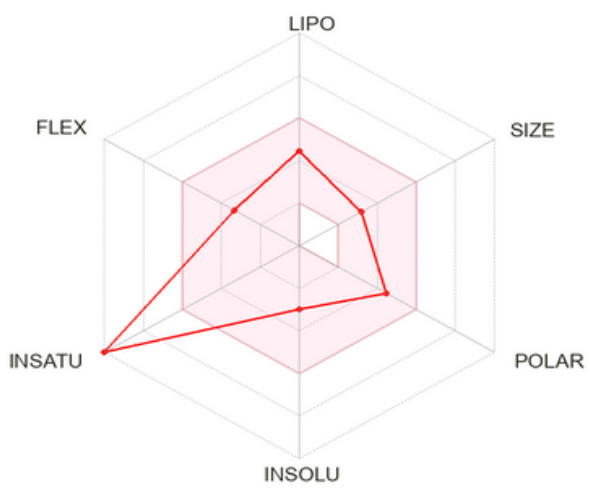

SMILES NC(=N)Nc1ccc(cc1)C(=O)Oc1ccccc1

Physicochemical Properties	
Formula	C14H13N3O2
Molecular weight	255.27 g/mol
Num. heavy atoms	19
Num. arom. heavy atoms	12
Fraction Csp3	0.00
Num. rotatable bonds	5
Num. H-bond acceptors	3
Num. H-bond donors	3
Molar Refractivity	73.45
TPSA	88.20 Å²

Lipophilicity	
Log $P_{o/w}$ (iLOGP)	1.64
Log $P_{o/w}$ (XLOGP3)	2.28
Log $P_{o/w}$ (WLOGP)	2.02
Log $P_{o/w}$ (MLOGP)	2.53
Log $P_{o/w}$ (SILICOS-IT)	1.43
Consensus Log $P_{o/w}$	1.98

Water Solubility	
Log *S* (ESOL)	-3.00
Solubility	2.57e-01 mg/ml ; 1.01e-03 mol/l
Class	Soluble
Log *S* (Ali)	-3.77
Solubility	4.34e-02 mg/ml ; 1.70e-04 mol/l
Class	Soluble
Log *S* (SILICOS-IT)	-4.39
Solubility	1.03e-02 mg/ml ; 4.04e-05 mol/l
Class	Moderately soluble

Pharmacokinetics	
GI absorption	High
BBB permeant	No
P-gp substrate	No
CYP1A2 inhibitor	No
CYP2C19 inhibitor	No
CYP2C9 inhibitor	No
CYP2D6 inhibitor	No
CYP3A4 inhibitor	No
Log K_p (skin permeation)	-6.24 cm/s

Druglikeness	
Lipinski	Yes; 0 violation
Ghose	Yes
Veber	Yes
Egan	Yes
Muegge	Yes
Bioavailability Score	0.55

Medicinal Chemistry	
PAINS	0 alert
Brenk	3 alerts: imine_1, imine_2, phenol_ester
Leadlikeness	Yes
Synthetic accessibility	2.01

Compound 5

Molecule 1

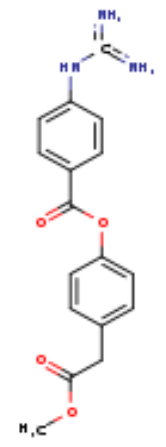

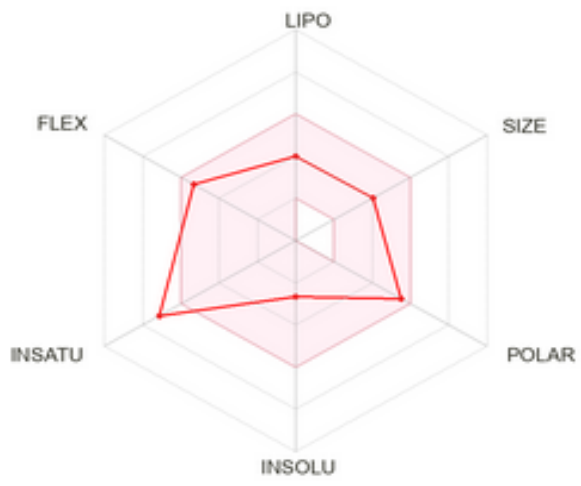

SMILES COC(=O)Cc1ccc(cc1)OC(=O)c1ccc(cc1)N[C+](N)N

Physicochemical Properties	
Formula	C17H18N3O4+
Molecular weight	328.34 g/mol
Num. heavy atoms	24
Num. arom. heavy atoms	12
Fraction Csp3	0.18
Num. rotatable bonds	8
Num. H-bond acceptors	6
Num. H-bond donors	3
Molar Refractivity	87.92
TPSA	116.67 Å²

Lipophilicity	
Log $P_{o/w}$ (iLOGP)	0.00
Log $P_{o/w}$ (XLOGP3)	1.48
Log $P_{o/w}$ (WLOGP)	1.21
Log $P_{o/w}$ (MLOGP)	2.07
Log $P_{o/w}$ (SILICOS-IT)	1.12
Consensus Log $P_{o/w}$	1.18

Water Solubility	
Log *S* (ESOL)	-2.65
Solubility	7.35e-01 mg/ml ; 2.24e-03 mol/l
Class	Soluble
Log *S* (Ali)	-3.54
Solubility	9.53e-02 mg/ml ; 2.90e-04 mol/l
Class	Soluble
Log *S* (SILICOS-IT)	-4.44
Solubility	1.19e-02 mg/ml ; 3.62e-05 mol/l
Class	Moderately soluble

Pharmacokinetics	
GI absorption	High
BBB permeant	No
P-gp substrate	No
CYP1A2 inhibitor	No
CYP2C19 inhibitor	No
CYP2C9 inhibitor	No
CYP2D6 inhibitor	No
CYP3A4 inhibitor	No
Log K_p (skin permeation)	-7.25 cm/s

Druglikeness	
Lipinski	Yes; 0 violation
Ghose	Yes
Veber	Yes
Egan	Yes
Muegge	Yes
Bioavailability Score	0.55

Medicinal Chemistry	
PAINS	0 alert
Brenk	2 alerts: carbo_cation_anion, phenol_ester
Leadlikeness	No; 1 violation: Rotors>7
Synthetic accessibility	2.37

Molecule 1

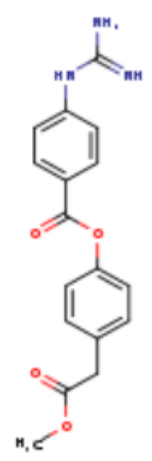

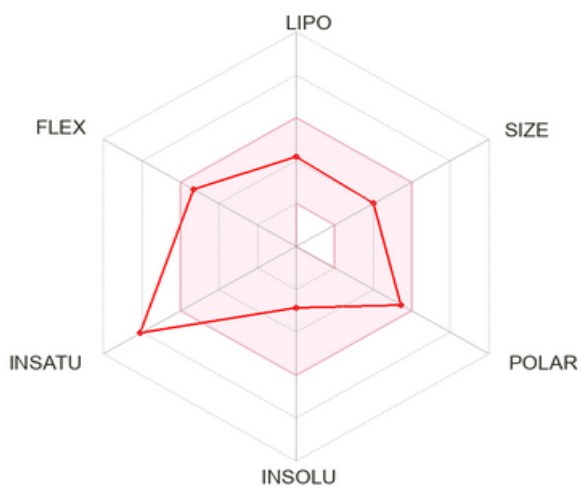

SMILES COC(=O)Cc1ccc(cc1)OC(=O)c1ccc(cc1)NC(=N)N

Physicochemical Properties	
Formula	C17H17N3O4
Molecular weight	327.33 g/mol
Num. heavy atoms	24
Num. arom. heavy atoms	12
Fraction Csp3	0.12
Num. rotatable bonds	8
Num. H-bond acceptors	5
Num. H-bond donors	3
Molar Refractivity	89.31
TPSA	114.50 Å²

Lipophilicity	
Log $P_{o/w}$ (iLOGP)	2.15
Log $P_{o/w}$ (XLOGP3)	1.81
Log $P_{o/w}$ (WLOGP)	1.74
Log $P_{o/w}$ (MLOGP)	2.40
Log $P_{o/w}$ (SILICOS-IT)	1.81
Consensus Log $P_{o/w}$	1.98

Water Solubility	
Log *S* (ESOL)	-2.85
Solubility	4.60e-01 mg/ml ; 1.41e-03 mol/l
Class	Soluble
Log *S* (Ali)	-3.83
Solubility	4.80e-02 mg/ml ; 1.47e-04 mol/l
Class	Soluble
Log *S* (SILICOS-IT)	-4.85
Solubility	4.67e-03 mg/ml ; 1.43e-05 mol/l
Class	Moderately soluble

Pharmacokinetics	
GI absorption	High
BBB permeant	No
P-gp substrate	No
CYP1A2 inhibitor	No
CYP2C19 inhibitor	No
CYP2C9 inhibitor	No
CYP2D6 inhibitor	No
CYP3A4 inhibitor	No
Log K_p (skin permeation)	-7.01 cm/s

Druglikeness	
Lipinski	Yes; 0 violation
Ghose	Yes
Veber	Yes
Egan	Yes
Muegge	Yes
Bioavailability Score	0.55

Medicinal Chemistry	
PAINS	0 alert
Brenk	3 alerts: imine_1, imine_2, phenol_ester
Leadlikeness	No; 1 violation: Rotors>7
Synthetic accessibility	2.44

Compound 6

Molecule 1

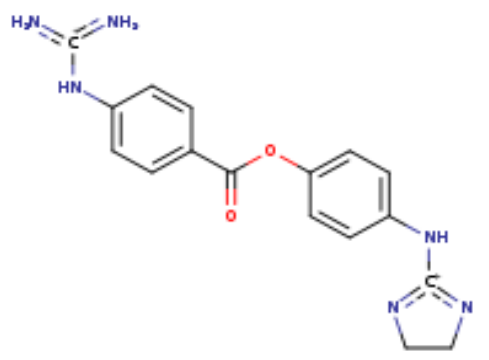

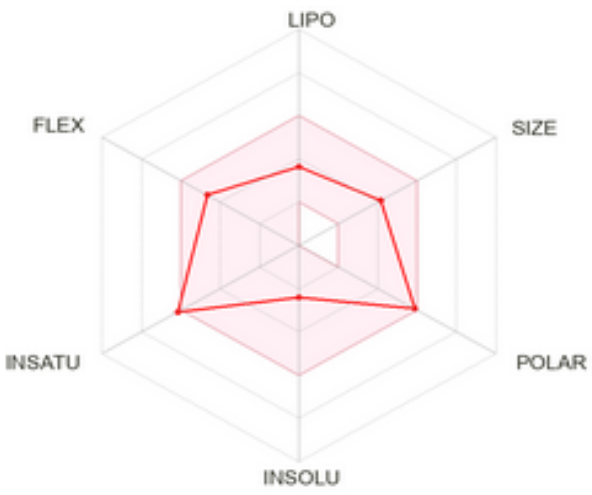

SMILES N[C+](Nc1ccc(cc1)C(=O)Oc1ccc(cc1)N[C+]1NCCN1)N

Physicochemical Properties	
Formula	C17H20N6O2++
Molecular weight	340.38 g/mol
Num. heavy atoms	25
Num. arom. heavy atoms	12
Fraction Csp3	0.24
Num. rotatable bonds	7
Num. H-bond acceptors	6
Num. H-bond donors	6
Molar Refractivity	101.79
TPSA	126.46 Å²

Lipophilicity	
Log $P_{o/w}$ (iLOGP)	0.00
Log $P_{o/w}$ (XLOGP3)	0.86
Log $P_{o/w}$ (WLOGP)	-0.41
Log $P_{o/w}$ (MLOGP)	1.24
Log $P_{o/w}$ (SILICOS-IT)	-0.47
Consensus Log $P_{o/w}$	0.24

Water Solubility	
Log *S* (ESOL)	-2.39
Solubility	1.40e+00 mg/ml ; 4.12e-03 mol/l
Class	Soluble
Log *S* (Ali)	-3.10
Solubility	2.71e-01 mg/ml ; 7.96e-04 mol/l
Class	Soluble
Log *S* (SILICOS-IT)	-4.71
Solubility	6.70e-03 mg/ml ; 1.97e-05 mol/l
Class	Moderately soluble

Pharmacokinetics	
GI absorption	High
BBB permeant	No
P-gp substrate	Yes
CYP1A2 inhibitor	No
CYP2C19 inhibitor	No
CYP2C9 inhibitor	No
CYP2D6 inhibitor	No
CYP3A4 inhibitor	No
Log K_p (skin permeation)	-7.77 cm/s

Druglikeness	
Lipinski	Yes; 1 violation: NHorOH>5
Ghose	No; 1 violation: WLOGP<-0.4
Veber	Yes
Egan	Yes
Muegge	No; 1 violation: H-don>5
Bioavailability Score	0.55

Medicinal Chemistry	
PAINS	0 alert
Brenk	2 alerts: carbo_cation_anion, phenol_ester
Leadlikeness	Yes
Synthetic accessibility	2.54

Molecule 1

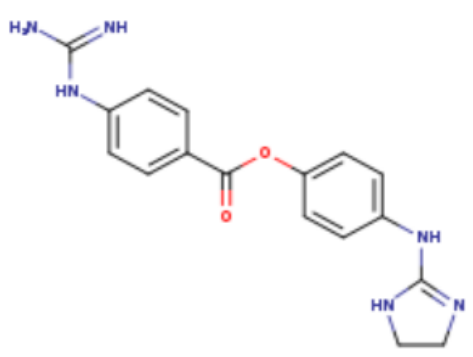

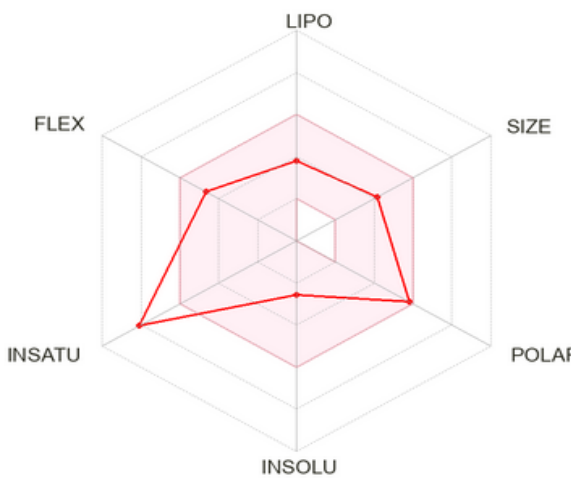

SMILES NC(=N)Nc1ccc(cc1)C(=O)Oc1ccc(cc1)NC1=NCCN1

Physicochemical Properties	
Formula	C17H18N6O2
Molecular weight	338.36 g/mol
Num. heavy atoms	25
Num. arom. heavy atoms	12
Fraction Csp3	0.12
Num. rotatable bonds	7
Num. H-bond acceptors	4
Num. H-bond donors	5
Molar Refractivity	103.98
TPSA	124.62 Å²

Lipophilicity	
Log $P_{o/w}$ (iLOGP)	1.46
Log $P_{o/w}$ (XLOGP3)	1.16
Log $P_{o/w}$ (WLOGP)	0.44
Log $P_{o/w}$ (MLOGP)	1.90
Log $P_{o/w}$ (SILICOS-IT)	1.29
Consensus Log $P_{o/w}$	1.25

Water Solubility	
Log *S* (ESOL)	-2.56
Solubility	9.28e-01 mg/ml ; 2.74e-03 mol/l
Class	Soluble
Log *S* (Ali)	-3.37
Solubility	1.44e-01 mg/ml ; 4.25e-04 mol/l
Class	Soluble
Log *S* (SILICOS-IT)	-5.33
Solubility	1.60e-03 mg/ml ; 4.73e-06 mol/l
Class	Moderately soluble

Pharmacokinetics	
GI absorption	High
BBB permeant	No
P-gp substrate	Yes
CYP1A2 inhibitor	No
CYP2C19 inhibitor	No
CYP2C9 inhibitor	No
CYP2D6 inhibitor	No
CYP3A4 inhibitor	No
Log K_p (skin permeation)	-7.54 cm/s

Druglikeness	
Lipinski	Yes; 0 violation
Ghose	Yes
Veber	Yes
Egan	Yes
Muegge	Yes
Bioavailability Score	0.55

Medicinal Chemistry	
PAINS	0 alert
Brenk	3 alerts: imine_1, imine_2, phenol_ester
Leadlikeness	Yes
Synthetic accessibility	3.03

Compound 7

Molecule 1

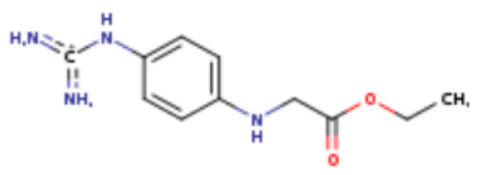

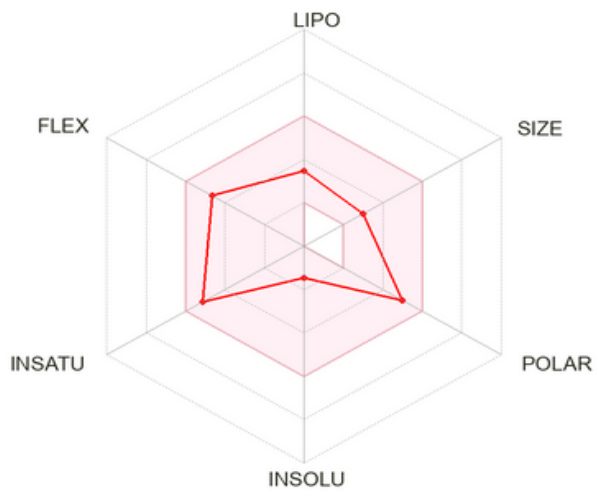

SMILES CCOC(=O)CNc1ccc(cc1)N[C+](N)N

Physicochemical Properties	
Formula	C11H17N4O2+
Molecular weight	237.28 g/mol
Num. heavy atoms	17
Num. arom. heavy atoms	6
Fraction Csp3	0.36
Num. rotatable bonds	7
Num. H-bond acceptors	4
Num. H-bond donors	4
Molar Refractivity	65.67
TPSA	102.40 Å²

Lipophilicity	
Log $P_{o/w}$ (iLOGP)	0.00
Log $P_{o/w}$ (XLOGP3)	0.56
Log $P_{o/w}$ (WLOGP)	0.06
Log $P_{o/w}$ (MLOGP)	0.55
Log $P_{o/w}$ (SILICOS-IT)	-0.47
Consensus Log $P_{o/w}$	0.14

Water Solubility	
Log *S* (ESOL)	-1.46
Solubility	8.17e+00 mg/ml ; 3.44e-02 mol/l
Class	Very soluble
Log *S* (Ali)	-2.28
Solubility	1.24e+00 mg/ml ; 5.22e-03 mol/l
Class	Soluble
Log *S* (SILICOS-IT)	-2.73
Solubility	4.47e-01 mg/ml ; 1.88e-03 mol/l
Class	Soluble

Pharmacokinetics	
GI absorption	High
BBB permeant	No
P-gp substrate	No
CYP1A2 inhibitor	No
CYP2C19 inhibitor	No
CYP2C9 inhibitor	No
CYP2D6 inhibitor	No
CYP3A4 inhibitor	No
Log K_p (skin permeation)	-7.35 cm/s

Druglikeness	
Lipinski	Yes; 0 violation
Ghose	Yes
Veber	Yes
Egan	Yes
Muegge	Yes
Bioavailability Score	0.55

Medicinal Chemistry	
PAINS	0 alert
Brenk	1 alert: carbo_cation_anion
Leadlikeness	No; 1 violation: MW<250
Synthetic accessibility	1.91

Molecule 1

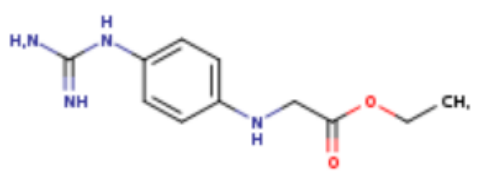

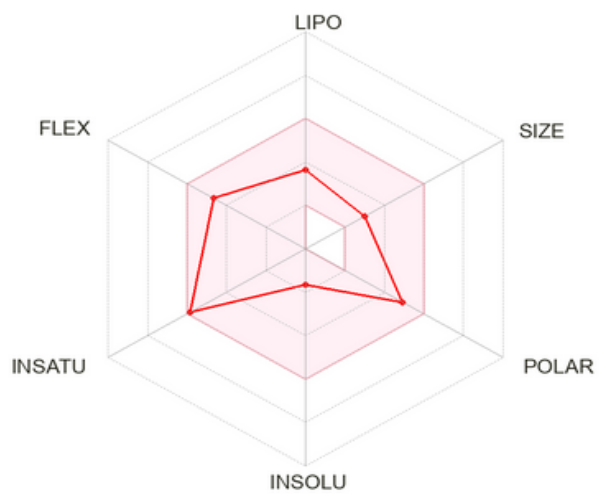

SMILES CCOC(=O)CNc1ccc(cc1)NC(=N)N

Physicochemical Properties	
Formula	C11H16N4O2
Molecular weight	236.27 g/mol
Num. heavy atoms	17
Num. arom. heavy atoms	6
Fraction Csp3	0.27
Num. rotatable bonds	7
Num. H-bond acceptors	3
Num. H-bond donors	4
Molar Refractivity	67.06
TPSA	100.23 Å²

Lipophilicity	
Log $P_{o/w}$ (iLOGP)	1.13
Log $P_{o/w}$ (XLOGP3)	0.89
Log $P_{o/w}$ (WLOGP)	0.59
Log $P_{o/w}$ (MLOGP)	0.87
Log $P_{o/w}$ (SILICOS-IT)	0.22
Consensus Log $P_{o/w}$	0.74

Water Solubility	
Log *S* (ESOL)	-1.66
Solubility	5.11e+00 mg/ml ; 2.16e-02 mol/l
Class	Very soluble
Log *S* (Ali)	-2.58
Solubility	6.22e-01 mg/ml ; 2.63e-03 mol/l
Class	Soluble
Log *S* (SILICOS-IT)	-3.13
Solubility	1.76e-01 mg/ml ; 7.45e-04 mol/l
Class	Soluble

Pharmacokinetics	
GI absorption	High
BBB permeant	No
P-gp substrate	No
CYP1A2 inhibitor	No
CYP2C19 inhibitor	No
CYP2C9 inhibitor	No
CYP2D6 inhibitor	No
CYP3A4 inhibitor	No
Log K_p (skin permeation)	-7.11 cm/s

Druglikeness	
Lipinski	Yes; 0 violation
Ghose	Yes
Veber	Yes
Egan	Yes
Muegge	Yes
Bioavailability Score	0.55

Medicinal Chemistry	
PAINS	0 alert
Brenk	2 alerts: imine_1, imine_2
Leadlikeness	No; 1 violation: MW<250
Synthetic accessibility	2.06

Compound 8

Molecule 1

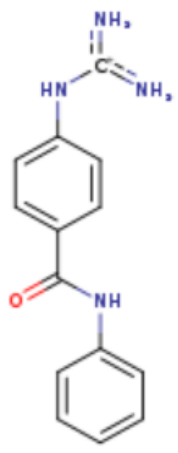

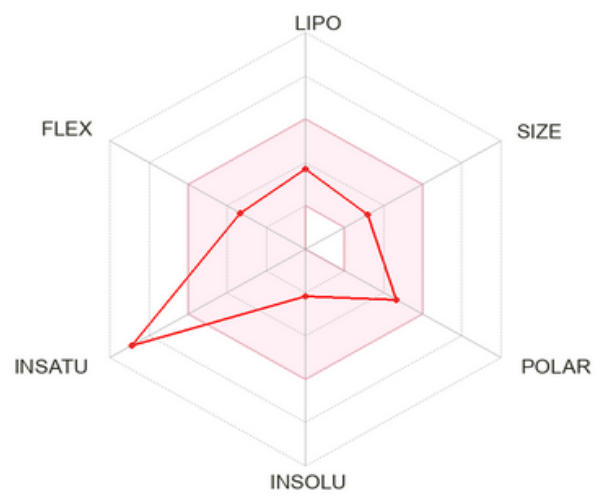

SMILES N[C+](Nc1ccc(cc1)C(=O)Nc1ccccc1)N

Physicochemical Properties	
Formula	C14H15N4O+
Molecular weight	255.30 g/mol
Num. heavy atoms	19
Num. arom. heavy atoms	12
Fraction Csp3	0.07
Num. rotatable bonds	5
Num. H-bond acceptors	3
Num. H-bond donors	4
Molar Refractivity	74.87
TPSA	93.17 Å²

Lipophilicity	
Log $P_{o/w}$ (iLOGP)	0.00
Log $P_{o/w}$ (XLOGP3)	0.98
Log $P_{o/w}$ (WLOGP)	1.33
Log $P_{o/w}$ (MLOGP)	1.79
Log $P_{o/w}$ (SILICOS-IT)	0.42
Consensus Log $P_{o/w}$	0.90

Water Solubility	
Log *S* (ESOL)	-2.18
Solubility	1.70e+00 mg/ml ; 6.64e-03 mol/l
Class	Soluble
Log *S* (Ali)	-2.52
Solubility	7.63e-01 mg/ml ; 2.99e-03 mol/l
Class	Soluble
Log *S* (SILICOS-IT)	-4.30
Solubility	1.28e-02 mg/ml ; 5.00e-05 mol/l
Class	Moderately soluble

Pharmacokinetics	
GI absorption	High
BBB permeant	No
P-gp substrate	No
CYP1A2 inhibitor	No
CYP2C19 inhibitor	No
CYP2C9 inhibitor	No
CYP2D6 inhibitor	No
CYP3A4 inhibitor	No
Log K_p (skin permeation)	-7.16 cm/s

Druglikeness	
Lipinski	Yes; 0 violation
Ghose	Yes
Veber	Yes
Egan	Yes
Muegge	Yes
Bioavailability Score	0.55

Medicinal Chemistry	
PAINS	0 alert
Brenk	1 alert: carbo_cation_anion
Leadlikeness	Yes
Synthetic accessibility	1.54

Molecule 1

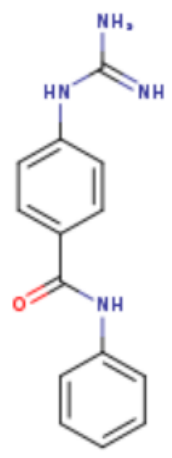

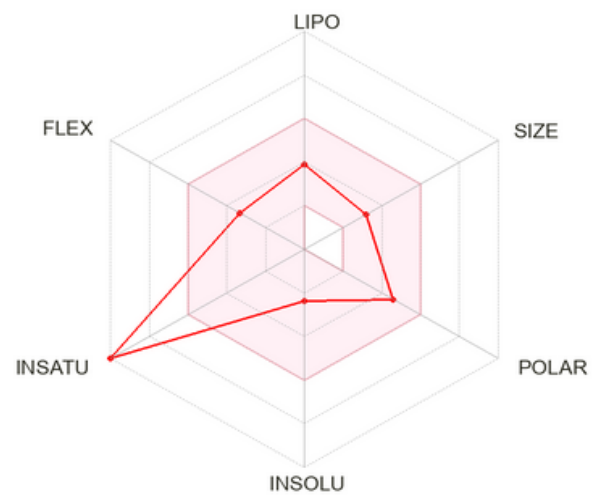

SMILES NC(=N)Nc1ccc(cc1)C(=O)Nc1ccccc1

Physicochemical Properties	
Formula	C14H14N4O
Molecular weight	254.29 g/mol
Num. heavy atoms	19
Num. arom. heavy atoms	12
Fraction Csp3	0.00
Num. rotatable bonds	5
Num. H-bond acceptors	2
Num. H-bond donors	4
Molar Refractivity	76.26
TPSA	91.00 Å²

Lipophilicity	
Log $P_{o/w}$ (iLOGP)	1.23
Log $P_{o/w}$ (XLOGP3)	1.31
Log $P_{o/w}$ (WLOGP)	1.86
Log $P_{o/w}$ (MLOGP)	2.12
Log $P_{o/w}$ (SILICOS-IT)	1.11
Consensus Log $P_{o/w}$	1.53

Water Solubility	
Log *S* (ESOL)	-2.38
Solubility	1.06e+00 mg/ml ; 4.18e-03 mol/l
Class	Soluble
Log *S* (Ali)	-2.82
Solubility	3.83e-01 mg/ml ; 1.51e-03 mol/l
Class	Soluble
Log *S* (SILICOS-IT)	-4.71
Solubility	5.02e-03 mg/ml ; 1.97e-05 mol/l
Class	Moderately soluble

Pharmacokinetics	
GI absorption	High
BBB permeant	No
P-gp substrate	No
CYP1A2 inhibitor	No
CYP2C19 inhibitor	No
CYP2C9 inhibitor	No
CYP2D6 inhibitor	No
CYP3A4 inhibitor	No
Log K_p (skin permeation)	-6.92 cm/s

Druglikeness	
Lipinski	Yes; 0 violation
Ghose	Yes
Veber	Yes
Egan	Yes
Muegge	Yes
Bioavailability Score	0.55

Medicinal Chemistry	
PAINS	0 alert
Brenk	2 alerts: imine_1, imine_2
Leadlikeness	Yes
Synthetic accessibility	1.71

Compound 9

Molecule 1

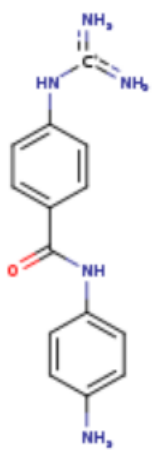

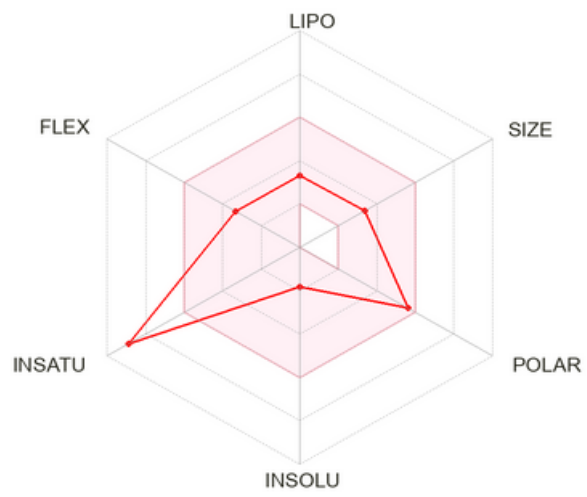

SMILES N[C+](Nc1ccc(cc1)C(=O)Nc1ccc(cc1)N)N

Physicochemical Properties	
Formula	C14H16N5O+
Molecular weight	270.31 g/mol
Num. heavy atoms	20
Num. arom. heavy atoms	12
Fraction Csp3	0.07
Num. rotatable bonds	5
Num. H-bond acceptors	3
Num. H-bond donors	5
Molar Refractivity	79.27
TPSA	119.19 Å²

Lipophilicity	
Log $P_{o/w}$ (iLOGP)	0.00
Log $P_{o/w}$ (XLOGP3)	0.30
Log $P_{o/w}$ (WLOGP)	0.92
Log $P_{o/w}$ (MLOGP)	1.25
Log $P_{o/w}$ (SILICOS-IT)	-0.30
Consensus Log $P_{o/w}$	0.43

Water Solubility	
Log *S* (ESOL)	-1.82
Solubility	4.10e+00 mg/ml ; 1.52e-02 mol/l
Class	Very soluble
Log *S* (Ali)	-2.37
Solubility	1.17e+00 mg/ml ; 4.31e-03 mol/l
Class	Soluble
Log *S* (SILICOS-IT)	-3.94
Solubility	3.11e-02 mg/ml ; 1.15e-04 mol/l
Class	Soluble

Pharmacokinetics	
GI absorption	High
BBB permeant	No
P-gp substrate	No
CYP1A2 inhibitor	No
CYP2C19 inhibitor	No
CYP2C9 inhibitor	No
CYP2D6 inhibitor	No
CYP3A4 inhibitor	No
Log K_p (skin permeation)	-7.74 cm/s

Druglikeness	
Lipinski	Yes; 0 violation
Ghose	Yes
Veber	Yes
Egan	Yes
Muegge	Yes
Bioavailability Score	0.55

Medicinal Chemistry	
PAINS	1 alert: anil_no_alk
Brenk	2 alerts: aniline, carbo_cation_anion
Leadlikeness	Yes
Synthetic accessibility	1.62

Molecule 1

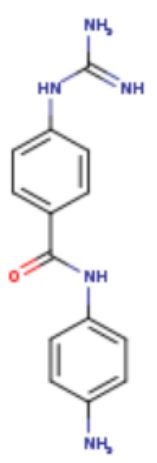

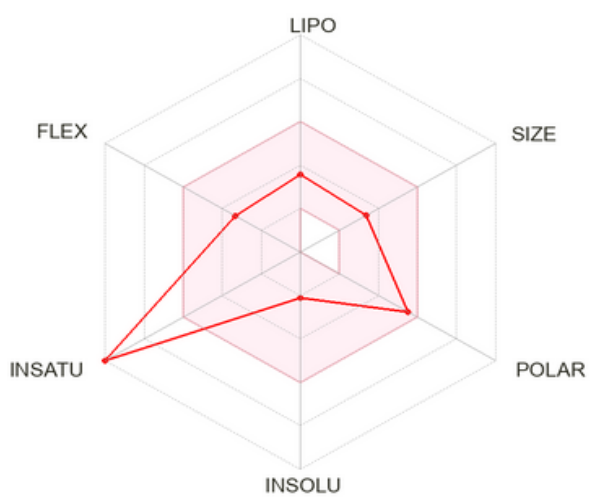

SMILES NC(=N)Nc1ccc(cc1)C(=O)Nc1ccc(cc1)N

Physicochemical Properties	
Formula	C14H15N5O
Molecular weight	269.30 g/mol
Num. heavy atoms	20
Num. arom. heavy atoms	12
Fraction Csp3	0.00
Num. rotatable bonds	5
Num. H-bond acceptors	2
Num. H-bond donors	5
Molar Refractivity	80.66
TPSA	117.02 Å²

Lipophilicity	
Log $P_{o/w}$ (iLOGP)	0.91
Log $P_{o/w}$ (XLOGP3)	0.77
Log $P_{o/w}$ (WLOGP)	1.45
Log $P_{o/w}$ (MLOGP)	1.58
Log $P_{o/w}$ (SILICOS-IT)	0.39
Consensus Log $P_{o/w}$	1.02

Water Solubility	
Log *S* (ESOL)	-2.11
Solubility	2.10e+00 mg/ml ; 7.78e-03 mol/l
Class	Soluble
Log *S* (Ali)	-2.81
Solubility	4.19e-01 mg/ml ; 1.56e-03 mol/l
Class	Soluble
Log *S* (SILICOS-IT)	-4.34
Solubility	1.22e-02 mg/ml ; 4.54e-05 mol/l
Class	Moderately soluble

Pharmacokinetics	
GI absorption	High
BBB permeant	No
P-gp substrate	No
CYP1A2 inhibitor	No
CYP2C19 inhibitor	No
CYP2C9 inhibitor	No
CYP2D6 inhibitor	No
CYP3A4 inhibitor	No
Log K_p (skin permeation)	-7.40 cm/s

Druglikeness	
Lipinski	Yes; 0 violation
Ghose	Yes
Veber	Yes
Egan	Yes
Muegge	Yes
Bioavailability Score	0.55

Medicinal Chemistry	
PAINS	1 alert: anil_no_alk
Brenk	3 alerts: aniline, imine_1, imine_2
Leadlikeness	Yes
Synthetic accessibility	1.79

Compound 10

Molecule 1

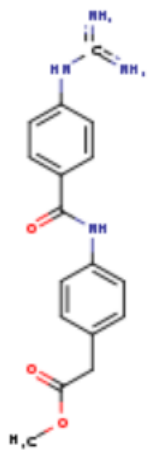

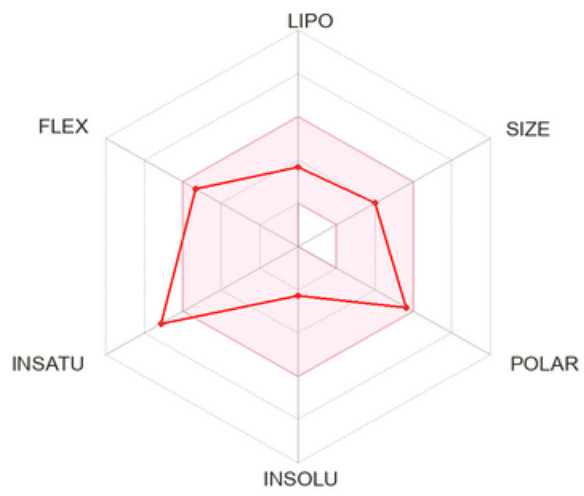

SMILES COC(=O)Cc1ccc(cc1)NC(=O)c1ccc(cc1)N[C+](N)N

Physicochemical Properties	
Formula	C17H19N4O3+
Molecular weight	327.36 g/mol
Num. heavy atoms	24
Num. arom. heavy atoms	12
Fraction Csp3	0.18
Num. rotatable bonds	8
Num. H-bond acceptors	5
Num. H-bond donors	4
Molar Refractivity	90.73
TPSA	119.47 Å²

Lipophilicity	
Log $P_{o/w}$ (iLOGP)	0.00
Log $P_{o/w}$ (XLOGP3)	0.91
Log $P_{o/w}$ (WLOGP)	1.05
Log $P_{o/w}$ (MLOGP)	1.67
Log $P_{o/w}$ (SILICOS-IT)	0.80
Consensus Log $P_{o/w}$	0.88

Water Solubility	
Log *S* (ESOL)	-2.28
Solubility	1.70e+00 mg/ml ; 5.19e-03 mol/l
Class	Soluble
Log *S* (Ali)	-3.00
Solubility	3.24e-01 mg/ml ; 9.90e-04 mol/l
Class	Soluble
Log *S* (SILICOS-IT)	-4.75
Solubility	5.78e-03 mg/ml ; 1.77e-05 mol/l
Class	Moderately soluble

Pharmacokinetics	
GI absorption	High
BBB permeant	No
P-gp substrate	No
CYP1A2 inhibitor	No
CYP2C19 inhibitor	No
CYP2C9 inhibitor	No
CYP2D6 inhibitor	No
CYP3A4 inhibitor	No
Log K_p (skin permeation)	-7.65 cm/s

Druglikeness	
Lipinski	Yes; 0 violation
Ghose	Yes
Veber	Yes
Egan	Yes
Muegge	Yes
Bioavailability Score	0.55

Medicinal Chemistry	
PAINS	0 alert
Brenk	1 alert: carbo_cation_anion
Leadlikeness	No; 1 violation: Rotors>7
Synthetic accessibility	2.10

Molecule 1

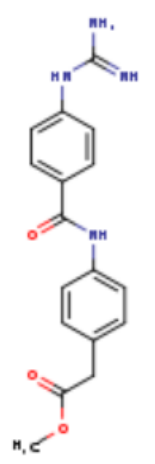

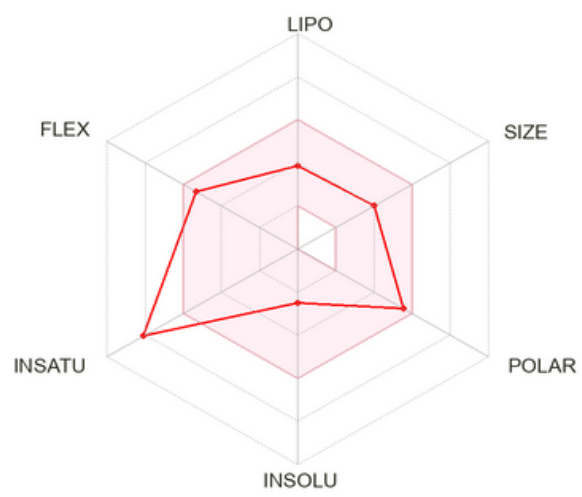

SMILES COC(=O)Cc1ccc(cc1)NC(=O)c1ccc(cc1)NC(=N)N

Physicochemical Properties	
Formula	C17H18N4O3
Molecular weight	326.35 g/mol
Num. heavy atoms	24
Num. arom. heavy atoms	12
Fraction Csp3	0.12
Num. rotatable bonds	8
Num. H-bond acceptors	4
Num. H-bond donors	4
Molar Refractivity	92.12
TPSA	117.30 Å²

Lipophilicity	
Log $P_{o/w}$ (iLOGP)	1.78
Log $P_{o/w}$ (XLOGP3)	1.24
Log $P_{o/w}$ (WLOGP)	1.58
Log $P_{o/w}$ (MLOGP)	2.00
Log $P_{o/w}$ (SILICOS-IT)	1.49
Consensus Log $P_{o/w}$	1.62

Water Solubility	
Log *S* (ESOL)	-2.49
Solubility	1.06e+00 mg/ml ; 3.26e-03 mol/l
Class	Soluble
Log *S* (Ali)	-3.30
Solubility	1.63e-01 mg/ml ; 5.00e-04 mol/l
Class	Soluble
Log *S* (SILICOS-IT)	-5.16
Solubility	2.27e-03 mg/ml ; 6.95e-06 mol/l
Class	Moderately soluble

Pharmacokinetics	
GI absorption	High
BBB permeant	No
P-gp substrate	No
CYP1A2 inhibitor	No
CYP2C19 inhibitor	No
CYP2C9 inhibitor	No
CYP2D6 inhibitor	No
CYP3A4 inhibitor	No
Log K_p (skin permeation)	-7.41 cm/s

Druglikeness	
Lipinski	Yes; 0 violation
Ghose	Yes
Veber	Yes
Egan	Yes
Muegge	Yes
Bioavailability Score	0.55

Medicinal Chemistry	
PAINS	0 alert
Brenk	2 alerts: imine_1, imine_2
Leadlikeness	No; 1 violation: Rotors>7
Synthetic accessibility	2.20

Compound 11

Molecule 1

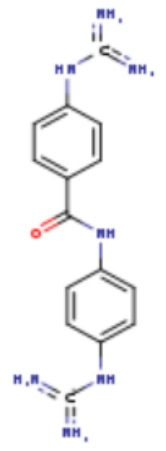

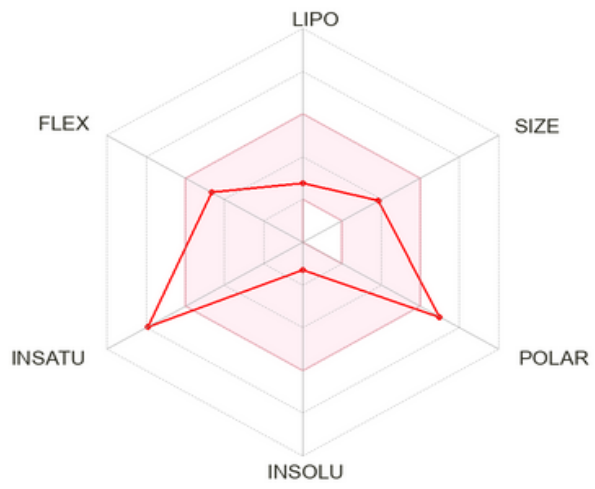

SMILES N[C+](Nc1ccc(cc1)NC(=O)c1ccc(cc1)N[C+](N)N)N

Physicochemical Properties	
Formula	C15H19N7O++
Molecular weight	313.36 g/mol
Num. heavy atoms	23
Num. arom. heavy atoms	12
Fraction Csp3	0.13
Num. rotatable bonds	7
Num. H-bond acceptors	5
Num. H-bond donors	7
Molar Refractivity	89.08
TPSA	157.24 Å²

Lipophilicity	
Log $P_{o/w}$ (iLOGP)	0.00
Log $P_{o/w}$ (XLOGP3)	-0.65
Log $P_{o/w}$ (WLOGP)	-0.08
Log $P_{o/w}$ (MLOGP)	0.73
Log $P_{o/w}$ (SILICOS-IT)	-1.70
Consensus Log $P_{o/w}$	-0.34

Water Solubility	
Log *S* (ESOL)	-1.30
Solubility	1.58e+01 mg/ml ; 5.04e-02 mol/l
Class	Very soluble
Log *S* (Ali)	-2.18
Solubility	2.08e+00 mg/ml ; 6.63e-03 mol/l
Class	Soluble
Log *S* (SILICOS-IT)	-3.66
Solubility	6.84e-02 mg/ml ; 2.18e-04 mol/l
Class	Soluble

Pharmacokinetics	
GI absorption	Low
BBB permeant	No
P-gp substrate	No
CYP1A2 inhibitor	No
CYP2C19 inhibitor	No
CYP2C9 inhibitor	No
CYP2D6 inhibitor	No
CYP3A4 inhibitor	No
Log K_p (skin permeation)	-8.67 cm/s

Druglikeness	
Lipinski	Yes; 1 violation: NHorOH>5
Ghose	Yes
Veber	No; 1 violation: TPSA>140
Egan	No; 1 violation: TPSA>131.6
Muegge	No; 2 violations: TPSA>150, H-don>5
Bioavailability Score	0.55

Medicinal Chemistry	
PAINS	0 alert
Brenk	1 alert: carbo_cation_anion
Leadlikeness	Yes
Synthetic accessibility	1.83

Molecule 1

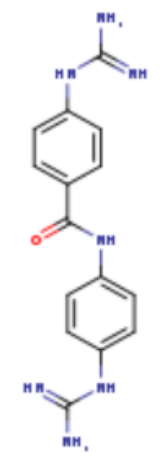

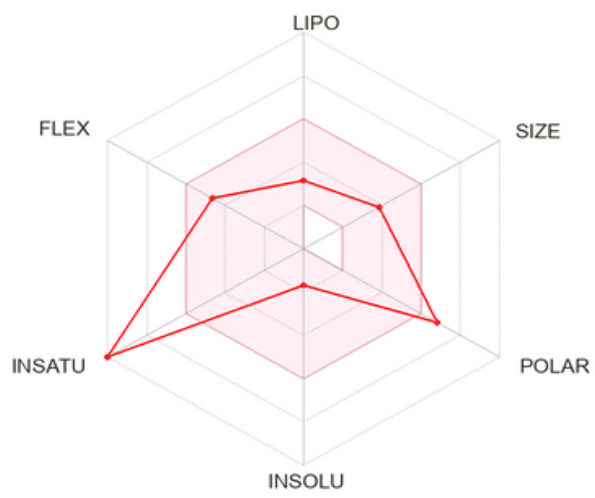

SMILES NC(=N)Nc1ccc(cc1)NC(=O)c1ccc(cc1)NC(=N)N

Physicochemical Properties	
Formula	C15H17N7O
Molecular weight	311.34 g/mol
Num. heavy atoms	23
Num. arom. heavy atoms	12
Fraction Csp3	0.00
Num. rotatable bonds	7
Num. H-bond acceptors	3
Num. H-bond donors	7
Molar Refractivity	91.86
TPSA	152.90 Å²

Lipophilicity	
Log $P_{o/w}$ (iLOGP)	0.68
Log $P_{o/w}$ (XLOGP3)	0.01
Log $P_{o/w}$ (WLOGP)	0.98
Log $P_{o/w}$ (MLOGP)	1.39
Log $P_{o/w}$ (SILICOS-IT)	-0.31
Consensus Log $P_{o/w}$	0.55

Water Solubility	
Log *S* (ESOL)	-1.70
Solubility	6.20e+00 mg/ml ; 1.99e-02 mol/l
Class	Very soluble
Log *S* (Ali)	-2.77
Solubility	5.26e-01 mg/ml ; 1.69e-03 mol/l
Class	Soluble
Log *S* (SILICOS-IT)	-4.47
Solubility	1.05e-02 mg/ml ; 3.38e-05 mol/l
Class	Moderately soluble

Pharmacokinetics	
GI absorption	Low
BBB permeant	No
P-gp substrate	No
CYP1A2 inhibitor	No
CYP2C19 inhibitor	No
CYP2C9 inhibitor	No
CYP2D6 inhibitor	No
CYP3A4 inhibitor	No
Log K_p (skin permeation)	-8.19 cm/s

Druglikeness	
Lipinski	Yes; 1 violation: NHorOH>5
Ghose	Yes
Veber	No; 1 violation: TPSA>140
Egan	No; 1 violation: TPSA>131.6
Muegge	No; 2 violations: TPSA>150, H-don>5
Bioavailability Score	0.55

Medicinal Chemistry	
PAINS	0 alert
Brenk	2 alerts: imine_1, imine_2
Leadlikeness	Yes
Synthetic accessibility	1.96

Compound 12

Molecule 1

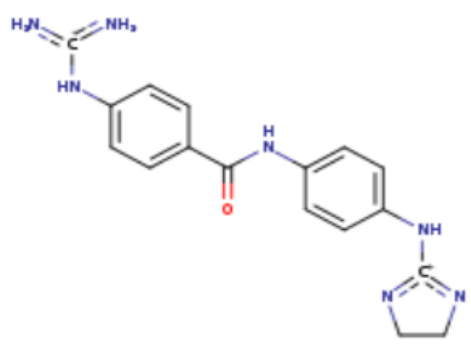

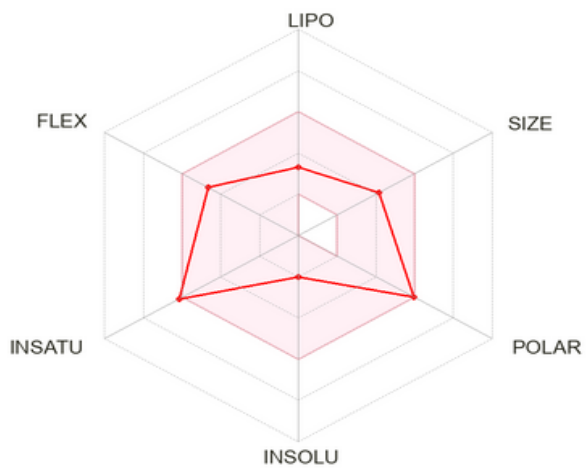

SMILES N[C+](Nc1ccc(cc1)C(=O)Nc1ccc(cc1)N[C+]1NCCN1)N

Physicochemical Properties	
Formula	C17H21N7O++
Molecular weight	339.39 g/mol
Num. heavy atoms	25
Num. arom. heavy atoms	12
Fraction Csp3	0.24
Num. rotatable bonds	7
Num. H-bond acceptors	5
Num. H-bond donors	7
Molar Refractivity	104.60
TPSA	129.26 Å²

Lipophilicity	
Log $P_{o/w}$ (iLOGP)	0.00
Log $P_{o/w}$ (XLOGP3)	0.28
Log $P_{o/w}$ (WLOGP)	-0.57
Log $P_{o/w}$ (MLOGP)	0.83
Log $P_{o/w}$ (SILICOS-IT)	-0.79
Consensus Log $P_{o/w}$	-0.05

Water Solubility	
Log *S* (ESOL)	-2.01
Solubility	3.29e+00 mg/ml ; 9.69e-03 mol/l
Class	Soluble
Log *S* (Ali)	-2.56
Solubility	9.43e-01 mg/ml ; 2.78e-03 mol/l
Class	Soluble
Log *S* (SILICOS-IT)	-5.02
Solubility	3.26e-03 mg/ml ; 9.59e-06 mol/l
Class	Moderately soluble

Pharmacokinetics	
GI absorption	High
BBB permeant	No
P-gp substrate	Yes
CYP1A2 inhibitor	No
CYP2C19 inhibitor	No
CYP2C9 inhibitor	No
CYP2D6 inhibitor	No
CYP3A4 inhibitor	No
Log K_p (skin permeation)	-8.17 cm/s

Druglikeness	
Lipinski	Yes; 1 violation: NHorOH>5
Ghose	No; 1 violation: WLOGP<-0.4
Veber	Yes
Egan	Yes
Muegge	No; 1 violation: H-don>5
Bioavailability Score	0.55

Medicinal Chemistry	
PAINS	0 alert
Brenk	1 alert: carbo_cation_anion
Leadlikeness	Yes
Synthetic accessibility	2.30

Molecule 1

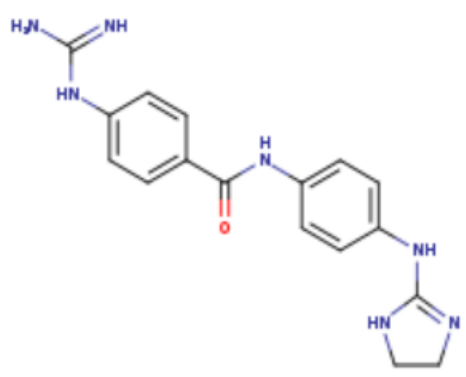

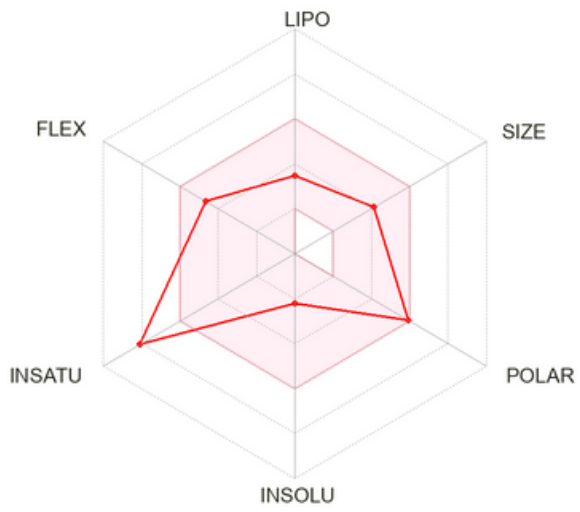

SMILES NC(=N)Nc1ccc(cc1)C(=O)Nc1ccc(cc1)NC1=NCCN1

Physicochemical Properties	
Formula	C17H19N7O
Molecular weight	337.38 g/mol
Num. heavy atoms	25
Num. arom. heavy atoms	12
Fraction Csp3	0.12
Num. rotatable bonds	7
Num. H-bond acceptors	3
Num. H-bond donors	6
Molar Refractivity	106.79
TPSA	127.42 Å²

Lipophilicity	
Log $P_{o/w}$ (iLOGP)	1.01
Log $P_{o/w}$ (XLOGP3)	0.59
Log $P_{o/w}$ (WLOGP)	0.28
Log $P_{o/w}$ (MLOGP)	1.49
Log $P_{o/w}$ (SILICOS-IT)	0.97
Consensus Log $P_{o/w}$	0.87

Water Solubility	
Log *S* (ESOL)	-2.20
Solubility	2.15e+00 mg/ml ; 6.36e-03 mol/l
Class	Soluble
Log *S* (Ali)	-2.84
Solubility	4.88e-01 mg/ml ; 1.45e-03 mol/l
Class	Soluble
Log *S* (SILICOS-IT)	-5.64
Solubility	7.78e-04 mg/ml ; 2.31e-06 mol/l
Class	Moderately soluble

Pharmacokinetics	
GI absorption	High
BBB permeant	No
P-gp substrate	Yes
CYP1A2 inhibitor	No
CYP2C19 inhibitor	No
CYP2C9 inhibitor	No
CYP2D6 inhibitor	No
CYP3A4 inhibitor	No
Log K_p (skin permeation)	-7.94 cm/s

Druglikeness	
Lipinski	Yes; 1 violation: NHorOH>5
Ghose	Yes
Veber	Yes
Egan	Yes
Muegge	No; 1 violation: H-don>5
Bioavailability Score	0.55

Medicinal Chemistry	
PAINS	0 alert
Brenk	2 alerts: imine_1, imine_2
Leadlikeness	Yes
Synthetic accessibility	2.94

Compound 13

Molecule 1

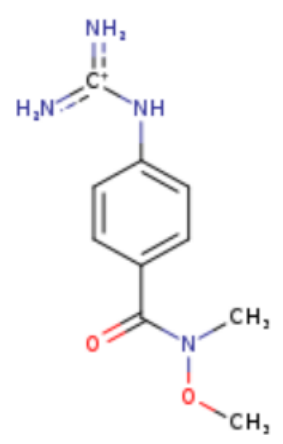

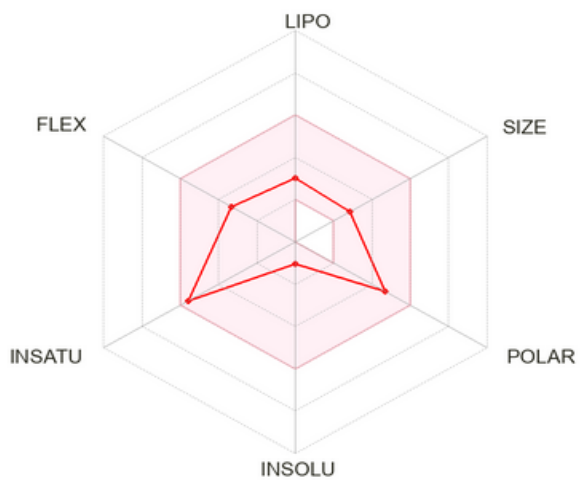

SMILES CON(C(=O)c1ccc(cc1)N[C+](N)N)C

Physicochemical Properties	
Formula	C10H15N4O2+
Molecular weight	223.25 g/mol
Num. heavy atoms	16
Num. arom. heavy atoms	6
Fraction Csp3	0.30
Num. rotatable bonds	5
Num. H-bond acceptors	4
Num. H-bond donors	3
Molar Refractivity	59.64
TPSA	93.61 Å²

Lipophilicity	
Log $P_{o/w}$ (iLOGP)	0.00
Log $P_{o/w}$ (XLOGP3)	-0.21
Log $P_{o/w}$ (WLOGP)	-0.09
Log $P_{o/w}$ (MLOGP)	0.67
Log $P_{o/w}$ (SILICOS-IT)	-1.29
Consensus Log $P_{o/w}$	-0.19

Water Solubility	
Log *S* (ESOL)	-1.04
Solubility	2.04e+01 mg/ml ; 9.13e-02 mol/l
Class	Very soluble
Log *S* (Ali)	-1.30
Solubility	1.12e+01 mg/ml ; 5.02e-02 mol/l
Class	Very soluble
Log *S* (SILICOS-IT)	-1.59
Solubility	5.76e+00 mg/ml ; 2.58e-02 mol/l
Class	Soluble

Pharmacokinetics	
GI absorption	High
BBB permeant	No
P-gp substrate	No
CYP1A2 inhibitor	No
CYP2C19 inhibitor	No
CYP2C9 inhibitor	No
CYP2D6 inhibitor	No
CYP3A4 inhibitor	No
Log K_p (skin permeation)	-7.81 cm/s

Druglikeness	
Lipinski	Yes; 0 violation
Ghose	Yes
Veber	Yes
Egan	Yes
Muegge	Yes
Bioavailability Score	0.55

Medicinal Chemistry	
PAINS	0 alert
Brenk	2 alerts: carbo_cation_anion, oxygen-nitrogen_single_bond
Leadlikeness	No; 1 violation: MW<250
Synthetic accessibility	1.76

Molecule 1

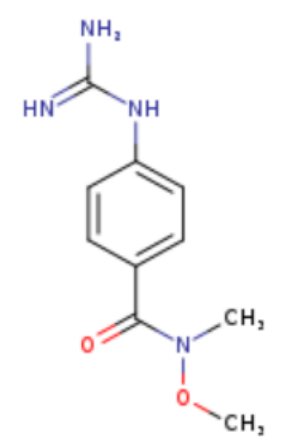

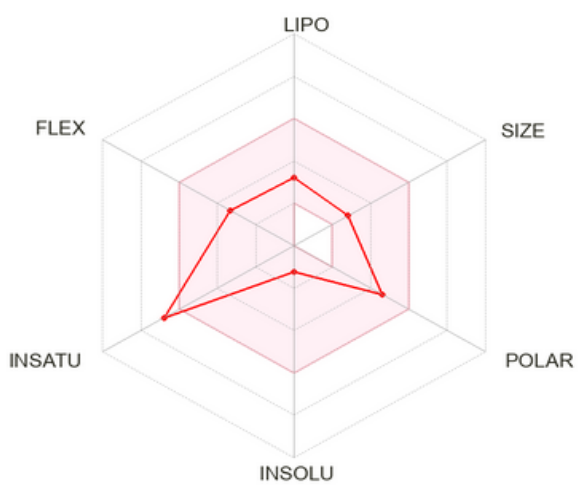

SMILES CON(C(=O)c1ccc(cc1)NC(=N)N)C

Physicochemical Properties	
Formula	C10H14N4O2
Molecular weight	222.24 g/mol
Num. heavy atoms	16
Num. arom. heavy atoms	6
Fraction Csp3	0.20
Num. rotatable bonds	5
Num. H-bond acceptors	3
Num. H-bond donors	3
Molar Refractivity	61.03
TPSA	91.44 Å²

Lipophilicity	
Log $P_{o/w}$ (iLOGP)	1.42
Log $P_{o/w}$ (XLOGP3)	0.12
Log $P_{o/w}$ (WLOGP)	0.43
Log $P_{o/w}$ (MLOGP)	0.99
Log $P_{o/w}$ (SILICOS-IT)	-0.60
Consensus Log $P_{o/w}$	0.47

Water Solubility	
Log *S* (ESOL)	-1.24
Solubility	1.28e+01 mg/ml ; 5.74e-02 mol/l
Class	Very soluble
Log *S* (Ali)	-1.60
Solubility	5.63e+00 mg/ml ; 2.54e-02 mol/l
Class	Very soluble
Log *S* (SILICOS-IT)	-1.99
Solubility	2.27e+00 mg/ml ; 1.02e-02 mol/l
Class	Soluble

Pharmacokinetics	
GI absorption	High
BBB permeant	No
P-gp substrate	No
CYP1A2 inhibitor	No
CYP2C19 inhibitor	No
CYP2C9 inhibitor	No
CYP2D6 inhibitor	No
CYP3A4 inhibitor	No
Log K_p (skin permeation)	-7.57 cm/s

Druglikeness	
Lipinski	Yes; 0 violation
Ghose	Yes
Veber	Yes
Egan	Yes
Muegge	Yes
Bioavailability Score	0.55

Medicinal Chemistry	
PAINS	0 alert
Brenk	3 alerts: imine_1, imine_2, oxygen-nitrogen_single_bond
Leadlikeness	No; 1 violation: MW<250
Synthetic accessibility	1.86

Compound 14

Molecule 1

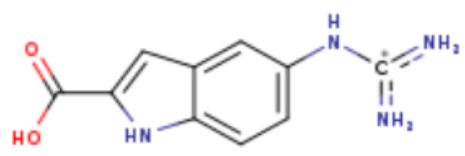

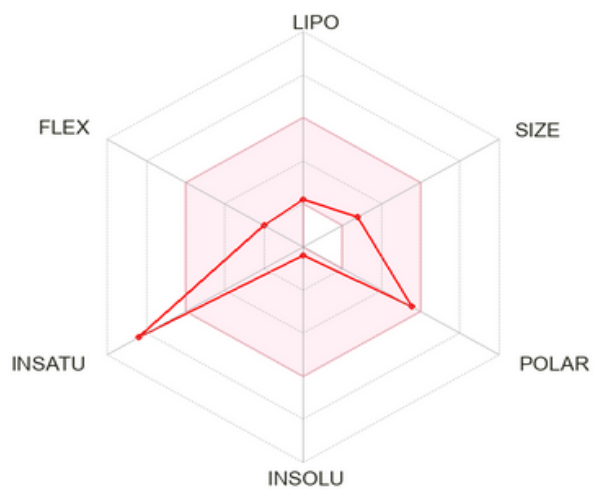

SMILES N[C+](Nc1ccc2c(c1)cc([nH]2)C(=O)O)N

Physicochemical Properties	
Formula	C10H11N4O2+
Molecular weight	219.22 g/mol
Num. heavy atoms	16
Num. arom. heavy atoms	9
Fraction Csp3	0.10
Num. rotatable bonds	3
Num. H-bond acceptors	4
Num. H-bond donors	5
Molar Refractivity	59.47
TPSA	117.16 Å²

Lipophilicity	
Log $P_{o/w}$ (iLOGP)	0.00
Log $P_{o/w}$ (XLOGP3)	-1.61
Log $P_{o/w}$ (WLOGP)	0.45
Log $P_{o/w}$ (MLOGP)	-0.03
Log $P_{o/w}$ (SILICOS-IT)	-0.31
Consensus Log $P_{o/w}$	-0.30

Water Solubility	
Log *S* (ESOL)	-0.40
Solubility	8.66e+01 mg/ml ; 3.95e-01 mol/l
Class	Very soluble
Log *S* (Ali)	-0.34
Solubility	1.00e+02 mg/ml ; 4.56e-01 mol/l
Class	Very soluble
Log *S* (SILICOS-IT)	-2.05
Solubility	1.97e+00 mg/ml ; 8.97e-03 mol/l
Class	Soluble

Pharmacokinetics	
GI absorption	High
BBB permeant	No
P-gp substrate	No
CYP1A2 inhibitor	No
CYP2C19 inhibitor	No
CYP2C9 inhibitor	No
CYP2D6 inhibitor	No
CYP3A4 inhibitor	No
Log K_p (skin permeation)	-8.78 cm/s

Druglikeness	
Lipinski	Yes; 0 violation
Ghose	Yes
Veber	Yes
Egan	Yes
Muegge	Yes
Bioavailability Score	0.55

Medicinal Chemistry	
PAINS	0 alert
Brenk	1 alert: carbo_cation_anion
Leadlikeness	No; 1 violation: MW<250
Synthetic accessibility	1.69

Molecule 1

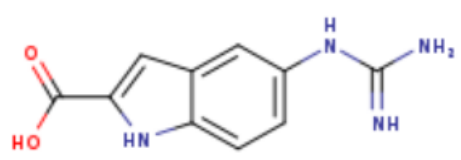

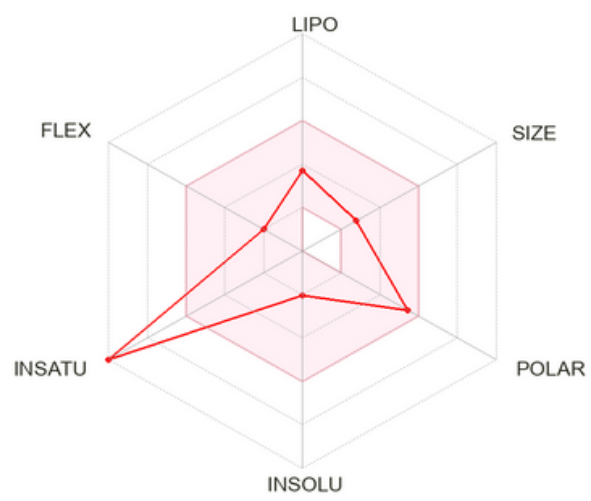

SMILES NC(=N)Nc1ccc2c(c1)cc([nH]2)C(=O)O

Physicochemical Properties	
Formula	C10H10N4O2
Molecular weight	218.21 g/mol
Num. heavy atoms	16
Num. arom. heavy atoms	9
Fraction Csp3	0.00
Num. rotatable bonds	3
Num. H-bond acceptors	3
Num. H-bond donors	5
Molar Refractivity	60.86
TPSA	114.99 Å²

Lipophilicity	
Log $P_{o/w}$ (iLOGP)	0.49
Log $P_{o/w}$ (XLOGP3)	1.00
Log $P_{o/w}$ (WLOGP)	0.98
Log $P_{o/w}$ (MLOGP)	0.29
Log $P_{o/w}$ (SILICOS-IT)	0.38
Consensus Log $P_{o/w}$	0.63

Water Solubility	
Log *S* (ESOL)	-2.04
Solubility	1.98e+00 mg/ml ; 9.10e-03 mol/l
Class	Soluble
Log *S* (Ali)	-3.00
Solubility	2.16e-01 mg/ml ; 9.92e-04 mol/l
Class	Soluble
Log *S* (SILICOS-IT)	-2.45
Solubility	7.72e-01 mg/ml ; 3.54e-03 mol/l
Class	Soluble

Pharmacokinetics	
GI absorption	High
BBB permeant	No
P-gp substrate	No
CYP1A2 inhibitor	No
CYP2C19 inhibitor	No
CYP2C9 inhibitor	No
CYP2D6 inhibitor	No
CYP3A4 inhibitor	No
Log K_p (skin permeation)	-6.92 cm/s

Druglikeness	
Lipinski	Yes; 0 violation
Ghose	Yes
Veber	Yes
Egan	Yes
Muegge	Yes
Bioavailability Score	0.55

Medicinal Chemistry	
PAINS	0 alert
Brenk	2 alerts: imine_1, imine_2
Leadlikeness	No; 1 violation: MW<250
Synthetic accessibility	1.78

Compound 15

Molecule 1

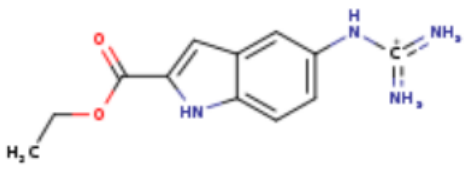

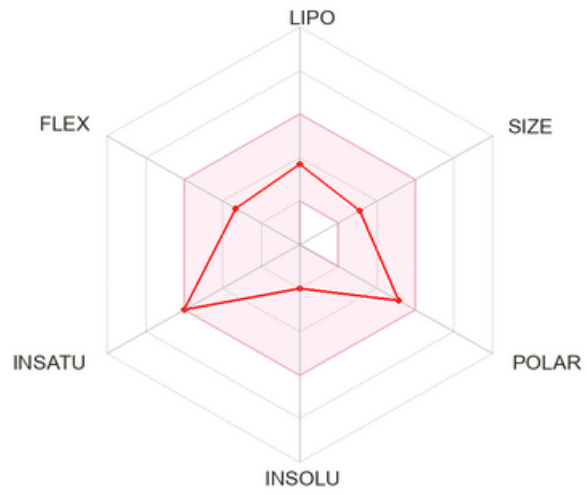

SMILES CCOC(=O)c1cc2c([nH]1)ccc(c2)N[C+](N)N

Physicochemical Properties	
Formula	C12H15N4O2+
Molecular weight	247.27 g/mol
Num. heavy atoms	18
Num. arom. heavy atoms	9
Fraction Csp3	0.25
Num. rotatable bonds	5
Num. H-bond acceptors	4
Num. H-bond donors	4
Molar Refractivity	68.60
TPSA	106.16 Å²

Lipophilicity	
Log $P_{o/w}$ (iLOGP)	0.00
Log $P_{o/w}$ (XLOGP3)	0.96
Log $P_{o/w}$ (WLOGP)	0.93
Log $P_{o/w}$ (MLOGP)	0.54
Log $P_{o/w}$ (SILICOS-IT)	0.56
Consensus Log $P_{o/w}$	0.60

Water Solubility	
Log *S* (ESOL)	-2.02
Solubility	2.37e+00 mg/ml ; 9.60e-03 mol/l
Class	Soluble
Log *S* (Ali)	-2.78
Solubility	4.13e-01 mg/ml ; 1.67e-03 mol/l
Class	Soluble
Log *S* (SILICOS-IT)	-3.15
Solubility	1.74e-01 mg/ml ; 7.03e-04 mol/l
Class	Soluble

Pharmacokinetics	
GI absorption	High
BBB permeant	No
P-gp substrate	Yes
CYP1A2 inhibitor	No
CYP2C19 inhibitor	No
CYP2C9 inhibitor	No
CYP2D6 inhibitor	No
CYP3A4 inhibitor	No
Log K_p (skin permeation)	-7.13 cm/s

Druglikeness	
Lipinski	Yes; 0 violation
Ghose	Yes
Veber	Yes
Egan	Yes
Muegge	Yes
Bioavailability Score	0.55

Medicinal Chemistry	
PAINS	0 alert
Brenk	1 alert: carbo_cation_anion
Leadlikeness	No; 1 violation: MW<250
Synthetic accessibility	2.12

Molecule 1

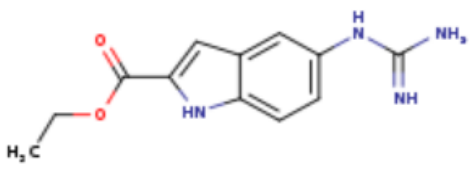

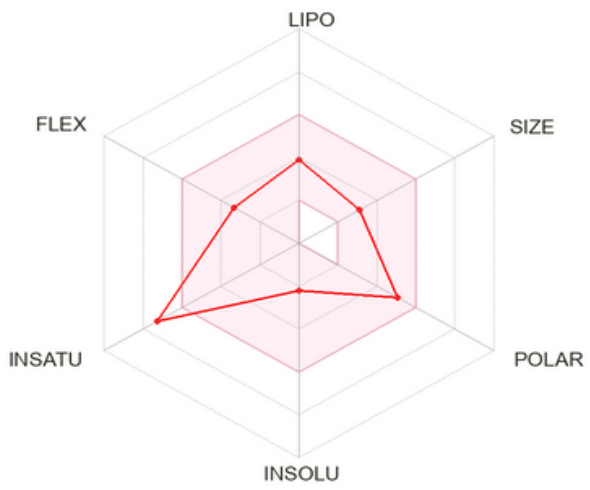

SMILES CCOC(=O)c1cc2c([nH]1)ccc(c2)NC(=N)N

Physicochemical Properties	
Formula	C12H14N4O2
Molecular weight	246.27 g/mol
Num. heavy atoms	18
Num. arom. heavy atoms	9
Fraction Csp3	0.17
Num. rotatable bonds	5
Num. H-bond acceptors	3
Num. H-bond donors	4
Molar Refractivity	69.99
TPSA	103.99 Å²

Lipophilicity	
Log $P_{o/w}$ (iLOGP)	1.52
Log $P_{o/w}$ (XLOGP3)	1.29
Log $P_{o/w}$ (WLOGP)	1.46
Log $P_{o/w}$ (MLOGP)	0.87
Log $P_{o/w}$ (SILICOS-IT)	1.25
Consensus Log $P_{o/w}$	1.28

Water Solubility	
Log *S* (ESOL)	-2.22
Solubility	1.49e+00 mg/ml ; 6.03e-03 mol/l
Class	Soluble
Log *S* (Ali)	-3.07
Solubility	2.08e-01 mg/ml ; 8.44e-04 mol/l
Class	Soluble
Log *S* (SILICOS-IT)	-3.56
Solubility	6.83e-02 mg/ml ; 2.77e-04 mol/l
Class	Soluble

Pharmacokinetics	
GI absorption	High
BBB permeant	No
P-gp substrate	No
CYP1A2 inhibitor	No
CYP2C19 inhibitor	No
CYP2C9 inhibitor	No
CYP2D6 inhibitor	No
CYP3A4 inhibitor	No
Log K_p (skin permeation)	-6.89 cm/s

Druglikeness	
Lipinski	Yes; 0 violation
Ghose	Yes
Veber	Yes
Egan	Yes
Muegge	Yes
Bioavailability Score	0.55

Medicinal Chemistry	
PAINS	0 alert
Brenk	2 alerts: imine_1, imine_2
Leadlikeness	No; 1 violation: MW<250
Synthetic accessibility	2.18

Compound 16

Molecule 1

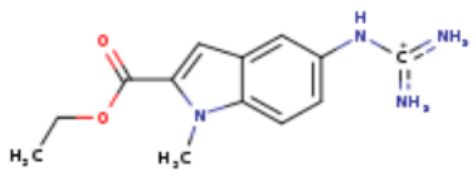

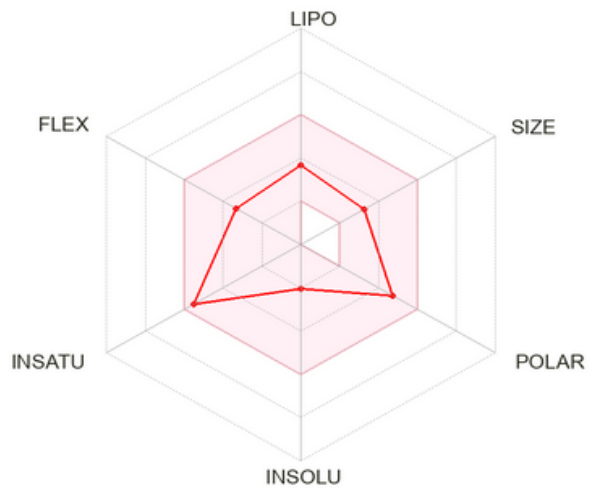

SMILES CCOC(=O)c1cc2c(n1C)ccc(c2)N[C+](N)N

Physicochemical Properties	
Formula	C13H17N4O2+
Molecular weight	261.30 g/mol
Num. heavy atoms	19
Num. arom. heavy atoms	9
Fraction Csp3	0.31
Num. rotatable bonds	5
Num. H-bond acceptors	4
Num. H-bond donors	3
Molar Refractivity	73.50
TPSA	95.30 Å²

Lipophilicity	
Log $P_{o/w}$ (iLOGP)	0.00
Log $P_{o/w}$ (XLOGP3)	0.92
Log $P_{o/w}$ (WLOGP)	0.94
Log $P_{o/w}$ (MLOGP)	0.81
Log $P_{o/w}$ (SILICOS-IT)	0.02
Consensus Log $P_{o/w}$	0.54

Water Solubility	
Log *S* (ESOL)	-2.06
Solubility	2.27e+00 mg/ml ; 8.71e-03 mol/l
Class	Soluble
Log *S* (Ali)	-2.51
Solubility	8.13e-01 mg/ml ; 3.11e-03 mol/l
Class	Soluble
Log *S* (SILICOS-IT)	-2.71
Solubility	5.07e-01 mg/ml ; 1.94e-03 mol/l
Class	Soluble

Pharmacokinetics	
GI absorption	High
BBB permeant	No
P-gp substrate	Yes
CYP1A2 inhibitor	No
CYP2C19 inhibitor	No
CYP2C9 inhibitor	No
CYP2D6 inhibitor	No
CYP3A4 inhibitor	No
Log K_p (skin permeation)	-7.24 cm/s

Druglikeness	
Lipinski	Yes; 0 violation
Ghose	Yes
Veber	Yes
Egan	Yes
Muegge	Yes
Bioavailability Score	0.55

Medicinal Chemistry	
PAINS	0 alert
Brenk	1 alert: carbo_cation_anion
Leadlikeness	Yes
Synthetic accessibility	2.24

Molecule 1

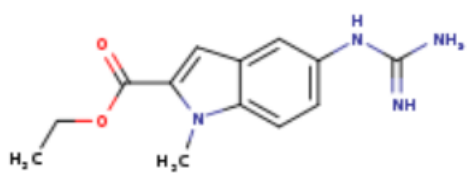

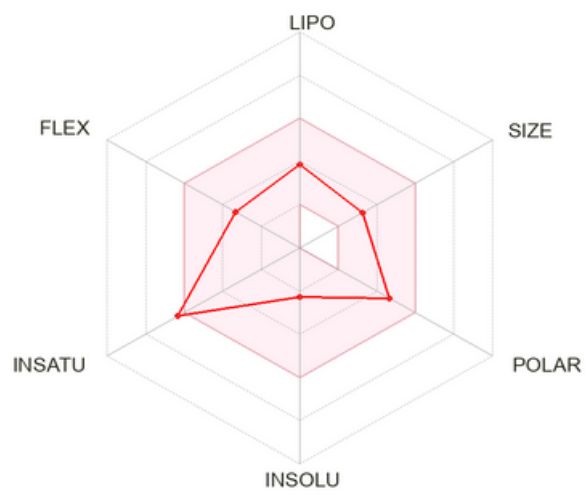

SMILES CCOC(=O)c1cc2c(n1C)ccc(c2)NC(=N)N

Physicochemical Properties	
Formula	C13H16N4O2
Molecular weight	260.29 g/mol
Num. heavy atoms	19
Num. arom. heavy atoms	9
Fraction Csp3	0.23
Num. rotatable bonds	5
Num. H-bond acceptors	3
Num. H-bond donors	3
Molar Refractivity	74.89
TPSA	93.13 Å²

Lipophilicity	
Log $P_{o/w}$ (iLOGP)	2.01
Log $P_{o/w}$ (XLOGP3)	1.25
Log $P_{o/w}$ (WLOGP)	1.47
Log $P_{o/w}$ (MLOGP)	1.14
Log $P_{o/w}$ (SILICOS-IT)	0.71
Consensus Log $P_{o/w}$	1.32

Water Solubility	
Log *S* (ESOL)	-2.26
Solubility	1.42e+00 mg/ml ; 5.47e-03 mol/l
Class	Soluble
Log *S* (Ali)	-2.80
Solubility	4.09e-01 mg/ml ; 1.57e-03 mol/l
Class	Soluble
Log *S* (SILICOS-IT)	-3.12
Solubility	1.99e-01 mg/ml ; 7.66e-04 mol/l
Class	Soluble

Pharmacokinetics	
GI absorption	High
BBB permeant	No
P-gp substrate	No
CYP1A2 inhibitor	No
CYP2C19 inhibitor	No
CYP2C9 inhibitor	No
CYP2D6 inhibitor	No
CYP3A4 inhibitor	No
Log K_p (skin permeation)	-7.00 cm/s

Druglikeness	
Lipinski	Yes; 0 violation
Ghose	Yes
Veber	Yes
Egan	Yes
Muegge	Yes
Bioavailability Score	0.55

Medicinal Chemistry	
PAINS	0 alert
Brenk	2 alerts: imine_1, imine_2
Leadlikeness	Yes
Synthetic accessibility	2.27

Compound 17

Molecule 1

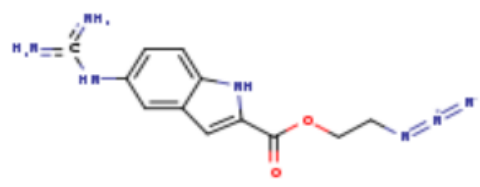

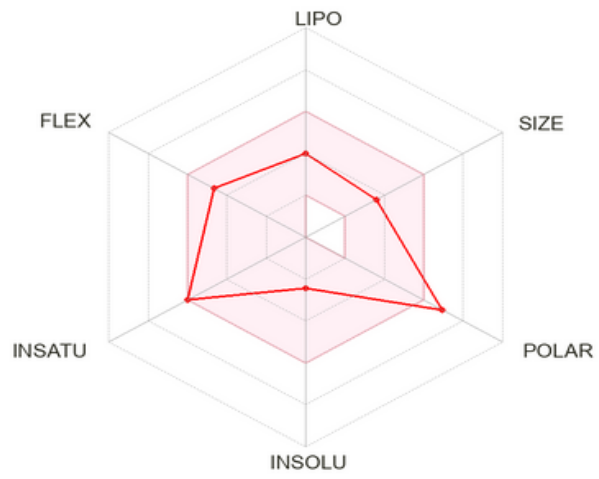

SMILES [N-]=[N+]=NCCOC(=O)c1cc2c([nH]1)ccc(c2)N[C+](N)N

Physicochemical Properties	
Formula	C12H14N7O2+
Molecular weight	288.29 g/mol
Num. heavy atoms	21
Num. arom. heavy atoms	9
Fraction Csp3	0.25
Num. rotatable bonds	7
Num. H-bond acceptors	7
Num. H-bond donors	4
Molar Refractivity	73.42
TPSA	155.91 Å²

Lipophilicity	
Log $P_{o/w}$ (iLOGP)	0.00
Log $P_{o/w}$ (XLOGP3)	1.50
Log $P_{o/w}$ (WLOGP)	1.22
Log $P_{o/w}$ (MLOGP)	-0.36
Log $P_{o/w}$ (SILICOS-IT)	-0.76
Consensus Log $P_{o/w}$	0.32

Water Solubility	
Log *S* (ESOL)	-2.43
Solubility	1.08e+00 mg/ml ; 3.74e-03 mol/l
Class	Soluble
Log *S* (Ali)	-4.38
Solubility	1.20e-02 mg/ml ; 4.15e-05 mol/l
Class	Moderately soluble
Log *S* (SILICOS-IT)	-3.30
Solubility	1.43e-01 mg/ml ; 4.98e-04 mol/l
Class	Soluble

Pharmacokinetics	
GI absorption	Low
BBB permeant	No
P-gp substrate	No
CYP1A2 inhibitor	No
CYP2C19 inhibitor	No
CYP2C9 inhibitor	No
CYP2D6 inhibitor	No
CYP3A4 inhibitor	No
Log K_p (skin permeation)	-6.99 cm/s

Druglikeness	
Lipinski	Yes; 0 violation
Ghose	Yes
Veber	No; 1 violation: TPSA>140
Egan	No; 1 violation: TPSA>131.6
Muegge	No; 1 violation: TPSA>150
Bioavailability Score	0.55

Medicinal Chemistry	
PAINS	1 alert: azo_A
Brenk	4 alerts: azido_group, carbo_cation_anion, diazo_group, quaternary_nitrogen_3
Leadlikeness	Yes
Synthetic accessibility	2.46

Molecule 1

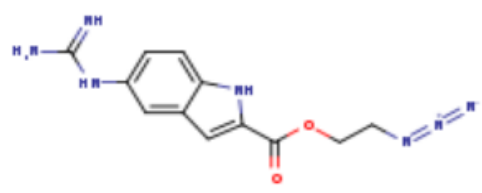

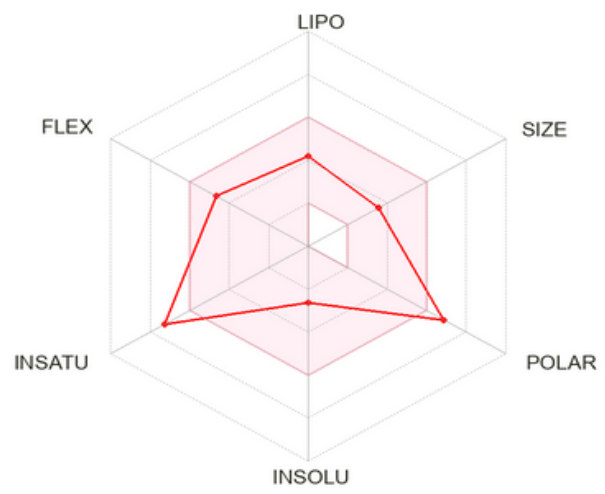

SMILES [N-]=[N+]=NCCOC(=O)c1cc2c([nH]1)ccc(c2)NC(=N)N

Physicochemical Properties	
Formula	C12H13N7O2
Molecular weight	287.28 g/mol
Num. heavy atoms	21
Num. arom. heavy atoms	9
Fraction Csp3	0.17
Num. rotatable bonds	7
Num. H-bond acceptors	6
Num. H-bond donors	4
Molar Refractivity	74.81
TPSA	153.74 Å²

Lipophilicity	
Log $P_{o/w}$ (iLOGP)	1.69
Log $P_{o/w}$ (XLOGP3)	1.83
Log $P_{o/w}$ (WLOGP)	1.75
Log $P_{o/w}$ (MLOGP)	-0.03
Log $P_{o/w}$ (SILICOS-IT)	-0.06
Consensus Log $P_{o/w}$	1.04

Water Solubility	
Log *S* (ESOL)	-2.63
Solubility	6.75e-01 mg/ml ; 2.35e-03 mol/l
Class	Soluble
Log *S* (Ali)	-4.68
Solubility	6.02e-03 mg/ml ; 2.10e-05 mol/l
Class	Moderately soluble
Log *S* (SILICOS-IT)	-3.71
Solubility	5.63e-02 mg/ml ; 1.96e-04 mol/l
Class	Soluble

Pharmacokinetics	
GI absorption	Low
BBB permeant	No
P-gp substrate	No
CYP1A2 inhibitor	No
CYP2C19 inhibitor	No
CYP2C9 inhibitor	No
CYP2D6 inhibitor	No
CYP3A4 inhibitor	No
Log K_p (skin permeation)	-6.75 cm/s

Druglikeness	
Lipinski	Yes; 0 violation
Ghose	Yes
Veber	No; 1 violation: TPSA>140
Egan	No; 1 violation: TPSA>131.6
Muegge	No; 1 violation: TPSA>150
Bioavailability Score	0.55

Medicinal Chemistry	
PAINS	1 alert: azo_A
Brenk	5 alerts: azido_group, diazo_group, imine_1, imine_2, quaternary_nitrogen_3
Leadlikeness	Yes
Synthetic accessibility	2.49

Compound 18

Molecule 1

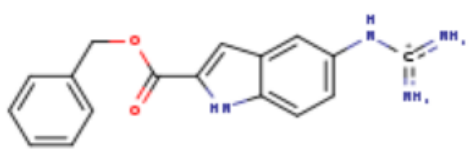

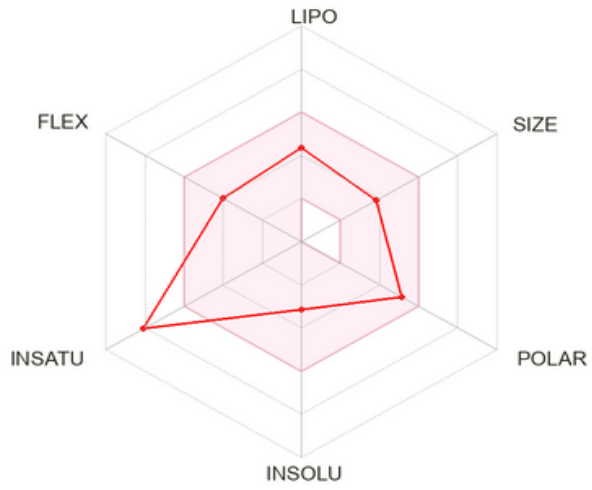

SMILES N[C+](Nc1ccc2c(c1)cc([nH]2)C(=O)OCc1ccccc1)N

Physicochemical Properties	
Formula	C17H17N4O2+
Molecular weight	309.34 g/mol
Num. heavy atoms	23
Num. arom. heavy atoms	15
Fraction Csp3	0.12
Num. rotatable bonds	6
Num. H-bond acceptors	4
Num. H-bond donors	4
Molar Refractivity	88.28
TPSA	106.16 Å^2

Lipophilicity	
Log $P_{o/w}$ (iLOGP)	0.00
Log $P_{o/w}$ (XLOGP3)	2.09
Log $P_{o/w}$ (WLOGP)	1.96
Log $P_{o/w}$ (MLOGP)	1.59
Log $P_{o/w}$ (SILICOS-IT)	1.69
Consensus Log $P_{o/w}$	1.47

Water Solubility	
Log *S* (ESOL)	-3.16
Solubility	2.13e-01 mg/ml ; 6.90e-04 mol/l
Class	Soluble
Log *S* (Ali)	-3.95
Solubility	3.48e-02 mg/ml ; 1.12e-04 mol/l
Class	Soluble
Log *S* (SILICOS-IT)	-5.26
Solubility	1.70e-03 mg/ml ; 5.48e-06 mol/l
Class	Moderately soluble

Pharmacokinetics	
GI absorption	High
BBB permeant	No
P-gp substrate	Yes
CYP1A2 inhibitor	Yes
CYP2C19 inhibitor	No
CYP2C9 inhibitor	No
CYP2D6 inhibitor	Yes
CYP3A4 inhibitor	No
Log K_p (skin permeation)	-6.70 cm/s

Druglikeness	
Lipinski	Yes; 0 violation
Ghose	Yes
Veber	Yes
Egan	Yes
Muegge	Yes
Bioavailability Score	0.55

Medicinal Chemistry	
PAINS	0 alert
Brenk	1 alert: carbo_cation_anion
Leadlikeness	Yes
Synthetic accessibility	2.41

Molecule 1

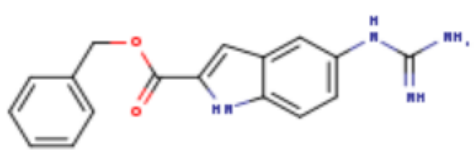

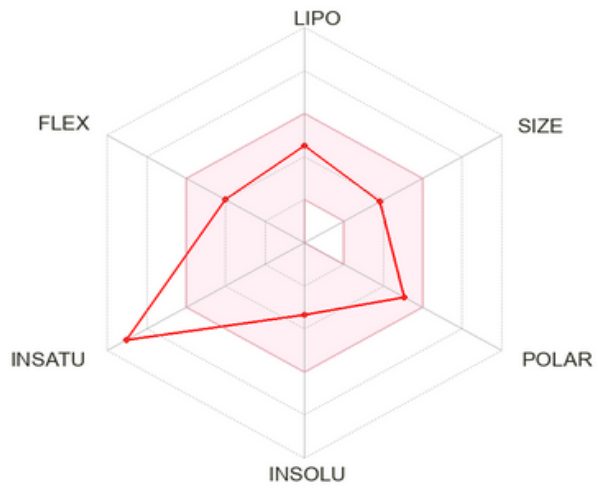

SMILES NC(=N)Nc1ccc2c(c1)cc([nH]2)C(=O)OCc1ccccc1

Physicochemical Properties	
Formula	C17H16N4O2
Molecular weight	308.33 g/mol
Num. heavy atoms	23
Num. arom. heavy atoms	15
Fraction Csp3	0.06
Num. rotatable bonds	6
Num. H-bond acceptors	3
Num. H-bond donors	4
Molar Refractivity	89.67
TPSA	103.99 Å^2

Lipophilicity	
Log $P_{o/w}$ (iLOGP)	1.76
Log $P_{o/w}$ (XLOGP3)	2.42
Log $P_{o/w}$ (WLOGP)	2.49
Log $P_{o/w}$ (MLOGP)	1.92
Log $P_{o/w}$ (SILICOS-IT)	2.38
Consensus Log $P_{o/w}$	2.20

Water Solubility	
Log *S* (ESOL)	-3.36
Solubility	1.34e-01 mg/ml ; 4.34e-04 mol/l
Class	Soluble
Log *S* (Ali)	-4.25
Solubility	1.75e-02 mg/ml ; 5.67e-05 mol/l
Class	Moderately soluble
Log *S* (SILICOS-IT)	-5.67
Solubility	6.65e-04 mg/ml ; 2.16e-06 mol/l
Class	Moderately soluble

Pharmacokinetics	
GI absorption	High
BBB permeant	No
P-gp substrate	No
CYP1A2 inhibitor	Yes
CYP2C19 inhibitor	No
CYP2C9 inhibitor	No
CYP2D6 inhibitor	No
CYP3A4 inhibitor	No
Log K_p (skin permeation)	-6.46 cm/s

Druglikeness	
Lipinski	Yes; 0 violation
Ghose	Yes
Veber	Yes
Egan	Yes
Muegge	Yes
Bioavailability Score	0.55

Medicinal Chemistry	
PAINS	0 alert
Brenk	2 alerts: imine_1, imine_2
Leadlikeness	Yes
Synthetic accessibility	2.47

Compound 19

Molecule 1

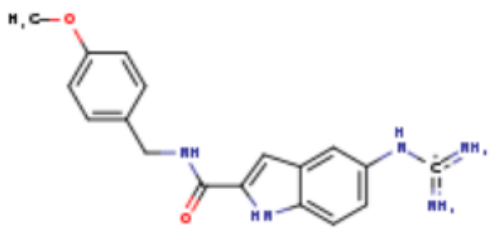

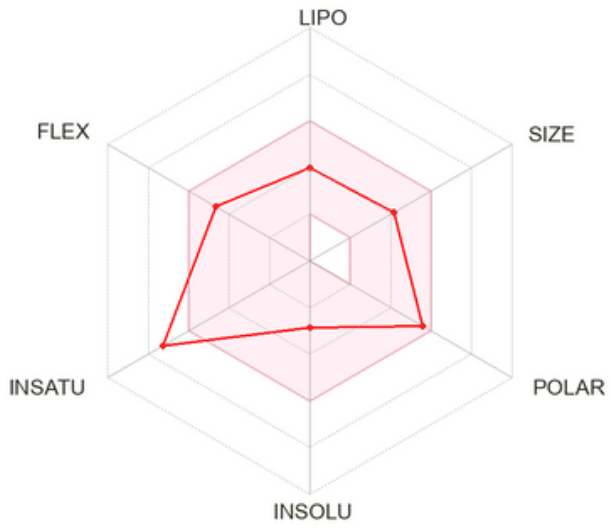

SMILES COc1ccc(cc1)CNC(=O)c1cc2c([nH]1)ccc(c2)N[C+](N)N

Physicochemical Properties	
Formula	C18H20N5O2+
Molecular weight	338.38 g/mol
Num. heavy atoms	25
Num. arom. heavy atoms	15
Fraction Csp3	0.17
Num. rotatable bonds	7
Num. H-bond acceptors	4
Num. H-bond donors	5
Molar Refractivity	96.49
TPSA	118.19 Å²

Lipophilicity	
Log $P_{o/w}$ (iLOGP)	0.00
Log $P_{o/w}$ (XLOGP3)	1.49
Log $P_{o/w}$ (WLOGP)	1.54
Log $P_{o/w}$ (MLOGP)	0.89
Log $P_{o/w}$ (SILICOS-IT)	1.43
Consensus Log $P_{o/w}$	1.07

Water Solubility	
Log *S* (ESOL)	-2.86
Solubility	4.69e-01 mg/ml ; 1.38e-03 mol/l
Class	Soluble
Log *S* (Ali)	-3.58
Solubility	8.91e-02 mg/ml ; 2.63e-04 mol/l
Class	Soluble
Log *S* (SILICOS-IT)	-5.68
Solubility	7.02e-04 mg/ml ; 2.07e-06 mol/l
Class	Moderately soluble

Pharmacokinetics	
GI absorption	High
BBB permeant	No
P-gp substrate	Yes
CYP1A2 inhibitor	Yes
CYP2C19 inhibitor	No
CYP2C9 inhibitor	No
CYP2D6 inhibitor	Yes
CYP3A4 inhibitor	No
Log K_p (skin permeation)	-7.31 cm/s

Druglikeness	
Lipinski	Yes; 0 violation
Ghose	Yes
Veber	Yes
Egan	Yes
Muegge	Yes
Bioavailability Score	0.55

Medicinal Chemistry	
PAINS	0 alert
Brenk	1 alert: carbo_cation_anion
Leadlikeness	Yes
Synthetic accessibility	2.24

Molecule 1

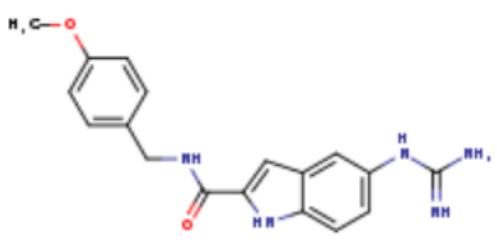

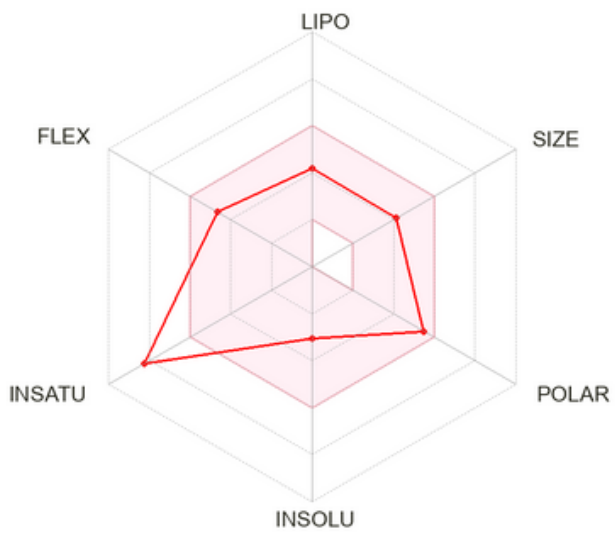

SMILES COc1ccc(cc1)CNC(=O)c1cc2c([nH]1)ccc(c2)NC(=N)N

Physicochemical Properties	
Formula	C18H19N5O2
Molecular weight	337.38 g/mol
Num. heavy atoms	25
Num. arom. heavy atoms	15
Fraction Csp3	0.11
Num. rotatable bonds	7
Num. H-bond acceptors	3
Num. H-bond donors	5
Molar Refractivity	97.88
TPSA	116.02 Å²

Lipophilicity	
Log $P_{o/w}$ (iLOGP)	1.65
Log $P_{o/w}$ (XLOGP3)	1.82
Log $P_{o/w}$ (WLOGP)	2.07
Log $P_{o/w}$ (MLOGP)	1.22
Log $P_{o/w}$ (SILICOS-IT)	2.12
Consensus Log $P_{o/w}$	1.78

Water Solubility	
Log *S* (ESOL)	-3.06
Solubility	2.94e-01 mg/ml ; 8.70e-04 mol/l
Class	Soluble
Log *S* (Ali)	-3.88
Solubility	4.49e-02 mg/ml ; 1.33e-04 mol/l
Class	Soluble
Log *S* (SILICOS-IT)	-6.09
Solubility	2.75e-04 mg/ml ; 8.17e-07 mol/l
Class	Poorly soluble

Pharmacokinetics	
GI absorption	High
BBB permeant	No
P-gp substrate	Yes
CYP1A2 inhibitor	Yes
CYP2C19 inhibitor	No
CYP2C9 inhibitor	No
CYP2D6 inhibitor	Yes
CYP3A4 inhibitor	No
Log K_p (skin permeation)	-7.07 cm/s

Druglikeness	
Lipinski	Yes; 0 violation
Ghose	Yes
Veber	Yes
Egan	Yes
Muegge	Yes
Bioavailability Score	0.55

Medicinal Chemistry	
PAINS	0 alert
Brenk	2 alerts: imine_1, imine_2
Leadlikeness	Yes
Synthetic accessibility	2.31

Compound 20

Molecule 1

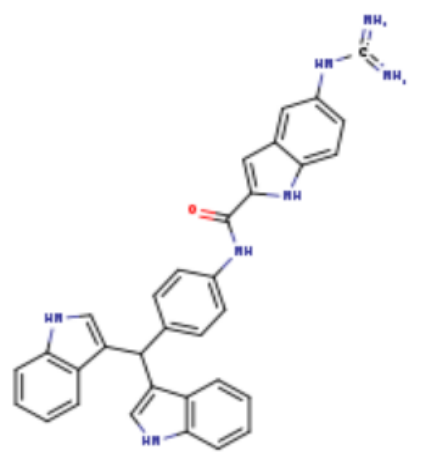

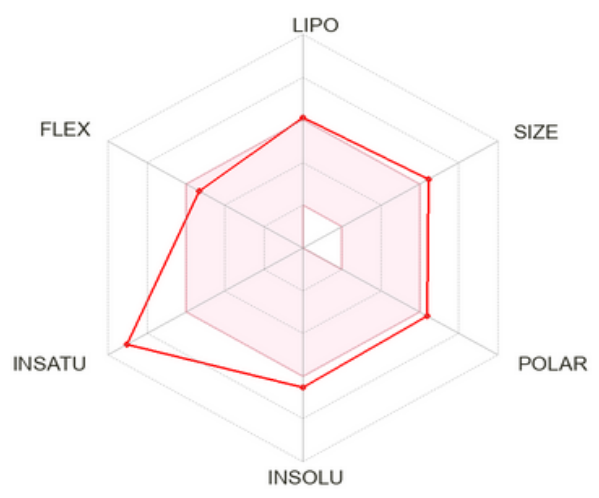

SMILES N[C+](Nc1ccc2c(c1)cc([nH]2)C(=O)Nc1ccc(cc1)C(c1c[nH]c2c1cccc2)c1c[nH]c2c1cccc2)N

Physicochemical Properties	
Formula	C33H28N7O+
Molecular weight	538.62 g/mol
Num. heavy atoms	41
Num. arom. heavy atoms	33
Fraction Csp3	0.06
Num. rotatable bonds	8
Num. H-bond acceptors	3
Num. H-bond donors	7
Molar Refractivity	164.38
TPSA	140.54 Å²

Lipophilicity	
Log $P_{o/w}$ (iLOGP)	0.00
Log $P_{o/w}$ (XLOGP3)	5.21
Log $P_{o/w}$ (WLOGP)	5.96
Log $P_{o/w}$ (MLOGP)	3.04
Log $P_{o/w}$ (SILICOS-IT)	5.28
Consensus Log $P_{o/w}$	3.90

Water Solubility	
Log *S* (ESOL)	-6.53
Solubility	1.59e-04 mg/ml ; 2.96e-07 mol/l
Class	Poorly soluble
Log *S* (Ali)	-7.91
Solubility	6.64e-06 mg/ml ; 1.23e-08 mol/l
Class	Poorly soluble
Log *S* (SILICOS-IT)	-11.81
Solubility	8.30e-10 mg/ml ; 1.54e-12 mol/l
Class	Insoluble

Pharmacokinetics	
GI absorption	Low
BBB permeant	No
P-gp substrate	No
CYP1A2 inhibitor	Yes
CYP2C19 inhibitor	Yes
CYP2C9 inhibitor	No
CYP2D6 inhibitor	No
CYP3A4 inhibitor	No
Log K_p (skin permeation)	-5.89 cm/s

Druglikeness	
Lipinski	No; 2 violations: MW>500, NHorOH>5
Ghose	No; 3 violations: MW>480, WLOGP>5.6, MR>130
Veber	No; 1 violation: TPSA>140
Egan	No; 2 violations: WLOGP>5.88, TPSA>131.6
Muegge	No; 2 violations: XLOGP3>5, H-don>5
Bioavailability Score	0.17

Medicinal Chemistry	
PAINS	0 alert
Brenk	1 alert: carbo_cation_anion
Leadlikeness	No; 3 violations: MW>350, Rotors>7, XLOGP3>3.5
Synthetic accessibility	3.49

Molecule 1

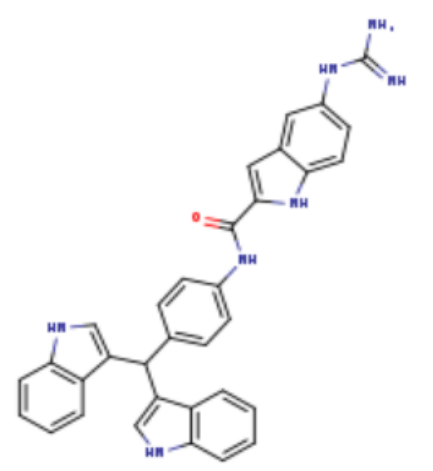

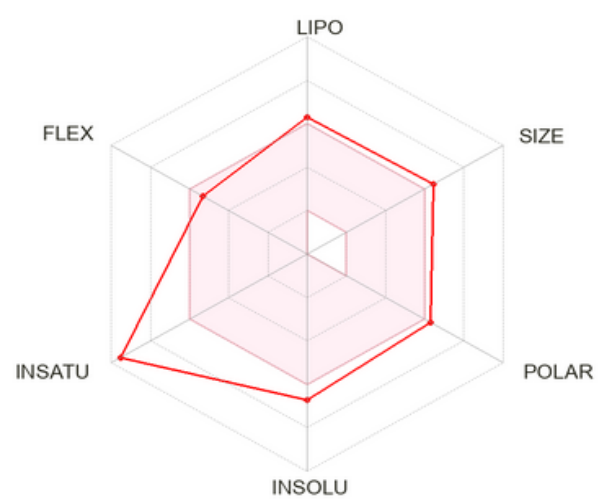

SMILES NC(=N)Nc1ccc2c(c1)cc([nH]2)C(=O)Nc1ccc(cc1)C(c1c[nH]c2c1cccc2)c1c[nH]c2c1cccc2

Physicochemical Properties	
Formula	C33H27N7O
Molecular weight	537.61 g/mol
Num. heavy atoms	41
Num. arom. heavy atoms	33
Fraction Csp3	0.03
Num. rotatable bonds	8
Num. H-bond acceptors	2
Num. H-bond donors	7
Molar Refractivity	165.77
TPSA	138.37 Å²

Lipophilicity	
Log $P_{o/w}$ (iLOGP)	2.03
Log $P_{o/w}$ (XLOGP3)	5.54
Log $P_{o/w}$ (WLOGP)	6.49
Log $P_{o/w}$ (MLOGP)	3.38
Log $P_{o/w}$ (SILICOS-IT)	5.97
Consensus Log $P_{o/w}$	4.68

Water Solubility	
Log *S* (ESOL)	-6.73
Solubility	9.99e-05 mg/ml ; 1.86e-07 mol/l
Class	Poorly soluble
Log *S* (Ali)	-8.21
Solubility	3.35e-06 mg/ml ; 6.23e-09 mol/l
Class	Poorly soluble
Log *S* (SILICOS-IT)	-12.22
Solubility	3.25e-10 mg/ml ; 6.04e-13 mol/l
Class	Insoluble

Pharmacokinetics	
GI absorption	Low
BBB permeant	No
P-gp substrate	No
CYP1A2 inhibitor	Yes
CYP2C19 inhibitor	Yes
CYP2C9 inhibitor	No
CYP2D6 inhibitor	No
CYP3A4 inhibitor	No
Log K_p (skin permeation)	-5.65 cm/s

Druglikeness	
Lipinski	No; 2 violations: MW>500, NHorOH>5
Ghose	No; 3 violations: MW>480, WLOGP>5.6, MR>130
Veber	Yes
Egan	No; 2 violations: WLOGP>5.88, TPSA>131.6
Muegge	No; 2 violations: XLOGP3>5, H-don>5
Bioavailability Score	0.17

Medicinal Chemistry	
PAINS	0 alert
Brenk	2 alerts: imine_1, imine_2
Leadlikeness	No; 3 violations: MW>350, Rotors>7, XLOGP3>3.5
Synthetic accessibility	3.53

Compound 21

Molecule 1

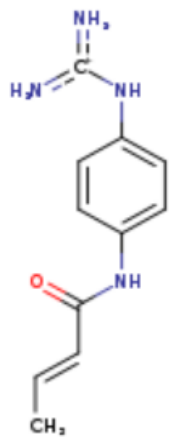

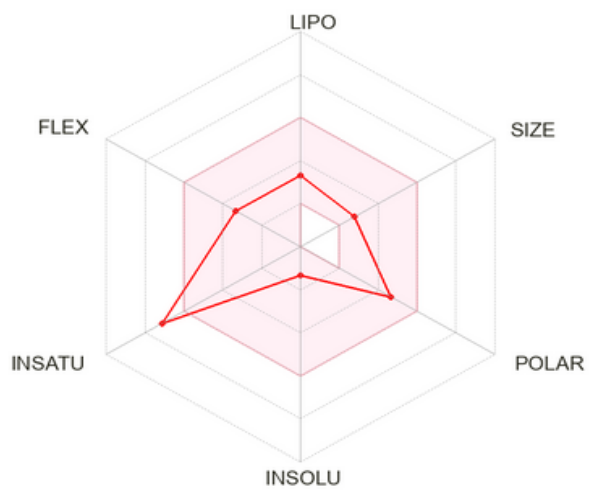

SMILES C/C=C/C(=O)Nc1ccc(cc1)N[C+](N)N

Physicochemical Properties	
Formula	C11H15N4O+
Molecular weight	219.26 g/mol
Num. heavy atoms	16
Num. arom. heavy atoms	6
Fraction Csp3	0.18
Num. rotatable bonds	5
Num. H-bond acceptors	3
Num. H-bond donors	4
Molar Refractivity	64.11
TPSA	93.17 Å²

Lipophilicity	
Log $P_{o/w}$ (iLOGP)	0.00
Log $P_{o/w}$ (XLOGP3)	0.28
Log $P_{o/w}$ (WLOGP)	0.60
Log $P_{o/w}$ (MLOGP)	0.87
Log $P_{o/w}$ (SILICOS-IT)	-0.22
Consensus Log $P_{o/w}$	0.31

Water Solubility	
Log *S* (ESOL)	-1.32
Solubility	1.04e+01 mg/ml ; 4.75e-02 mol/l
Class	Very soluble
Log *S* (Ali)	-1.80
Solubility	3.49e+00 mg/ml ; 1.59e-02 mol/l
Class	Very soluble
Log *S* (SILICOS-IT)	-2.27
Solubility	1.19e+00 mg/ml ; 5.43e-03 mol/l
Class	Soluble

Pharmacokinetics	
GI absorption	High
BBB permeant	No
P-gp substrate	No
CYP1A2 inhibitor	No
CYP2C19 inhibitor	No
CYP2C9 inhibitor	No
CYP2D6 inhibitor	No
CYP3A4 inhibitor	No
Log K_p (skin permeation)	-7.44 cm/s

Druglikeness	
Lipinski	Yes; 0 violation
Ghose	Yes
Veber	Yes
Egan	Yes
Muegge	Yes
Bioavailability Score	0.55

Medicinal Chemistry	
PAINS	0 alert
Brenk	2 alerts: carbo_cation_anion, michael_acceptor_1
Leadlikeness	No; 1 violation: MW<250
Synthetic accessibility	1.94

Molecule 1

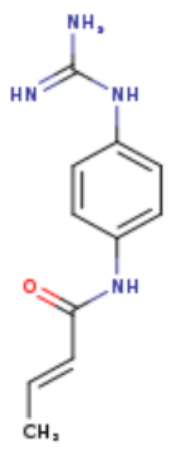

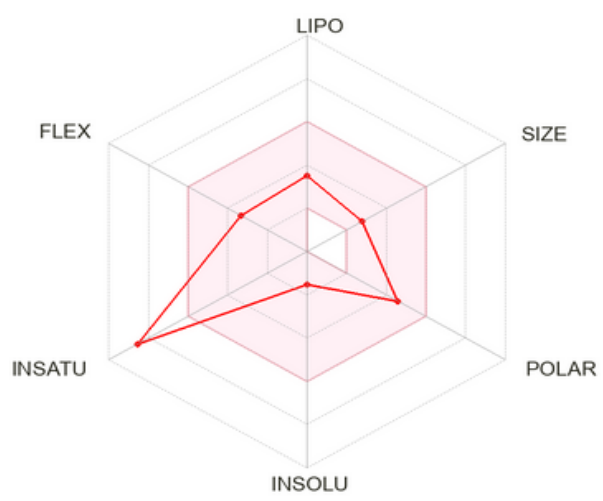

SMILES C/C=C/C(=O)Nc1ccc(cc1)NC(=N)N

Physicochemical Properties	
Formula	C11H14N4O
Molecular weight	218.26 g/mol
Num. heavy atoms	16
Num. arom. heavy atoms	6
Fraction Csp3	0.09
Num. rotatable bonds	5
Num. H-bond acceptors	2
Num. H-bond donors	4
Molar Refractivity	65.50
TPSA	91.00 Å²

Lipophilicity	
Log $P_{o/w}$ (iLOGP)	1.29
Log $P_{o/w}$ (XLOGP3)	0.61
Log $P_{o/w}$ (WLOGP)	1.12
Log $P_{o/w}$ (MLOGP)	1.20
Log $P_{o/w}$ (SILICOS-IT)	0.47
Consensus Log $P_{o/w}$	0.94

Water Solubility	
Log *S* (ESOL)	-1.52
Solubility	6.52e+00 mg/ml ; 2.99e-02 mol/l
Class	Very soluble
Log *S* (Ali)	-2.10
Solubility	1.75e+00 mg/ml ; 8.03e-03 mol/l
Class	Soluble
Log *S* (SILICOS-IT)	-2.67
Solubility	4.69e-01 mg/ml ; 2.15e-03 mol/l
Class	Soluble

Pharmacokinetics	
GI absorption	High
BBB permeant	No
P-gp substrate	No
CYP1A2 inhibitor	No
CYP2C19 inhibitor	No
CYP2C9 inhibitor	No
CYP2D6 inhibitor	No
CYP3A4 inhibitor	No
Log K_p (skin permeation)	-7.20 cm/s

Druglikeness	
Lipinski	Yes; 0 violation
Ghose	Yes
Veber	Yes
Egan	Yes
Muegge	Yes
Bioavailability Score	0.55

Medicinal Chemistry	
PAINS	0 alert
Brenk	3 alerts: imine_1, imine_2, michael_acceptor_1
Leadlikeness	No; 1 violation: MW<250
Synthetic accessibility	2.08

Compound 22

Molecule 1

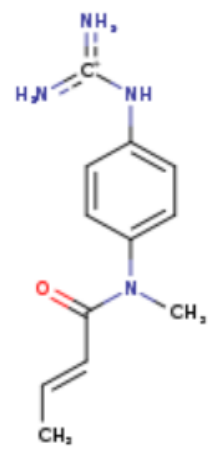

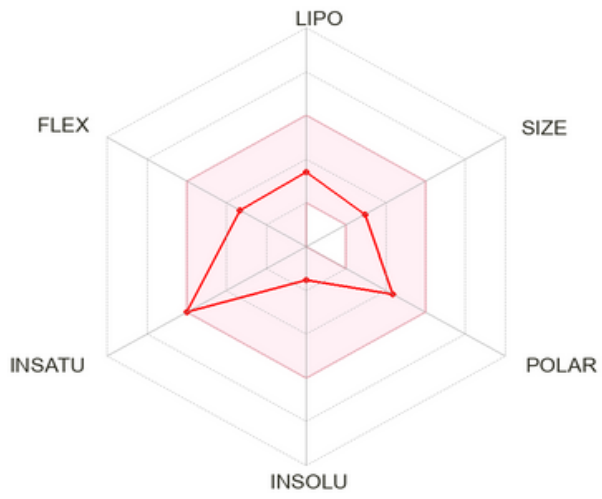

SMILES C/C=C/C(=O)N(c1ccc(cc1)N[C+](N)N)C

Physicochemical Properties	
Formula	C12H17N4O+
Molecular weight	233.29 g/mol
Num. heavy atoms	17
Num. arom. heavy atoms	6
Fraction Csp3	0.25
Num. rotatable bonds	5
Num. H-bond acceptors	3
Num. H-bond donors	3
Molar Refractivity	69.01
TPSA	84.38 Å²
**Lipophilicity**	
Log $P_{o/w}$ (iLOGP)	0.00
Log $P_{o/w}$ (XLOGP3)	0.47
Log $P_{o/w}$ (WLOGP)	0.81
Log $P_{o/w}$ (MLOGP)	1.15
Log $P_{o/w}$ (SILICOS-IT)	-0.28
Consensus Log $P_{o/w}$	0.43

Water Solubility	
Log *S* (ESOL)	-1.51
Solubility	7.15e+00 mg/ml ; 3.06e-02 mol/l
Class	Very soluble
Log *S* (Ali)	-1.81
Solubility	3.61e+00 mg/ml ; 1.55e-02 mol/l
Class	Very soluble
Log *S* (SILICOS-IT)	-1.94
Solubility	2.70e+00 mg/ml ; 1.16e-02 mol/l
Class	Soluble
**Pharmacokinetics**	
GI absorption	High
BBB permeant	No
P-gp substrate	No
CYP1A2 inhibitor	No
CYP2C19 inhibitor	No
CYP2C9 inhibitor	No
CYP2D6 inhibitor	No
CYP3A4 inhibitor	No
Log K_p (skin permeation)	-7.39 cm/s
**Druglikeness**	
Lipinski	Yes; 0 violation
Ghose	Yes
Veber	Yes
Egan	Yes
Muegge	Yes
Bioavailability Score	0.55
**Medicinal Chemistry**	
PAINS	0 alert
Brenk	2 alerts: carbo_cation_anion, michael_acceptor_1
Leadlikeness	No; 1 violation: MW<250
Synthetic accessibility	2.04

Molecule 1

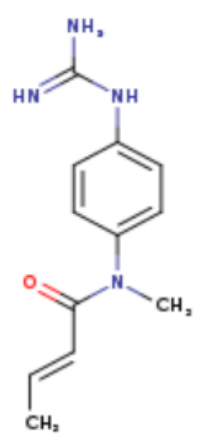

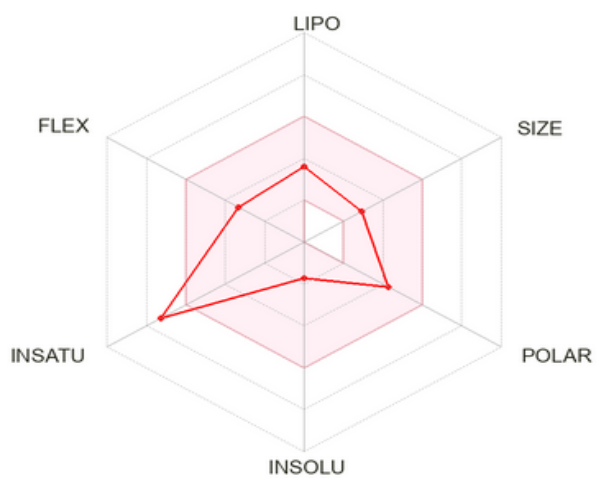

SMILES C/C=C/C(=O)N(c1ccc(cc1)NC(=N)N)C

Physicochemical Properties	
Formula	C12H16N4O
Molecular weight	232.28 g/mol
Num. heavy atoms	17
Num. arom. heavy atoms	6
Fraction Csp3	0.17
Num. rotatable bonds	5
Num. H-bond acceptors	2
Num. H-bond donors	3
Molar Refractivity	70.40
TPSA	82.21 Å²
**Lipophilicity**	
Log $P_{o/w}$ (iLOGP)	1.69
Log $P_{o/w}$ (XLOGP3)	0.80
Log $P_{o/w}$ (WLOGP)	1.34
Log $P_{o/w}$ (MLOGP)	1.48
Log $P_{o/w}$ (SILICOS-IT)	0.41
Consensus Log $P_{o/w}$	1.14

Water Solubility	
Log *S* (ESOL)	-1.72
Solubility	4.47e+00 mg/ml ; 1.93e-02 mol/l
Class	Very soluble
Log *S* (Ali)	-2.11
Solubility	1.81e+00 mg/ml ; 7.80e-03 mol/l
Class	Soluble
Log *S* (SILICOS-IT)	-2.34
Solubility	1.06e+00 mg/ml ; 4.57e-03 mol/l
Class	Soluble
**Pharmacokinetics**	
GI absorption	High
BBB permeant	No
P-gp substrate	No
CYP1A2 inhibitor	No
CYP2C19 inhibitor	No
CYP2C9 inhibitor	No
CYP2D6 inhibitor	No
CYP3A4 inhibitor	No
Log K_p (skin permeation)	-7.15 cm/s
**Druglikeness**	
Lipinski	Yes; 0 violation
Ghose	Yes
Veber	Yes
Egan	Yes
Muegge	Yes
Bioavailability Score	0.55
**Medicinal Chemistry**	
PAINS	0 alert
Brenk	3 alerts: imine_1, imine_2, michael_acceptor_1
Leadlikeness	No; 1 violation: MW<250
Synthetic accessibility	2.18

Compound 23

Molecule 1

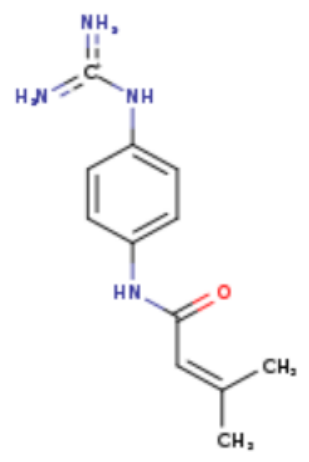

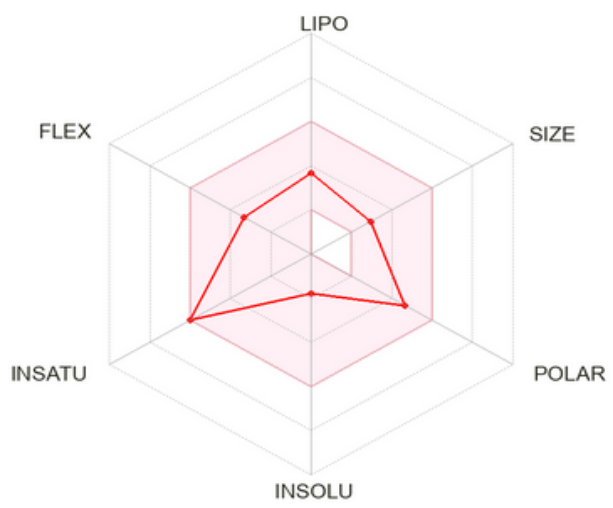

SMILES O=C(Nc1ccc(cc1)N[C+](N)N)C=C(C)C

Physicochemical Properties	
Formula	C12H17N4O+
Molecular weight	233.29 g/mol
Num. heavy atoms	17
Num. arom. heavy atoms	6
Fraction Csp3	0.25
Num. rotatable bonds	5
Num. H-bond acceptors	3
Num. H-bond donors	4
Molar Refractivity	68.92
TPSA	93.17 Å²

Lipophilicity	
Log $P_{o/w}$ (iLOGP)	0.00
Log $P_{o/w}$ (XLOGP3)	0.91
Log $P_{o/w}$ (WLOGP)	0.99
Log $P_{o/w}$ (MLOGP)	1.15
Log $P_{o/w}$ (SILICOS-IT)	0.01
Consensus Log $P_{o/w}$	0.61

Water Solubility	
Log *S* (ESOL)	-1.79
Solubility	3.78e+00 mg/ml ; 1.62e-02 mol/l
Class	Very soluble
Log *S* (Ali)	-2.45
Solubility	8.24e-01 mg/ml ; 3.53e-03 mol/l
Class	Soluble
Log *S* (SILICOS-IT)	-2.65
Solubility	5.20e-01 mg/ml ; 2.23e-03 mol/l
Class	Soluble

Pharmacokinetics	
GI absorption	High
BBB permeant	No
P-gp substrate	No
CYP1A2 inhibitor	No
CYP2C19 inhibitor	No
CYP2C9 inhibitor	No
CYP2D6 inhibitor	No
CYP3A4 inhibitor	No
Log K_p (skin permeation)	-7.08 cm/s

Druglikeness	
Lipinski	Yes; 0 violation
Ghose	Yes
Veber	Yes
Egan	Yes
Muegge	Yes
Bioavailability Score	0.55

Medicinal Chemistry	
PAINS	0 alert
Brenk	2 alerts: carbo_cation_anion, michael_acceptor_1
Leadlikeness	No; 1 violation: MW<250
Synthetic accessibility	2.01

Molecule 1

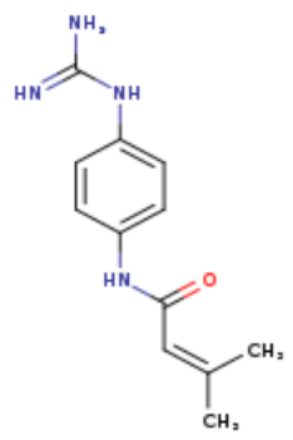

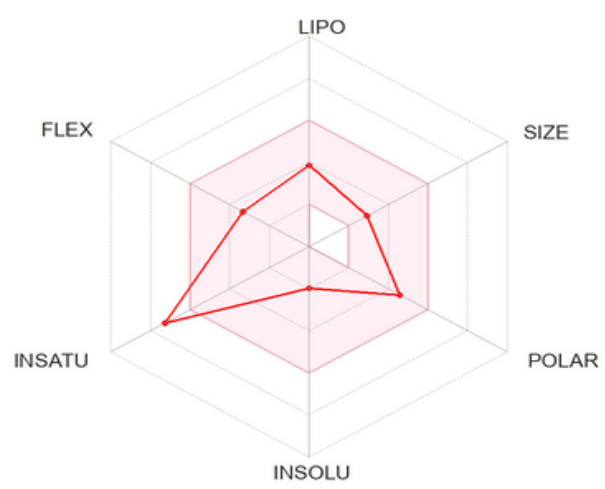

SMILES NC(=N)Nc1ccc(cc1)NC(=O)C=C(C)C

Physicochemical Properties	
Formula	C12H16N4O
Molecular weight	232.28 g/mol
Num. heavy atoms	17
Num. arom. heavy atoms	6
Fraction Csp3	0.17
Num. rotatable bonds	5
Num. H-bond acceptors	2
Num. H-bond donors	4
Molar Refractivity	70.31
TPSA	91.00 Å²

Lipophilicity	
Log $P_{o/w}$ (iLOGP)	1.35
Log $P_{o/w}$ (XLOGP3)	1.24
Log $P_{o/w}$ (WLOGP)	1.52
Log $P_{o/w}$ (MLOGP)	1.48
Log $P_{o/w}$ (SILICOS-IT)	0.70
Consensus Log $P_{o/w}$	1.26

Water Solubility	
Log *S* (ESOL)	-1.99
Solubility	2.36e+00 mg/ml ; 1.02e-02 mol/l
Class	Very soluble
Log *S* (Ali)	-2.75
Solubility	4.14e-01 mg/ml ; 1.78e-03 mol/l
Class	Soluble
Log *S* (SILICOS-IT)	-3.06
Solubility	2.04e-01 mg/ml ; 8.79e-04 mol/l
Class	Soluble

Pharmacokinetics	
GI absorption	High
BBB permeant	No
P-gp substrate	No
CYP1A2 inhibitor	No
CYP2C19 inhibitor	No
CYP2C9 inhibitor	No
CYP2D6 inhibitor	No
CYP3A4 inhibitor	No
Log K_p (skin permeation)	-6.84 cm/s

Druglikeness	
Lipinski	Yes; 0 violation
Ghose	Yes
Veber	Yes
Egan	Yes
Muegge	Yes
Bioavailability Score	0.55

Medicinal Chemistry	
PAINS	0 alert
Brenk	3 alerts: imine_1, imine_2, michael_acceptor_1
Leadlikeness	No; 1 violation: MW<250
Synthetic accessibility	2.15

Compound 24

Molecule 1

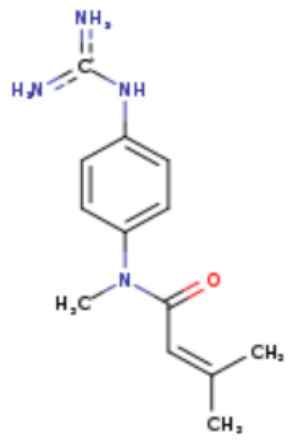

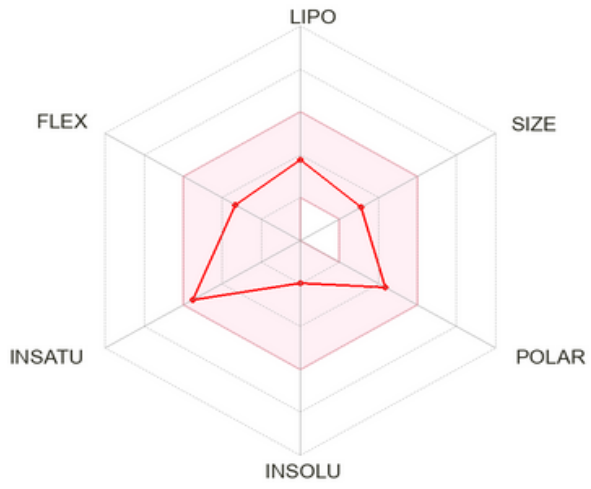

SMILES CN(c1ccc(cc1)N[C+](N)N)C(=O)C=C(C)C

Physicochemical Properties	
Formula	C13H19N4O+
Molecular weight	247.32 g/mol
Num. heavy atoms	18
Num. arom. heavy atoms	6
Fraction Csp3	0.31
Num. rotatable bonds	5
Num. H-bond acceptors	3
Num. H-bond donors	3
Molar Refractivity	73.82
TPSA	84.38 Å²

Lipophilicity	
Log $P_{o/w}$ (iLOGP)	0.00
Log $P_{o/w}$ (XLOGP3)	1.09
Log $P_{o/w}$ (WLOGP)	1.20
Log $P_{o/w}$ (MLOGP)	1.43
Log $P_{o/w}$ (SILICOS-IT)	-0.04
Consensus Log $P_{o/w}$	0.74

Water Solubility	
Log *S* (ESOL)	-1.98
Solubility	2.61e+00 mg/ml ; 1.06e-02 mol/l
Class	Very soluble
Log *S* (Ali)	-2.45
Solubility	8.69e-01 mg/ml ; 3.51e-03 mol/l
Class	Soluble
Log *S* (SILICOS-IT)	-2.32
Solubility	1.18e+00 mg/ml ; 4.76e-03 mol/l
Class	Soluble

Pharmacokinetics	
GI absorption	High
BBB permeant	No
P-gp substrate	No
CYP1A2 inhibitor	No
CYP2C19 inhibitor	No
CYP2C9 inhibitor	No
CYP2D6 inhibitor	No
CYP3A4 inhibitor	No
Log K_p (skin permeation)	-7.03 cm/s

Druglikeness	
Lipinski	Yes; 0 violation
Ghose	Yes
Veber	Yes
Egan	Yes
Muegge	Yes
Bioavailability Score	0.55

Medicinal Chemistry	
PAINS	0 alert
Brenk	2 alerts: carbo_cation_anion, michael_acceptor_1
Leadlikeness	No; 1 violation: MW<250
Synthetic accessibility	2.11

Molecule 1

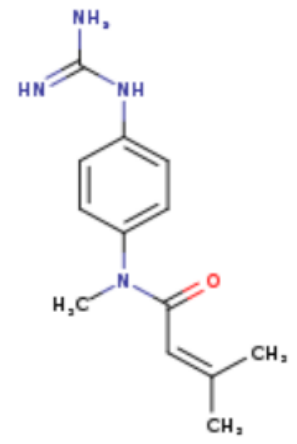

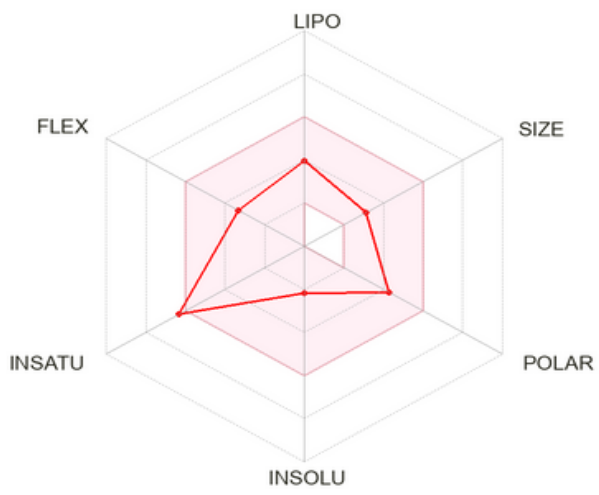

SMILES NC(=N)Nc1ccc(cc1)N(C(=O)C=C(C)C)C

Physicochemical Properties	
Formula	C13H18N4O
Molecular weight	246.31 g/mol
Num. heavy atoms	18
Num. arom. heavy atoms	6
Fraction Csp3	0.23
Num. rotatable bonds	5
Num. H-bond acceptors	2
Num. H-bond donors	3
Molar Refractivity	75.21
TPSA	82.21 Å²

Lipophilicity	
Log $P_{o/w}$ (iLOGP)	1.76
Log $P_{o/w}$ (XLOGP3)	1.42
Log $P_{o/w}$ (WLOGP)	1.73
Log $P_{o/w}$ (MLOGP)	1.75
Log $P_{o/w}$ (SILICOS-IT)	0.65
Consensus Log $P_{o/w}$	1.46

Water Solubility	
Log *S* (ESOL)	-2.18
Solubility	1.63e+00 mg/ml ; 6.63e-03 mol/l
Class	Soluble
Log *S* (Ali)	-2.75
Solubility	4.37e-01 mg/ml ; 1.77e-03 mol/l
Class	Soluble
Log *S* (SILICOS-IT)	-2.73
Solubility	4.63e-01 mg/ml ; 1.88e-03 mol/l
Class	Soluble

Pharmacokinetics	
GI absorption	High
BBB permeant	No
P-gp substrate	No
CYP1A2 inhibitor	No
CYP2C19 inhibitor	No
CYP2C9 inhibitor	No
CYP2D6 inhibitor	No
CYP3A4 inhibitor	No
Log K_p (skin permeation)	-6.79 cm/s

Druglikeness	
Lipinski	Yes; 0 violation
Ghose	Yes
Veber	Yes
Egan	Yes
Muegge	Yes
Bioavailability Score	0.55

Medicinal Chemistry	
PAINS	0 alert
Brenk	3 alerts: imine_1, imine_2, michael_acceptor_1
Leadlikeness	No; 1 violation: MW<250
Synthetic accessibility	2.26

Compound 25

Molecule 1

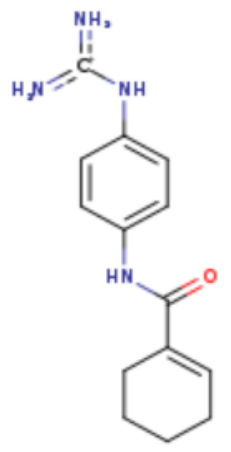

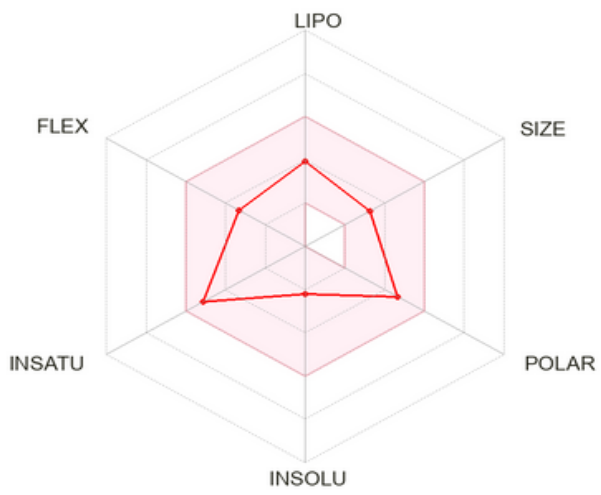

SMILES N[C+](Nc1ccc(cc1)NC(=O)C1=CCCCC1)N

Physicochemical Properties	
Formula	C14H19N4O+
Molecular weight	259.33 g/mol
Num. heavy atoms	19
Num. arom. heavy atoms	6
Fraction Csp3	0.36
Num. rotatable bonds	5
Num. H-bond acceptors	3
Num. H-bond donors	4
Molar Refractivity	76.42
TPSA	93.17 Å²

Lipophilicity	
Log $P_{o/w}$ (iLOGP)	0.00
Log $P_{o/w}$ (XLOGP3)	1.38
Log $P_{o/w}$ (WLOGP)	1.52
Log $P_{o/w}$ (MLOGP)	1.30
Log $P_{o/w}$ (SILICOS-IT)	0.51
Consensus Log $P_{o/w}$	0.94

Water Solubility	
Log *S* (ESOL)	-2.22
Solubility	1.56e+00 mg/ml ; 6.01e-03 mol/l
Class	Soluble
Log *S* (Ali)	-2.94
Solubility	2.98e-01 mg/ml ; 1.15e-03 mol/l
Class	Soluble
Log *S* (SILICOS-IT)	-3.20
Solubility	1.63e-01 mg/ml ; 6.30e-04 mol/l
Class	Soluble

Pharmacokinetics	
GI absorption	High
BBB permeant	No
P-gp substrate	Yes
CYP1A2 inhibitor	No
CYP2C19 inhibitor	No
CYP2C9 inhibitor	No
CYP2D6 inhibitor	No
CYP3A4 inhibitor	No
Log K_p (skin permeation)	-6.90 cm/s

Druglikeness	
Lipinski	Yes; 0 violation
Ghose	Yes
Veber	Yes
Egan	Yes
Muegge	Yes
Bioavailability Score	0.55

Medicinal Chemistry	
PAINS	0 alert
Brenk	1 alert: carbo_cation_anion
Leadlikeness	Yes
Synthetic accessibility	2.50

Molecule 1

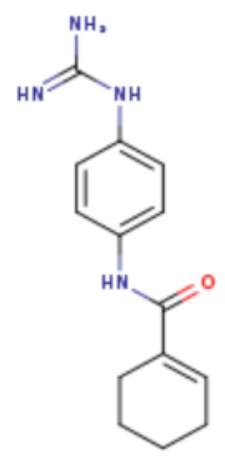

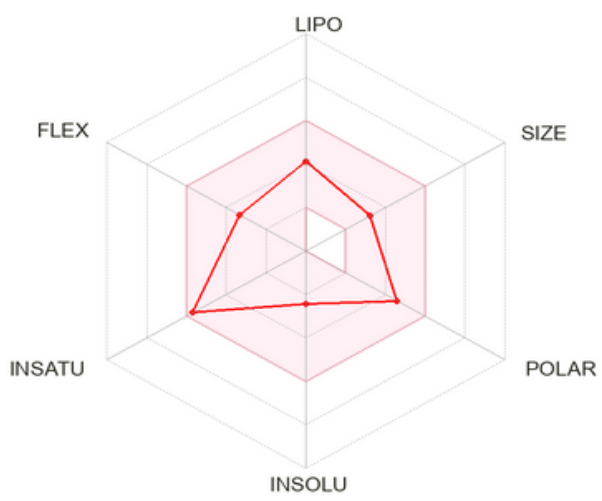

SMILES NC(=N)Nc1ccc(cc1)NC(=O)C1=CCCCC1

Physicochemical Properties	
Formula	C14H18N4O
Molecular weight	258.32 g/mol
Num. heavy atoms	19
Num. arom. heavy atoms	6
Fraction Csp3	0.29
Num. rotatable bonds	5
Num. H-bond acceptors	2
Num. H-bond donors	4
Molar Refractivity	77.81
TPSA	91.00 Å²

Lipophilicity	
Log $P_{o/w}$ (iLOGP)	1.69
Log $P_{o/w}$ (XLOGP3)	1.71
Log $P_{o/w}$ (WLOGP)	2.05
Log $P_{o/w}$ (MLOGP)	1.62
Log $P_{o/w}$ (SILICOS-IT)	1.20
Consensus Log $P_{o/w}$	1.65

Water Solubility	
Log *S* (ESOL)	-2.42
Solubility	9.76e-01 mg/ml ; 3.78e-03 mol/l
Class	Soluble
Log *S* (Ali)	-3.24
Solubility	1.50e-01 mg/ml ; 5.80e-04 mol/l
Class	Soluble
Log *S* (SILICOS-IT)	-3.60
Solubility	6.41e-02 mg/ml ; 2.48e-04 mol/l
Class	Soluble

Pharmacokinetics	
GI absorption	High
BBB permeant	No
P-gp substrate	Yes
CYP1A2 inhibitor	No
CYP2C19 inhibitor	No
CYP2C9 inhibitor	No
CYP2D6 inhibitor	No
CYP3A4 inhibitor	No
Log K_p (skin permeation)	-6.66 cm/s

Druglikeness	
Lipinski	Yes; 0 violation
Ghose	Yes
Veber	Yes
Egan	Yes
Muegge	Yes
Bioavailability Score	0.55

Medicinal Chemistry	
PAINS	0 alert
Brenk	2 alerts: imine_1, imine_2
Leadlikeness	Yes
Synthetic accessibility	2.55

Compound 26

Molecule 1

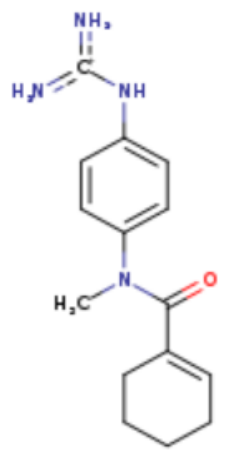

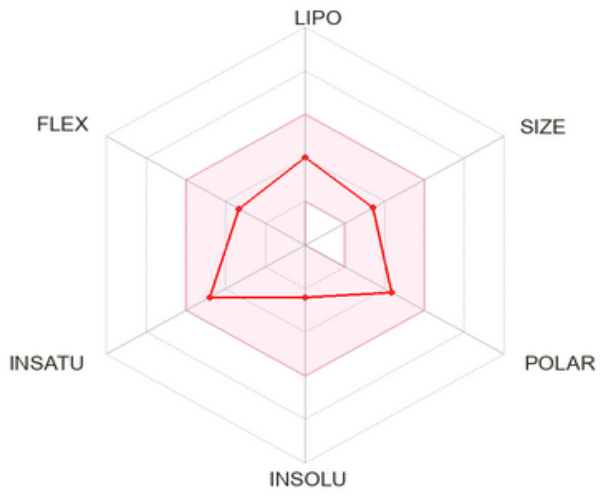

SMILES N[C+](Nc1ccc(cc1)N(C(=O)C1=CCCCC1)C)N

Physicochemical Properties	
Formula	C15H21N4O+
Molecular weight	273.35 g/mol
Num. heavy atoms	20
Num. arom. heavy atoms	6
Fraction Csp3	0.40
Num. rotatable bonds	5
Num. H-bond acceptors	3
Num. H-bond donors	3
Molar Refractivity	81.32
TPSA	84.38 Å²

Lipophilicity	
Log $P_{o/w}$ (iLOGP)	0.00
Log $P_{o/w}$ (XLOGP3)	1.56
Log $P_{o/w}$ (WLOGP)	1.74
Log $P_{o/w}$ (MLOGP)	1.55
Log $P_{o/w}$ (SILICOS-IT)	0.44
Consensus Log $P_{o/w}$	1.06

Water Solubility	
Log *S* (ESOL)	-2.41
Solubility	1.06e+00 mg/ml ; 3.89e-03 mol/l
Class	Soluble
Log *S* (Ali)	-2.94
Solubility	3.12e-01 mg/ml ; 1.14e-03 mol/l
Class	Soluble
Log *S* (SILICOS-IT)	-2.87
Solubility	3.70e-01 mg/ml ; 1.35e-03 mol/l
Class	Soluble

Pharmacokinetics	
GI absorption	High
BBB permeant	No
P-gp substrate	Yes
CYP1A2 inhibitor	No
CYP2C19 inhibitor	No
CYP2C9 inhibitor	No
CYP2D6 inhibitor	No
CYP3A4 inhibitor	No
Log K_p (skin permeation)	-6.86 cm/s

Druglikeness	
Lipinski	Yes; 0 violation
Ghose	Yes
Veber	Yes
Egan	Yes
Muegge	Yes
Bioavailability Score	0.55

Medicinal Chemistry	
PAINS	0 alert
Brenk	1 alert: carbo_cation_anion
Leadlikeness	Yes
Synthetic accessibility	2.61

Molecule 1

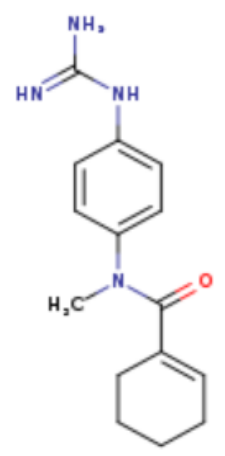

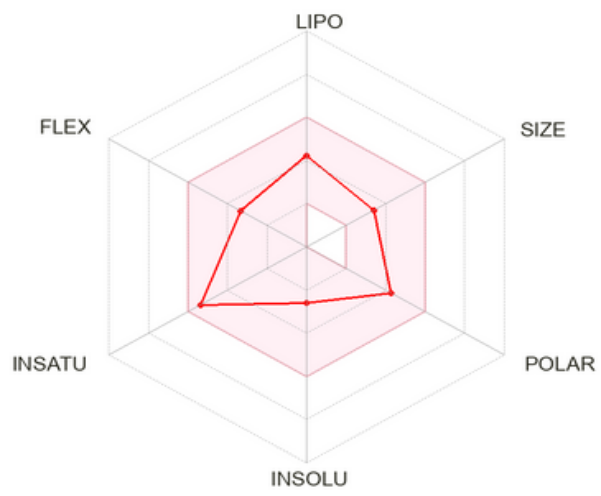

SMILES NC(=N)Nc1ccc(cc1)N(C(=O)C1=CCCCC1)C

Physicochemical Properties	
Formula	C15H20N4O
Molecular weight	272.35 g/mol
Num. heavy atoms	20
Num. arom. heavy atoms	6
Fraction Csp3	0.33
Num. rotatable bonds	5
Num. H-bond acceptors	2
Num. H-bond donors	3
Molar Refractivity	82.71
TPSA	82.21 Å²

Lipophilicity	
Log $P_{o/w}$ (iLOGP)	1.95
Log $P_{o/w}$ (XLOGP3)	1.89
Log $P_{o/w}$ (WLOGP)	2.26
Log $P_{o/w}$ (MLOGP)	1.88
Log $P_{o/w}$ (SILICOS-IT)	1.13
Consensus Log $P_{o/w}$	1.82

Water Solubility	
Log *S* (ESOL)	-2.61
Solubility	6.67e-01 mg/ml ; 2.45e-03 mol/l
Class	Soluble
Log *S* (Ali)	-3.24
Solubility	1.57e-01 mg/ml ; 5.77e-04 mol/l
Class	Soluble
Log *S* (SILICOS-IT)	-3.27
Solubility	1.45e-01 mg/ml ; 5.32e-04 mol/l
Class	Soluble

Pharmacokinetics	
GI absorption	High
BBB permeant	No
P-gp substrate	No
CYP1A2 inhibitor	No
CYP2C19 inhibitor	No
CYP2C9 inhibitor	No
CYP2D6 inhibitor	No
CYP3A4 inhibitor	No
Log K_p (skin permeation)	-6.62 cm/s

Druglikeness	
Lipinski	Yes; 0 violation
Ghose	Yes
Veber	Yes
Egan	Yes
Muegge	Yes
Bioavailability Score	0.55

Medicinal Chemistry	
PAINS	0 alert
Brenk	2 alerts: imine_1, imine_2
Leadlikeness	Yes
Synthetic accessibility	2.66

Compound 27

Molecule 1

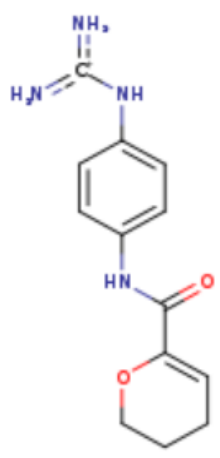

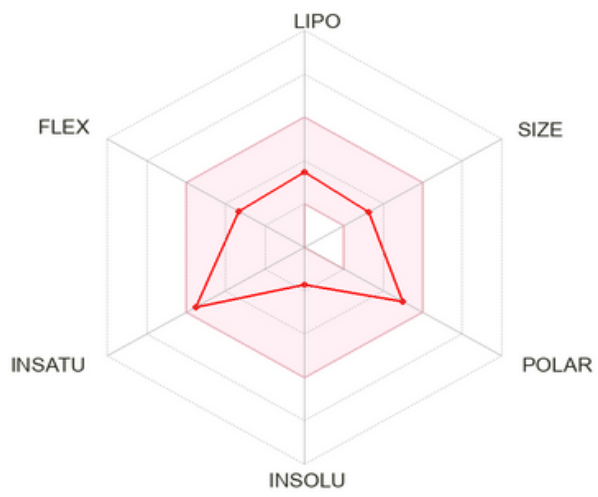

SMILES N[C+](Nc1ccc(cc1)NC(=O)C1=CCCCO1)N

Physicochemical Properties	
Formula	C13H17N4O2+
Molecular weight	261.30 g/mol
Num. heavy atoms	19
Num. arom. heavy atoms	6
Fraction Csp3	0.31
Num. rotatable bonds	5
Num. H-bond acceptors	4
Num. H-bond donors	4
Molar Refractivity	72.69
TPSA	102.40 Å²

Lipophilicity	
Log $P_{o/w}$ (iLOGP)	0.00
Log $P_{o/w}$ (XLOGP3)	0.57
Log $P_{o/w}$ (WLOGP)	0.71
Log $P_{o/w}$ (MLOGP)	0.22
Log $P_{o/w}$ (SILICOS-IT)	-0.11
Consensus Log $P_{o/w}$	0.28

Water Solubility	
Log *S* (ESOL)	-1.72
Solubility	4.95e+00 mg/ml ; 1.89e-02 mol/l
Class	Very soluble
Log *S* (Ali)	-2.29
Solubility	1.33e+00 mg/ml ; 5.09e-03 mol/l
Class	Soluble
Log *S* (SILICOS-IT)	-2.66
Solubility	5.68e-01 mg/ml ; 2.17e-03 mol/l
Class	Soluble

Pharmacokinetics	
GI absorption	High
BBB permeant	No
P-gp substrate	Yes
CYP1A2 inhibitor	No
CYP2C19 inhibitor	No
CYP2C9 inhibitor	No
CYP2D6 inhibitor	No
CYP3A4 inhibitor	No
Log K_p (skin permeation)	-7.49 cm/s

Druglikeness	
Lipinski	Yes; 0 violation
Ghose	Yes
Veber	Yes
Egan	Yes
Muegge	Yes
Bioavailability Score	0.55

Medicinal Chemistry	
PAINS	0 alert
Brenk	1 alert: carbo_cation_anion
Leadlikeness	Yes
Synthetic accessibility	2.92

Molecule 1

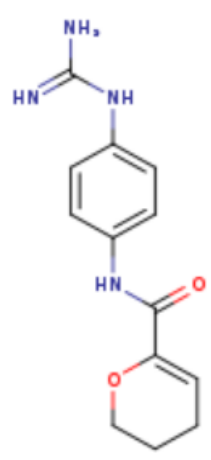

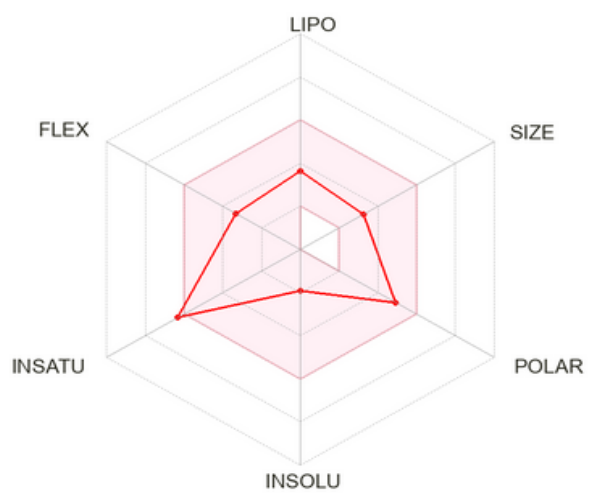

SMILES NC(=N)Nc1ccc(cc1)NC(=O)C1=CCCCO1

Physicochemical Properties	
Formula	C13H16N4O2
Molecular weight	260.29 g/mol
Num. heavy atoms	19
Num. arom. heavy atoms	6
Fraction Csp3	0.23
Num. rotatable bonds	5
Num. H-bond acceptors	3
Num. H-bond donors	4
Molar Refractivity	74.08
TPSA	100.23 Å²

Lipophilicity	
Log $P_{o/w}$ (iLOGP)	1.77
Log $P_{o/w}$ (XLOGP3)	0.89
Log $P_{o/w}$ (WLOGP)	1.24
Log $P_{o/w}$ (MLOGP)	0.54
Log $P_{o/w}$ (SILICOS-IT)	0.58
Consensus Log $P_{o/w}$	1.01

Water Solubility	
Log *S* (ESOL)	-1.92
Solubility	3.14e+00 mg/ml ; 1.21e-02 mol/l
Class	Very soluble
Log *S* (Ali)	-2.58
Solubility	6.85e-01 mg/ml ; 2.63e-03 mol/l
Class	Soluble
Log *S* (SILICOS-IT)	-3.07
Solubility	2.23e-01 mg/ml ; 8.57e-04 mol/l
Class	Soluble

Pharmacokinetics	
GI absorption	High
BBB permeant	No
P-gp substrate	Yes
CYP1A2 inhibitor	No
CYP2C19 inhibitor	No
CYP2C9 inhibitor	No
CYP2D6 inhibitor	No
CYP3A4 inhibitor	No
Log K_p (skin permeation)	-7.26 cm/s

Druglikeness	
Lipinski	Yes; 0 violation
Ghose	Yes
Veber	Yes
Egan	Yes
Muegge	Yes
Bioavailability Score	0.55

Medicinal Chemistry	
PAINS	0 alert
Brenk	2 alerts: imine_1, imine_2
Leadlikeness	Yes
Synthetic accessibility	2.93

Compound 28

Molecule 1

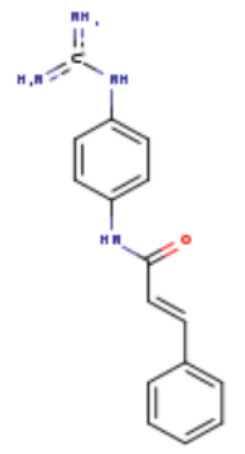

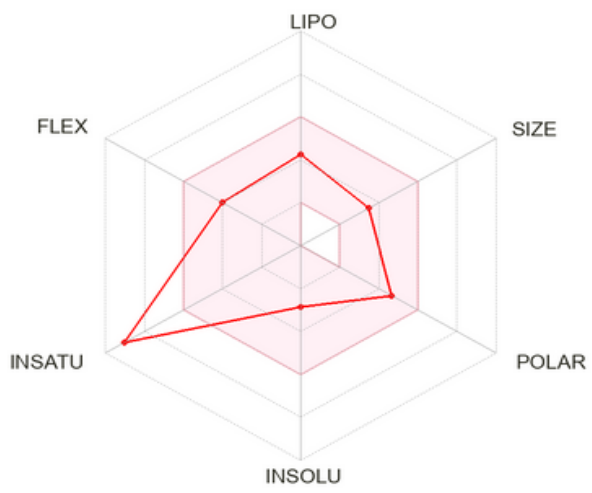

SMILES N[C+](Nc1ccc(cc1)NC(=O)/C=C/c1ccccc1)N

Physicochemical Properties	
Formula	C16H17N4O+
Molecular weight	281.33 g/mol
Num. heavy atoms	21
Num. arom. heavy atoms	12
Fraction Csp3	0.06
Num. rotatable bonds	6
Num. H-bond acceptors	3
Num. H-bond donors	4
Molar Refractivity	84.58
TPSA	93.17 Å²

Lipophilicity	
Log $P_{o/w}$ (iLOGP)	0.00
Log $P_{o/w}$ (XLOGP3)	1.97
Log $P_{o/w}$ (WLOGP)	1.62
Log $P_{o/w}$ (MLOGP)	1.95
Log $P_{o/w}$ (SILICOS-IT)	0.99
Consensus Log $P_{o/w}$	1.31

Water Solubility	
Log *S* (ESOL)	-2.85
Solubility	3.95e-01 mg/ml ; 1.41e-03 mol/l
Class	Soluble
Log *S* (Ali)	-3.55
Solubility	7.89e-02 mg/ml ; 2.81e-04 mol/l
Class	Soluble
Log *S* (SILICOS-IT)	-4.38
Solubility	1.16e-02 mg/ml ; 4.14e-05 mol/l
Class	Moderately soluble

Pharmacokinetics	
GI absorption	High
BBB permeant	No
P-gp substrate	No
CYP1A2 inhibitor	No
CYP2C19 inhibitor	No
CYP2C9 inhibitor	No
CYP2D6 inhibitor	No
CYP3A4 inhibitor	No
Log K_p (skin permeation)	-6.62 cm/s

Druglikeness	
Lipinski	Yes; 0 violation
Ghose	Yes
Veber	Yes
Egan	Yes
Muegge	Yes
Bioavailability Score	0.55

Medicinal Chemistry	
PAINS	0 alert
Brenk	2 alerts: carbo_cation_anion, michael_acceptor_1
Leadlikeness	Yes
Synthetic accessibility	2.42

Molecule 1

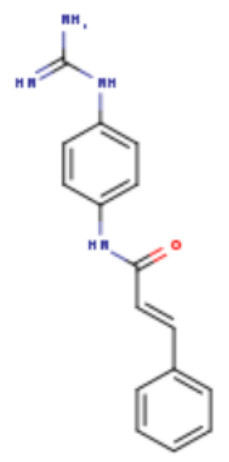

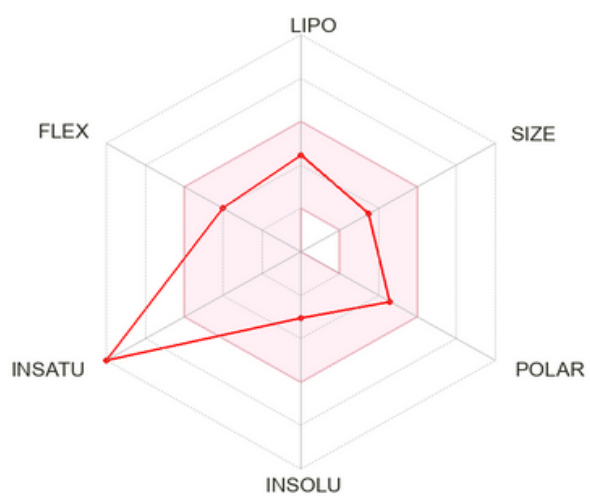

SMILES NC(=N)Nc1ccc(cc1)NC(=O)/C=C/c1ccccc1

Physicochemical Properties	
Formula	C16H16N4O
Molecular weight	280.32 g/mol
Num. heavy atoms	21
Num. arom. heavy atoms	12
Fraction Csp3	0.00
Num. rotatable bonds	6
Num. H-bond acceptors	2
Num. H-bond donors	4
Molar Refractivity	85.97
TPSA	91.00 Å²

Lipophilicity	
Log $P_{o/w}$ (iLOGP)	1.68
Log $P_{o/w}$ (XLOGP3)	2.30
Log $P_{o/w}$ (WLOGP)	2.15
Log $P_{o/w}$ (MLOGP)	2.29
Log $P_{o/w}$ (SILICOS-IT)	1.69
Consensus Log $P_{o/w}$	2.02

Water Solubility	
Log *S* (ESOL)	-3.05
Solubility	2.48e-01 mg/ml ; 8.83e-04 mol/l
Class	Soluble
Log *S* (Ali)	-3.85
Solubility	3.97e-02 mg/ml ; 1.42e-04 mol/l
Class	Soluble
Log *S* (SILICOS-IT)	-4.79
Solubility	4.57e-03 mg/ml ; 1.63e-05 mol/l
Class	Moderately soluble

Pharmacokinetics	
GI absorption	High
BBB permeant	No
P-gp substrate	No
CYP1A2 inhibitor	No
CYP2C19 inhibitor	No
CYP2C9 inhibitor	Yes
CYP2D6 inhibitor	No
CYP3A4 inhibitor	No
Log K_p (skin permeation)	-6.38 cm/s

Druglikeness	
Lipinski	Yes; 0 violation
Ghose	Yes
Veber	Yes
Egan	Yes
Muegge	Yes
Bioavailability Score	0.55

Medicinal Chemistry	
PAINS	0 alert
Brenk	3 alerts: imine_1, imine_2, michael_acceptor_1
Leadlikeness	Yes
Synthetic accessibility	2.50

Compound 29

Molecule 1

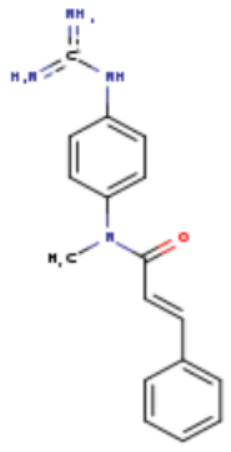

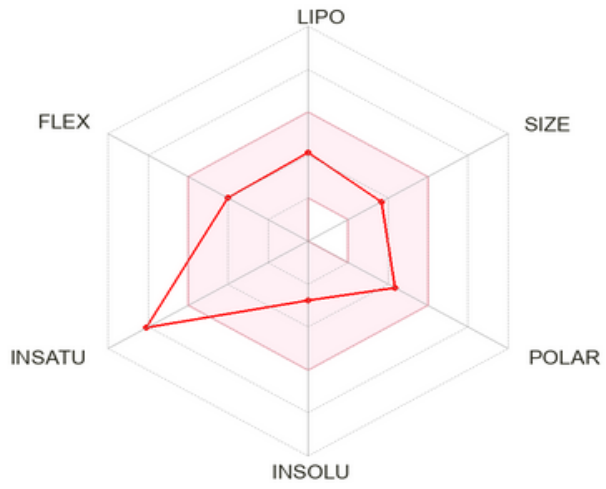

SMILES N[C+](Nc1ccc(cc1)N(C(=O)/C=C/c1ccccc1)C)N

Physicochemical Properties	
Formula	C17H19N4O+
Molecular weight	295.36 g/mol
Num. heavy atoms	22
Num. arom. heavy atoms	12
Fraction Csp3	0.12
Num. rotatable bonds	6
Num. H-bond acceptors	3
Num. H-bond donors	3
Molar Refractivity	89.48
TPSA	84.38 Å²

Lipophilicity	
Log $P_{o/w}$ (iLOGP)	0.00
Log $P_{o/w}$ (XLOGP3)	1.73
Log $P_{o/w}$ (WLOGP)	1.84
Log $P_{o/w}$ (MLOGP)	2.20
Log $P_{o/w}$ (SILICOS-IT)	0.94
Consensus Log $P_{o/w}$	1.34

Water Solubility	
Log *S* (ESOL)	-2.77
Solubility	5.03e-01 mg/ml ; 1.70e-03 mol/l
Class	Soluble
Log *S* (Ali)	-3.12
Solubility	2.25e-01 mg/ml ; 7.61e-04 mol/l
Class	Soluble
Log *S* (SILICOS-IT)	-4.05
Solubility	2.63e-02 mg/ml ; 8.91e-05 mol/l
Class	Moderately soluble

Pharmacokinetics	
GI absorption	High
BBB permeant	No
P-gp substrate	No
CYP1A2 inhibitor	No
CYP2C19 inhibitor	No
CYP2C9 inhibitor	No
CYP2D6 inhibitor	No
CYP3A4 inhibitor	No
Log K_p (skin permeation)	-6.87 cm/s

Druglikeness	
Lipinski	Yes; 0 violation
Ghose	Yes
Veber	Yes
Egan	Yes
Muegge	Yes
Bioavailability Score	0.55

Medicinal Chemistry	
PAINS	0 alert
Brenk	2 alerts: carbo_cation_anion, michael_acceptor_1
Leadlikeness	Yes
Synthetic accessibility	2.52

Molecule 1

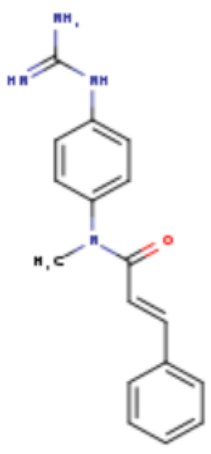

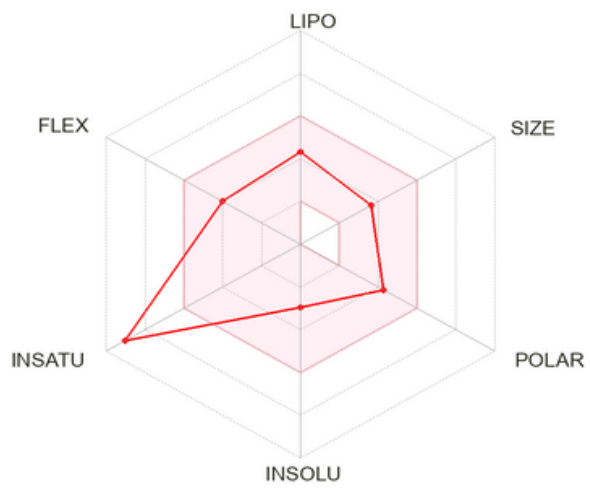

SMILES NC(=N)Nc1ccc(cc1)N(C(=O)/C=C/c1ccccc1)C

Physicochemical Properties	
Formula	C17H18N4O
Molecular weight	294.35 g/mol
Num. heavy atoms	22
Num. arom. heavy atoms	12
Fraction Csp3	0.06
Num. rotatable bonds	6
Num. H-bond acceptors	2
Num. H-bond donors	3
Molar Refractivity	90.87
TPSA	82.21 Å²

Lipophilicity	
Log $P_{o/w}$ (iLOGP)	2.03
Log $P_{o/w}$ (XLOGP3)	2.06
Log $P_{o/w}$ (WLOGP)	2.37
Log $P_{o/w}$ (MLOGP)	2.53
Log $P_{o/w}$ (SILICOS-IT)	1.63
Consensus Log $P_{o/w}$	2.12

Water Solubility	
Log *S* (ESOL)	-2.97
Solubility	3.15e-01 mg/ml ; 1.07e-03 mol/l
Class	Soluble
Log *S* (Ali)	-3.42
Solubility	1.13e-01 mg/ml ; 3.84e-04 mol/l
Class	Soluble
Log *S* (SILICOS-IT)	-4.45
Solubility	1.03e-02 mg/ml ; 3.52e-05 mol/l
Class	Moderately soluble

Pharmacokinetics	
GI absorption	High
BBB permeant	No
P-gp substrate	No
CYP1A2 inhibitor	No
CYP2C19 inhibitor	No
CYP2C9 inhibitor	Yes
CYP2D6 inhibitor	No
CYP3A4 inhibitor	No
Log K_p (skin permeation)	-6.63 cm/s

Druglikeness	
Lipinski	Yes; 0 violation
Ghose	Yes
Veber	Yes
Egan	Yes
Muegge	Yes
Bioavailability Score	0.55

Medicinal Chemistry	
PAINS	0 alert
Brenk	3 alerts: imine_1, imine_2, michael_acceptor_1
Leadlikeness	Yes
Synthetic accessibility	2.60

Compound 30

Molecule 1

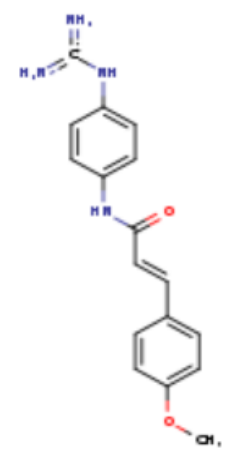

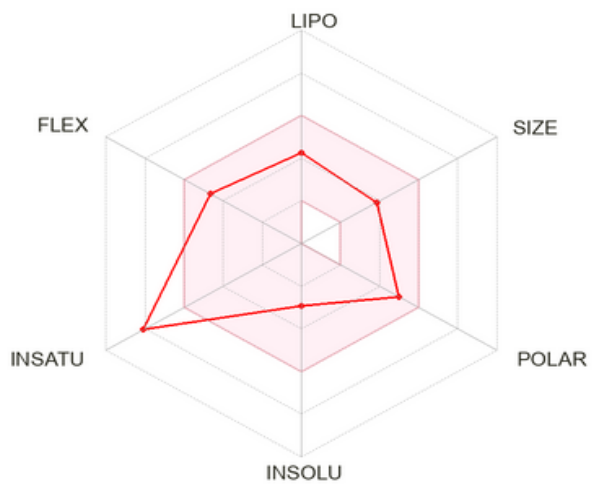

SMILES COc1ccc(cc1)/C=C/C(=O)Nc1ccc(cc1)N[C+](N)N

Physicochemical Properties	
Formula	C17H19N4O2+
Molecular weight	311.36 g/mol
Num. heavy atoms	23
Num. arom. heavy atoms	12
Fraction Csp3	0.12
Num. rotatable bonds	7
Num. H-bond acceptors	4
Num. H-bond donors	4
Molar Refractivity	91.07
TPSA	102.40 Å²

Lipophilicity	
Log $P_{o/w}$ (iLOGP)	0.00
Log $P_{o/w}$ (XLOGP3)	1.94
Log $P_{o/w}$ (WLOGP)	1.63
Log $P_{o/w}$ (MLOGP)	1.65
Log $P_{o/w}$ (SILICOS-IT)	1.06
Consensus Log $P_{o/w}$	1.26

Water Solubility	
Log *S* (ESOL)	-2.92
Solubility	3.77e-01 mg/ml ; 1.21e-03 mol/l
Class	Soluble
Log *S* (Ali)	-3.71
Solubility	6.01e-02 mg/ml ; 1.93e-04 mol/l
Class	Soluble
Log *S* (SILICOS-IT)	-4.50
Solubility	9.89e-03 mg/ml ; 3.18e-05 mol/l
Class	Moderately soluble

Pharmacokinetics	
GI absorption	High
BBB permeant	No
P-gp substrate	No
CYP1A2 inhibitor	No
CYP2C19 inhibitor	No
CYP2C9 inhibitor	No
CYP2D6 inhibitor	Yes
CYP3A4 inhibitor	No
Log K_p (skin permeation)	-6.82 cm/s

Druglikeness	
Lipinski	Yes; 0 violation
Ghose	Yes
Veber	Yes
Egan	Yes
Muegge	Yes
Bioavailability Score	0.55

Medicinal Chemistry	
PAINS	0 alert
Brenk	2 alerts: carbo_cation_anion, michael_acceptor_1
Leadlikeness	Yes
Synthetic accessibility	2.46

Molecule 1

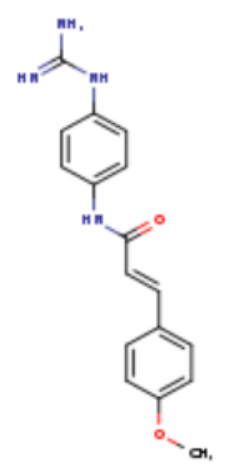

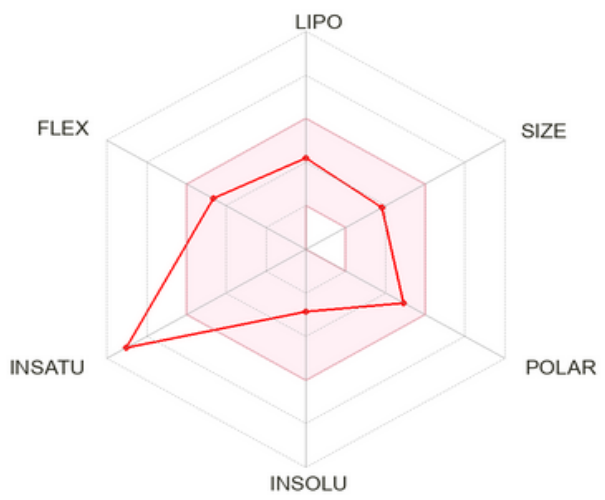

SMILES COc1ccc(cc1)/C=C/C(=O)Nc1ccc(cc1)NC(=N)N

Physicochemical Properties	
Formula	C17H18N4O2
Molecular weight	310.35 g/mol
Num. heavy atoms	23
Num. arom. heavy atoms	12
Fraction Csp3	0.06
Num. rotatable bonds	7
Num. H-bond acceptors	3
Num. H-bond donors	4
Molar Refractivity	92.46
TPSA	100.23 Å²

Lipophilicity	
Log $P_{o/w}$ (iLOGP)	1.98
Log $P_{o/w}$ (XLOGP3)	1.85
Log $P_{o/w}$ (WLOGP)	2.16
Log $P_{o/w}$ (MLOGP)	1.98
Log $P_{o/w}$ (SILICOS-IT)	1.75
Consensus Log $P_{o/w}$	1.95

Water Solubility	
Log *S* (ESOL)	-2.85
Solubility	4.35e-01 mg/ml ; 1.40e-03 mol/l
Class	Soluble
Log *S* (Ali)	-3.58
Solubility	8.24e-02 mg/ml ; 2.66e-04 mol/l
Class	Soluble
Log *S* (SILICOS-IT)	-4.90
Solubility	3.88e-03 mg/ml ; 1.25e-05 mol/l
Class	Moderately soluble

Pharmacokinetics	
GI absorption	High
BBB permeant	No
P-gp substrate	No
CYP1A2 inhibitor	No
CYP2C19 inhibitor	No
CYP2C9 inhibitor	Yes
CYP2D6 inhibitor	No
CYP3A4 inhibitor	No
Log K_p (skin permeation)	-6.88 cm/s

Druglikeness	
Lipinski	Yes; 0 violation
Ghose	Yes
Veber	Yes
Egan	Yes
Muegge	Yes
Bioavailability Score	0.55

Medicinal Chemistry	
PAINS	0 alert
Brenk	3 alerts: imine_1, imine_2, michael_acceptor_1
Leadlikeness	Yes
Synthetic accessibility	2.52

Compound 31

Molecule 1

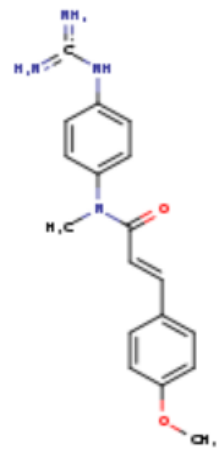

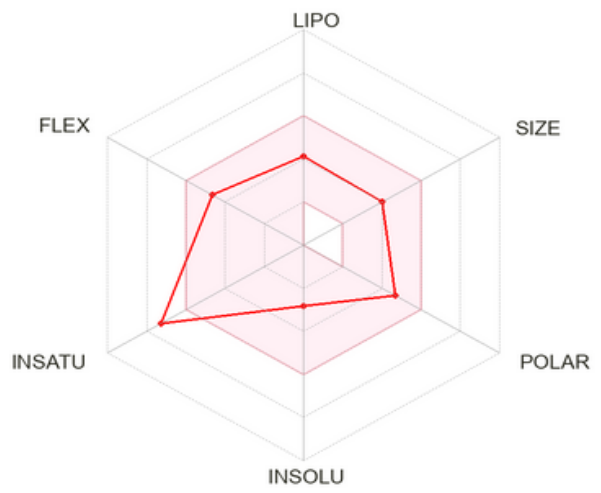

SMILES COc1ccc(cc1)/C=C/C(=O)N(c1ccc(cc1)N[C+](N)N)C

Physicochemical Properties	
Formula	C18H21N4O2+
Molecular weight	325.38 g/mol
Num. heavy atoms	24
Num. arom. heavy atoms	12
Fraction Csp3	0.17
Num. rotatable bonds	7
Num. H-bond acceptors	4
Num. H-bond donors	3
Molar Refractivity	95.97
TPSA	93.61 Å²

Lipophilicity	
Log $P_{o/w}$ (iLOGP)	0.00
Log $P_{o/w}$ (XLOGP3)	1.70
Log $P_{o/w}$ (WLOGP)	1.85
Log $P_{o/w}$ (MLOGP)	1.89
Log $P_{o/w}$ (SILICOS-IT)	1.01
Consensus Log $P_{o/w}$	1.29

Water Solubility	
Log *S* (ESOL)	-2.84
Solubility	4.74e-01 mg/ml ; 1.46e-03 mol/l
Class	Soluble
Log *S* (Ali)	-3.28
Solubility	1.70e-01 mg/ml ; 5.23e-04 mol/l
Class	Soluble
Log *S* (SILICOS-IT)	-4.16
Solubility	2.24e-02 mg/ml ; 6.89e-05 mol/l
Class	Moderately soluble

Pharmacokinetics	
GI absorption	High
BBB permeant	No
P-gp substrate	No
CYP1A2 inhibitor	No
CYP2C19 inhibitor	No
CYP2C9 inhibitor	No
CYP2D6 inhibitor	No
CYP3A4 inhibitor	No
Log K_p (skin permeation)	-7.08 cm/s

Druglikeness	
Lipinski	Yes; 0 violation
Ghose	Yes
Veber	Yes
Egan	Yes
Muegge	Yes
Bioavailability Score	0.55

Medicinal Chemistry	
PAINS	0 alert
Brenk	2 alerts: carbo_cation_anion, michael_acceptor_1
Leadlikeness	Yes
Synthetic accessibility	2.57

Molecule 1

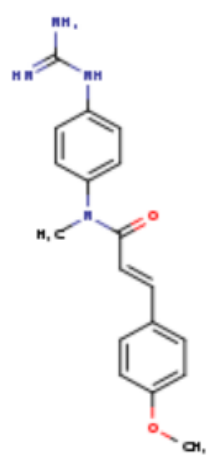

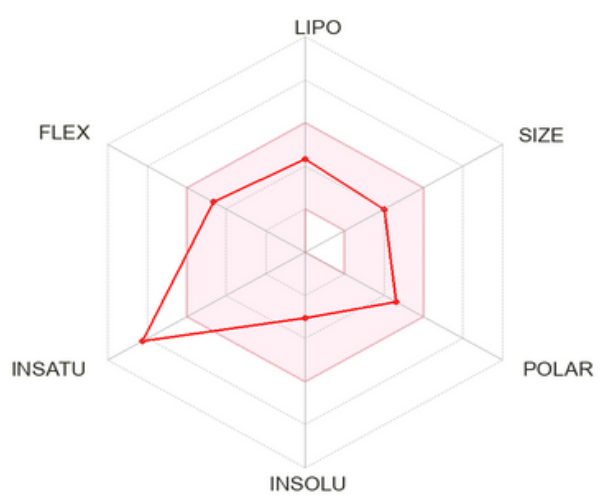

SMILES COc1ccc(cc1)/C=C/C(=O)N(c1ccc(cc1)NC(=N)N)C

Physicochemical Properties	
Formula	C18H20N4O2
Molecular weight	324.38 g/mol
Num. heavy atoms	24
Num. arom. heavy atoms	12
Fraction Csp3	0.11
Num. rotatable bonds	7
Num. H-bond acceptors	3
Num. H-bond donors	3
Molar Refractivity	97.36
TPSA	91.44 Å²

Lipophilicity	
Log $P_{o/w}$ (iLOGP)	2.54
Log $P_{o/w}$ (XLOGP3)	2.03
Log $P_{o/w}$ (WLOGP)	2.38
Log $P_{o/w}$ (MLOGP)	2.22
Log $P_{o/w}$ (SILICOS-IT)	1.70
Consensus Log $P_{o/w}$	2.17

Water Solubility	
Log *S* (ESOL)	-3.04
Solubility	2.97e-01 mg/ml ; 9.16e-04 mol/l
Class	Soluble
Log *S* (Ali)	-3.58
Solubility	8.57e-02 mg/ml ; 2.64e-04 mol/l
Class	Soluble
Log *S* (SILICOS-IT)	-4.57
Solubility	8.79e-03 mg/ml ; 2.71e-05 mol/l
Class	Moderately soluble

Pharmacokinetics	
GI absorption	High
BBB permeant	No
P-gp substrate	No
CYP1A2 inhibitor	No
CYP2C19 inhibitor	No
CYP2C9 inhibitor	Yes
CYP2D6 inhibitor	No
CYP3A4 inhibitor	No
Log K_p (skin permeation)	-6.84 cm/s

Druglikeness	
Lipinski	Yes; 0 violation
Ghose	Yes
Veber	Yes
Egan	Yes
Muegge	Yes
Bioavailability Score	0.55

Medicinal Chemistry	
PAINS	0 alert
Brenk	3 alerts: imine_1, imine_2, michael_acceptor_1
Leadlikeness	Yes
Synthetic accessibility	2.63

Compound 32

Molecule 1

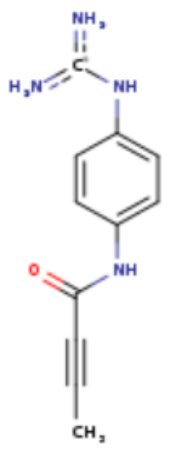

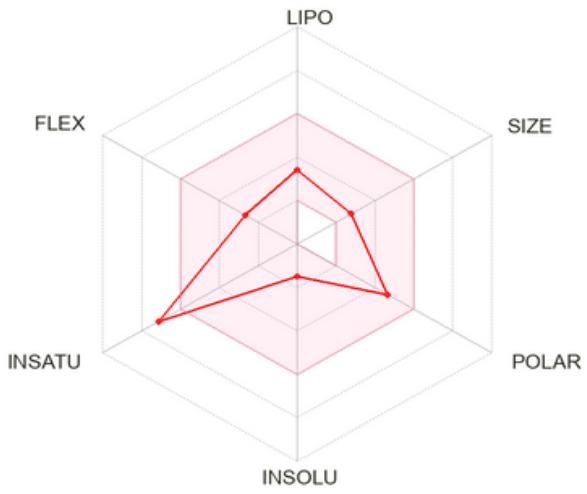

SMILES CC#CC(=O)Nc1ccc(cc1)N[C+](N)N

Physicochemical Properties	
Formula	C11H13N4O+
Molecular weight	217.25 g/mol
Num. heavy atoms	16
Num. arom. heavy atoms	6
Fraction Csp3	0.18
Num. rotatable bonds	4
Num. H-bond acceptors	3
Num. H-bond donors	4
Molar Refractivity	62.74
TPSA	93.17 Å²

Lipophilicity	
Log $P_{o/w}$ (iLOGP)	0.00
Log $P_{o/w}$ (XLOGP3)	0.45
Log $P_{o/w}$ (WLOGP)	0.12
Log $P_{o/w}$ (MLOGP)	0.87
Log $P_{o/w}$ (SILICOS-IT)	-0.36
Consensus Log $P_{o/w}$	0.22

Water Solubility	
Log *S* (ESOL)	-1.48
Solubility	7.13e+00 mg/ml ; 3.28e-02 mol/l
Class	Very soluble
Log *S* (Ali)	-1.97
Solubility	2.30e+00 mg/ml ; 1.06e-02 mol/l
Class	Very soluble
Log *S* (SILICOS-IT)	-2.26
Solubility	1.20e+00 mg/ml ; 5.52e-03 mol/l
Class	Soluble

Pharmacokinetics	
GI absorption	High
BBB permeant	No
P-gp substrate	No
CYP1A2 inhibitor	No
CYP2C19 inhibitor	No
CYP2C9 inhibitor	No
CYP2D6 inhibitor	No
CYP3A4 inhibitor	No
Log K_p (skin permeation)	-7.31 cm/s

Druglikeness	
Lipinski	Yes; 0 violation
Ghose	Yes
Veber	Yes
Egan	Yes
Muegge	Yes
Bioavailability Score	0.55

Medicinal Chemistry	
PAINS	0 alert
Brenk	2 alerts: carbo_cation_anion, triple_bond
Leadlikeness	No; 1 violation: MW<250
Synthetic accessibility	2.20

Molecule 1

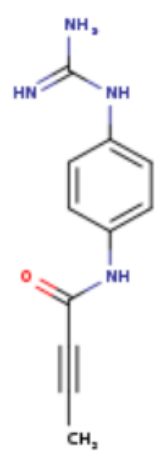

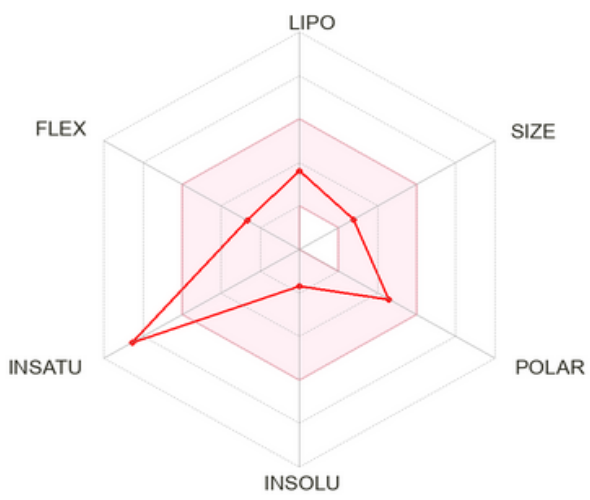

SMILES CC#CC(=O)Nc1ccc(cc1)NC(=N)N

Physicochemical Properties	
Formula	C11H12N4O
Molecular weight	216.24 g/mol
Num. heavy atoms	16
Num. arom. heavy atoms	6
Fraction Csp3	0.09
Num. rotatable bonds	4
Num. H-bond acceptors	2
Num. H-bond donors	4
Molar Refractivity	64.13
TPSA	91.00 Å²

Lipophilicity	
Log $P_{o/w}$ (iLOGP)	1.39
Log $P_{o/w}$ (XLOGP3)	0.78
Log $P_{o/w}$ (WLOGP)	0.65
Log $P_{o/w}$ (MLOGP)	1.20
Log $P_{o/w}$ (SILICOS-IT)	0.33
Consensus Log $P_{o/w}$	0.87

Water Solubility	
Log *S* (ESOL)	-1.69
Solubility	4.46e+00 mg/ml ; 2.06e-02 mol/l
Class	Very soluble
Log *S* (Ali)	-2.27
Solubility	1.16e+00 mg/ml ; 5.35e-03 mol/l
Class	Soluble
Log *S* (SILICOS-IT)	-2.66
Solubility	4.72e-01 mg/ml ; 2.18e-03 mol/l
Class	Soluble

Pharmacokinetics	
GI absorption	High
BBB permeant	No
P-gp substrate	No
CYP1A2 inhibitor	No
CYP2C19 inhibitor	No
CYP2C9 inhibitor	No
CYP2D6 inhibitor	No
CYP3A4 inhibitor	No
Log K_p (skin permeation)	-7.07 cm/s

Druglikeness	
Lipinski	Yes; 0 violation
Ghose	Yes
Veber	Yes
Egan	Yes
Muegge	Yes
Bioavailability Score	0.55

Medicinal Chemistry	
PAINS	0 alert
Brenk	3 alerts: imine_1, imine_2, triple_bond
Leadlikeness	No; 1 violation: MW<250
Synthetic accessibility	2.31

Compound 33

Molecule 1

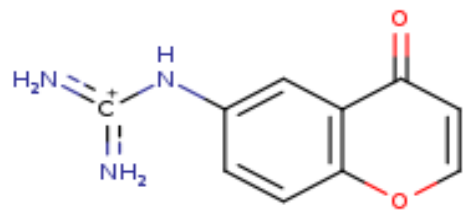

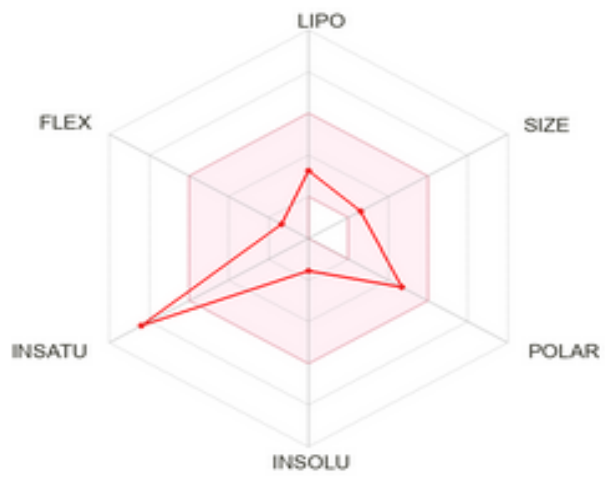

SMILES N[C+](Nc1ccc2c(c1)c(=O)cco2)N

Physicochemical Properties	
Formula	C10H10N3O2+
Molecular weight	204.21 g/mol
Num. heavy atoms	15
Num. arom. heavy atoms	10
Fraction Csp3	0.10
Num. rotatable bonds	2
Num. H-bond acceptors	4
Num. H-bond donors	3
Molar Refractivity	56.70
TPSA	94.28 Å²

Lipophilicity	
Log $P_{o/w}$ (iLOGP)	0.00
Log $P_{o/w}$ (XLOGP3)	0.16
Log $P_{o/w}$ (WLOGP)	0.38
Log $P_{o/w}$ (MLOGP)	-0.34
Log $P_{o/w}$ (SILICOS-IT)	0.22
Consensus Log $P_{o/w}$	0.08

Water Solubility	
Log *S* (ESOL)	-1.57
Solubility	5.52e+00 mg/ml ; 2.70e-02 mol/l
Class	Very soluble
Log *S* (Ali)	-1.70
Solubility	4.10e+00 mg/ml ; 2.01e-02 mol/l
Class	Very soluble
Log *S* (SILICOS-IT)	-3.00
Solubility	2.05e-01 mg/ml ; 1.00e-03 mol/l
Class	Soluble

Pharmacokinetics	
GI absorption	High
BBB permeant	No
P-gp substrate	No
CYP1A2 inhibitor	No
CYP2C19 inhibitor	No
CYP2C9 inhibitor	No
CYP2D6 inhibitor	No
CYP3A4 inhibitor	No
Log K_p (skin permeation)	-7.43 cm/s

Druglikeness	
Lipinski	Yes; 0 violation
Ghose	Yes
Veber	Yes
Egan	Yes
Muegge	Yes
Bioavailability Score	0.55

Medicinal Chemistry	
PAINS	0 alert
Brenk	1 alert: carbo_cation_anion
Leadlikeness	No; 1 violation: MW<250
Synthetic accessibility	2.64

Molecule 1

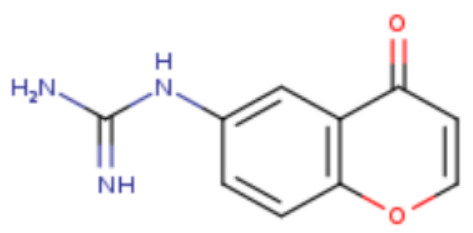

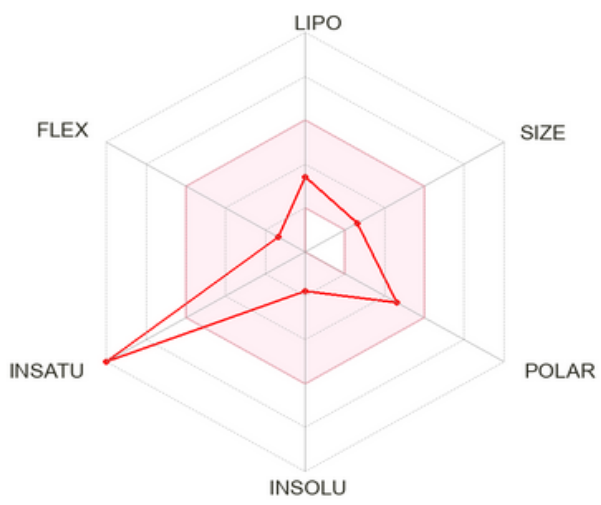

SMILES NC(=N)Nc1ccc2c(c1)c(=O)cco2

Physicochemical Properties	
Formula	C10H9N3O2
Molecular weight	203.20 g/mol
Num. heavy atoms	15
Num. arom. heavy atoms	10
Fraction Csp3	0.00
Num. rotatable bonds	2
Num. H-bond acceptors	3
Num. H-bond donors	3
Molar Refractivity	58.09
TPSA	92.11 Å²

Lipophilicity	
Log $P_{o/w}$ (iLOGP)	1.06
Log $P_{o/w}$ (XLOGP3)	0.49
Log $P_{o/w}$ (WLOGP)	0.91
Log $P_{o/w}$ (MLOGP)	-0.01
Log $P_{o/w}$ (SILICOS-IT)	0.91
Consensus Log $P_{o/w}$	0.67

Water Solubility	
Log *S* (ESOL)	-1.77
Solubility	3.45e+00 mg/ml ; 1.70e-02 mol/l
Class	Very soluble
Log *S* (Ali)	-1.99
Solubility	2.06e+00 mg/ml ; 1.01e-02 mol/l
Class	Very soluble
Log *S* (SILICOS-IT)	-3.40
Solubility	8.03e-02 mg/ml ; 3.95e-04 mol/l
Class	Soluble

Pharmacokinetics	
GI absorption	High
BBB permeant	No
P-gp substrate	No
CYP1A2 inhibitor	No
CYP2C19 inhibitor	No
CYP2C9 inhibitor	No
CYP2D6 inhibitor	No
CYP3A4 inhibitor	No
Log K_p (skin permeation)	-7.19 cm/s

Druglikeness	
Lipinski	Yes; 0 violation
Ghose	Yes
Veber	Yes
Egan	Yes
Muegge	Yes
Bioavailability Score	0.55

Medicinal Chemistry	
PAINS	0 alert
Brenk	2 alerts: imine_1, imine_2
Leadlikeness	No; 1 violation: MW<250
Synthetic accessibility	2.68

Compound 34

Molecule 1

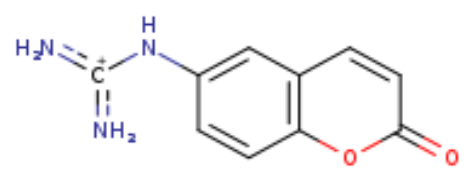

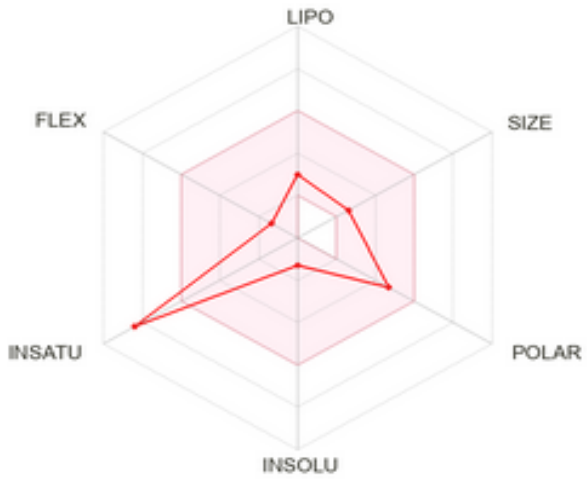

SMILES N[C+](Nc1ccc2c(c1)ccc(=O)o2)N

Physicochemical Properties	
Formula	C10H10N3O2+
Molecular weight	204.21 g/mol
Num. heavy atoms	15
Num. arom. heavy atoms	10
Fraction Csp3	0.10
Num. rotatable bonds	2
Num. H-bond acceptors	4
Num. H-bond donors	3
Molar Refractivity	56.70
TPSA	94.28 Å²

Lipophilicity	
Log $P_{o/w}$ (iLOGP)	0.00
Log $P_{o/w}$ (XLOGP3)	-0.25
Log $P_{o/w}$ (WLOGP)	0.38
Log $P_{o/w}$ (MLOGP)	0.47
Log $P_{o/w}$ (SILICOS-IT)	0.22
Consensus Log $P_{o/w}$	0.16

Water Solubility	
Log *S* (ESOL)	-1.31
Solubility	1.00e+01 mg/ml ; 4.90e-02 mol/l
Class	Very soluble
Log *S* (Ali)	-1.27
Solubility	1.09e+01 mg/ml ; 5.35e-02 mol/l
Class	Very soluble
Log *S* (SILICOS-IT)	-3.00
Solubility	2.05e-01 mg/ml ; 1.00e-03 mol/l
Class	Soluble

Pharmacokinetics	
GI absorption	High
BBB permeant	No
P-gp substrate	No
CYP1A2 inhibitor	No
CYP2C19 inhibitor	No
CYP2C9 inhibitor	No
CYP2D6 inhibitor	No
CYP3A4 inhibitor	No
Log K_p (skin permeation)	-7.72 cm/s

Druglikeness	
Lipinski	Yes; 0 violation
Ghose	Yes
Veber	Yes
Egan	Yes
Muegge	Yes
Bioavailability Score	0.55

Medicinal Chemistry	
PAINS	0 alert
Brenk	2 alerts: carbo_cation_anion, cumarine
Leadlikeness	No; 1 violation: MW<250
Synthetic accessibility	2.69

Molecule 1

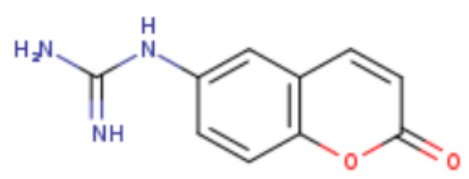

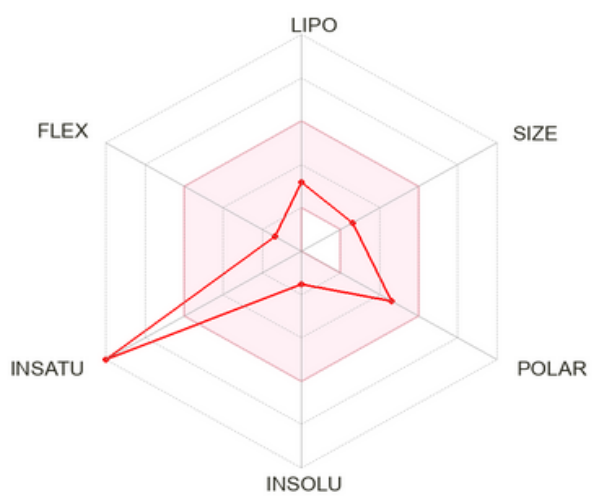

SMILES NC(=N)Nc1ccc2c(c1)ccc(=O)o2

Physicochemical Properties	
Formula	C10H9N3O2
Molecular weight	203.20 g/mol
Num. heavy atoms	15
Num. arom. heavy atoms	10
Fraction Csp3	0.00
Num. rotatable bonds	2
Num. H-bond acceptors	3
Num. H-bond donors	3
Molar Refractivity	58.09
TPSA	92.11 Å²

Lipophilicity	
Log $P_{o/w}$ (iLOGP)	0.87
Log $P_{o/w}$ (XLOGP3)	0.08
Log $P_{o/w}$ (WLOGP)	0.91
Log $P_{o/w}$ (MLOGP)	0.80
Log $P_{o/w}$ (SILICOS-IT)	0.91
Consensus Log $P_{o/w}$	0.71

Water Solubility	
Log *S* (ESOL)	-1.51
Solubility	6.26e+00 mg/ml ; 3.08e-02 mol/l
Class	Very soluble
Log *S* (Ali)	-1.57
Solubility	5.49e+00 mg/ml ; 2.70e-02 mol/l
Class	Very soluble
Log *S* (SILICOS-IT)	-3.40
Solubility	8.03e-02 mg/ml ; 3.95e-04 mol/l
Class	Soluble

Pharmacokinetics	
GI absorption	High
BBB permeant	No
P-gp substrate	No
CYP1A2 inhibitor	No
CYP2C19 inhibitor	No
CYP2C9 inhibitor	No
CYP2D6 inhibitor	No
CYP3A4 inhibitor	No
Log K_p (skin permeation)	-7.48 cm/s

Druglikeness	
Lipinski	Yes; 0 violation
Ghose	Yes
Veber	Yes
Egan	Yes
Muegge	Yes
Bioavailability Score	0.55

Medicinal Chemistry	
PAINS	0 alert
Brenk	3 alerts: cumarine, imine_1, imine_2
Leadlikeness	No; 1 violation: MW<250
Synthetic accessibility	2.72

Compound 35

Molecule 1

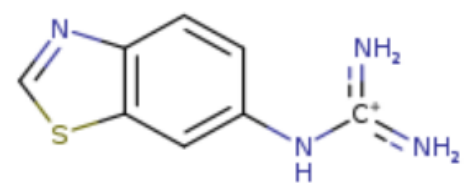

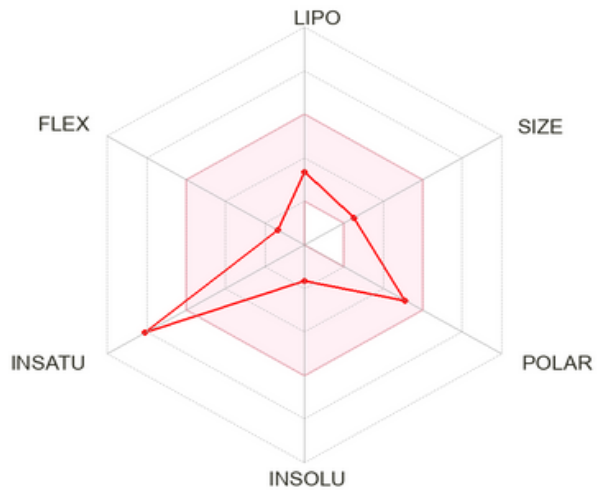

SMILES N[C+](Nc1ccc2c(c1)scn2)N

Physicochemical Properties	
Formula	C8H9N4S+
Molecular weight	193.25 g/mol
Num. heavy atoms	13
Num. arom. heavy atoms	9
Fraction Csp3	0.12
Num. rotatable bonds	2
Num. H-bond acceptors	3
Num. H-bond donors	3
Molar Refractivity	53.83
TPSA	105.20 Å²

Lipophilicity	
Log $P_{o/w}$ (iLOGP)	0.00
Log $P_{o/w}$ (XLOGP3)	0.37
Log $P_{o/w}$ (WLOGP)	0.88
Log $P_{o/w}$ (MLOGP)	0.26
Log $P_{o/w}$ (SILICOS-IT)	0.90
Consensus Log $P_{o/w}$	0.48

Water Solubility	
Log *S* (ESOL)	-1.65
Solubility	4.31e+00 mg/ml ; 2.23e-02 mol/l
Class	Very soluble
Log *S* (Ali)	-2.14
Solubility	1.39e+00 mg/ml ; 7.17e-03 mol/l
Class	Soluble
Log *S* (SILICOS-IT)	-2.36
Solubility	8.51e-01 mg/ml ; 4.41e-03 mol/l
Class	Soluble

Pharmacokinetics	
GI absorption	High
BBB permeant	No
P-gp substrate	No
CYP1A2 inhibitor	No
CYP2C19 inhibitor	No
CYP2C9 inhibitor	No
CYP2D6 inhibitor	No
CYP3A4 inhibitor	No
Log K_p (skin permeation)	-7.22 cm/s

Druglikeness	
Lipinski	Yes; 0 violation
Ghose	Yes
Veber	Yes
Egan	Yes
Muegge	No; 1 violation: MW<200
Bioavailability Score	0.55

Medicinal Chemistry	
PAINS	0 alert
Brenk	1 alert: carbo_cation_anion
Leadlikeness	No; 1 violation: MW<250
Synthetic accessibility	2.21

Molecule 1

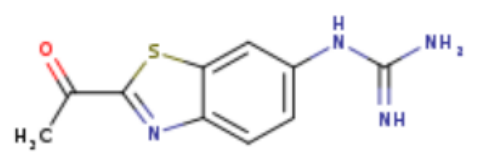

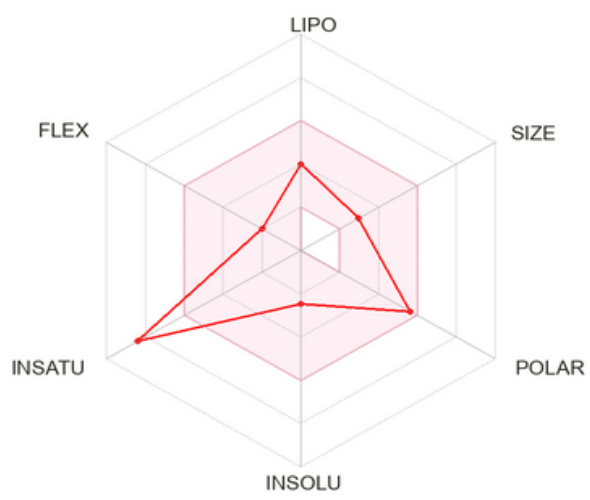

SMILES NC(=N)Nc1ccc2c(c1)sc(n2)C(=O)C

Physicochemical Properties	
Formula	C10H10N4OS
Molecular weight	234.28 g/mol
Num. heavy atoms	16
Num. arom. heavy atoms	9
Fraction Csp3	0.10
Num. rotatable bonds	3
Num. H-bond acceptors	3
Num. H-bond donors	3
Molar Refractivity	65.42
TPSA	120.10 Å²

Lipophilicity	
Log $P_{o/w}$ (iLOGP)	1.24
Log $P_{o/w}$ (XLOGP3)	1.50
Log $P_{o/w}$ (WLOGP)	1.61
Log $P_{o/w}$ (MLOGP)	0.29
Log $P_{o/w}$ (SILICOS-IT)	1.93
Consensus Log $P_{o/w}$	1.32

Water Solubility	
Log *S* (ESOL)	-2.46
Solubility	8.20e-01 mg/ml ; 3.50e-03 mol/l
Class	Soluble
Log *S* (Ali)	-3.63
Solubility	5.49e-02 mg/ml ; 2.35e-04 mol/l
Class	Soluble
Log *S* (SILICOS-IT)	-3.10
Solubility	1.87e-01 mg/ml ; 7.96e-04 mol/l
Class	Soluble

Pharmacokinetics	
GI absorption	High
BBB permeant	No
P-gp substrate	No
CYP1A2 inhibitor	No
CYP2C19 inhibitor	No
CYP2C9 inhibitor	No
CYP2D6 inhibitor	No
CYP3A4 inhibitor	No
Log K_p (skin permeation)	-6.66 cm/s

Druglikeness	
Lipinski	Yes; 0 violation
Ghose	Yes
Veber	Yes
Egan	Yes
Muegge	Yes
Bioavailability Score	0.55

Medicinal Chemistry	
PAINS	0 alert
Brenk	2 alerts: imine_1, imine_2
Leadlikeness	No; 1 violation: MW<250
Synthetic accessibility	2.50

Compound 36

Molecule 1

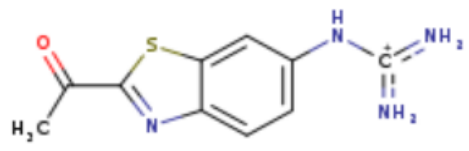

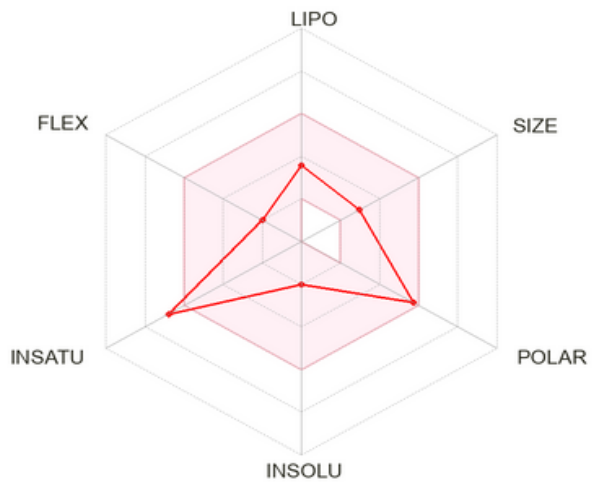

SMILES N[C+](Nc1ccc2c(c1)sc(n2)C(=O)C)N

Physicochemical Properties	
Formula	C10H11N4OS+
Molecular weight	235.29 g/mol
Num. heavy atoms	16
Num. arom. heavy atoms	9
Fraction Csp3	0.20
Num. rotatable bonds	3
Num. H-bond acceptors	4
Num. H-bond donors	3
Molar Refractivity	64.03
TPSA	122.27 Å²

Lipophilicity	
Log $P_{o/w}$ (iLOGP)	0.00
Log $P_{o/w}$ (XLOGP3)	0.76
Log $P_{o/w}$ (WLOGP)	1.08
Log $P_{o/w}$ (MLOGP)	-0.03
Log $P_{o/w}$ (SILICOS-IT)	1.24
Consensus Log $P_{o/w}$	0.61

Water Solubility	
Log *S* (ESOL)	-2.00
Solubility	2.38e+00 mg/ml ; 1.01e-02 mol/l
Class	Very soluble
Log *S* (Ali)	-2.91
Solubility	2.91e-01 mg/ml ; 1.24e-03 mol/l
Class	Soluble
Log *S* (SILICOS-IT)	-2.69
Solubility	4.75e-01 mg/ml ; 2.02e-03 mol/l
Class	Soluble

Pharmacokinetics	
GI absorption	High
BBB permeant	No
P-gp substrate	No
CYP1A2 inhibitor	No
CYP2C19 inhibitor	No
CYP2C9 inhibitor	No
CYP2D6 inhibitor	No
CYP3A4 inhibitor	No
Log K_p (skin permeation)	-7.20 cm/s

Druglikeness	
Lipinski	Yes; 0 violation
Ghose	Yes
Veber	Yes
Egan	Yes
Muegge	Yes
Bioavailability Score	0.55

Medicinal Chemistry	
PAINS	0 alert
Brenk	1 alert: carbo_cation_anion
Leadlikeness	No; 1 violation: MW<250
Synthetic accessibility	2.48

Molecule 1

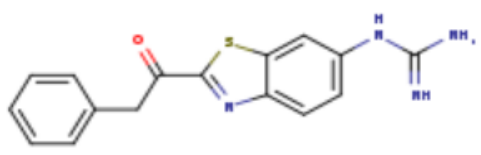

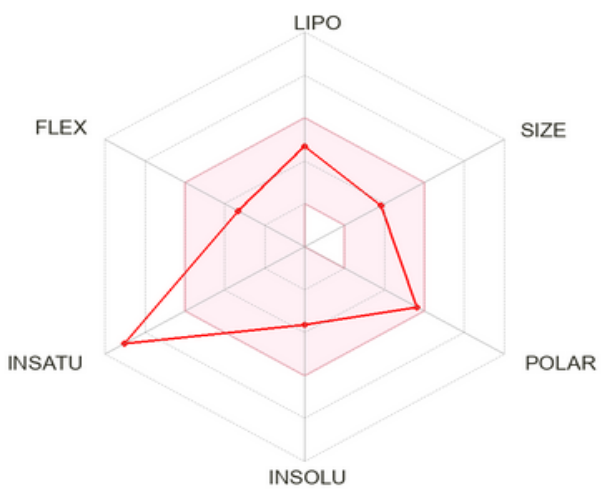

SMILES NC(=N)Nc1ccc2c(c1)sc(n2)C(=O)Cc1ccccc1

Physicochemical Properties	
Formula	C16H14N4OS
Molecular weight	310.37 g/mol
Num. heavy atoms	22
Num. arom. heavy atoms	15
Fraction Csp3	0.06
Num. rotatable bonds	5
Num. H-bond acceptors	3
Num. H-bond donors	3
Molar Refractivity	89.91
TPSA	120.10 Å²

Lipophilicity	
Log $P_{o/w}$ (iLOGP)	1.98
Log $P_{o/w}$ (XLOGP3)	2.68
Log $P_{o/w}$ (WLOGP)	2.84
Log $P_{o/w}$ (MLOGP)	1.68
Log $P_{o/w}$ (SILICOS-IT)	3.41
Consensus Log $P_{o/w}$	2.52

Water Solubility	
Log *S* (ESOL)	-3.63
Solubility	7.32e-02 mg/ml ; 2.36e-04 mol/l
Class	Soluble
Log *S* (Ali)	-4.85
Solubility	4.34e-03 mg/ml ; 1.40e-05 mol/l
Class	Moderately soluble
Log *S* (SILICOS-IT)	-5.61
Solubility	7.60e-04 mg/ml ; 2.45e-06 mol/l
Class	Moderately soluble

Pharmacokinetics	
GI absorption	High
BBB permeant	No
P-gp substrate	No
CYP1A2 inhibitor	Yes
CYP2C19 inhibitor	Yes
CYP2C9 inhibitor	Yes
CYP2D6 inhibitor	No
CYP3A4 inhibitor	Yes
Log K_p (skin permeation)	-6.29 cm/s

Druglikeness	
Lipinski	Yes; 0 violation
Ghose	Yes
Veber	Yes
Egan	Yes
Muegge	Yes
Bioavailability Score	0.55

Medicinal Chemistry	
PAINS	0 alert
Brenk	2 alerts: imine_1, imine_2
Leadlikeness	Yes
Synthetic accessibility	2.84

Compound 37

Molecule 1

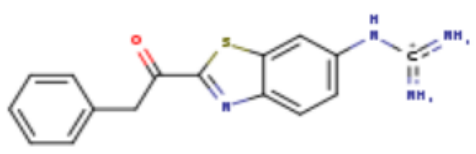

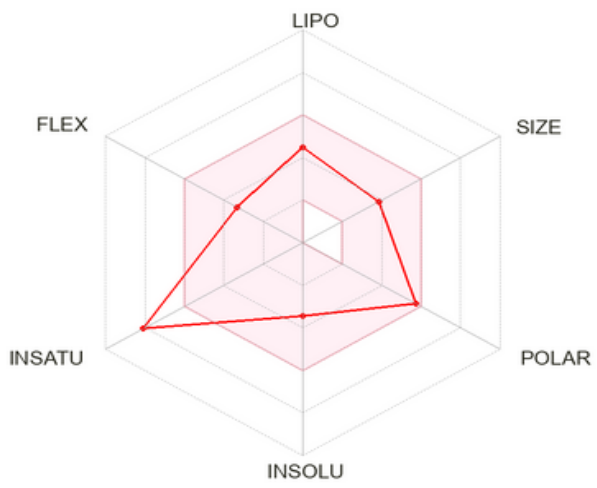

SMILES N[C+](Nc1ccc2c(c1)sc(n2)C(=O)Cc1ccccc1)N

Physicochemical Properties	
Formula	C16H15N4OS+
Molecular weight	311.38 g/mol
Num. heavy atoms	22
Num. arom. heavy atoms	15
Fraction Csp3	0.12
Num. rotatable bonds	5
Num. H-bond acceptors	4
Num. H-bond donors	3
Molar Refractivity	88.51
TPSA	122.27 Å²

Lipophilicity	
Log $P_{o/w}$ (iLOGP)	0.00
Log $P_{o/w}$ (XLOGP3)	2.35
Log $P_{o/w}$ (WLOGP)	2.31
Log $P_{o/w}$ (MLOGP)	1.35
Log $P_{o/w}$ (SILICOS-IT)	2.72
Consensus Log $P_{o/w}$	1.74

Water Solubility	
Log *S* (ESOL)	-3.43
Solubility	1.17e-01 mg/ml ; 3.75e-04 mol/l
Class	Soluble
Log *S* (Ali)	-4.56
Solubility	8.63e-03 mg/ml ; 2.77e-05 mol/l
Class	Moderately soluble
Log *S* (SILICOS-IT)	-5.21
Solubility	1.94e-03 mg/ml ; 6.22e-06 mol/l
Class	Moderately soluble

Pharmacokinetics	
GI absorption	High
BBB permeant	No
P-gp substrate	Yes
CYP1A2 inhibitor	No
CYP2C19 inhibitor	No
CYP2C9 inhibitor	No
CYP2D6 inhibitor	Yes
CYP3A4 inhibitor	Yes
Log K_p (skin permeation)	-6.53 cm/s

Druglikeness	
Lipinski	Yes; 0 violation
Ghose	Yes
Veber	Yes
Egan	Yes
Muegge	Yes
Bioavailability Score	0.55

Medicinal Chemistry	
PAINS	0 alert
Brenk	1 alert: carbo_cation_anion
Leadlikeness	Yes
Synthetic accessibility	2.83

Molecule 1

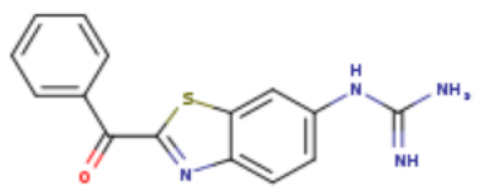

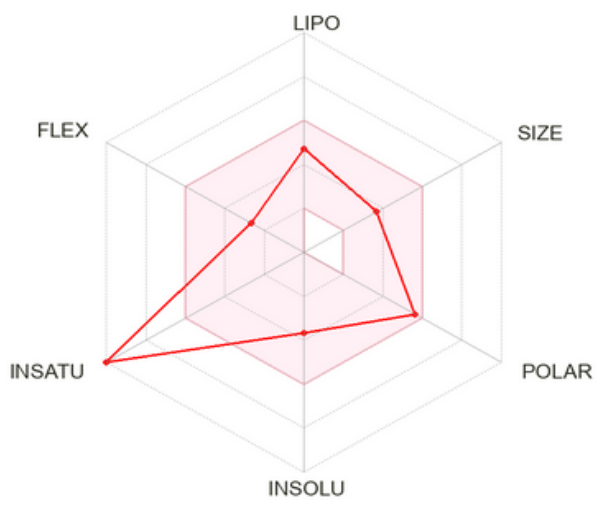

SMILES NC(=N)Nc1ccc2c(c1)sc(n2)C(=O)c1ccccc1

Physicochemical Properties	
Formula	C15H12N4OS
Molecular weight	296.35 g/mol
Num. heavy atoms	21
Num. arom. heavy atoms	15
Fraction Csp3	0.00
Num. rotatable bonds	4
Num. H-bond acceptors	3
Num. H-bond donors	3
Molar Refractivity	85.10
TPSA	120.10 Å²

Lipophilicity	
Log $P_{o/w}$ (iLOGP)	1.18
Log $P_{o/w}$ (XLOGP3)	2.75
Log $P_{o/w}$ (WLOGP)	2.64
Log $P_{o/w}$ (MLOGP)	1.43
Log $P_{o/w}$ (SILICOS-IT)	3.03
Consensus Log $P_{o/w}$	2.21

Water Solubility	
Log *S* (ESOL)	-3.67
Solubility	6.27e-02 mg/ml ; 2.12e-04 mol/l
Class	Soluble
Log *S* (Ali)	-4.93
Solubility	3.51e-03 mg/ml ; 1.18e-05 mol/l
Class	Moderately soluble
Log *S* (SILICOS-IT)	-5.21
Solubility	1.82e-03 mg/ml ; 6.14e-06 mol/l
Class	Moderately soluble

Pharmacokinetics	
GI absorption	High
BBB permeant	No
P-gp substrate	No
CYP1A2 inhibitor	Yes
CYP2C19 inhibitor	No
CYP2C9 inhibitor	No
CYP2D6 inhibitor	No
CYP3A4 inhibitor	No
Log K_p (skin permeation)	-6.16 cm/s

Druglikeness	
Lipinski	Yes; 0 violation
Ghose	Yes
Veber	Yes
Egan	Yes
Muegge	Yes
Bioavailability Score	0.55

Medicinal Chemistry	
PAINS	0 alert
Brenk	2 alerts: imine_1, imine_2
Leadlikeness	Yes
Synthetic accessibility	2.66

Compound 38

Molecule 1

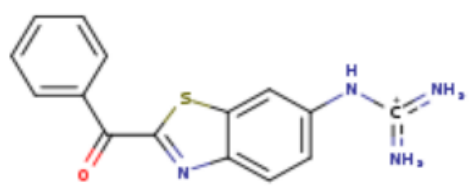

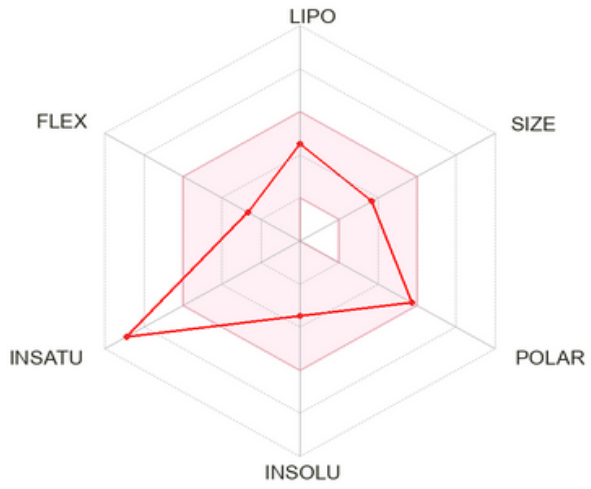

SMILES N[C+](Nc1ccc2c(c1)sc(n2)C(=O)c1ccccc1)N

Physicochemical Properties	
Formula	C15H13N4OS+
Molecular weight	297.35 g/mol
Num. heavy atoms	21
Num. arom. heavy atoms	15
Fraction Csp3	0.07
Num. rotatable bonds	4
Num. H-bond acceptors	4
Num. H-bond donors	3
Molar Refractivity	83.71
TPSA	122.27 Å²

Lipophilicity	
Log $P_{o/w}$ (iLOGP)	0.00
Log $P_{o/w}$ (XLOGP3)	2.42
Log $P_{o/w}$ (WLOGP)	2.11
Log $P_{o/w}$ (MLOGP)	1.10
Log $P_{o/w}$ (SILICOS-IT)	2.34
Consensus Log $P_{o/w}$	1.59

Water Solubility	
Log *S* (ESOL)	-3.47
Solubility	1.00e-01 mg/ml ; 3.37e-04 mol/l
Class	Soluble
Log *S* (Ali)	-4.63
Solubility	6.97e-03 mg/ml ; 2.34e-05 mol/l
Class	Moderately soluble
Log *S* (SILICOS-IT)	-4.81
Solubility	4.63e-03 mg/ml ; 1.56e-05 mol/l
Class	Moderately soluble

Pharmacokinetics	
GI absorption	High
BBB permeant	No
P-gp substrate	Yes
CYP1A2 inhibitor	No
CYP2C19 inhibitor	No
CYP2C9 inhibitor	No
CYP2D6 inhibitor	No
CYP3A4 inhibitor	No
Log K_p (skin permeation)	-6.40 cm/s

Druglikeness	
Lipinski	Yes; 0 violation
Ghose	Yes
Veber	Yes
Egan	Yes
Muegge	Yes
Bioavailability Score	0.55

Medicinal Chemistry	
PAINS	0 alert
Brenk	1 alert: carbo_cation_anion
Leadlikeness	Yes
Synthetic accessibility	2.64

Molecule 1

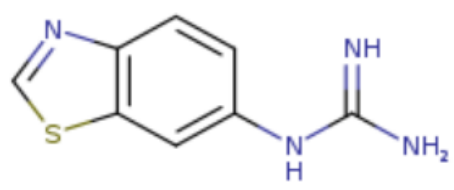

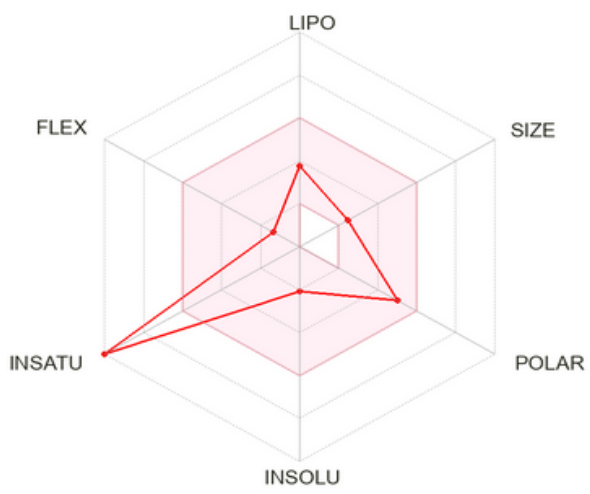

SMILES NC(=N)Nc1ccc2c(c1)scn2

Physicochemical Properties	
Formula	C8H8N4S
Molecular weight	192.24 g/mol
Num. heavy atoms	13
Num. arom. heavy atoms	9
Fraction Csp3	0.00
Num. rotatable bonds	2
Num. H-bond acceptors	2
Num. H-bond donors	3
Molar Refractivity	55.22
TPSA	103.03 Å²

Lipophilicity	
Log $P_{o/w}$ (iLOGP)	1.07
Log $P_{o/w}$ (XLOGP3)	1.07
Log $P_{o/w}$ (WLOGP)	1.41
Log $P_{o/w}$ (MLOGP)	0.58
Log $P_{o/w}$ (SILICOS-IT)	1.59
Consensus Log $P_{o/w}$	1.15

Water Solubility	
Log *S* (ESOL)	-2.09
Solubility	1.58e+00 mg/ml ; 8.20e-03 mol/l
Class	Soluble
Log *S* (Ali)	-2.83
Solubility	2.88e-01 mg/ml ; 1.50e-03 mol/l
Class	Soluble
Log *S* (SILICOS-IT)	-2.76
Solubility	3.35e-01 mg/ml ; 1.74e-03 mol/l
Class	Soluble

Pharmacokinetics	
GI absorption	High
BBB permeant	No
P-gp substrate	No
CYP1A2 inhibitor	No
CYP2C19 inhibitor	No
CYP2C9 inhibitor	No
CYP2D6 inhibitor	No
CYP3A4 inhibitor	No
Log K_p (skin permeation)	-6.71 cm/s

Druglikeness	
Lipinski	Yes; 0 violation
Ghose	Yes
Veber	Yes
Egan	Yes
Muegge	No; 1 violation: MW<200
Bioavailability Score	0.55

Medicinal Chemistry	
PAINS	0 alert
Brenk	2 alerts: imine_1, imine_2
Leadlikeness	No; 1 violation: MW<250
Synthetic accessibility	2.26

Compound 39

Molecule 1

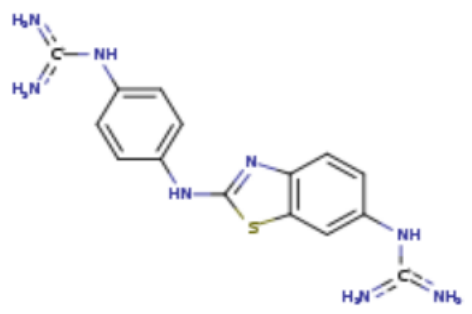

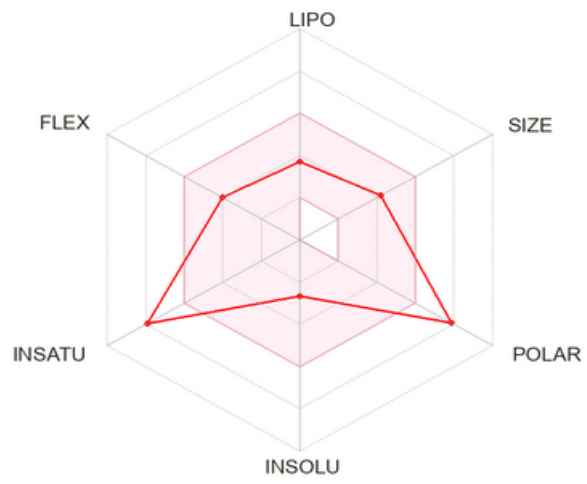

SMILES N[C+](Nc1ccc(cc1)Nc1sc2c(n1)ccc(c2)N[C+](N)N)N

Physicochemical Properties	
Formula	C15H18N8S++
Molecular weight	342.42 g/mol
Num. heavy atoms	24
Num. arom. heavy atoms	15
Fraction Csp3	0.13
Num. rotatable bonds	6
Num. H-bond acceptors	5
Num. H-bond donors	7
Molar Refractivity	97.59
TPSA	181.30 Å²

Lipophilicity	
Log $P_{o/w}$ (iLOGP)	0.00
Log $P_{o/w}$ (XLOGP3)	0.98
Log $P_{o/w}$ (WLOGP)	1.21
Log $P_{o/w}$ (MLOGP)	0.80
Log $P_{o/w}$ (SILICOS-IT)	-0.49
Consensus Log $P_{o/w}$	0.50

Water Solubility	
Log *S* (ESOL)	-2.65
Solubility	7.72e-01 mg/ml ; 2.25e-03 mol/l
Class	Soluble
Log *S* (Ali)	-4.38
Solubility	1.44e-02 mg/ml ; 4.21e-05 mol/l
Class	Moderately soluble
Log *S* (SILICOS-IT)	-4.27
Solubility	1.85e-02 mg/ml ; 5.40e-05 mol/l
Class	Moderately soluble

Pharmacokinetics	
GI absorption	Low
BBB permeant	No
P-gp substrate	No
CYP1A2 inhibitor	Yes
CYP2C19 inhibitor	No
CYP2C9 inhibitor	No
CYP2D6 inhibitor	No
CYP3A4 inhibitor	No
Log K_p (skin permeation)	-7.69 cm/s

Druglikeness	
Lipinski	Yes; 1 violation: NHorOH>5
Ghose	Yes
Veber	No; 1 violation: TPSA>140
Egan	No; 1 violation: TPSA>131.6
Muegge	No; 2 violations: TPSA>150, H-don>5
Bioavailability Score	0.55

Medicinal Chemistry	
PAINS	0 alert
Brenk	1 alert: carbo_cation_anion
Leadlikeness	Yes
Synthetic accessibility	3.21

Molecule 1

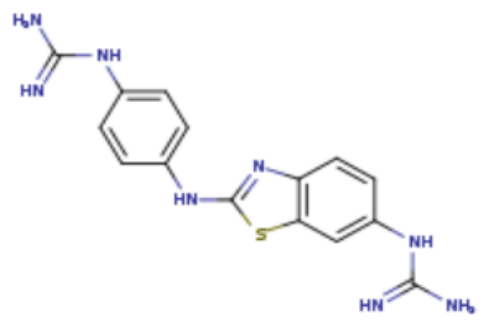

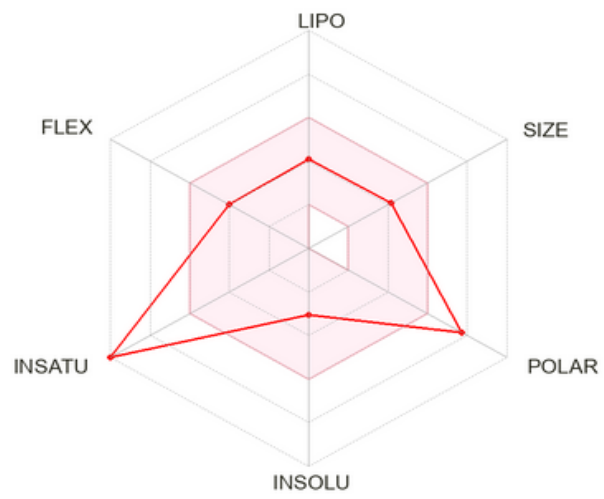

SMILES NC(=N)Nc1ccc(cc1)Nc1sc2c(n1)ccc(c2)NC(=N)N

Physicochemical Properties	
Formula	C15H16N8S
Molecular weight	340.41 g/mol
Num. heavy atoms	24
Num. arom. heavy atoms	15
Fraction Csp3	0.00
Num. rotatable bonds	6
Num. H-bond acceptors	3
Num. H-bond donors	7
Molar Refractivity	100.37
TPSA	176.96 Å²

Lipophilicity	
Log $P_{o/w}$ (iLOGP)	1.03
Log $P_{o/w}$ (XLOGP3)	1.64
Log $P_{o/w}$ (WLOGP)	2.27
Log $P_{o/w}$ (MLOGP)	1.46
Log $P_{o/w}$ (SILICOS-IT)	0.89
Consensus Log $P_{o/w}$	1.46

Water Solubility	
Log *S* (ESOL)	-3.05
Solubility	3.03e-01 mg/ml ; 8.91e-04 mol/l
Class	Soluble
Log *S* (Ali)	-4.97
Solubility	3.65e-03 mg/ml ; 1.07e-05 mol/l
Class	Moderately soluble
Log *S* (SILICOS-IT)	-5.08
Solubility	2.84e-03 mg/ml ; 8.36e-06 mol/l
Class	Moderately soluble

Pharmacokinetics	
GI absorption	Low
BBB permeant	No
P-gp substrate	No
CYP1A2 inhibitor	Yes
CYP2C19 inhibitor	No
CYP2C9 inhibitor	No
CYP2D6 inhibitor	No
CYP3A4 inhibitor	No
Log K_p (skin permeation)	-7.21 cm/s

Druglikeness	
Lipinski	Yes; 1 violation: NHorOH>5
Ghose	Yes
Veber	No; 1 violation: TPSA>140
Egan	No; 1 violation: TPSA>131.6
Muegge	No; 2 violations: TPSA>150, H-don>5
Bioavailability Score	0.55

Medicinal Chemistry	
PAINS	0 alert
Brenk	2 alerts: imine_1, imine_2
Leadlikeness	Yes
Synthetic accessibility	3.18

Compound 40

Molecule 1

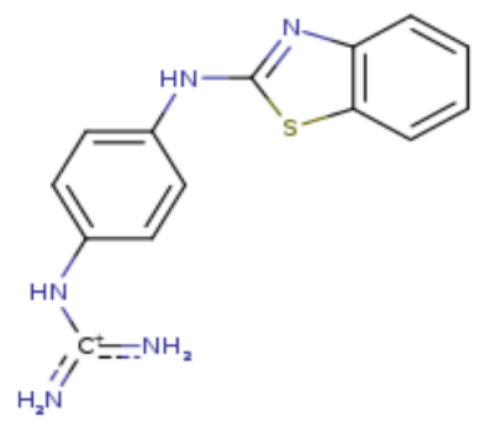

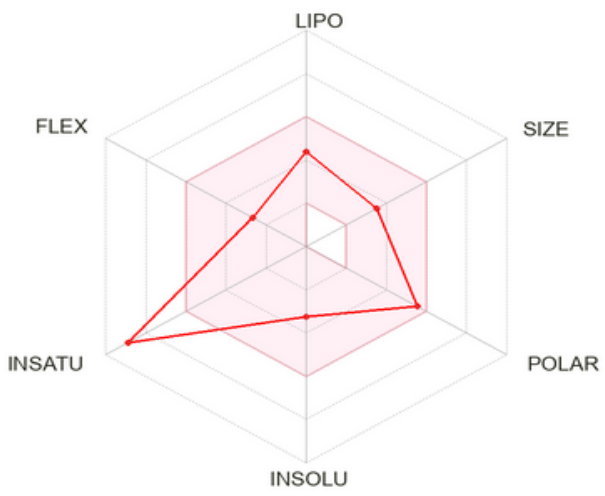

SMILES N[C+](Nc1ccc(cc1)Nc1nc2c(s1)cccc2)N

Physicochemical Properties	
Formula	C14H14N5S+
Molecular weight	284.36 g/mol
Num. heavy atoms	20
Num. arom. heavy atoms	15
Fraction Csp3	0.07
Num. rotatable bonds	4
Num. H-bond acceptors	3
Num. H-bond donors	4
Molar Refractivity	83.38
TPSA	117.23 Å²

Lipophilicity	
Log $P_{o/w}$ (iLOGP)	0.00
Log $P_{o/w}$ (XLOGP3)	2.17
Log $P_{o/w}$ (WLOGP)	2.63
Log $P_{o/w}$ (MLOGP)	1.86
Log $P_{o/w}$ (SILICOS-IT)	1.65
Consensus Log $P_{o/w}$	1.66

Water Solubility	
Log *S* (ESOL)	-3.26
Solubility	1.56e-01 mg/ml ; 5.48e-04 mol/l
Class	Soluble
Log *S* (Ali)	-4.26
Solubility	1.55e-02 mg/ml ; 5.43e-05 mol/l
Class	Moderately soluble
Log *S* (SILICOS-IT)	-4.92
Solubility	3.45e-03 mg/ml ; 1.21e-05 mol/l
Class	Moderately soluble

Pharmacokinetics	
GI absorption	High
BBB permeant	No
P-gp substrate	Yes
CYP1A2 inhibitor	Yes
CYP2C19 inhibitor	No
CYP2C9 inhibitor	No
CYP2D6 inhibitor	No
CYP3A4 inhibitor	Yes
Log K_p (skin permeation)	-6.49 cm/s

Druglikeness	
Lipinski	Yes; 0 violation
Ghose	Yes
Veber	Yes
Egan	Yes
Muegge	Yes
Bioavailability Score	0.55

Medicinal Chemistry	
PAINS	0 alert
Brenk	1 alert: carbo_cation_anion
Leadlikeness	Yes
Synthetic accessibility	2.76

Molecule 1

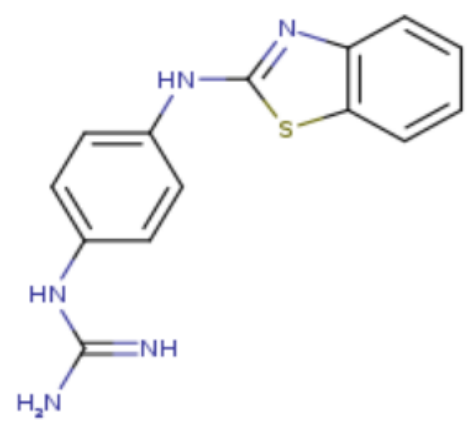

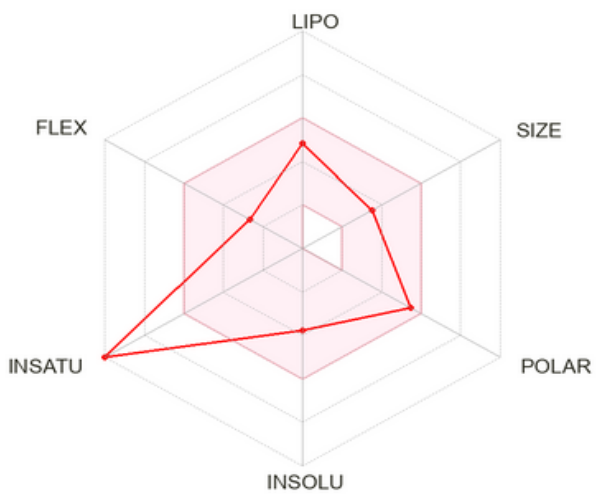

SMILES NC(=N)Nc1ccc(cc1)Nc1nc2c(s1)cccc2

Physicochemical Properties	
Formula	C14H13N5S
Molecular weight	283.35 g/mol
Num. heavy atoms	20
Num. arom. heavy atoms	15
Fraction Csp3	0.00
Num. rotatable bonds	4
Num. H-bond acceptors	2
Num. H-bond donors	4
Molar Refractivity	84.77
TPSA	115.06 Å²

Lipophilicity	
Log $P_{o/w}$ (iLOGP)	1.70
Log $P_{o/w}$ (XLOGP3)	2.95
Log $P_{o/w}$ (WLOGP)	3.15
Log $P_{o/w}$ (MLOGP)	2.19
Log $P_{o/w}$ (SILICOS-IT)	2.34
Consensus Log $P_{o/w}$	2.47

Water Solubility	
Log *S* (ESOL)	-3.75
Solubility	5.08e-02 mg/ml ; 1.79e-04 mol/l
Class	Soluble
Log *S* (Ali)	-5.03
Solubility	2.65e-03 mg/ml ; 9.36e-06 mol/l
Class	Moderately soluble
Log *S* (SILICOS-IT)	-5.32
Solubility	1.35e-03 mg/ml ; 4.78e-06 mol/l
Class	Moderately soluble

Pharmacokinetics	
GI absorption	High
BBB permeant	No
P-gp substrate	No
CYP1A2 inhibitor	Yes
CYP2C19 inhibitor	Yes
CYP2C9 inhibitor	No
CYP2D6 inhibitor	No
CYP3A4 inhibitor	Yes
Log K_p (skin permeation)	-5.93 cm/s

Druglikeness	
Lipinski	Yes; 0 violation
Ghose	Yes
Veber	Yes
Egan	Yes
Muegge	Yes
Bioavailability Score	0.55

Medicinal Chemistry	
PAINS	0 alert
Brenk	2 alerts: imine_1, imine_2
Leadlikeness	Yes
Synthetic accessibility	2.76

S8. References

1 Glide: A New Approach for Rapid, Accurate Docking and Scoring. 1. Method and Assessment of Docking Accuracy | Journal of Medicinal Chemistry ; Extra Precision Glide: Docking and Scoring Incorporating a Model of Hydrophobic Enclosure for Protein−Ligand Complexes | Journal of Medicinal Chemistry

2 Docking Covalent Inhibitors: A Parameter Free Approach To Pose Prediction and Scoring | Journal of Chemical Information and Modeling

3 W. Sherman, T. Day, M. P. Jacobson, R. A. Friesner, R. Farid, "Novel procedure for modeling ligand/receptor induced fit effects" J. Med. Chem. 2006, 49, 534-553

4 Epik: pKa and Protonation State Prediction through Machine Learning - PubMed

5 Protein and ligand preparation: parameters, protocols, and influence on virtual screening enrichments | Journal of Computer-Aided Molecular Design | Springer Nature Link
